# Three-Component Suzuki–Knoevenagel Synthesis of Merocyanine Libraries and Correlation Analyses of Their Oxidation Potentials and Optical Band Gaps

**DOI:** 10.3390/molecules26175149

**Published:** 2021-08-25

**Authors:** Tim Meyer, Roxanne Krug, Thomas J. J. Müller

**Affiliations:** Institut für Organische Chemie und Makromolekulare Chemie, Heinrich-Heine-Universität Düsseldorf, Universitätsstrasse 1, D-40225 Düsseldorf, Germany; tim-meyer@outlook.com (T.M.); roxanne.krug@web.de (R.K.)

**Keywords:** absorption, aldol condensation, correlation analysis, cross-coupling reactions, cyclic voltammetry, fluorescence

## Abstract

The Suzuki coupling Knoevenagel condensation one-pot synthesis of boronic acids/esters, (hetero)aromatic bromo aldehydes and methylene active compounds is a highly practical consecutive three-component process to provide substance libraries with 60 donor-π-bridge-acceptor molecules, i.e., merocyanines in a broader sense, in moderate to excellent yield. As already seen with the naked eye, a broad variation of the optical properties becomes accessible using this practical synthetic tool. More systematically, correlation analyses upon plotting the optical band gaps against the first oxidation potentials of redox active systems of consanguineous series furnishes linear correlations and, by extension, two parameter plots (oxidation potential and emission maximum) planar correlations with the optical band gaps.

## 1. Introduction

Multicomponent reactions (MCR) [1,2,3,4], conducted in domino, sequential or consecutive fashion, represent a powerful, efficient and efficacious tool for constructing complex molecular scaffolds in a one-pot fashion. In recent years, we have developed and explored MCRs as concise entries to functional chromophores. In particular, for fluorophores and electrophores [5,6,7], MCRs are most fruitfully applied in the sense of a chromogenic approach, which results directly in the formation of the functional chromophore of interest.

Among functional chromophores, merocyanines [8,9,10,11,12,13] are particularly interesting. In a very general sense, merocyanines can be structurally characterized as donor-π-bridge-acceptor scaffolds with high extinction coefficients, and as chromophores they are omnipresent in optoelectronics [14,15,16,17], organic semiconductors [18] and organic photovoltaics (OPV) [19]. Due to their inherent dipolar nature, the self-assembly of merocyanines in solution even allows for concise access to nanoscale objects and supramolecular materials [20,21]. The classical synthesis of merocyanines, as for many polymethine dyes, is governed by conventional aldol or Knoevenagel condensations [8,22,23,24].

Besides MCR syntheses of indolone [25,26,27], aroyl [28,29] and coumarin [30]-based merocyanines, we just recently communicated the versatile access to DSSC (dye sensitized solar cell) merocyanines bearing carboxylic acid end groups for immobilization on TiO_2_ by a consecutive three-component Suzuki–Knoevenagel condensation sequence [31].

Since merocyanines are outstandingly important in OPV [19], the effect of acceptor strength in molecular architectures on the morphology of bulk-heterojunction fullerene-free solar cells [32] as well as their theory-based definition of donor and acceptor strength in conjugated copolymers [33] are of general relevance. Therefore, we assumed that a diversity oriented one-pot methodology could not only provide libraries of various consanguineous series of merocyanines, but also could provide access to systematic correlation analyses. Here, we present the methodological extension of our recently reported consecutive Suzuki–Knoevenagel three-component synthesis to a broad range of merocyanines, their electronic data by photophysics (absorption and emission spectroscopy) and cyclic voltammetry, as well as several established structure–property relationships by correlation analyses between the compounds’ oxidation potentials and their optical band gaps.

## 2. Results and Discussion

### 2.1. Synthesis

**Scheme 1:**
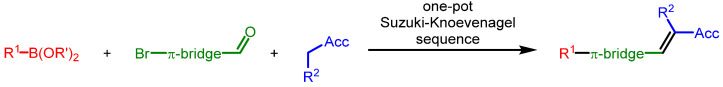
Multicomponent synthetic concept based upon a Suzuki–Knoevenagel sequence.

Recently, we have established the one-pot Suzuki–Knoevenagel condensation (SuKnoCon) sequence for synthesizing carboxylic acid functionalized merocyanines with tunable donors for DSSC studies [31]. We reasoned that this general, straightforward principle of concatenating Suzuki arylation and Knoevenagel condensation in a consecutive three-component fashion can be particularly favorable for accessing substance libraries of dyes for establishing structure-property relationships based upon correlation analysis (Scheme 1).

**Scheme 2:**
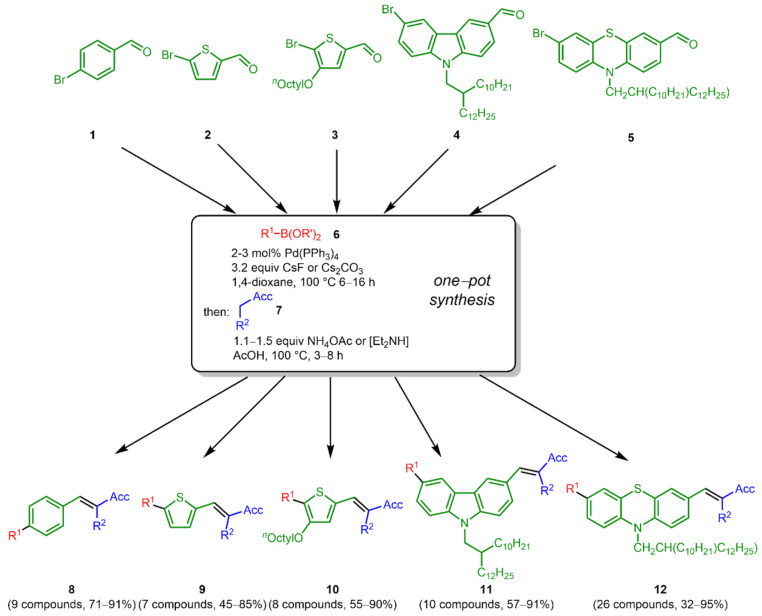
Consecutive three-component Suzuki–Knoevenagel synthesis of merocyanines **8**–**12**.

**Scheme 3:**
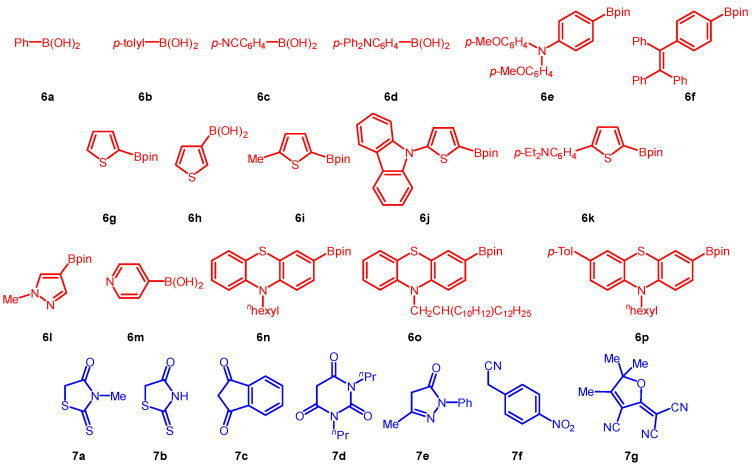
(Hetero)aryl boronates or boronic acids **6** (red) and methylene active compounds **7** (blue) employed in consecutive three-component Suzuki–Knoevenagel syntheses.

**Scheme 4:**
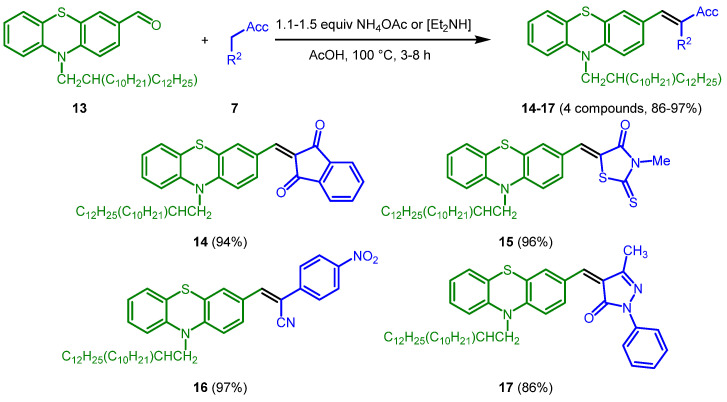
Knoevenagel synthesis of merocyanines **14**–**17**.

As a central bifunctional relay, we chose five different types of bromo aldehydes bearing the two prerequisite functionalities for Suzuki arylation and Knoevenagel condensation and representing π-bridges ranging from *p*-phenylene (**1**) over 2,5-thienylene (**2**) to 4-ocyl 2,5-thienyl (**3**), 3,6-carbazolylene (**4**) and 3,7-phenazinylene (**5**). While the first two possess high lying oxidation potentials, the latter three are increasingly easy to oxidize. The conditions from the sequences were essentially transposed from our previous synthetic study on DSSC dyes [31]. Upon consecutive three-component Suzuki–Knoevenagel synthesis of bromo aldehydes **1**–**5** with (hetero)aryl boronates or boronic acids **6** and methylene active compounds **7** in a one-pot fashion five different libraries of merocyanines **8**–**12**, in total 59 compounds, were efficiently synthesized in largely good to excellent yield (Scheme 2). Besides the bromo aldehydes **1**–**5** the diversity of the libraries relies on sixteen (hetero)aryl boronates or boronic acids **6** and seven methylene active compounds **7** (Scheme 3). While the boronic acid derivatives are electro neutral (**6a**, **6f**, **6g**, **6h**, and **6i**), electron deficient (**6c** and **6m**) and mostly electron-rich (**6b**, **6d**, **6e**, **6j**–**6l**, **6n**–**6p**), the employed methylene active compounds **7** furnish variably strong electron acceptors upon Knoevenagel condensation. All new compounds **8**–**12**, as well as their electronic reference systems **14**–**17** (vide infra, Scheme 4), were unambiguously assigned in their structure by ^1^H and ^13^C NMR spectroscopy, IR spectroscopy and mass spectrometry. The elemental compositions were confirmed by combustion analyses.

**Chart 1:**
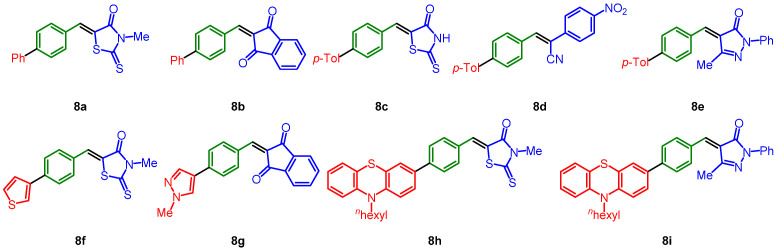
*p*-Phenylene-bridged systems **8**.

**Chart 2:**
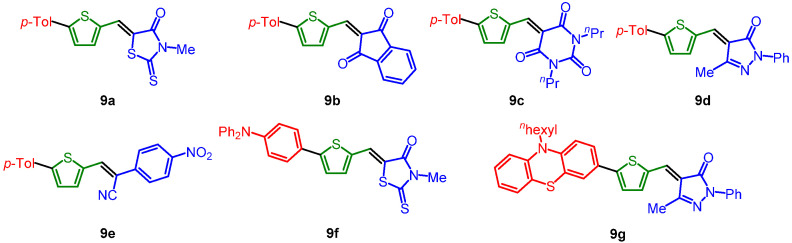
Thienylene-bridged systems **9**.

**Chart 3:**
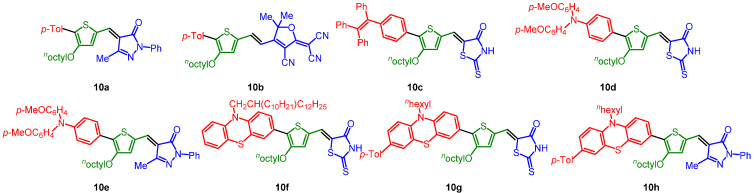
4-Octyl thienylene-bridged systems **10**.

**Chart 4:**
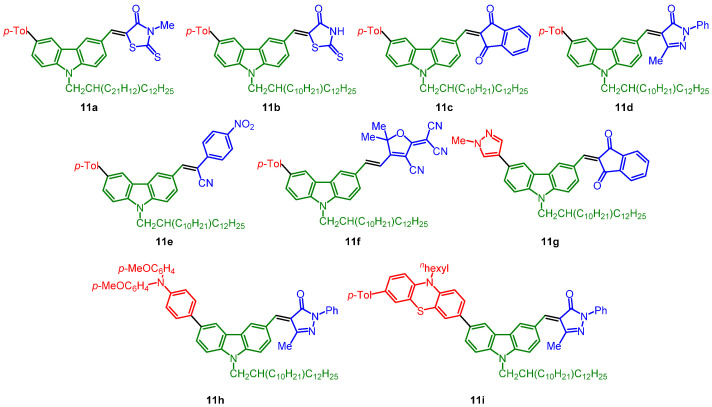
Carbazole-bridged systems **11**.

**Chart 5:**
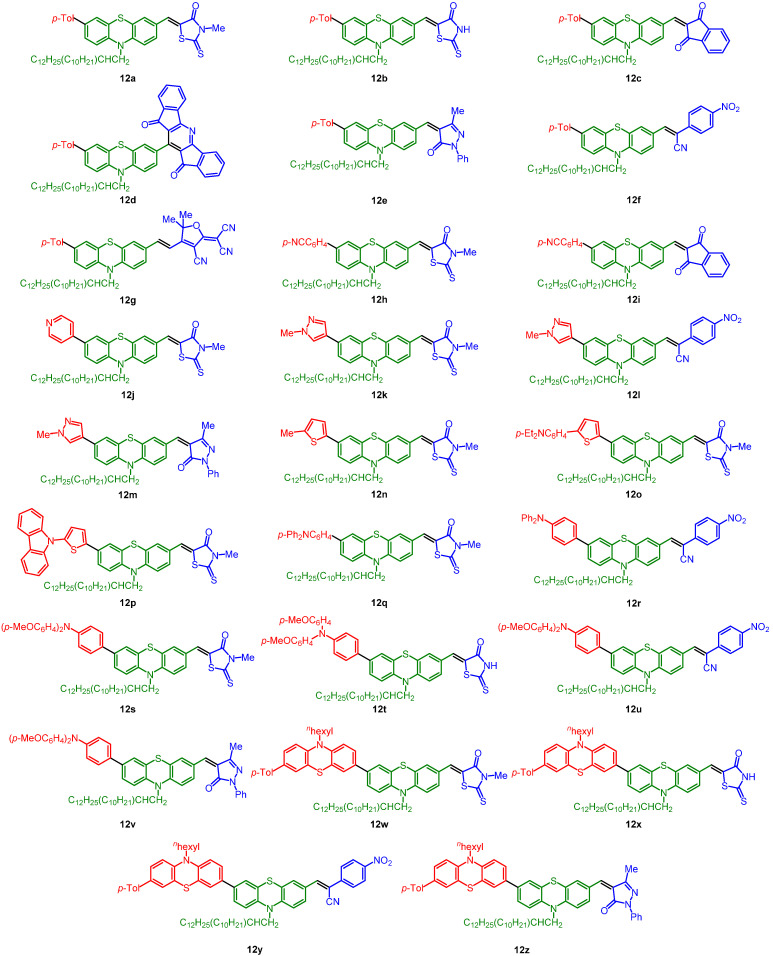
Phenothiazine-bridged systems **12**.

The obtained five libraries of *p*-phenylene-bridged (**8**), thienylene-bridged (**9**), 4-octyl thienylene-bridged (**10**), carbazole-bridged systems (**11**), and phenothiazine-bridged (**12**) systems containing the merocyanine typical donor-π-bridge-acceptor motif are depicted in Chart 1, Chart 2, Chart 3, Chart 4 and Chart 5.

For electronic comparison and for establishing acceptor parameters (vide infra) phenothiazine aldehyde **13** was reacted by Knoevenagel condensation with methylene active compounds **7a**, **7c**, **7e**, and **7f** to give merocyanines **14**–**17** in excellent yield (Scheme 4).

### 2.2. Electronic Characteristics

The electronic properties of the merocyanines **8**–**12** were determined by recording the absorption and emission spectra as well as the cyclic voltammograms in the anodic regions (oxidations) (Table 1). While oxidation potentials reflect a property of the electronic ground state of the chromophores, their absorption and emission behavior correlate to the photonic transitions from the electronic ground to the excited states and back. The oxidation potential of the compounds predominantly depends on the presence of a readily oxidizable moiety in the π-bridge, such as 4-octyl thienyl, carbazole or phenothiazine units, or in the substituent R^1^. As in our previous study [31], we calculated the half-wave potentials *E*_1/2_ referenced against the normal hydrogen electrode by adding 0.2 V to the measured value of *E*_0_ (against Ag/AgCl). The first oxidations were mostly obtained as electrochemically reversible waves in a range from 0.79 to 1.71 V. For irreversible potentials, only the anodic peak potential was documented. The obtained merocyanines display longest wavelength absorption maxima *λ*_max,abs_ covering a broad part of the *UV*/*V* is spectrum ranging from 367 to 580 nm. As seen from the molar decadic extinction coefficients, these transitions are quite intense and account to charge transfer character from the donor to the acceptor moiety, as previously corroborated for phenothiazine-based DSSC merocyanines [31]. In addition, most of the compounds reveal emission maxima *λ*_max,em_ ranging from 412 to 668 nm, which were not quantified due to very variable intensity. Nevertheless, Stokes shifts Δ*ṽ*, i.e., energy differences between longest wavelength absorption maxima and emission maxima, as an indicator of changes in the electronic structure upon photonic excitation from the ground state to the vibrationally relaxed excited states were calculated in a range between 1200 and 8000 cm^−1^ (0.147–0.990 eV). In addition, from the absorption and emission maxima the optical band gap, i.e., the *E*_0-0_ transition, was estimated by the arithmetic average of the corresponding and ranging from 2.083 to 3.197 eV. 

### 2.3. Correlation Analyses of the Electronic Properties

**Figure 1:**
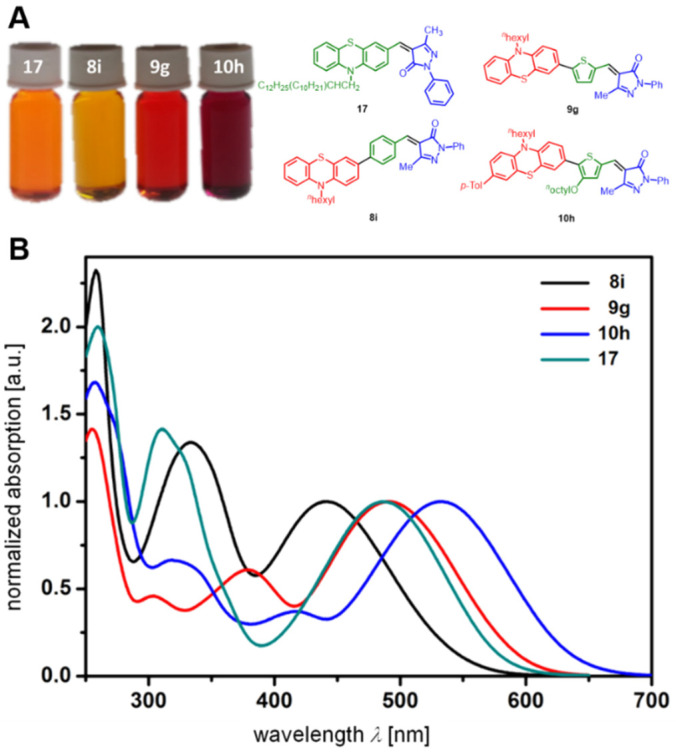
Comparison of four phenothiazine-*N*-phenyl methyl pyrazolone merocyanines with variable π-bridging. (**A**) Solutions of compounds **17**, **8i**, **9g** and **10h** (from left to right) under daylight (*c* = 10^−3^ m). (**B**) Normalized UV/Vis spectra of compounds **17**, **8i**, **9g** and **10h** (recorded in dichloromethane, *c* = 10^−3^ m, *T* = 298 K).

The extensive merocyanine libraries **8**–**12** encouraged us to take a closer look on a semiempirical physical organic correlation analysis. Correlations between clearly assignable oxidation potentials and *E*_0-0_ transitions should provide an insight into structure-property relationships of this class of compounds. Already visible to the naked eye, the nature of the π-bridge between donor and acceptor moieties, as seen for four phenothiazine-*N*-phenyl methyl pyrazolone merocyanines **17**, **8i**, **9g** and **10h**, causes significant differences in the electronic properties (Figure 1).

For establishing a set of structure-property relationships we considered four classes of acceptors, i.e., 3-methyl-4-oxo-2-thioxothiazolidin-5-ylidene (from condensation of **7a**), 1,3-dioxo-1,3-dihydro-2*H*-inden-2-ylidene (from condensation of **7c**), 3-methyl-5-oxo-1-phenyl-1,5-dihydro-4*H*-pyrazol-4-ylidene (from condensation of **7e**), cyano(4-nitrophenyl)-methylene (from condensation of **7f**), and four classes of donor bridge systems of the merocyanine series **9** (5-(*p*-tolyl)thien-2-yl)), **11** (6-(*p*-tolyl)carbzol-3-yl)), **12** (7-(*p*-tolyl)phenothiazin-3-yl) as well as the reference chromophores **14**–**17** (phenothiazin-3-yl) (Table 2).

**Figure 2:**
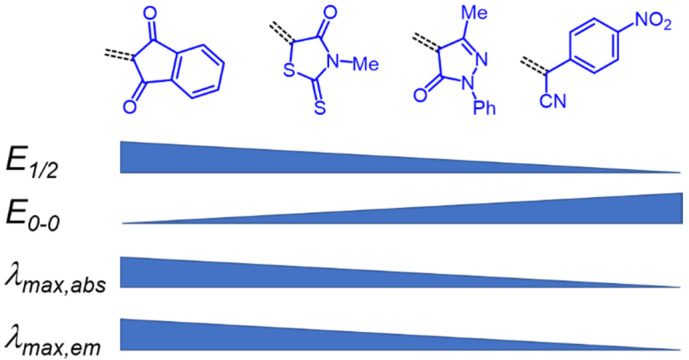
Qualitative acceptor strength decreasing from left to right and their influence on selected electronic data (*E*_1/2_, *E*_0-0_, *λ*_max,abs_ and *λ*_max,em_).

A qualitative look at the electronic data (*E*_1/2_, *λ*_max,abs_, *λ*_max,em_, Stokes shift Δ*ṽ*, and *E*_0-0_) shows that the oxidation potential *E*_1/2_ reflecting the donor strength is affected by the acceptor strength and a qualitative alignment acceptors with decreasing strength concomitantly correlates with an increasing optical band gap *E*_0-0_ (Figure 2). In the same trend absorption and emission bands are blue shifted (to shorter wavelength) with decreasing acceptor strength. For the unperturbed reference merocyanines **14**–**17**, the plots of *λ*_max,abs_, *λ*_max,em_ and *E*_0-0_ (as energies in eV) against *E*_1/2_ (in Volt) give reasonably good linear correlations (r^2^ > 0.90) (for details on correlation analyses, see Supplementary Material). Since the series **9**, **11** and **12**, maintaining the constant donor moiety while varying the acceptor parts, give poor correlations, we instead considered variations of the donor parts while maintaining the corresponding acceptors constant as grouped in Table 2.

The consanguineous acceptor series of 3-methyl-4-oxo-2-thioxothiazolidin-5-ylidene, 1,3-dioxo-1,3-dihydro-2H-inden-2-ylidene, 3-methyl-5-oxo-1-phenyl-1,5-dihydro-4H-pyrazol-4-ylidene and cyano(4-nitrophenyl)-methylene derivatives gave good to excellent linear correlations for the plots of *λ*_max,abs_, *λ*_max,em_, Stokes shift Δ*ṽ* and *E*_0-0_ (as energies in eV) against *E*_1/2_ (in Volt) (r^2^ > 0.90) (for details on correlation analyses, see Supplementary Material). In particular, the excellent linear correlations (r^2^ = 0.943–0.999) between the oxidation potential *E*_1/2_ and the optical band gap *E*_0-0_ (Table 3)) suggest that the electronic ground state property, i.e., oxidation potential, affects the photonic property, i.e., band gap, and could lead to a more general correlation beyond consanguineous chromophore series. Therefore, we expanded the basis of chromophores to a total of 24 (for correlation analyses, see Supplementary Information). An attempt to establish a direct relation between *E*_1/2_ and *E_00_* gave only a poor linear correlation (r^2^ = 0.7338).

**Figure 3:**
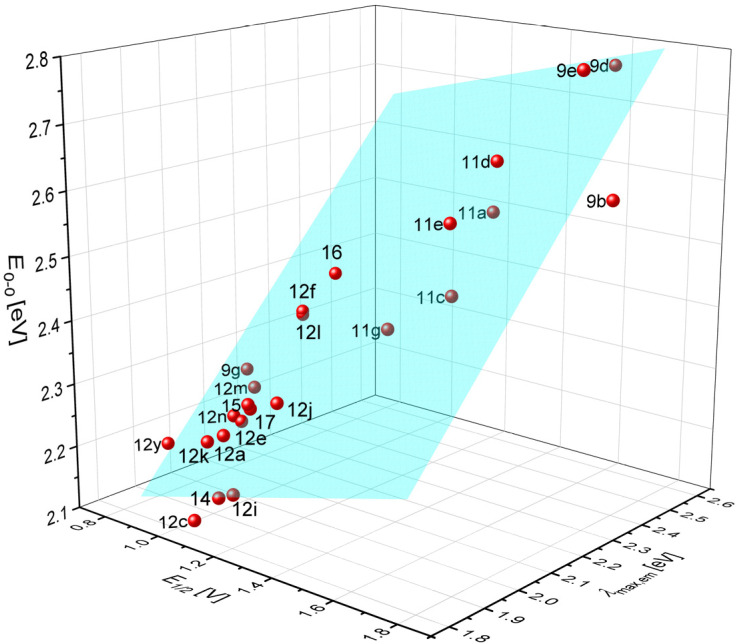
Correlation of the optical band gap *E*_0-0_ vs. oxidation potential *E*_1/2_ and emission maximum *λ*_max,em_: *E*_0-0_ = 0.147·*E*_1/2_ + 0.7019·*λ*_max,em_ + 0.7296; r^2^ = 0.93504.

However, plotting *E*_0-0_ vs. the two parameters *E*_1/2_ and *λ*_max,em_ representing both ground and excited state energetics give a quite good planar correlation (r^2^ = 0.93504), were the slopes indicate that the emission *λ*_max,em_ contributes to a larger extent than the oxidation potentials (Figure 3). While the oxidation potential represents an electronic ground state parameter the emission, mostly resulting from radiative deactivation of the vibrationally relaxed excited state S_1_ depends on the electronic structure of the excited state. For merocyanines typical are highly polar excited states, which attribute for a distinct degree of charge transfer character. This planar correlation now allows for a series of merocyanines to predict optical band gaps from first oxidation potentials and emission maxima.

## 3. Materials and Methods

All synthetic details on the preparation as well as the ^1^H and ^13^C NMR spectra of the series **8**, **9**, **10**, **11** and **12**, and the reference chromophores **14**–**17** are compiled in the Supporting Information.

### Synthesis of Compound ***9d*** by Coupling-Condensation One-Pot Synthesis (Typical Procedure)

4-Methylphenylboronic acid (**6b**) (150 mg, 1.10 mmol), 5-bromothiophene-2-carbaldehyde (**2**) (190 mg, 1.00 mmol), cesium fluoride (486 mg, 3.20 mmol) and tetrakis(triphenylphosphane)palladium(0) (24 mg, 0.02 mmol) were placed in a Schlenk flask with magnetic stir bar under nitrogen and dry 1,4-dioxane (4 mL) were added. The solution was heated to 100 °C under reflux for 8 h. After cooling to room temp acetic acid (2 mL), 5-methyl-2-phenyl-2,4-dihydro-3*H*-pyrazol-3-one (**7e**) (192 mg, 1.10 mmol), and 1 drop of diethylamine was added to the reaction mixture. This mixture was heated under nitrogen to 95 °C under reflux for 4 h. An intensive orange red solution was formed. After cooling to room temp the reaction mixture was diluted with dichloromethane (30 mL) and the organic layer was washed with distilled water until the aqueous phase did not smell similar to acetic acid. The combined aqueous phases were extracted with dichloromethane and the combined organic layers were dried (anhydrous magnesium sulfate) and the solvents were removed in vacuo. The residue was adsorbed on celite® and purified by flash chromatography on silica gel (*n*-hexane/acetone 10:1) to furnish after drying under vacuo compound **9d** (290 mg, 81%) as a red solid, Mp 133 °C. R*_f_* (*n*-hexane/acetone 4:1) = 0.27.

^1^H NMR (600 MHz, acetone-d_6_/CS_2_): *δ* 2.35 (s, 3 H), 2.41 (s, 3 H), 7.41 (tt, ^3^*J* = 7.4 Hz, ^4^*J* = 1.1 Hz, 1 H), 7.27–7.31 (m, 2 H), 7.37–7.42 (m, 2 H), 7.56 (d, ^3^*J* = 4.0 Hz, 1 H), 7.68–7.72 (m, 2 H), 7.86 (s, 1 H), 8.05–8.08 (m, 2 H), 8.11 (d, ^3^*J* = 4.0 Hz, 1 H). ^13^C NMR (150 MHz, acetone-d_6_/CS_2_): *δ* 13.3 (CH_3_), 21.6 (CH_3_), 118.7 (CH), 122.1 (C_quat_), 124.7 (CH), 124.9 (CH), 127.1 (CH), 129.3 (CH), 130.7 (CH), 131.6 (C_quat_), 136.7 (C_quat_), 137.2 (CH), 139.9 (C_quat_), 140.3 (C_quat_), 143.7 (CH), 150.7 (C_quat_), 157.4 (C_quat_), 162.9 (C_quat_). MS (MALDI-TOF) calcd for C_22_H_18_N_2_OS *m/z:* 358.11; Found: 359.1 ([MH]^+^). IR: *ṽ* [cm^−1^] = 2980 (w), 2972 (w), 2918 (w), 2851 (w), 1678 (s), 1653 (w), 1593 (m), 1557 (m), 1500 (m), 1489 (m), 1431 (m), 1410 (m), 1375 (m), 1360 (m), 1335 (w), 1314 (m), 1304 (m), 1255 (w), 1213 (m), 1142 (m), 1096 (w), 1078 (m), 1024 (m), 1001 (m), 957 (w), 926 (m), 912 (w), 891 (w), 804 (s), 797 (s), 762 (m), 756 (s), 729 (w), 689 (s), 673 (m), 658 (m), 619 (w). Anal calcd for C_22_H_18_N_2_OS [358.5]: C 73.71, H 5.06, N 7.82, S 8.95; Found: C 73.63, H 5.09, N 7.86, S 8.98. 

## 4. Conclusions

In summary we have elaborated a concise Suzuki coupling Knoevenagel condensation one-pot synthesis of boronic acids/esters, (hetero)aromatic bromo aldehydes and methylene active compounds in the sense of a consecutive three-component process giving rise to the formation products that are merocyanines due to the donor nature of the bromo aldehydes or the Suzuki intermediates in moderate to excellent yield. This synthetic one-pot synthesis is so general that it can be applied as a tool to access huge substance libraries suitable for hit screening and lead finding. As examples, we conducted correlation analyses with consanguineous series by plotting their optical band gap (determined from the intersection of absorption and emission spectra) against the first oxidation potentials (determined by cyclic voltammetry). Indeed, linear correlations could be established for consanguineous of systems with varying the donor part with the same acceptor part. In the sense of a two parameter planar correlation for 24 representatives of four different series a common two parameter planar correlation was found upon plotting the optical band gap energy *E*_0-0_ against the oxidation potential *E*_1/2_ and the emission maximum *λ*_max,em_. Both the synthetic concept warranting rapid access to substance libraries and experimentally-based correlation analyses of consanguineous series, and also of combinations of several series, set the stage for establishing extensive structure–property relationships of chromophores. Further studies are currently underway.

## Figures and Tables

**Table 1:** Selected electronic properties of Merocyanines **8**–**12** (recorded in CH_2_Cl_2_, *T* = 298 K).

Compound	*E*_1/2_ vs. NHE	*λ*_max,abs_	*λ*_max,em_	Stokes Shift ∆*ṽ*	*E*_0-0_
	[V] ^1^	[nm] (*ε* [m^−1^cm^−1^])	[nm] ^2^	[cm^−1^] ([eV]) ^3^	[eV] ^4^
**8a**	>2.00	293 (18,000), 396 (42,000)	451	3100 (0.382)	2.943
**8b**	>2.00	261 (30,000), 302 (20,000), 384 (24,000), 438 (26,000)	462	1200 (0.147)	2.760
**8c**	>2.00	293 (14,000), 398 (44,000)	470	3900 (0.478)	2.879
**8d**	>2.00	294 (15,000), 364 (23,000)	513	8000 (0.990)	2.914
**8e**	1.57 (irrev.)	250 (25,000), 367 (35,000)	412	3000 (0.369)	3.197
**8f**	>2.00	296 (15,000), 402 (38,000)	463	3300 (0.407)	2.820
**8g**	1.55 (irrev.)	258 (22,000), 265 (21,000), 403 (38,000)	486	4200 (0.526)	2.866
**8h**	0.95, 1.66	261 (30,000), 302 (20,000), 384 (24,000), 438 (26,000)	555	4800 (0.597)	2.535
**8i**	0.93, 1.61	259 (35,000), 334 (20,000), 442 (15,000)	561	4800 (0.596)	2.510
**9a**	1.60 (irrev.)	289 (12,000), 313 (18,000), 451 (48,000)	514	2700 (0.337)	2.583
**9b**	1.76	267 (23,000), 278 (14,000), 304 (8000), 450 (43,000)	513	2700 (0.339)	2.588
**9c**	>2.00	275 (13,000), 301 (4000), 434 (41,000)	502	3100 (0.387)	2.666
**9d**	1.61 (irrev.)	259 (25,000), 307 (5000), 421 (37,000)	481	3000 (0.368)	2.764
**9e**	1.71	257 (43,000), 387 (54,000)	525	6800 (0.843)	2.785
**9f**	1.13 (irr)	305 (27,000), 352 (15,000), 499 (42,000)	632	4200 (0.523)	2.225
**9g**	0.93, 1.61	256 (40,000), 304 (13,000), 379 (17,000), 490 (28,000)	604	3900 (0.478)	2.294
**10a**	1.45	259 (21,000), 337 (9000), 462 (31,000)	– ^5^	– ^5^	–
**10b**	1.46	270 (13,000), 324 (21,000), 550 (26,000)	650	2800 (0.347)	2.083
**10c**	1.38, 1.57	289 (22,000), 314 (22,000), 349 (25,000), 500 (50,000)	594	3200 (0.393)	2.286
**10d**	0.87, 1.38	305 (27,000), 351 (23,000), 550 (40,000)	– ^5^	– ^5^	–
**10e**	0.87, 1.43	256 (28,000), 310 (21,000), 401 (6000), 556 (41,000)	– ^5^	– ^5^	–
**10f**	0.93, 1.46	262 (21,000), 313 (19,000), 347 (17,000), 419 (11,000), 523 (33,000)	– ^5^	– ^5^	–
**10g**	0.86, 1.40	274 (37,000), 310 (26,000), 345 (20,000), 420 (12,000), 527 (37000)	– ^5^	– ^5^	–
**10h**	0.86, 1.51	258 (51,000), 319 (20,000), 417 (11,000), 533 (30,000)	– ^5^	– ^5^	–
**11a**	1.44	253 (41,000), 262 (40,000), 298 (41,000), 354 (15,000), 453 (44,000)	526	3100 (0.380)	2.549
**11b**	1.44	253 (34,000), 262 (35,000), 286 (35,000), 356 (13,000), 453 (39,000)	530	3200 (0.398)	2.541
**11c**	1.46	253 (55,000), 285 (46,000), 347 (21,000), 464 (52,000)	561	3700 (0.462)	2.443
**11d**	1.45	256 (44,000), 280 (40,000), 342 (22,000), 428 (29,000)	526	4400 (0.540)	2.629
**11e**	1.44	260 (37,000), 284 (40,000), 344 (19,000), 431 (31,000)	558	5200 (0.655)	2.552
**11f**	1.44	254 (40,000), 290 (32,000), 366 (13,000), 530 (45,000)	628	2900 (0.365)	2.159
**11g**	1.34	281 (34,000), 303 (21,000), 352 (19,000), 467 (43,000)	583	4300 (0.529)	2.393
**11h**	0.82, 1.38	252 (42,000), 307 (39,000), 329 (40,000), 425 (23,000)	– ^5^	– ^5^	–
**11i**	0.81, 1.39	286 (69,000), 341 (28,000), 424 (26,000)	– ^5^	– ^5^	–
**12a**	1.05, 1.68	271 (34,000), 302 (27,000), 361 (20,000), 482 (24,000)	655	5500 (0.680)	2.235
**12b**	1.05, 1.68	271 (30,000), 303 (24,000), 358 (21,000), 483 (25,000)	658	5500 (0.683)	2.228
**12c**	1.05, 1.69	268 (46,000), 309 (23,000), 338 (25,000), 512 (27,000)	685	4900 (0.612)	2.118
**12e**	1.05, 1.70	260 (49,000), 311 (35,000), 487 (25,000)	638	4900 (0.603)	2.247
**12f**	1.03, 1.69	271 (29,000), 319 (27,000), 472 (21,000)	580	4000 (0.490)	2.384
**12g**	1.05, 1.69	267 (34,000), 320 (13,000), 392 (20,000), 410 (19,000), 580 (34,000)	– ^5^	– ^5^	–
**12h**	– ^6^	280 (27,000), 307 (28,000), 358 (26,000), 473 (28,000)	641	5500 (0.688)	2.280
**12i**	1.13	268 (35,000), 331 (29,000), 362 (21,000), 504 (28,000)	668	4900 (0.605)	2.160
**12j**	1.17	277 (24,000), 304 (29,000), 358 (23,000), 472 (29,000)	636	5500 (0.678)	2.290
**12k**	1.01	267 (32,000), 297 (25,000), 363 (21,000), 484 (25,000)	660	5500 (0.684)	2.222
**12l**	1.03, 1.69	270 (29,000), 332 (25,000), 474 (21,000)	580	4000 (0.478)	2.379
**12m**	1.00, 1.61	261 (45,000), 312 (27,000), 489 (21,000)	614	4200 (0.517)	2.280
**12n**	1.03, 1.57	303 (29,000), 360 (23,000), 483 (23,000)	640	5100 (0.630)	2.254
**12o**	0.79, 1.01	305 (23,000), 371 (49,000), 492 (28,000)	– ^5^	– ^5^	–
**12p**	1.06, 1.33	293 (18,000), 396 (42,000)	651	5400 (0.674)	2.243
**12r**	0.98, 1.20	261 (30,000), 302 (20,000), 384 (24,000), 438 (26,000)	– ^5^	– ^5^	–
**12s**	0.86, 1.12	293 (14,000), 398 (44,000)	629	4600 (0.565)	2.255
**12t**	0.86, 1.80	294 (15,000), 364 (23,000)	634	4500 (0.560)	2.237
**12u**	0.86, 1.11	250 (25,000), 367 (35,000)	– ^5^	– ^5^	–
**12v**	0.85, 1.11, 1.67, 1.79	296 (15,000), 402 (38,000)	– ^5^	– ^5^	–
**12w**	0.85, 1.12	258 (22,000), 265 (21,000), 403 (38,000)	658	5400 (0.667)	2.220
**12x**	0.86, 1.14, 1.47	261 (30,000), 302 (20,000), 384 (24,000), 438 (26,000)	641	4700 (0.586)	2.229
**12y**	0.85, 1.17	259 (35,000), 334 (20,000), 442 (15,000)	657	4900 (0.613)	2.195
**12z**	0.85, 1.11, 1.66, 1.81	289 (12,000), 313 (18,000), 451 (48,000)	– ^5^	– ^5^	–
**14**	1.10	267 (23,000), 278 (14,000), 304 (8000), 450 (43,000)	674	5000 (0.626)	2.154
**15**	1.09	275 (13,000), 301 (4000), 434 (41,000)	642	5700 (0.696)	2.281
**16**	1.08, 1.76	259 (25,000), 307 (5000), 421 (37,000)	565	3900 (0.490)	2.441
**17**	1.09	257 (43,000), 387 (54,000)	640	5400 (0.668)	2.273

^1^ Recorded in CH_2_Cl_2_, *T* = 293 K, *v* = 100 mV·s^−1^, electrolyte: [Bu_4_N][PF_6_], Pt-working electrode, Pt-counter electrode, Ag/AgCl reference electrode; *E*_0_ = (*E*_pa_ + *E*_pc_)/2 referenced to [Fc]/[Fc]^+^ = 0.45 V [34]; *E*_1/2_ = *E*_0_ + 0.20 V (referenced against NHE). ^2^ λ_exc_ at longest wavelength maximum. ^3^ Δ*ṽ* = 1/*λ*_max,abs_ − 1/*λ*_max,em_ [cm^–1^] (Δ*ṽ* = 1241·(1/*λ*_max,abs_ − 1/*λ*_max,em_) [eV]). ^4^ *E*_0-0_: the energy of the hypothetical 0-0-transition determined from the intersection of the absorption and emission band, i.e., *E*_0-0_ = [1241·(1/*λ*_max,abs_ + 1/*λ*_max,em_)]/2 [eV] (wavelength inserted as nanometer value). ^5^ No detectable emission. ^6^ The potential was not determined.

**Table 2:** Selected electronic properties of merocyanine series **9**, **11**, **12**, and **14**–**17** employed in the correlation analyses.

Donor	** 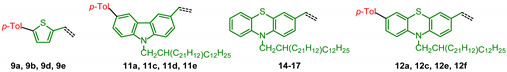 **					
Acceptor		E_1/2_ [V]	λ_max,abs_ [eV]	λ_max,em_ [eV]	Δ*ṽ* [eV]	E_0-0_ [eV]
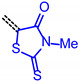	**9a**	1.66	2.752	2.414	0.337	2.583
	**11a**	1.44	2.740	2.359	0.380	2.549
	**12a**	1.05	2.575	1.895	0.680	2.235
	**15**	1.09	2.629	1.933	0.696	2.281
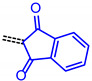	**9b**	1.76	2.758	2.419	0.339	2.588
	**11c**	1.46	2.675	2.212	0.462	2.443
	**12c**	1.05	2.424	1.812	0.612	2.118
	**14**	1.10	2.467	1.841	0.626	2.154
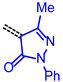	**9d**	1.61	2.948	2.580	0.368	2.764
	**11d**	1.45	2.899	2.359	0.540	2.629
	**12e**	1.05	2.548	1.945	0.603	2.246
	**17**	1.09	2.607	1.939	0.668	2.273
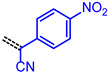	**9e**	1.71	3.207	2.364	0.843	2.785
	**11e**	1.44	2.879	2.224	0.655	2.552
	**12f**	1.03	2.629	2.140	0.490	2.384
	**16**	1.08	2.686	2.196	0.489	2.441

**Table 3:** Linear correlation analyses of the consanguineous acceptor series.

Acceptor	Linear Correlation Equations *E*_0-0_ vs. *E*_1/2_	Correlation Coefficient
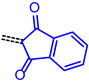	*E*_0-0_ = 1.41368 + 0.67953·*E*_1/2_ [eV]	r^2^ = 0.98716
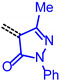	*E*_0-0_ = 1.25336 + 0.94225·*E*_1/2_ [eV]	r^2^ = 0.99896
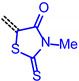	*E*_0-0_ = 1.62924 + 0.59758·*E*_1/2_ [eV]	r^2^ = 0.94309
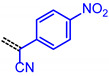	*E*_0-0_ = 1.83274 + 0.53835·*E*_1/2_ [eV]	r^2^ = 0.94765

## Data Availability

All data of this work are included in the manuscript and the Supplementary Materials. *UV*/*VIS* and fluorescence spectra as well as cyclic voltammograms can be provided from the authors upon request.

## Supplementary Materials

The following are available online, Supporting Information: Synthetic and analytic details, and ^1^H and ^13^C NMR spectra of compounds **8**–**12**, and **14**–**17**, correlation analysis of the different series.

## Sample Availability

Samples of the compounds are not available from the authors.
