# Supplementary material for: Three-Component Suzuki–Knoevenagel Synthesis of Merocyanine Libraries and Correlation Analyses of Their Oxidation Potentials and Optical Band Gaps
Source: Molecules. 2021 Aug 25;26(17):5149. doi: 10.3390/molecules26175149 (PMC8433686; doi:10.3390/molecules26175149)
Supplement: Supplementary file 1 [file molecules-26-05149-s001.zip › molecules-1323319-supplementary.pdf]

Supporting Information

Consecutive Three-Component Suzuki-Knoevenagel Synthesis of Merocyanine Libraries and Correlation Analyses of their Oxidation Potentials and Optical Band Gaps

Tim Meyer, Roxanne Krug, and Thomas J. J. Müller

Institut für Organische Chemie und Makromolekulare Chemie, Heinrich-Heine-Universität Düsseldorf, Universitätsstrasse 1, D-40225 Düsseldorf, Germany

E-Mail: ThomasJJ.Mueller@hhu.de

Table of Contents

1. General Considerations **8**

2. Syntheses **9**

2.1. General Procedure (GP) for the Coupling-Condensation One-pot Synthesis of Chromophores **8-12** **9**

2.1.1. Consecutive Three-component Suzuki-Knoevenagel Synthesis of *p*-Phenylene bridged Systems **8** **9**

2.1.1.1. 5-(Biphenyl-4-ylmethylene)-3-methyl-2-thioxothiazolidin-4-one (**8a**) **10**

2.1.1.2. 2-(Biphenyl-4-ylmethylene)-1*H*-inden-1,3[2*H*]-dione (**8b**) **10**

2.1.1.3. 5-{[4'-Methyl-(1,1'-biphenyl)-4-yl]methylene}-2-thioxothiazolidin-4-one (**8c**) **11**

2.1.1.4. 3-(4'-Methyl-[1,1'-biphenyl]-4-yl)-2-(4-nitrophenyl)acrylnitrile (**8d**) **11**

2.1.1.5. 3-Methyl-4-{[4'-methyl-(1,1'-biphenyl)-4-yl]methylene}-1-phenyl-1*H*-pyrazol-5[4*H*]-one (**8e**) **12**

2.1.1.6. 3-Methyl-5-(4-(thiophen-3-yl)benzylidene)-2-thioxothiazolidin-4-one (**8f**) **12**

2.1.1.7. 2-[4-(1-Methyl-1*H*-pyrazol-4-yl)benzylidene]-1*H*-inden-1,3[2*H*]-dione (**8g**) **13**

2.1.1.8. 5-[4-(10-Hexyl-10*H*-phenothiazin-3-yl)benzylidene]-3-methyl-2-thioxothiazolidin-4-one (**8h**) **14**

2.1.1.9. 4-[4-(10-Hexyl-10*H*-phenothiazin-3-yl)benzylidene]-3-methyl-1-phenyl-1*H*-pyrazol-5[4*H*]-one (**8i**) **14**

2.1.2. Consecutive Three-component Suzuki-Knoevenagel Synthesis of Thienylene-bridged Systems **9** **15**

2.1.2.1. 3-Methyl-2-thioxo-5-{[5-(*p*-tolyl)thiophen-2-yl]methylene}thiazolidin-4-one (**9a**) **16**

2.1.2.2. 2-{[5-(*p*-Tolyl)thiophen-2-yl]methylene}-1*H*-inden-1,3[2*H*]-dione (**9b**) **16**

2.1.2.3. 1,3-Dipropyl-5-{[5-(*p*-tolyl)thiophen-2-yl]methylene}pyrimidin-2,4,6[1*H*,3*H*,5*H*]-trione (**9c**) **17**

2.1.2.4. 3-Methyl-1-phenyl-4-{[5-(*p*-tolyl)thiophen-2-yl]methylene}-1H-pyrazol-5[4*H*]-one (**9d**) **17**

2.1.2.5. 2-(4-Nitrophenyl)-3-[5-(*p*-tolyl)thiophen-2-yl]acrylonitrile (**9e**) **18**

2.1.2.6. 5-{[5-(4-{Diphenylamino}phenyl)thiophen-2-yl]methylene}-3-methyl-2-thioxothiazolidin-4-one (**9f**) **18**

2.1.2.7. 4-{[5-(10-Hexyl-10*H*-phenothiazin-3-yl)thiophen-2-yl]methylene}-3-methyl-1-phenyl-1*H*-pyrazol-5[4*H*]-one (**9g**) **19**

2.1.3. Consecutive Three-component Suzuki-Knoevenagel Synthesis of 4-^{n}Ocytyloxy-substituted Thienylene-bridged Systems **9** **20**

2.1.3.1. (*Z*)-5-Methyl-4-((4-(octyloxy)-5-(*p*-tolyl)thiophen-2-yl)methylene)-2-phenyl-2,4-dihydro-3*H*-pyrazol-3-one (**10a**) **20**

2.1.3.2. (*E*)-2-(3-Cyano-5,5-dimethyl-4-(2-(4-(octyloxy)-5-(*p*-tolyl)thiophen-2-yl)vinyl)furan-2(5*H*)-ylidene)malononitrile (**10b**) **21**
2.1.3.3. (*Z*)-5-((4-(Octyloxy)-5-(4-(1,2,2-triphenylvinyl)phenyl)thiophen-2-yl)methylene)-2-thioxothiazolidin-4-one (**10c**) **22**
2.1.3.4. 5-{[5-(4-{Bis[4-methoxyphenyl]amino}phenyl)-3-(octyloxy)thiophen-2-yl]methylene}-2-thioxothiazolidin-4-one (**10d**) **23**
2.1.3.5. (*Z*)-4-((5-(4-(Bis(4-methoxyphenyl)amino)phenyl)-4-(octyloxy)thiophen-2-yl)methylene)-5-methyl-2-phenyl-2,4-dihydro-3*H*-pyrazol-3-one (**10e**) **23**
2.1.3.6. (*Z*)-5-((5-(10-(2-Decyltetradecyl)-10*H*-phenothiazin-3-yl)-4-(octyloxy)thiophen-2-yl)methylene)-2-thioxothiazolidin-4-one (**10f**) **24**
2.1.3.7. (*Z*)-5-((5-(10-Hexyl-7-(*p*-tolyl)-10*H*-phenothiazin-3-yl)-4-(octyloxy)thiophen-2-yl)methylene)-2-thioxothiazolidin-4-one (**10g**) **25**
2.1.3.8. (*Z*)-4-((5-(10-Hexyl-7-(*p*-tolyl)-10*H*-phenothiazin-3-yl)-4-(octyloxy)thiophen-2-yl)methylene)-5-methyl-2-phenyl-2,4-dihydro-3*H*-pyrazol-3-one (**10h**) **26**
2.1.4. Consecutive Three-component Suzuki-Knoevenagel Synthesis of Carbazole-bridged Systems **11** **27**
2.1.4.1. 5-{[9-(2-Decyltetradecyl)-6-(*p*-tolyl)-9*H*-carbazol-3-yl]methylene}-3-methyl-2-thioxothiazolidin-4-one (11a) **27**
2.1.4.2. 5-{[9-(2-Decyltetradecyl)-6-(*p*-tolyl)-9H-carbazol-3-yl]methylene}-2-thioxothiazolidin-4-one (**11b**) **28**
2.1.4.3. 2-{[9-(2-Decyltetradecyl)-6-(*p*-tolyl)-9*H*-carbazol-3-yl]methylene}-1*H*-inden-1,3[2*H*]-dione (**11c**) **29**
2.1.4.4. 4-{[9-(2-Decyltetradecyl)-6-(*p*-tolyl)-9*H*-carbazol-3-yl]methylene}-3-methyl-1-phenyl-1*H*-pyrazol-5[4*H*]-one (**11d**) **30**
2.1.4.5. 3-[9-(2-Decyltetradecyl)-6-(*p*-tolyl)-9*H*-carbazol-3-yl]-2-(4-nitrophenyl)acrylonitrile (**11e**) **31**
2.1.4.6. (*E*)-2-{3-Cyano-4-[2-(9-{2-decyltetradecyl}-6-{*p*-tolyl}-9*H*-carbazol-3-yl)vinyl]-5,5-dimethylfuran-2[5*H*]-yliden}malonitrile (**11f**) **32**
2.1.4.7. 2-{[9-(2-Decyltetradecyl)-6-(1-methyl-1H-pyrazol-4-yl)-9*H*-carbazol-3-yl]methylene}-1*H*-inden-1,3[2*H*]-dione (**11g**) **33**
2.1.4.8. 4-{[6-(4-{Bis[4-methoxyphenyl]amino}phenyl)-9-(2-decyltetradecyl)-9*H*-carbazol-3-yl]methylene}-3-methyl-1-phenyl-1*H*-pyrazol-5[4*H*]-one (**11h**) **34**
2.1.4.9. 4-{[9-(2-Decyltetradecyl)-6-(10-hexyl-7-{*p*-tolyl}-10*H*-phenothiazin-3-yl)-9*H*-carbazol-3-yl]methylene}-3-methyl-1-phenyl-1H-pyrazol-5[4*H*]-one (**11i**) **35**
2.1.5. Consecutive Three-component Suzuki-Knoevenagel Synthesis of Phenothiazine-bridged Systems **12** **36**
2.1.5.1. (*Z*)-5-{[10-(2-Decyltetradecyl)-7-(*p*-tolyl)-10*H*-phenothiazin-3-yl]methylene}-3-methyl-2-thioxothiazolidin-4-one (**12a**) **37**
2.1.5.2. (*Z*)-5-{[10-(2-Decyltetradecyl)-7-(*p*-tolyl)-10*H*-phenothiazin-3-yl]methylene}-2-thioxothiazolidin-4-one (**12b**) **38**
2.1.5.3. 2-{[10-(2-Decyltetradecyl)-7-(*p*-tolyl)-10*H*-phenothiazin-3-yl]methylene}-1*H*-inden-1,3[2*H*]-dione (**12c**) **39**

2.1.5.4. 11-[10-(2-Decyltetradecyl)-7-(*p*-tolyl)-10*H*-phenothiazin-3-yl]diindeno[1,2-*b*:2',1'-*e*]pyridin-10,12-dione (**12d**) **40**
2.1.5.5. (*Z*)-4-[(10-(2-Decyltetradecyl)-7-(*p*-tolyl)-10*H*-phenothiazin-3-yl)methylene]-3-methyl-1-phenyl-1*H*-pyrazol-5[4*H*]-one (**12e**) **41**
2.1.5.6. (*Z*)-3-[10-(2-Decyltetradecyl)-7-(*p*-tolyl)-10H-phenothiazin-3-yl]-2-(4-nitrophenyl)acrylonitrile (**12f**) **41**
2.1.5.7. (*E*)-2-{3-Cyano-4-[2-(10-{2-decyltetradecyl}-7-{*p*-tolyl}-10*H*-phenothiazin-3-yl)vinyl]-5,5-dimethylfuran-2[5*H*]-yliden}malonitrile (**12g**) **42**
2.1.5.8. (*Z*)-5-{[10-(2-Decyltetradecyl)-7-(*p*-tolyl)-10*H*-phenothiazin-3-yl]methylene}-3-methyl-2-thioxothiazolidin-4-one (**12h**) **43**
2.1.5.9. 4-{10-(2-Decyltetradecyl)-7-[(1,3-dioxo-1H-inden-2[3*H*]-yliden)methyl]-10*H*-phenothiazin-3-yl}benzonitrile (**12i**) **44**
2.1.5.10. (Z)-5-{[10-(2-Decyltetradecyl)-7-(pyridin-4-yl)-10*H*-phenothiazin-3-yl]methylene}-3-methyl-2-thioxothiazolidin-4-one (**12j**) **45**
2.1.5.11. (*Z*)-5-{[10-(2-Decyltetradecyl)-7-(1-methyl-1*H*-pyrazol-4-yl)-10*H*-phenothiazin-3-yl]methylene}-3-methyl-2-thioxothiazolidin-4-one (**12k**) **45**
2.1.5.12. (*Z*)-3-[10-(2-Decyltetradecyl)-7-(1-methyl-1*H*-pyrazol-4-yl)-10*H*-phenothiazin-3-yl]-2-(4-nitrophenyl)acrylonitrile (**12l**) **46**
2.1.5.13. (*Z*)-4-{[10-(2-Decyltetradecyl)-7-(1-methyl-1H-pyrazol-4-yl)-10*H*-phenothiazin-3-yl]methylene}-3-methyl-1-phenyl-1H-pyrazol-5[4*H*]-one (**12m**) **47**
2.1.5.14. (*Z*)-5-{[10-(2-Decyltetradecyl)-7-(5-methylthiophen-2-yl)-10*H*-phenothiazin-3-yl]methylene}-3-methyl-2-thioxothiazolidin-4-one (**12n**) **48**
2.1.5.15. (*Z*)-5-{[10-(2-Decyltetradecyl)-7-(5-{4-[diethylamino]phenyl}thiophen-2-yl)-10*H*-phenothiazin-3-yl]methylene}-3-methyl-2-thioxothiazolidin-4-one (**12o**) **49**
2.1.5.16. (*Z*)-5-{[7-(5-{9*H*-Carbazol-9-yl}thiophen-2-yl)-10-(2-decyltetradecyl)-10*H*-phenothiazin-3-yl]methylene}-3-methyl-2-thioxothiazolidin-4-one (**12p**) **50**
2.1.5.17. (*Z*)-5-{[10-(2-Decyltetradecyl)-7-(4-{diphenylamino}phenyl)-10*H*-phenothiazin-3-yl]methylene}-3-methyl-2-thioxothiazolidin-4-one (**12q**) **50**
2.1.5.18. (*Z*)-3-{10-(2-Decyltetradecyl)-7-[4-(diphenylamino)phenyl]-10*H*-phenothiazin-3-yl}-2-(4-nitrophenyl)acrylonitrile (**12r**) **51**
2.1.5.19. (*Z*)-5-{[7-(4-{Bis[4-methoxyphenyl]amino}phenyl)-10-(2-decyltetradecyl)-10*H*-phenothiazin-3-yl]methylene}-3-methyl-2-thioxothiazolidin-4-one (**12s**) **52**
2.1.5.20. (*Z*)-5-{[7-(4-{Bis[4-methoxyphenyl]amino}phenyl)-10-(2-decyltetradecyl)-10*H*-phenothiazin-3-yl]methylene}-2-thioxothiazolidin-4-one (**12t**) **53**
2.1.5.21. (*Z*)-3-{7-[4-(Bis{4-methoxyphenyl}amino)phenyl]-10-(2-decyltetradecyl)-10*H*-phenothiazin-3-yl}-2-(4-nitrophenyl)acrylonitrile (**12u**) **54**
2.1.5.22. (*Z*)-4-{[7-(4-(Bis{4-methoxyphenyl}amino)phenyl]-10-(2-decyltetradecyl)-10*H*-phenothiazin-3-yl]methylene}-3-methyl-1-phenyl-1H-pyrazol-5[4*H*]-one (**12v**) **55**
2.1.5.23. (*Z*)-5-{[10-(2-Decyltetradecyl)-10'-hexyl-7'-(*p*-tolyl)-10*H*,10'*H*-(3,3'-biphenothiazin)-7-yl]methylene}-3-methyl-2-thioxothiazolidin-4-one (**12w**) **56**

2.1.5.24. (*Z*)-5-{[10-(2-Decyltetradecyl)-10'-hexyl-7'-(*p*-tolyl)-10*H*,10'*H*-(3,3'-biphenothiazin)-7-yl]methylene}-2-thioxothiazolidin-4-one (**12x**) **57**
2.1.5.25. (*Z*)-3-[10-(2-Decyltetradecyl)-10'-hexyl-7'-(*p*-tolyl)-10*H*,10'*H*-(3,3'-biphenothiazin)-7-yl]-2-(4-nitrophenyl)acrylonitrile (**12y**) **58**
2.1.5.26. (*Z*)-4-{[10-(2-Decyltetradecyl)-10'-hexyl-7'-(*p*-tolyl)-10H,10'H-(3,3'-biphenothiazin)-7-yl]methylene}-3-methyl-1-phenyl-1H-pyrazol-5[4H]-one (**12z**) **59**
2.2. General Procedure (GP) for the Knoevenagel Condensation Synthesis of Reference Merocyanines **14-17** **60**
2.2.1. 2-{[10-(2-Decyltetradecyl)-10*H*-phenothiazin-3-yl]methylene}-1*H*-inden-1,3[2*H*]-dione (**14**) **60**
2.2.2. (*Z*)-5-{[10-(2-Decyltetradecyl)-10H-phenothiazin-3-yl]methylene}-3-methyl-2-thioxothiazolidin-4-one (**15**) **61**
2.2.3. (*Z*)-3-(10-(2-Decyltetradecyl)-10*H*-phenothiazin-3-yl)-2-(4-nitrophenyl)acrylonitrile (**16**) **62**
2.2.4. (*Z*)-4-{[10-(2-Decyltetradecyl)-10*H*-phenothiazin-3-yl]methylene}-5-methyl-2-phenyl-2,4-dihydro-3*H*-pyrazol-3-one (**17**) **63**
3. ^{1}H and ^{13}C NMR Spectra **64**
3.1. 5-(Biphenyl-4-ylmethylene)-3-methyl-2-thioxothiazolidin-4-one (**8a**) **64**
3.2. 2-(Biphenyl-4-ylmethylene)-1*H*-inden-1,3[2*H*]-dione (**8b**) **66**
3.3. 5-{[4'-Methyl-(1,1'-biphenyl)-4-yl]methylene}-2-thioxothiazolidin-4-one (**8c**) **68**
3.4. 3-(4'-Methyl-[1,1'-biphenyl]-4-yl)-2-(4-nitrophenyl)acrylnitrile (**8d**) **70**
3.5. 3-Methyl-4-{[4'-methyl-(1,1'-biphenyl)-4-yl]methylene}-1-phenyl-1*H*-pyrazol-5[4*H*]-one (**8e**) **72**
3.6. 3-Methyl-5-(4-(thiophen-3-yl)benzylidene)-2-thioxothiazolidin-4-one (**8f**) **74**
3.7. 2-[4-(1-Methyl-1*H*-pyrazol-4-yl)benzylidene]-1*H*-inden-1,3[2*H*]-dione (**8g**) **76**
3.8. 5-[4-(10-Hexyl-10*H*-phenothiazin-3-yl)benzylidene]-3-methyl-2-thioxothiazolidin-4-one (**8h**) **78**
3.9. 4-[4-(10-Hexyl-10*H*-phenothiazin-3-yl)benzylidene]-3-methyl-1-phenyl-1*H*-pyrazol-5[4*H*]-one (**8i**) **80**
3.10. 3-Methyl-2-thioxo-5-{[5-(*p*-tolyl)thiophen-2-yl]methylene}thiazolidin-4-one (**9a**) **82**
3.11. 2-{[5-(*p*-Tolyl)thiophen-2-yl]methylene}-1*H*-inden-1,3[2*H*]-dione (**9b**) **84**
3.12. 1,3-Dipropyl-5-{[5-(*p*-tolyl)thiophen-2-yl]methylene}pyrimidin-2,4,6[1*H*,3*H*,5*H*]-trione (**9c**) **86**
3.13. 3-Methyl-1-phenyl-4-{[5-(*p*-tolyl)thiophen-2-yl]methylene}-1H-pyrazol-5[4*H*]-one (**9d**) **88**
3.14. 2-(4-Nitrophenyl)-3-[5-(*p*-tolyl)thiophen-2-yl]acrylonitrile (**9e**) **90**
3.15. 5-{[5-(4-{Diphenylamino}phenyl)thiophen-2-yl]methylene}-3-methyl-2-thioxothiazolidin-4-one (**9f**) **92**
3.16. 4-{[5-(10-Hexyl-10*H*-phenothiazin-3-yl)thiophen-2-yl]methylene}-3-methyl-1-phenyl-1*H*-pyrazol-5[4*H*]-one (**9g**) **94**
3.17. (*Z*)-5-Methyl-4-((4-(octyloxy)-5-(*p*-tolyl)thiophen-2-yl)methylene)-2-phenyl-2,4-dihydro-3*H*-pyrazol-3-one (**10a**) **96**
3.18. (*E*)-2-(3-Cyano-5,5-dimethyl-4-(2-(4-(octyloxy)-5-(*p*-tolyl)thiophen-2-yl)vinyl)furan-2(5*H*)-ylidene)malononitrile (**10b**) **98**
3.19. (*Z*)-5-((4-(Octyloxy)-5-(4-(1,2,2-triphenylvinyl)phenyl)thiophen-2-yl)methylene)-2-thioxothiazolidin-4-one (**10c**) **100**

3.20. 5-{[5-(4-{Bis[4-methoxyphenyl]amino}phenyl)-3-(octyloxy)thiophen-2-yl]methylene}-2-thioxothiazolidin-4-one (**10d**) **102**

3.21. (*Z*)-4-((5-(4-(Bis(4-methoxyphenyl)amino)phenyl)-4-(octyloxy)thiophen-2-yl)methylene)-5-methyl-2-phenyl-2,4-dihydro-3*H*-pyrazol-3-one (**10e**) **104**

3.22. (*Z*)-5-((5-(10-(2-Decyltetradecyl)-10*H*-phenothiazin-3-yl)-4-(octyloxy)thiophen-2-yl)methylene)-2-thioxothiazolidin-4-one (**10f**) **106**

3.23. (*Z*)-5-((5-(10-Hexyl-7-(*p*-tolyl)-10*H*-phenothiazin-3-yl)-4-(octyloxy)thiophen-2-yl)methylene)-2-thioxothiazolidin-4-one (**10g**) **108**

3.24. (*Z*)-4-((5-(10-Hexyl-7-(*p*-tolyl)-10*H*-phenothiazin-3-yl)-4-(octyloxy)thiophen-2-yl)methylene)-5-methyl-2-phenyl-2,4-dihydro-3*H*-pyrazol-3-one (**10h**) **110**

3.25. 5-{[9-(2-Decyltetradecyl)-6-(*p*-tolyl)-9*H*-carbazol-3-yl]methylene}-3-methyl-2-thioxothiazolidin-4-one (**11a**) **112**

3.26. 5-{[9-(2-Decyltetradecyl)-6-(*p*-tolyl)-9*H*-carbazol-3-yl]methylene}-2-thioxothiazolidin-4-one (**11b**) **114**

3.27. 2-{[9-(2-Decyltetradecyl)-6-(*p*-tolyl)-9*H*-carbazol-3-yl]methylene}-1*H*-inden-1,3[2*H*]-dione (**11c**) **116**

3.28. 4-{[9-(2-Decyltetradecyl)-6-(*p*-tolyl)-9*H*-carbazol-3-yl]methylene}-3-methyl-1-phenyl-1*H*-pyrazol-5[4*H*]-one (**11d**) **118**

3.29. 3-[9-(2-Decyltetradecyl)-6-(*p*-tolyl)-9*H*-carbazol-3-yl]-2-(4-nitrophenyl)acrylonitrile (**11e**) **120**

3.30. (*E*)-2-{3-Cyano-4-[2-(9-{2-decyltetradecyl}-6-{*p*-tolyl}-9*H*-carbazol-3-yl)vinyl]-5,5-dimethylfuran-2[5*H*]-yliden}malonitrile (**11f**) **122**

3.31. 2-{[9-(2-Decyltetradecyl)-6-(1-methyl-1H-pyrazol-4-yl)-9*H*-carbazol-3-yl]methylene}-1*H*-inden-1,3[2*H*]-dione (**11g**) **124**

3.32. 4-{[6-(4-{Bis[4-methoxyphenyl]amino}phenyl)-9-(2-decyltetradecyl)-9*H*-carbazol-3-yl]methylene}-3-methyl-1-phenyl-1*H*-pyrazol-5[4*H*]-one (**11h**) **126**

3.33. 4-{[9-(2-Decyltetradecyl)-6-(10-hexyl-7-{*p*-tolyl}-10*H*-phenothiazin-3-yl)-9*H*-carbazol-3-yl]methylene}-3-methyl-1-phenyl-1H-pyrazol-5[4*H*]-one (**11i**) **128**

3.34. (*Z*)-5-{[10-(2-Decyltetradecyl)-7-(*p*-tolyl)-10*H*-phenothiazin-3-yl]methylene}-3-methyl-2-thioxothiazolidin-4-one (12a) **130**

3.35. (*Z*)-5-{[10-(2-Decyltetradecyl)-7-(*p*-tolyl)-10*H*-phenothiazin-3-yl]methylene}-2-thioxothiazolidin-4-one (**12b**) **132**

3.36. 2-{[10-(2-Decyltetradecyl)-7-(*p*-tolyl)-10*H*-phenothiazin-3-yl]methylene}-1*H*-inden-1,3[2*H*]-dione (**12c**) **134**

3.37. 11-[10-(2-Decyltetradecyl)-7-(*p*-tolyl)-10*H*-phenothiazin-3-yl]diindeno[1,2-*b*:2',1'-*e*]pyridin-10,12-dione (**12d**) **136**

3.38. (*Z*)-4-[(10-(2-Decyltetradecyl)-7-(*p*-tolyl)-10*H*-phenothiazin-3-yl)methylene]-3-methyl-1-phenyl-1*H*-pyrazol-5[4*H*]-one (**12e**) **138**

3.39. (*Z*)-3-[10-(2-Decyltetradecyl)-7-(*p*-tolyl)-10H-phenothiazin-3-yl]-2-(4-nitrophenyl)acrylonitrile (**12f**) **140**

3.40. (*E*)-2-{3-Cyano-4-[2-(10-{2-decyltetradecyl}-7-{*p*-tolyl}-10*H*-phenothiazin-3-yl)vinyl]-5,5-dimethylfuran-2[5*H*]-yliden}malonitrile (**12g**) **142**

3.41. (*Z*)-5-{[10-(2-Decyltetradecyl)-7-(*p*-tolyl)-10*H*-phenothiazin-3-yl]methylene}-3-methyl-2-thioxothiazolidin-4-one (**12h**) **144**

3.42. 4-{10-(2-Decyltetradecyl)-7-[(1,3-dioxo-1H-inden-2[3*H*]-yliden)methyl]-10*H*-phenothiazin-3-yl}benzonitrile (**12i**) **146**

3.43. (Z)-5-{[10-(2-Decyltetradecyl)-7-(pyridin-4-yl)-10*H*-phenothiazin-3-yl]methylene}-3-methyl-2-thioxothiazolidin-4-one (**12j**) **148**

3.44. (*Z*)-5-{[10-(2-Decyltetradecyl)-7-(1-methyl-1*H*-pyrazol-4-yl)-10*H*-phenothiazin-3-yl]methylene}-3-methyl-2-thioxothiazolidin-4-one (**12k**) **150**

3.45. (*Z*)-3-[10-(2-Decyltetradecyl)-7-(1-methyl-1*H*-pyrazol-4-yl)-10*H*-phenothiazin-3-yl]-2-(4-nitrophenyl)acrylonitrile (**12l**) **152**

3.46. (*Z*)-4-{[10-(2-Decyltetradecyl)-7-(1-methyl-1H-pyrazol-4-yl)-10*H*-phenothiazin-3-yl]methylene}-3-methyl-1-phenyl-1H-pyrazol-5[4*H*]-one (**12m**) **154**

3.47. (*Z*)-5-{[10-(2-Decyltetradecyl)-7-(5-methylthiophen-2-yl)-10*H*-phenothiazin-3-yl]methylene}-3-methyl-2-thioxothiazolidin-4-one (**12n**) **156**

3.48. (*Z*)-5-{[10-(2-Decyltetradecyl)-7-(5-{4-[diethylamino]phenyl}thiophen-2-yl)-10*H*-phenothiazin-3-yl]methylene}-3-methyl-2-thioxothiazolidin-4-one (**12o**) **158**

3.49. (*Z*)-5-{[7-(5-{9*H*-Carbazol-9-yl}thiophen-2-yl)-10-(2-decyltetradecyl)-10*H*-phenothiazin-3-yl]methylene}-3-methyl-2-thioxothiazolidin-4-one (**12p**) **160**

3.50. (*Z*)-5-{[10-(2-Decyltetradecyl)-7-(4-{diphenylamino}phenyl)-10*H*-phenothiazin-3-yl]methylene}-3-methyl-2-thioxothiazolidin-4-one (**12q**) **162**

3.51. (*Z*)-3-{10-(2-Decyltetradecyl)-7-[4-(diphenylamino)phenyl]-10*H*-phenothiazin-3-yl}-2-(4-nitrophenyl)acrylonitrile (**12r**) **164**

3.52. (*Z*)-5-{[7-(4-{Bis[4-methoxyphenyl]amino}phenyl)-10-(2-decyltetradecyl)-10*H*-phenothiazin-3-yl]methylene}-3-methyl-2-thioxothiazolidin-4-one (**12s**) **166**

3.53. (*Z*)-5-{[7-(4-{Bis[4-methoxyphenyl]amino}phenyl)-10-(2-decyltetradecyl)-10*H*-phenothiazin-3-yl]methylene}-2-thioxothiazolidin-4-one (**12t**) **168**

3.54. (*Z*)-3-{7-[4-(Bis{4-methoxyphenyl}amino)phenyl]-10-(2-decyltetradecyl)-10*H*-phenothiazin-3-yl}-2-(4-nitrophenyl)acrylonitrile (**12u**) **170**

3.55. (*Z*)-4-{[7-(4-(Bis{4-methoxyphenyl}amino)phenyl]-10-(2-decyltetradecyl)-10*H*-phenothiazin-3-yl]methylene}-3-methyl-1-phenyl-1H-pyrazol-5[4*H*]-one (**12v**) **172**

3.56. (*Z*)-5-{[10-(2-Decyltetradecyl)-10'-hexyl-7'-(*p*-tolyl)-10*H*,10'*H*-(3,3'-biphenothiazin)-7-yl]methylene}-3-methyl-2-thioxothiazolidin-4-one (**12w**) **174**

3.57. (*Z*)-5-{[10-(2-Decyltetradecyl)-10'-hexyl-7'-(*p*-tolyl)-10*H*,10'*H*-(3,3'-biphenothiazin)-7-yl]methylene}-2-thioxothiazolidin-4-one (**12x**) **176**

3.58. (*Z*)-3-[10-(2-Decyltetradecyl)-10'-hexyl-7'-(*p*-tolyl)-10*H*,10'*H*-(3,3'-biphenothiazin)-7-yl]-2-(4-nitrophenyl)acrylonitrile (**12y**) **178**

3.59. (*Z*)-4-{[10-(2-Decyltetradecyl)-10'-hexyl-7'-(*p*-tolyl)-10H,10'H-(3,3'-biphenothiazin)-7-yl]methylene}-3-methyl-1-phenyl-1H-pyrazol-5[4H]-one (**12z**) **180**

3.60. 2-{[10-(2-Decyltetradecyl)-10*H*-phenothiazin-3-yl]methylene}-1*H*-inden-1,3[2*H*]-dione (**14**) **182**

3.61. (*Z*)-5-{[10-(2-Decyltetradecyl)-10H-phenothiazin-3-yl]methylene}-3-methyl-2-thioxothiazolidin-4-one (**15**) **184**

3.62. (*Z*)-3-(10-(2-Decyltetradecyl)-10*H*-phenothiazin-3-yl)-2-(4-nitrophenyl)acrylonitrile (**16**) **186**

3.63. (*Z*)-4-{[10-(2-Decyltetradecyl)-10*H*-phenothiazin-3-yl]methylene}-5-methyl-2-phenyl-2,4-dihydro-3*H*-pyrazol-3-one (**17**) **188**

4. Correlation Analyses **190**

4.1. Correlations of the Model Chromophores **14-17** **190**

4.2. Correlations of Consanguineous Acceptor Series **193**

4.2.1. Correlations of 3-Methyl-4-oxo-2-thioxothiazolidin-5-ylidene Merocyanines **194**

4.2.2. Correlations of 1,3-Dioxo-1,3-dihydro-2*H*-inden-2-ylidene Merocyanines **196**

4.2.3. Correlations of 3-Methyl-5-oxo-1-phenyl-1,5-dihydro-4*H*-pyrazol-4-ylidene Merocyanines **198**

4.2.4. Correlations of Cyano(4-nitrophenyl)-methylene Merocyanines **200**

4.3. Linear and Planar Correlations with 24 Compounds **202**

5. References **205**

1. General Considerations

Reagents, catalysts, ligands, and solvents were purchased reagent grade and used without further purification. DMSO was dried and distilled from CaH_2 under argon atmosphere. Boronates/boronic acids **1** were prepared according to our previously published protocols from bromo (hetero)arenes by bromine-lithium exchange, borylation with trialkyl borates, and esterification with pinacol,[1] or from thienyl derivatives by lithiation, borylation with trialkyl borates, and esterification with pinacol.[2] Bromo aldehydes were prepared according to our protocol by desymmetrization of dibromo (heteroarenes)[3] or thiophenes.[2] Column chromatography: silica gel 60, mesh 70-230. TLC: silica gel plates 60 F254.

Instrumentation: Chemical shifts δ in the ^1H NMR and ^{13}C NMR spectra are reported relative to deuterated solvents ($CDCl_3$, acetone-d^6, or DMSO-d^6). The assignments of quaternary C, CH, CH_2, and CH_3 signals were made by using DEPT spectra. The absorption spectra of the dyes were recorded in dichloromethane solutions on a diode array UV-vis spectrometer. Cyclic voltammetry experiments were performed under argon in dry and degassed CH_2Cl_2 at room temperature and at scan rates of 100, 250, 500 and 1000 mVs^{-1} using an electrochemical workstation. The electrolyte was Bu_4NPF_6 (0.025 m). The working electrode was a 1 mm platinum disk, the counter electrode was a platinum wire, and the reference electrode was an Ag/AgCl electrode. The potentials E_0 were corrected to the internal standard of Fc/Fc^+ in CH_2Cl_2 ($E_0^{0/+1}$ = 450 mV) and calculated as $E_{1/2}$ referenced to NHE (normal hydrogen electrode) according to $E_{1/2} = E_0 + 0.20$ V. Mass spectroscopic measurements were conducted on a quadrupole (EI) analyzer (TSQ 7000, Finnigan MAT) in the Department of Mass Spectrometry of the Institute of Inorganic and Structural Chemistry, Heinrich-Heine-Universität Düsseldorf. IR spectra were measured using ATR technique (Shimadzu IR Affinity-1). The intensities of the IR bands are abbreviated as w (weak), m (medium), s (strong) and vs (very strong). Elemental analyses were carried out on a Perkin Elmer Series II Analyser 2400 in the microanalytical laboratory of the Pharmazeutisches Institut of the Heinrich-Heine-Universität Düsseldorf. Absorption spectra were recorded in various spectroscopy grade solvents at 293 K using a PerkinElmer UV/VIS/NIR Lambda 19 spectrometer. Emission spectra in solution were recorded at room temperature on a LS55 spectrometer (Perkin Elmer). Melting points and melting/softening intervals were determined on the apparatus Thermovar, Reichert-Jung/Depew using the method according to L. Kofler.[4]

2. Syntheses

2.1. General Procedure (GP) for the Coupling-Condensation One-pot Synthesis of Chromophores 8-12

(Hetero)aryl boronic acid or boronate **6** (1.1-1.2 equivs), bromoaldehyde **1-5** (1.0 equiv), cesium fluoride or cesium carbonate (3.2 equivs), and tetrakis(triphenylphosphane)-palladium(0) (0.02-0.03 equivs) were placed in a Schlenk flask with magnetic stir bar under nitrogen and dry 1,4-dioxane (4 mL/mmol) were added (for experimental details see Tables S1-5). The solution was heated to 100 °C under reflux for 8-16 h. After cooling to room temp acetic acid (2 mL/mmol), the CH-acidic compound **7** (1.1-1.2 equivs), and a catalytic or equimolar amount of ammonium acetate was added to the reaction mixture. This mixture was heated under nitrogen to 95 °C under reflux for 3-8 h. Intensive orange, red or dark violet solutions were formed. After cooling to room temp the reaction mixture was diluted with dichloromethane (30 mL/mmol) and the organic layer was washed with distilled water until the aqueous phase did not smell like acetic acid. The combined aqueous phases were extracted with dichloromethane and the combined organic layers were dried (anhydrous magnesium sulfate) and the solvents were removed in vacuo. The residue was adsorbed on celite® and purified by flash chromatography on silica gel (*n*-hexane/acetone, toluene or ethyl acetate and gradients thereof) to furnish the chromophores **8-12**.

2.1.1. Consecutive Three-component Suzuki-Knoevenagel Synthesis of *p*-Phenylene bridged Systems 8

Table S1. Experimental details of the consecutive three-component Suzuki-Knoevenagel synthesis of *p*-phenylene bridged systems **8**.

**Entry**	**Bromo-aldehyde 1** [mg] (mmol)	**Boronic acid/ester 6** [mg] (mmol)	**CsF** [mg] (mmol)	**$Pd(PPh_3)_4$** [mg] (mmol)	t_1 [h]	**methylene active compound 7** [mg] (mmol)	**Organo catalyst** [mg] (mmol)	t_2 [h]	**Product** [mg] (%)
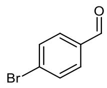	**1**								
1	185 (1.00) of **1**	134 (1.1) of **6a**	CsF 487 (3.2)	24 (0.02)	6	162 (1.1) of **7a**	77 (1.0) of NH_4OAc	6	282 (91) of **8a**
2	185 (1.00) of **1**	134 (1.1) of **6a**	CsF 486 (3.2)	24 (0.02)	6	161 (1.1) of **7c**	1 drop of Et_2NH	3	254 (82) of **8b**
3	185 (1.00) of **1**	150 (1.1) of **6b**	CsF 486 (3.2)	24 (0.02)	6	160 (1.2) of **7b**	78 (1.0) of NH_4OAc	5	254 (82) of **8c**
4	185 (1.00) of **1**	150 (1.1) of **6b**	CsF 486 (3.2)	24 (0.02)	6	178 (1.1) of **7f**	1 drop of Et_2NH	6	293 (86) of **8d**
5	185 (1.00) of **1**	150 (1.1) of **6b**	CsF 486 (3.2)	24 (0.02)	6	192 (1.1) of **7e**	1 drop of Et_2NH	4	306 (87) of **8e**

6	185 (1.00) of **1**	141 (1.1) of **6h**	CsF 486 (3.2)	24 (0.02)	6	162 (1.1) of **7a**	78 (1.0) of NH_4OAc	6	269 (85) of **8f**
7	185 (1.00) of **1**	229 (1.1) of **6l**	CsF 486 (3.2)	24 (0.02)	16	161 (1.1) of **7c**	1 drop of Et_2NH	3	223 (71) of **8g**
8	93 (0.50) of **1**	256 (1.2) of **6n**	CsF 242 (1.6)	15 (0.013)	16	81 (0.55) of **7a**	39 (0.5) of NH_4OAc	6	201 (79) of **8h**
9	93 (0.50) of **1**	256 (1.2) of **6p**	CsF 242 (1.6)	18 (0.015)	16	96 (0.55) of **7e**	1 drop of Et_2NH	4	207 (76) of **8i**

2.1.1.1. 5-(Biphenyl-4-ylmethylene)-3-methyl-2-thioxothiazolidin-4-one (8a)

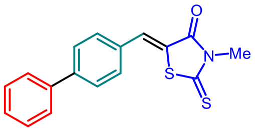

$C_{17}H_{13}NOS_2$ [311.42]

According to the GP and after purification by chromatography on silica gel (*n*-hexane/acetone 6:1) compound **8a** (282 mg, 91%) was obtained as yellow crystals, Mp 135 °C. R_f (*n*-hexan/dichlormethane 1:1) = 0.58.

^{1}H NMR (300 MHz, $CDCl_3$): δ 3.48 (s, 3 H), 7.34–7.74 (m, 10 H). ^{13}C NMR (75 MHz, $CDCl_3$): δ 31.4 (CH_3), 123.0 (C_{quat}), 127.2 (CH), 128.0 (CH), 128.4 (CH), 129.2 (CH), 131.4 (CH), 132.3 (C_{quat}), 132.8 (CH), 139.6 (C_{quat}), 143.5 (C_{quat}), 167.9 (C_{quat}), 193.5 (C_{quat}). MS (EI) *m/z* (%): 311 (43, $[M]^+$), 235 (2, $[C_{16}H_{13}NO]^+$), 210 (100, $[C_{14}H_{10}S]^+$), 165 (21, $[C_{18}H_9]^+$). IR: $\tilde{\nu}$ [cm^{-1}] = 3013 (w), 2876 (w), 1709 (m), 1670 (w), 1589 (w), 1553 (w), 1520 (w), 1422 (m), 1352 (w), 1292 (s), 1261 (m), 1126 (s), 1098 (m), 1072 (m), 1018 (m), 993 (m), 922 (m), 829 (m), 797 (m), 762 (s), 737 (m), 716 (m), 696 (m), 679 (s), 623 (m). Anal calcd for $C_{17}H_{13}NOS_2$ [311.4]: C 65.56, H 4.21, N 4.50; Found: C 65.56, H 4.00, N 4.51.

2.1.1.2. 2-(Biphenyl-4-ylmethylene)-1*H*-inden-1,3[2*H*]-dione (8b)

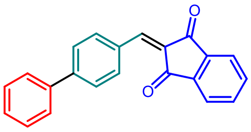

$C_{22}H_{14}O_2$ [310.35]

According to the GP and after purification by chromatography on silica gel (*n*-hexane/dichloromethane 15:1) and recrystallization from *n*-hexane/dichloromethane compound **8b** (254 mg, 82%) was obtained as a yellow voluminous solid, Mp 144 °C. R_f (*n*-hexane/dichloromethane 1:1) = 0.26.

^{1}H NMR (300 MHz, $CDCl_3$): δ 7.27–7.42 (m, 3 H), 7.54–7.60 (m, 2 H), 7.61–7.67 (m, 2 H), 7.67–7.74 (m, 2 H), 7.82 (s, 1 H), 7.87–7.95 (m, 2 H), 8.42–8.48 (m, 2 H). ^{13}C NMR (75 MHz, $CDCl_3$): δ 123.4 (CH), 123.5 (CH), 127.4 (CH), 127.5 (CH), 128.5 (CH), 129.0 (C_{quat}), 129.1

(CH), 132.3 (C_{quat}), 135.0 (CH), 135.3 (CH), 135.5 (CH), 139.9 (C_{quat}), 140.2 (C_{quat}), 142.7 (C_{quat}), 145.9 (C_{quat}), 146.5 (CH), 189.3 (C_{quat}), 190.5 (C_{quat}). MS (EI) *m/z* (%): 310 (37, $[M]^+$), 282 (3, $[C_{21}H_{14}O]^+$), 233 (21, $[C_{16}H_{10}O_2]^+$), 200 (100). IR: $\tilde{\nu}$ [cm^{-1}] = 3055 (w), 3032 (w), 1682 (s), 1616 (m), 1578 (s), 1551 (m), 1487 (w), 1450 (w), 1418 (w), 1383 (w), 1352 (m), 1335 (w), 1323 (w), 1287 (w), 1252 (w), 1219 (w), 1198 (m), 1152 (m), 1082 (m), 989 (m), 968 (w), 841 (m), 812 (w), 760 (s), 737 (s), 721 (m), 691 (m), 681 (m), 646 (w). Anal calcd for $C_{22}H_{14}O_2$ [310.4]: C 85.14, H 4.55; Found: C 84.93, H 4.62.

2.1.1.3. 5-{[4'-Methyl-(1,1'-biphenyl)-4-yl]methylene}-2-thioxothiazolidin-4-one (8c)

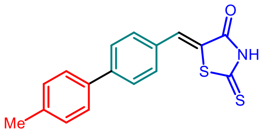

$C_{17}H_{13}NOS_2$ [311.42]

According to the GP and after purification by chromatography on silica gel (*n*-hexane/acetone 15:1) and recrystallization from ethanol compound **8c** (254 mg, 82%) was obtained as yellow crystals, Mp 243 °C. R_f (*n*-hexane/acetone 1:1) = 0.72.

^{1}H NMR (300 MHz, DMSO-d_6): δ 2.35 (s, 3 H), 7.25–7.36 (m, 2 H), 7.62–7.70 (m, 5 H), 7.81–7.86 (m, 2 H), 13.84 (br, 1 H). ^{13}C NMR (75 MHz, DMSO-d_6): δ 20.8 (CH_3), 125.0 (C_{quat}), 126.7 (CH), 127.2 (CH), 129.7 (CH), 131.2 (CH), 131.3 (CH), 131.7 (C_{quat}), 135.8 (C_{quat}), 137.9 (C_{quat}), 142.0 (C_{quat}), 169.4 (C_{quat}), 195.5 (C_{quat}). MS (EI) *m/z* (%): 311 (42, $[M]^+$), 224 (100, $[C_{15}H_{12}S]^+$). IR: $\tilde{\nu}$ [cm^{-1}] = 3098 (w), 1709 (w), 1589 (m), 1574 (w), 1500 (w), 1433 (w), 1418 (w), 1402 (w), 1344 (w), 1290 (w), 1283 (w), 1236 (w), 1167 (m), 1153 (m), 1123 (w), 1099 (w), 1059 (m), 1034 (w), 1002 (m), 980 (w), 968 (w), 932 (w), 912 (m), 851 (w), 797 (s), 745 (m), 729 (m), 721 (m), 708 (w), 677 (s), 644 (m), 627 (m). Anal calcd for $C_{17}H_{13}NOS_2$ [311.4]: C 65.56, H 4.21, N 4.50, S 20.59; Found: C 65.30, H 4.33, N 4.46, S 20.30.

2.1.1.4. 3-(4'-Methyl-[1,1'-biphenyl]-4-yl)-2-(4-nitrophenyl)acrylnitrile (8d)

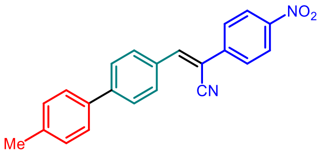

$C_{22}H_{16}N_2O_2$ [340.37]

According to the GP and after purification by chromatography on silica gel (*n*-hexane/acetone 20:1) compound **8d** (293 mg, 86%) was obtained as yellow voluminous crystals, Mp 158 °C. R_f (*n*-hexane/acetone 10:1) = 0.19.

^{1}H NMR (300 MHz, acetone-d_6/CS_2 4:1): δ 2.40 (s, 3 H), 7.31 (d, ^3J = 7.9 Hz, 2 H), 7.61–7.68 (m, 2 H), 7.80–7.87 (m, 2 H), 8.03–8.10 (m, 2 H), 8.11–8.17 (m, 2 H), 8.18 (s, 1 H), 8.30–8.42

(m, 2 H). ^{13}C NMR (75 MHz, acetone-d$_6$/CS$_2$ 4:1): δ 21.3 (CH$_3$), 109.4 (C$_{quat}$), 118.0 (C$_{quat}$), 125.1 (CH), 127.7 (CH), 127.8 (CH), 128.0 (CH), 130.6 (CH), 131.4 (CH), 133.0 (C$_{quat}$), 137.5 (C$_{quat}$), 139.0 (C$_{quat}$), 141.7 (C$_{quat}$), 144.6 (C$_{quat}$), 146.2 (CH), 148.7 (C$_{quat}$). MS (EI) *m/z* (%): 340 (100, [M]$^+$), 294 (4, [C$_{22}$H$_{16}$N]$^+$), 279 (10, [C$_{21}$H$_{13}$N]$^+$), 91 (2, [C$_7$H$_7$]$^+$). IR: $\tilde{\nu}$ [cm^{-1}] = 3030 (w), 2968 (w), 2920 (w), 2214 (w), 1584 (m), 1506 (m), 1497 (m), 1447 (w), 1418 (w), 1387 (w), 1328 (s), 1283 (w), 1227 (w), 1196 (m), 1182 (m), 1109 (m), 1034 (w), 1002 (w), 955 (w), 912 (m), 855 (s), 806 (s), 797 (m), 775 (w), 750 (m), 733 (m), 692 (m), 673 (w). Anal calcd for C$_{22}$H$_{16}$N$_2$O$_2$ [340.4]: C 77.63, H 4.74, N 8.23; Found: C 77.66, H 4.73, N 8.13.

2.1.1.5. 3-Methyl-4-{[4'-methyl-(1,1'-biphenyl)-4-yl]methylene}-1-phenyl-1*H*-pyrazol-5[4*H*]-one (8e)

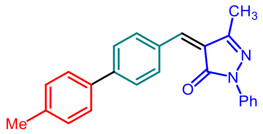

C$_{24}$H$_{20}$N$_2$O [352.43]

According to the GP and after purification by chromatography on silica gel (*n*-hexane/acetone 20:1) compound **8e** (306 mg, 87%) was obtained as an orange red solid, Mp 105–109 °C. R$_f$ (*n*-hexane/acetone 10:1) = 0.18.

^{1}H NMR (600 MHz, acetone-d$_6$/CS$_2$ 4:1): δ 2.38 (s, 3 H), 2.41 (s, 3 H), 7.16 (dt, ^3J = 7.4 Hz, ^4J = 1.1 Hz, 1 H), 7.31 (d, ^3J = 7.8 Hz, 2 H), 7.40 (t, ^3J = 8.0 Hz, 2 H), 7.65 (d, ^3J = 7.7 Hz, 2 H), 7.69 (s, 1 H), 7.79 (d, ^3J = 8.5 Hz, 2 H), 8.04 (d, ^3J = 8.7 Hz, 2 H), 8.73 (d, ^3J = 8.1 Hz, 2 H). ^{13}C NMR (150 MHz, acetone-d$_6$/CS$_2$ 4:1): δ 13.6 (CH$_3$), 21.4 (CH$_3$), 119.1 (CH), 125.0 (CH), 127.4 (CH), 127.8 (CH), 129.4 (CH), 130.6 (CH), 133.1 (C$_{quat}$), 135.1 (C$_{quat}$), 135.6 (CH), 137.5 (C$_{quat}$), 139.1 (C$_{quat}$), 139.7 (C$_{quat}$), 145.9 (C$_{quat}$), 147.3 (CH), 151.8 (C$_{quat}$), 162.5 (C$_{quat}$). MS (EI) *m/z* (%): 352 (100, [M]$^+$), 261 (13, [C$_{17}$H$_{13}$N$_2$O]$^+$), 185 (45, [C$_{11}$H$_9$N$_2$O]$^+$), 91 (6, [C$_7$H$_7$]$^+$), 77 (8, [C$_6$H$_5$]$^+$). IR: $\tilde{\nu}$ [cm^{-1}] = 3030 (w), 2914 (w), 1680 (m), 1614 (w), 1591 (m), 1560 (w), 1547 (m), 1493 (m), 1410 (w), 1366 (w), 1314 (s), 1196 (m), 1148 (m), 1136 (m), 1111 (w), 1099 (w), 1034 (w), 995 (m), 922 (w), 855 (m), 806 (s), 768 (m), 747 (s), 689 (m), 665 (m), 646 (w). Anal calcd for C$_{24}$H$_{20}$N$_2$O [352.4]: C 81.79, H 5.72, N 7.95; Found: C 81.68, H 5.81, N 7.85.

2.1.1.6. 3-Methyl-5-(4-(thiophen-3-yl)benzylidene)-2-thioxothiazolidin-4-one (8f)

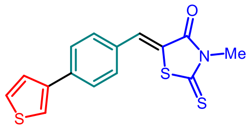

C$_{15}$H$_{11}$NOS$_3$ [317.45]

According to the GP and after purification by chromatography on silica gel (*n*-hexane/dichloromethane 3:2) and crystallization from *n*-hexane/dichloromethane compound **8f** (269 mg, 85%) was obtained as yellow orange crystals, Mp 205 °C. R_f (*n*-hexane/dichloromethane 1:1) = 0.45.

^{1}H NMR (300 MHz, CD_2Cl_2): δ 3.50 (s, 3 H), 7.45–7.48 (m, 2 H), 7.54–7.59 (m, 2 H), 7.63 (dd, ^{4}J = 2.6 Hz, ^{4}J = 1.7 Hz, 1 H), 7.73–7.75 (m, 3 H). ^{13}C NMR (75 MHz, CD_2Cl_2): δ 31.7 (CH_3), 122.6 (CH), 123.2 (C_{quat}), 126.5 (CH), 127.4 (CH), 127.6 (CH), 131.9 (CH), 132.6 (C_{quat}), 133.1 (CH), 138.4 (C_{quat}), 141.4 (C_{quat}), 168.3 (C_{quat}), 194.2 (C_{quat}). MS (EI) *m/z* (%): 317 (51, $[M]^+$), 301 (11, $[C_{14}H_8NOS_3]^+$), 216 (100, $[C_{12}H_8S_2]^+$). IR: $\tilde{\nu}$ [cm^{-1}] = 3094 (w), 3007 (w), 2938 (w), 1707 (m), 1678 (m), 1587 (w), 1557 (w), 1530 (w), 1497 (w), 1452 (w), 1422 (m), 1352 (w), 1290 (s), 1229 (w), 1198 (w), 1125 (m), 1099 (m), 1088 (m), 1014 (w), 993 (w), 922 (w), 864 (w), 842 (m), 781 (s), 718 (s), 681 (m), 646 (w), 621 (w). Anal calcd for $C_{15}H_{11}NOS_3$ [317.5]: C 56.75, H 3.49, N 4.41; Found: C 56.95, H 3.34, N 4.37.

2.1.1.7. 2-[4-(1-Methyl-1*H*-pyrazol-4-yl)benzylidene]-1*H*-inden-1,3[2*H*]-dione (8g)

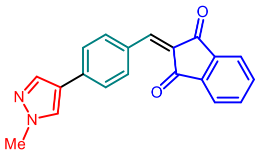

$C_{20}H_{14}N_2O_2$ [314.34]

According to the GP and after purification by chromatography on silica gel (dichloromethane/dichloromethane/methanol 40:1) and crystallization from *n*-hexane/ dichloromethane compound **8g** (223 mg, 71%) was obtained as a yellow orange voluminous solid, Mp 209 °C. R_f (dichloromethane/methanol 15:1) = 0.65.

^{1}H NMR (600 MHz, CD_2Cl_2): δ 3.94 (s, 3 H), 7.62–7.67 (m, 2 H), 7.79 (s, 1 H), 7.80–7.87 (m, 4 H), 7.94–8.03 (m, 2 H), 8.48–8.54 (m, 2 H). ^{13}C NMR (150 MHz, CD_2Cl_2): δ 39.7 (CH_3), 122.6 (C_{quat}), 123.5 (CH), 123.6 (CH), 125.7 (CH), 128.4 (CH), 128.9 (C_{quat}), 131.8 (C_{quat}), 135.65 (CH), 135.73 (CH), 135.8 (CH), 137.5 (CH), 138.4 (C_{quat}), 140.6 (C_{quat}), 143.1 (C_{quat}), 146.5 (CH), 189.7 (C_{quat}), 190.7 (C_{quat}). MS (EI) *m/z* (%): 314 (97, $[M]^+$), 313 (100, $[M-H]^+$), 286 (6, $[C_{19}H_{14}N_2O]^+$), 233 (3, $[C_{16}H_9O_2]^+$), 157 (5, $[C_{10}H_9N_2]^+$), 129 (2, $[C_9H_5O]^+$), 104 (3, $[C_7H_4O]^+$), 76 (4, $[C_6H_4]^+$). IR: $\tilde{\nu}$ [cm^{-1}] = 3107 (w), 3084 (w), 1717 (w), 1672 (s), 1626 (w), 1593 (w), 1578 (m), 1556 (s), 1541 (s), 1510 (m), 1469 (w), 1439 (m), 1415 (w), 1379 (m), 1354 (m), 1339 (m), 1321 (m), 1310 (w), 1285 (w), 1256 (w), 1213 (w), 1190 (s), 1159 (m), 1088 (m), 1074 (m), 1059 (w), 993 (m), 982 (w), 949 (s), 833 (m), 810 (m), 743 (s), 733 (w). Anal calcd for $C_{20}H_{14}N_2O_2$ [314.3]: C 76.42, H 4.49, N 8.91; Found: C 76.24, H 4.45, N 8.83.

2.1.1.8. 5-[4-(10-Hexyl-10*H*-phenothiazin-3-yl)benzylidene]-3-methyl-2-thioxothiazolidin-4-one (8h)

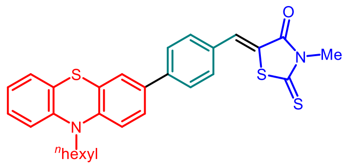

$C_{29}H_{28}N_2OS_3$ [516.74]

According to the GP and after purification by chromatography on silica gel (*n*-hexane/acetone 20:1) and drying und vacuo compound **8h** (201 mg, 79%) was obtained as orange red crystal needles, Mp 130 °C. R_f (*n*-hexane/acetone 10:1) = 0.42.

^{1}H NMR (300 MHz, acetone-d_6/CS_2 4:1): δ 0.86–0.97 (m, 3 H), 1.29–1.39 (m, 4 H), 1.45–1.57 (m, 2 H), 1.85 (quin, ^3J = 7.6 Hz, 2 H), 3.49 (s, 3 H), 3.95 (d, ^3J = 7.1 Hz, 2 H), 6.88–6.98 (m, 2 H), 7.02 (d, ^3J = 8.5 Hz, 1 H), 7.10 (dd, ^3J = 7.6 Hz, ^4J = 1.6 Hz, 1 H), 7.17 (ddd, ^3J = 8.2 Hz, ^3J = 7.2 Hz, ^4J = 1.6 Hz, 1 H), 7.43 (d, ^4J = 2.2 Hz, 1 H), 7.52 (dd, ^3J = 8.5 Hz, ^4J = 2.2 Hz, 1 H), 7.61–7.67 (m, 2 H), 7.72–7.79 (m, 3 H). ^{13}C NMR (75 MHz, acetone-d_6/CS_2 4:1): δ 14.7 (CH_3), 23.7 (CH_2), 27.5 (CH_2), 27.7 (CH_2), 31.6 (CH_3), 32.5 (CH_2), 48.1 (CH_2), 116.4 (CH), 116.6 (CH), 123.3 (C_{quat}), 123.4 (CH), 124.8 (C_{quat}), 126.1 (CH), 126.2 (C_{quat}), 126.8 (CH), 127.7 (CH), 128.0 (CH), 128.2 (CH), 132.2 (CH), 132.7 (C_{quat}), 133.0 (CH), 134.1 (C_{quat}), 142.6 (C_{quat}), 145.3 (C_{quat}), 146.1 (C_{quat}), 167.7 (C_{quat}), 193.6 (C_{quat}). MS (MALDI-TOF) calcd for $C_{29}H_{28}N_2OS_3$ *m/z:* 516.14; Found: 516.2 ($[M]^+$). IR: $\tilde{\nu}$ [cm^{-1}] = 3063 (w), 2957 (w), 2924 (w), 2853 (w), 1713 (s), 1587 (m), 1572 (M), 1545 (w), 1493 (w), 1464 (s), 1441 (m), 1425 (m), 1395 (w), 1358 (m), 1290 (s), 1277 (s), 1248 (s), 1223 (w), 1194 (m), 1180 (w), 1128 (m), 1123 (s), 1101 (m), 1047 (w), 1038 (w), 991 (w), 955 (w), 907 (w), 878 (w), 872 (w), 813 (w), 808 (s), 721 (s), 718 (m), 716 (m), 667 (w). Anal calcd for $C_{29}H_{28}N_2OS_3$ [516.7]: C 67.41, H 5.46, N 5.42, S 18.62; Found: C 67.17, H 5.42, N 5.28, S 18.33.

2.1.1.9. 4-[4-(10-Hexyl-10*H*-phenothiazin-3-yl)benzylidene]-3-methyl-1-phenyl-1*H*-pyrazol-5[4*H*]-one (8i)

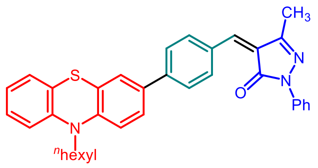

$C_{35}H_{33}N_3OS$ [543.72]

According to the GP and after purification by chromatography on silica gel (*n*-hexane/acetone 30:1) and drying under vacuo compound **8i** (207 mg, 76%) was obtained as a crystalline black red solid, Mp 58–61 °C. R_f (*n*-hexane/acetone 10:1) = 0.29.

^{1}H NMR (300 MHz, acetone-d$_6$/CS$_2$ 4:1): δ 0.86–0.94 (m, 3 H), 1.28–1.41 (m, 4 H), 1.43–1.56 (m, 2 H), 1.84 (quin, ^3J = 7.6 Hz, 2 H), 2.37 (s, 3 H), 3.96 (d, ^3J = 7.0 Hz, 2 H), 6.94 (dt, ^3J = 7.4 Hz, ^4J = 1.2 Hz, 1 H), 6.99 (d, ^3J = 7.7 Hz, 1 H), 7.06 (d, ^3J = 8.5 Hz, 1 H), 7.11 (d, ^4J = 1.6 Hz, 1 H), 7.12–7.22 (m, 2 H), 7.36–7.45 (m, 2 H), 7.51 (d, ^4J = 2.2 Hz, 1 H), 7.59 (dd, ^3J = 8.5 Hz, ^4J = 2.2 Hz, 1 H), 7.67 (s, 1 H), 7.73–7.80 (m, 2 H), 8.01–8.08 (m, 2 H), 8.68–8.75 (m, 2 H). ^{13}C NMR (75 MHz, acetone-d$_6$/CS$_2$ 4:1): δ 13.6 (CH$_3$), 14.5 (CH$_3$), 23.5 (CH$_2$), 27.4 (CH$_2$), 27.6 (CH$_2$), 32.4 (CH$_2$), 48.1 (CH$_2$), 116.6 (CH), 116.7 (CH), 119.1 (CH), 123.5 (CH), 124.9 (C$_{quat}$), 125.0 (CH), 126.1 (C$_{quat}$), 126.3 (CH), 126.8 (CH), 127.1 (CH), 127.6 (C$_{quat}$), 128.0 (CH), 128.3 (CH), 129.4 (CH), 133.0 (C$_{quat}$), 134.3 (C$_{quat}$), 135.7 (CH), 139.8 (C$_{quat}$), 144.7 (C$_{quat}$), 145.4 (C$_{quat}$), 146.4 (C$_{quat}$), 147.3 (CH), 151.9 (C$_{quat}$), 162.6 (C$_{quat}$). MS (MALDI-TOF) calcd for C$_{35}$H$_{33}$N$_3$OS *m/z:* 543.72; Found: 543.2 ([M]$^+$). IR: $\tilde{\nu}$ [cm^{-1}] = 2953 (w), 2924 (w), 2855 (w), 1680 (w), 1616 (w), 1586 (m), 1574 (m), 1559 (m), 1541 (w), 1497 (m), 1460 (s), 1443 (m), 1427 (w), 1395 (w), 1362 (m), 1316 (s), 1289 (m), 1250 (m), 1225 (w), 1192 (m), 1142 (m), 1119 (m), 1111 (w), 1040 (w), 995 (m), 963 (w), 922 (w), 899 (w), 883 (w), 851 (w), 808 (m), 753 (m), 748 (s), 691 (m), 667 (m), 640 (w). Anal calcd for C$_{35}$H$_{33}$N$_3$OS [543.7]: C 77.31, H 6.12, N 7.73, S 5.90; Found: C 77.38, H 6.05, N 7.68, S 5.75.

2.1.2. Consecutive Three-component Suzuki-Knoevenagel Synthesis of Thienylene-bridged Systems 9

Table S2. Experimental details of the consecutive three-component Suzuki-Knoevenagel synthesis of thienylene-bridged systems **9**

**Entry**	**Bromo-aldehyde 2** [mg] (mmol)	**Boronic acid/ester 6** [mg] (mmol)	**CsF** [mg] (mmol)	**Pd(PPh$_3$)$_4$** [mg] (mmol)	***t$_1$*** [h]	**methylene active compound 7** [mg] (mmol)	**Organo catalyst** [mg] (mmol)	***t$_2$*** [h]	**Product** [mg] (%)
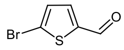 **2**									
1	191 (1.00) of **2**	150 (1.1) of **6b**	486 (3.2)	24 (0.02)	6	162 (1.10) of **7a**	78 (1.0) of NH$_4$OAc	5	251 (76) of **9a**
2	190 (1.00) of **2**	150 (1.1) of **6b**	486 (3.2)	24 (0.02)	8	161 (1.10) of **7c**	1 drop of Et$_2$NH	3	264 (80) of **9b**
3	190 (1.00) of **2**	150 (1.1) of **6b**	486 (3.2)	24 (0.02)	6	254 (1.20) of **7d**	1 drop of Et$_2$NH	8	337 (85) of **9c**
4	190 (1.00) of **2**	150 (1.1) of **6b**	486 (3.2)	24 (0.02)	8	192 (1.10) of **7e**	1 drop of Et$_2$NH	4	290 (81) of **9d**
5	115 (0.60) of **2**	90 (1.1) of **6b**	292 (3.2)	21 (0.02)	8	178 (0.66) of **7f**	46 (0.6) of NH$_4$OAc	6	94 (45) of **9e**
6	115 (0.60) of of **2**	267 (1.2) of **6d**	291 (3.2)	21 (0.02)	8	98 (0.66) of **7a**	46 (0.6) of NH$_4$OAc	5	174 (60) of **9f**
7	95 (0.50) of **2**	256 (1.2) of **6n**	242 (3.2)	15 (0.013)	16	96 (0.55) of of **7e**	1 drop of Et$_2$NH	4	218 (79) of **9g**

2.1.2.1. 3-Methyl-2-thioxo-5-{[5-(*p*-tolyl)thiophen-2-yl]methylene}thiazolidin-4-one (9a)

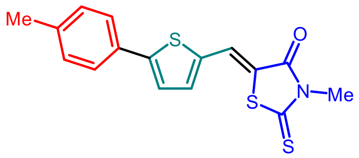

$C_{16}H_{13}NOS_3$ [331.48]

According to the GP and after purification by chromatography on silica gel (*n*-hexane/acetone 15:1) and drying under vacuo compound **9a** (251 mg, 76%) was obtained as a crystalline orange brown solid, Mp 212 °C. R$_f$ (*n*-hexane/acetone 7:1) = 0.38.

^{1}H NMR (300 MHz, DMSO-d$_6$/CS$_2$ 8:1): δ 2.38 (s, 3 H), 3.43 (s, 3 H), 7.24–7.31 (m, 2 H), 7.63–7.69 (m, 3 H), 7.74 (dd, ^3J = 4.0 Hz, ^4J = 0.7 Hz, 1 H), 8.07 (d, ^4J = 0.7 Hz, 1 H). ^{13}C NMR (75 MHz, DMSO-d$_6$/CS$_2$ 8:1): δ 20.9 (CH$_3$), 31.1 (CH$_3$), 119.4 (C$_{quat}$), 125.1 (CH), 125.7 (CH), 125.9 (CH), 129.7 (CH), 129.8 (C$_{quat}$), 136.1 (C$_{quat}$), 137.4 (CH), 138.8 (C$_{quat}$), 152.0 (C$_{quat}$), 166.4 (C$_{quat}$), 191.7 (C$_{quat}$). MS (EI) *m/z* (%): 331 (59, [M]$^+$), 230 (100, [$C_{13}H_{10}S$]$^+$). IR: $\tilde{\nu}$ [cm^{-1}] = 3007 (w), 2914 (w), 1694 (s), 1578 (s), 1435 (m), 1421 (m), 1350 (m), 1289 (s), 1265 (s), 1175 (m), 1120 (m), 1117 (m), 1053 (m), 991 (m), 951 (m), 936 (w), 905 (w), 882 (w), 819 (w), 810 (w), 785 (s), 731 (m), 706 (w), 631 (m). Anal calcd for $C_{16}H_{13}NOS_3$ [331.5]: C 57.97, H 3.95, N 4.23, S 29.02; Found: C 57.73, H 3.89, N 4.26, S 28.75.

2.1.2.2. 2-{[5-(*p*-Tolyl)thiophen-2-yl]methylene}-1*H*-inden-1,3[2*H*]-dione (9b)

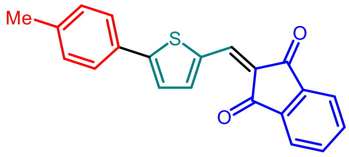

$C_{21}H_{14}O_2S$ [330.40]

According to the GP and after purification by chromatography on silica gel (*n*-hexane/acetone 15:1) and drying under vacuo compound **9b** (264 mg, 80%) was obtained as an orange voluminous solid, Mp 154 °C. R$_f$ (*n*-hexane/acetone 4:1) = 0.20.

^{1}H NMR (300 MHz, acetone-d$_6$/CS$_2$ 4:1): δ 2.43 (s, 3 H), 7.28–7.34 (m, 2 H), 7.58 (d, ^3J = 4.1 Hz, 1 H), 7.69–7.75 (m, 2 H), 7.85–7.97 (m, 5 H), 8.17 (dd, ^3J = 4.1 Hz, ^4J = 0.6 Hz, 1 H). ^{13}C NMR (75 MHz, acetone-d$_6$/CS$_2$ 4:1): δ 21.7 (CH$_3$), 123.37 (CH), 123.43 (CH), 124.8 (C$_{quat}$), 125.4 (CH), 127.2 (CH), 130.8 (CH), 131.5 (C$_{quat}$), 135.6 (CH), 135.8 (CH), 136.1 (CH), 137.2 (C$_{quat}$), 140.4 (C$_{quat}$), 141.2 (C$_{quat}$), 142.8 (C$_{quat}$), 144.5 (CH), 157.7 (C$_{quat}$), 189.3 (C$_{quat}$), 189.6 (C$_{quat}$). MS (MALDI-TOF) calcd for $C_{21}H_{14}O_2S$ *m/z:* 330.07; Found: 330.7 ([M]$^+$). IR: $\tilde{\nu}$ [cm^{-1}] = 1719 (w), 1678 (s), 1628 (w), 1600 (m), 1580 (s), 1524 (w), 1493 (w), 1437 (m), 1416 (w), 1379 (m), 1356 (w), 1341 (w), 1325 (w), 1233 (w), 1211 (m), 1198 (w), 1179 (w), 1159 (w), 1126 (w),

1098 (m), 1076 (w), 1026 (w), 993 (w), 947 (w), 824 (w), 799 (s), 783 (w), 733 (s), 642 (w). Anal calcd for $C_{21}H_{14}O_2S$ [330.4]: C 76.34, H 4.27; Found: C 76.07, H 4.38.

2.1.2.3. 1,3-Dipropyl-5-{[5-(*p*-tolyl)thiophen-2-yl]methylene}pyrimidin-2,4,6[1*H*,3*H*,5*H*]-trione (9c)

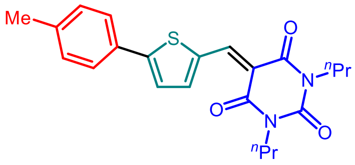

$C_{22}H_{24}N_2O_3S$ [396.50]

According to the GP and after purification by chromatography on silica gel (*n*-hexane/acetone 25:1) and drying under vacuo compound **9c** (337 mg, 85%) was obtained as yellow crystalline needles, Mp 155 °C. R_f (*n*-hexane/acetone 10:1) = 0.32.

^{1}H NMR (300 MHz, acetone-d$_6$/CS_2 4:1): δ 0.95 (t, ^3J = 7.5 Hz, 3 H), 0.99 (t, ^3J = 7.5 Hz, 3 H), 1.58–1.77 (m, 4 H), 2.42 (s, 3 H), 3.84–3.95 (m, 4 H), 7.30 (d, ^3J = 8.1 Hz, 2 H), 7.60 (d, ^3J = 4.1 Hz, 1 H), 7.68–7.76 (m, 2 H), 7.99 (d, ^3J = 4.1 Hz, 1 H), 8.55 (s, 1 H). ^{13}C NMR (300 MHz, acetone-d$_6$/CS_2 4:1): δ 11.8 (CH_3), 11.9 (CH_3), 21.7 (CH_3), 22.17 (CH_2), 22.21 (CH_2), 43.4 (CH_2), 44.0 (CH_2), 110.9 (C_{quat}), 125.0 (CH), 127.2 (CH), 130.8 (CH), 131.5 (C_{quat}), 136.8 (C_{quat}), 140.7 (C_{quat}), 147.5 (CH), 148.1 (CH), 151.3 (C_{quat}), 160.3 (C_{quat}), 162.1 (C_{quat}), 162.5 (C_{quat}). MS (MALDI-TOF) calcd for $C_{22}H_{24}N_2O_3S$ *m/z:* 396.15; Found: 397.0 ([MH]$^+$). IR: $\tilde{\nu}$ [cm^{-1}] = 2973 (w), 2970 (m), 2901 (w), 1649 (s), 1551 (m), 1520 (w), 1490 (w), 1452 (w), 1441 (w), 1398 (s), 1368 (m), 1340 (w), 1310 (m), 1255 (w), 1238 (m), 1201 (w), 1153 (m), 1107 (m), 1074 (s), 1057 (m), 1005 (w), 893 (w), 800 (m), 790 (m), 660 (w), 600 (w). Anal calcd for $C_{22}H_{24}N_2O_3S$ [396.5]: C 66.64, H 6.10, N 7.07, S 8.09; Found: C 66.57, H 5.92, N 7.15, S 7.99.

2.1.2.4. 3-Methyl-1-phenyl-4-{[5-(*p*-tolyl)thiophen-2-yl]methylene}-1H-pyrazol-5[4*H*]-one (9d)

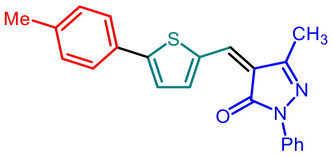

$C_{22}H_{18}N_2OS$ [358.46]

According to the GP and after purification by chromatography on silica gel (*n*-hexane/acetone 10:1) and drying under vacuo compound **9d** (290 mg, 81%) was obtained as a red solid, Mp 133 °C. R_f (*n*-hexane/acetone 4:1) = 0.27.

^{1}H NMR (600 MHz, acetone-d$_6$/CS_2): δ 2.35 (s, 3 H), 2.41 (s, 3 H), 7.41 (tt, ^3J = 7.4 Hz, ^4J = 1.1 Hz, 1 H), 7.27–7.31 (m, 2 H), 7.37–7.42 (m, 2 H), 7.56 (d, ^3J = 4.0 Hz, 1 H), 7.68–7.72 (m,

2 H), 7.86 (s, 1 H), 8.05–8.08 (m, 2 H), 8.11 (d, ^3J = 4.0 Hz, 1 H). ^{13}C NMR (150 MHz, acetone-d_6/CS_2): δ 13.3 (CH_3), 21.6 (CH_3), 118.7 (CH), 122.1 (C_{quat}), 124.7 (CH), 124.9 (CH), 127.1 (CH), 129.3 (CH), 130.7 (CH), 131.6 (C_{quat}), 136.7 (C_{quat}), 137.2 (CH), 139.9 (C_{quat}), 140.3 (C_{quat}), 143.7 (CH), 150.7 (C_{quat}), 157.4 (C_{quat}), 162.9 (C_{quat}). MS (MALDI-TOF) calcd for $C_{22}H_{18}N_2OS$ *m/z:* 358.11; Found: 359.1 ([MH]$^+$). IR: $\tilde{\nu}$ [cm^{-1}] = 2980 (w), 2972 (w), 2918 (w), 2851 (w), 1678 (s), 1653 (w), 1593 (m), 1557 (m), 1500 (m), 1489 (m), 1431 (m), 1410 (m), 1375 (m), 1360 (m), 1335 (w), 1314 (m), 1304 (m), 1255 (w), 1213 (m), 1142 (m), 1096 (w), 1078 (m), 1024 (m), 1001 (m), 957 (w), 926 (m), 912 (w), 891 (w), 804 (s), 797 (s), 762 (m), 756 (s), 729 (w), 689 (s), 673 (m), 658 (m), 619 (w). Anal calcd for $C_{22}H_{18}N_2OS$ [358.5]: C 73.71, H 5.06, N 7.82, S 8.95; Found: C 73.63, H 5.09, N 7.86, S 8.98.

2.1.2.5. 2-(4-Nitrophenyl)-3-[5-(*p*-tolyl)thiophen-2-yl]acrylonitrile (9e)

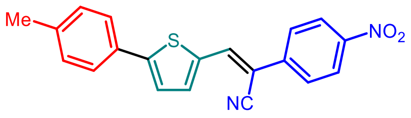

$C_{20}H_{14}N_2O_2S$ [346.40]

According to the GP and after purification by chromatography on silica gel (*n*-hexane/acetone 15:1) and crystallization from a *n*-hexane/dichloromethane solution compound **9e** (94 mg, 45%) was obtained as a yellow golden amorphous voluminous solid, Mp 245 °C. R_f (*n*-hexane/acetone 10:1) = 0.13.

^{1}H NMR (300 MHz, DMSO-d_6): δ 2.37 (s, 3 H), 7.30 (d, ^3J = 7.7 Hz, 2 H), 7.63–7.73 (m, 3 H), 7.86 (d, ^3J = 3.9 Hz, 1 H), 8.00 (d, ^3J = 8.5 Hz, 2 H), 8.34 (d, ^3J = 8.5 Hz, 2 H), 8.56 (s, 1 H). ^{13}C NMR (75 MHz, DMSO-d_6): δ 20.9 (CH_3), 103.2 (C_{quat}), 117.5 (C_{quat}), 124.3 (CH), 124.35 (CH), 124.39 (C_{quat}), 125.9 (CH), 126.3 (CH), 129.86 (C_{quat}), 129.91 (CH), 135.9 (C_{quat}), 138.4 (CH), 138.9 (CH), 139.0 (C_{quat}), 140.0 (C_{quat}), 146.8 (C_{quat}). MS (MALDI-TOF) calcd for $C_{20}H_{14}N_2O_2S$ *m/z:* 346.07; Found: 346.3 ([M]$^+$). IR: $\tilde{\nu}$ [cm^{-1}] = 3674 (w), 2988 (m), 2972 (m), 2901 (m), 1600 (w), 1572 (w), 1512 (m), 1502 (m), 1493 (m), 1443 (m), 1410 (m), 1373 (w), 1339 (m), 1269 (w), 1236 (m), 1165 (w), 1111 (m), 1067 (s), 1057 (m), 1044 (m), 1013 (w), 902 (m), 820 (w), 801 (s), 748 (m), 687 (m). Anal calcd for $C_{20}H_{14}N_2O_2S$ [346.4]: C 69.35, H 4.07, N 8.09, S 9.26; Found: C 69.13, H 4.02, N 8.04, S 9.46.

2.1.2.6. 5-{[5-(4-{Diphenylamino}phenyl)thiophen-2-yl]methylene}-3-methyl-2-thioxothiazolidin-4-one (9f)

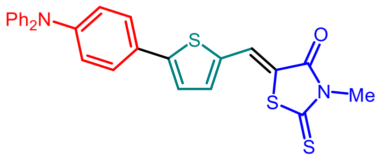

$C_{27}H_{20}N_2OS_3$ [484.66]

According to the GP and after purification by chromatography on silica gel (*n*-hexane/acetone 20:1) and drying under vacuo compound **9f** (174 mg, 60%) was obtained as a red amorphous solid, Mp 150–154 °C. R_f (*n*-hexane/acetone 10:1) = 0.39.

^{1}H NMR (300 MHz, acetone-d$_6$/CS$_2$ 4:1): δ 3.46 (s, 3 H), 7.03–7.08 (m, 2 H), 7.09–7.15 (m, 6 H), 7.28–7.35 (m, 4 H), 7.44 (d, ^3J = 4.0 Hz, 1 H), 7.56 (dd, ^3J = 4.0 Hz, ^4J = 0.7 Hz, 1 H), 7.58–7.62 (m, 2 H), 7.87 (d, ^4J = 0.6 Hz, 1 H). ^{13}C NMR (75 MHz, acetone-d$_6$/CS$_2$ 4:1): δ 31.6 (CH$_3$), 120.5 (C$_{quat}$), 123.1 (CH), 124.7 (CH), 124.8 (CH), 125.8 (CH), 126.1 (CH), 127.1 (C$_{quat}$), 127.7 (CH), 130.3 (CH), 136.9 (C$_{quat}$), 137.2 (CH), 147.6 (C$_{quat}$), 149.4 (C$_{quat}$), 153.3 (C$_{quat}$), 167.2 (C$_{quat}$), 192.5 (C$_{quat}$). MS (MALDI-TOF) calcd for $C_{27}H_{20}N_2OS_3$ *m/z:* 484.07; Found: 484.1 ([M]$^+$). IR: $\tilde{\nu}$ [cm^{-1}] = 3063 (w), 3030 (w), 3011 (w), 1697 (s), 1578 (s), 1526 (w), 1485 (m), 1431 (m), 1420 (m), 1389 (w), 1348 (m), 1329 (m), 1289 (s), 1283 (s), 1269 (s), 1196 (w), 1173 (m), 1126 (m), 1076 (w), 1051 (m), 1030 (w), 989 (w), 951 (w), 883 (w), 826 (w), 806 (w), 783 (m), 747 (s), 729 (m), 694 (s), 631 (m), 621 (w). Anal calcd for $C_{27}H_{20}N_2OS_3$ [484.7]: C 66.91, H 4.16, N 5.78, S 19.85; Found: C 66.69, H 4.26, N 5.61, S 19.87.

2.1.2.7. 4-{[5-(10-Hexyl-10*H*-phenothiazin-3-yl)thiophen-2-yl]methylene}-3-methyl-1-phenyl-1H-pyrazol-5[4*H*]-one (9g)

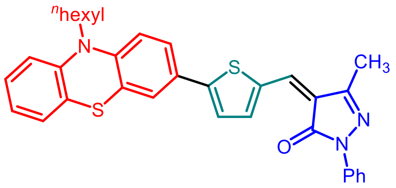

$C_{33}H_{31}N_3OS_2$ [549.75]

According to the GP and after purification by chromatography on silica gel (*n*-hexane/acetone 30:1) and drying under vacuo compound **9g** (218 mg, 79%) was obtained as a dark brown amorphous solid, Mp 61 °C. R_f (*n*-hexane/acetone 10:1) = 0.23.

^{1}H NMR (300 MHz, acetone-d$_6$/CS$_2$ 4:1): δ 0.86–0.94 (m, 3 H), 1.28–1.42 (m, 4 H), 1.43–1.57 (m, 2 H), 1.84 (quin, ^3J = 7.6 Hz, 2 H), 2.33 (s, 3 H), 3.96 (t, ^3J = 7.1 Hz, 2 H), 6.95 (dt, ^3J = 7.5 Hz, ^4J = 1.2 Hz, 1 H), 6.98–7.05 (m, 2 H), 7.12 (dd, ^3J = 7.5 Hz, ^4J = 1.5 Hz, 2 H), 7.15–7.22 (m, 1 H), 7.36–7.44 (m, 2 H), 7.52 (d, ^4J = 1.3 Hz, 1 H), 7.53 (d, ^3J = 0.7 Hz, 1 H), 7.61 (dd, ^3J = 8.5 Hz, ^4J = 2.2 Hz, 1 H), 7.85 (d, ^3J = 0.6 Hz, 1 H), 8.05–8.10 (m, 3 H). ^{13}C NMR (75 MHz, acetone-d$_6$/CS$_2$ 4:1): δ 13.3 (CH$_3$), 14.5 (CH$_3$), 23.5 (CH$_2$), 27.3 (CH$_2$), 27.6 (CH$_2$), 32.4 (CH$_2$), 48.2 (CH$_2$), 116.7 (CH),[1] 118.7 (CH), 121.8 (C$_{quat}$), 123.7 (CH), 124.49 (C$_{quat}$), 124.54 (CH), 124.7 (CH), 125.4 (CH), 126.2 (C$_{quat}$), 126.6 (CH), 128.1 (CH), 128.4 (CH), 128.6 (C$_{quat}$), 129.4 (CH), 136.4 (C$_{quat}$), 137.2 (CH), 140.0 (C$_{quat}$), 144.0 (CH), 145.1 (C$_{quat}$), 147.0 (C$_{quat}$), 150.8

[1] Two CH signals coincide.

(C_{quat}), 156.6 (C_{quat}), 163.0 (C_{quat}). MS (MALDI-TOF) calcd for $C_{33}H_{31}N_3OS_2$ *m/z:* 549.19; Found: 550.2 ($[MH]^+$). IR: $\tilde{\nu}$ [cm^{-1}] = 2953 (w), 2924 (w), 2853 (w), 1674 (m), 1591 (s), 1574 (m), 1557 (w), 1497 (m), 1466 (m), 1425 (s), 1404 (m), 1364 (m), 1333 (m), 1312 (s), 1271 (m), 1252 (m), 1219 (m), 1196 (w), 1140 (m), 1109 (w), 1074 (m), 1057 (w), 1024 (w), 999 (m), 918 (w), 905 (w), 892 (w), 876 (w), 797 (m), 748 (s), 691 (m), 665 (w), 662 (w). Anal calcd for $C_{33}H_{31}N_3OS_2$ [549.8]: C 72.10, H 5.68, N 7.64, S 11.67; Found: C 72.24, H 5.82, N 7.52, S 11.53.

2.1.3. Consecutive Three-component Suzuki-Knoevenagel Synthesis of 4-^{n}Ocytyloxy-substituted Thienylene-bridged Systems 9

Table S3. Experimental details of the consecutive three-component Suzuki-Knoevenagel synthesis of 4-^{n}ocytyloxy-substituted thienylene-bridged systems **10**.

Entry	**Bromo-aldehyde 3** [mg] (mmol)	**Boronic acid/ester 6** [mg] (mmol)	**CsF** [mg] (mmol)	**$Pd(PPh_3)_4$** [mg] (mmol)	t_1 [h]	**methylene active compound 7** [mg] (mmol)	**Organo catalyst** [mg] (mmol)	t_2 [h]	**Product** [mg] (%)
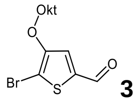 **3**									
31	256 (0.80) of **3**	131 (0.96) of **6b**	389 (2.56)	28 (0.024)	16	167 (0.96) of **7e**	1 drop of Et_2NH	4	268 (69) of **10a**
37	192 (0.60) of **3**	98 (0.72) of **6b**	292 (1.92)	21 (0.018)	16	156 (0.78) of **7g**	46 (0.60) of NH_4OAc	5	169 (55) of **10b**
25	160 (0.50) of **3**	276 (0.60) of **6f**	243 (1.60)	17 (0.015)	16	80 (0.60) of **7b**	39 (0.50) of NH_4OAc	5	204 (59) of **10c**
26	192 (0.60) of **3**	311 (0.72) of **6e**	292 (1.92)	21 (0.018)	16	96 (0.72) of **7b**	46 (0.60) of NH_4OAc	5	355 (90) of **10d**
32	320 (1.00) of **3**	519 (1.20) of **6e**	486 (3.20)	35 (0.030)	16	209 (1.20) of **7e**	1 drop of Et_2NH	4	545 (78) of **10e**
27	192 (0.60) of **3**	478 (0.72) of **6o**	292 (1.92)	21 (0.018)	16	96 (0.72) of **7b**	46 (0.60) of NH_4OAc	5	470 (88) of **10f**
28	192 (0.60) of **3**	360 (0.72) of **6p**	292 (1.92)	21 (0.018)	16	96 (0.72) of **7b**	46 (0.60) of NH_4OAc	5	350 (80) of **10g**
33	300 (0.94) of **3**	563 (1.13) of **6p**	457 (3.01)	33 (0.028)	16	197 (1.13) of **7e**	1 drop of Et_2NH	4	431 (60) of **10h**

2.1.3.1. (*Z*)-5-methyl-4-((4-(octyloxy)-5-(*p*-tolyl)thiophen-2-yl)methylene)-2-phenyl-2,4-dihydro-3*H*-pyrazol-3-one (10a)

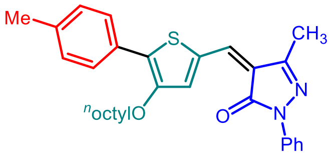

$C_{30}H_{34}N_2O_2S$ [486.67]

According to the GP and after purification by chromatography on silica gel (*n*-hexane/acetone 20:1) and drying under vacuo compound **10a** (268 mg, 69%) was obtained as a red brown amorphous solid, Mp 102–104 °C. R_f (*n*-hexane/acetone 10:1) = 0.19.

^{1}H NMR (300 MHz, acetone-d$_6$/CS$_2$ 4:1): δ 0.87–0.98 (m, 3 H), 1.30–1.47 (m, 8 H), 1.55 (quin, ^3J = 7.1 Hz, 2 H), 1.87 (quin, ^3J = 6.4 Hz, 2 H), 2.33 (s, 3 H), 2.40 (s, 3 H), 4.21 (t, ^3J = 6.4 Hz, 2 H), 7.13 (t, ^3J = 7.4 Hz, 1 H), 7.24 (d, ^3J = 8.1 Hz, 2 H), 7.34–7.42 (m, 2 H), 7.64 (s, 1 H), 7.78–7.85 (m, 2 H), 8.03–8.09 (m, 2 H), 8.28 (s, 1 H). ^{13}C NMR (75 MHz, acetone-d$_6$/CS$_2$ 4:1): δ 13.3 (CH_3), 14.7 (CH_3), 21.7 (CH_3), 23.7 (CH_2), 27.0 (CH_2), 30.2 (CH_2), 30.3 (CH_2), 30.4 (CH_2), 32.8 (CH_2), 72.4 (CH_2), 118.8 (CH), 122.6 (C_{quat}), 124.7 (CH), 128.3 (CH), 129.3 (CH), 129.5 (CH), 130.2 (CH), 130.8 (C_{quat}), 132.6 (C_{quat}), 135.3 (C_{quat}), 136.5 (CH), 139.0 (C_{quat}), 139.9 (C_{quat}), 150.5 (C_{quat}), 155.2 (C_{quat}), 162.9 (C_{quat}). MS (MALDI-TOF) calcd for $C_{30}H_{34}N_2O_2S$ *m/z:* 486.23; Found: 487.0 ([MH]$^+$). IR: $\tilde{\nu}$ [cm^{-1}] = 2988 (w), 2953 (w), 2924 (w), 2855 (w), 1672 (m), 1595 (m), 1559 (w), 1535 (w), 1497 (m), 1429 (w), 1412 (m), 1383 (w), 1366 (w), 1306 (s), 1271 (w), 1242 (m), 1213 (w), 1188 (w), 1142 (m), 1130 (w), 1109 (w), 1080 (m), 1061 (w), 1026 (w), 1001 (w), 978 (w), 943 (w), 909 (w), 889 (w), 810 (m), 791 (w), 766 (w), 748 (s), 716 (w), 689 (m), 662 (m). Anal calcd for $C_{30}H_{34}N_2O_2S$ [486.7]: C 74.04, H 7.04, N 5.76, S 6.59; Found: C 74.17, H 7.11, N 6.01, S 6.25.

2.1.3.2. (*E*)-2-(3-cyano-5,5-dimethyl-4-(2-(4-(octyloxy)-5-(*p*-tolyl)thiophen-2-yl)vinyl)furan-2(5*H*)-ylidene)malononitrile (10b)

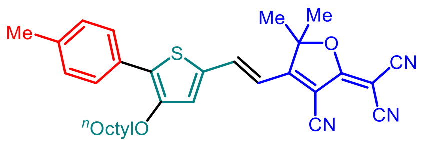

$C_{31}H_{33}N_3O_2S$ [511.23]

According to the GP and after purification by chromatography on silica gel (*n*-hexane/acetone 20:1) and drying under vacuo compound **10b** (169 mg, 55%) was obtained as a black amorphous solid, Mp 164–167 °C. R_f (*n*-hexane/acetone 3:1) = 0.34.

^{1}H NMR (300 MHz, acetone-d$_6$/CS$_2$ 4:1): δ 0.91 (t, ^3J = 6.7 Hz, 3 H), 1.25–1.45 (m, 8 H), 1.52 (quin, ^3J = 7.5 Hz, 2 H), 1.87 (m, 8 H), 2.38 (s, 3 H), 4.20 (t, ^3J = 6.6 Hz, 2 H), 6.94 (d, ^3J = 15.7 Hz, 1 H), 7.24 (d, ^3J = 7.6 Hz, 2 H), 7.70–7.80 (m, 3 H), 8.05 (d, ^3J = 15.8 Hz, 1 H). ^{13}C NMR (75 MHz, acetone-d$_6$/CS$_2$ 4:1): δ 14.6 (CH_3), 21.5 (CH_3), 23.5 (CH_2), 26.2 (CH_3), 26.9 (CH_2), 30.1 (CH_2),[2] 30.2 (CH_2), 32.7 (CH_2), 72.7 (CH_2), 98.8 (C_{quat}), 98.8 (C_{quat}),[3] 111.4 (C_{quat}), 112.2 (C_{quat}), 112.9 (C_{quat}), 113.5 (CH), 125.0 (CH), 128.0 (CH), 130.3 (CH), 130.5 (C_{quat}), 130.7 (C_{quat}), 135.6 (C_{quat}), 139.1 (C_{quat}), 140.2 (CH), 155.7 (C_{quat}), 174.5 (C_{quat}), 176.9 (C_{quat}). MS

[2] Two CH_2 signals coincide.

[3] Two C_{quat} signals coincide.

(MALDI-TOF) calcd for $C_{31}H_{33}N_3O_2S$ *m/z:* 511.23; Found: 511.2 ($[M]^+$). IR: $\tilde{\nu}$ [cm^{-1}] = 3672 (w), 2987 (w), 2922 (w), 2866 (w), 2228 (m), 1599 (w), 1565 (s), 1559 (s), 1545 (s), 1522 (w), 1470 (w), 1439 (m), 1410 (w), 1395 (w), 1360 (s), 1306 (w), 1275 (m), 1256 (s), 1213 (w), 1188 (w), 1171 (m), 1105 (m), 1078 (m), 976 (w), 955 (m), 941 (w), 858 (w), 841 (w), 817 (m), 750 (w), 714 (w), 652 (w), 625 (w) . Anal calcd for $C_{31}H_{33}N_3O_2S$ [511.2]: C 72.77, H 6.50, N 8.21, S 6.27; Found: C 72.60, H 6.55, N 7.99, S 6.38.

2.1.3.3. (*Z*)-5-((4-(octyloxy)-5-(4-(1,2,2-triphenylvinyl)phenyl)thiophen-2-yl)methylene)-2-thioxothiazolidin-4-one (10c)

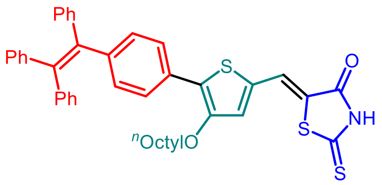

$C_{42}H_{39}NO_2S_3$ [685.96]

According to the GP and after purification by chromatography on silica gel (*n*-hexane/acetone 10:1), precipitation from a *n*-hexane/dichloromethane solution and drying under vacuo compound **10c** (204 mg, 59%) was obtained as orange red crystal platelets, Mp 154–157 °C. R_f (*n*-hexane/ethyl acetate 5:1) = 0.24.

^1H NMR (300 MHz, acetone-d_6/CS_2 4:1): δ 0.87–0.95 (m, 3 H), 1.25–1.56 (m, 10 H), 1.82 (quin, ^3J = 6.5 Hz, 2 H), 4.20 (t, ^3J = 6.4 Hz, 2 H), 7.01–7.17 (m, 17 H), 7.49 (s, 1 H), 7.62–7.67 (m, 2 H), 7.68 (s, 1 H). ^{13}C NMR (75 MHz, acetone-d_6/CS_2 4:1): δ 14.7 (CH_3), 23.6 (CH_2), 26.9 (CH_2), 30.1 (CH_2),[4] 30.3 (CH_2), 32.7 (CH_2), 72.7 (CH_2), 123.7 (C_{quat}), 124.9 (CH), 124.9 (CH), 126.9 (CH), 127.3 (CH), 127.4 (CH), 127.5 (CH), 128.5 (CH), 128.6 (CH), 128.7 (CH), 129.5 (C_{quat}), 131.6 (C_{quat}), 131.96 (CH), 131.98 (CH), 132.1 (CH), 132.4 (CH), 133.7 (C_{quat}), 141.2 (C_{quat}), 142.3 (C_{quat}), 144.0 (C_{quat}), 144.2 (C_{quat}),[5] 144.4 (2 C_{quat}), 155.7 (C_{quat}), 168.9 (C_{quat}), 194.1 (C_{quat}). MS (MALDI-TOF) calcd for $C_{42}H_{39}NO_2S_3$ *m/z:* 685.21; Found: 685.2 ($[M]^+$). IR: $\tilde{\nu}$ [cm^{-1}] = 3138 (w), 3005 (w), 2922 (w), 2847 (w), 1686 (m), 1649 (w), 1572 (s), 1545 (m), 1530 (m), 1491 (w), 1431 (m), 1406 (m), 1379 (w), 1296 (m), 1263 (m), 1213 (s), 1167 (m), 1153 (m), 1126 (w), 1078 (m), 1063 (m), 1014 (w), 977 (w), 965 (w), 941 (w), 891 (w), 850 (w), 826 (m), 751 (m), 696 (s), 665 (s), 652 (m), 619 (w). Anal calcd for $C_{42}H_{39}NO_2S_3$ [686.0]: C 73.54, H 5.73, N 2.04, S 14.02; Found: C 73.62, H 5.89, N 1.92, S 13.98.

[4] Two CH_2 signals coincide.
[5] Two C_{quat} signals coincide.

2.1.3.4. 5-{[5-(4-{Bis[4-methoxyphenyl]amino}phenyl)-3-(octyloxy)thiophen-2-yl]methylene}-2-thioxothiazolidin-4-one (10d)

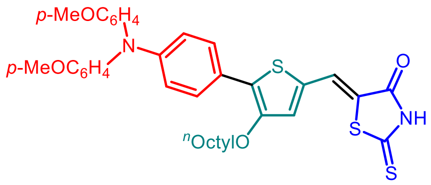

$C_{36}H_{38}N_2O_4S_3$ [658.89]

According to the GP and after purification by chromatography on silica gel (*n*-hexane/acetone 15:1) and drying under vacuo compound **10d** (355 mg, 90%) was obtained as a dark red amorphous solid, Mp 92–94 °C. R_f (*n*-hexane/acetone 1:1) = 0.74.

^{1}H NMR (300 MHz, acetone-d$_6$/CS$_2$ 4:1): δ 0.84–0.97 (m, 3 H), 1.22–1.47 (m, 8 H), 1.51 (quin, ^3J = 7.1 Hz, 2 H), 1.84 (quin, ^3J = 6.5 Hz, 2 H), 3.80 (s, 6 H), 4.19 (t, ^3J = 6.4 Hz, 2 H), 6.83–6.93 (m, 6 H), 7.04–7.12 (m, 4 H), 7.44 (s, 1 H), 7.65 (s, 1 H), 7.66–7.71 (m, 2 H), 12.06 (br, 1 H). ^{13}C NMR (75 MHz, acetone-d$_6$/CS$_2$ 4:1): δ 14.7 (CH_3), 23.6 (CH_2), 27.0 (CH_2), 30.2 (CH_2),[6] 30.4 (CH_2), 32.7 (CH_2), 55.7 (CH_3), 72.6 (CH_2), 115.6 (CH), 119.9 (CH), 122.4 (C_{quat}), 124.8 (C_{quat}), 125.08 (CH), 125.11 (CH), 127.9 (CH), 128.6 (CH), 131.2 (C_{quat}), 132.0 (C_{quat}), 140.7 (C_{quat}), 149.3 (C_{quat}), 154.6 (C_{quat}), 157.4 (C_{quat}), 168.8 (C_{quat}), 194.0 (C_{quat}). MS (MALDI-TOF) calcd for $C_{36}H_{38}N_2O_4S_3$ *m/z:* 658.20; Found: 658.1 ([M]$^+$). IR: IR: $\tilde{\nu}$ [cm^{-1}] = 3671 (w), 2988 (w), 2955 (w), 2922 (w), 2901 (w), 2853 (w), 1697 (w), 1574 (m), 1533 (w), 1501 (s), 1462 (w), 1433 (m), 1416 (m), 1412 (m), 1400 (m), 1381 (m), 1319 (w), 1289 (m), 1263 (m), 1238 (s), 1207 (s), 1196 (s), 1161 (s), 1126 (m), 1105 (m), 1076 (m), 1057 (m), 1036 (s), 1013 (m), 974 (w), 947 (w), 891 (w), 822 (s), 779 (m), 764 (w), 729 (w), 710 (m), 675 (m), 648 (m), 617 (w). Anal calcd for $C_{36}H_{38}N_2O_4S_3$ [658.9]: C 65.62, H 5.81, N 4.25, S 14.60; Found: C 65.63, H 5.91, N 4.11, S 14.30.

2.1.3.5. (*Z*)-4-((5-(4-(bis(4-methoxyphenyl)amino)phenyl)-4-(octyloxy)thiophen-2-yl)methylene)-5-methyl-2-phenyl-2,4-dihydro-3*H*-pyrazol-3-one (10e)

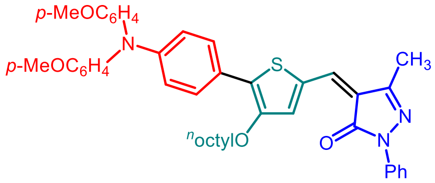

$C_{43}H_{45}N_3O_4S$ [699.90]

[6] Two CH_2 signals coincide.

According to the GP and after purification by chromatography on silica gel (*n*-hexane/acetone 30:1) and drying under vacuo compound **10e** (545 mg, 78%) was obtained as a dark violet amorphous solid, Mp softening >60 °C, melting >90 °C. R_f (*n*-hexane/acetone 10:1) = 0.15.

^{1}H NMR (300 MHz, acetone-d$_6$/CS$_2$ 4:1): δ 0.83–0.94 (m, 3 H), 1.24–1.46 (m, 8 H), 1.51 (quin, ^3J =7.2 Hz, 2 H), 1.84 (quin, ^3J = 6.5 Hz, 2 H), 2.30 (s, 3 H), 3.80 (s, 6 H), 4.19 (t, ^3J = 6.4 Hz, 2 H), 6.83–6.95 (m, 6 H), 7.07–7.16 (m, 5 H), 7.33–7.42 (m, 2 H), 7.60–7.63 (s, 1 H), 7.72–7.80 (m, 2 H), 8.03–8.09 (m, 2 H), 8.24 (s, 1 H). ^{13}C NMR (75 MHz, acetone-d$_6$/CS$_2$ 4:1): δ 13.3 (CH$_3$), 14.6 (CH$_3$), 23.5 (CH$_2$), 27.0 (CH$_2$), 30.1 (CH$_2$), 30.2 (CH$_2$), 30.3 (CH$_2$), 32.7 (CH$_2$), 55.8 (CH$_3$), 72.4 (CH$_2$), 115.7 (CH), 118.8 (CH), 119.5 (CH), 121.5 (C$_{quat}$), 124.6 (CH), 124.9 (C$_{quat}$), 128.2 (CH), 129.2 (CH), 129.3 (CH), 129.7 (CH), 131.5 (C$_{quat}$), 136.5 (CH), 136.7 (C$_{quat}$), 140.1 (C$_{quat}$), 140.6 (C$_{quat}$), 149.9 (C$_{quat}$), 150.6 (C$_{quat}$), 154.8 (C$_{quat}$), 157.6 (C$_{quat}$), 163.1 (C$_{quat}$). MS (MALDI-TOF) calcd for $C_{43}H_{45}N_3O_4S$ *m/z:* 699.31; Found: 699.3 ([M]$^+$). IR: $\tilde{\nu}$ [cm^{-1}] = 2924 (w), 2833 (w), 1670 (w), 1580 (m), 1557 (w), 1530 (w), 1499 (s), 1464 (w), 1433 (w), 1406 (m), 1364 (m), 1317 (m), 1302 (m), 1275 (m), 1238 (s), 1194 (m), 1179 (m), 1165 (w), 1123 (m), 1109 (w), 1078 (w), 1029 (m), 1026 (m), 997 (w), 976 (w), 951 (w), 909 (w), 880 (w), 826 (m), 793 (w), 779 (w), 752 (m), 721 (w), 704 (w), 691 (m), 660 (m), 633 (w). Anal calcd for $C_{43}H_{45}N_3O_4S$ [699.9]: C 73.79, H 6.48, N 6.00, S 4.58; Found: C 74.03, H 6.66, N 6.03, S 4.70.

2.1.3.6. (*Z*)-5-((5-(10-(2-decyltetradecyl)-10*H*-phenothiazin-3-yl)-4-(octyloxy)thiophen-2-yl)methylene)-2-thioxothiazolidin-4-one (10f)

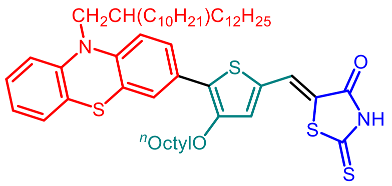

$C_{52}H_{76}N_2O_2S_4$ [889.43]

According to the GP and after purification by chromatography on silica gel (*n*-hexane/acetone 20:1) and drying under vacuo compound **10f** (470 mg, 88%) was obtained as a dark red resin. R_f (*n*-hexane/acetone 10:1) = 0.14.

^{1}H NMR (300 MHz, acetone-d$_6$/CS$_2$ 4:1): δ 0.87–0.98 (m, 9 H), 1.20–1.65 (m, 50 H), 1.81–1.97 (m, 2 H), 1.97–2.05 (m, 1 H), 3.85 (d, ^3J = 7.0 Hz, 2 H), 4.23 (t, ^3J = 6.3 Hz, 2 H), 6.91–7.02 (m, 3 H), 7.13 (dd, ^3J = 7.7 Hz, ^4J = 1.5 Hz, 1 H), 7.12–7.24 (m, 1 H), 7.47 (s, 1 H), 7.64 (dd, ^3J = 8.6 Hz, ^4J = 2.2 Hz, 1 H), 7.67 (s, 1 H), 7.70 (d, ^4J = 2.2 Hz, 1 H), 12.10 (br, 1 H). ^{13}C NMR (75 MHz, acetone-d$_6$/CS$_2$ 4:1): δ 14.76 (CH$_3$),[7] 14.78 (CH$_3$), 23.7 (CH$_2$),[8] 27.1 (CH$_2$), 27.16 (CH$_2$), 27.18 (CH$_2$), 30.29 (CH$_2$), 30.32 (CH$_2$), 30.4 (CH$_2$),[8] 30.45 (CH$_2$), 30.48 (CH$_2$),[8] 30.6

[7] Two CH_3 signals coincide.
[8] Two CH_2 signals coincide.

(CH_2),[9] 30.65 (CH_2),[8] 30.66 (CH_2), 30.69 (CH_2), 31.0 (CH_2),[8] 32.4 (CH_2),[8] 32.8 (CH_2), 32.9 (CH_2),[8] 35.5 (CH), 52.2 (CH_2), 72.7 (CH_2), 116.9 (CH), 117.1 (CH), 123.2 (C_{quat}), 123.6 (CH), 124.9 (CH),[10] 125.8 (C_{quat}), 126.3 (CH), 126.77 (C_{quat}), 126.81 (CH), 127.8 (C_{quat}), 128.2 (CH),[10] 129.4 (C_{quat}), 132.9 (C_{quat}), 145.9 (C_{quat}), 146.0 (C_{quat}), 155.0 (C_{quat}), 168.8 (C_{quat}), 193.9 (C_{quat}). MS (MALDI-TOF) calcd for $C_{52}H_{76}N_2O_2S_4$ *m/z:* 888.48; Found: 888.5 ($[M]^+$). IR: $\tilde{\nu}$ [cm^{-1}] = 3150 (w), 3090 (w), 3071 (w), 2953 (w), 2920 (m), 2851 (m), 1699 (m), 1581 (m), 1572 (m), 1533 (w), 1495 (w), 1464 (m), 1427 (s), 1395 (w), 1377 (w), 1331 (w), 1294 (m), 1277 (m), 1250 (m), 1213 (s), 1165 (m), 1132 (w), 1107 (w), 1084 (w), 1063 (w), 1037 (w), 1003 (w), 949 (w), 926 (w), 889 (w), 870 (w), 816 (w), 791 (w), 768 (w), 745 (m), 729 (w), 673 (m), 656 (w), 611 (w). Anal calcd for $C_{52}H_{76}N_2O_2S_4$ [889.4]: C 70.22, H 8.61, N 3.15, S 14.42; Found: C 70.05, H 8.38, N 3.09, S 14.33.

2.1.3.7. (*Z*)-5-((5-(10-hexyl-7-(*p*-tolyl)-10*H*-phenothiazin-3-yl)-4-(octyloxy)thiophen-2-yl)methylene)-2-thioxothiazolidin-4-one (10g)

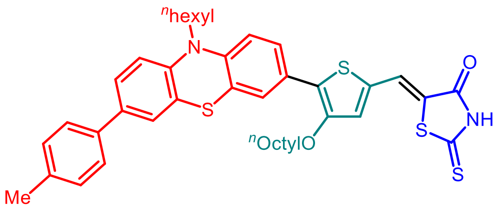

$C_{41}H_{46}N_2O_2S_4$ [727.08]

According to the GP and after purification by chromatography on silica gel (*n*-hexane/acetone 15:1), precipitation from a *n*-hexane/acetone solution, and drying under vacuo compound **10g** (350 mg, 80%) was obtained as a black amorphous solid, Mp 121–123 °C. R_f (*n*-hexane/acetone 1:1) = 0.77.

^1H NMR (300 MHz, acetone-d_6/CS_2 4:1): δ 0.85–0.95 (m, 6 H), 1.27–1.57 (m, 16 H), 1.70–1.88 (m, 4 H), 2.36 (s, 3 H), 3.88 (t, ^3J = 7.0 Hz, 2 H), 4.13 (t, ^3J = 6.3 Hz, 2 H), 6.90 (d, ^3J = 8.5 Hz, 1 H), 6.96 (d, ^3J = 8.6 Hz, 1 H), 7.19 (d, ^3J = 8.0 Hz, 2 H), 7.26 (d, ^4J = 2.1 Hz, 1 H), 7.37 (dd, ^3J = 8.5 Hz, ^4J = 2.2 Hz, 1 H), 7.39–7.44 (m, 2 H), 7.50–7.58 (m, 3 H), 7.71 (s, 1 H), 13.63 (br, 1 H). ^{13}C NMR (75 MHz, acetone-d_6/CS_2 4:1): δ 14.7 (CH_3), 14.8 (CH_3), 21.5 (CH_3), 23.29 (CH_2), 23.31 (CH_2), 26.7 (CH_2), 27.0 (CH_2), 27.1 (CH_2), 29.8 (CH_2), 29.9 (CH_2), 30.0 (CH_2), 32.0 (CH_2), 32.3 (CH_2), 47.6 (CH_2), 72.1 (CH_2), 115.8 (CH), 116.3 (CH), 122.9 (C_{quat}), 124.2 (C_{quat}), 124.4 (C_{quat}), 124.7 (CH), 125.3 (CH), 125.4 (CH), 125.4 (CH), 126.1 (CH), 126.4 (CH), 126.5 (CH), 127.1 (C_{quat}), 128.4 (C_{quat}), 130.0 (CH), 132.5 (C_{quat}), 135.4 (C_{quat}), 136.75 (C_{quat}), 136.81 (C_{quat}), 143.4 (C_{quat}), 144.3 (C_{quat}), 154.4 (C_{quat}), 169.4 (C_{quat}), 194.2 (C_{quat}). MS (MALDI-TOF) calcd for $C_{41}H_{46}N_2O_2S_4$ *m/z:* 726.24; Found: 726.2 ($[M]^+$). IR: $\tilde{\nu}$ [cm^{-1}] = 3078 (w), 2951

[9] Three CH_2 signals coincide.
[10] Two CH signals coincide.

(w), 2868 (w), 2835 (w), 1692 (m), 1570 (m), 1532 (m), 1474 (m), 1422 (m), 1389 (m), 1364 (m), 1298 (w), 1265 (w), 1245 (w), 1206 (s), 1163 (m), 1100 (w), 1078 (m), 1061 (m), 1005 (m), 982 (w), 947 (w), 885 (w), 820 (w), 795 (m), 745 (w), 675 (s), 648 (m). Anal calcd for $C_{41}H_{46}N_2O_2S_4$ [727.1]: C 67.73, H 6.38, N 3.85, S 17.64; Found: C 67.44, H 6.28, N 3.86, S 17.53.

2.1.3.8. (*Z*)-4-((5-(10-hexyl-7-(*p*-tolyl)-10*H*-phenothiazin-3-yl)-4-(octyloxy)thiophen-2-yl)methylene)-5-methyl-2-phenyl-2,4-dihydro-3*H*-pyrazol-3-one (10h)

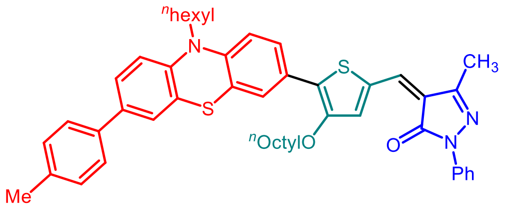

$C_{48}H_{53}N_3O_2S_2$ [768.08]

According to the GP and after purification by chromatography on silica gel (*n*-hexane/acetone 30:1) and drying under vacuo compound **10h** (431 mg, 60%) was obtained as a dark violet amorphous solid, Mp softening >60 °C, melting >95 °C. R_f (*n*-hexane/acetone 10:1) = 0.23. ^{1}H NMR (300 MHz, acetone-d$_6$/CS_2 4:1): δ 0.87–0.97 (m, 6 H), 1.30–1.60 (m, 16 H), 1.79–1.97 (m, 4 H), 2.30 (s, 3 H), 2.37 (s, 3 H), 3.96 (t, ^3J = 7.1 Hz, 2 H), 4.21 (t, ^3J = 6.3 Hz, 2 H), 7.00 (t, ^3J = 8.9 Hz, 2 H), 7.09–7.18 (m, 1 H), 7.22 (d, ^3J = 8.0 Hz, 2 H), 7.32–7.50 (m, 6 H), 7.63 (s, 1 H), 7.70 (dd, ^3J = 8.6 Hz, ^4J = 2.2 Hz, 1 H), 7.76 (d, ^4J = 2.1 Hz, 1 H), 8.04–8.11 (m, 2 H), 8.21 (s, 1 H). ^{13}C NMR (75 MHz, acetone-d$_6$/CS_2 4:1): δ 13.3 (CH_3), 14.6 (CH_3), 14.7 (CH_3), 21.4 (CH_3), 23.6 (CH_2), 23.7 (CH_2), 27.2 (CH_2), 27.4 (CH_2), 27.6 (CH_2), 30.30 (CH_2), 30.34 (CH_2), 30.40 (CH_2), 30.44 (CH_2), 32.8 (CH_2), 48.2 (CH_2), 72.5 (CH_2), 116.2 (CH), 116.7 (CH), 118.8 (CH),[11] 122.2 (C_{quat}), 124.7 (CH), 124.97 (CH), 125.04 (C_{quat}), 126.0 (C_{quat}), 126.6 (CH), 127.0 (CH), 127.5 (CH), 127.9 (C_{quat}), 129.3 (CH), 129.6 (CH), 130.3 (CH), 132.2 (C_{quat}), 134.5 (C_{quat}), 136.35 (C_{quat}), 136.40 (CH), 137.4 (C_{quat}), 137.6 (C_{quat}), 140.0 (C_{quat}), 144.0 (C_{quat}), 145.6 (C_{quat}), 150.6 (C_{quat}), 155.1 (C_{quat}), 163.0 (C_{quat}). MS (MALDI-TOF) calcd for $C_{48}H_{53}N_3O_2S_2$ *m/z:* 767.36; Found: 767.3 ($[M]^+$). IR: $\tilde{\nu}$ [cm^{-1}] = 3026 (w), 2951 (w), 2922 (w), 2853 (w), 1672 (w), 1593 (m), 1578 (m), 1557 (w), 1532 (w), 1497 (m), 1472 (m), 1416 (s), 1396 (m), 1362 (s), 1319 (m), 1306 (s), 1277 (s), 1262 (m), 1238 (m), 1173 (w), 1136 (m), 1121 (m), 1111 (m), 1086 (w), 1026 (w), 997 (s), 949 (w), 907 (w), 880 (w), 802 (s), 754 (m), 752 (m), 712 (w), 691 (m), 660 (m). Anal calcd for $C_{48}H_{53}N_3O_2S_2$ [768.1]: C 75.06, H 6.96, N 5.47, S 8.35; Found: C 74.99, H 6.83, N 5.65, S 8.08.

[11] Two CH signals coincide.

2.1.4. Consecutive Three-component Suzuki-Knoevenagel Synthesis of Carbazole-bridged Systems 11

Table S4. Experimental details of the consecutive three-component Suzuki-Knoevenagel synthesis of carbazole-bridged systems **11**.

**Entry**	**Bromo-aldehyde 4** [mg] (mmol)	**Boronic acid/ester 6** [mg] (mmol)	**CsX** [mg] (mmol)	**Pd(PPh₃)₄** [mg] (µmol)	***t₁*** [h]	**methylene active compound 7** [mg] (mmol)	**Organo catalyst** [mg] (mmol)	***t₂*** [h]	**Product** [mg] (%)
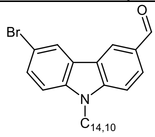	**4**								
1	300 (0.492) of **4**	81 (0.59) of **6b**	239 (1.57) of CsF	17 (15)	16	80 (0.54) of **7a**	38 (0.49) of NH_4OAc	6	332 (90) of **11a**
2	325 (0.532) of **4**	87 (0.64) of **6b**	259 (1.70) of CsF	18 (16)	16	78 (0.585) of **7b**	41 (0.53) of NH_4OAc	5	337 (86) of **11b**
3	302 (0.495) of **4**	81 (0.59) of **6b**	516 (0.79) of Cs_2CO_3	17 (15)	16	87 (0.59) of **7c**	1 drop of Et_2NH	4	293 (59) of **11c**
4	318 (0.521) of **4**	85 (0.63) of **6b**	253 (1.67) of CsF	18 (16)	16	109 (0.63) of **7e**	1 drop of Et_2NH	5	320 (79) of **11d**
5	330 (0.54) of **4**	88 (0.648) of **6b**	262 (1.73) of CsF	19 (16)	16	101 (0.62) of **7f**	1 drop of Et_2NH	8	287 (73) of **11e**
6	330 (0.540) of **4**	88 (0.648) of **6b**	262 (1.73) of CsF	19 (16)	16	140 (0.70) of **7g**	42 (0.54) of NH_4OAc	6	395 (91) of **11f**
7	300 (0.492) of **4**	123 (0.59) of **6l**	512 (0.77) of Cs_2CO_3	17 (15)	16	86 (0.59) of **7c**	1 drop of Et_2NH	4	292 (60) of **11g**
8	315 (0.516) of **4**	267 (0.62) of **6e**	538 (1.65) of Cs_2CO_3	18 (16)	16	108 (0.63) of **7e**	1 drop of Et_2NH	5	289 (57) of **11h**
9	315 (0.516) of **4**	309 (0.62) of **6p**	538 (1.65) of Cs_2CO_3	18 (16)	16	109 (0.63) of **7e**	1 drop of Et_2NH	5	334 (61) of **11i**

2.1.4.1. 5-{[9-(2-Decyltetradecyl)-6-(*p*-tolyl)-9*H*-carbazol-3-yl]methylene}-3-methyl-2-thioxothiazolidin-4-one (11a)

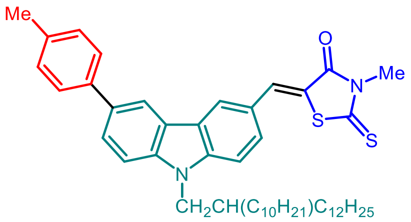

$C_{48}H_{66}N_2OS_2$ [751.18]

According to the GP and after purification by chromatography on silica gel (*n*-hexane/acetone 40:1) and drying under vacuo compound **11a** (332 mg, 90%) was obtained as a viscous orange red oil. R_f (*n*-hexane/acetone 10:1) = 0.43.

^{1}H NMR (300 MHz, acetone-d$_6$/CS$_2$ 4:1): δ 0.86 (t, ^3J = 6.7 Hz, 3 H), 0.87 (t, ^3J = 6.7 Hz, 3 H), 1.14–1.45 (m, 40 H), 2.09–2.21 (m, 1 H), 2.40 (s, 3 H), 3.44 (s, 3 H), 4.21 (d, ^3J = 7.5 Hz, 2 H), 7.26–7.33 (m, 2 H), 7.52–7.60 (m, 3 H), 7.64–7.70 (m, 2 H), 7.77 (dd, ^3J = 8.5 Hz, ^4J = 1.8 Hz, 1 H), 7.83 (s, 1 H), 8.32 (d, ^4J = 1.3 Hz, 1 H), 8.46 (d, ^4J = 1.7 Hz, 1 H). ^{13}C NMR (75 MHz, acetone-d$_6$/CS$_2$ 4:1): δ 14.5 (CH_3),[12] 21.2 (CH_3), 23.5 (CH_2),[13] 27.26 (CH_2), 27.29 (CH_2), 30.18 (CH_2), 30.20 (CH_2), 30.23 (CH_2), 30.3 (CH_2), 30.39 (CH_2), 30.42 (CH_2), 30.44 (CH_2), 30.47 (CH_2), 30.49 (CH_3), 30.51 (CH_2), 30.65 (CH_2), 30.69 (CH_2), 31.5 (CH_2), 32.55 (CH_2), 32.58 (CH_2), 32.7 (2 CH_2), 38.6 (CH), 48.5 (CH_2), 111.1 (CH), 111.2 (CH), 119.4 (C_{quat}), 119.5 (CH), 124.0 (C_{quat}), 124.7 (C_{quat}), 125.0 (CH), 125.2 (C_{quat}), 126.6 (CH), 127.7 (CH), 129.6 (CH), 130.4 (CH), 134.1 (C_{quat}), 135.3 (CH), 137.0 (C_{quat}), 139.4 (C_{quat}), 141.7 (C_{quat}), 143.3 (C_{quat}), 168.1 (C_{quat}), 194.4 (C_{quat}). MS (MALDI-TOF) calcd for $C_{48}H_{66}N_2OS_2$-H^+ *m/z:* 750.5; Found: 750.5. IR: $\tilde{\nu}$ [cm^{-1}] = 2920 (m), 2851 (w), 1705 (m), 1578 (m), 1479 (m), 1464 (w), 1424 (w), 1389 (w), 1350 (w), 1287 (s), 1260 (m), 1233 (w), 1207 (w), 1157 (w), 1125 (s), 1099 (s), 1065 (w), 990 (w), 955 (w), 899 (w), 887 (w), 824 (w), 799 (s), 729 (m), 718 (w), 687 (w), 631 (w). Anal calcd for $C_{48}H_{66}N_2OS_2$ [751.2]: C 76.75, H 8.86, N 3.73, S 8.54; Found: C 76.53, H 8.79, N 3.69, S 8.69.

2.1.4.2. 5-{[9-(2-Decyltetradecyl)-6-(*p*-tolyl)-9H-carbazol-3-yl]methylene}-2-thioxothiazolidin-4-one (11b)

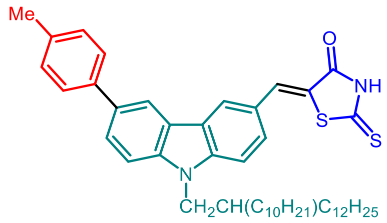

$C_{47}H_{64}N_2OS_2$ [737.15]

According to the GP and after purification by chromatography on silica gel (*n*-hexane/acetone 30:1) and drying under vacuo compound **11b** (337 mg, 86%) was obtained as a yellow orange amorphous solid, Mp 110–115 °C. R$_f$ (*n*-hexane/acetone 5:1) = 0.51.

^{1}H NMR (300 MHz, acetone-d$_6$/CS$_2$ 4:1): δ 0.90 (t, ^3J = 6.7 Hz, 3 H), 0.94 (t, ^3J = 6.7 Hz, 3 H), 1.21–1.47 (m, 40 H), 2.19 (hep, ^3J = 6.9 Hz, 1 H), 2.42 (s, 3 H), 4.32 (d, ^3J = 7.5 Hz, 2 H), 7.25–7.31 (m, 2 H), 7.55–7.69 (m, 5 H), 7.77 (dd, ^3J = 8.6 Hz, ^4J = 1.8 Hz, 1 H), 7.79 (s, 1 H), 8.39 (d, ^4J = 1.6 Hz, 1 H), 8.46 (d, ^4J = 1.7 Hz, 1 H), 12.08 (s, 1 H). ^{13}C NMR (75 MHz, acetone-d$_6$/CS$_2$ 4:1): δ 14.7 (CH_3),[14] 21.4 (CH_3), 23.6 (CH_2),[15] 27.5 (CH_2),[15] 30.31 (CH_2), 30.33 (CH_2), 30.5 (CH_2),[15] 30.59 (CH_2),[16] 30.61 (CH_2),[15] 30.64 (CH_2), 30.87 (CH_2), 30.88 (CH_2), 32.7 (CH_2),[15]

---

[12] Two CH_3 signals coincide.
[13] Two CH_2 signals coincide.
[14] Two CH_3 signals coincide.
[15] Two CH_2 signals coincide.
[16] Three CH_2 signals coincide.

32.9 (CH_2),[15] 38.8 (CH), 48.6 (CH_2), 111.0 (CH), 111.2 (CH), 119.7 (CH), 122.4 (C_{quat}), 124.0 (C_{quat}), 124.7 (C_{quat}), 124.8 (CH), 125.2 (C_{quat}), 126.7 (CH), 127.8 (CH), 129.5 (CH), 130.3 (CH), 134.2 (C_{quat}), 134.4 (CH), 136.8 (C_{quat}), 139.4 (C_{quat}), 141.6 (C_{quat}), 143.2 (C_{quat}), 169.3 (C_{quat}), 195.2 (C_{quat}). MS (MALDI-TOF) calcd for $C_{47}H_{64}N_2OS_2$ *m/z:* 736.45; Found: 736.5 ($[M]^+$). IR: $\tilde{\nu}$ [cm^{-1}] = 3686 (w), 3669 (w), 3138 (w), 2988 (w), 2957 (m), 2918 (s), 2851 (m), 1694 (s), 1576 (s), 1559 (m), 1485 (m), 1451 (m), 1445 (m), 1393 (w), 1344 (w), 1310 (w), 1285 (w), 1258 (w), 1238 (m), 1209 (s), 1171 (m), 1159 (m), 1140 (m), 1098 (w), 1067 (m), 1020 (w), 899 (w), 862 (w), 824 (w), 795 (m), 721 (w), 677 (m), 646 (w). Anal calcd for $C_{47}H_{64}N_2OS_2$ [737.2]: C 76.58, H 8.75, N 3.80, S 8.70; Found: C 76.43, H 8.61, N 3.70, S 8.63.

2.1.4.3. 2-{[9-(2-Decyltetradecyl)-6-(*p*-tolyl)-9*H*-carbazol-3-yl]methylene}-1*H*-inden-1,3[2*H*]-dione (11c)

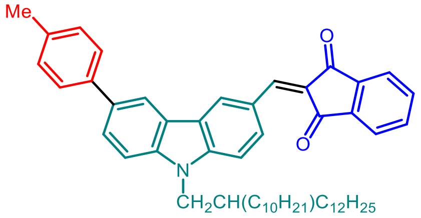

$C_{53}H_{67}NO_2$ [750.12]

According to the GP and after purification by chromatography on silica gel (*n*-hexane/acetone 30:1) and drying under vacuo compound **11c** (220 mg, 57%) was obtained as an orange red amorphous solid, Mp 65–67 °C. R_f (*n*-hexane/acetone 10:1) = 0.31.

^{1}H NMR (600 MHz, acetone-d$_6$/CS_2 4:1): δ 0.87 (t, ^3J = 7.0 Hz, 3 H), 0.88 (t, ^3J = 7.0 Hz, 3 H), 1.18–1.49 (m, 40 H), 2.21 (hep, ^3J = 6.6 Hz, 1 H), 2.42 (s, 3 H), 4.32 (d, ^3J = 7.6 Hz, 2 H), 7.31 (d, ^3J = 7.8 Hz, 2 H), 7.60 (t, ^3J = 9.1 Hz, 2 H), 7.65–7.69 (m, 2 H), 7.77 (dd, ^3J = 8.4 Hz, ^4J = 1.8 Hz, 1 H), 7.86–7.94 (m, 3 H), 7.86–7.94 (m, 2 H), 8.46 (d, ^4J = 1.7 Hz, 1 H), 8.80 (dd, ^3J = 8.7 Hz, ^4J = 1.7 Hz, 1 H), 9.53 (d, ^4J = 1.8 Hz, 1 H). ^{13}C NMR (150 MHz, acetone-d$_6$/CS_2 4:1): δ 14.6 (CH_3),[17] 21.4 (CH_3), 23.6 (CH_2),[18] 27.4 (CH_2), 27.5 (CH_2), 30.27 (CH_2), 30.29 (CH_2), 30.40 (CH_2), 30.41 (CH_2), 30.51 (CH_2), 30.53 (CH_2),[18] 30.58 (CH_2),[18] 30.60 (CH_2), 30.81 (CH_2), 30.84 (CH_2), 32.65 (CH_2), 32.67 (CH_2), 32.8 (CH_2),[18] 38.7 (CH), 48.6 (CH_2), 110.6 (CH), 111.2 (CH), 119.6 (CH), 123.4 (CH), 123.6 (CH), 124.4 (C_{quat}), 124.6 (C_{quat}), 126.0 (C_{quat}), 126.5 (C_{quat}), 126.6 (CH), 127.8 (CH), 129.6 (CH), 130.4 (CH), 133.9 (CH), 134.7 (C_{quat}), 135.6 (CH), 135.8 (CH), 137.0 (C_{quat}), 139.4 (C_{quat}), 140.7 (C_{quat}), 141.7 (C_{quat}), 143.3 (C_{quat}), 145.2 (C_{quat}), 148.5 (CH), 189.7 (C_{quat}), 190.5 (C_{quat}). MS (MALDI-TOF) calcd for $C_{53}H_{67}NO_2$-H^+ *m/z:* 750.52; Found: 750.5. IR: $\tilde{\nu}$ [cm^{-1}] = 2953 (w), 2920 (m), 2851 (m), 1719 (w), 1678 (s), 1595 (w), 1566 (m), 1551 (s), 1487 (m), 1462 (m), 1435 (w), 1383 (m), 1333 (m), 1302 (m), 1256 (w), 1209

[17] Two CH_3 signals coincide.
[18] Two CH_2 signals coincide.

(m), 1180 (m), 1152 (m), 1092 (w), 1067 (w), 1016 (w), 993 (m), 963 (w), 926 (w), 887 (w), 824 (w), 806 (m), 793 (m), 785 (w), 760 (w), 729 (s), 677 (w), 650 (w), 602 (w). Anal calcd for $C_{53}H_{67}NO_2$ [782.2]: C 84.86, H 9.00, N 1.87; Found: C 85.03, H 8.88, N 1.84.

2.1.4.4. 4-{[9-(2-Decyltetradecyl)-6-(*p*-tolyl)-9*H*-carbazol-3-yl]methylene}-3-methyl-1-phenyl-1*H*-pyrazol-5[4*H*]-one (11d)

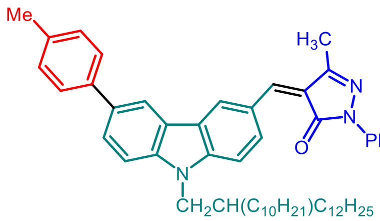

$C_{54}H_{71}N_3O$ [778.16]

According to the GP and after purification by chromatography on silica gel (*n*-hexane/acetone 40:1) and drying under vacuo compound **11d** (329 mg, 81%) was obtained as an orange red amorphous solid, Mp 95–99 °C. R_f (*n*-hexane/acetone 10:1) = 0.41.

^{1}H NMR (300 MHz, acetone-d$_6$/CS_2 4:1): δ 0.89 (t, ^3J = 6.6 Hz, 3 H), 0.90 (t, ^3J = 6.6 Hz, 3 H), 1.20–1.50 (m, 40 H), 2.14–2.27 (m, 1 H), 2.37 (s, 3 H), 2.43 (s, 3 H), 4.30 (d, ^3J = 7.5 Hz, 2 H), 7.15 (tt, ^3J = 7.4 Hz, ^4J = 1.2 Hz, 1 H), 7.30 (d, ^3J = 8.0, 2 H), 7.37–7.45 (m, 2 H), 7.55 (d, ^3J = 8.7 Hz, 1 H), 7.56 (d, ^3J = 8.7 Hz, 1 H), 7.62–7.67 (m, 2 H), 7.73–7.78 (m, 2 H), 8.09–8.17 (m, 2 H), 8.39 (d, ^4J = 1.7 Hz, 1 H), 8.87 (dd, ^3J = 8.8 Hz, ^4J = 1.7 Hz, 1 H), 9.63 (d, ^4J = 1.7 Hz, 1 H). ^{13}C NMR (75 MHz, acetone-d$_6$/CS_2 4:1): δ 13.7 (CH_3), 14.7 (CH_3),[19] 21.4 (CH_3), 23.6 (CH_2),[20] 27.48 (CH_2),[20] 30.30 (CH_2), 30.01 (CH_2), 30.5 (CH_2),[20] 30.6 (CH_2),[21] 30.61 (CH_2),[20] 30.63 (CH_2), 30.87 (CH_2), 30.88 (CH_2), 32.68 (CH_2),[20] 32.84 (CH_2),[20] 38.8 (CH), 48.6 (CH_2), 110.3 (CH), 111.1 (CH), 119.1 (CH), 119.5 (CH), 124.2 (C_{quat}), 124.4 (C_{quat}), 124.6 (C_{quat}), 124.7 (CH), 126.1 (C_{quat}), 126.6 (CH), 127.8 (CH), 129.2 (CH), 129.3 (CH), 130.4 (CH), 133.8 (CH), 134.6 (C_{quat}), 136.9 (C_{quat}), 139.4 (C_{quat}), 140.1 (C_{quat}), 141.6 (C_{quat}), 144.9 (C_{quat}), 149.0 (CH), 151.7 (C_{quat}), 163.0 (C_{quat}). MS (MALDI-TOF) calcd for $C_{54}H_{71}N_3O$-H^+ *m/z:* 777.56; Found: 778.6. IR: $\tilde{\nu}$ [cm^{-1}] = 2951 (w), 2920 (m), 2851 (m), 1678 (m), 1582 (s), 1557 (m), 1499 (m), 1479 (s), 1464 (m), 1456 (m), 1389 (w), 1358 (w), 1314 (m), 1309 (m), 1261 (w), 1229 (m), 1134 (s), 997 (m), 926 (w), 870 (w), 808 (m), 795 (s), 764 (m), 747 (m), 721 (w), 689 (m), 669 (s). Anal calcd for $C_{54}H_{71}N_3O$ [778.2]: C 83.35, H 9.20, N 5.40; Found: C 83.12, H 9.09, N 5.17.

[19] Two CH_3 signals coincide.
[20] Two CH_2 signals coincide.
[21] Three CH_2 signals coincide.

2.1.4.5. 3-[9-(2-Decyltetradecyl)-6-(*p*-tolyl)-9*H*-carbazol-3-yl]-2-(4-nitrophenyl)acrylonitrile (11e)

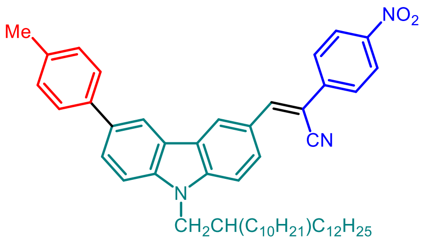

$C_{52}H_{67}N_3O_2$ [766.11]

According to the GP and after purification by chromatography on silica gel (*n*-hexane/acetone 30:1) and drying under vacuo compound **11e** (287 mg, 69%) was obtained as an orange amorphous solid, Mp 87–96 °C. R_f (*n*-hexane/acetone 10:1) = 0.26.

^{1}H NMR (600 MHz, acetone-d$_6$/CS_2 4:1): δ 0.89 (t, ^3J = 7.0 Hz, 3 H), 0.90 (t, ^3J = 7.0 Hz, 3 H), 1.22–1.49 (m, 40 H), 2.21 (hep, ^3J = 6.9 Hz, 1 H), 2.42 (s, 3 H), 4.33 (d, ^3J = 7.5 Hz, 2 H), 7.28 (d, ^3J = 7.8 Hz, 2 H), 7.59 (d, ^3J = 8.5 Hz, 1 H), 7.60–7.64 (m, 3 H), 7.76 (dd, ^3J = 8.5 Hz, ^4J = 1.8 Hz, 1 H), 8.00–8.04 (m, 2 H), 8.21 (s, 1 H), 8.29 (dd, ^3J = 8.7 Hz, ^4J = 1.9 Hz, 1 H), 8.30–8.33 (m, 2 H), 8.34 (d, ^4J = 1.8 Hz, 1 H), 8.84 (d, ^4J = 1.8 Hz, 1 H). ^{13}C NMR (150 MHz, acetone-d$_6$/CS_2 4:1): δ 14.7 (2 CH_3),[22] 21.4 (CH_3), 23.6 (2 CH_2),[23] 27.50 (CH_2), 27.51 (CH_2), 30.31 (CH_2), 30.33 (CH_2), 30.5 (2 CH_2),[23] 30.58 (3 CH_2),[24] 30.61 (CH_2), 30.62 (CH_2), 30.7 (CH_2), 30.91 (CH_2), 39.92 (CH_2), 32.7 (2 CH_2),[23] 32.9 (2 CH_2),[23] 38.8 (CH), 48.6 (CH_2), 105.3 (C_{quat}), 110.8 (CH), 111.0 (CH), 118.8 (C_{quat}), 119.4 (CH), 124.1 (C_{quat}), 124.2 (C_{quat}), 124.7 (CH), 125.0 (CH), 125.4 (C_{quat}), 126.7 (CH), 127.1 (CH), 127.8 (CH), 128.3 (CH), 130.4 (CH), 134.3 (C_{quat}), 136.9 (C_{quat}), 139.4 (C_{quat}), 141.6 (C_{quat}), 142.4 (C_{quat}), 143.8 (C_{quat}), 147.3 (CH), 148.0 (C_{quat}). MS (MALDI-TOF) calcd for $C_{52}H_{67}N_3O_2$ *m/z:* 765.52; Found: 765.5. IR: $\tilde{\nu}$ [cm^{-1}] = 2980 (w), 2953 (w), 2920 (m), 2851 (w), 2210 (w), 1680 (w), 1632 (w), 1599 (w), 1576 (m), 1560 (m), 1514 (m), 1493 (m), 1478 (m), 1458 (w), 1437 (w), 1393 (m), 1377 (w), 1337 (s), 1263 (w), 1227 (m), 1196 (w), 1163 (m), 1148 (m), 1109 (m), 1067 (w), 997 (w), 932 (w), 891 (w), 845 (m), 822 (w), 810 (m), 795 (s), 750 (m), 720 (w), 689 (m), 635 (w), 617 (w). Anal calcd for $C_{52}H_{67}N_3O_2$ [766.1]: C 81.52, H 8.81, N 5.48; Found: C 81.46, H 8.66, N 5.43.

---

[22] Two CH_3 signals coincide.
[23] Two CH_2 signals coincide.
[24] Three CH_2 signals coincide.

2.1.4.6. (*E*)-2-{3-Cyano-4-[2-(9-{2-decyltetradecyl}-6-{*p*-tolyl}-9*H*-carbazol-3-yl)vinyl]-5,5-dimethylfuran-2[5*H*]-yliden}malonitrile (11f)

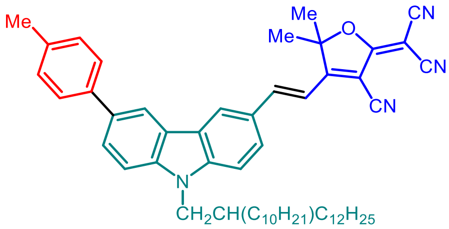

$C_{55}H_{70}N_4O$ [803.17]

According to the GP and after purification by chromatography on silica gel (*n*-hexane/acetone 30:1) and drying under vacuo compound **11f** (355 mg, 82%) was obtained as a dark red amorphous solid, Mp 156–161 °C. R_f (*n*-hexane/acetone 10:1) = 0.23.

^{1}H NMR (300 MHz, acetone-d$_6$/CS$_2$ 4:1): δ 0.82–0.93 (m, 6 H), 1.16–1.49 (m, 40 H), 1.93 (s, 6 H), 2.14–2.26 (m, 1 H), 2.40 (s, 3 H), 4.34 (d, ^3J = 7.5 Hz, 2 H), 7.27–7.36 (m, 3 H), 7.61–7.68 (m, 4 H), 7.80 (dd, ^3J = 8.6 Hz, ^4J = 1.8 Hz, 1 H), 8.01 (dd, ^3J = 8.8 Hz, ^4J = 1.7 Hz, 1 H), 8.23 (d, ^3J = 16.3 Hz, 1 H), 8.45 (d, ^4J = 1.7 Hz, 1 H), 8.84 (d, ^4J = 1.7 Hz, 1 H). ^{13}C NMR (75 MHz, acetone-d$_6$/CS$_2$ 4:1): δ 14.5 (CH$_3$),[25] 21.2 (CH$_3$), 23.4 (CH$_2$),[26] 26.3 (CH$_3$), 27.3 (CH$_2$),[26] 30.15 (CH$_2$), 30.18 (CH$_2$), 30.3 (CH$_2$),[26] 30.39 (CH$_2$),[26] 30.4 (CH$_2$), 30.46 (CH$_2$),[26] 30.49 (CH$_2$), 30.7 (CH$_2$),[26] 32.6 (CH$_2$),[26] 32.71 (CH$_2$), 32.73 (CH$_2$), 38.6 (CH), 48.6 (CH$_2$), 97.8 (C$_{quat}$), 99.1 (C$_{quat}$),[27] 111.36 (CH), 111.38 (CH), 111.8 (C$_{quat}$), 112.5 (C$_{quat}$), 112.8 (CH), 113.2 (C$_{quat}$), 119.6 (CH), 124.3 (C$_{quat}$), 124.4 (CH), 124.8 (C$_{quat}$), 126.7 (CH), 126.8 (C$_{quat}$), 127.7 (CH), 128.4 (CH), 130.4 (CH), 134.4 (C$_{quat}$), 137.1 (C$_{quat}$), 139.3 (C$_{quat}$), 141.7 (C$_{quat}$), 144.9 (C$_{quat}$), 150.4 (CH), 176.1 (C$_{quat}$), 177.5 (C$_{quat}$). MS (MALDI-TOF) calcd for $C_{55}H_{70}N_4O$ *m/z:* 802.56; Found: 802.5 ([M]$^+$). IR: $\tilde{\nu}$ [cm^{-1}] = 2922 (m), 2851 (w), 2228 (w), 1565 (m), 1559 (s), 1557 (s), 1528 (s), 1476 (m), 1462 (m), 1433 (w), 1381 (m), 1343 (w), 1283 (s), 1262 (w), 1227 (m), 1209 (w), 1192 (w), 1155 (w), 1138 (m), 1107 (m), 1063 (w), 1024 (w), 1011 (w), 974 (w), 939 (w), 899 (w), 885 (w), 858 (w), 835 (w), 799 (s), 737 (w), 719 (w), 706 (w), 660 (w), 610 (w). Anal calcd for $C_{55}H_{70}N_4O$ [803.2]: C 82.25, H 8.78, N 6.98; Found: C 82.09, H 8.52, N 6.85.

---

[25] Two CH$_3$ signals coincide.
[26] Two CH$_2$ signals coincide.
[27] Two C$_{quat}$ signals coincide.

2.1.4.7. 2-{[9-(2-Decyltetradecyl)-6-(1-methyl-1H-pyrazol-4-yl)-9*H*-carbazol-3-yl]methylene}-1*H*-inden-1,3[2*H*]-dione (11g)

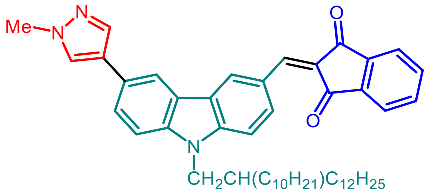

$C_{50}H_{65}N_3O_2$ [740.07]

According to the GP and after purification by chromatography on silica gel (*n*-hexane/acetone 15:1) and drying under vacuo compound **11g** (216 mg, 59%) was obtained as a red amorphous solid, Mp 121–125 °C. R_f (*n*-hexane/acetone 10:1) = 0.05.

^{1}H NMR (600 MHz, acetone-d$_6$/CS$_2$ 4:1): δ 0.88 (t, ^3J = 7.0 Hz, 3 H), 0.89 (t, ^3J = 7.0 Hz, 3 H), 1.16–1.49 (m, 40 H), 2.18 (hep, ^3J = 6.8 Hz, 1 H), 3.96 (s, 3 H), 4.28 (d, ^3J = 7.6 Hz, 2 H), 7.49 (d, ^3J = 8.3 Hz, 1 H), 7.56 (d, ^3J = 8.8 Hz, 1 H), 7.67 (d, ^3J = 8.4 Hz, 1 H), 7.82 (s, 1 H), 7.85–8.01 (m, 6 H), 8.35 (s, 1 H), 8.77 (d, ^3J = 8.6 Hz, 1 H), 9.44 (s, 1 H). ^{13}C NMR (150 MHz, acetone-d$_6$/CS$_2$ 4:1): δ 14.7 (CH_3),[28] 23.6 (CH_2),[29] 27.5 (CH_2),[29] 30.29 (CH_2), 30.30 (CH_2), 30.4 (CH_2),[29] 30.55 (CH_2),[29] 30.59 (CH_2),[29] 30.62 (CH_2), 30.85 (CH_2), 30.86 (CH_2), 32.7 (CH_2),[29] 32.8 (CH_2),[29] 38.7 (CH), 39.2 (CH_3), 48.6 (CH_2), 110.4 (CH), 111.2 (CH), 118.0 (CH), 123.3 (CH), 123.5 (CH), 124.2 (C_{quat}), 124.3 (C_{quat}), 124.5 (C_{quat}), 125.4 (CH), 125.8 (C_{quat}), 126.3 (C_{quat}), 126.9 (C_{quat}), 127.5 (CH), 129.6 (CH), 133.9 (CH), 135.6 (CH), 135.7 (CH), 136.8 (CH), 140.7 (C_{quat}), 141.0 (C_{quat}), 143.2 (C_{quat}), 145.0 (C_{quat}), 148.5 (CH), 189.6 (C_{quat}), 190.4 (C_{quat}). MS (MALDI-TOF) calcd for $C_{50}H_{65}N_3O_2$-H^+ *m/z:* 740.5; Found: 740.5. ESI-HRMS calcd for $C_{50}H_{65}N_3O_2$-H^+: 740.51495; Found: 740.51514 ([MH]$^+$). IR: $\tilde{\nu}$ [cm^{-1}] = 3065 (w), 2951 (w), 2918 (m), 2851 (w), 1717 (w), 1678 (s), 1622 (w), 1595 (w), 1566 (m), 1549 (s), 1491 (m), 1468 (w), 1435 (w), 1383 (m), 1343 (m), 1325 (m), 1294 (w), 1210 (w), 1190 (m), 1175 (w), 1150 (m), 1142 (m), 1092 (w), 1072 (w), 1049 (w), 1018 (w), 993 (m), 970 (w), 963 (w), 924 (w), 845 (w), 808 (m), 791 (m), 760 (w), 733 (s), 721 (m), 708 (w), 685 (w), 648 (w), 665 (w). UV/VIS (CH_2Cl_2) λ_{max} (ε 10^3 [M^{-1}cm^{-1}]) [nm] = 281 (34), 303 (21), 352 (19), 467 (43).

[28] Two CH_3 signals coincide.
[29] Two CH_2 signals coincide.

2.1.4.8. 4-{[6-(4-{Bis[4-methoxyphenyl]amino}phenyl)-9-(2-decyltetradecyl)-9*H*-carbazol-3-yl]methylene}-3-methyl-1-phenyl-1*H*-pyrazol-5[4*H*]-one (11h)

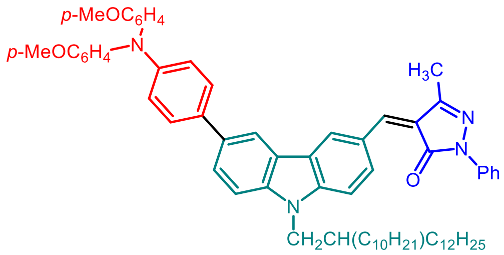

$C_{67}H_{82}N_4O_3$ [991.39]

According to the GP and after purification by chromatography on silica gel (*n*-hexane/acetone 25:1) and drying under vacuo compound **11h** (289 mg, 57%) was obtained as a dark red amorphous solid, Mp softening >40 °C, melting >65 °C. R_f (*n*-hexane/acetone 10:1) = 0.22. ^{1}H NMR (300 MHz, acetone-d$_6$/CS$_2$ 4:1): δ 0.83 (t, ^3J = 6.8 Hz, 3 H), 0.84 (t, ^3J = 6.8 Hz, 3 H), 1.09–1.36 (m, 40 H), 2.07–2.12 (m, 1 H), 2.28 (s, 3 H), 3.79 (s, 6 H), 4.13 (d, ^3J = 6.2 Hz, 2 H), 6.87–7.00 (m, 6 H), 7.03–7.10 (m, 4 H), 7.15 (t, ^3J = 7.5 Hz, 1 H), 7.36–7.51 (m, 4 H), 7.52–7.59 (m, 2 H), 7.22–7.63 (m, 2 H), 8.09–8.16 (m, 2 H), 8.28–8.35 (m, 1 H), 8.82 (d, ^3J = 8.7 Hz, 1 H), 9.48 (s, 1 H). ^{13}C NMR (75 MHz, acetone-d$_6$/CS$_2$ 4:1): δ 13.5 (CH$_3$), 14.4 (CH$_3$),[30] 23.4 (CH$_2$),[31] 27.16 (CH$_2$), 27.18 (CH$_2$), 30.12 (CH$_2$),[31] 30.14 (CH$_2$), 30.2 (CH$_2$), 30.3 (CH$_2$), 30.35 (CH$_2$), 30.37 (CH$_2$), 30.41 (CH$_2$),[31] 30.44 (CH$_2$), 30.5 (CH$_2$), 30.6 (CH$_2$), 32.49 (CH$_2$), 32.51 (CH$_2$), 32.7 (CH$_2$),[31] 38.5 (CH), 48.5 (CH$_2$), 55.7 (CH$_3$), 110.3 (CH), 111.2 (CH), 115.6 (CH), 118.8 (CH), 119.1 (CH), 121.5 (CH), 124.1 (C$_{quat}$), 124.2 (C$_{quat}$), 124.5 (C$_{quat}$), 124.8 (CH), 126.0 (C$_{quat}$), 126.1 (CH), 127.5 (CH), 128.3 (CH), 129.2 (CH), 129.4 (CH), 133.6 (CH), 134.2 (C$_{quat}$), 134.3 (C$_{quat}$), 140.3 (C$_{quat}$), 141.4 (C$_{quat}$), 141.7 (C$_{quat}$), 144.9 (C$_{quat}$), 148.7 (C$_{quat}$), 149.4 (CH), 152.1 (C$_{quat}$), 157.1 (C$_{quat}$), 163.3 (C$_{quat}$). MS (MALDI-TOF) calcd for $C_{67}H_{82}N_4O_3$ *m/z:* 990.64; Found: 990.7 ([M]$^+$). IR: $\tilde{\nu}$ [cm^{-1}] = 2922 (m), 2851 (w), 1678 (w), 1582 (m), 1551 (m), 1505 (s), 1478 (m), 1462 (m), 1441 (w), 1418 (w), 1387 (w), 1379 (w), 1360 (w), 1317 (m), 1277 (w), 1238 (s), 1179 (w), 1159 (w), 1132 (m), 1105 (w), 1065 (w), 1036 (m), 995 (m), 934 (w), 903 (w), 872 (w), 828 (m), 802 (m), 768 (w), 752 (m), 725 (w), 706 (w), 691 (m), 669 (m), 646 (w), 631 (w). Anal calcd for $C_{67}H_{82}N_4O_3$ [991.4]: C 81.17, H 8.34, N 5.65; Found: C 81.08, H 8.23, N 5.57.

[30] Two CH_3 signals coincide.
[31] Two CH_2 signals coincide.

2.1.4.9. 4-{[9-(2-Decyltetradecyl)-6-(10-hexyl-7-{*p*-tolyl}-10*H*-phenothiazin-3-yl)-9*H*-carbazol-3-yl]methylene}-3-methyl-1-phenyl-1H-pyrazol-5[4*H*]-one (11i)

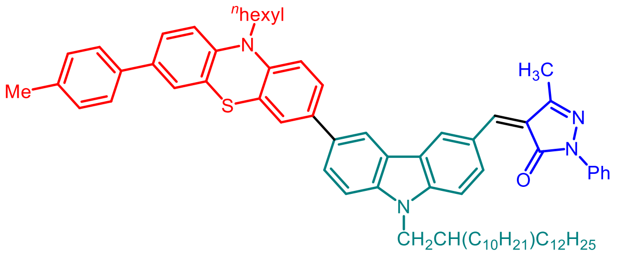

$C_{72}H_{90}N_4OS$ [1059.58]

According to the GP and after purification by chromatography on silica gel (*n*-hexane/acetone 30:1) and drying under vacuo compound **11i** (334 mg, 61%) was obtained as a red amorphous solid, Mp softening >47 °C, melting >60 °C. R_f (*n*-hexane/acetone 10:1) = 0.38.

^{1}H NMR (300 MHz, acetone-d$_6$/CS$_2$ 4:1): δ 0.86–0.96 (m, 9 H), 1.18–1.59 (m, 46 H), 1.89 (quin, ^3J = 6.6 Hz, 2 H), 2.12–2.23 (m, 1 H), 2.37 (s, 6 H), 3.98 (t, ^3J = 7.1 Hz, 2 H), 4.27 (d, ^3J = 7.4 Hz, 2 H), 7.01 (d, ^3J = 8.4 Hz, 1 H), 7.05 (d, ^3J = 8.4 Hz, 1 H), 7.11–7.18 (m, 1 H), 7.22 (d, ^3J = 8.0 Hz, 2 H), 7.36–7.57 (m, 10 H), 7.72 (dd, ^3J = 8.4 Hz, ^4J = 1.9 Hz, 1 H), 7.73 (s, 1 H), 8.09–8.17 (m, 2 H), 8.36 (d, ^4J = 1.7 Hz, 1 H), 8.88 (dd, ^3J = 8.8 Hz, ^4J = 1.7 Hz, 1 H), 9.59 (d, ^4J = 1.6 Hz, 1 H). ^{13}C NMR (75 MHz, acetone-d$_6$/CS$_2$ 4:1): δ 13.7 (CH$_3$), 14.68 (CH$_3$), 14.73 (CH$_3$),[32] 21.4 (CH$_3$), 23.6 (CH$_2$),[33] 23.7 (CH$_2$), 27.49 (CH$_2$),[33] 27.52 (CH$_2$), 27.7 (CH$_2$), 30.3 (CH$_2$),[33] 30.5 (CH$_2$),[33] 30.57 (CH$_2$),[34] 30.61 (CH$_2$),[33] 30.64 (CH$_2$), 30.9 (CH$_2$),[33] 32.5 (CH$_2$), 32.7 (CH$_2$),[33] 32.9 (CH$_2$),[33] 38.8 (CH), 48.1 (CH$_2$), 48.6 (CH$_2$), 110.3 (CH), 111.1 (CH), 116.5 (CH), 116.6 (CH), 119.1 (CH),[35] 124.2 (C$_{quat}$), 124.5 (C$_{quat}$), 124.6 (C$_{quat}$), 124.7 (CH), 125.55 (C$_{quat}$), 125.62 (C$_{quat}$), 126.0 (CH), 126.1 (C$_{quat}$), 126.2 (CH), 126.3 (CH), 126.4 (CH), 126.8 (CH), 127.0 (CH), 129.3 (CH),[35] 130.3 (CH), 133.4 (C$_{quat}$), 133.8 (CH), 136.0 (C$_{quat}$), 136.6 (C$_{quat}$), 137.2 (C$_{quat}$), 137.7 (C$_{quat}$), 140.1 (C$_{quat}$), 141.6 (C$_{quat}$), 144.5 (C$_{quat}$), 144.8 (C$_{quat}$), 144.9 (C$_{quat}$), 149.0 (CH), 151.7 (C$_{quat}$), 163.0 (C$_{quat}$). MS (MALDI-TOF) calcd for $C_{72}H_{90}N_4OS$ *m/z:* 1058.68; Found: 1058.7 ([M]$^+$). IR: $\tilde{\nu}$ [cm^{-1}] = 2951 (w), 2920 (m), 2851 (w), 1676 (w), 1578 (m), 1551 (m), 1495 (w), 1461 (m), 1458 (m), 1389 (w), 1377 (w), 1356 (w), 1317 (m), 1298 (w), 1275 (w), 1256 (m), 1229 (m), 1215 (w), 1190 (w), 1130 (s), 1067 (w), 1018 (w), 995 (m), 932 (w), 901 (w), 874 (w), 802 (s), 785 (w), 768 (m), 752 (m), 720 (w), 704 (w), 691 (m), 669 (w), 646 (w), 615 (w). Anal calcd for $C_{72}H_{90}N_4OS$ [1060]: C 81.61, H 8.56, N 5.29; Found: C 81.55, H 8.53, N 5.21.

[32] Two CH_3 signals coincide.
[33] Two CH_2 signals coincide.
[34] Three CH_3 signals coincide.
[35] Two CH signals coincide.

2.1.5. Consecutive Three-component Suzuki-Knoevenagel Synthesis of Phenothiazine-bridged Systems 12

Table S5. Experimental details of the consecutive three-component Suzuki-Knoevenagel synthesis of phenothiazine-bridged systems **12**.

**Entry**	**Bromo-aldehyde 5** [mg] (mmol)	**Boronic acid/ester 6** [mg] (mmol)	**CsF** [mg] (mmol)	**Pd(PPh$_3$)$_4$** [mg] (μmol)	***t*₁** [h]	**methylene active compound 7** [mg] (mmol)	**Organo catalyst** [mg] (mmol)	***t*₂** [h]	**Product** [mg] (%)
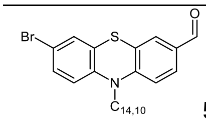 **5**									
59	340 (0.530) of **5**	87 (0.636) of **6b**	258 (1.70)	18 (16)	14	94 (0.636) of **7a**	41 (0.53) of NH_4OAc	6	365 (88) of **12a**
56	324 (0.504) of **5**	82 (0.605) of **6b**	245 (1.61)	18 (15)	16	81 (0.605) of **7b**	39 (0.50) of NH_4OAc	6	335 (87) of **12b**
74	327 (0.509) of **5**	83 (0.611) of **6b**	248 (1.63)	18 (16)	16	89 (0.61) of **7c**	1 drop of Et2NH	4	314 (79) of **12c**
75	324 (0.504) of **5**	82 (0.605) of **6b**	245 (1.61)	18 (16)	16	188 (1.06) of **7c**	47 (0.605) of NH_4OAc	8	134 (32) of **12d**
82	335 (0.521) of **5**	85 (0.625) of **6b**	253 (1.68)	18 (16)	14	109 (0.625) of **7e**	1 drop of Et_2NH	4	325 (77) of **12e**
77	452 (0.703) of **5**	115 (0.843) of **6b**	342 (2.25)	24 (21)	14	137 (0.843) of **7f**	54 (0.70) of NH_4OAc	8	490 (87) of **12f**
88	340 (0.529) of **5**	86 (0.635) of **6b**	257 (1.69)	18 (16)	14	127 (0.635) of **7g**	41 (0.53) of NH_4OAc	6	358 (81) of **12g**
60	321 (0.499) of **5**	137 (0.599) of **6c**	241 (1.60)	17 (15)	16	88 (0.599) of **7a**	39 (0.50) of NH_4OAc	6	375 (95) of **12h**
76	310 (0.482) of **5**	133 (0.578) of **6c**	236 (1.54)	17 (15)	16	85 (0.578) of **7c**	1 drop of Et_2NH	4	309 (81) of **12i**
64	330 (0.513) of **5**	76 (0.616) of **6m**	250 (1.65)	18 (16)	16	91 (0.616) of **7a**	40 (0.51) of NH_4OAc	6	308 (78) of **12j**
65	345 (0.537) of **5**	134 (0.644) of **6l**	261 (1.72)	19 (17)	16	95 (0.644) of **7a**	41 (0.53) of NH_4OAc	6	333 (80) of **12k**
78	331 (0.515) of **5**	129 (0.618) of **6l**	250 (1.65)	18 (16)	16	100 (0.618) of **7f**	40 (0.51) of NH_4OAc	8	288 (71) of **12l**
83	331 (0.515) of **5**	129 (0.618) of **6l**	250 (1.65)	17 (15)	16	108 (0.618) of **7e**	1 drop of Et_2NH	4	288 (69) of **12m**
61	310 (0.482) of **5**	130 (0.578) of **6i**	234 (1.54)	17 (15)	14	85 (0.578) of **7a**	37 (0.48) of NH_4OAc	6	322 (85) of **12n**
62	371 (0.577) of **5**	247 (0.692) of **6k**	281 (1.85)	20 (17)	16	102 (0.692) of **7a**	45 (0.58) of NH_4OAc	6	355 (67) of **12o**
63	363 (0.565) of **5**	255 (0.678) of **6j**	275 (1.81)	20 (17)	16	100 (0.678) of **7a**	44 (0.57) of NH_4OAc	6	391 (74) of **12p**

66	315 (0.490) of **5**	218 (0.588) of **6d**	238 (1.57)	17 (15)	16	**7a** 87 (0.59)	38 (0.49) of NH_4OAc	6	383 (84) of **12q**
79	326 (0.507) of **5**	226 (0.608) of **6d**	247 (1.62)	18 (16)	16	99 (0.608) of **7f**	39 (0.50) of NH_4OAc	8	394 (82) of **12r**
67	322 (0.501) of **5**	259 (0.601) of **6e**	244 (1.60)	17 (15)	16	89 (0.60) of **7a**	39 (0.50) of NH_4OAc	6	417 (83) of **12s**
57	332 (0.516) of **5**	267 (0.619) of **6e**	251 (1.65)	18 (16)	16	83 (0.62) of **7b**	40 (0.51) of NH_4OAc	6	400 (79) of **12t**
80	323 (0.502) of **5**	260 (0.602) of **6e**	245 (1.61)	17 (15)	16	98 (0.602) of **7f**	39 (0.50) of NH_4OAc	8	411 (81) of **12u**
84	330 (0.513) of **5**	266 (0.616) of **6e**	249 (1.64)	18 (16)	16	107 (0.616) of **7e**	1 drop of Et_2NH	4	376 (72) of **12v**
68	338 (0.526) of **5**	315 (0.631) of **6p**	256 (1.68)	18 (16)	16	93 (0.63) of **7a**	41 (0.53) of NH_4OAc	6	446 (80) of **12w**
58	356 (0.554) of **5**	332 (0.665) of **6p**	269 (1.77)	19 (17)	16	89 (0.665) of **7b**	43 (0.55) of NH_4OAc	6	520 (89) of **12x**
81	330 (0.513) of **5**	308 (0.616) of **6p**	250 (1.65)	18 (16)	16	100 (0.616) of **7f**	40 (0.51) of NH_4OAc	8	436 (79) of **12y**
85	320 (0.498) of **5**	299 (0.598) of **6p**	500 (1.59) of Cs_2CO_3	17 (15)	16	104 (0.598) of **7e**	1 drop of Et_2NH	4	371 (68) of **12z**

2.1.5.1. (*Z*)-5-{[10-(2-Decyltetradecyl)-7-(*p*-tolyl)-10*H*-phenothiazin-3-yl]methylene}-3-methyl-2-thioxothiazolidin-4-one (12a)

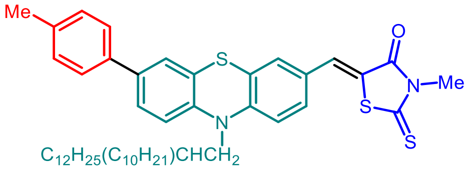

$C_{48}H_{66}N_2OS_3$ [783.25]

According to the GP and after purification by chromatography on silica gel (*n*-hexane, *n*-hexane/acetone 40:1) and drying under vacuo compound **12a** (365 mg, 88%) was obtained as a dark red resin. R_f (*n*-hexane/acetone 10:1) = 0.63.

^{1}H NMR (300 MHz, acetone-d$_6$/CS_2 4:1): δ 0.91 (t, ^3J = 6.5 Hz, 3 H), 0.92 (t, ^3J = 6.5 Hz, 3 H), 1.21–1.52 (m, 40 H), 1.99–2.04 (m, 1 H), 2.38 (s, 3 H), 3.47 (s, 3 H), 3.92 (d, ^3J = 7.1 Hz, 2 H), 7.06 (d, ^3J = 8.5 Hz, 1 H), 7.10 (d, ^3J = 8.6 Hz, 1 H), 7.19–7.25 (m, 2 H), 7.33 (d, ^4J = 2.1 Hz, 1 H), 7.37 (d, ^4J = 2.1 Hz, 1 H), 7.40–7.48 (m, 4 H), 7.61 (s, 1 H). ^{13}C NMR (75 MHz, acetone-d$_6$/CS_2 4:1): δ 14.8 (2 CH_3),[36] 21.5 (CH_3), 23.7 (2 CH_2),[37] 27.12 (CH_2), 27.14 (CH_2), 30.4 (2 CH_2),[37] 30.5 (2 CH_2),[37] 30.6 (3 CH_2),[38] 30.65 (CH_2), 30.68 (CH_2), 30.70 (CH_2), 30.99 (2 CH_2),[37] 31.5 (CH_3), 32.3 (2 CH_2),[37] 32.9 (2 CH_2),[37] 35.7 (CH), 52.3 (CH_2), 117.2 (CH), 117.7 (CH),

[36] Two CH_3 signals coincide.
[37] Two CH_2 signals coincide.
[38] Three CH_2 signals coincide.

121.1 (C_{quat}), 125.8 (C_{quat}), 126.3 (CH), 126.7 (CH), 126.9 (C_{quat}), 127.0 (CH), 128.5 (C_{quat}), 130.2 (CH), 130.3 (CH), 131.5 (CH), 132.6 (CH), 137.2 (C_{quat}), 137.4 (C_{quat}), 137.5 (C_{quat}), 143.8 (C_{quat}), 148.7 (C_{quat}), 167.6 (C_{quat}), 193.3 (C_{quat}). MS (MALDI-TOF) calcd for $C_{48}H_{66}N_2OS_3$ *m/z:* 782.43; Found: 782.4 ($[M]^+$). IR: $\tilde{\nu}$ [cm^{-1}] = 3023 (w), 2920 (m), 2851 (m), 1709 (m), 1595 (w), 1574 (m), 1543 (w), 1495 (w), 1460 (s), 1424 (m), 1402 (m), 1348 (m), 1285 (s), 1250 (m), 1221 (m), 1167 (w), 1126 (s), 1099 (s), 1057 (w), 1017 (w), 990 (w), 959 (w), 901 (w), 885 (w), 804 (s), 781 (w), 762 (w), 725 (w), 721 (w), 692 (w), 673 (w), 652 (w), 613 (w). Anal calcd for $C_{48}H_{66}N_2OS_3$ [783.3]: C 73.61, H 8.49, N 3.58, S 12.28; Found: C 73.65, H 8.41, N 3.51, S 12.34.

2.1.5.2. (*Z*)-5-{[10-(2-Decyltetradecyl)-7-(*p*-tolyl)-10*H*-phenothiazin-3-yl]methylene}-2-thioxothiazolidin-4-one (12b)

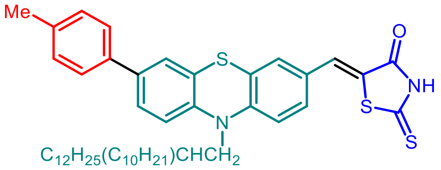

$C_{47}H_{64}N_2OS_3$ [769.22]

According to the GP and after purification by chromatography on silica gel (*n*-hexane/acetone 15:1) and drying under vacuo compound **12b** (337 mg, 87%) was obtained as a dark red resin. R_f (*n*-hexane/acetone 10:1) = 0.18.

^1H NMR (300 MHz, acetone-d$_6$/CS_2 4:1): δ 0.905 (t, ^3J = 6.8 Hz, 3 H), 0.914 (t, ^3J = 6.8 Hz, 3 H), 1.20–1.55 (m, 40 H), 2.05–2.09 (m, 1 H), 2.38 (s, 3 H), 3.93 (d, ^3J = 7.1 Hz, 2 H), 7.07 (d, ^3J = 8.6 Hz, 1 H), 7.12 (d, ^3J = 8.7 Hz, 1 H), 7.22 (d, ^3J = 7.9 Hz, 2 H), 7.32 (d, ^4J = 2.1 Hz, 1 H), 7.38 (d, ^4J = 2.0 Hz, 1 H), 7.38–7.49 (m, 5 H), 12.03 (br, 1 H). ^{13}C NMR (75 MHz, acetone-d$_6$/CS_2 4:1): δ 14.8 (CH_3),[39] 21.4 (CH_3), 23.7 (CH_2),[40] 27.13 (CH_2),[40] 30.4 (CH_2),[40] 30.5 (CH_2),[40] 30.6 (CH_2),[41] 30.70 (CH_2),[40] 31.0 (CH_2),[40] 32.3 (CH_2),[40] 32.9 (CH_2),[40] 35.7 (CH), 52.3 (CH_2), 117.2 (CH), 117.7 (CH), 123.8 (C_{quat}), 125.8 (C_{quat}), 126.3 (CH), 126.7 (CH), 126.9 (C_{quat}), 127.1 (CH), 128.5 (C_{quat}), 130.1 (CH), 130.3 (CH), 131.3 (CH), 131.7 (CH), 137.1 (C_{quat}), 137.4 (C_{quat}), 137.5 (C_{quat}), 143.9 (C_{quat}), 148.6 (C_{quat}), 169.1 (C_{quat}), 194.6 (C_{quat}). MS (MALDI-TOF) calcd for $C_{47}H_{64}N_2OS_3$ *m/z:* 768.42; Found: 768.4 ($[M]^+$). ESI-HRMS calcd for $C_{47}H_{64}N_2OS_3$: 768.41808; Found: 768.4183 ($[M]^+$). IR: $\tilde{\nu}$ [cm^{-1}] = 3066 (w), 3026 (w), 2922 (m), 2851 (m), 1686 (m), 1595 (w), 1570 (m), 1499 (w), 1458 (s), 1437 (w), 1404 (m), 1389 (w), 1375 (w), 1341 (w), 1319 (w), 1289 (w), 1279 (w), 1258 (w), 1234 (m), 1217 (w), 1192 (s), 1179 (s), 1126 (w), 1107 (w), 1063

[39] Two CH_3 signals coincide.
[40] Two CH_2 signals coincide.
[41] Four CH_2 signals coincide.

(w), 1018 (w), 997 (w), 922 (w), 883 (w), 856 (w), 806 (m), 770 (w), 741 (w), 718 (w), 700 (w), 660 (w).

2.1.5.3. 2-{[10-(2-Decyltetradecyl)-7-(*p*-tolyl)-10*H*-phenothiazin-3-yl]methylene}-1*H*-inden-1,3[2*H*]-dione (12c)

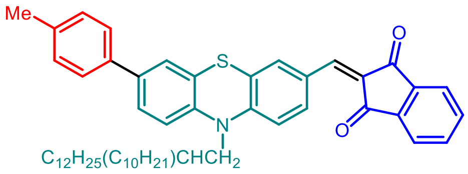

$C_{53}H_{67}NO_2S$ [782.49]

According to the GP and after purification by chromatography on silica gel (*n*-hexane/ethyl acetate 25:1) and drying under vacuo compound **12c** (314 mg, 79%) was obtained as a dark red resin. R_f (*n*-hexane/ethyl acetate 10:1) = 0.28.

^{1}H NMR (300 MHz, acetone-d$_6$/CS_2 4:1): δ 0.82 (t, ^3J = 6.7 Hz, 3 H), 0.84 (t, ^3J = 6.7 Hz, 3 H), 1.13–1.46 (m, 40 H), 1.98–2.04 (m, 1 H), 2.34 (s, 3 H), 3.93 (d, ^3J = 7.1 Hz, 2 H), 7.09 (t, ^3J = 8.4 Hz, 2 H), 7.23 (d, ^3J = 8.0 Hz, 2 H), 7.40 (d, ^4J = 2.2 Hz, 1 H), 7.47 (dd, ^3J = 8.4 Hz, ^4J = 2.2 Hz, 1 H), 7.51 (d, ^3J = 8.1 Hz, 2 H), 7.66 (s, 1 H), 7.83–7.98 (m, 4 H), 8.33 (dd, ^3J = 8.7 Hz, ^4J = 2.1 Hz, 1 H), 8.59 (d, ^4J = 2.1 Hz, 1 H). ^{13}C NMR (75 MHz, acetone-d$_6$/CS_2 4:1): δ 14.4 (CH_3),[42] 21.3 (CH_3), 23.4 (CH_2),[43] 26.7 (CH_2), 26.8 (CH_2), 30.12 (CH_2), 30.14 (CH_2),[43] 30.36 (CH_2), 30.39 (CH_2), 30.40 (CH_2),[43] 30.43 (CH_2),[43] 30.5 (CH_2), 30.58 (CH_2), 30.63 (CH_2), 31.87 (CH_2), 31.91 (CH_2), 32.66 (CH_2), 32.67 (CH_2), 35.5 (CH), 52.3 (CH_2), 116.7 (CH), 118.2 (CH), 123.5 (CH), 123.6 (CH), 125.3 (C_{quat}), 125.8 (C_{quat}), 126.1 (CH), 126.6 (CH), 127.0 (CH), 127.5 (C_{quat}), 128.9 (C_{quat}), 130.4 (CH), 133.6 (CH), 135.9 (CH), 136.1 (CH), 136.6 (CH), 137.3 (C_{quat}), 137.4 (C_{quat}), 137.7 (C_{quat}), 140.8 (C_{quat}), 143.2 (C_{quat}), 143.5 (C_{quat}), 145.5 (CH), 151.2 (C_{quat}), 189.8 (C_{quat}), 190.4 (C_{quat}). MS (MALDI-TOF) calcd for $C_{53}H_{67}NO_2S$ *m/z:* 781.49; Found: 781.5 ([M]$^+$). ESI-HRMS calcd for $C_{53}H_{67}NO_2S$: 781.48925; Found: 781.48968 ([M]$^+$). IR: $\tilde{\nu}$ [cm^{-1}] = 3061 (w), 2955 (w), 2920 (m), 2851 (m), 1724 (w), 1678 (m), 1597 (w), 1562 (m), 1537 (m), 1497 (w), 1460 (s), 1412 (m), 1377 (w), 1331 (m), 1317 (m), 1277 (w), 1258 (m), 1198 (s), 1173 (m), 1153 (m), 1018 (m), 997 (m), 961 (w), 922 (w), 909 (w), 882 (w), 804 (s), 735 (s), 694 (w), 675 (w), 602 (w).

---

[42] Two CH_3 signals coincide.

[43] Two CH_2 signals coincide.

2.1.5.4. 11-[10-(2-Decyltetradecyl)-7-(*p*-tolyl)-10*H*-phenothiazin-3-yl]diindeno[1,2-*b*:2',1'-*e*]pyridin-10,12-dione (12d)

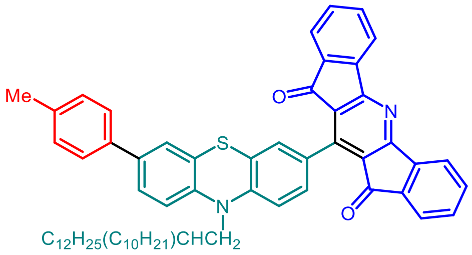

$C_{62}H_{70}N_2O_2S$ [907.30]

According to the GP and after purification by chromatography on silica gel (*n*-hexane/acetone 30:1) and drying under vacuo compound **12d** (143 mg, 32%) was obtained as a red brown resin. R_f (*n*-hexane/ethyl acetate 20:1) = 0.20.

^{1}H NMR (300 MHz, CD_2Cl_2): δ 0.75–0.96 (m, 6 H), 1.09–1.58 (m, 40 H), 2.09 (hep, ^3J = 6.1 Hz, 1 H), 2.38 (s, 3 H), 3.86 (d, ^3J = 7.1 Hz, 2 H), 6.99 (dd, ^3J = 8.8 Hz, ^4J = 2.9 Hz, 2 H), 7.23 (d, ^3J = 7.8 Hz, 2 H), 7.39–7.55 (m, 6 H), 7.58–7.70 (m, 3 H), 7.73 (d, ^3J = 7.3 Hz, 1 H), 7.87 (d, ^4J = 2.1 Hz, 1 H), 7.91 (dd, ^3J = 7.7 Hz, ^4J = 3.3 Hz, 2 H), 8.81 (d, ^3J = 7.5 Hz, 1 H). ^{13}C NMR (75 MHz, CD_2Cl_2): δ 14.5 (CH_3),[44] 21.4 (CH_3), 23.3 (CH_2),[45] 26.86 (CH_2), 26.89 (CH_2), 30.0 (CH_2),[45] 30.1 (CH_2),[45] 30.27 (CH_2),[46] 30.30 (CH_2), 30.32 (CH_2), 30.62 (CH_2), 30.63 (CH_2), 32.15 (CH_2), 32.16 (CH_2), 32.5 (CH_2),[45] 35.4 (CH), 52.3 (CH_2), 115.2 (CH), 117.1 (CH), 121.0 (C_{quat}), 122.4 (CH), 124.1 (C_{quat}), 124.25 (CH), 124.30 (CH), 124.6 (C_{quat}), 126.1 (CH), 126.3 (C_{quat}), 126.7 (CH), 128.3 (CH), 130.0 (2 CH), 130.1 (CH), 130.9 (CH), 131.1 (C_{quat}), 132.7 (CH), 133.0 (CH), 135.4 (C_{quat}), 135.5 (CH), 135.7 (CH), 136.3 (C_{quat}), 137.1 (C_{quat}), 137.4 (C_{quat}), 137.5 (C_{quat}), 140.0 (C_{quat}), 143.3 (C_{quat}), 144.4 (C_{quat}), 148.7 (C_{quat}), 152.2 (C_{quat}), 160.0 (C_{quat}), 168.8 (C_{quat}), 191.1 (C_{quat}), 191.3 (C_{quat}). MS (MALDI-TOF) calcd for $C_{62}H_{70}N_2O_2S$ *m/z:* 906.52; Found: 906.5 ($[M]^+$). ESI-HRMS calcd for $C_{62}H_{70}N_2O_2S$: 906.51580; Found: 906.51602 ($[M]^+$). IR: $\tilde{\nu}$ [cm^{-1}] = 3030 (w), 2922 (m), 2851 (m), 2220 (w), 1686 (w), 1597 (w), 1572 (m), 1541 (w), 1460 (s), 1402 (m), 1395 (m), 1377 (m), 1339 (m), 1308 (m), 1277 (m), 1252 (m), 1207 (s), 1173 (m), 1105 (w), 1076 (w), 1022 (w), 999 (w), 957 (w), 939 (w), 883 (m), 816 (m), 789 (w), 760 (s), 741 (w), 720 (m), 696 (s), 629 (w), 611 (m).

---

[44] Two CH_3 signals coincide.

[45] Two CH_2 signals coincide.

[46] Four CH_2 signals coincide.

2.1.5.5. (*Z*)-4-[(10-(2-Decyltetradecyl)-7-(*p*-tolyl)-10*H*-phenothiazin-3-yl)methylene]-3-methyl-1-phenyl-1*H*-pyrazol-5[4*H*]-one (12e)

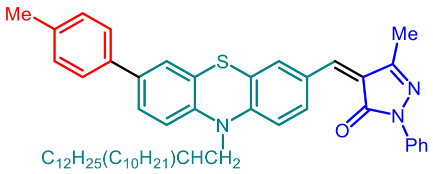

$C_{54}H_{71}N_3OS$ [810.23]

According to the GP and after purification by chromatography on silica gel (*n*-hexane, *n*-hexane/ethyl acetate 30:1) and drying under vacuo compound **12e** (325 mg, 77%) was obtained as a dark red resin. R_f (*n*-hexane/acetone 10:1) = 0.22.

^{1}H NMR (300 MHz, acetone-d$_6$/CS$_2$ 4:1): δ 0.90 (t, ^3J = 6.7 Hz, 3 H), 0.91 (t, ^3J = 6.7 Hz, 3 H), 1.23–1.52 (m, 40 H), 2.06–2.12 (m, 1 H), 2.43 (s, 3 H), 2.39 (s, 3 H), 3.97 (d, ^3J = 7.1 Hz, 2 H), 7.08 (dd, ^3J = 8.6 Hz, ^4J = 2.5 Hz, 2 H), 7.12–7.17 (m, 1 H), 7.23 (d, ^3J = 7.9 Hz, 2 H), 7.34–7.49 (m, 7 H), 8.00–8.10 (m, 2 H), 8.50 (dd, ^3J = 8.7 Hz, ^4J = 2.1 Hz, 1 H), 8.67 (d, ^4J = 2.1 Hz, 1 H). ^{13}C NMR (75 MHz, acetone-d$_6$/CS$_2$ 4:1): δ 13.6 (CH_3), 14.8 (CH_3),[47] 21.5 (CH_3), 23.7 (CH_2),[48] 27.1 (CH_2),[48] 30.4 (CH_2),[48] 30.5 (CH_2),[48] 30.6 (CH_2),[49] 30.67 (CH_2), 30.69 (CH_2), 31.0 (CH_2),[48] 32.2 (CH_2),[48] 32.9 (CH_2),[48] 35.8 (CH), 52.4 (CH_2), 116.3 (CH), 117.9 (CH), 119.0 (CH), 124.8 (CH), 125.4 (C_{quat}), 125.5 (C_{quat}), 126.1 (C_{quat}), 126.3 (CH), 126.6 (CH), 127.1 (CH), 129.0 (C_{quat}), 129.2 (CH), 130.3 (CH), 133.7 (CH), 135.9 (CH), 137.3 (C_{quat}), 137.4 (C_{quat}), 137.5 (C_{quat}), 139.9 (C_{quat}), 143.4 (C_{quat}), 146.1 (CH), 150.8 (C_{quat}), 151.5 (C_{quat}), 162.7 (C_{quat}). MS (MALDI-TOF) calcd for $C_{54}H_{71}N_3OS$-H^+ *m/z:* 810.54; Found: 810.5. IR: $\tilde{\nu}$ [cm^{-1}] = 3061 (w), 2920 (m), 2851 (m), 1678 (w), 1614 (w), 1597 (m), 1574 (m), 1557 (m), 1537 (w), 1497 (m), 1460 (s), 1418 (m), 1389 (w), 1356 (w), 1341 (w), 1317 (s), 1279 (w), 1250 (m), 1215 (s), 1190 (w), 1138 (m), 1109 (w), 1022 (w), 955 (m), 930 (w), 903 (w), 882 (w), 806 (m), 785 (w), 768 (m), 752 (m), 725 (w), 691 (m), 662 (w). Anal calcd for $C_{54}H_{71}N_3OS$ [810.2]: C 80.05, H 8.83, N 5.19, S 3.96; Found: C 80.29, H 8.82, N 5.27, S 3.95.

2.1.5.6. (*Z*)-3-[10-(2-Decyltetradecyl)-7-(*p*-tolyl)-10H-phenothiazin-3-yl]-2-(4-nitrophenyl)acrylonitrile (12f)

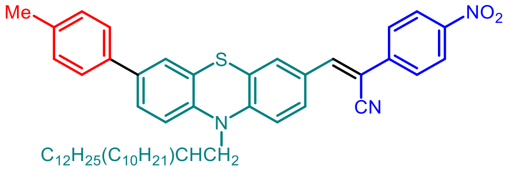

$C_{52}H_{67}N_3O_2S$ [798.17]

---

[47] Two CH_3 signals coincide.
[48] Two CH_2 signals coincide.
[49] Four CH_2 signals coincide.

According to the GP and after purification by chromatography on silica gel (*n*-hexane, *n*-hexane/ethyl acetate 30:1) and drying under vacuo compound **12f** (490 mg, 87%) was obtained as a dark red resin. R_f (*n*-hexane/ethyl acetate 20:1) = 0.22.

^{1}H NMR (600 MHz, acetone-d$_6$): δ 0.84 (t, ^3J = 7.0 Hz, 3 H), 0.85 (t, ^3J = 7.0 Hz, 3 H), 1.16–1.48 (m, 40 H), 1.99–2.05 (m, 1 H), 2.35 (s, 3 H), 3.94 (d, ^3J = 7.2 Hz, 2 H), 7.09–7.17 (m, 2 H), 7.24 (d, ^3J = 7.9 Hz, 2 H), 7.41 (d, ^4J = 2.1 Hz, 1 H), 7.49 (dd, ^3J = 8.4 Hz, ^4J = 2.2 Hz, 1 H), 7.51 (d, ^3J = 8.0 Hz, 2 H), 7.84 (s, 1 H), 7.95 (dd, ^3J = 8.6 Hz, ^4J = 2.2 Hz, 1 H), 7.97–8.00 (m, 3 H), 8.32 (d, ^3J = 8.5 Hz, 2 H). ^{13}C NMR (150 MHz, acetone-d$_6$): δ 14.4 (CH_3),[50] 21.1 (CH_3), 23.4 (CH_2),[51] 26.7 (CH_2), 26.8 (CH_2), 30.1 (CH_2),[52] 30.2 (CH_2), 30.35 (CH_2), 30.37 (CH_2), 30.39 (CH_2), 30.42 (CH_2), 30.43 (CH_2), 30.5 (CH_2), 30.60 (CH_2), 30.64 (CH_2), 31.9 (CH_2), 32.0 (CH_2), 32.66 (CH_2), 32.67 (CH_2), 35.5 (CH), 52.2 (CH_2), 106.49 (C_{quat}), 117.1 (CH), 118.0 (CH), 118.5 (C_{quat}), 125.1 (CH), 125.7 (C_{quat}), 126.09 (C_{quat}), 126.13 (CH), 126.7 (CH), 127.0 (CH), 127.4 (CH), 128.6 (C_{quat}), 129.5 (CH), 130.4 (2 CH), 130.9 (CH), 137.1 (C_{quat}), 137.4 (C_{quat}), 137.7 (C_{quat}), 142.0 (C_{quat}), 144.0 (C_{quat}), 145.1 (CH), 148.4 (C_{quat}), 149.5 (C_{quat}). MS (MALDI-TOF) calcd for $C_{52}H_{67}N_3O_2S$ *m/z:* 797.50; Found: 797.5 ($[M]^+$). IR: $\tilde{\nu}$ [cm^{-1}] = 3024 (w), 2920 (m), 2851 (m), 2212 (w), 1603 (w), 1570 (m), 1520 (m), 1497 (w), 1462 (s), 1418 (w), 1402 (m), 1377 (w), 1337 (s), 1277 (m), 1206 (m), 1175 (m), 1047 (w), 1036 (w), 1003 (w), 918 (w), 881 (w), 872 (w), 851 (m), 806 (s), 781 (w), 752 (m), 719 (w), 688 (w), 663 (w), 623 (w). Anal calcd for $C_{52}H_{67}N_3O_2S$ [798.2]: C 78.25, H 8.46, N 5.26; Found: C 77.96, H 8.23, N 5.18.

2.1.5.7. (*E*)-2-{3-Cyano-4-[2-(10-{2-decyltetradecyl}-7-{*p*-tolyl}-10*H*-phenothiazin-3-yl)vinyl]-5,5-dimethylfuran-2[5*H*]-yliden}malonitrile (12g)

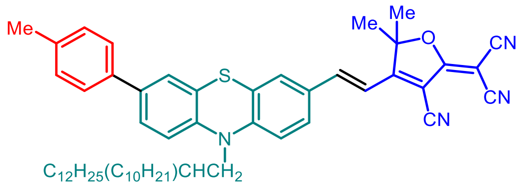

$C_{55}H_{70}N_4OS$ [835.24]

According to the GP and after purification by chromatography on silica gel (*n*-hexane, *n*-hexane/acetone 30:1) and drying under vacuo compound **12g** (358 mg, 81%) was obtained as a dark red resin. R_f (*n*-hexane/acetone 3:1) = 0.24.

^{1}H NMR (300 MHz, acetone-d$_6$/CS_2 4:1): δ 0.90 (t, ^3J = 6.7 Hz, 3 H), 0.91 (t, ^3J = 6.7 Hz, 3 H), 1.21–1.52 (m, 40 H), 1.87 (s, 6 H), 2.01–2.07 (m, 1 H), 2.38 (s, 3 H), 3.96 (d, ^3J = 7.1 Hz, 2 H), 7.10 (d, ^3J = 8.8 Hz, 2 H), 7.15 (d, ^3J = 16.3 Hz, 1 H), 7.20–7.26 (m, 2 H), 7.37 (d, ^4J = 2.1 Hz,

---

[50] Two CH_3 signals coincide.

[51] Two CH_2 signals coincide.

[52] Three CH_2 signals coincide.

1 H), 7.42–7.49 (m, 3 H), 7.66–7.73 (m, 2 H), 7.91 (d, ^3J = 16.3 Hz, 1 H). ^{13}C NMR (75 MHz, acetone-d$_6$/CS_2 4:1): δ 14.7 (CH_3),[53] 21.4 (CH_3), 23.6 (CH_2),[54] 26.2 (CH_3), 27.1 (CH_2),[54] 30.30 (CH_2), 30.31 (CH_2), 30.4 (CH_2),[54] 30.58 (CH_2),[55] 30.60 (CH_2), 30.62 (CH_2), 30.64 (CH_2), 31.0 (CH_2),[54] 32.2 (CH_2),[54] 32.8 (CH_2),[54] 35.8 (CH), 52.5 (CH_2), 98.7 (C_{quat}), 98.9 (C_{quat}),[56] 111.3 (C_{quat}), 112.0 (C_{quat}), 112.8 (C_{quat}), 113.7 (CH), 117.1 (CH), 118.0 (CH), 125.7 (C_{quat}), 126.2 (CH), 126.6 (C_{quat}), 126.7 (CH), 127.0 (CH), 128.7 (CH), 129.9 (C_{quat}), 130.4 (CH), 130.9 (CH), 137.3 (C_{quat}),[56] 137.6 (C_{quat}), 143.5 (C_{quat}), 147.1 (CH), 150.5 (C_{quat}), 175.2 (C_{quat}), 177.0 (C_{quat}). MS (MALDI-TOF) calcd for $C_{55}H_{70}N_4OS$ *m/z:* 834.53; Found: 834.6 ($[M]^+$). IR: $\tilde{\nu}$ [cm^{-1}] = 2955 (w), 2922 (m), 2853 (w), 2220 (w), 2210 (w), 1601 (w), 1568 (m), 1516 (s), 1495 (m), 1454 (s), 1420 (w), 1381 (w), 1339 (s), 1302 (s), 1279 (m), 1248 (m), 1209 (s), 1184 (m), 1155 (m), 1107 (m), 1057 (w), 1015 (w), 964 (m), 930 (w), 897 (w), 806 (m), 714 (w), 658 (w), 617 (w). Anal calcd for $C_{55}H_{70}N_4OS$ [835.2]: C 79.09, H 8.45, N 6.71, S 3.84; Found: C 79.00, H 8.41, N 6.63, S 3.89.

2.1.5.8. (*Z*)-5-{[10-(2-Decyltetradecyl)-7-(*p*-tolyl)-10*H*-phenothiazin-3-yl]methylene}-3-methyl-2-thioxothiazolidin-4-one (12h)

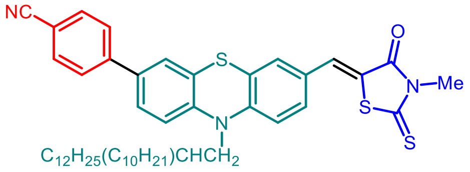

$C_{48}H_{63}N_3OS_3$ [794.23]

According to the GP and after purification by chromatography on silica gel (*n*-hexane, *n*-hexane/acetone 40:1) and drying under vacuo compound **12h** (375 mg, 95%) was obtained as a dark red solid, Mp 120–126 °C. R_f (*n*-hexane/acetone 10:1) = 0.51.

^{1}H NMR (300 MHz, CD_2Cl_2): δ 0.86 (t, ^3J = 6.7 Hz, 3 H), 0.88 (t, ^3J = 6.7 Hz, 3 H), 1.17–1.44 (m, 40 H), 1.94–2.04 (m, 1 H), 3.49 (s, 3 H), 3.82 (d, ^3J = 7.1 Hz, 2 H), 6.98 (d, ^3J = 8.4 Hz, 1 H), 7.01 (d, ^3J = 8.3 Hz, 1 H), 7.28 (d, ^4J = 2.2 Hz, 1 H), 7.35 (dd, ^3J = 8.4 Hz, ^4J = 2.2 Hz, 1 H), 7.41 (d, ^4J = 2.1 Hz, 1 H), 7.45 (dd, ^3J = 8.4 Hz, ^4J = 2.2 Hz, 1 H), 7.61 (s, 1 H), 7.63–7.74 (m, 4 H). ^{13}C NMR (75 MHz, CD_2Cl_2): δ 14.5 (CH_3),[57] 23.3 (CH_2),[58] 26.7 (CH_2),[58] 29.9 (CH_2),[58] 30.0 (CH_2),[58] 30.18 (CH_2),[58] 30.20 (CH_2), 30.24 (CH_2),[58] 30.3 (CH_2), 30.45 (CH_2),[58] 31.7 (CH_3), 31.9 (CH_2),[58] 32.5 (CH_2),[58] 35.3 (CH), 52.4 (CH_2), 111.2 (C_{quat}), 117.0 (CH), 117.5 (CH), 119.4 (C_{quat}), 121.1 (C_{quat}), 126.1 (C_{quat}), 126.5 (CH), 126.6 (C_{quat}), 126.9 (CH), 127.4 (CH), 128.5

[53] Two CH_3 signals coincide.
[54] Two CH_2 signals coincide.
[55] Three CH_2 signals coincide.
[56] Two quaternary signals coincide.
[57] Two CH_3 signals coincide.
[58] Two CH_2 signals coincide.

(C_{quat}), 129.9 (CH), 131.4 (CH), 132.5 (CH), 133.2 (CH), 134.7 (C_{quat}), 144.5 (C_{quat}), 145.3 (C_{quat}), 148.2 (C_{quat}), 168.2 (C_{quat}), 193.9 (C_{quat}). MS (MALDI-TOF) calcd for $C_{48}H_{63}N_3OS_3$ *m/z:* 793.41; Found: 793.4 ($[M]^+$). IR: $\tilde{\nu}$ [cm^{-1}] = 2920 (m), 2850 (w), 2225 (w), 1705 (m), 1565 (m), 1475 (m), 1398 (m), 1356 (w), 1279 (s), 1223 (m), 1126 (m), 1100 (s), 835 (m), 795 (m), 713 (w). Anal calcd for $C_{48}H_{63}N_3OS_3$ [794.2]: C 72.59, H 8.00, N 5.29; Found: C 72.38, H 7.78, N 5.27.

2.1.5.9. 4-{10-(2-Decyltetradecyl)-7-[(1,3-dioxo-1H-inden-2[3*H*]-yliden)methyl]-10*H*-phenothiazin-3-yl}benzonitrile (12i)

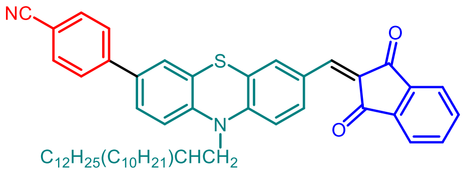

$C_{53}H_{64}N_2O_2S$ [793.15]

According to the GP and after purification by chromatography on silica gel (*n*-hexane/acetone 20:1) and drying under vacuo compound **12i** (309 mg, 81%) was obtained as a dark red resin. R_f (*n*-hexane/acetone 10:1) = 0.18.

^{1}H NMR (300 MHz, acetone-d$_6$/CS_2 4:1): δ 0.87 (t, ^3J = 6.6 Hz, 3 H), 0.88 (t, ^3J = 6.6 Hz, 3 H), 1.20–1.53 (m, 40 H), 2.07–2.13 (m, 1 H), 4.01 (d, ^3J = 7.2 Hz, 2 H), 7.16 (t, ^3J = 8.9 Hz, 2 H), 7.52 (d, ^4J = 2.2 Hz, 1 H), 7.58 (dd, ^3J = 8.5 Hz, ^4J = 2.2 Hz, 1 H), 7.68 (s, 1 H), 7.75–8.00 (m, 8 H), 8.38 (dd, ^3J = 8.7 Hz, ^4J = 2.1 Hz, 1 H), 8.62 (d, ^4J = 2.1 Hz, 1 H). ^{13}C NMR (75 MHz, acetone-d$_6$/CS_2 4:1): δ 14.7 (CH_3),[59] 23.6 (CH_2),[60] 27.0 (CH_2), 27.1 (CH_2), 30.3 (CH_2),[60] 30.4 (CH_2),[60] 30.6 (CH_2),[61] 30.6 (CH_2), 30.7 (CH_2), 30.9 (CH_2),[60] 32.2 (CH_2),[60] 32.8 (CH_2),[60] 35.8 (CH), 52.5 (CH_2), 111.6 (C_{quat}), 116.8 (CH), 118.2 (CH), 119.1 (C_{quat}), 123.5 (CH), 123.7 (CH), 125.4 (C_{quat}), 126.5 (C_{quat}), 126.8 (CH), 127.3 (CH), 127.7 (C_{quat}), 127.9 (CH), 129.2 (C_{quat}), 133.4 (CH), 133.8 (CH), 135.1 (C_{quat}), 135.9 (CH), 136.0 (CH), 136.5 (CH), 140.8 (C_{quat}), 143.2 (C_{quat}), 144.5 (C_{quat}), 145.1 (C_{quat}), 145.3 (CH), 150.7 (C_{quat}), 189.5 (C_{quat}), 190.0 (C_{quat}). MS (MALDI-TOF) calcd for $C_{53}H_{64}N_2O_2S$ *m/z:* 792.47; Found: 792.5 ($[M]^+$). IR: $\tilde{\nu}$ [cm^{-1}] = 3065 (w), 2920 (m), 2851 (w), 2224 (w), 1722 (w), 1678 (m), 1597 (m), 1560 (s), 1537 (m), 1493 (w), 1460 (s), 1412 (m), 1375 (w), 1337 (m), 1279 (m), 1250 (m), 1198 (s), 1175 (m), 1153 (m), 1107 (w), 1084 (m), 1045 (w), 1018 (w), 997 (m), 961 (w), 922 (w), 909 (w), 883 (w), 841 (w), 812 (m), 785 (w), 735 (m), 694 (w), 675 (w). Anal calcd for $C_{53}H_{64}N_2O_2S$ [793.2]: C 80.26, H 8.13, N 3.53, S 4.04; Found: C 80.27, H 7.91, N 3.50, S 4.02.

[59] Two CH_3 signals coincide.
[60] Two CH_2 signals coincide.
[61] Four CH_2 signals coincide.

2.1.5.10. (Z)-5-{[10-(2-Decyltetradecyl)-7-(pyridin-4-yl)-10*H*-phenothiazin-3-yl]methylene}-3-methyl-2-thioxothiazolidin-4-one (12j)

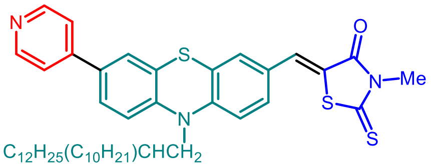

$C_{46}H_{63}N_3OS_3$ [770.21]

According to the GP and after purification by chromatography on silica gel (*n*-hexane/acetone 20:1) and drying under vacuo compound **12j** (308 mg, 78%) was obtained as a dark red resin. R_f (*n*-hexane/acetone 1:2) = 0.46.

^{1}H NMR (300 MHz, acetone-d$_6$/CS$_2$ 4:1): δ 0.89 (t, ^3J = 6.7 Hz, 3 H), 0.91 (t, ^3J = 6.7 Hz, 3 H), 1.22–1.51 (m, 40 H), 2.02–2.08 (m, 1 H), 3.47 (s, 3 H), 3.96 (d, ^3J = 7.2 Hz, 2 H), 7.16 (dd, ^3J = 8.6 Hz, ^4J = 1.6 Hz, 2 H), 7.36 (d, ^4J = 2.1 Hz, 1 H), 7.46 (dd, ^3J = 8.6 Hz, ^4J = 2.2 Hz, 1 H), 7.55 (d, ^4J = 2.2 Hz, 1 H), 7.55–7.58 (m, 2 H), 7.61 (dd, ^3J = 8.5 Hz, ^4J = 2.1, 1 H), 7.63 (s, 1 H), 8.55–8.61 (m, 2 H). ^{13}C NMR (75 MHz, acetone-d$_6$/CS$_2$ 4:1): δ 14.7 (CH$_3$),[62] 23.6 (CH$_2$),[63] 27.06 (CH$_2$), 27.07 (CH$_2$), 30.3 (CH$_2$),[63] 30.4 (CH$_2$),[63] 30.59 (CH$_2$),[64] 30.62 (CH$_2$), 30.63 (CH$_2$), 30.7 (CH$_2$), 30.93 (2 CH$_2$),[63] 31.6 (CH), 32.2 (CH$_2$),[63] 32.9 (CH$_2$),[63] 35.7 (CH), 52.4 (CH$_2$), 117.6 (CH), 118.0 (CH), 121.3 (CH), 121.4 (C$_{quat}$), 126.2 (C$_{quat}$), 126.5 (CH), 126.7 (C$_{quat}$), 127.1 (CH), 129.0 (C$_{quat}$), 130.3 (CH), 131.6 (CH), 132.4 (CH), 133.9 (C$_{quat}$), 145.9 (C$_{quat}$), 146.8 (C$_{quat}$), 148.3 (C$_{quat}$), 151.1 (CH), 167.7 (C$_{quat}$), 193.5 (C$_{quat}$). MS (MALDI-TOF) calcd for $C_{46}H_{63}N_3OS_3$-H$^+$ *m/z:* 770.42; Found: 770.5. IR: $\tilde{\nu}$ [cm^{-1}] = 2920 (m), 2851 (w), 1715 (w), 1591 (w), 1568 (w), 1462 (m), 1422 (w), 1404 (w), 1348 (w), 1281 (s), 1252 (w), 1221 (w), 1192 (w), 1153 (w), 1125 (s), 1101 (s), 1057 (w), 991 (w), 959 (w), 899 (w), 870 (w), 804 (m), 731 (w), 720 (w), 692 (w), 673 (w), 610 (w). Anal calcd for $C_{46}H_{63}N_3OS_3$ [770.2]: C 71.73, H 8.24, N 5.46, S 12.49 gef.: C 71.58, H 8.05, N 5.32, S 12.59.

2.1.5.11. (*Z*)-5-{[10-(2-Decyltetradecyl)-7-(1-methyl-1*H*-pyrazol-4-yl)-10*H*-phenothiazin-3-yl]methylene}-3-methyl-2-thioxothiazolidin-4-one (12k)

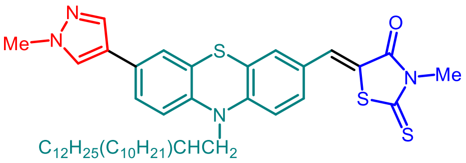

$C_{45}H_{64}N_4OS_3$ [773.21]

[62] Two CH$_3$ signals coincide.
[63] Two CH$_2$ signals coincide.
[64] Three CH$_3$ signals coincide.

According to the GP and after purification by chromatography on silica gel (*n*-hexane, *n*-hexane/acetone 15:1) and drying under vacuo compound **12k** (333 mg, 80%) was obtained as a dark red viscous oil. R_f (*n*-hexane/acetone 4:1) = 0.25.

^{1}H NMR (300 MHz, acetone-d$_6$/CS_2 4:1): δ 0.90 (t, ^3J = 6.7 Hz, 3 H), 0.91 (t, ^3J = 6.7 Hz, 3 H), 1.23–1.49 (m, 40 H), 1.98–2.04 (m, 1 H), 3.47 (s, 3 H), 3.88–3.95 (m, 5 H), 6.99 (d, ^3J = 8.4 Hz, 1 H), 7.08 (d, ^3J = 8.6 Hz, 1 H), 7.29 (d, ^4J = 2.1 Hz, 1 H), 7.31 (d, ^4J = 2.2 Hz, 1 H), 7.34 (dd, ^3J = 8.4 Hz, ^4J = 2.1 Hz, 1 H), 7.42 (dd, ^3J = 8.6 Hz, ^4J = 2.2 Hz, 1 H), 7.61 (s, 1 H), 7.65 (s, 1 H), 7.82 (s, 1 H). ^{13}C NMR (75 MHz, acetone-d$_6$/CS_2 4:1): δ 14.7 (CH_3),[65] 23.7 (CH_2),[66] 27.1 (CH_2),[66] 30.3 (CH_2),[66] 30.4 (CH_2),[66] 30.60 (CH_2),[67] 30.63 (CH_2), 30.64 (CH_2), 30.7 (CH_2), 31.0 (CH_2),[66] 31.5 (CH_3), 32.2 (CH_2),[66] 32.9 (CH_2),[66] 35.7 (CH), 39.2 (CH_3), 52.3 (CH_2), 117.1 (CH), 117.8 (CH), 120.9 (C_{quat}), 122.4 (C_{quat}), 124.9 (CH), 125.3 (CH), 125.7 (C_{quat}), 126.9 (C_{quat}), 127.4 (CH), 128.4 (C_{quat}), 129.7 (C_{quat}), 130.2 (CH), 131.5 (CH), 132.7 (CH), 136.5 (CH), 142.7 (C_{quat}), 148.9 (C_{quat}), 167.7 (C_{quat}), 193.1 (C_{quat}). MS (MALDI-TOF) calcd for $C_{45}H_{64}N_4OS_3$ *m/z:* 772.42; Found: 772.4 ([M]$^+$). ESI-HRMS calcd for $C_{45}H_{64}N_4OS_3$: 772.42422; Found: 772.42365 ([M]$^+$). IR: $\tilde{\nu}$ [cm^{-1}] = 2920 (m), 2851 (m), 1707 (m), 1600 (w), 1578 (m), 1561 (w), 1499 (w), 1474 (m), 1464 (m), 1424 (m), 1404 (m), 1350 (m), 1285 (s), 1254 (m), 1219 (m), 1169 (w), 1126 (s), 1099 (s), 1078 (w), 1057 (w), 1042 (w), 986 (w), 974 (m), 959 (w), 901 (w), 876 (w), 847 (w), 812 (m), 762 (w), 729 (w), 720 (w), 704 (w), 692 (w), 662 (w), 629 (w).

2.1.5.12. (*Z*)-3-[10-(2-Decyltetradecyl)-7-(1-methyl-1*H*-pyrazol-4-yl)-10*H*-phenothiazin-3-yl]-2-(4-nitrophenyl)acrylonitrile (12l)

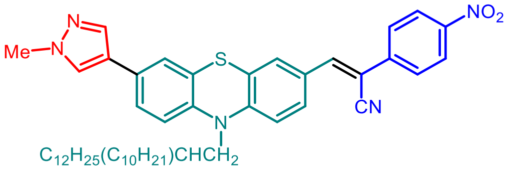

$C_{49}H_{65}N_5O_2S$ [788.14]

According to the GP and after purification by chromatography on silica gel (*n*-hexane, *n*-hexane/ethyl acetate 5:1) and drying under vacuo compound **12l** (288 mg, 71%) was obtained as a red brown amorphous solid, Mp 103–108 °C. R_f (*n*-hexane/ethyl acetate 5:1) = 0.13.

^{1}H NMR (300 MHz, acetone-d$_6$): δ 0.87 (t, ^3J = 6.7 Hz, 3 H), 0.88 (t, ^3J = 6.7 Hz, 3 H), 1.16–1.53 (m, 40 H), 1.98–2.03 (m, 1 H), 3.89 (s, 3 H), 3.92 (d, ^3J = 7.5 Hz, 2 H), 7.03 (d, ^3J = 8.4 Hz, 1 H), 7.12 (d, ^3J = 8.7 Hz, 1 H), 7.33 (d, ^4J = 2.0 Hz, 1 H), 7.38 (dd, ^3J = 8.5 Hz, ^4J = 2.0 Hz, 1 H), 7.71 (s, 1 H), 7.85 (d, ^4J = 2.1 Hz, 1 H), 7.88 (s, 1 H), 7.92–8.02 (m, 4 H), 8.31 (d, ^3J

[65] Two CH_3 signals coincide.
[66] Two CH_2 signals coincide.
[67] Three CH_2 signals coincide.

= 8.7 Hz, 2 H). ^{13}C NMR (150 MHz, acetone-d_6): δ 14.6 (CH_3),[68] 23.5 (CH_2),[69] 26.95 (CH_2), 26.96 (CH_2), 30.2 (CH_2),[69] 30.3 (CH_2),[69] 30.49 (CH_2),[70] 30.52 (CH_2), 30.54 (CH_2), 30.6 (CH_2), 30.8 (CH_2),[69] 32.1 (CH_2),[69] 32.8 (CH_2),[69] 35.6 (CH), 39.2 (CH_3), 52.2 (CH_2), 106.3 (C_{quat}), 116.8 (CH), 117.9 (CH), 118.4 (C_{quat}), 122.4 (C_{quat}), 124.9 (CH), 125.0 (CH), 125.3 (CH), 125.7 (C_{quat}), 126.1 (C_{quat}), 127.3 (CH), 127.6 (CH), 128.4 (C_{quat}), 129.6 (CH), 129.7 (C_{quat}), 130.8 (CH), 136.6 (CH), 142.0 (C_{quat}), 142.8 (C_{quat}), 145.0 (CH), 148.2 (C_{quat}), 149.6 (C_{quat}). MS (MALDI-TOF) calcd for $C_{49}H_{65}N_5O_2S$ *m/z:* 787.49; Found: 787.5 ($[M]^+$). IR: $\tilde{\nu}$ [cm^{-1}] = 2920 (m), 2851 (w), 2209 (w), 1603 (w), 1576 (m), 1560 (w), 1541 (w), 1516 (m), 1505 (m), 1475 (m), 1462 (m), 1402 (w), 1371 (w), 1335 (s), 1312 (m), 1254 (m), 1238 (m), 1209 (m), 1177 (w), 1109 (w), 1080 (w), 1038 (w), 1007 (w), 976 (w), 928 (w), 880 (w), 851 (m), 816 (m), 802 (m), 781 (w), 752 (w), 721 (w), 689 (w), 664 (w). Anal calcd for $C_{49}H_{65}N_5O_2S$ [788.1]: C 74.67, H 8.31, N 8.89; Found: C 74.55, H 8.22, N 8.74.

2.1.5.13. (*Z*)-4-{[10-(2-Decyltetradecyl)-7-(1-methyl-1H-pyrazol-4-yl)-10*H*-phenothiazin-3-yl]methylene}-3-methyl-1-phenyl-1H-pyrazol-5[4*H*]-one (12m)

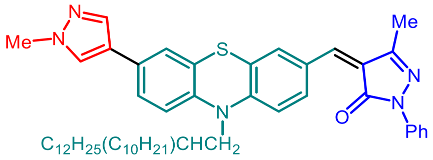

$C_{51}H_{69}N_5OS$ [800.19]

According to the GP and after purification by chromatography on silica gel (*n*-hexane, *n*-hexane/acetone 15:1) and drying under vacuo compound **12m** (288 mg, 69%) was obtained as a dark red viscous oil. R_f (*n*-hexane/acetone 10:1) = 0.10.

^{1}H NMR (300 MHz, acetone-d_6/CS_2 4:1): δ 0.89 (t, ^3J = 6.7 Hz, 3 H), 0.90 (t, ^3J = 6.7 Hz, 3 H), 1.23–1.49 (m, 40 H), 2.06–2.10 (m, 1 H), 2.34 (s, 3 H), 3.89 (s, 3 H), 3.94 (d, ^3J = 7.2 Hz, 2 H), 7.01 (d, ^3J = 8.4 Hz, 1 H), 7.07 (d, ^3J = 8.8 Hz, 1 H), 7.09–7.18 (m, 1 H), 7.28–7.43 (m, 4 H), 7.48 (s, 1 H), 7.66 (s, 1 H), 7.83 (s, 1 H), 8.03 (d, ^4J = 1.2 Hz, 1 H), 8.06 (d, ^4J = 1.4 Hz, 1 H), 8.48 (dd, ^3J = 8.7 Hz, ^4J = 2.1 Hz, 1 H), 8.68 (d, ^4J = 2.1 Hz, 1 H). ^{13}C NMR (75 MHz, acetone-d_6/CS_2 4:1): δ 13.8 (CH_3), 14.9 (CH_3),[71] 23.8 (CH_2),[72] 27.3 (CH_2),[72] 30.5 (CH_2),[72] 30.6 (CH_2),[72] 30.77 (CH_2),[73] 30.82 (CH_2), 30.83 (CH_2), 31.1 (CH_2),[72] 32.4 (CH_2),[72] 33.0 (CH_2),[72] 35.9 (CH), 39.3 (CH_3), 52.5 (CH_2), 116.2 (CH), 117.9 (CH), 119.0 (CH), 122.4 (C_{quat}), 124.8 (CH), 124.9 (CH), 125.2 (CH), 125.27 (C_{quat}), 125.31 (C_{quat}), 126.0 (C_{quat}), 127.5 (CH), 128.9 (C_{quat}), 129.3

[68] Two CH_3 signals coincide.
[69] Two CH_2 signals coincide.
[70] Three CH_2 signals coincide.
[71] Two CH_3 signals coincide.
[72] Two CH_2 signals coincide.
[73] Four CH_2 signals coincide.

(CH), 129.9 (C_{quat}), 133.7 (CH), 136.0 (CH), 136.5 (CH), 139.9 (C_{quat}), 142.3 (C_{quat}), 146.2 (CH), 151.0 (C_{quat}), 151.5 (C_{quat}), 162.8 (C_{quat}). MS (MALDI-TOF) calcd for $C_{51}H_{69}N_5OS\text{-}H^+$ *m/z:* 800.53; Found: 800.5. ESI-HRMS calcd for $C_{51}H_{69}N_5OS$: 799.52228; Found: 799.52225 ($[M]^+$). IR: $\tilde{\nu}$ [cm^{-1}] = 2922 (s), 2851 (m), 1678 (w), 1597 (w), 1578 (m), 1555 (m), 1537 (w), 1499 (m), 1474 (s), 1464 (s), 1416 (m), 1358 (m), 1317 (s), 1254 (m), 1215 (s), 1138 (s), 1105 (w), 1078 (w), 1065 (w), 1022 (w), 955 (m), 974 (m), 932 (w), 903 (w), 878 (w), 845 (w), 816 (m), 793 (w), 768 (m), 752 (m), 723 (w), 714 (w), 691 (m), 664 (m), 652 (w), 640 (w).

2.1.5.14. (*Z*)-5-{[10-(2-Decyltetradecyl)-7-(5-methylthiophen-2-yl)-10*H*-phenothiazin-3-yl]methylene}-3-methyl-2-thioxothiazolidin-4-one (12n)

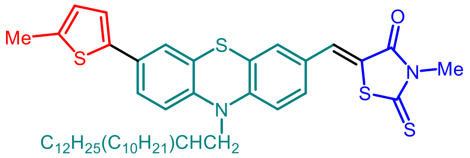

$C_{46}H_{64}N_2OS_4$ [789.27]

According to the GP and after purification by chromatography on silica gel (*n*-hexane, *n*-hexane/acetone 30:1) and drying under vacuo compound **12n** (322 mg, 85%) was obtained as a red resin. R_f (*n*-hexane/acetone 10:1) = 0.47.

^{1}H NMR (300 MHz, acetone-d_6/CS_2 4:1): δ 0.91 (t, ^3J = 6.7 Hz, 3 H), 0.92 (t, ^3J = 6.7 Hz, 3 H), 1.20–1.50 (m, 40 H), 1.99–2.04 (m, 1 H), 2.51 (s, 3 H), 3.47 (s, 3 H), 3.91 (d, ^3J = 7.2 Hz, 2 H), 6.73 (dd, ^3J = 3.6 Hz, ^5J = 1.2 Hz, 1 H), 7.01 (d, ^3J = 8.5 Hz, 1 H), 7.08 (d, ^3J = 3.5 Hz, 1 H), 7.11 (d, ^3J = 8.6 Hz, 1 H), 7.31–7.35 (m, 2 H), 7.37 (dd, ^3J = 8.4 Hz, ^4J = 2.2 Hz, 1 H), 7.44 (dd, ^3J = 8.6 Hz, ^4J = 2.2 Hz, 1 H), 7.62 (s, 1 H). ^{13}C NMR (75 MHz, acetone-d_6/CS_2 4:1): δ 14.8 (CH_3),[74] 15.7 (CH_3), 23.7 (CH_2),[75] 27.10 (CH_2), 27.12 (CH_2), 30.4 (CH_2),[75] 30.46 (CH_2), 30.48 (CH_2), 30.6 (CH_2),[76] 30.66 (CH_2), 30.68 (CH_2), 30.70 (CH_2), 30.97 (CH_2), 30.99 (CH_2), 31.5 (CH_3), 32.3 (CH_2),[75] 32.9 (CH_2),[75] 35.7 (CH), 52.4 (CH_2), 117.3 (CH), 117.8 (CH), 121.2 (C_{quat}), 123.5 (CH), 124.8 (CH), 125.4 (CH), 125.9 (C_{quat}), 126.7 (C_{quat}), 127.4 (CH), 128.6 (C_{quat}), 130.2 (CH), 131.2 (C_{quat}), 131.6 (CH), 132.5 (CH), 139.7 (C_{quat}), 141.3 (C_{quat}), 143.7 (C_{quat}), 148.6 (C_{quat}), 167.7 (C_{quat}), 193.2 (C_{quat}). MS (MALDI-TOF) calcd for $C_{46}H_{64}N_2OS_4$ *m/z:* 788.39; Found: 788.3 ($[M]^+$). IR: $\tilde{\nu}$ [cm^{-1}] = 2920 (m), 2851 (m), 1709 (m), 1591 (w), 1574 (m), 1505 (w), 1481 (m), 1458 (s), 1424 (m), 1402 (m), 1375 (w), 1348 (m), 1287 (s), 1265 (m), 1246 (m), 1221 (m), 1177 (w), 1165 (w), 1125 (s), 1101 (s), 1057 (w), 1040 (w), 991 (w), 959 (w), 937 (w), 897 (w), 864 (w), 814 (m), 795 (m), 758 (w), 729 (w), 720 (w), 691 (w), 662 (w), 646 (w),

[74] Two CH_3 signals coincide.
[75] Two CH_2 signals coincide.
[76] Three CH_2 signals coincide.

606 (w). Anal calcd for $C_{46}H_{64}N_2OS_4$ [789.3]: C 70.00, H 8.17, N 3.55, S 16.25; Found: C 70.06, H 8.12, N 3.26, S 15.96.

2.1.5.15. (*Z*)-5-{[10-(2-Decyltetradecyl)-7-(5-{4-[diethylamino]phenyl}thiophen-2-yl)-10*H*-phenothiazin-3-yl]methylene}-3-methyl-2-thioxothiazolidin-4-one (12o)

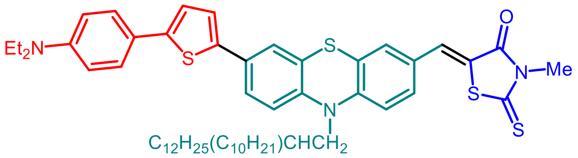

$C_{55}H_{75}N_3OS_4$ [922.46]

According to the GP and after purification by chromatography on silica gel (*n*-hexane/acetone 30:1) and drying under vacuo compound **12o** (355 mg, 67%) was obtained as a dark red amorphous solid, Mp 128–131 °C. R_f (*n*-hexane/acetone 10:1) = 0.43.

^{1}H NMR (300 MHz, acetone-d$_6$/CS_2 4:1): δ 0.91 (t, ^3J = 6.3 Hz, 6 H), 1.21 (t, ^3J = 7.0 Hz, 6 H), 1.23–1.55 (m, 40 H), 1.98–2.04 (m, 1 H), 3.44 (quart, ^3J = 7.0 Hz, 4 H), 3.47 (s, 3 H), 3.91 (d, ^3J = 7.0 Hz, 2 H), 6.68 (d, ^3J = 8.6 Hz, 2 H), 7.02 (d, ^3J = 8.4 Hz, 1 H), 7.08–7.14 (m, 2 H), 7.23 (d, ^3J = 3.8 Hz, 1 H), 7.32–7.47 (m, 6 H), 7.62 (s, 1 H). ^{13}C NMR (75 MHz, acetone-d$_6$/CS_2 4:1): δ 13.2 (CH_3), 14.8 (CH_3),[77] 23.7 (CH_2),[78] 27.10 (CH_2), 27.12 (CH_2), 30.4 (CH_2),[78] 30.5 (CH_2), 30.6 (CH_2),[79] 30.67 (CH_2),[78] 30.71 (CH_2), 30.97 (CH_2),[78] 31.5 (CH_3), 32.3 (CH_2),[78] 32.9 (CH_2),[78] 35.7 (CH), 45.1 (CH_2),[78] 52.4 (CH_2), 112.6 (CH), 117.3 (CH), 117.8 (CH), 121.1 (C_{quat}), 122.15 (CH), 122.19 (C_{quat}), 124.5 (CH), 124.7 (CH), 125.3 (CH), 125.9 (C_{quat}), 126.7 (C_{quat}), 127.5 (CH), 128.6 (C_{quat}), 130.3 (CH), 131.2 (C_{quat}), 131.6 (CH), 132.57 (CH), 139.9 (C_{quat}), 143.6 (C_{quat}), 145.4 (C_{quat}), 148.1 (C_{quat}), 148.6 (C_{quat}), 167.7 (C_{quat}), 193.3 (C_{quat}). MS (MALDI-TOF) calcd for $C_{55}H_{75}N_3OS_4$ *m/z:* 921.48; Found: 921.5 ($[M]^+$). IR: IR: $\tilde{\nu}$ [cm^{-1}] = 2955 (w), 2918 (m), 2849 (w), 1707 (m), 1607 (w), 1589 (w), 1574 (m), 1555 (w), 1541 (w), 1518 (w), 1466 (s), 1425 (w), 1404 (m), 1375 (w), 1352 (m), 1339 (m), 1294 (m), 1287 (s), 1265 (m), 1250 (m), 1221 (m), 1198 (m), 1175 (w), 1155 (w), 1121 (m), 1109 (m), 1078 (m), 1039 (w), 1018 (w), 990 (w), 959 (w), 937 (w), 895 (w), 872 (w), 812 (w), 791 (m), 756 (w), 725 (w), 714 (w), 608 (w). Anal calcd for $C_{55}H_{75}N_3OS_4$ [922.5]: C 71.61, H 8.19, N 4.56, S 13.90; Found: C 71.82, H 8.13, N 4.47, S 13.60.

---

[77] Two CH_3 signals coincide.
[78] Two CH_2 signals coincide.
[79] Three CH_2 signals coincide.

2.1.5.16. (*Z*)-5-{[7-(5-{9*H*-Carbazol-9-yl}thiophen-2-yl)-10-(2-decyltetradecyl)-10*H*-phenothiazin-3-yl]methylene}-3-methyl-2-thioxothiazolidin-4-one (12p)

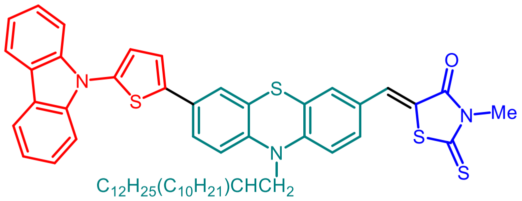

$C_{57}H_{69}N_3OS_4$ [940.44]

According to the GP and after purification by chromatography on silica gel (*n*-hexane/acetone 30:1) and drying under vacuo compound **12p** (391 mg, 74%) was obtained as an orange solid, Mp 95–98 °C. R_f (*n*-hexane/acetone 10:1) = 0.43.

^{1}H NMR (300 MHz, acetone-d$_6$/CS_2 4:1): δ 0.86–0.94 (m, 6 H), 1.21–1.52 (m, 40 H), 2.06–2.09 (m, 1 H), 3.47 (s, 3 H), 3.94 (d, ^3J = 7.1 Hz, 2 H), 7.09 (d, ^3J = 8.3 Hz, 1 H), 7.14 (d, ^3J = 8.6 Hz, 1 H), 7.26 (d, ^3J = 4.0 Hz, 1 H), 7.27–7.33 (m, 2 H), 7.35 (d, ^4J = 2.1 Hz, 1 H), 7.42–7.56 (m, 8 H), 7.62 (s, 1 H), 8.13 (dt, ^3J = 7.8 Hz, ^4J = 0.9 Hz, 2 H). ^{13}C NMR (75 MHz, acetone-d$_6$/CS_2 4:1): δ 14.76 (CH_3), 14.77 (CH_3), 23.7 (CH_2),[80] 27.1 (CH_2),[80] 30.4 (CH_2), 30.5 (CH_2),[80] 30.6 (CH_2),[81] 30.65 (CH_2), 30.67 (CH_2), 30.69 (CH_2), 31.0 (CH_2),[80] 31.5 (CH_3), 32.3 (CH_2),[80] 32.9 (CH_2), 35.7 (CH), 52.4 (CH_2), 111.0 (CH), 117.4 (CH), 117.9 (CH), 121.1 (CH), 121.3 (C_{quat}), 121.7 (CH), 122.6 (CH), 124.4 (C_{quat}), 125.2 (CH), 125.9 (CH), 126.3 (C_{quat}), 126.6 (C_{quat}), 126.8 (CH), 127.2 (CH), 128.8 (C_{quat}), 130.29 (C_{quat}), 130.31 (C_{quat}), 131.6 (CH), 132.5 (CH), 137.9 (C_{quat}), 142.0 (C_{quat}), 142.4 (C_{quat}), 144.6 (C_{quat}), 148.4 (C_{quat}), 167.7 (C_{quat}), 193.4 (C_{quat}). MS (MALDI-TOF) calcd for $C_{57}H_{69}N_3OS_4$ *m/z:* 939.43; Found: 939.5 ([M]$^+$). IR: $\tilde{\nu}$ [cm^{-1}] = 3059 (w), 2953 (w), 2920 (m), 2851 (m), 1705 (m), 1595 (w), 1578 (m), 1557 (w), 1508 (w), 1481 (m), 1451 (m), 1443 (m), 1425 (w), 1404 (m), 1375 (w), 1335 (m), 1319 (m), 1281 (s), 1256 (m), 1223 (m), 1173 (w), 1161 (w), 1125 (m), 1105 (s), 1044 (w), 1018 (w), 990 (w), 959 (w), 926 (w), 891 (w), 870 (w), 851 (w), 804 (w), 797 (m), 748 (s), 740 (m), 719 (m), 714 (m), 692 (w), 667 (w), 652 (w), 637 (w), 617 (w). Anal calcd for $C_{57}H_{69}N_3OS_4$ [940.4]: C 72.80, H 7.40, N 4.47; Found: C 72.87, H 7.39, N 4.34.

2.1.5.17. (*Z*)-5-{[10-(2-Decyltetradecyl)-7-(4-{diphenylamino}phenyl)-10*H*-phenothiazin-3-yl]methylene}-3-methyl-2-thioxothiazolidin-4-one (12q)

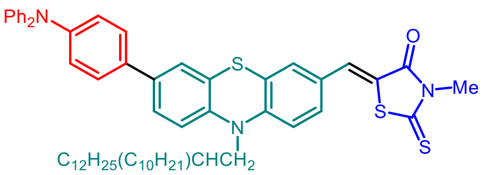

[80] Two CH_2 signals coincide.
[81] Three CH_2 signals coincide.

$C_{59}H_{73}N_3OS_3$ [936.43]

According to the GP and after purification by chromatography on silica gel (*n*-hexane, *n*-hexane/acetone 30:1) and drying under vacuo compound **12q** (383 mg, 84%) was obtained as a dark red resin. R_f (*n*-hexane/acetone 5:1) = 0.51.

^{1}H NMR (300 MHz, acetone-d$_6$/CS$_2$ 4:1): δ 0.86–0.92 (m, 6 H), 1.23–1.51 (m, 40 H), 2.06–2.09 (m, 1 H), 3.47 (s, 3 H), 3.94 (d, ^3J = 7.0 Hz, 2 H), 7.01–7.16 (m, 10 H), 7.25–7.36 (m, 5 H), 7.40 (d, ^4J = 2.1 Hz, 1 H), 7.46 (dt, ^3J = 8.3 Hz, ^4J = 2.2 Hz, 2 H), 7.49–7.55 (m, 2 H), 7.63 (s, 1 H). ^{13}C NMR (75 MHz, acetone-d$_6$/CS$_2$ 4:1): δ 14.6 (CH$_3$),[82] 23.4 (CH$_2$),[83] 26.97 (CH$_2$), 26.99 (CH$_2$), 30.27 (CH$_2$),[83] 30.33 (CH$_2$), 30.4 (CH$_2$), 30.52 (CH$_2$), 30.54 (CH$_2$),[83] 30.57 (CH$_2$),[83] 30.61 (CH$_2$), 30.82 (CH$_2$), 30.84 (CH$_2$), 31.5 (CH$_3$), 32.2 (CH$_2$),[83] 32.8 (CH$_2$),[83] 35.6 (CH), 52.3 (CH$_2$), 117.4 (CH), 118.0 (CH), 121.1 (C$_{quat}$), 124.0 (CH), 124.5 (CH), 125.2 (CH), 125.8 (C$_{quat}$), 126.0 (CH), 126.5 (CH), 126.9 (C$_{quat}$), 128.0 (2 CH), 128.6 (C$_{quat}$), 130.2 (CH), 130.3 (CH), 131.6 (CH), 132.7 (CH), 134.3 (C$_{quat}$), 136.7 (C$_{quat}$), 143.8 (C$_{quat}$), 147.9 (C$_{quat}$), 148.4 (C$_{quat}$), 148.8 (C$_{quat}$), 167.9 (C$_{quat}$), 193.8 (C$_{quat}$). MS (MALDI-TOF) calcd for $C_{59}H_{73}N_3OS_3$ *m/z:* 935.49; Found: 935.5 ([M]$^+$).

2.1.5.18. (*Z*)-3-{10-(2-Decyltetradecyl)-7-[4-(diphenylamino)phenyl]-10*H*-phenothiazin-3-yl}-2-(4-nitrophenyl)acrylonitrile (12r)

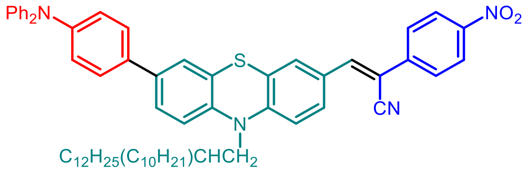

$C_{63}H_{74}N_4O_2S$ [951.35]

According to the GP and after purification by chromatography on silica gel (*n*-hexane, *n*-hexane/acetone 15:1) and drying under vacuo compound **12r** (394 mg, 82%) was obtained as a black amorphous solid, Mp 38–42 °C. R_f (*n*-hexane/acetone 10:1) = 0.23.

^{1}H NMR (600 MHz, acetone-d$_6$): δ 0.83 (t, ^3J = 7.0 Hz, 3 H), 0.84 (t, ^3J = 7.0 Hz, 3 H), 1.14–1.50 (m, 40 H), 1.99–2.04 (m, 1 H), 3.91 (d, ^3J = 7.1 Hz, 2 H), 7.02–7.10 (m, 9 H), 7.12 (d, ^3J = 8.7 Hz, 1 H), 7.26–7.31 (m, 4 H), 7.38 (d, ^4J = 2.1 Hz, 1 H), 7.47 (dd, ^3J = 8.5 Hz, ^4J = 2.2 Hz, 1 H), 7.50–7.54 (m, 2 H), 7.82 (d, ^3J = 7.2 Hz, 1 H), 7.94 (dd, ^3J = 8.8 Hz, ^4J = 2.2 Hz, 1 H), 7.95–7.99 (m, 3 H), 8.29–8.33 (m, 2 H). ^{13}C NMR (150 MHz, acetone-d$_6$): δ 14.41 (CH$_3$), 14.42 (CH$_3$), 23.34 (CH$_2$), 23.35 (CH$_2$), 26.7 (CH$_2$), 26.8 (CH$_2$), 30.12 (CH$_2$),[84] 30.13 (CH$_2$), 30.2 (CH$_2$), 30.35 (CH$_2$), 30.37 (CH$_2$), 30.38 (CH$_2$), 30.41 (CH$_2$), 30.43 (CH$_2$), 30.5 (CH$_2$), 30.60 (CH$_2$), 30.63 (CH$_2$), 31.9 (CH$_2$), 32.0 (CH$_2$), 32.65 (CH$_2$), 32.66 (CH$_2$), 35.5 (CH), 52.2 (CH$_2$),

[82] Two CH$_3$ signals coincide.
[83] Two CH$_2$ signals coincide.
[84] Two CH$_2$ signals coincide.

106.4 (C_{quat}), 177.0 (CH), 118.0 (CH), 118.5 (C_{quat}), 124.0 (CH), 124.5 (CH), 125.1 (CH), 125.2 (CH), 125.7 (C_{quat}), 125.8 (CH), 126.0 (C_{quat}), 126.4 (CH), 127.3 (CH), 127.9 (CH), 128.6 (C_{quat}), 129.5 (CH), 130.3 (CH), 130.9 (CH), 134.2 (C_{quat}), 136.5 (C_{quat}), 142.0 (C_{quat}), 143.8 (C_{quat}), 145.1 (CH), 148.0 (C_{quat}), 148.3 (C_{quat}), 148.5 (C_{quat}), 149.5 (C_{quat}). MS (MALDI-TOF) calcd for $C_{63}H_{74}N_4O_2S$ *m/z:* 950.55; Found: 950.6 ($[M]^+$). IR: $\tilde{\nu}$ [cm^{-1}] = 2953 (w), 2922 (m), 2851 (w), 1591 (w), 1572 (m), 1518 (m), 1493 (m), 1462 (s), 1402 (w), 1337 (s), 1316 (m), 1277 (m), 1271 (m), 1252 (m), 1206 (m), 1175 (w), 1155 (w), 1109 (w), 1074 (w), 1028 (w), 1001 (w), 914 (w), 880 (m), 851 (m), 812 (m), 752 (m), 723 (w), 694 (s), 667 (w). Anal calcd for $C_{63}H_{74}N_4O_2S$ [951.4]: C 79.54, H 7.84, N 5.89; Found: C 79.81, H 7.84, N 5.83.

2.1.5.19. (*Z*)-5-{[7-(4-{Bis[4-methoxyphenyl]amino}phenyl)-10-(2-decyltetradecyl)-10*H*-phenothiazin-3-yl]methylene}-3-methyl-2-thioxothiazolidin-4-one (12s)

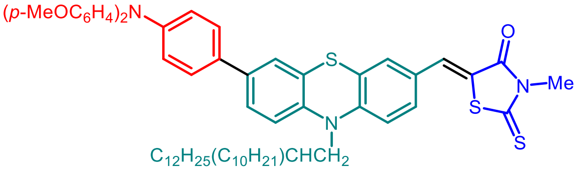

$C_{61}H_{77}N_3O_3S_3$ [996.48]

According to the GP and after purification by chromatography on silica gel (*n*-hexane, *n*-hexane/acetone 20:1) and drying under vacuo compound **12s** (417 mg, 83%) was obtained as a dark red resin. R_f (*n*-hexane/acetone 10:1) = 0.14.

^1H NMR (300 MHz, acetone-d$_6$): δ 0.85 (t, ^3J = 6.7 Hz, 3 H), 0.86 (t, ^3J = 6.7 Hz, 3 H), 1.15–1.44 (m, 40 H), 1.95–2.04 (m, 1 H), 3.44 (s, 3 H), 3.78 (s, 6 H), 3.86 (d, ^3J = 7.1 Hz, 2 H), 6.85–6.93 (m, 6 H), 7.00–7.09 (m, 6 H), 7.25 (d, ^4J = 2.1 Hz, 1 H), 7.35 (d, ^4J = 2.1 Hz, 1 H), 7.38 (dd, ^3J = 8.7 Hz, ^4J = 2.2 Hz, 1 H), 7.40–7.46 (m, 3 H), 7.58 (s, 1 H). ^{13}C NMR (75 MHz, acetone-d$_6$): δ 14.5 (CH_3),[85] 23.4 (CH_2),[86] 26.7 (CH_2), 26.8 (CH_2), 30.2 (CH_2),[87] 30.37 (CH_2), 30.40 (CH_2), 30.42 (CH_2), 30.45 (CH_2),[86] 30.49 (CH_2), 31.5 (CH_3), 30.60 (CH_2), 30.64 (CH_2), 31.96 (CH_2), 31.98 (CH_2), 32.7 (CH_2),[86] 35.4 (CH), 52.2 (CH_2), 55.7 (CH_3), 115.6 (CH, C_{quat}),[88] 117.4 (CH), 118.0 (CH), 120.9 (C_{quat}), 121.0 (CH), 125.57 (C_{quat}), 125.61 (CH), 126.1 (CH), 126.7 (C_{quat}), 127.6 (CH, C_{quat}),[89] 128.4 (C_{quat}), 130.3 (CH), 131.5 (CH), 132.7 (CH), 136.9 (C_{quat}), 141.5 (C_{quat}), 143.4 (C_{quat}), 148.8 (C_{quat}), 157.2 (C_{quat}), 168.0 (C_{quat}), 194.0 (C_{quat}). MS (MALDI-TOF) calcd for $C_{61}H_{77}N_3O_3S_3$ *m/z:* 995.51; Found: 995.5 ($[M]^+$). ESI-HRMS calcd for $C_{61}H_{77}N_3O_3S_3$: 995.51271; Found: 995.51226 ($[M]^+$). IR: $\tilde{\nu}$ [cm^{-1}] = 2920 (m), 2851 (w), 1732 (w), 1709 (w), 1595 (w), 1574 (m), 1503 (s), 1460 (s), 1439 (w), 1423 (w), 1404 (w), 1346 (w),

[85] Two CH_3 signals coincide.
[86] Two CH_2 signals coincide.
[87] Four CH_3 signals coincide.
[88] A CH and a quaternary signal coincide.
[89] Two CH and a quaternary signal coincide.

1317 (w), 1285 (s), 1238 (s), 1196 (w), 1179 (w), 1165 (w), 1126 (m), 1099 (m), 1036 (m), 990 (w), 959 (w), 901 (w), 883 (w), 826 (m), 812 (m), 766 (w), 718 (w), 694 (w), 660 (w), 638 (w). UV/VIS (CH_2Cl_2) λ_{max} (ε 10^3 [$M^{-1}cm^{-1}$]) [nm] = 303 (38), 345 (41), 489 (22).

2.1.5.20. (*Z*)-5-{[7-(4-{Bis[4-methoxyphenyl]amino}phenyl)-10-(2-decyltetradecyl)-10*H*-phenothiazin-3-yl]methylene}-2-thioxothiazolidin-4-one (12t)

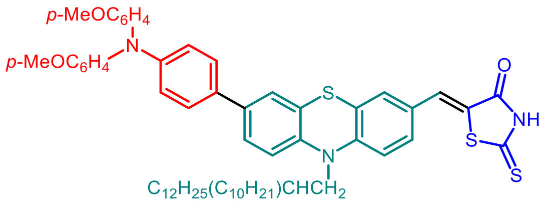

$C_{60}H_{75}N_3O_3S_3$ [982.45]

According to the GP and after purification by chromatography on silica gel (*n*-hexane, *n*-hexane/acetone 30:1) and drying under vacuo compound **12t** (400 mg, 79%) was obtained as a black amorphous solid, Mp softening >58 °C, melting >71 °C. R_f (*n*-hexane/ethyl acetate 5:1) = 0.26.

^{1}H NMR (300 MHz, acetone-d$_6$/CS_2 4:1): δ 0.90 (t, ^3J = 6.4 Hz, 6 H), 1.15–1.54 (m, 40 H), 1.99–2.04 (m, 1 H), 3.79 (s, 6 H), 3.90 (d, ^3J = 6.7 Hz, 2 H), 6.81–6.96 (m, 6 H), 6.98–7.15 (m, 6 H), 7.27–7.43 (m, 6 H), 7.46 (s, 1 H), 12.00 (br, 1 H). ^{13}C NMR (75 MHz, acetone-d$_6$/CS_2 4:1): δ 14.8 (CH_3),[90] 23.7 (CH_2),[91] 27.1 (CH_2),[91] 30.39 (CH_2), 30.41 (CH_2), 30.49 (CH_2), 30.51 (CH_2), 30.7 (CH_2),[92] 31.0 (CH_2),[91] 32.25 (CH_2), 32.28 (CH_2), 32.9 (CH_2),[91] 35.7 (CH), 52.3 (CH_2), 55.7 (CH_3), 115.5 (CH), 117.1 (CH), 117.7 (CH), 121.2 (CH), 123.7 (C_{quat}), 125.8 (C_{quat}), 125.8 (CH), 126.1 (CH), 126.9 (C_{quat}), 127.4 (CH), 127.6 (CH), 128.4 (C_{quat}), 130.1 (CH), 131.3 (CH), 131.7 (CH), 132.0 (C_{quat}), 136.9 (C_{quat}), 141.3 (C_{quat}), 143.3 (C_{quat}), 148.6 (C_{quat}), 148.8 (C_{quat}), 156.9 (C_{quat}), 169.1 (C_{quat}), 194.5 (C_{quat}). MS (MALDI-TOF) calcd for $C_{60}H_{75}N_3O_3S_3$ *m/z:* 981.50; Found: 981.5 ([M]$^+$). ESI-HRMS calcd for $C_{60}H_{75}N_3O_3S_3$: 981.4971; Found: 981.4968 ([M]$^+$). IR: IR: $\tilde{\nu}$ [cm^{-1}] = 2988 (w), 2970 (w), 2920 (m), 2851 (w), 1715 (w), 1697 (w), 1593 (w), 1572 (m), 1502 (m), 1460 (s), 1439 (m), 1404 (m), 1319 (w), 1275 (m), 1238 (s), 1175 (s), 1169 (s), 1105 (m), 1052 (m), 1038 (m), 903 (w), 872 (w), 824 (m), 808 (m), 781 (w), 718 (w), 692 (w), 669 (m), 645 (w), 631 (w).

[90] Two CH_3 signals coincide.
[91] Two CH_2 signals coincide.
[92] Six CH_2 signals coincide.

2.1.5.21. (*Z*)-3-{7-[4-(Bis{4-methoxyphenyl}amino)phenyl]-10-(2-decyltetradecyl)-10*H*-phenothiazin-3-yl}-2-(4-nitrophenyl)acrylonitrile (12u)

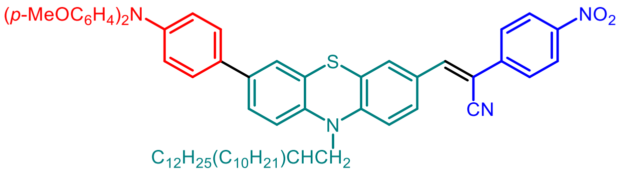

$C_{65}H_{78}N_4O_4S$ [1011.40]

According to the GP and after purification by chromatography on silica gel (*n*-hexane, *n*-hexane/ethyl acetate 25:1) and drying under vacuo compound **12u** (412 mg, 81%) was obtained as a dark red amorphous solid, Mp softening >43 °C, melting >54 °C. R_f (*n*-hexane/ethyl acetate 5:1) = 0.49.

^{1}H NMR (600 MHz, acetone-d$_6$): δ 0.83 (t, ^3J = 6.8 Hz, 3 H), 0.84 (t, ^3J = 6.8 Hz, 3 H), 1.13–1.49 (m, 40 H), 1.98–2.05 (m, 1 H), 3.77 (s, 6 H), 3.88 (d, ^3J = 7.0 Hz, 2 H), 6.87–6.91 (m, 6 H), 7.02–7.09 (m, 6 H), 7.34 (d, ^4J = 2.2 Hz, 1 H), 7.39–7.47 (m, 3 H), 7.80 (d, ^4J = 2.1 Hz, 1 H), 7.90–8.00 (m, 4 H), 8.28–8.34 (m, 2 H). ^{13}C NMR (150 MHz, acetone-d$_6$): δ 14.43 (CH_3), 14.44 (CH_3), 23.4 (CH_2),[93] 26.75 (CH_2), 26.79 (CH_2), 30.13 (CH_2),[94] 30.2 (CH_2), 30.37 (CH_2), 30.39 (CH_2), 30.40 (CH_2), 30.43 (CH_2), 30.44 (CH_2), 30.5 (CH_2), 30.6 (CH_2), 30.7 (CH_2), 31.9 (CH_2), 32.0 (CH_2), 32.7 (CH_2),[93] 35.5 (CH), 52.2 (CH_2), 55.7 (CH_3), 106.4 (C_{quat}), 115.6 (CH), 117.0 (CH), 118.0 (CH), 118.5 (C_{quat}), 121.0 (CH), 125.0 (CH), 125.6 (CH, C_{quat}),[95] 126.0 (C_{quat}), 126.1 (CH), 127.3 (CH), 127.6 (CH),[96] 128.5 (C_{quat}), 129.5 (CH), 130.8 (CH), 131.8 (C_{quat}), 136.9 (C_{quat}), 141.5 (C_{quat}), 142.0 (C_{quat}), 143.4 (C_{quat}), 145.0 (CH), 148.3 (C_{quat}), 149.1 (C_{quat}), 149.5 (C_{quat}), 157.2 (C_{quat}). MS (MALDI-TOF) calcd for $C_{65}H_{78}N_4O_4S$ *m/z:* 1010.57; Found: 1010.6 ([M]$^+$). IR: $\tilde{\nu}$ [cm^{-1}] = 2922 (m), 2851 (w), 2212 (w), 1601 (w), 1572 (m), 1505 (s), 1462 (s), 1439 (m), 1402 (w), 1339 (s), 1319 (m), 1285 (m), 1236 (s), 1206 (m), 1179 (m), 1109 (m), 1036 (m), 1009 (w), 955 (w), 912 (w), 880 (w), 851 (m), 820 (m), 814 (m), 781 (w), 752 (w), 729 (w), 692 (w), 637 (w), 623 (w). Anal calcd for $C_{65}H_{78}N_4O_4S$ [1011]: C 77.19, H 7.77, N 5.54, S 3.17; Found: C 77.07, H 7.67, N 5.50, S 2.99.

---

93 Two CH_2 signals coincide.
94 Three CH_2 signals coincide.
95 A CH and a quaternary signal coincide.
96 Two CH signals coincide.

2.1.5.22. (*Z*)-4-{[7-(4-(Bis{4-methoxyphenyl}amino)phenyl]-10-(2-decyltetradecyl)-10*H*-phenothiazin-3-yl]methylene}-3-methyl-1-phenyl-1H-pyrazol-5[4*H*]-one (12v)

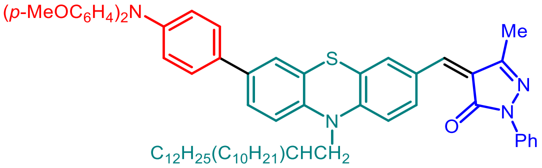

$C_{67}H_{82}N_4O_3S$ [1023.46]

According to the GP and after purification by chromatography on silica gel (*n*-hexane, *n*-hexane/acetone 20:1) and drying under vacuo compound **12v** (376 mg, 72%) was obtained as a dark red resin. R_f (*n*-hexane/acetone 10:1) = 0.20.

^{1}H NMR (300 MHz, acetone-d$_6$/CS$_2$ 4:1): δ 0.82–0.94 (m, 6 H), 1.23–1.51 (m, 40 H), 2.07–2.12 (m, 1 H), 2.32 (s, 3 H), 3.75 (s, 6 H), 3.96 (d, ^3J = 7.2 Hz, 2 H), 6.85–6.95 (m, 6 H), 7.02–7.18 (m, 7 H), 7.34–7.46 (m, 6 H), 7.50 (s, 1 H), 8.03–8.08 (m, 2 H), 8.47 (dd, ^3J = 8.8 Hz, ^4J = 2.1 Hz, 1 H), 8.73 (d, ^4J = 2.0 Hz, 1 H). ^{13}C NMR (75 MHz, acetone-d$_6$/CS$_2$ 4:1): δ 13.6 (CH$_3$), 14.6 (CH$_3$),[97] 23.6 (CH$_2$),[98] 27.00 (CH$_2$), 27.01 (CH$_2$), 30.3 (CH$_2$),[98] 30.36 (CH$_2$), 30.37 (CH$_2$), 30.55 (CH$_2$),[99] 30.57 (CH$_2$), 30.59 (CH$_2$), 30.62 (CH$_2$), 30.9 (CH$_2$),[98] 32.1 (CH$_2$),[98] 32.8 (CH$_2$),[98] 35.7 (CH), 52.4 (CH$_2$), 55.7 (CH$_3$), 115.6 (CH), 116.4 (CH), 118.1 (CH), 119.0 (CH), 121.2 (CH), 124.8 (CH), 125.3 (C$_{quat}$), 125.4 (C$_{quat}$), 125.7 (CH), 126.0 (C$_{quat}$), 126.1 (CH), 127.5 (CH), 127.7 (CH), 129.0 (C$_{quat}$), 129.3 (CH), 131.9 (C$_{quat}$), 133.7 (CH), 136.1 (CH), 137.2 (C$_{quat}$), 140.0 (C$_{quat}$), 141.4 (C$_{quat}$), 143.0 (C$_{quat}$), 146.4 (CH), 149.0 (C$_{quat}$), 151.0 (C$_{quat}$), 151.7 (C$_{quat}$), 157.1 (C$_{quat}$), 162.9 (C$_{quat}$). MS (MALDI-TOF) calcd for $C_{67}H_{82}N_4O_3S$ *m/z:* 1022.61; Found: 1022.6 ([M]$^+$). ESI-HRMS calcd for $C_{67}H_{82}N_4O_3S$: 1022.61076; Found: 1022.61107 ([M]$^+$). IR: $\tilde{\nu}$ [cm^{-1}] = 3059 (w), 2922 (m), 2851 (w), 2359 (w), 1676 (w), 1613 (w), 1595 (w), 1572 (w), 1559 (w), 1501 (s), 1458 (s), 1441 (w), 1420 (w), 1395 (w), 1317 (m), 1285 (w), 1238 (s), 1215 (s), 1177 (m), 1163 (m), 1138 (m), 1105 (w), 1036 (m), 995 (w), 930 (w), 878 (w), 826 (m), 814 (m), 779 (w), 768 (w), 752 (w), 721 (w), 664 (w), 635 (w).

[97] Two CH_3 signals coincide.
[98] Two CH_2 signals coincide.
[99] Three CH_2 signals coincide.

2.1.5.23. (*Z*)-5-{[10-(2-Decyltetradecyl)-10'-hexyl-7'-(*p*-tolyl)-10*H*,10'*H*-(3,3'-biphenothiazin)-7-yl]methylene}-3-methyl-2-thioxothiazolidin-4-one (12w)

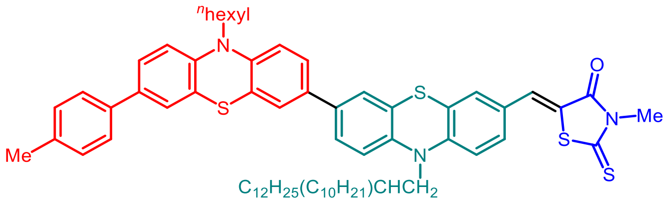

$C_{66}H_{85}N_3OS_4$ [1064.66]

According to the GP and after purification by chromatography on silica gel (*n*-hexane/acetone 30:1) and drying under vacuo compound **12w** (446 mg, 80%) was obtained as a black resin. R_f (*n*-hexane/acetone 10:1) = 0.45.

^{1}H NMR (300 MHz, acetone-d$_6$/CS$_2$ 4:1): δ 0.85–0.97 (m, 9 H), 1.19–1.60 (m, 46 H), 1.87 (quin, ^3J = 7.7 Hz, 2 H), 1.99–2.04 (m, 1 H), 2.37 (s, 3 H), 3.47 (s, 3 H), 3.92 (d, ^3J = 7.4 Hz, 2 H), 3.96 (t, ^3J = 7.1 Hz, 2 H), 6.98 (d, ^4J = 2.1 Hz, 1 H), 7.01 (d, ^3J = 2.0 Hz, 1 H), 7.06 (d, ^3J = 8.5 Hz, 1 H), 7.11 (d, ^3J = 8.6 Hz, 1 H), 7.21 (d, ^3J = 7.9 Hz, 2 H), 7.30–7.48 (m, 10 H), 7.61 (s, 1 H). ^{13}C NMR (75 MHz, acetone-d$_6$/CS$_2$ 4:1): δ 14.7 (CH$_3$), 14.8 (CH$_3$),[100] 21.4 (CH$_3$), 23.65 (CH$_2$), 23.67 (CH$_2$),[101] 27.1 (CH$_2$),[101] 27.5 (CH$_2$), 27.7 (CH$_2$), 30.4 (CH$_2$),[101] 30.5 (CH$_2$),[101] 30.6 (CH$_2$),[102] 30.67 (CH$_2$), 30.69 (CH$_2$), 31.0 (CH$_2$),[101] 31.5 (CH$_3$), 32.3 (CH$_2$),[101] 32.5 (CH$_2$), 32.9 (CH$_2$),[101] 35.7 (CH), 48.1 (CH$_2$), 52.4 (CH$_2$), 116.49 (CH), 116.51 (CH), 117.2 (CH), 117.8 (CH), 121.1 (C$_{quat}$), 125.4 (C$_{quat}$), 125.65 (CH), 125.66 (C$_{quat}$), 125.8 (C$_{quat}$), 125.9 (CH), 126.0 (CH), 126.1 (CH), 126.3 (CH), 126.5 (CH), 126.9 (C$_{quat}$), 127.0 (CH), 128.6 (C$_{quat}$), 130.2 (CH), 130.3 (CH), 131.6 (CH), 132.6 (CH), 134.5 (C$_{quat}$), 136.0 (C$_{quat}$), 136.1 (C$_{quat}$), 137.2 (C$_{quat}$), 137.7 (C$_{quat}$), 143.8 (C$_{quat}$), 144.6 (C$_{quat}$), 144.9 (C$_{quat}$), 148.7 (C$_{quat}$), 167.7 (C$_{quat}$), 193.3 (C$_{quat}$). MS (MALDI-TOF) calcd for $C_{66}H_{85}N_3OS_4$ *m/z:* 1063.56; Found: 1063.5 ([M]$^+$). IR: $\tilde{\nu}$ [cm^{-1}] = 2953 (w), 2920 (m), 2851 (m), 1711 (m), 1595 (w), 1574 (m), 1456 (s), 1418 (w), 1404 (w), 1377 (w), 1339 (m), 1287 (m), 1252 (m), 1221 (m), 1198 (w), 1126 (m), 1099 (m), 1063 (w), 1040 (w), 990 (w), 959 (w), 901 (w), 874 (w), 804 (m), 721 (w). Anal calcd for $C_{66}H_{85}N_3OS_4$ [1065]: C 74.46, H 8.05, N 3.95, S 12.05; Found: C 74.55, H 8.04, N 3.83, S 12.03.

[100] Two CH$_3$ signals coincide.
[101] Two CH$_2$ signals coincide.
[102] Four CH$_2$ signals coincide.

2.1.5.24. (*Z*)-5-{[10-(2-Decyltetradecyl)-10'-hexyl-7'-(*p*-tolyl)-10*H*,10'*H*-(3,3'-biphenothiazin)-7-yl]methylene}-2-thioxothiazolidin-4-one (12x)

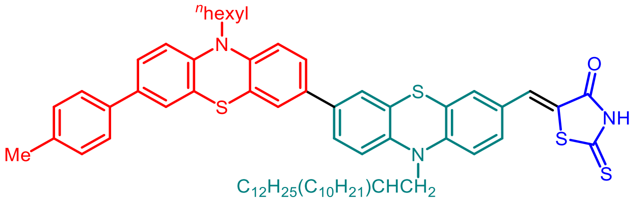

$C_{65}H_{83}N_3OS_4$ [1050.63]

According to the GP and after purification by chromatography on silica gel (*n*-hexane, *n*-hexane/acetone 30:1) and drying under vacuo compound **12x** (520 mg, 89%) was obtained as a black amorphous solid, Mp 57–63 °C. R_f (*n*-hexane/acetone 1:1) = 0.76.

^{1}H NMR (300 MHz, acetone-d$_6$/CS$_2$ 4:1): δ 0.86–0.95 (m, 9 H), 1.21–1.58 (m, 46 H), 1.87 (quin, ^3J = 7.5 Hz, 2 H), 1.99–2.04 (m, 1 H), 2.37 (s, 3 H), 3.92 (d, ^3J = 7.1 Hz, 2 H), 3.96 (t, ^3J = 7.1 Hz, 2 H), 6.98 (d, ^4J = 1.7 Hz, 1 H), 7.01 (d, ^4J = 1.7 Hz, 1 H), 7.06 (d, ^3J = 8.5 Hz, 1 H), 7.11 (d, ^3J = 8.7 Hz, 1 H), 7.18–7.24 (m, 2 H), 7.30–7.47 (m, 10 H), 7.48 (s, 1 H), 12.11 (br, 1 H). ^{13}C NMR (75 MHz, acetone-d$_6$/CS$_2$ 4:1): δ 14.7 (CH_3), 14.8 (CH_3),[103] 21.4 (CH_3), 23.66 (CH_2), 23.67 (CH_2),[104] 27.1 (CH_2),[104] 27.5 (CH_2), 27.7 (CH_2), 30.4 (CH_2),[104] 30.5 (CH_2),[104] 30.63 (CH_2), 30.64 (CH_2), 30.66 (CH_2),[105] 30.69 (CH_2), 31.0 (CH_2),[104] 32.3 (CH_2),[104] 32.5 (CH_2), 32.9 (CH_2),[104] 35.7 (CH), 48.1 (CH_2), 52.3 (CH_2), 116.5 (CH),[106] 117.2 (CH), 117.8 (CH), 123.8 (C_{quat}), 125.4 (C_{quat}), 125.7 (CH), 125.7 (C_{quat}), 125.9 (C_{quat}, CH),[107] 126.9 (CH), 126.1 (CH), 126.3 (CH), 126.5 (CH), 126.9 (C_{quat}), 127.0 (CH), 128.5 (C_{quat}), 130.1 (CH), 130.3 (CH), 131.4 (CH), 131.7 (CH), 134.5 (C_{quat}), 136.0 (C_{quat}), 136.1 (C_{quat}), 137.2 (C_{quat}), 137.7 (C_{quat}), 143.8 (C_{quat}), 144.6 (C_{quat}), 144.9 (C_{quat}), 148.6 (C_{quat}), 169.1 (C_{quat}), 194.6 (C_{quat}). MS (MALDI-TOF) calcd for $C_{65}H_{83}N_3OS_4$ *m/z:* 1049.54; Found: 1049.6 ([M]$^+$). IR: $\tilde{\nu}$ [cm^{-1}] = 2951 (w), 2920 (m), 2851 (w), 1690 (w), 1591 (w), 1570 (m) 1543 (w), 1458 (s), 1402 A(m), 1375 (w), 1335 (w), 1275 (w), 1250 (m), 1231 (m), 1182 (s), 1169 (s), 1105 (w), 1065 (w), 1038 (w), 1017 (w), 939 (w), 903 (w), 874 (w), 802 (m), 724 (w), 721 (w), 667 (m), 638 (w). Anal calcd for $C_{65}H_{83}N_3OS_4$ [1051]: C 74.31, H 7.96, N 4.00, S 12.21; Found: C 74.08, H 7.92, N 3.93, S 12.29.

---

[103] Two CH_3 signals coincide.
[104] Two CH_2 signals coincide.
[105] Three CH_2 signals coincide.
[106] Two CH signals coincide.
[107] A CH and quaternary signal coincide.

2.1.5.25. (*Z*)-3-[10-(2-Decyltetradecyl)-10'-hexyl-7'-(*p*-tolyl)-10*H*,10'*H*-(3,3'-biphenothiazin)-7-yl]-2-(4-nitrophenyl)acrylonitrile (12y)

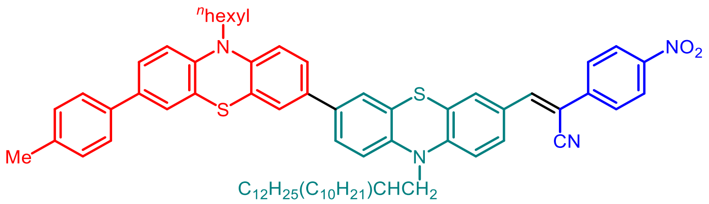

$C_{70}H_{86}N_4O_2S_2$ [1079.59]

According to the GP and after purification by chromatography on silica gel (*n*-hexane, *n*-hexane/acetone 30:1) and drying under vacuo compound **12y** (436 mg, 79%) was obtained as a dark red amorphous solid, Mp 62–64 °C. R_f (*n*-hexane/acetone 10:1) = 0.24.

^{1}H NMR (600 MHz, acetone-d$_6$): δ 0.79–0.87 (m, 9 H), 1.13–1.47 (m, 46 H), 1.79 (quin, ^3J = 7.8 Hz, 2 H), 1.99–2.04 (m, 1 H), 2.33 (s, 3 H), 3.86 (d, ^3J = 7.2 Hz, 2 H), 3.89 (t, ^3J = 7.1 Hz, 2 H), 6.95–7.01 (m, 2 H), 7.04 (d, ^3J = 8.6 Hz, 1 H), 7.06 (d, ^3J = 8.6 Hz, 1 H), 7.20 (d, ^3J = 7.9 Hz, 2 H), 7.31–7.35 (m, 3 H), 7.36–7.43 (m, 3 H), 7.45–7.47 (m, 2 H), 7.77 (d, ^4J = 2.1 Hz, 1 H), 7.87–7.96 (m, 4 H), 8.24–8.30 (m, 2 H). ^{13}C NMR (150 MHz, acetone-d$_6$): δ 14.35 (CH_3), 14.44 (CH_3), 14.45 (CH_3), 21.1 (CH_3), 23.35 (CH_2), 23.37 (CH_2), 23.38 (CH_2), 26.76 (CH_2), 26.81 (CH_2), 27.3 (CH_2), 27.5 (CH_2), 30.15 (CH_2), 30.16 (CH_2), 30.17 (CH_2), 30.20 (CH_2), 30.39 (CH_2), 30.41 (CH_2), 30.42 (CH_2), 30.45 (CH_2), 30.47 (CH_2), 30.51 (CH_2), 30.6 (CH_2), 30.7 (CH_2), 31.9 (CH_2), 32.0 (CH_2), 32.3 (CH_2), 32.68 (CH_2), 32.69 (CH_2), 35.5 (CH), 48.0 (CH_2), 52.2 (CH_2), 106.4 (C_{quat}), 116.6 (CH), 116.7 (CH), 117.0 (CH), 118.0 (CH), 118.5 (C_{quat}), 125.0 (CH), 125.3 (C_{quat}), 125.4 (C_{quat}), 125.6 (CH), 125.69 (C_{quat}), 125.72 (CH), 125.8 (CH), 126.0 (C_{quat}), 126.15 (CH), 126.22 (CH), 126.5 (CH), 126.9 (2 CH), 127.3 (2 CH), 128.5 (C_{quat}), 129.6 (CH), 130.3 (CH), 130.8 (CH), 131.2 (CH), 134.4 (C_{quat}), 135.9 (C_{quat}), 136.0 (C_{quat}), 137.4 (C_{quat}), 137.7 (C_{quat}), 142.0 (C_{quat}), 143.9 (C_{quat}), 144.8 (C_{quat}), 145.0 (C_{quat}), 148.3 (C_{quat}), 149.4 (C_{quat}). MS (MALDI-TOF) calcd for $C_{70}H_{86}N_4O_2S_2$ *m/z:* 1078.62; Found: 1078.6 ($[M]^+$). IR: $\tilde{\nu}$ [cm^{-1}] = 2980 (m), 2970 (w), 2959 (w), 2920 (m), 2851 (w), 1576 (m), 1562 (w), 1516 (m), 1505 (w), 1476 (m), 1462 (m), 1402 (w), 1373 (m), 1335 (s), 1312 (m), 1254 (m), 1238 (m), 1209 (m), 1163 (w), 1150 (w), 1109 (m), 1080 (w), 1007 (w), 990 (w), 974 (w), 930 (w), 893 (w), 879 (w), 851 (m), 815 (m), 801 (m), 750 (m), 720 (m), 687 (m), 665 (m), 635 (m). Anal calcd for $C_{70}H_{86}N_4O_2S_2$ [1080]: C 77.88, H 8.03, N 5.19; Found: C 77.62, H 8.05, N 5.02.

2.1.5.26. (*Z*)-4-{[10-(2-Decyltetradecyl)-10'-hexyl-7'-(*p*-tolyl)-10H,10'H-(3,3'-biphenothiazin)-7-yl]methylene}-3-methyl-1-phenyl-1H-pyrazol-5[4H]-one (12z)

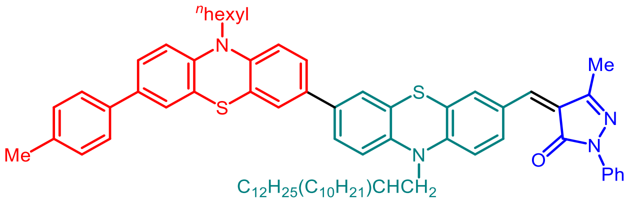

$C_{72}H_{90}N_4OS_2$ [1091.64]

According to the GP and after purification by chromatography on silica gel (*n*-hexane, *n*-hexane/acetone 30:1) and drying under vacuo compound **12z** (371 mg, 68%) was obtained as a dark red amorphous solid, Mp 55–59 °C. R_f (*n*-hexane/acetone 10:1) = 0.27.

^{1}H NMR (300 MHz, acetone-d$_6$/CS$_2$ 4:1): δ 0.84–0.96 (m, 9 H), 1.19–1.56 (m, 46 H), 1.85 (quin, ^3J = 7.5 Hz, 2 H), 2.06–2.11 (m, 1 H), 2.33 (s, 3 H), 2.36 (s, 3 H), 3.90–3.99 (m, 4 H), 7.00 (dd, ^3J = 8.5 Hz, ^4J = 2.4 Hz, 2 H), 7.05 (s, 1 H), 7.08 (d, ^4J = 1.3 Hz, 1 H), 7.10–7.18 (m, 1 H), 7.21 (d, ^3J = 8.0 Hz, 2 H), 7.31–7.51 (m, 11 H), 8.04–8.10 (m, 2 H), 8.48 (dd, ^3J = 8.7 Hz, ^4J = 2.0 Hz, 1 H), 8.70 (d, ^4J = 2.0 Hz, 1 H). ^{13}C NMR (75 MHz, acetone-d$_6$/CS$_2$ 4:1): δ 13.6 (CH$_3$), 14.6 (CH$_3$), 14.7 (CH$_3$),[108] 21.3 (CH$_3$), 23.6 (CH$_2$),[109] 27.0 (CH$_2$),[110] 27.4 (CH$_2$), 27.7 (CH$_2$), 30.3 (CH$_2$),[110] 30.4 (CH$_2$),[110] 30.59 (CH$_2$),[111] 30.63 (CH$_2$), 30.7 (CH$_2$), 30.9 (CH$_2$),[110] 32.2 (CH$_2$),[110] 32.4 (CH$_2$), 32.8 (CH$_2$),[110] 35.7 (CH), 48.1 (CH$_2$), 52.4 (CH$_2$), 116.4 (CH), 116.5 (CH), 116.6 (CH), 118.0 (CH), 119.0 (CH), 124.8 (CH), 125.3 (C$_{quat}$), 125.4 (C$_{quat}$),[112] 125.6 (CH, C$_{quat}$),[113] 125.8 (CH), 125.9 (CH), 126.1 (C$_{quat}$), 126.2 (CH), 126.5 (CH), 127.0 (CH), 129.1 (C$_{quat}$), 129.3 (CH), 130.3 (CH), 133.7 (CH), 134.5 (C$_{quat}$), 136.0 (CH, C$_{quat}$),[113] 136.2 (C$_{quat}$), 137.3 (C$_{quat}$), 137.7 (C$_{quat}$), 139.9 (C$_{quat}$), 143.4 (C$_{quat}$), 144.7 (C$_{quat}$), 145.0 (C$_{quat}$), 146.3 (CH), 150.8 (C$_{quat}$), 151.7 (C$_{quat}$), 162.9 (C$_{quat}$). MS (MALDI-TOF) calcd for $C_{72}H_{90}N_4OS_2$ *m/z:* 1090.66; Found: 1090.7 ([M]$^+$). IR: $\tilde{\nu}$ [cm^{-1}] = 2953 (w), 2920 (m), 2851 (w), 1678 (w), 1614 (w), 1595 (w), 1574 (w), 1559 (w), 1533 (w), 1497 (w), 1458 (s), 1416 (w), 1375 (w), 1356 (w), 1337 (w), 1317 (m), 1275 (w), 1250 (m), 1215 (w), 1138 (m), 1105 (w), 1057 (w), 1028 (w), 995 (w), 930 (w), 901 (w), 874 (w), 804 (m), 783 (w), 768 (w), 752 (w), 723 (w), 691 (w), 662 (w). Anal calcd for $C_{72}H_{90}N_4OS_2$ [1092]: C 79.22, H 8.31, N 5.13, S 5.87; Found: C 79.22, H 8.14, N 5.08, S 5.59.

[108] Two CH$_3$ signals coincide.
[109] Three CH$_2$ signals coincide.
[110] Two CH$_2$ signals coincide.
[111] Four CH$_2$ signals coincide
[112] Two quaternary signals coincide.
[113] A CH and a quaternary signal coincide.

2.2. General Procedure (GP) for the Knoevenagel Condensation Synthesis of Reference Merocyanines 14-17

10-(2-Decyltetradecyl)-10H-phenothiazine-3-carbaldehyde (**13**), CH-acidic compound **7** (1.20 equivs), and a catalytic or equimolar amount of ammonium acetate or diethylamine in a screw-cap Schlenk vessel with a magnetic stir bar were dissolved in a mixture of 1,4-dioxane and acetic acid and heated at 95 °C (oil bath) for 3-8 h (for experimental details, see Table S6). Intensive red to dark violet solutions were formed. After cooling to room temp the reaction mixture was diluted with dichloromethane (25 mL/mmol) and the organic layer was washed with distilled water and saturated sodium sulfite solution until the aqueous phase did not smell like acetic acid. The combined aqueous phases were extracted with dichloromethane and the combined organic layers were dried (anhydrous magnesium sulfate) and the solvents were removed in vacuo. The dark red to violet residue was adsorbed on celite® and purified by flash chromatography on silica gel (*n*-hexane/acetone, toluene or ethyl acetate and gradients thereof) to furnish after drying under high vacuum for one day the chromophores **14-17**.

Table S6. Experimental details of the Knoevenagel condensation synthesis of phenothiazinyl-merocyanines **14-17**.

**Entry**	**Phenothiazinyl aldehyde 13** [mg] (mmol)	**methylene active compound 7** [mg] (mmol)	**Organo catalyst** [mg] (mmol)	**1,4-dioxane/AcOH** [mL]	**Reaction time *t*** [h]	**Product** [mg] (%)[a]
1	260 (0.461)	81 (0.55) of **7c**	1 drop of Et_2NH	3.0/1.5	3	300 (94) of **14**
2	141 (0.250)	44 (0.30) of **7a**	20 (0.25) of NH_4OAc	2.0/1.0	5	166 (96) of **15**
3	247 (0.438)	88 (0.53) of **7f**	34 (0.44) of NH_4OAc	3.0/1.5	8	302 (97) of **16**
4	563 (0.998)	210 (1.20) of **7e**	1 drop of Et_2NH	4.0/2.0	3	621 (86) of **17**

2.2.1. 2-{[10-(2-Decyltetradecyl)-10*H*-phenothiazin-3-yl]methylene}-1*H*-inden-1,3[2*H*]-dione (14)

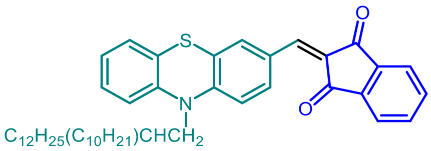

$C_{46}H_{61}NO_2S$ [692.05]

According to the GP and after purification by chromatography on silica gel (*n*-hexane, *n*-hexane/acetone 40:1) and drying under vacuo compound **14** (300 mg, 94%) was obtained as a red amorphous solid, Mp 65–69 °C. R_f (*n*-hexane/acetone 10:1) = 0.29.

^{1}H NMR (300 MHz, CD_2Cl_2): δ 0.86 (t, ^3J = 6.7 Hz, 3 H), 0.87 (t, ^3J = 6.7 Hz, 3 H), 1.12–1.46 (m, 40 H), 1.99 (hep, ^3J = 6.5 Hz, 1 H), 3.83 (d, ^3J = 7.2 Hz, 2 H), 6.91–7.04 (m, 3 H), 7.14–7.24 (m, 2 H), 7.69 (s, 1 H), 7.75–7.81 (m, 2 H), 7.89–7.99 (m, 2 H), 8.30 (dd, ^3J = 8.7 Hz, ^4J = 2.1 Hz, 1 H), 8.45 (d, ^4J = 2.0 Hz, 1 H). ^{13}C NMR (75 MHz, CD_2Cl_2): δ 14.5 (CH_3),[114] 23.3 (CH_2),[115] 26.7 (CH_2),[115] 29.95 (CH_2), 29.96 (CH_2), 30.0 (CH_2), 30.19 (CH_2),[115] 30.20 (CH_2), 30.25 (CH_2),[115] 30.29 (CH_2), 30.5 (CH_2),[115] 31.9 (CH_2),[115] 32.5 (CH_2),[115] 35.4 (CH), 52.4 (CH_2), 116.1 (CH), 117.3 (CH), 123.3 (CH), 123.4 (CH), 124.2 (CH), 125.5 (C_{quat}), 125.8 (C_{quat}), 127.1 (C_{quat}), 128.0 (CH), 128.2 (CH), 128.5 (C_{quat}), 133.6 (CH), 135.4 (CH), 135.6 (CH), 135.9 (CH), 140.6 (C_{quat}), 143.0 (C_{quat}), 144.4 (C_{quat}), 145.7 (CH), 151.4 (C_{quat}), 189.9 (C_{quat}), 190.9 (C_{quat}). MS (MALDI-TOF) calcd. for $C_{46}H_{61}NO_2S$ *m/z*: 691.44; Found: 691.5 ($[M]^+$). IR: $\tilde{\nu}$ [cm^{-1}] = 3055 (w), 2951 (w), 2920 (m), 2849 (m), 1719 (w), 1676 (s), 1609 (w), 1584 (m), 1560 (s), 1535 (m), 1491 (m), 1456 (s), 1422 (w), 1375 (w), 1344 (m), 1328 (m), 1310 (w), 1285 (m), 1250 (w), 1202 (s), 1171 (w), 1155 (m), 1105 (w), 1084 (w), 1040 (w), 1017 (w), 995 (m), 966 (w), 945 (w), 924 (w), 878 (w), 845 (w), 827 (w), 783 (w), 739 (s), 720 (m), 706 (w), 671 (w). Anal. calcd. for $C_{46}H_{61}NO_2S$ [692.1]: C 79.83, H 8.88, N 2.02, S 4.63; Found: C 79.61, H 8.68, N 2.01, S 4.34.

2.2.2. (*Z*)-5-{[10-(2-Decyltetradecyl)-10H-phenothiazin-3-yl]methylene}-3-methyl-2-thioxothiazolidin-4-one (15)

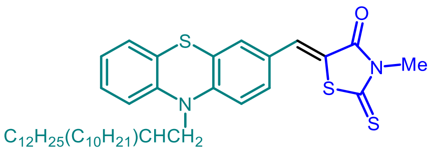

$C_{41}H_{60}N_2OS_3$ [693.12]

According to the GP and after purification by chromatography on silica gel (*n*-hexane, *n*-hexane/ethyl acetate 40:1) and drying under vacuo compound **15** (166 mg, 96%) was obtained as an orange amorphous solid, Mp 123–128 °C. R_f (*n*-hexane/acetone 10:1) = 0.49. ^{1}H NMR (300 MHz, acetone-d$_6$): δ 0.87 (t, ^3J = 6.7 Hz, 3 H), 0.88 (t, ^3J = 6.7 Hz, 3 H), 1.18–1.47 (m, 40 H), 1.94–2.05 (m, 1 H), 3.45 (s, 3 H), 3.91 (d, ^3J = 7.2 Hz, 2 H), 7.01 (dt, ^3J = 7.5 Hz, ^4J = 1.2 Hz, 1 H), 7.07–7.28 (m, 4 H), 7.31 (d, ^4J = 2.1 Hz, 1 H), 7.42 (dd, ^3J = 8.6 Hz, ^4J = 2.2 Hz, 1 H), 7.61 (s, 1 H). ^{13}C NMR (75 MHz, acetone-d$_6$): δ 14.4 (CH_3),[116] 23.4 (CH_2),[117] 26.7 (CH_2), 26.8 (CH_2), 30.11 (CH_2), 30.14 (CH_2),[117] 30.3 (CH_2), 30.35 (CH_2), 30.39 (CH_2), 30.42 (CH_2),[117] 30.43 (CH_2), 30.5 (CH_2), 30.59 (CH_2), 30.62 (CH_2), 31.5 (CH_3), 31.98 (CH_2), 32.00 (CH_2), 32.7 (CH_2),[117] 35.4 (CH), 52.2 (CH_2), 117.5 (CH), 117.8 (CH), 121.0 (C_{quat}), 124.4 (CH),

[114] Two CH_3 signals coincide.
[115] Two CH_2 signals coincide.
[116] Two CH_3 signals coincide.
[117] Two CH_2 signals coincide.

125.2 (C_{quat}), 127.1 (C_{quat}), 128.3 (CH), 128.5 (C_{quat}), 128.6 (CH), 130.2 (CH), 131.5 (CH), 132.6 (CH), 145.2 (C_{quat}), 149.1 (C_{quat}), 168.0 (C_{quat}), 194.1 (C_{quat}). MS (MALDI-TOF) calcd. for $C_{41}H_{60}N_2OS_3$ *m/z*: 692.39; Found: 692.4 ($[M]^+$). IR (KBr): $\tilde{\nu}$ [cm^{-1}] = 3055 (w), 2951 (w), 2916 (m), 2849 (m), 1707 (s), 1587 (m), 1570 (m), 1495 (m), 1458 (s), 1442 (w), 1425 (m), 1402 (m), 1348 (w), 1335 (w), 1285 (s), 1250 (m), 1223 (m), 1169 (w), 1123 (s), 1103 (s), 1037 (w), 991 (w), 959 (w), 937 (w), 895 (w), 853 (w), 804 (w), 772 (w), 752 (m), 741 (m), 719 (w), 677 (w), 651 (w), 611 (w). Anal. calcd. for $C_{41}H_{60}N_2OS_3$ [693.1]: C 71.05, H 8.73; N 4.04, S 13.88; Found: C 70.91, H 8.72, N 4.01, S 13.73.

2.2.3. (*Z*)-3-(10-(2-Decyltetradecyl)-10*H*-phenothiazin-3-yl)-2-(4-nitrophenyl)acrylonitrile (16)

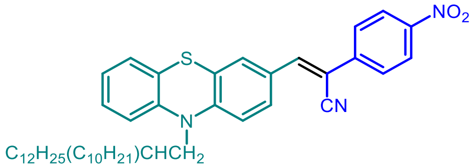

$C_{45}H_{61}N_3O_2S$ [708.05]

According to the GP and after purification by chromatography on silica gel (*n*-hexane, *n*-hexane/acetone 30:1) and drying under vacuo compound **15** (302 mg, 97%) was obtained as a dark red resin. R_f (*n*-hexane/acetone 10:1) = 0.37.

^{1}H NMR (300 MHz, acetone-d_6): δ 0.86 (t, ^3J = 6.4 Hz, 6 H), 1.13–1.53 (m, 40 H), 1.96–2.04 (m, 1 H), 3.94 (d, ^3J = 7.2 Hz, 2 H), 6.97–7.28 (m, 5 H), 7.85 (s, 1 H), 7.92–8.03 (m, 4 H), 8.33 (d, ^3J = 8.8 Hz, 2 H). ^{13}C NMR (75 MHz, acetone-d_6): δ 14.4 (CH_3),[118] 23.3 (CH_2),[119] 26.7 (CH_2), 26.8 (CH_2), 30.1 (CH_2),[120] 30.3 (CH_2),[119] 30.36 (CH_2), 30.41 (CH_2),[119] 30.5 (CH_2), 30.60 (CH_2), 30.63 (CH_2), 31.96 (CH_2), 31.99 (CH_2), 32.7 (CH_2),[119] 35.4 (CH), 52.2 (CH_2), 106.5 (C_{quat}), 117.1 (CH), 117.8 (CH), 118.5 (C_{quat}), 124.3 (CH), 125.1 (CH), 125.2 (C_{quat}), 126.5 (C_{quat}), 127.4 (CH), 128.3 (CH), 128.59 (CH), 128.61 (C_{quat}), 129.5 (CH), 130.8 (CH), 142.0 (C_{quat}), 145.1 (CH), 145.2 (C_{quat}), 148.4 (C_{quat}), 149.7 (C_{quat}). MS (MALDI-TOF) calcd. for $C_{45}H_{61}N_3O_2S$ *m/z*: 707.45; Found: 707.4 ($[M]^+$). IR: $\tilde{\nu}$ [cm^{-1}] = 2957 (w), 2920 (s), 2851 (m), 2212 (w), 1587 (m), 1566 (s), 1514 (s), 1468 (s), 1456 (s), 1443 (m), 1422 (m), 1402 (s), 1375 (w), 1339 (s), 1285 (w), 1252 (m), 1211 (m), 1194 (w), 1175 (m), 1132 (w), 1109 (m), 1042 (w), 1003 (w), 934 (w), 878 (w), 847 (s), 810 (m), 746 (s), 721 (m), 687 (m), 625 (w). Anal. calcd. for $C_{45}H_{61}N_3O_2S$ [708.1]: C 76.33, H 8.68, N 5.93, S 4.53; Found: C 76.36, H 8.70, N 5.83, S 4.41.

---

[118] Two CH_3 signals coincide.
[119] Two CH_2 signals coincide.
[120] Four CH_2 signals coincide.

2.2.4. (*Z*)-4-{[10-(2-Decyltetradecyl)-10*H*-phenothiazin-3-yl]methylene}-5-methyl-2-phenyl-2,4-dihydro-3*H*-pyrazol-3-one (17)

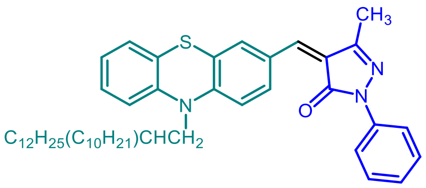

$C_{47}H_{65}N_3OS$ [720.10]

According to the GP and after purification by chromatography on silica gel (*n*-hexane, *n*-hexane/acetone 20:1) and drying under vacuo compound **16** (621 mg, 86%) was obtained as a dark red viscous oil, Mp 65–69 °C. R_f (*n*-hexane/acetone 10:1) = 0.18.

^{1}H NMR (300 MHz, CD_2Cl_2): δ 0.88 (m, 6 H), 1.16–1.46 (m, 40 H), 2.00 (hep, ^3J = 6.4 Hz, 1 H), 2.30 (s, 3 H), 3.82 (d, ^3J = 7.1 Hz, 2 H), 6.92–6.98 (m, 2 H), 7.00 (dd, ^3J = 7.5 Hz, ^4J = 1.1 Hz, 1 H), 7.13–7.21 (m, 3 H), 7.23 (s, 1 H), 7.37–7.45 (m, 2 H), 7.96–8.03 (m, 2 H), 8.38 (dd, ^3J = 8.7 Hz, ^4J = 2.1 Hz, 1 H), 8.51 (d, ^4J = 2.1 Hz, 1 H). ^{13}C NMR (75 MHz, CD_2Cl_2): δ 13.7 (CH_3), 14.5 (CH_3),[121] 23.3 (CH_2),[122] 26.7 (CH_2),[122] 29.95 (CH_2), 29.96 (CH_2), 30.03 (CH_2),[122] 30.19 (CH_2),[122] 30.21 (CH_2), 30.25 (CH_2),[122] 30.29 (CH_2), 30.5 (CH_2),[122] 32.0 (CH_2),[122] 32.5 (CH_2),[122] 35.4 (CH), 52.4 (CH_2), 116.0 (CH), 117.3 (CH), 119.3 (CH), 124.2 (CH), 124.9 (CH), 125.3 (C_{quat}), 125.5 (C_{quat}), 125.7 (C_{quat}), 128.0 (CH), 128.2 (CH), 128.4 (C_{quat}), 129.2 (CH), 133.3 (CH), 135.2 (CH), 139.4 (C_{quat}), 144.4 (C_{quat}), 145.8 (CH), 151.0 (C_{quat}), 151.5 (C_{quat}), 162.9 (C_{quat}). MS (MALDI-TOF) calcd. for $C_{47}H_{65}N_3OS$-H^+ *m/z*: 720.49; Found: 720.5 ($[MH]^+$). IR: $\tilde{\nu}$ [cm^{-1}] = 3059 (w), 2920 (s), 2851 (m), 1721 (w), 1676 (s), 1614 (w), 1586 (m), 1559 (s), 1537 (m), 1495 (m), 1456 (s), 1445 (m), 1418 (w), 1373 (w), 1343 (m), 1317 (s), 1285 (m), 1250 (m), 1215 (s), 1202 (s), 1169 (m), 1138 (m), 1105 (w), 1084 (m), 1061 (w), 1038 (w), 1017 (w), 995 (m), 966 (w), 924 (w), 903 (w), 878 (w), 847 (w), 824 (w), 783 (w), 739 (s), 720 (m), 691 (m), 664 (m), 637 (w), 621 (w). Anal. calcd. for $C_{47}H_{65}N_3OS$ [720.1]: C 78.39, H 9.10, N 5.84, S 4.45; Found: C 78.63, H 9.29, N 5.82, S 4.52.

---

[121] Two CH_3 signals coincide.
[122] Two CH_2 signals coincide.

3. ^{1}H and ^{13}C NMR Spectra

3.1. 5-(Biphenyl-4-ylmethylene)-3-methyl-2-thioxothiazolidin-4-one (8a)

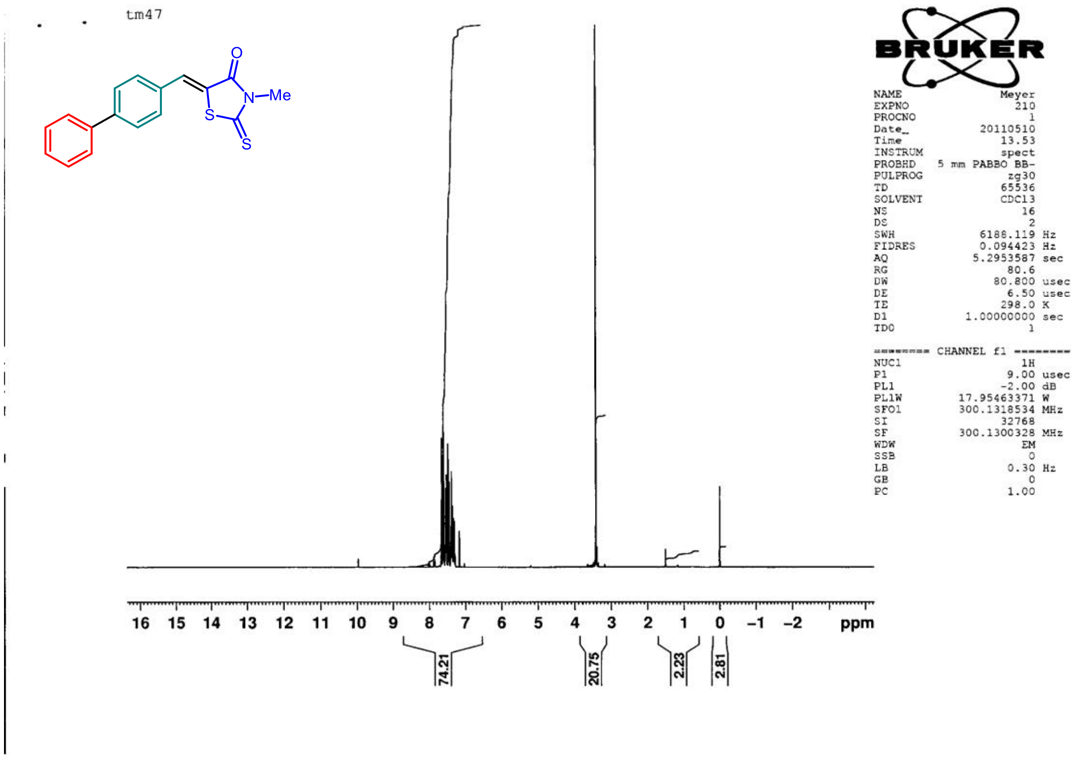

^{1}H NMR (300 MHz, $CDCl_3$) of compound **8a**.

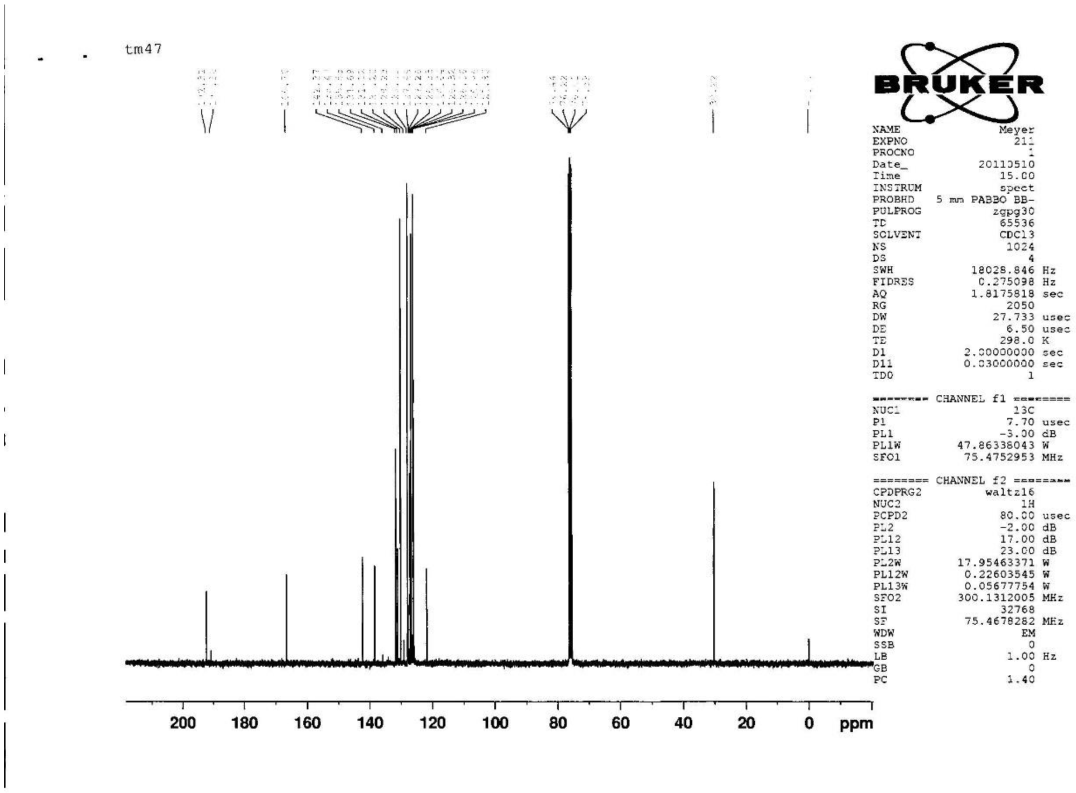

^{13}C NMR (75 MHz, $CDCl_3$) of compound **8a**.

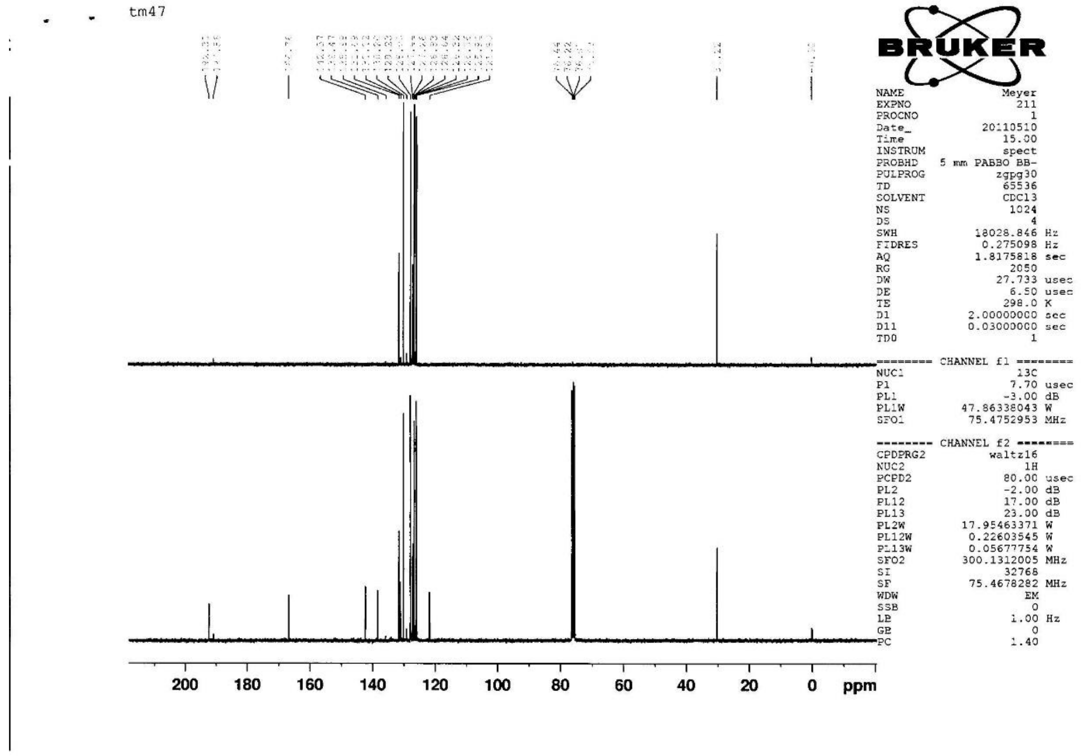

DEPT ^{13}C NMR (75 MHz, $CDCl_3$) of compound **8a**.

3.2. 2-(Biphenyl-4-ylmethylene)-1*H*-inden-1,3[2*H*]-dione (8b)

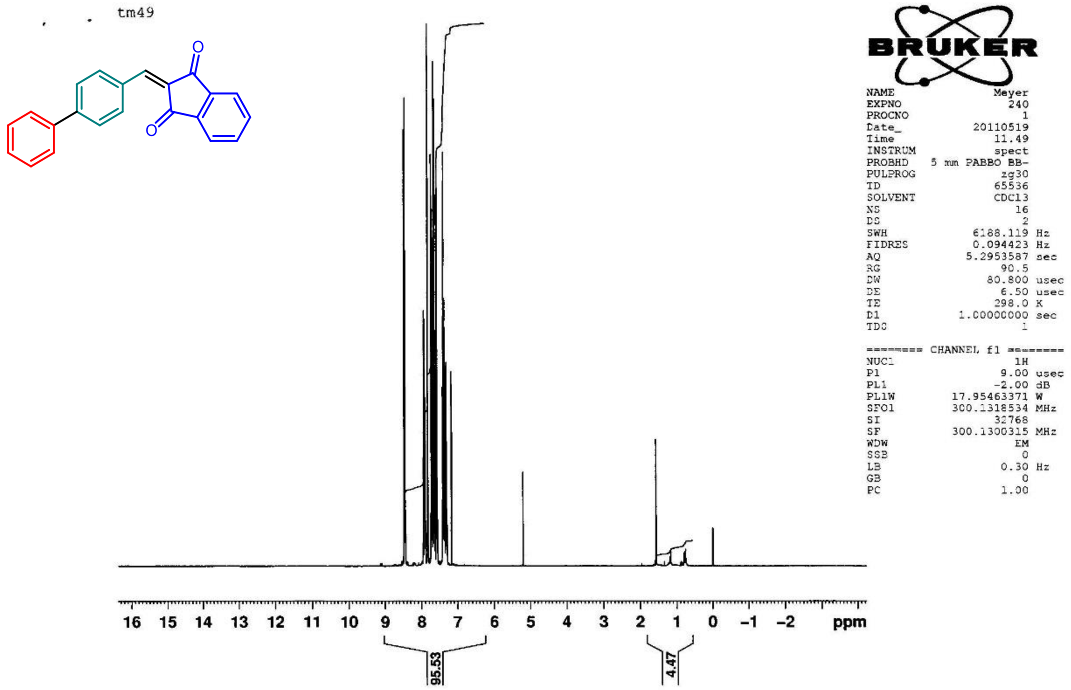

^{1}H NMR (300 MHz, $CDCl_3$) of compound **8b**.

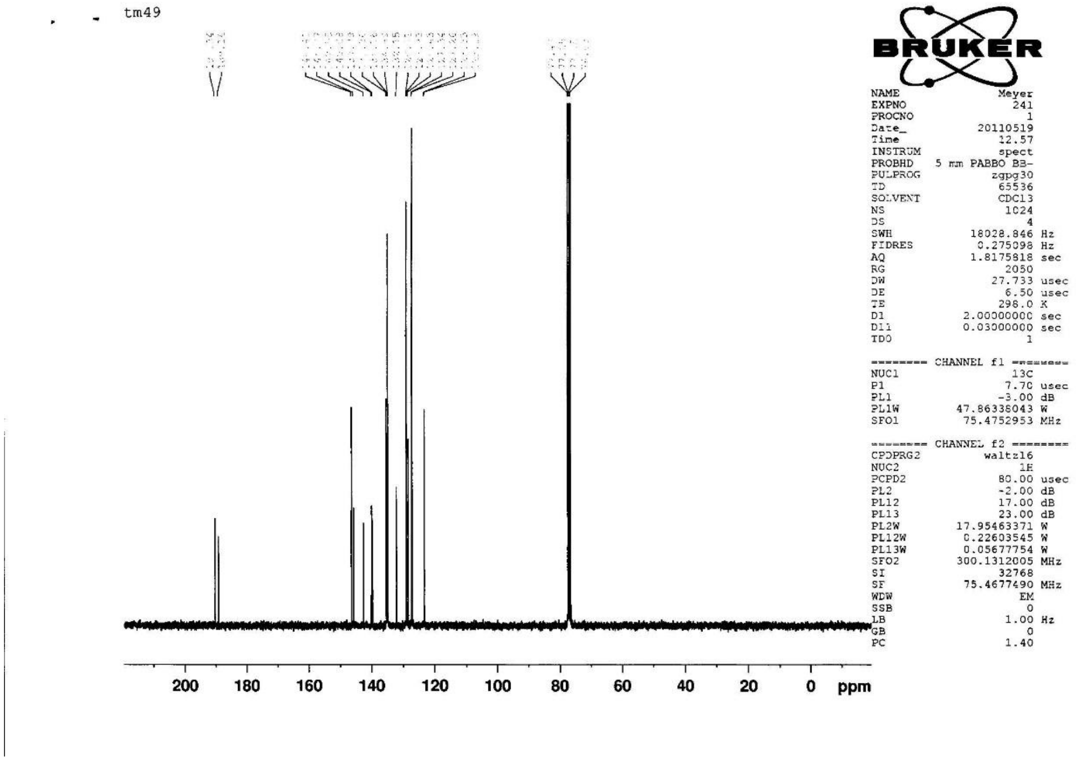

^{13}C NMR (75 MHz, $CDCl_3$) of compound **8b**.

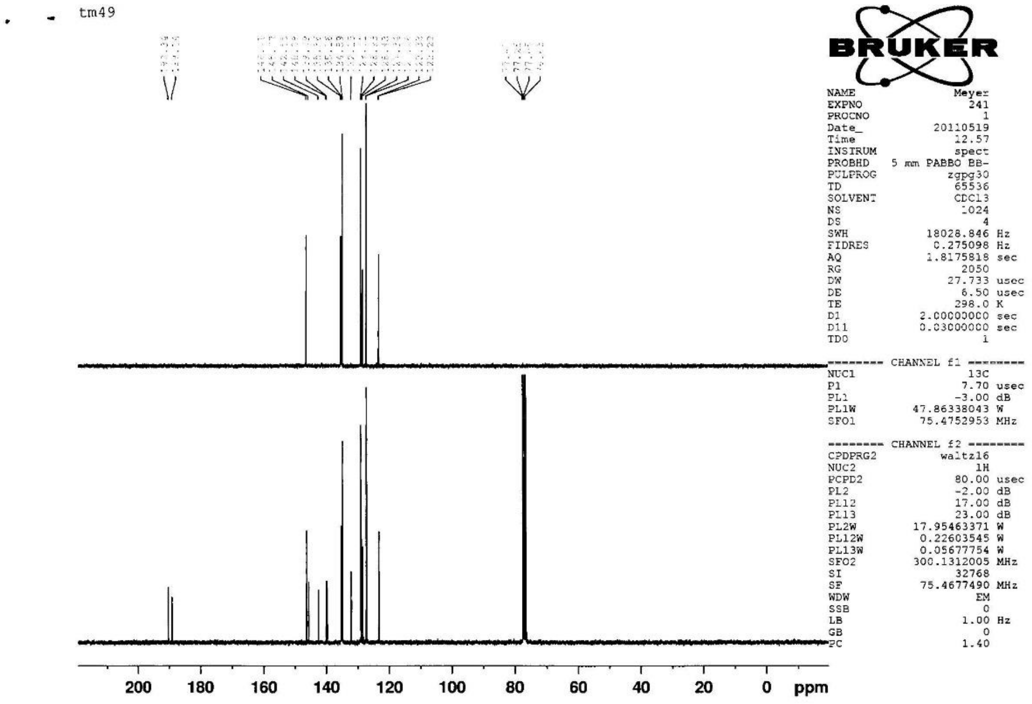

DEPT ^{13}C NMR (75 MHz, $CDCl_3$) of compound **8b**.

3.3. 5-{[4'-Methyl-(1,1'-biphenyl)-4-yl]methylene}-2-thioxothiazolidin-4-one (8c)

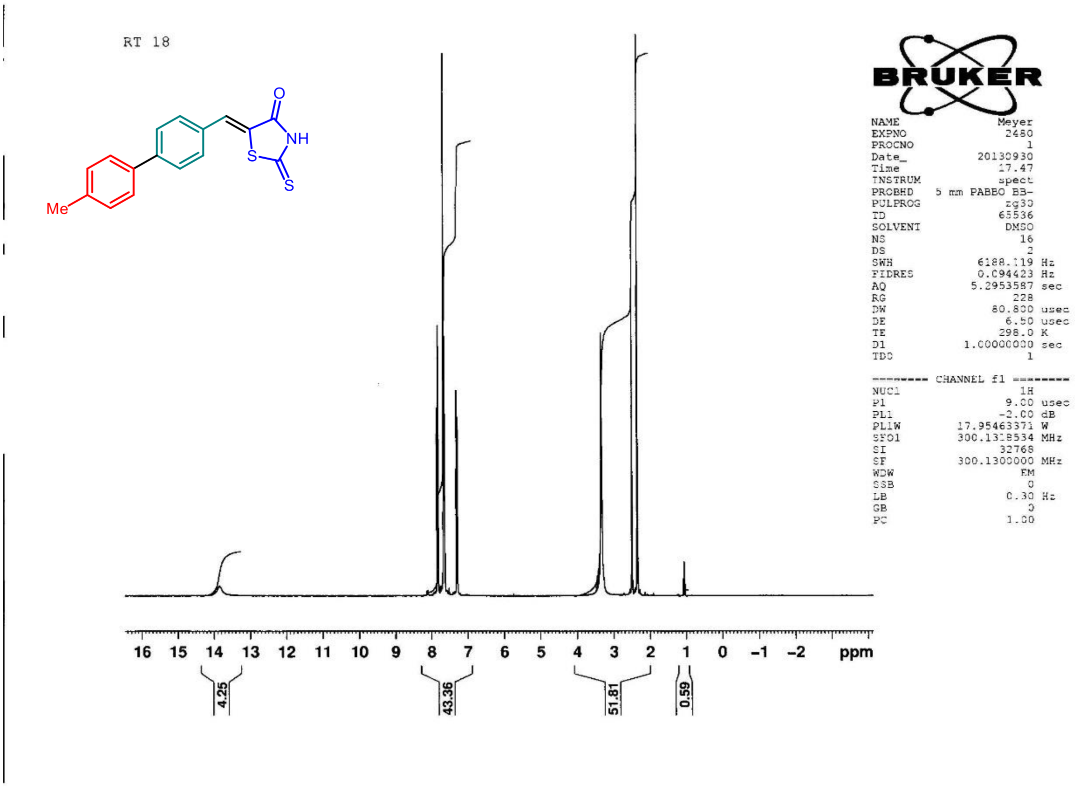

^{1}H NMR (300 MHz, DMSO-d_6) of compound **8c**.

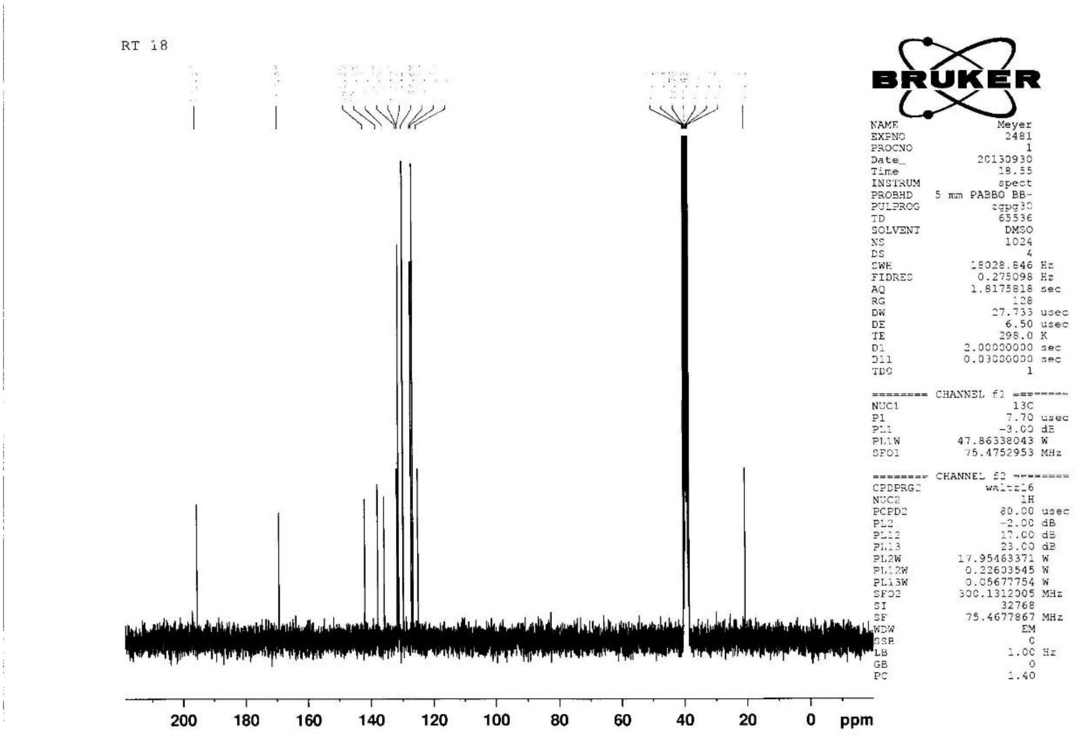

^{13}C NMR (75 MHz, DMSO-d_6) of compound **8c**.

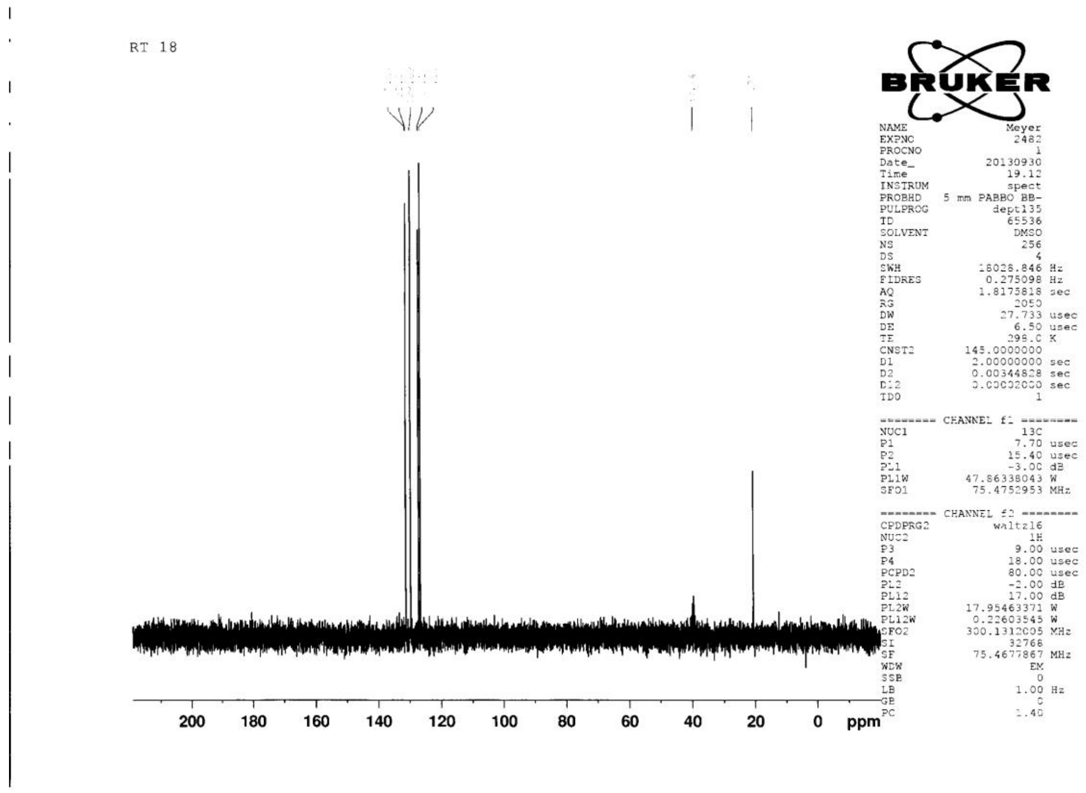

DEPT ^{13}C NMR (75 MHz, DMSO-d_6) of compound **8c**.

3.4. 3-(4'-Methyl-[1,1'-biphenyl]-4-yl)-2-(4-nitrophenyl)acrylnitrile (8d)

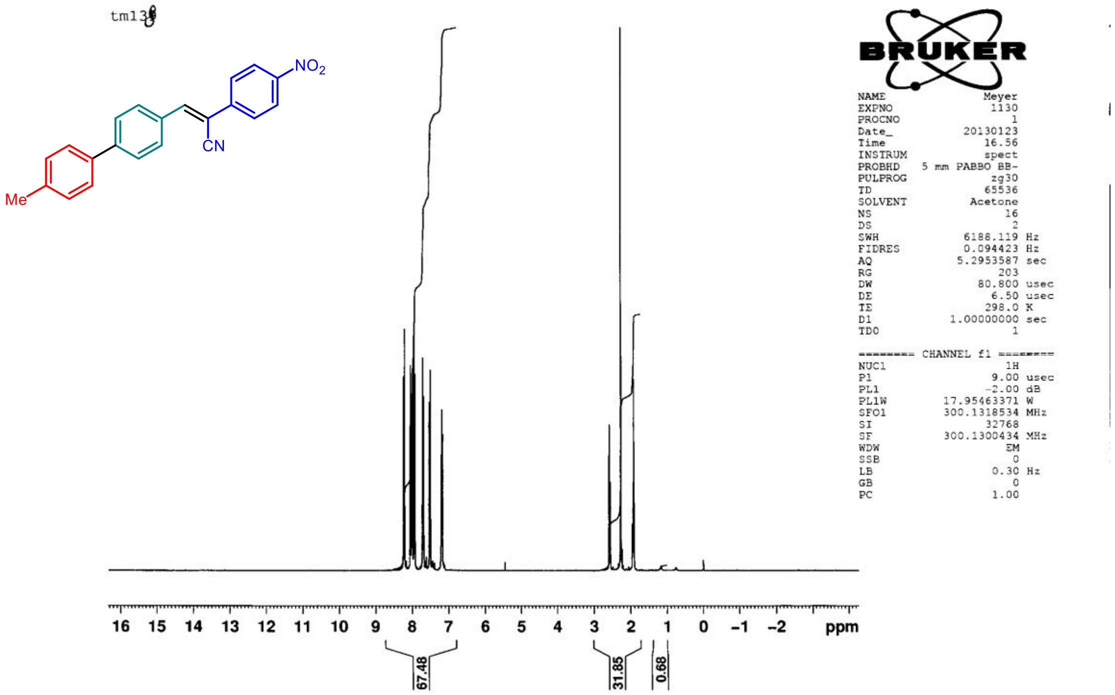

^{1}H NMR (300 MHz, acetone-d_6/CS_2 4:1) of compound **8d**.

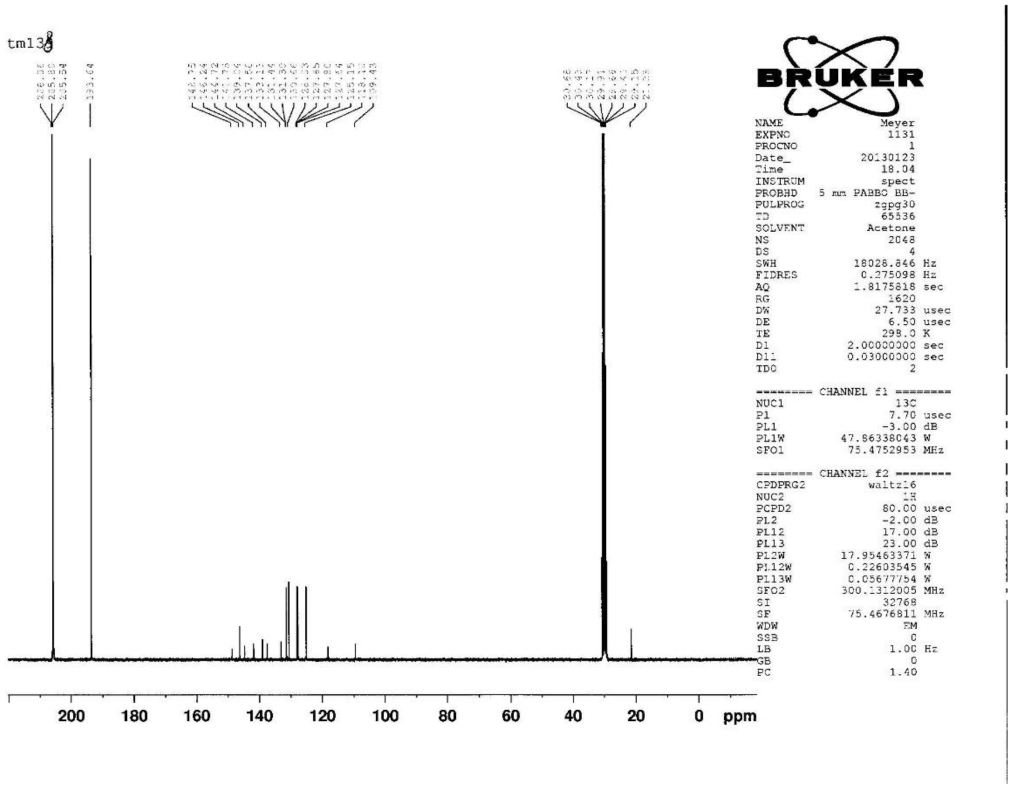

^{13}C NMR (75 MHz, acetone-d_6/CS_2 4:1) of compound **8d**.

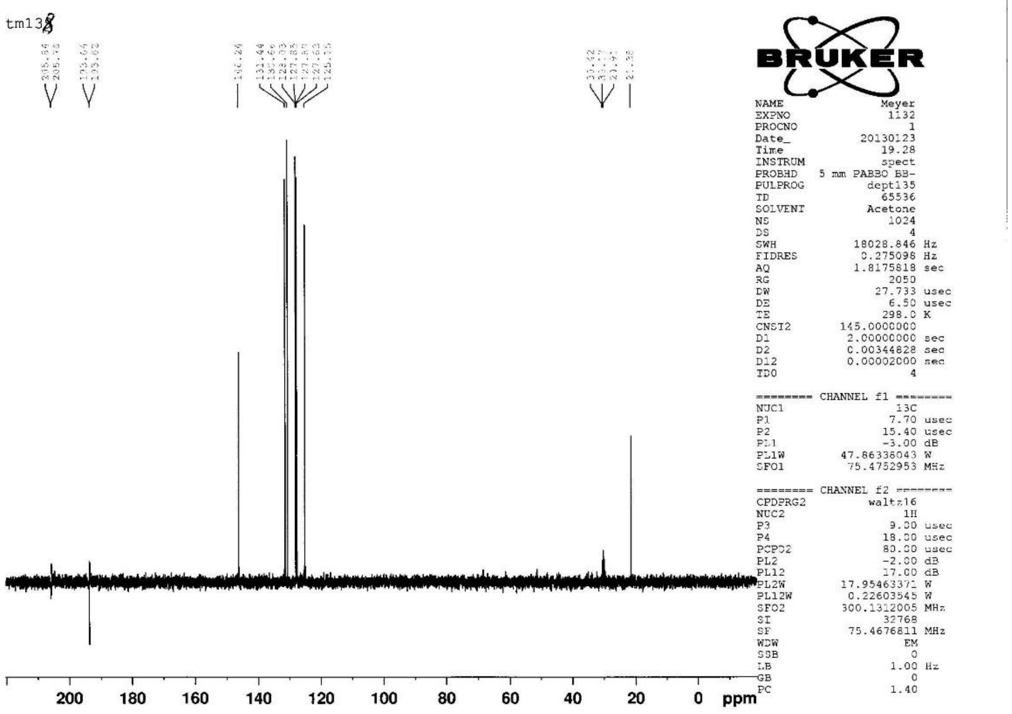

DEPT ^{13}C NMR (75 MHz, acetone-d_6/CS_2 4:1) of compound **8d**.

3.5. 3-Methyl-4-{[4'-methyl-(1,1'-biphenyl)-4-yl]methylene}-1-phenyl-1*H*-pyrazol-5[4*H*]-one (8e)

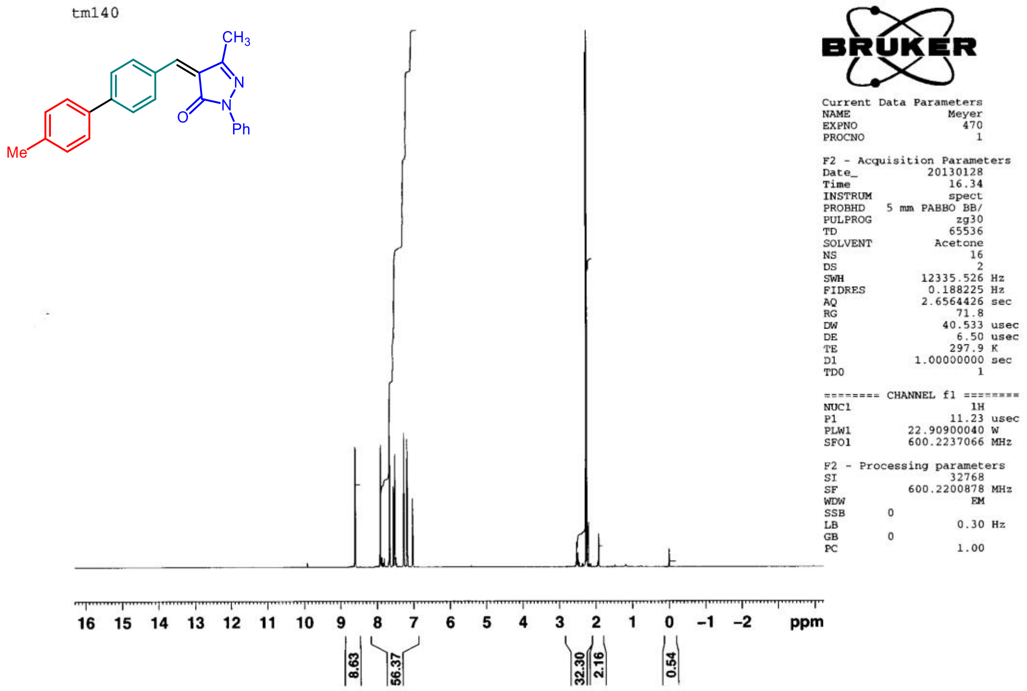

^{1}H NMR (600 MHz, acetone-d_6/CS_2 4:1) of compound **8e**.

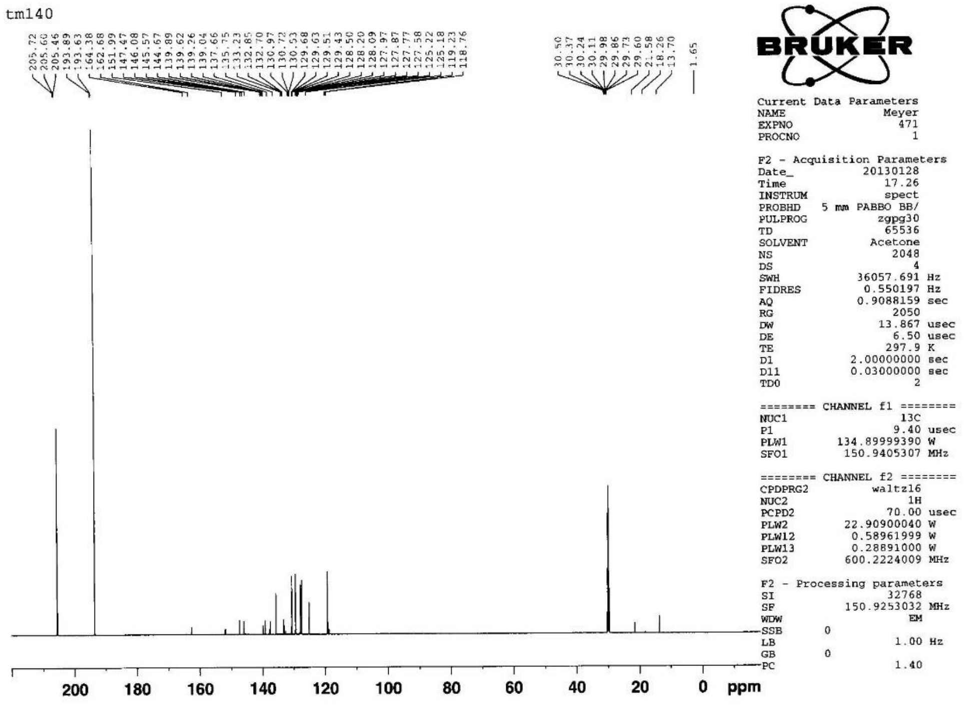

^{13}C NMR (150 MHz, acetone-d_6/CS_2 4:1) of compound **8e**.

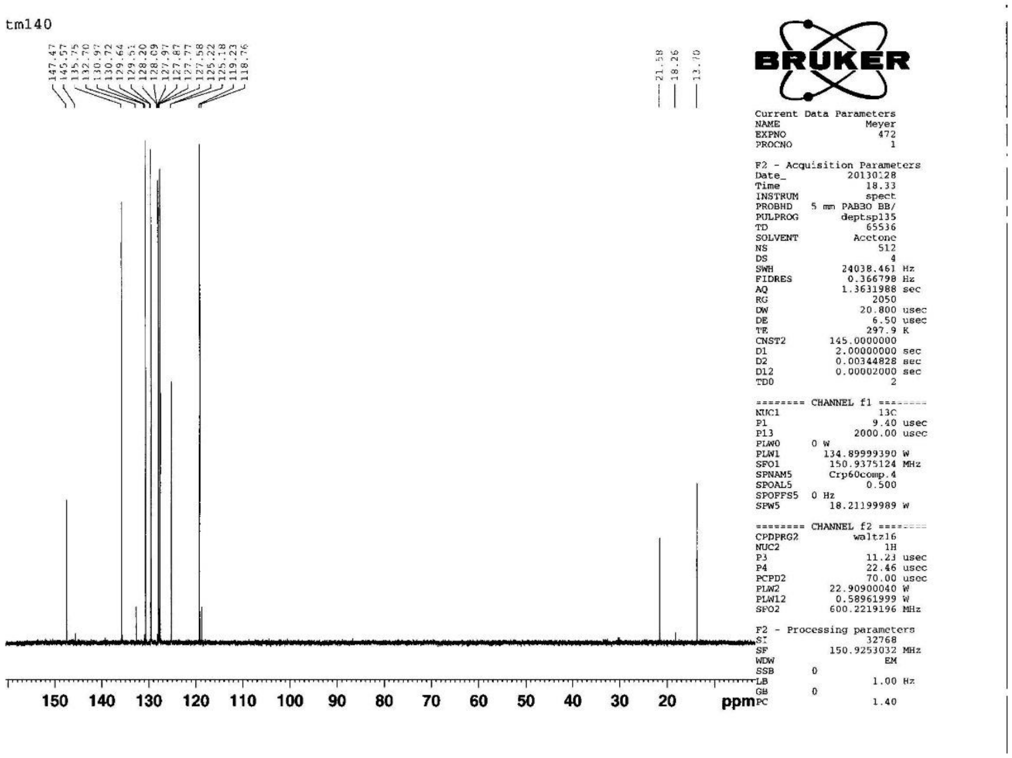

DEPT ^{13}C NMR (150 MHz, acetone-d_6/CS_2 4:1) of compound **8e**.

3.6. 3-Methyl-5-(4-(thiophen-3-yl)benzylidene)-2-thioxothiazolidin-4-one (8f)

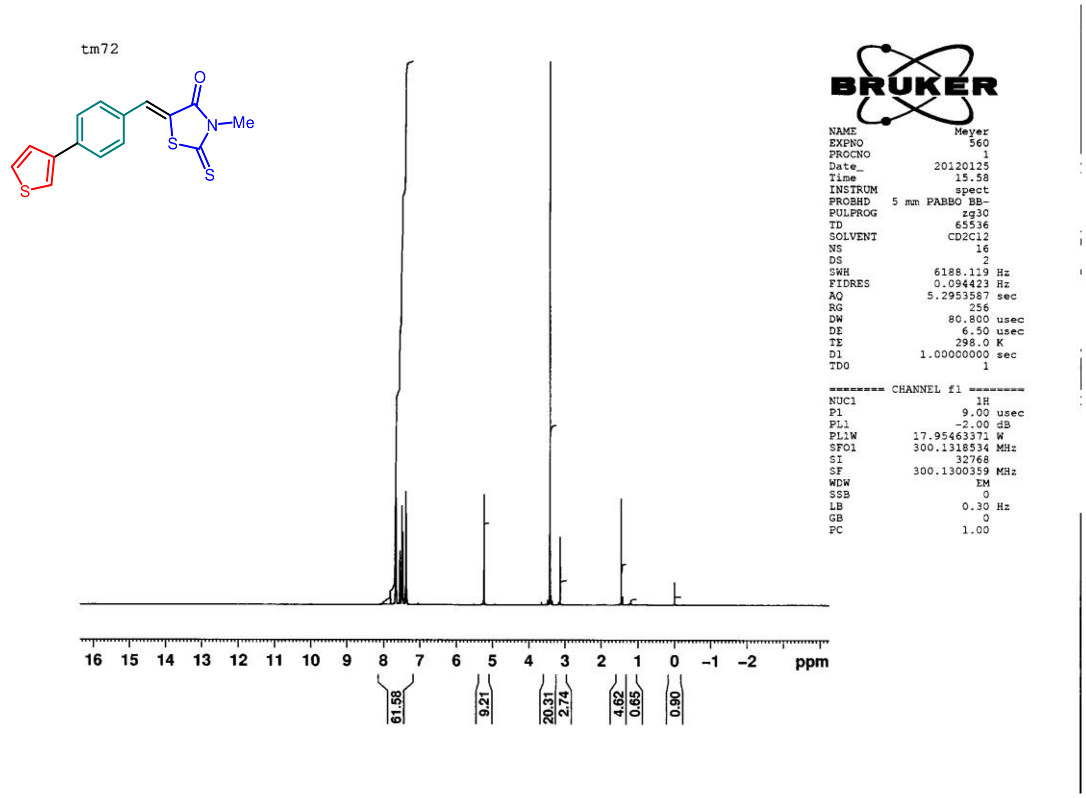

^{1}H NMR (300 MHz, CD_2Cl_2) of compound **8f**.

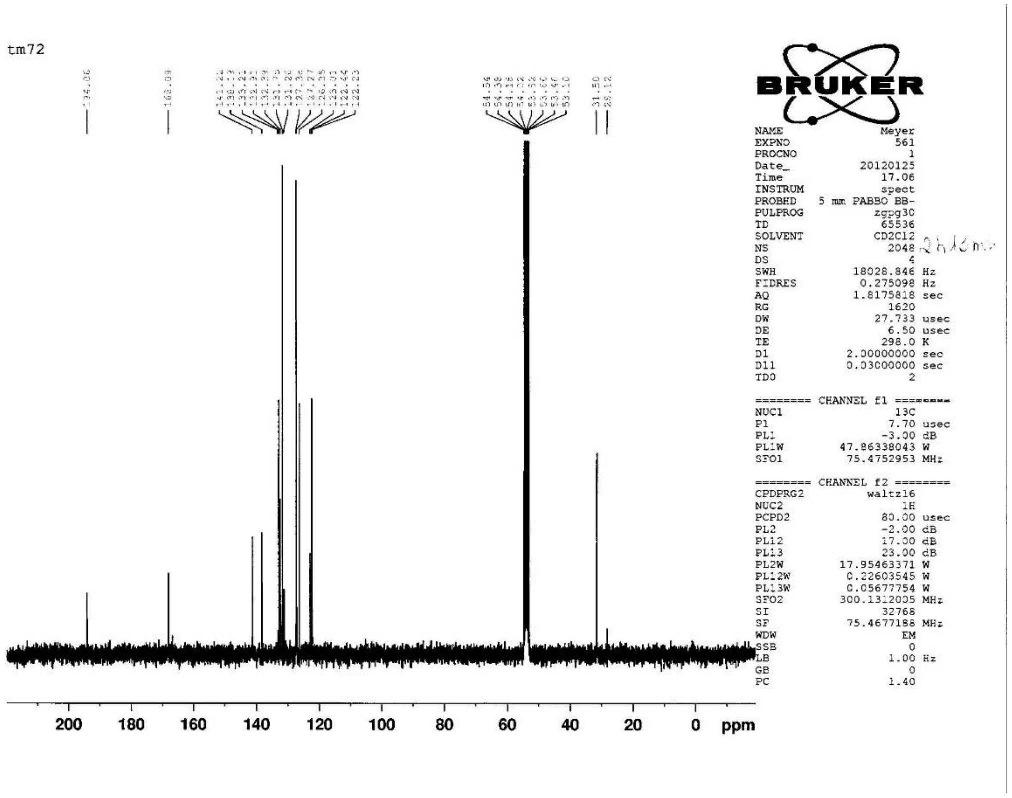

^{13}C NMR (75 MHz, CD_2Cl_2) of compound **8f**.

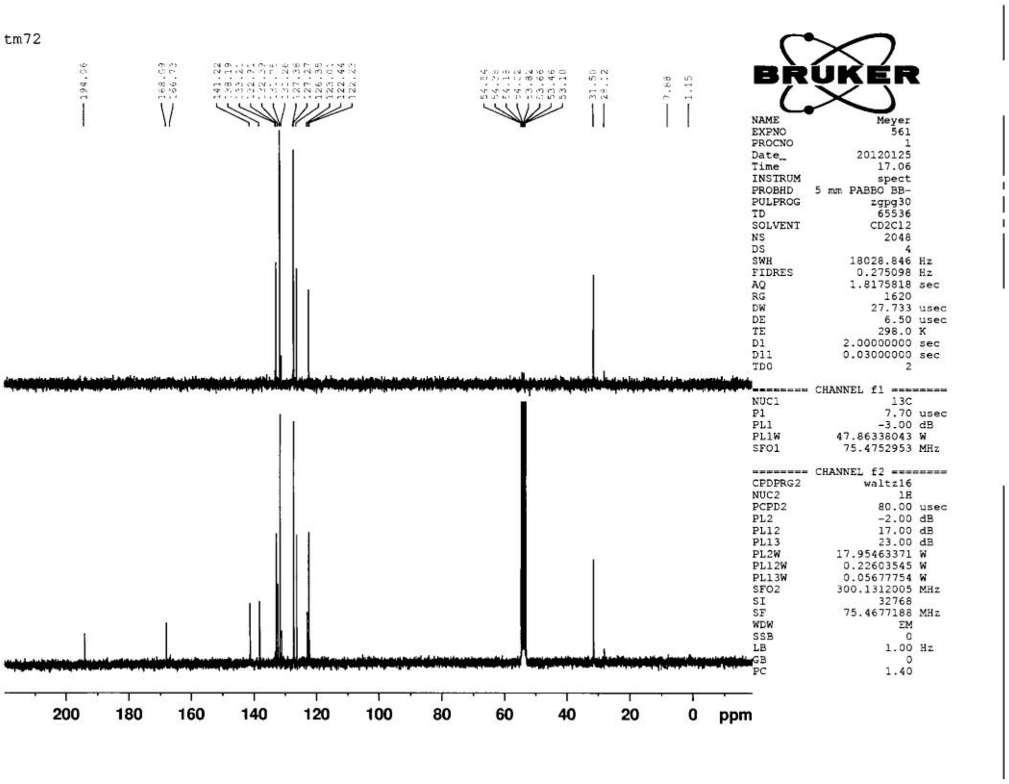

DEPT ^{13}C NMR (75 MHz, CD_2Cl_2) of compound **8f**.

3.7. 2-[4-(1-Methyl-1*H*-pyrazol-4-yl)benzylidene]-1*H*-inden-1,3[2*H*]-dione (8g)

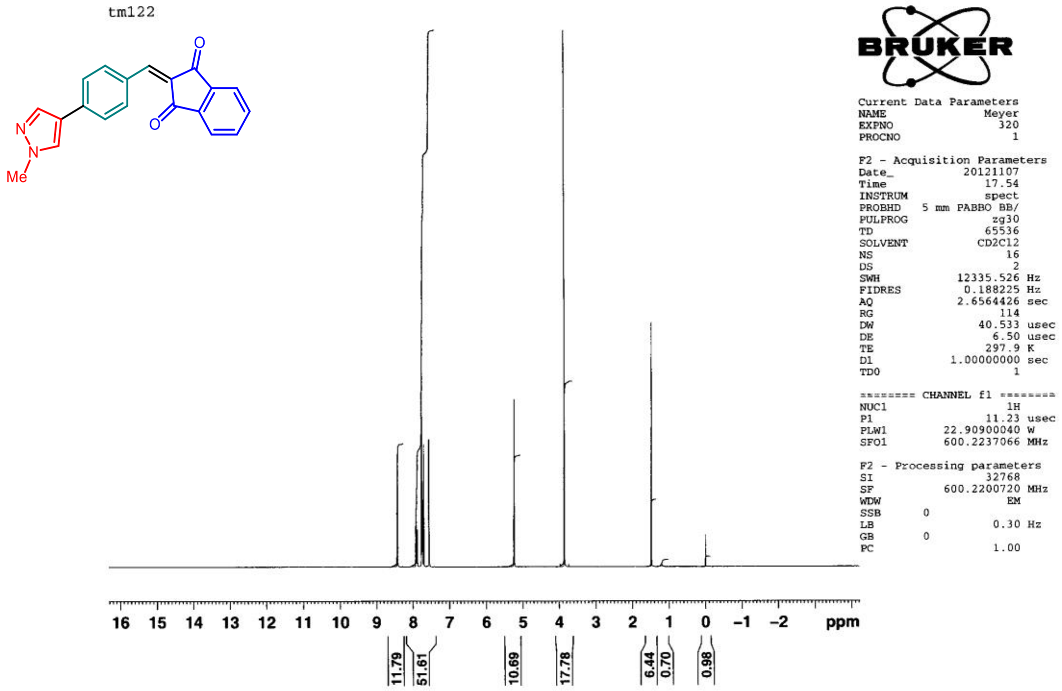

^1H NMR (600 MHz, CD_2Cl_2) of compound **8g**.

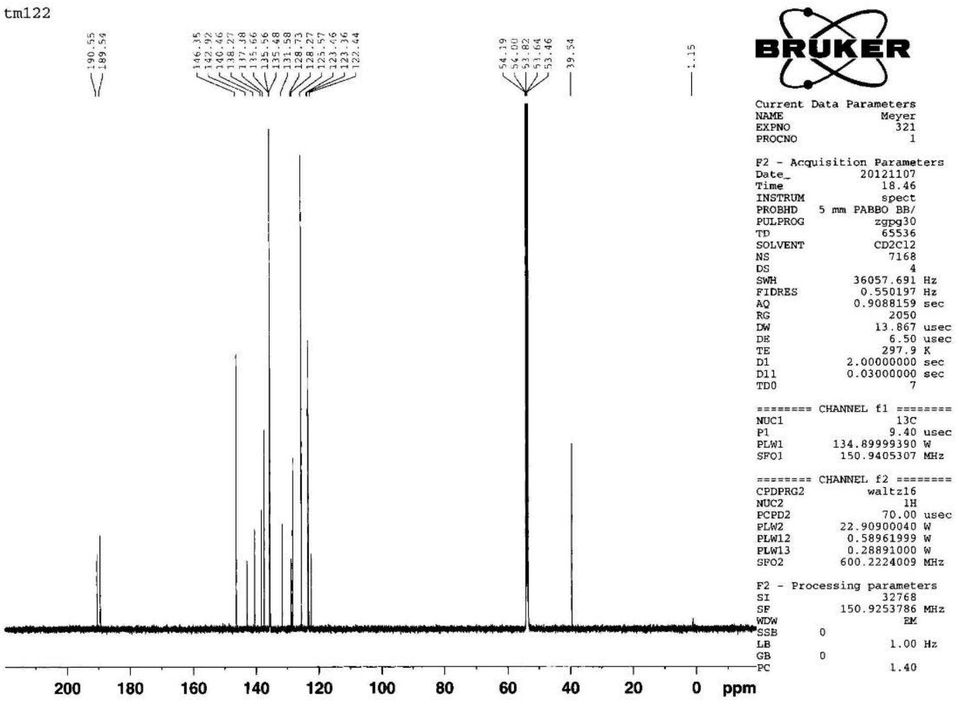

^{13}C NMR (150 MHz, CD_2Cl_2) of compound **8g**.

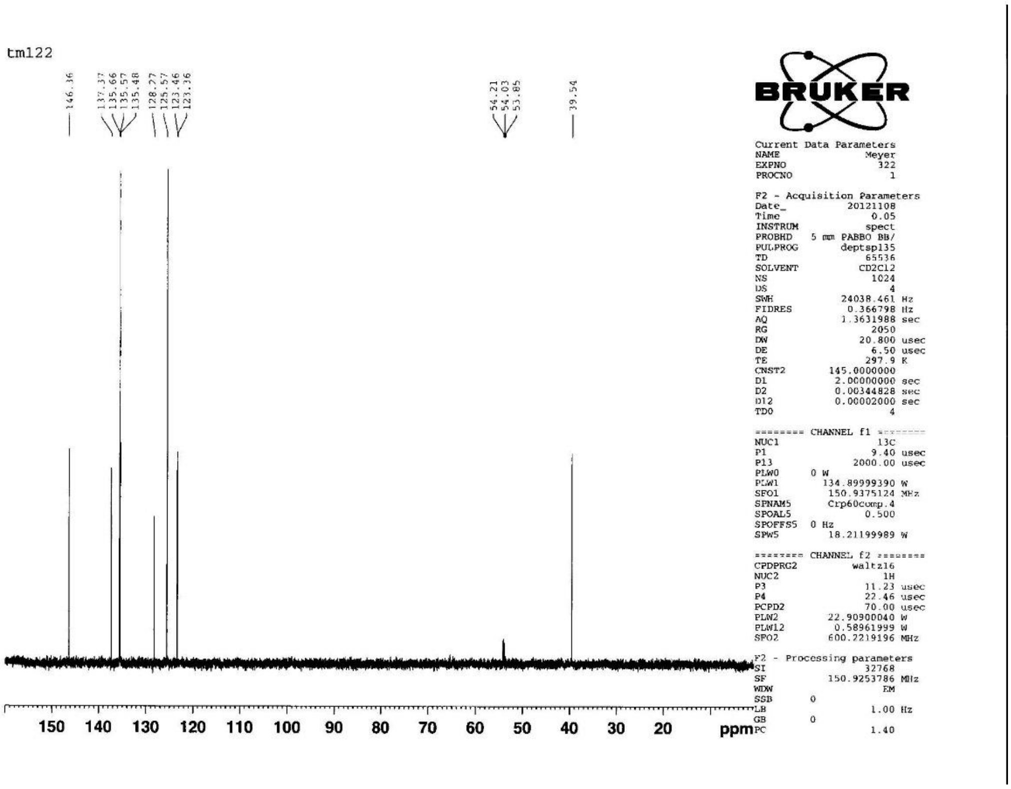

DEPT ^{13}C NMR (150 MHz, CD_2Cl_2) of compound **8g**.

3.8. 5-[4-(10-Hexyl-10*H*-phenothiazin-3-yl)benzylidene]-3-methyl-2-thioxothiazolidin-4-one (8h)

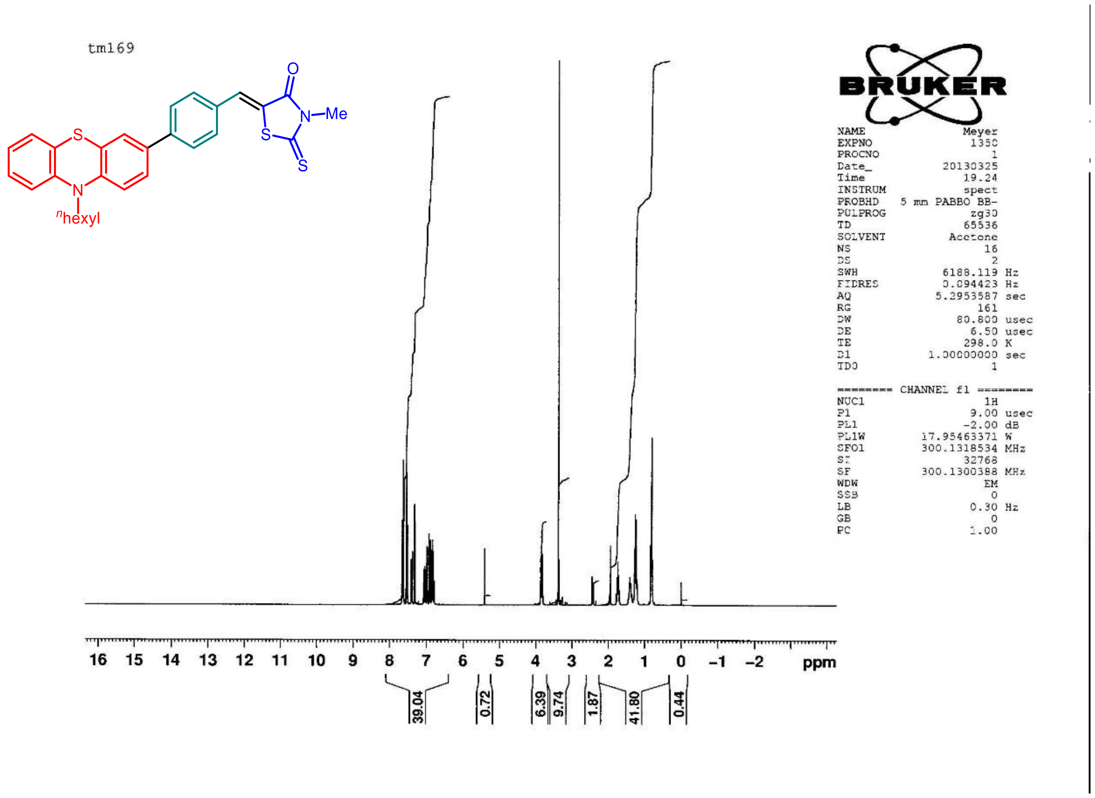

^{1}H NMR (300 MHz, acetone-d$_6$/CS_2 4:1) of compound **8h**.

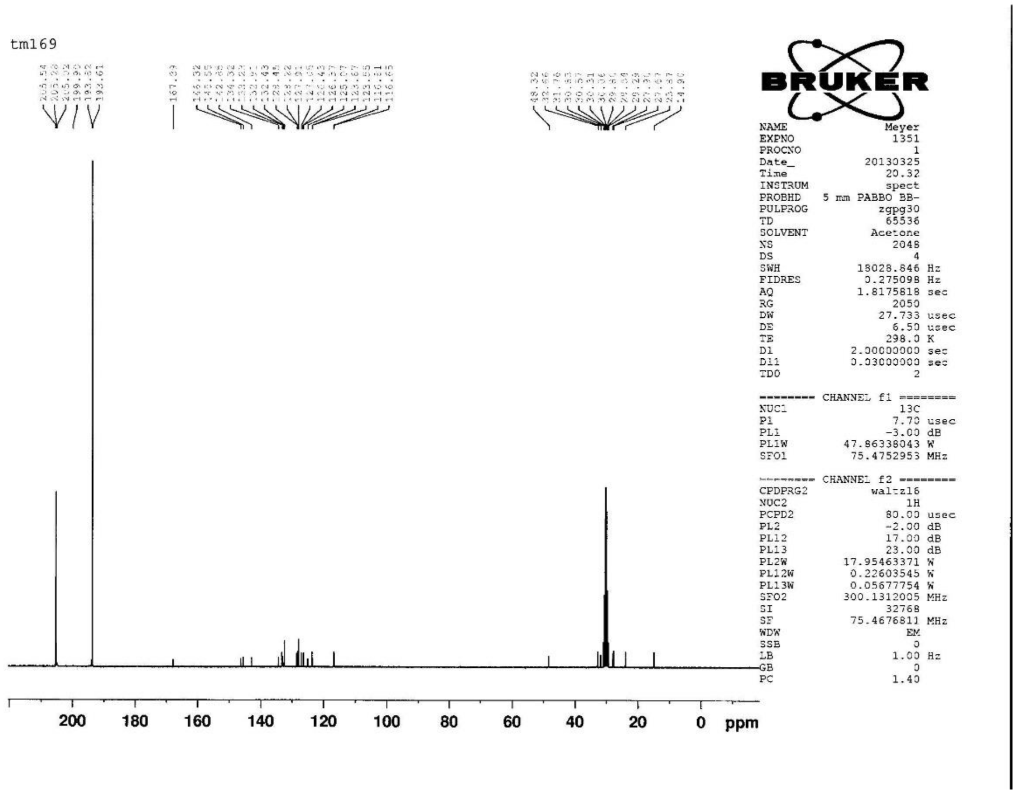

^{13}C NMR (75 MHz, acetone-d$_6$/CS_2 4:1) of compound **8h**.

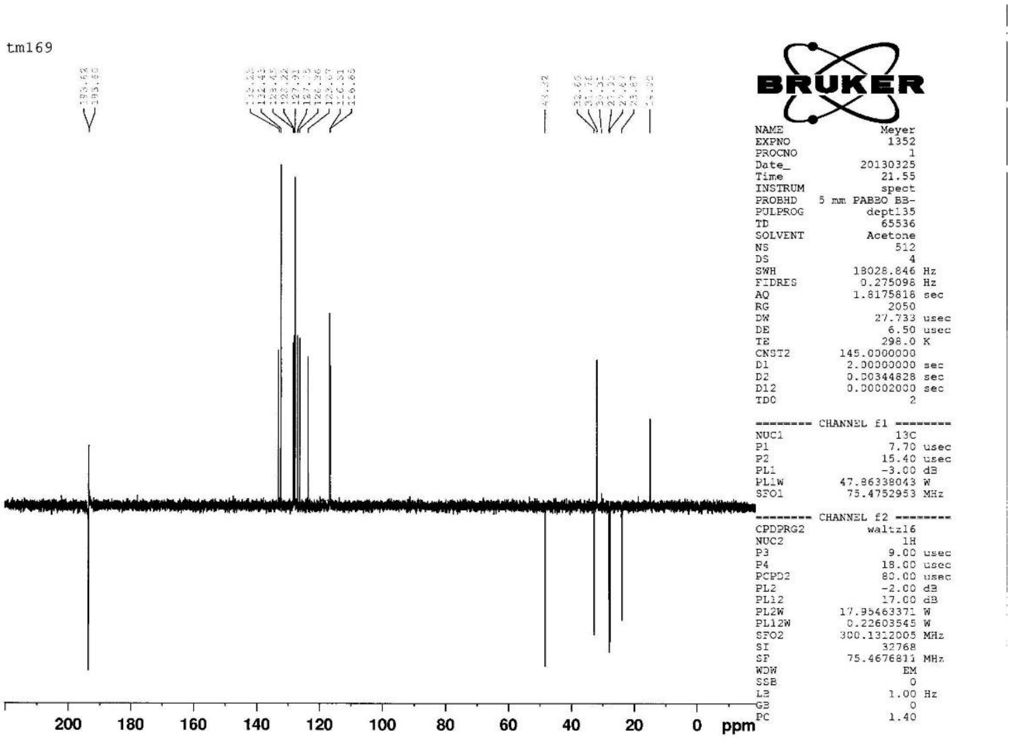

DEPT ^{13}C NMR (75 MHz, acetone-d_6/CS_2 4:1) of compound **8h**.

3.9. 4-[4-(10-Hexyl-10*H*-phenothiazin-3-yl)benzylidene]-3-methyl-1-phenyl-1*H*-pyrazol-5[4*H*]-one (8i)

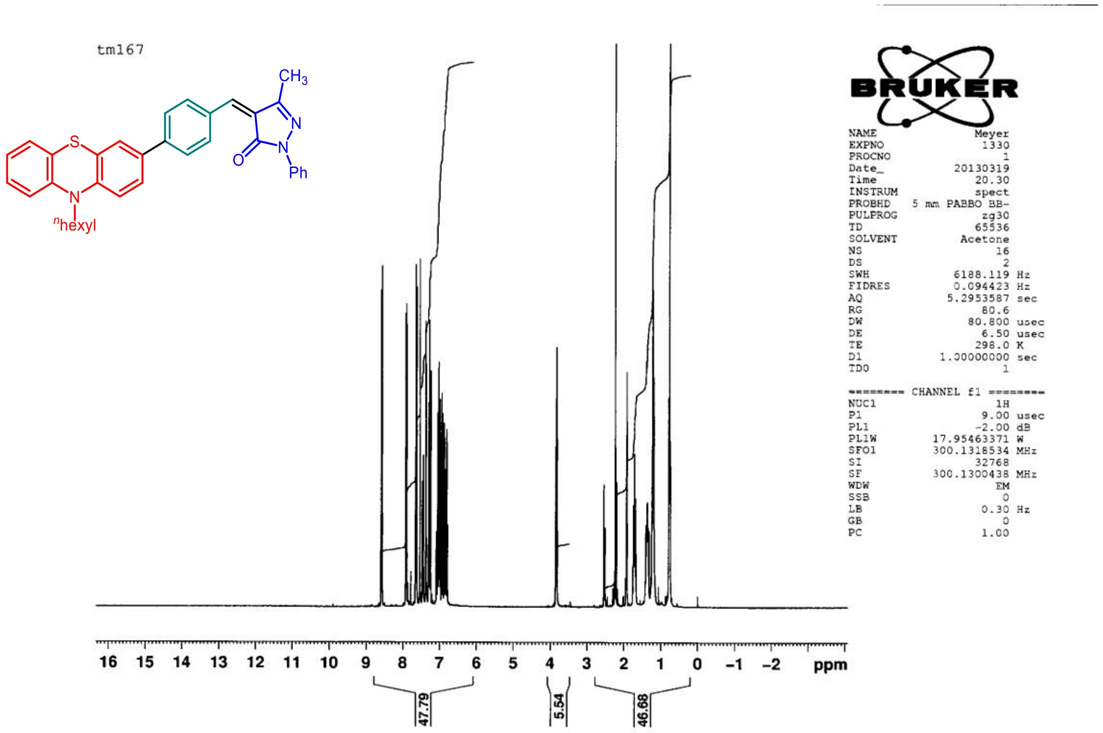

^{1}H NMR (300 MHz, acetone-d_6/CS_2 4:1) of compound **8i**.

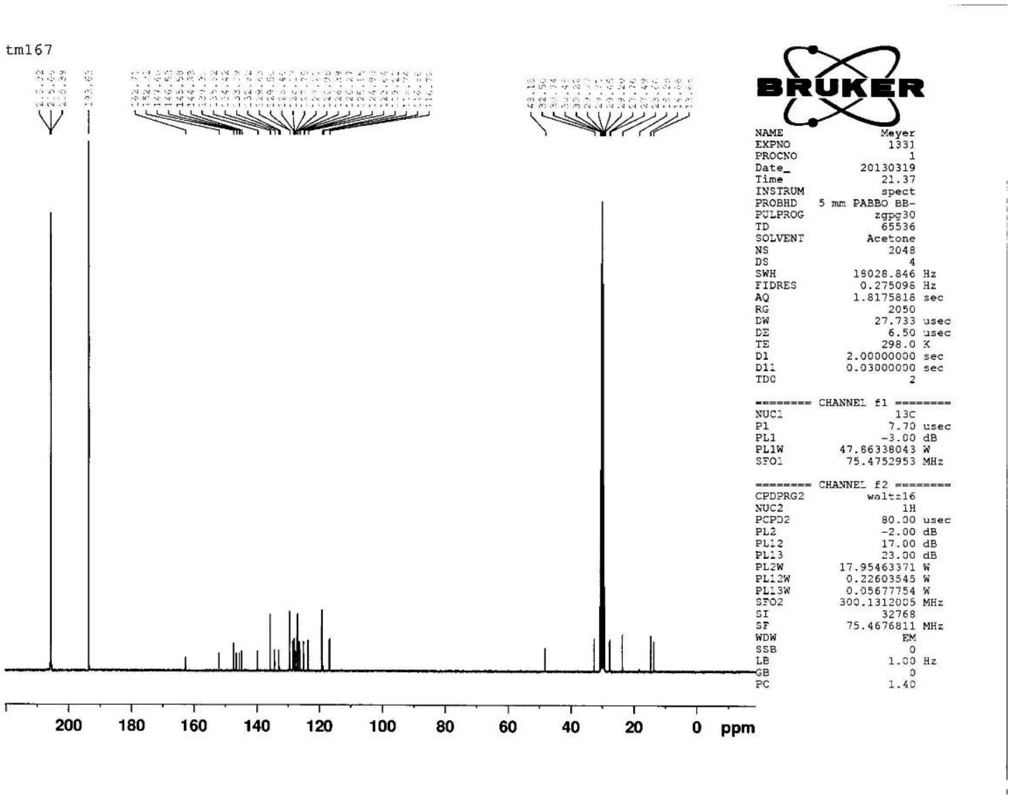

^{13}C NMR (75 MHz, acetone-d_6/CS_2 4:1) of compound **8i**.

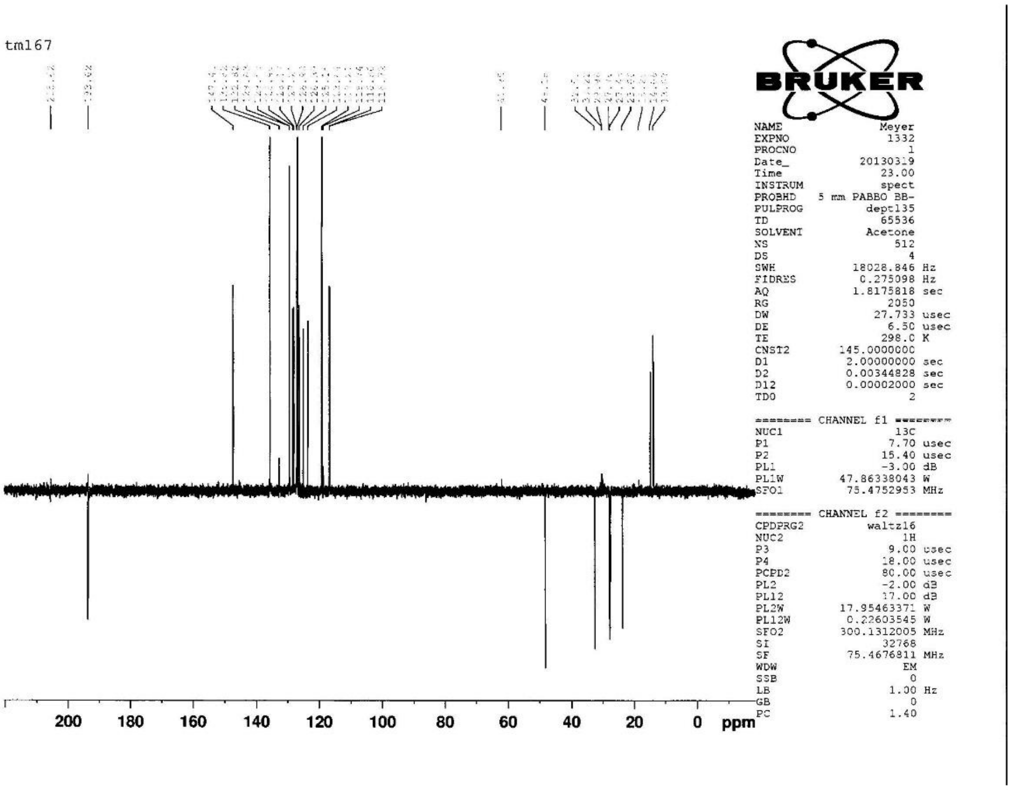

DEPT ^{13}C NMR (75 MHz, acetone-d_6/CS_2 4:1) of compound **8i**.

3.10. 3-Methyl-2-thioxo-5-{[5-(*p*-tolyl)thiophen-2-yl]methylene}thiazolidin-4-one (9a)

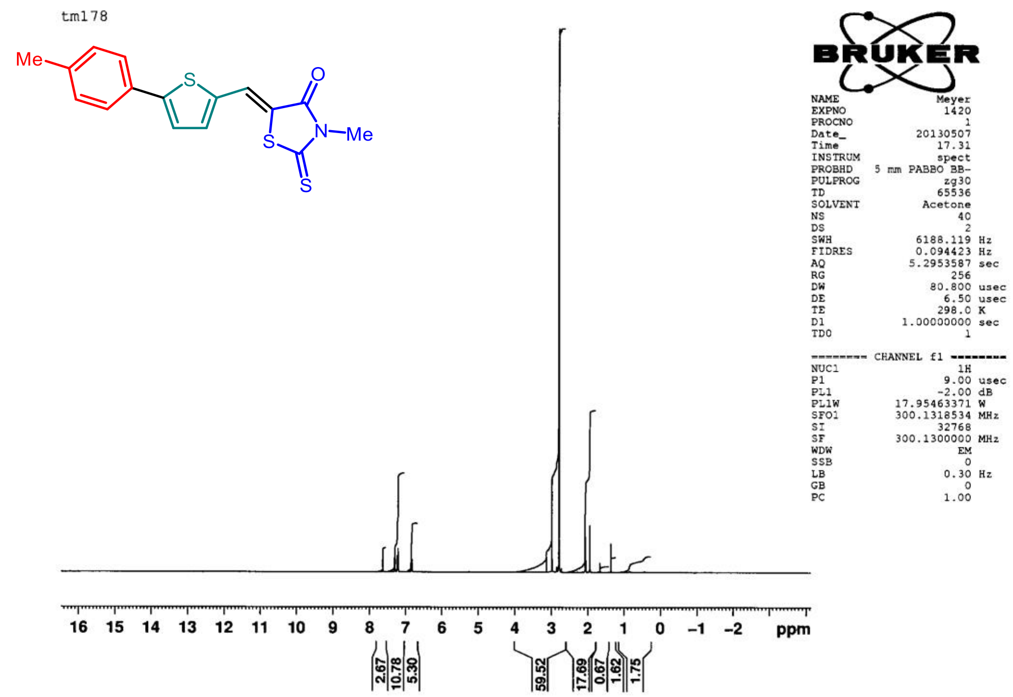

^{1}H NMR (300 MHz, DMSO-d_6/CS_2 8:1) of compound **9a**.

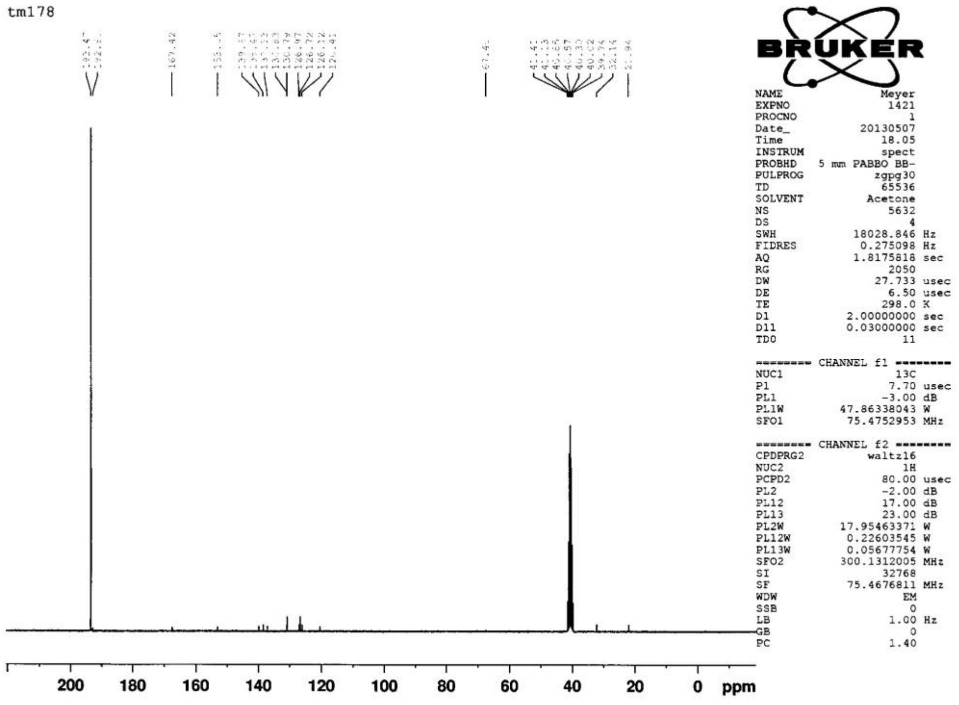

^{13}C NMR (75 MHz, DMSO-d_6/CS_2 8:1) of compound **9a**.

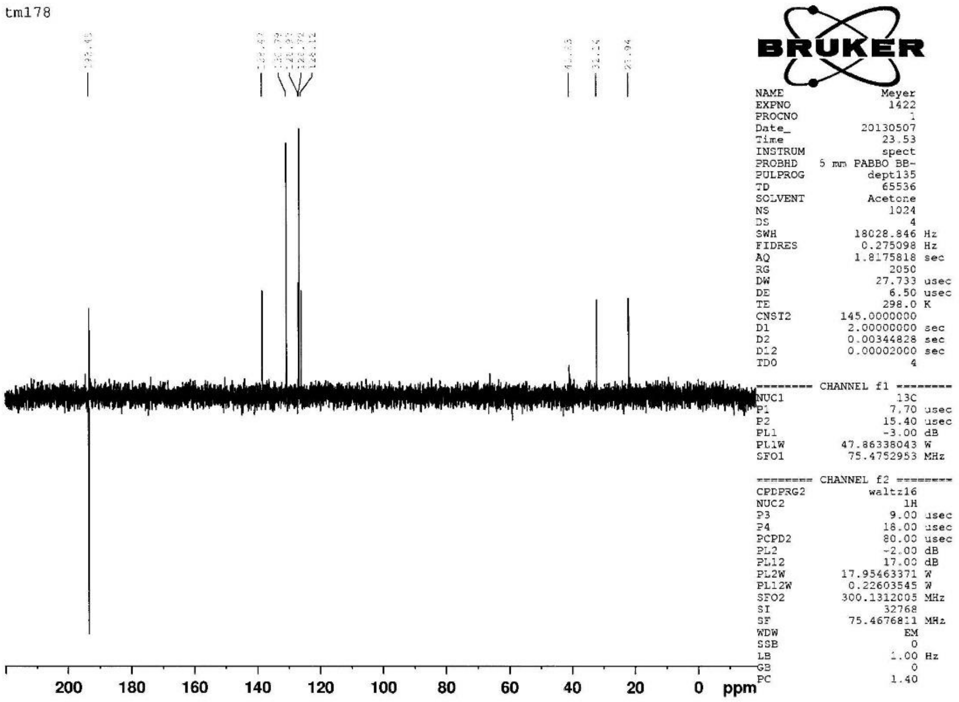

DEPT ^{13}C NMR (75 MHz, DMSO-d_6/CS_2 8:1) of compound **9a**.

3.11. 2-{[5-(*p*-Tolyl)thiophen-2-yl]methylene}-1*H*-inden-1,3[2*H*]-dione (9b)

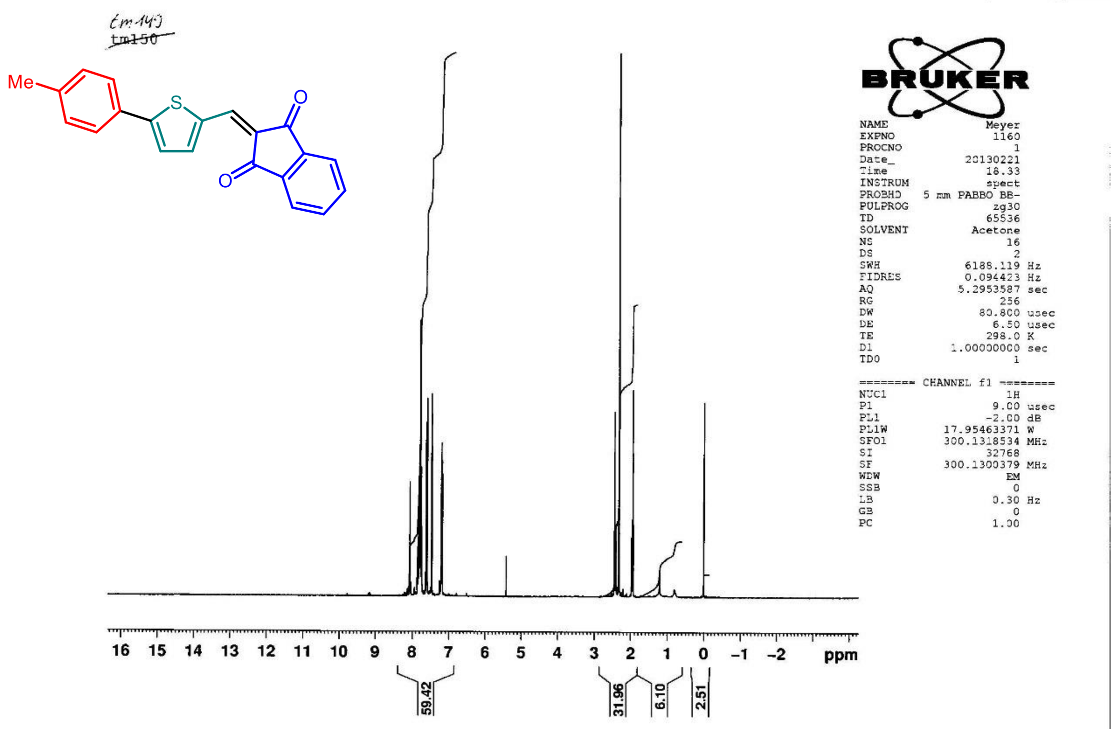

^{1}H NMR (300 MHz, acetone-d_6/CS_2 4:1) of compound **9b**.

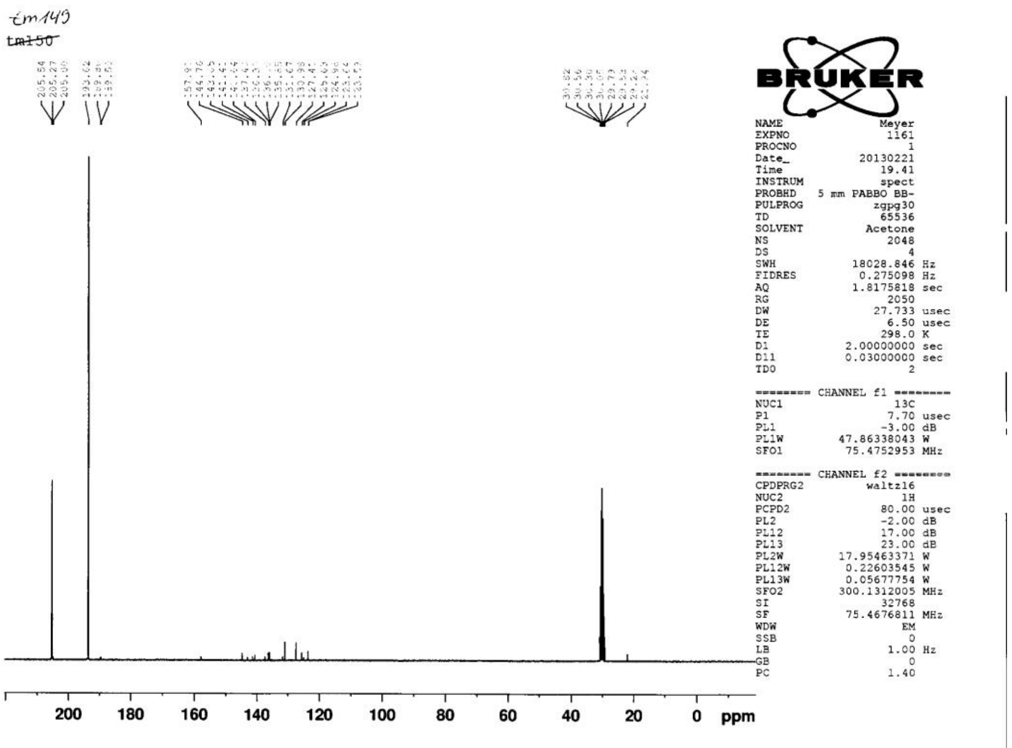

^{13}C NMR (75 MHz, acetone-d_6/CS_2 4:1) of compound **9b**.

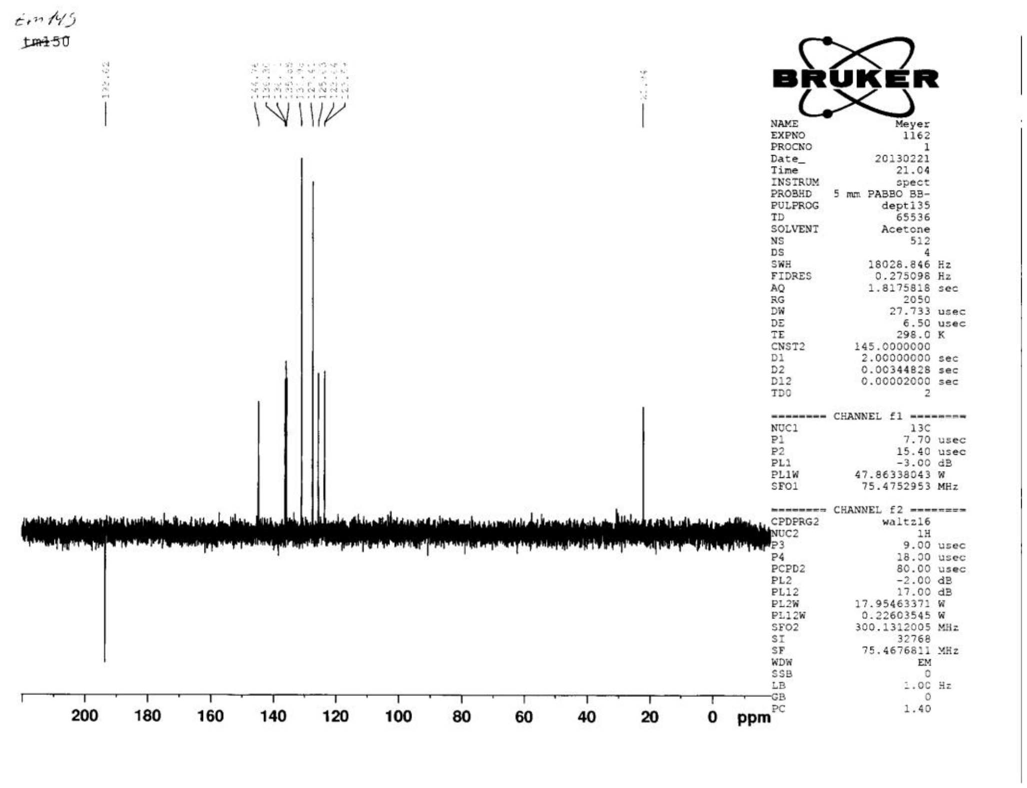

DEPT ^{13}C NMR (75 MHz, acetone-d_6/CS_2 4:1) of compound **9b**.

3.12. 1,3-Dipropyl-5-{[5-(*p*-tolyl)thiophen-2-yl]methylene}pyrimidin-2,4,6[1*H*,3*H*,5*H*]-trione (9c)

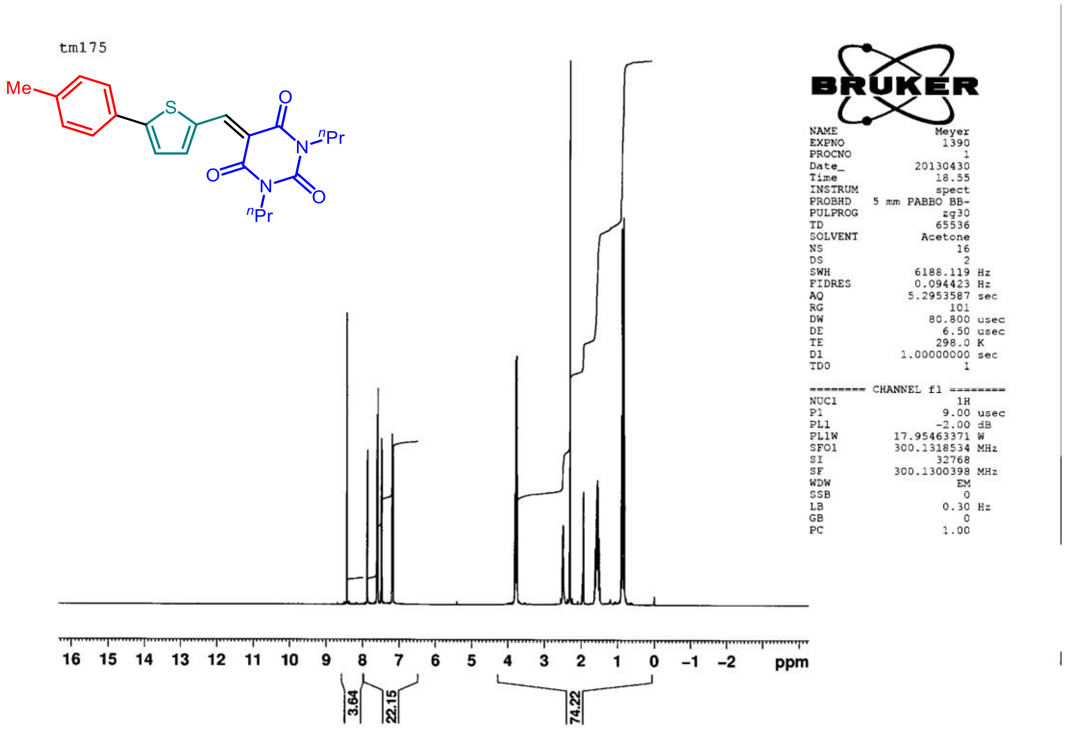

^{1}H NMR (300 MHz, acetone-d_6/CS_2 4:1) of compound **9c**.

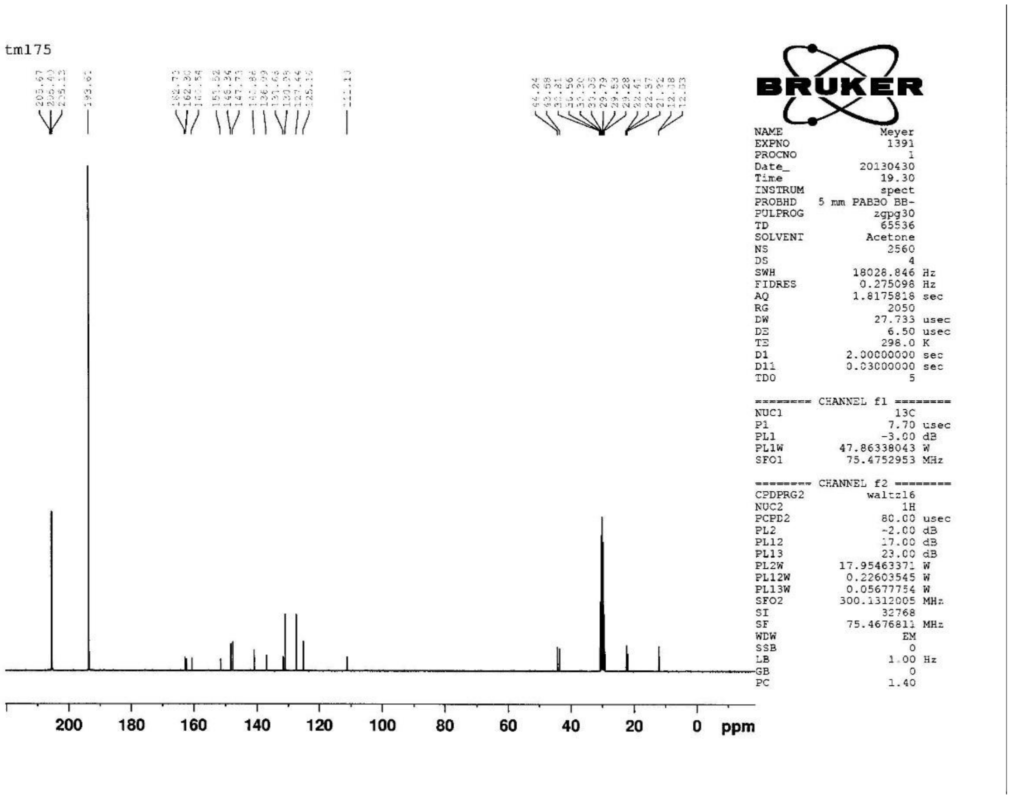

^{13}C NMR (300 MHz, acetone-d_6/CS_2 4:1) of compound **9c**.

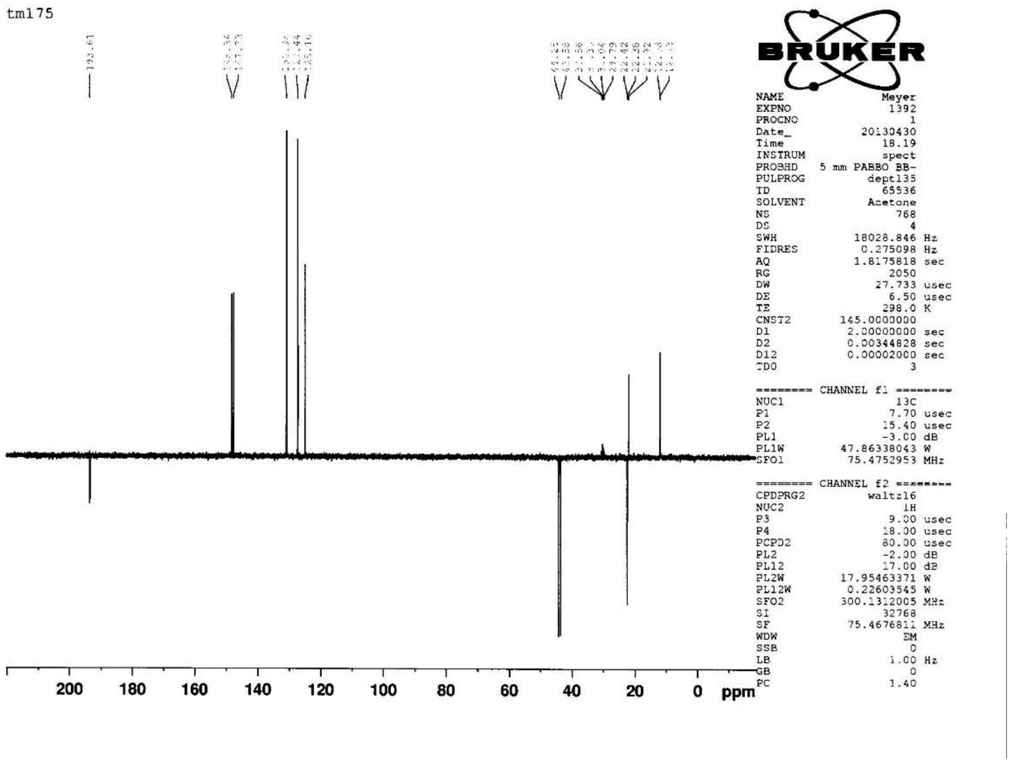

DEPT ^{13}C NMR (300 MHz, acetone-d_6/CS_2 4:1) of compound **9c**.

3.13. 3-Methyl-1-phenyl-4-{[5-(*p*-tolyl)thiophen-2-yl]methylene}-1H-pyrazol-5[4*H*]-one (9d)

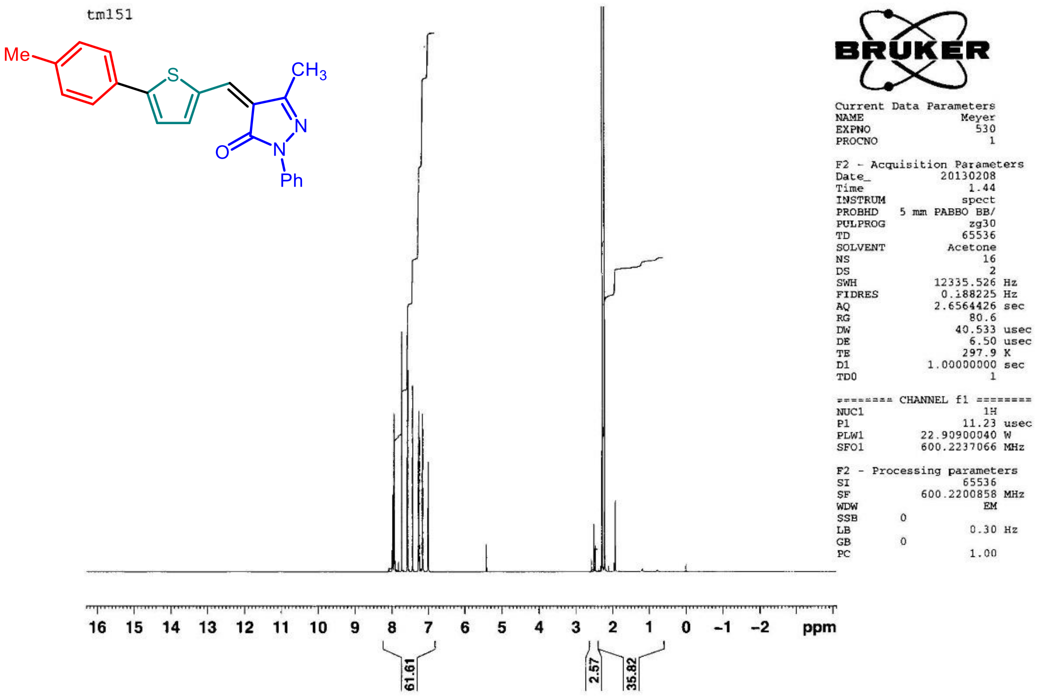

^{1}H NMR (600 MHz, acetone-d_6/CS_2) of compound **9d**.

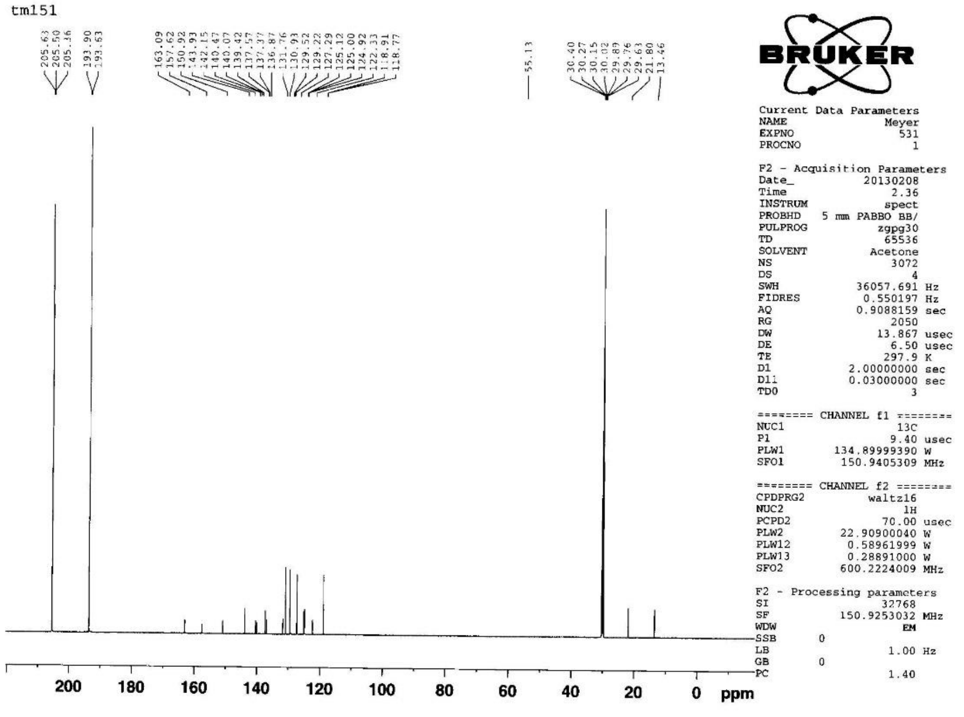

^{13}C NMR (150 MHz, acetone-d_6/CS_2) of compound **9d**.

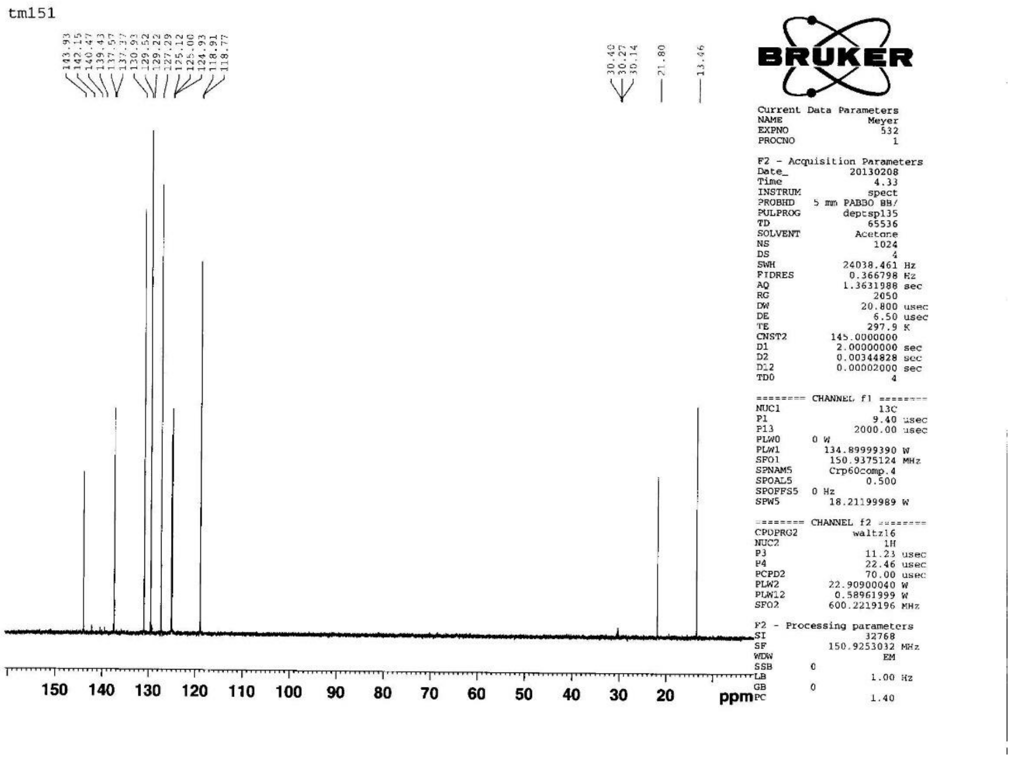

DEPT ^{13}C NMR (150 MHz, acetone-d_6/CS_2) of compound **9d**.

3.14. 2-(4-Nitrophenyl)-3-[5-(*p*-tolyl)thiophen-2-yl]acrylonitrile (9e)

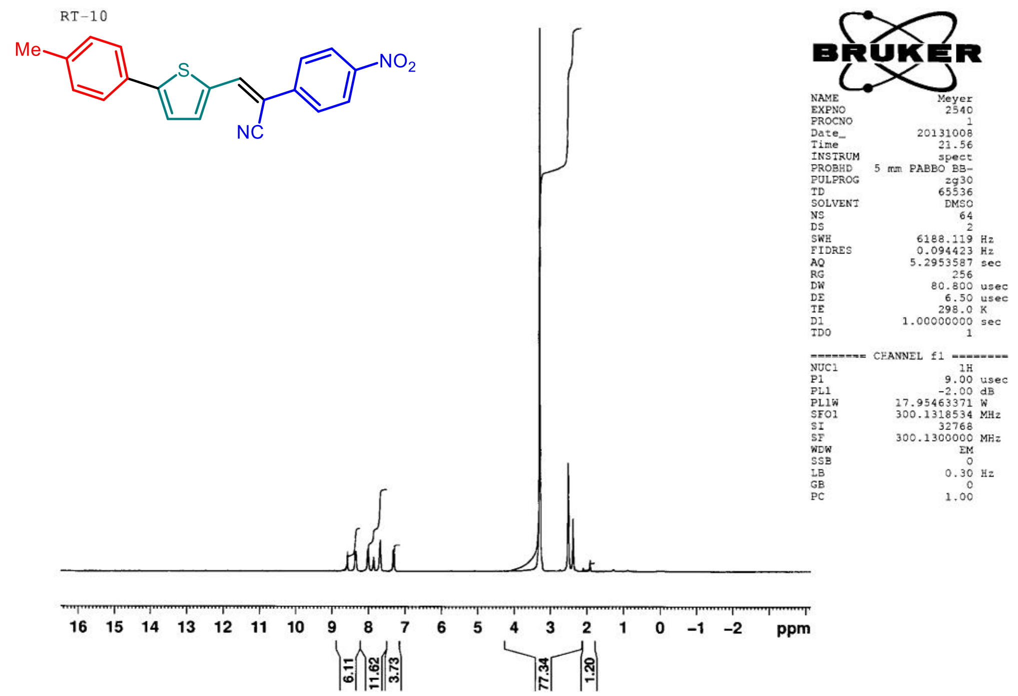

^{1}H NMR (300 MHz, DMSO-d$_6$) of compound **9e**.

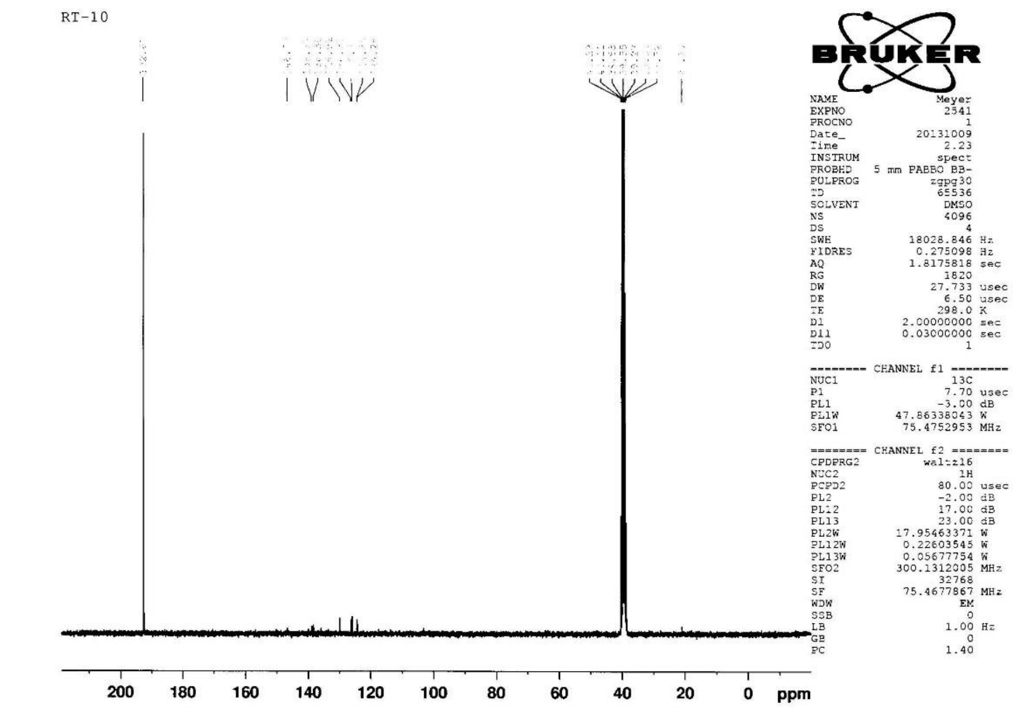

^{13}C NMR (75 MHz, DMSO-d$_6$) of compound **9e**.

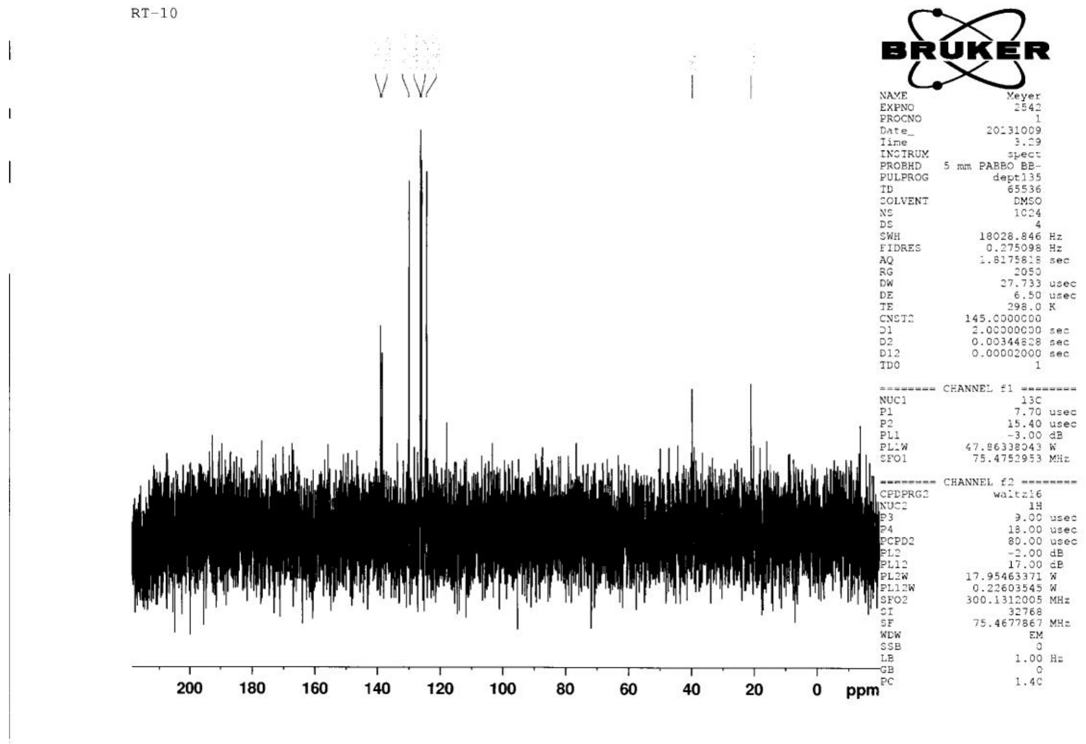

DEPT ^{13}C NMR (75 MHz, DMSO-d_6) of compound **9e**.

3.15. 5-{[5-(4-{Diphenylamino}phenyl)thiophen-2-yl]methylene}-3-methyl-2-thioxothiazolidin-4-one (9f)

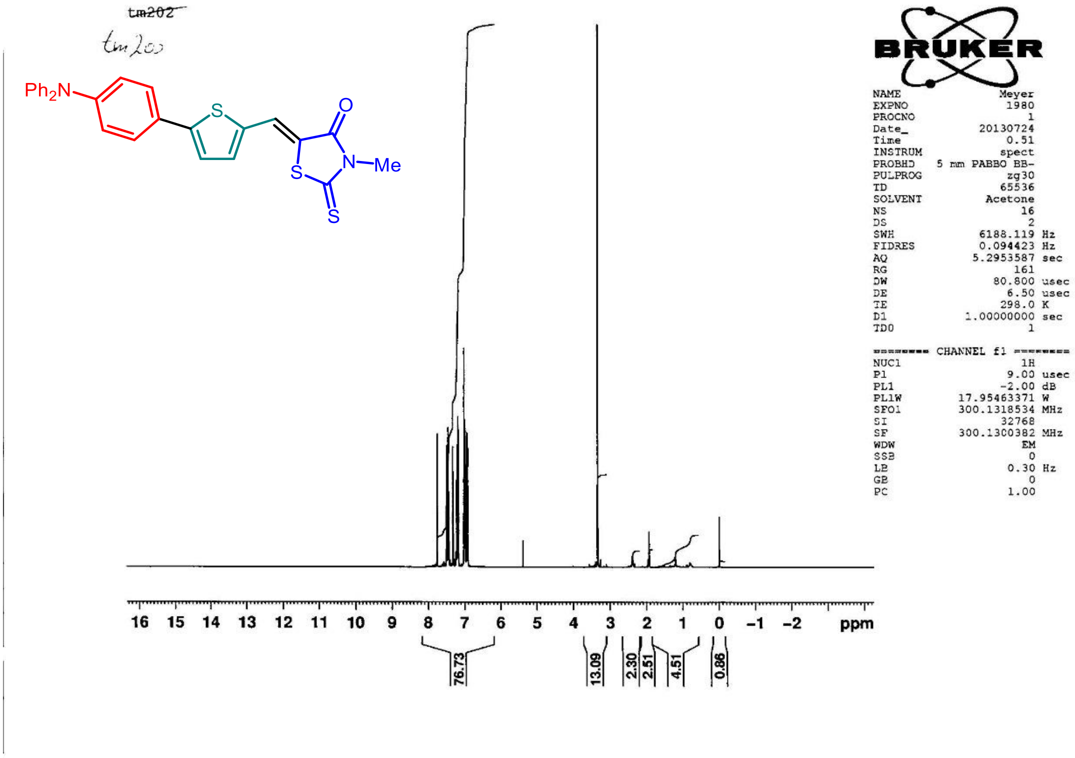

^{1}H NMR (300 MHz, acetone-d_6/CS_2 4:1) of compound **9f**.

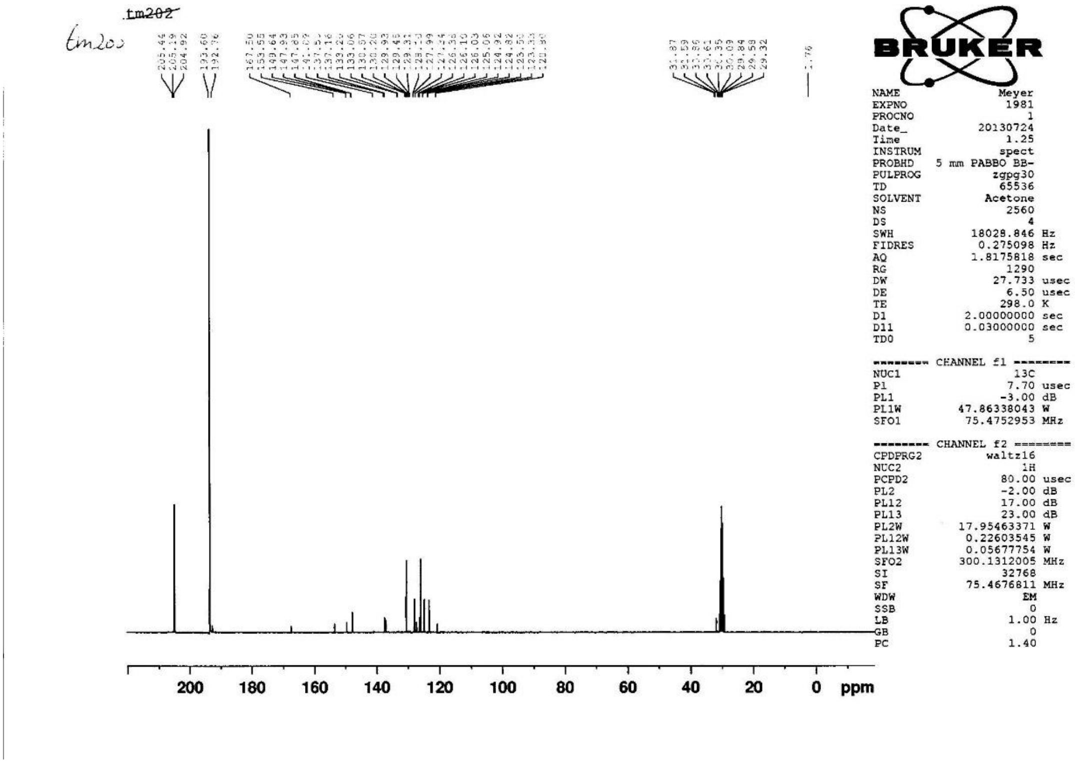

^{13}C NMR (75 MHz, acetone-d_6/CS_2 4:1) of compound **9f**.

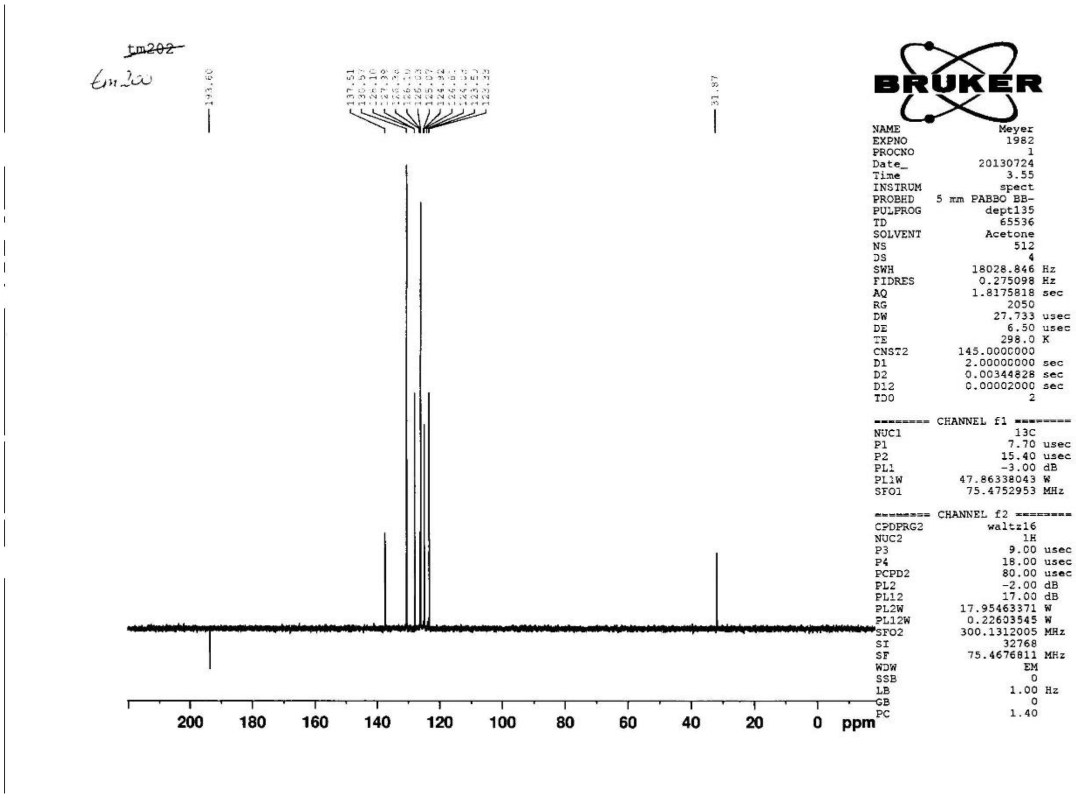

DEPT ^{13}C NMR (75 MHz, acetone-d_6/CS_2 4:1) of compound **9f**.

3.16. 4-{[5-(10-Hexyl-10*H*-phenothiazin-3-yl)thiophen-2-yl]methylene}-3-methyl-1-phenyl-1H-pyrazol-5[4*H*]-one (9g)

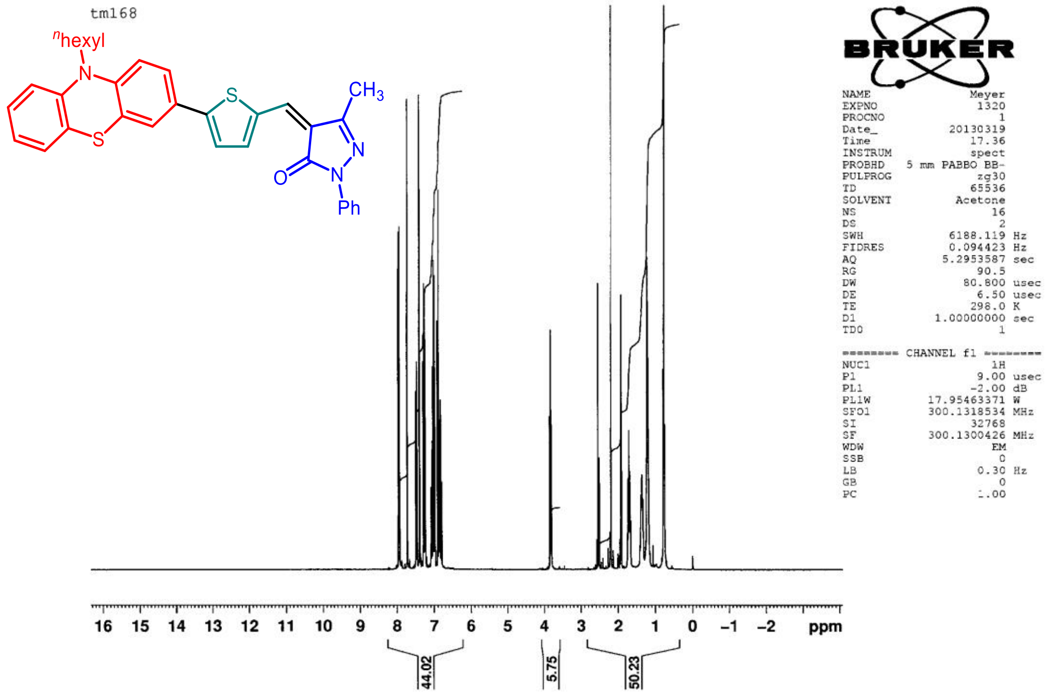

^{1}H NMR (300 MHz, acetone-d$_6$/CS$_2$ 4:1) of compound **9g**.

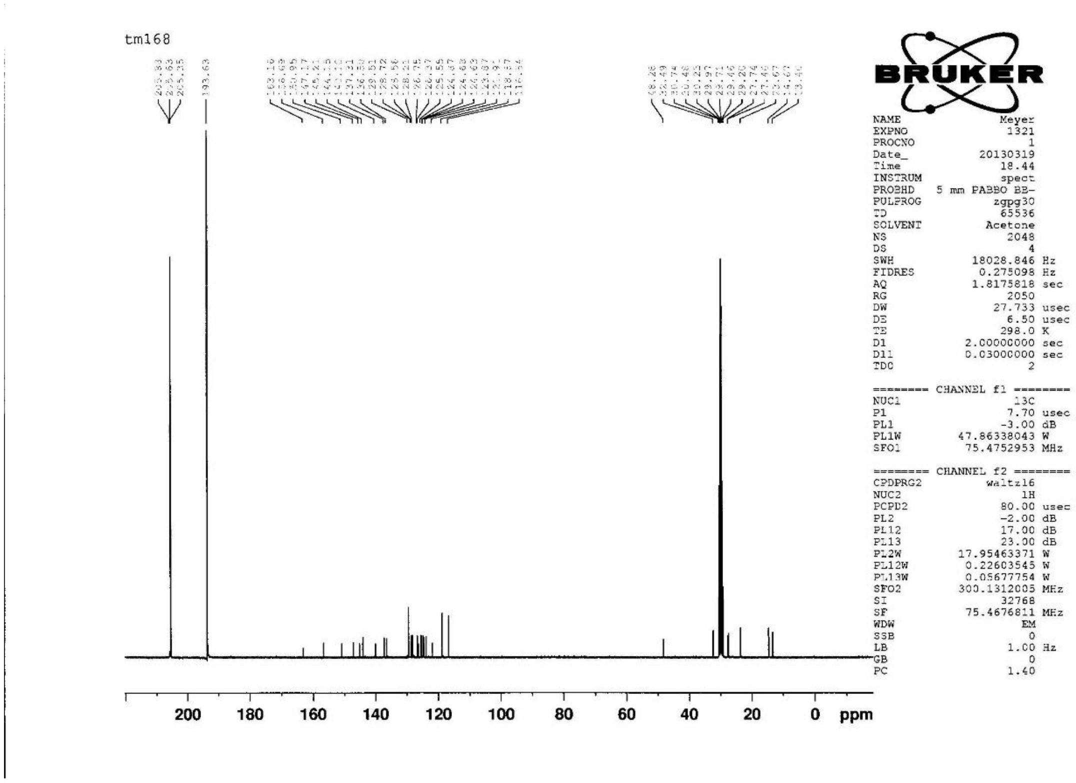

^{13}C NMR (75 MHz, acetone-d$_6$/CS$_2$ 4:1) of compound **9g**.

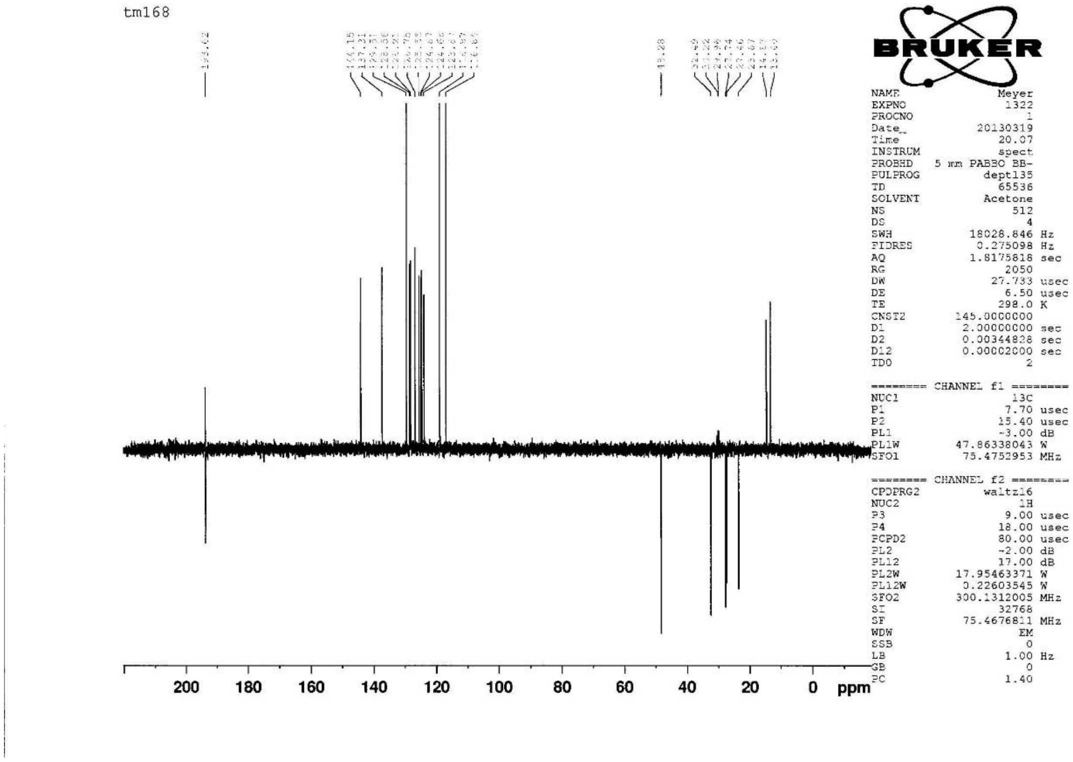

DEPT ^{13}C NMR (75 MHz, acetone-d_6/CS_2 4:1) of compound **9g**.

3.17. (*Z*)-5-methyl-4-((4-(octyloxy)-5-(*p*-tolyl)thiophen-2-yl)methylene)-2-phenyl-2,4-dihydro-3*H*-pyrazol-3-one (10a)

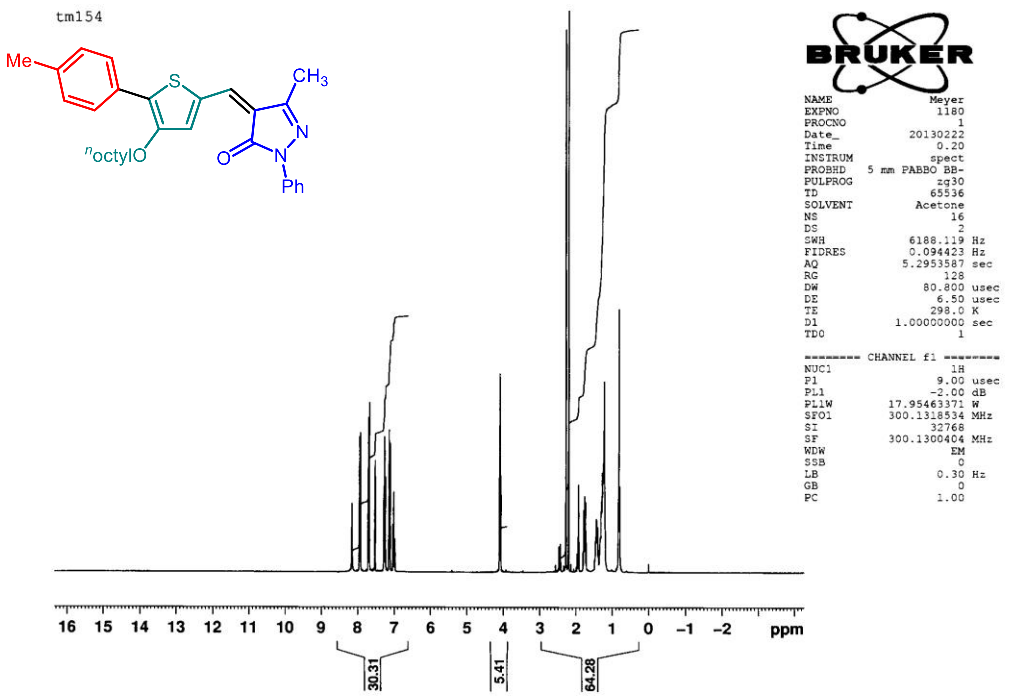

^{1}H NMR (300 MHz, acetone-d_6/CS_2 4:1) of compound **10a**.

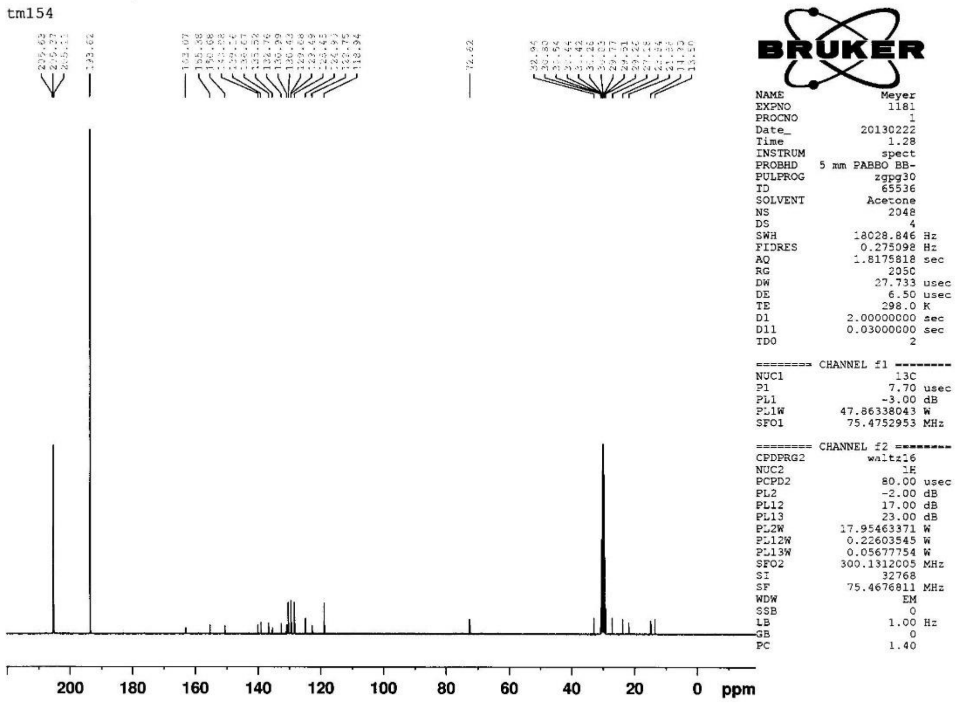

^{13}C NMR (75 MHz, acetone-d_6/CS_2 4:1) of compound **10a**.

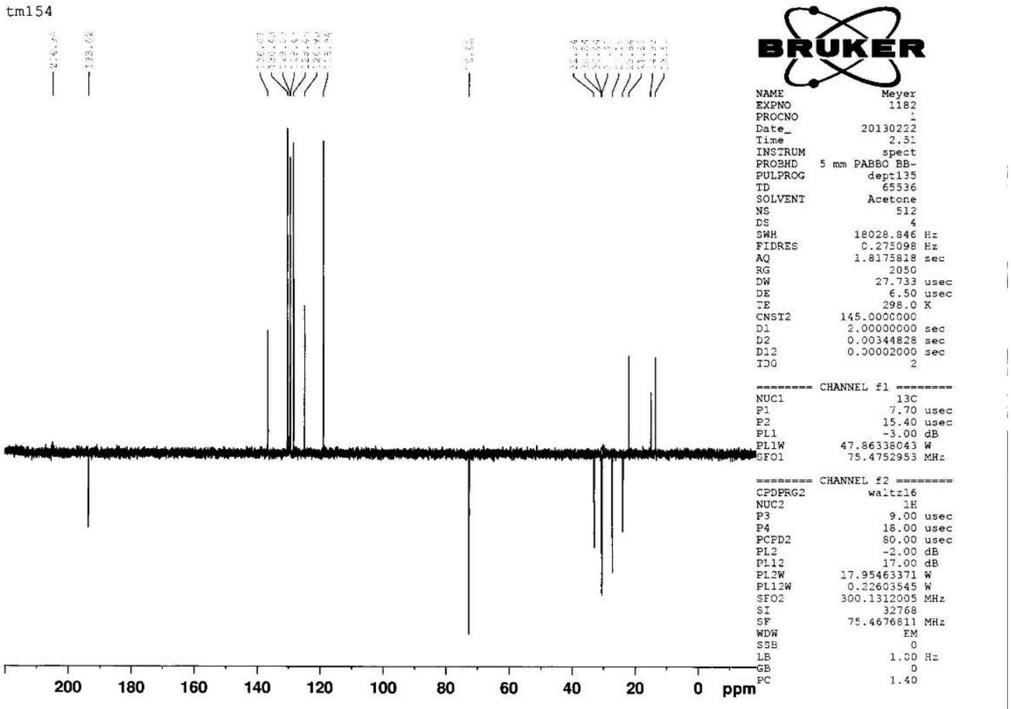

DEPT ^{13}C NMR (75 MHz, acetone-d_6/CS_2 4:1) of compound **10a**.

3.18. (*E*)-2-(3-cyano-5,5-dimethyl-4-(2-(4-(octyloxy)-5-(*p*-tolyl)thiophen-2-yl)vinyl)furan-2(5*H*)-ylidene)malononitrile (10b)

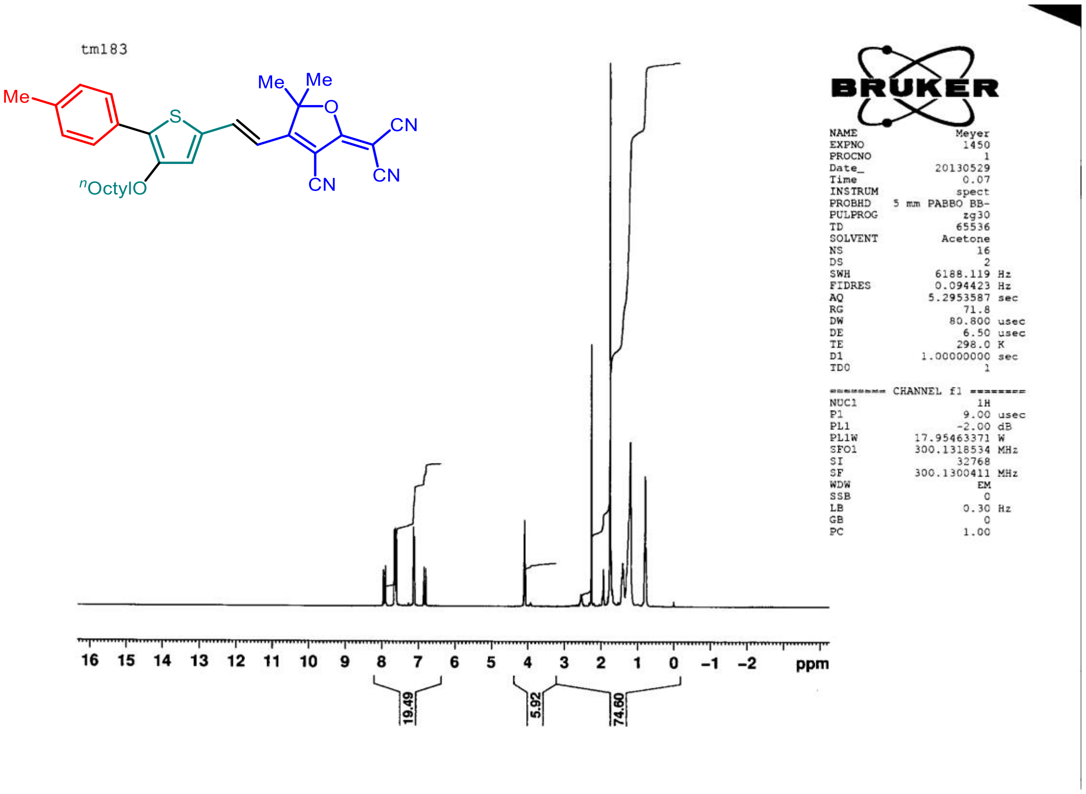

^{1}H NMR (300 MHz, acetone-d$_6$/CS$_2$ 4:1) of compound **10b**.

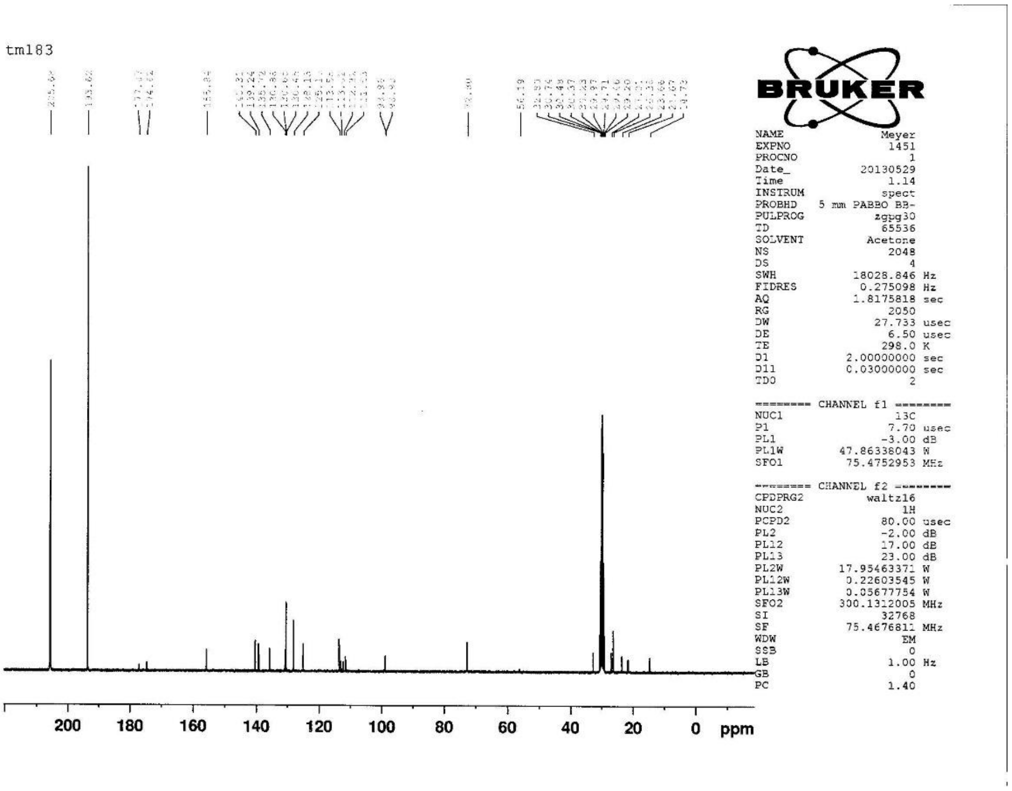

^{13}C NMR (75 MHz, acetone-d$_6$/CS$_2$ 4:1) of compound **10b**.

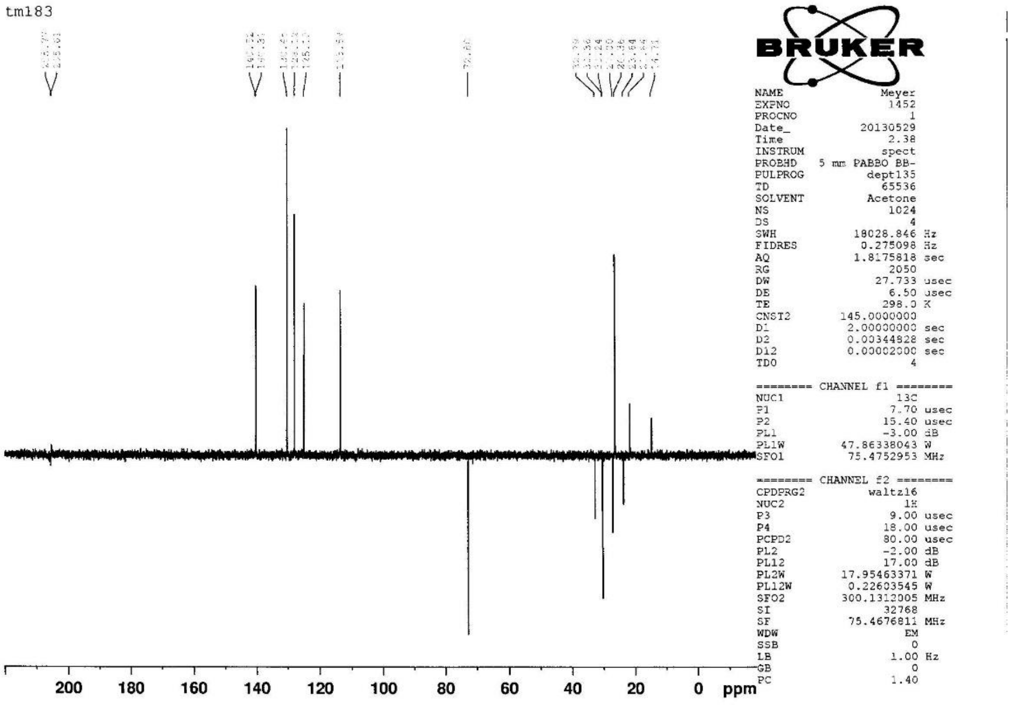

DEPT ^{13}C NMR (75 MHz, acetone-d_6/CS_2 4:1) of compound **10b**.

3.19. (*Z*)-5-((4-(octyloxy)-5-(4-(1,2,2-triphenylvinyl)phenyl)thiophen-2-yl)methylene)-2-thioxothiazolidin-4-one (10c)

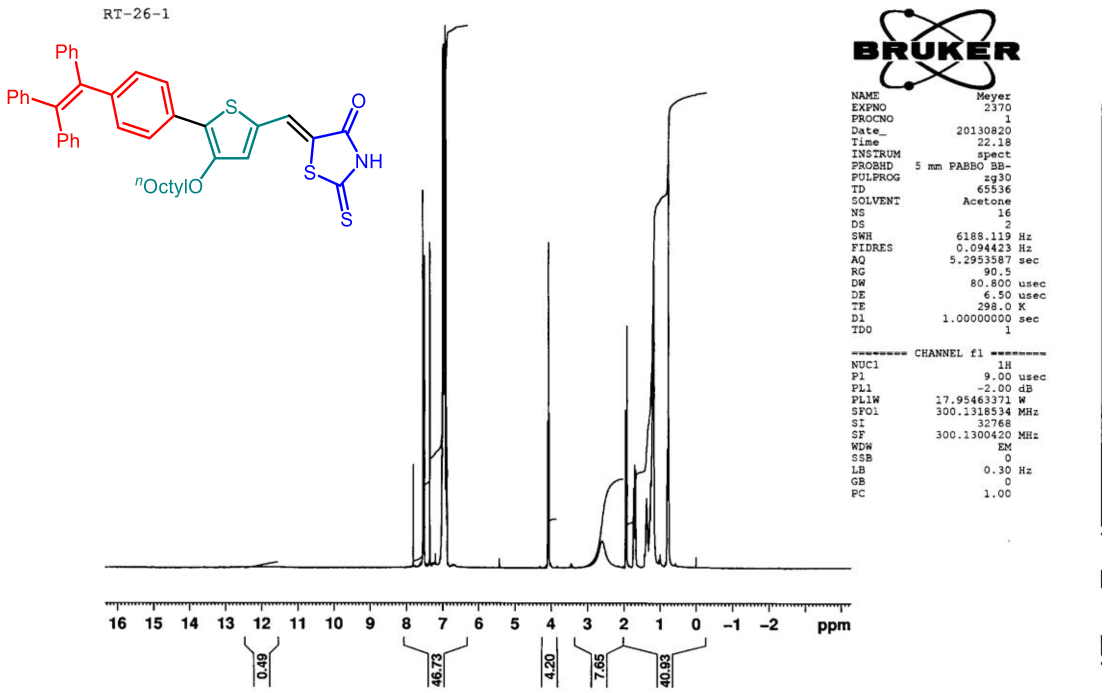

^{1}H NMR (300 MHz, acetone-d$_6$/CS$_2$ 4:1) of compound **10c**.

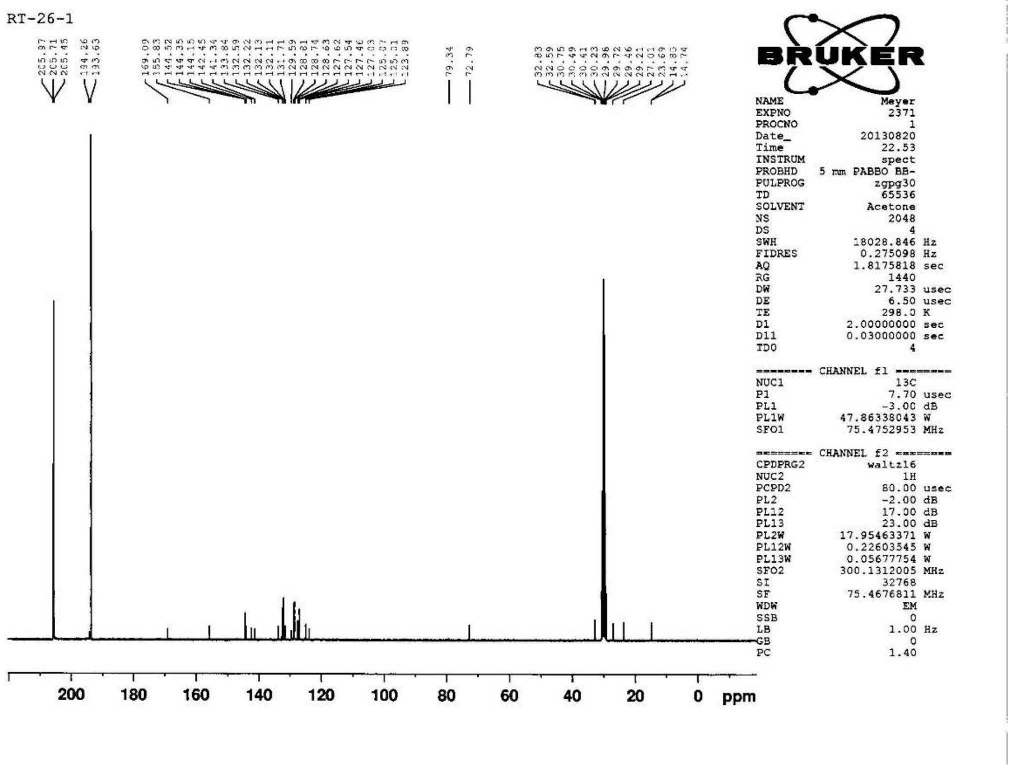

^{13}C NMR (75 MHz, acetone-d$_6$/CS$_2$ 4:1) of compound **10c**.

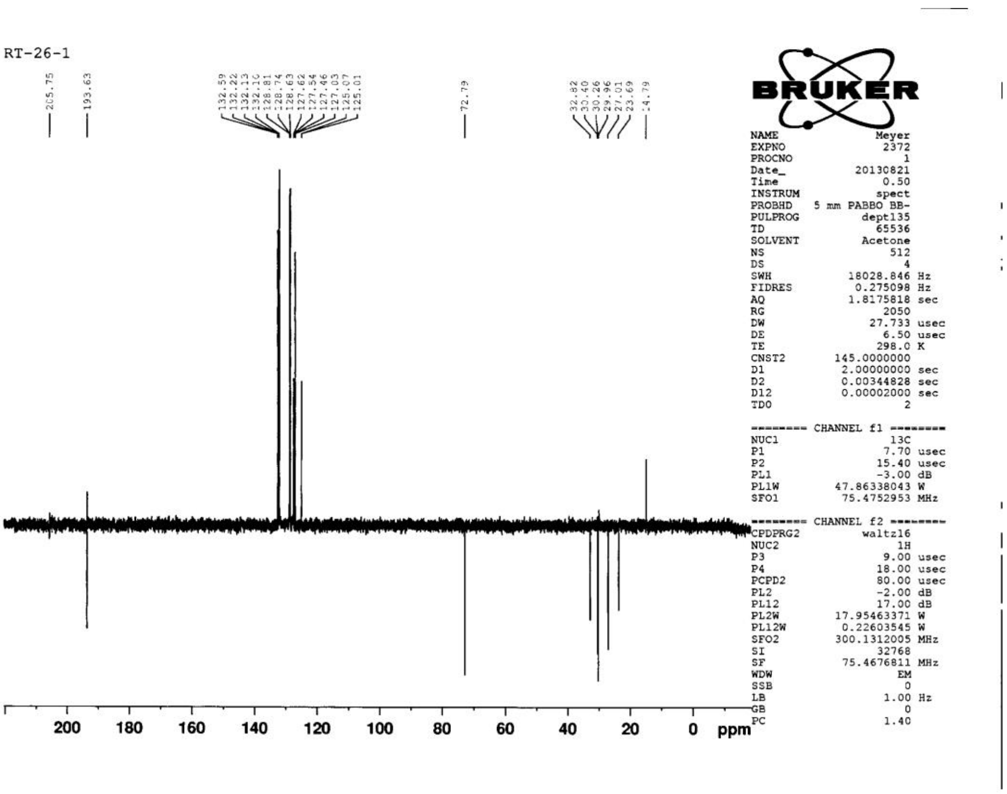

DEPT ^{13}C NMR (75 MHz, acetone-d_6/CS_2 4:1) of compound **10c**.

3.20. 5-{[5-(4-{Bis[4-methoxyphenyl]amino}phenyl)-3-(octyloxy)thiophen-2-yl]methylene}-2-thioxothiazolidin-4-one (10d)

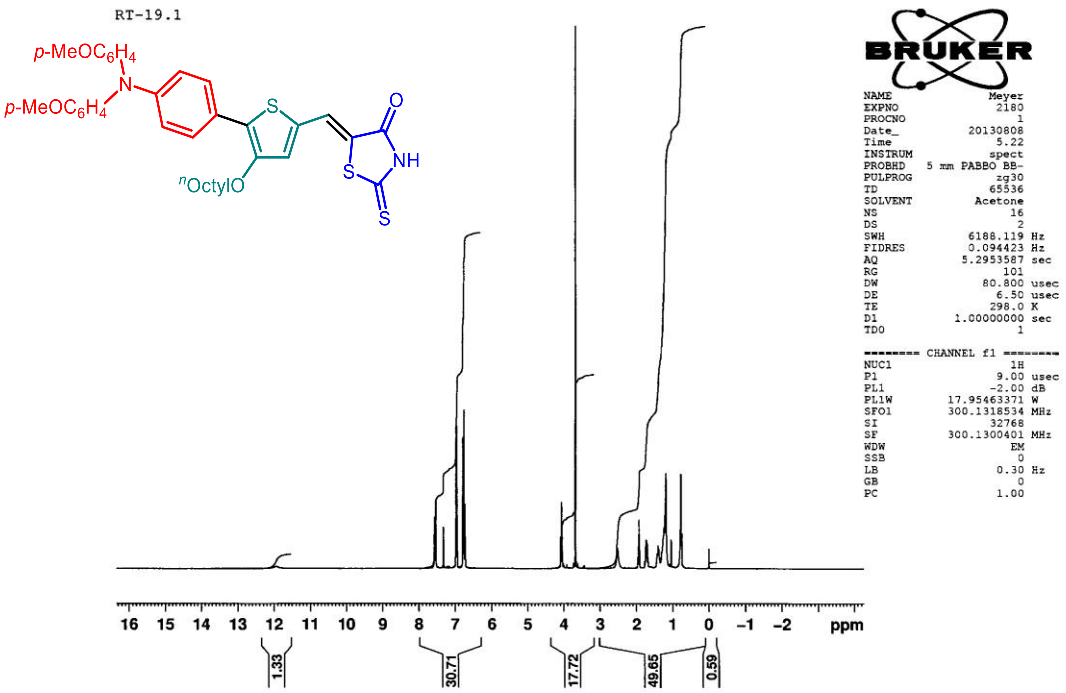

^{1}H NMR (300 MHz, acetone-d$_6$/CS$_2$ 4:1) of compound **10d**.

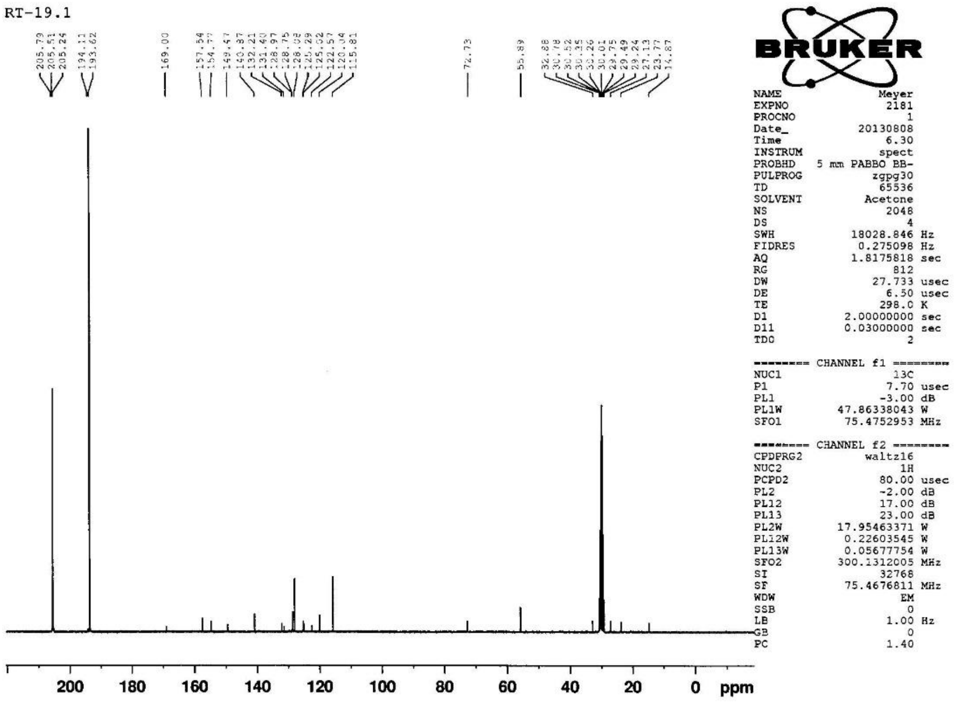

^{13}C NMR (75 MHz, acetone-d$_6$/CS$_2$ 4:1) of compound **10d**.

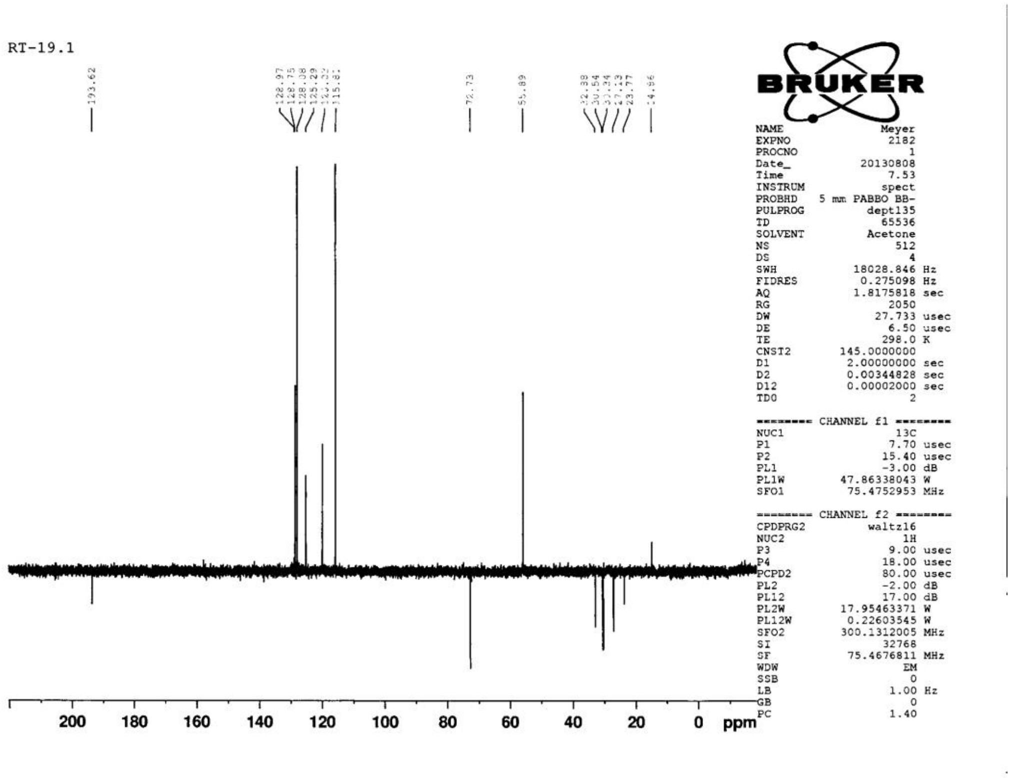

DEPT ^{13}C NMR (75 MHz, acetone-d_6/CS_2 4:1) of compound **10d**.

3.21. (*Z*)-4-((5-(4-(bis(4-methoxyphenyl)amino)phenyl)-4-(octyloxy)thiophen-2-yl)methylene)-5-methyl-2-phenyl-2,4-dihydro-3*H*-pyrazol-3-one (10e)

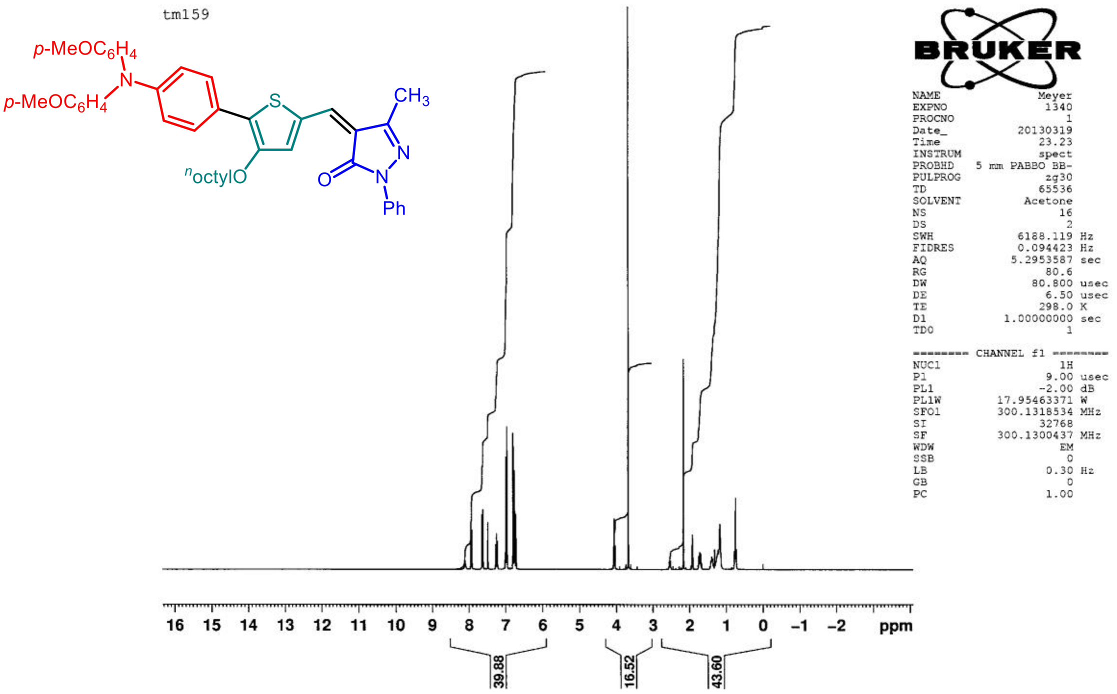

^{1}H NMR (300 MHz, acetone-d$_6$/CS$_2$ 4:1) of compound **10e**.

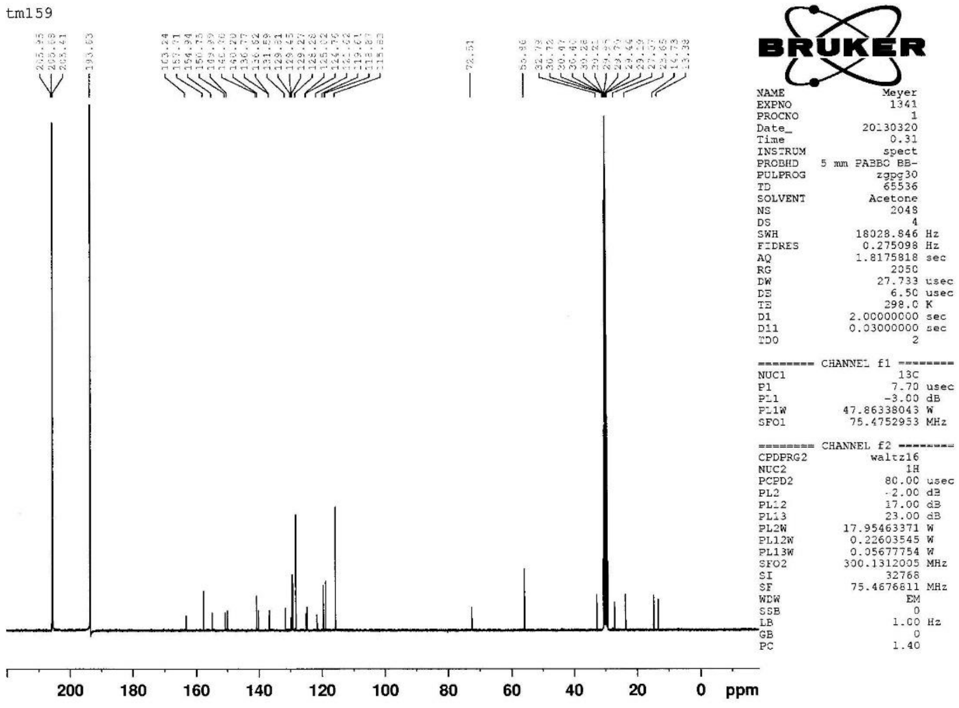

^{13}C NMR (75 MHz, acetone-d$_6$/CS$_2$ 4:1) of compound **10e**.

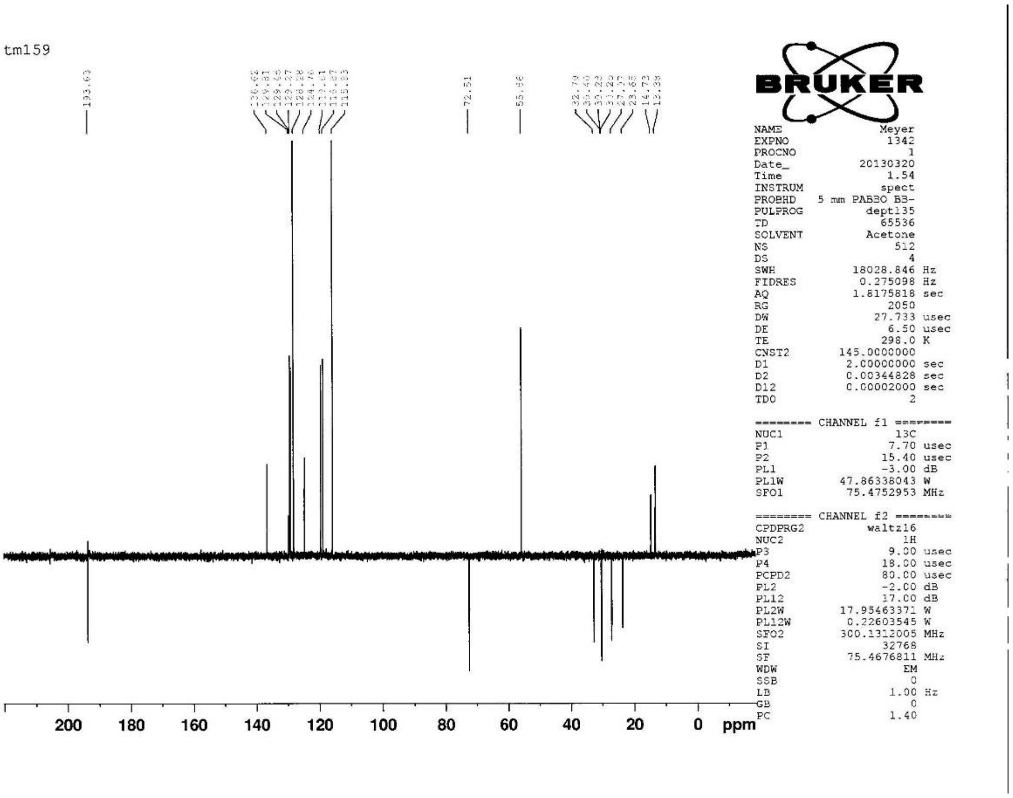

DEPT ^{13}C NMR (75 MHz, acetone-d_6/CS_2 4:1) of compound **10e**.

3.22. (*Z*)-5-((5-(10-(2-decyltetradecyl)-10*H*-phenothiazin-3-yl)-4-(octyloxy)thiophen-2-yl)methylene)-2-thioxothiazolidin-4-one (10f)

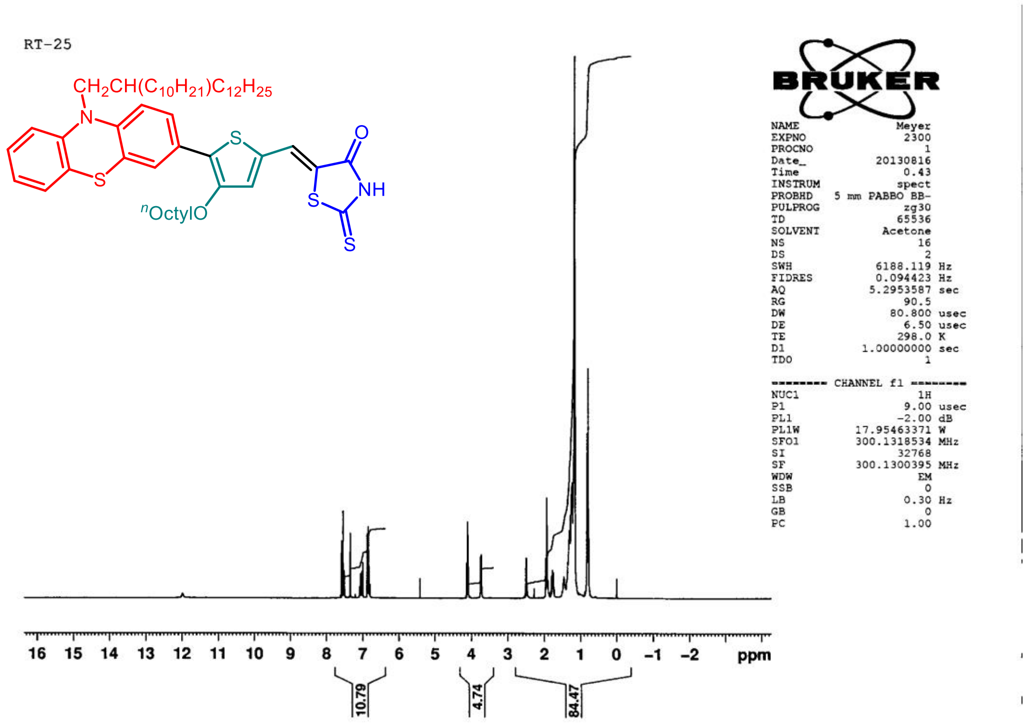

^{1}H NMR (300 MHz, acetone-d$_6$/CS$_2$ 4:1) of compound **10f**.

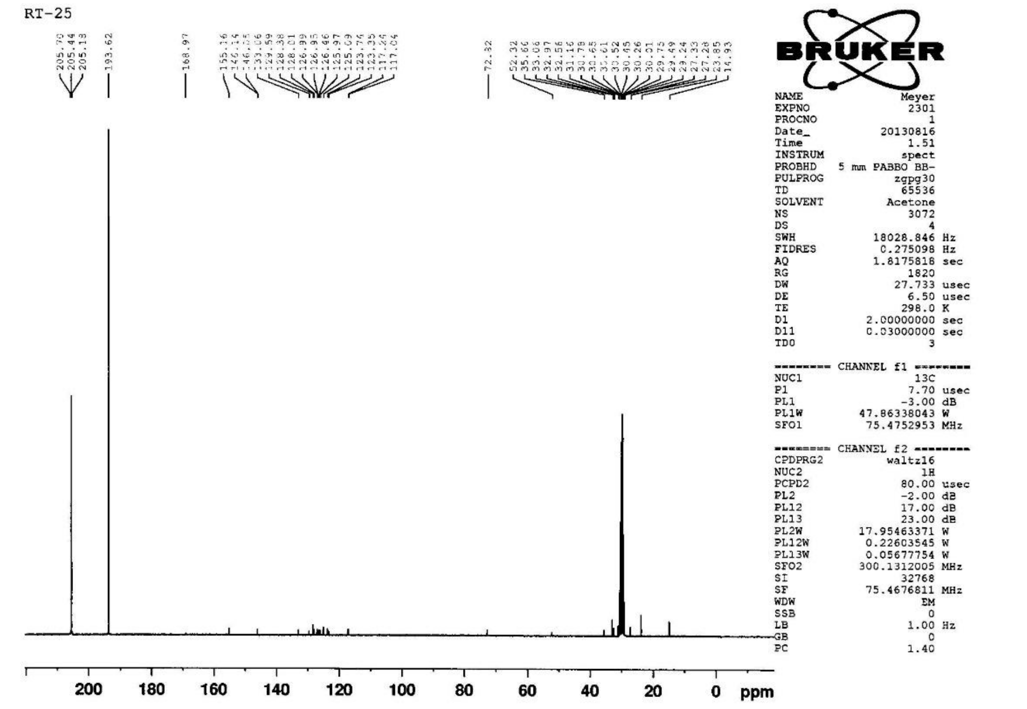

^{13}C NMR (75 MHz, acetone-d$_6$/CS$_2$ 4:1) of compound **10f**.

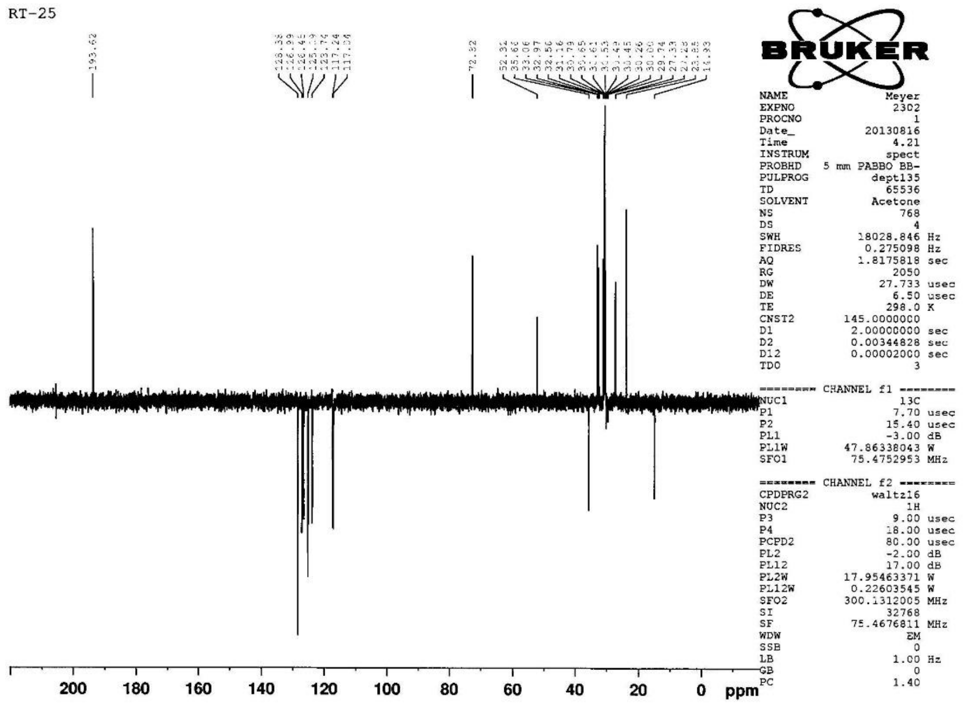

DEPT ^{13}C NMR (75 MHz, acetone-d_6/CS_2 4:1) of compound **10f**.

3.23. (*Z*)-5-((5-(10-hexyl-7-(*p*-tolyl)-10*H*-phenothiazin-3-yl)-4-(octyloxy)thiophen-2-yl)methylene)-2-thioxothiazolidin-4-one (10g)

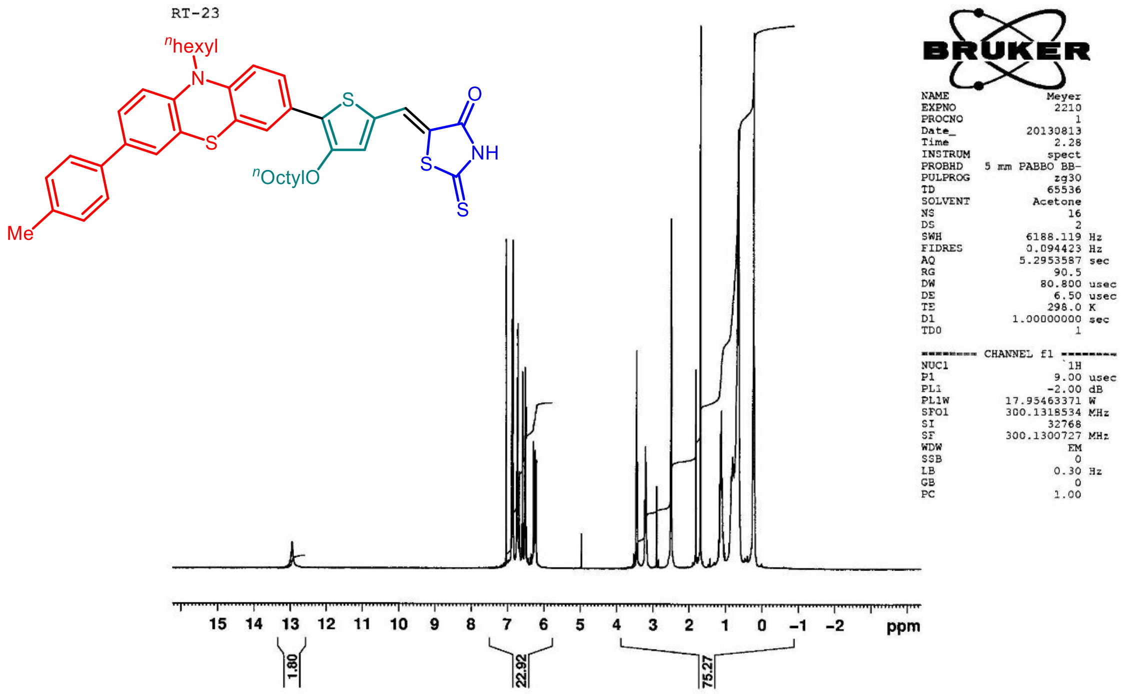

^{1}H NMR (300 MHz, acetone-d_6/CS_2 4:1) of compound **10g**.

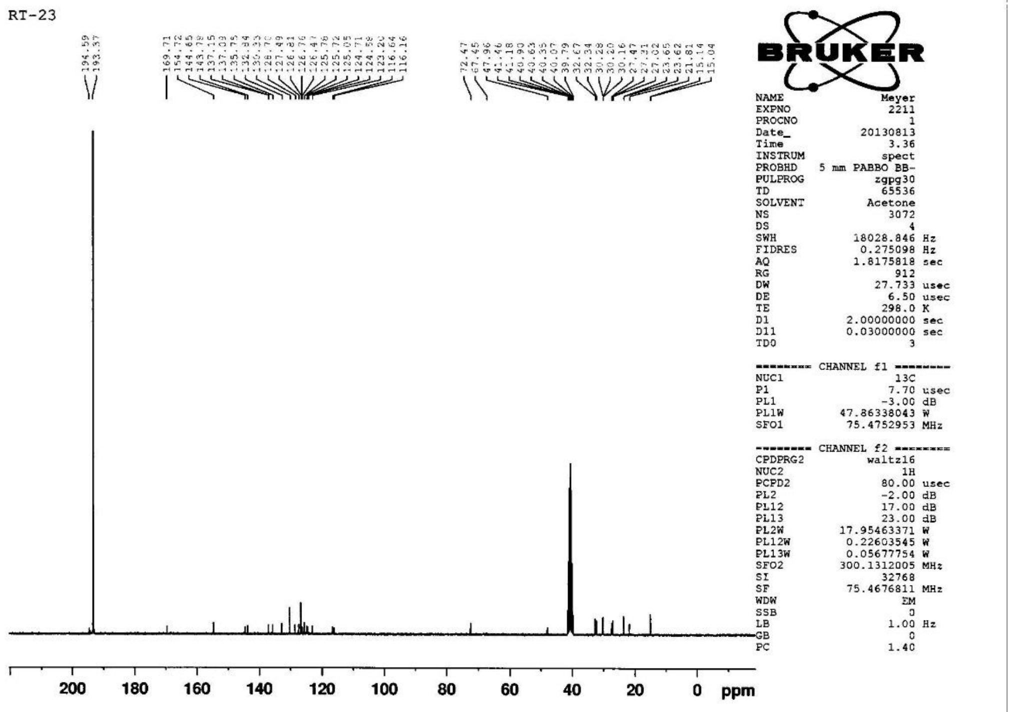

^{13}C NMR (75 MHz, acetone-d_6/CS_2 4:1) of compound **10g**.

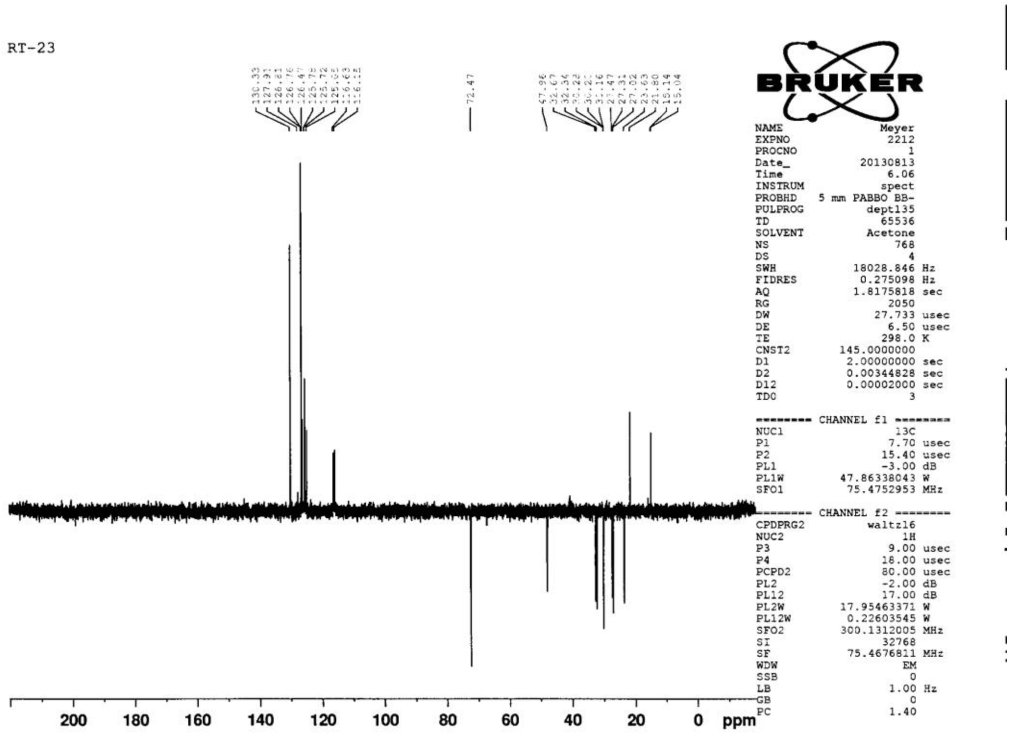

DEPT ^{13}C NMR (75 MHz, acetone-d_6/CS_2 4:1) of compound **10g**.

3.24. (*Z*)-4-((5-(10-hexyl-7-(*p*-tolyl)-10*H*-phenothiazin-3-yl)-4-(octyloxy)thiophen-2-yl)methylene)-5-methyl-2-phenyl-2,4-dihydro-3*H*-pyrazol-3-one (10h)

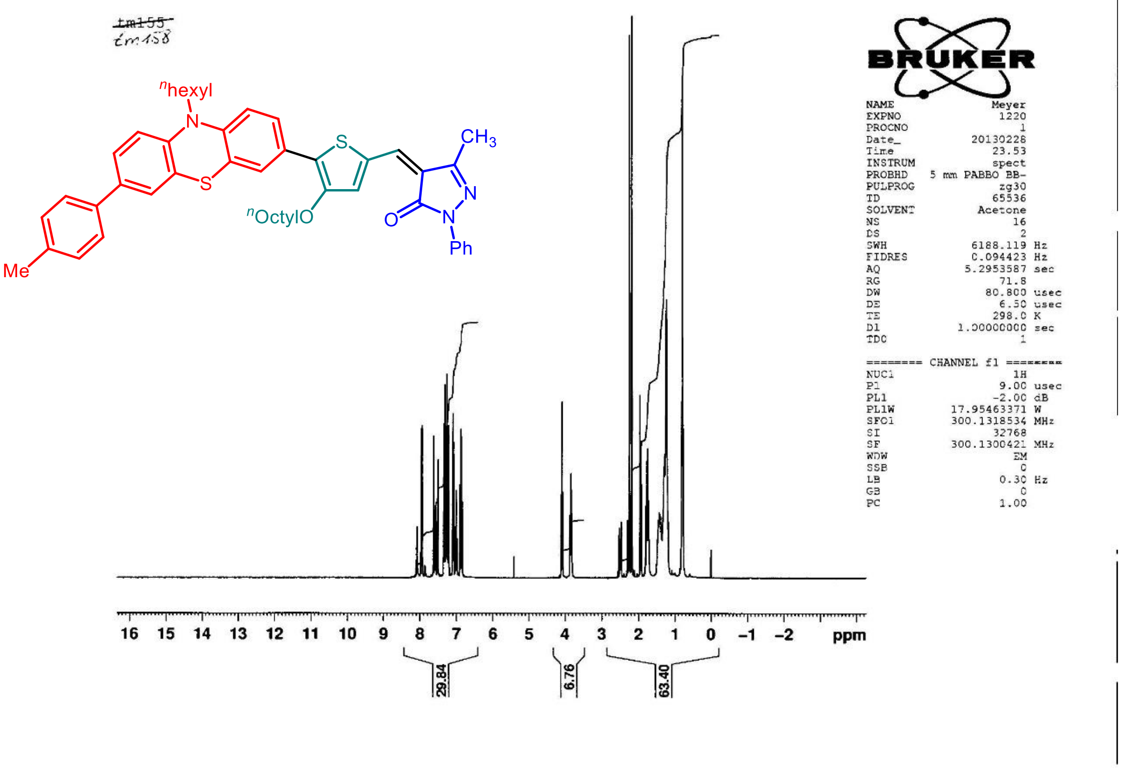

^{1}H NMR (300 MHz, acetone-d_6/CS_2 4:1) of compound **10h**.

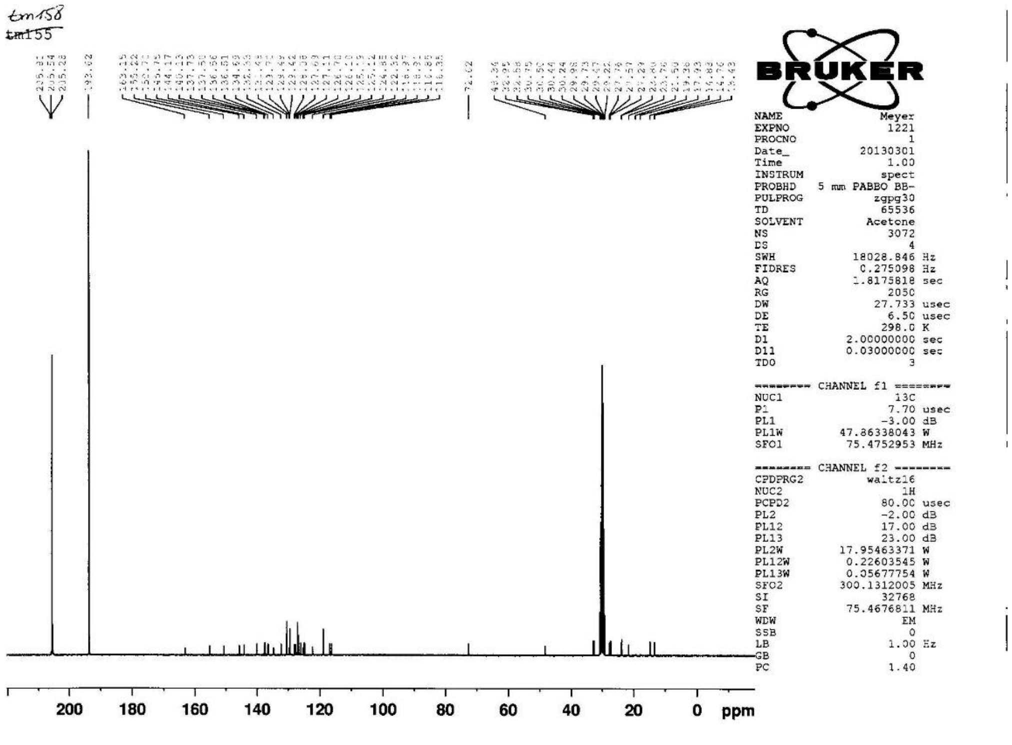

^{13}C NMR (75 MHz, acetone-d_6/CS_2 4:1) of compound **10h**.

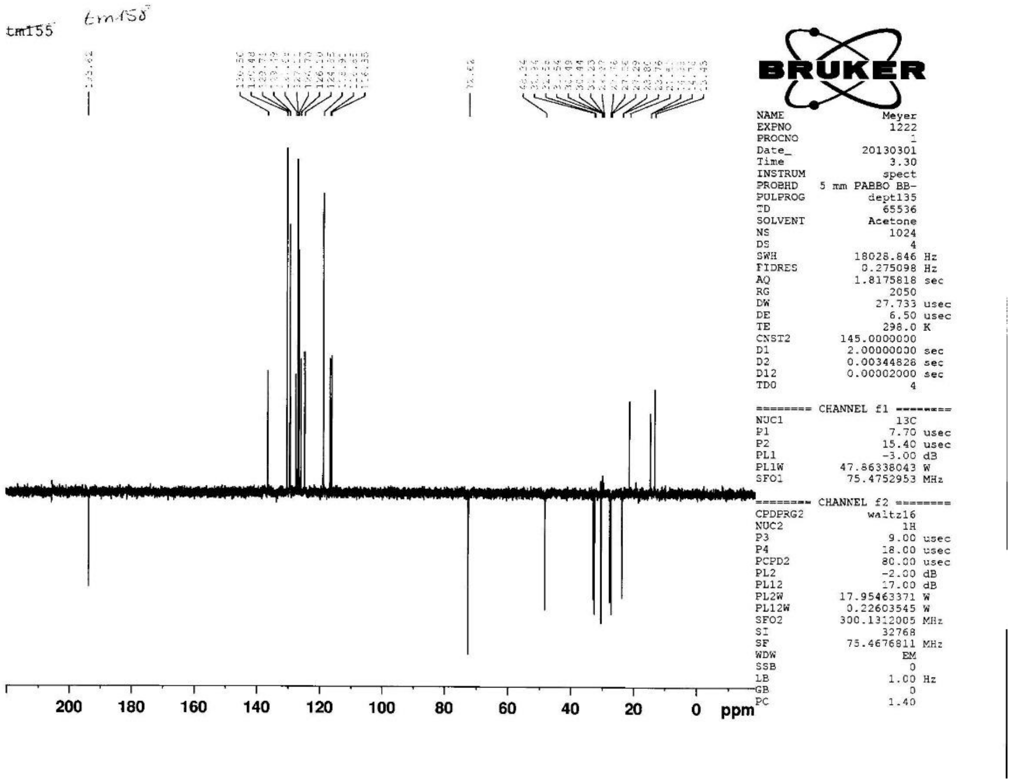

DEPT ^{13}C NMR (75 MHz, acetone-d_6/CS_2 4:1) of compound **10h**.

3.25. 5-{[9-(2-Decyltetradecyl)-6-(*p*-tolyl)-9*H*-carbazol-3-yl]methylene}-3-methyl-2-thioxothiazolidin-4-one (11a)

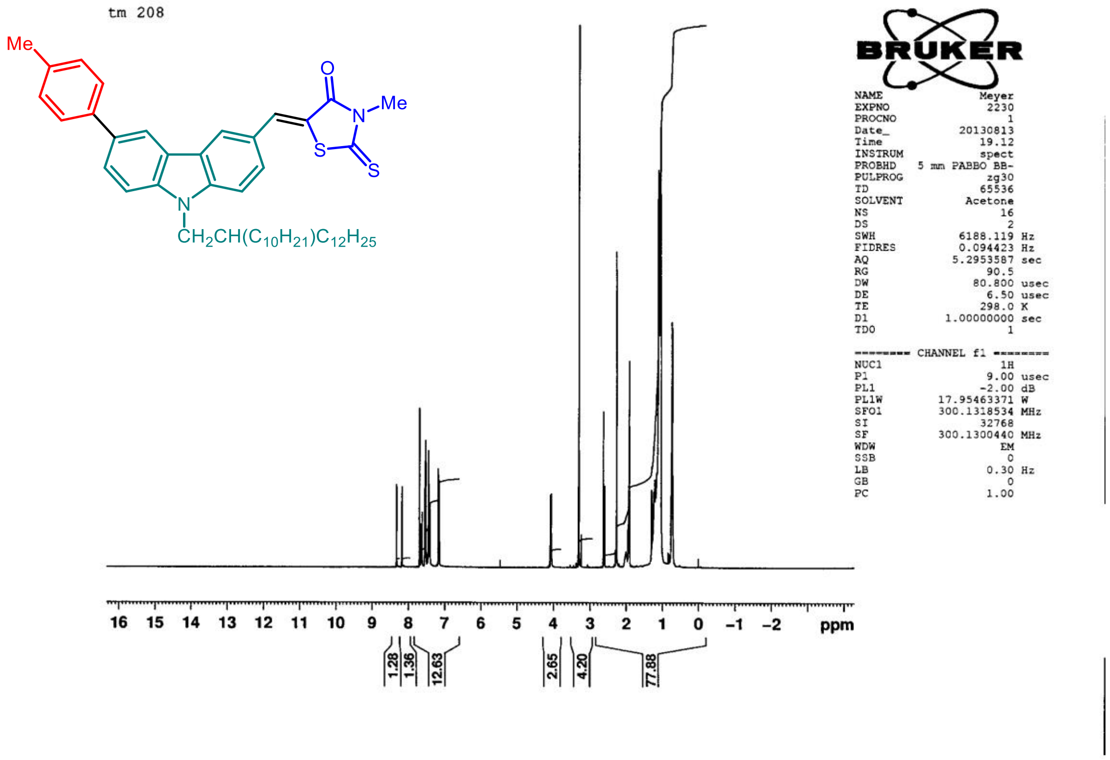

^{1}H NMR (300 MHz, acetone-d_6/CS_2 4:1) of compound **11a**.

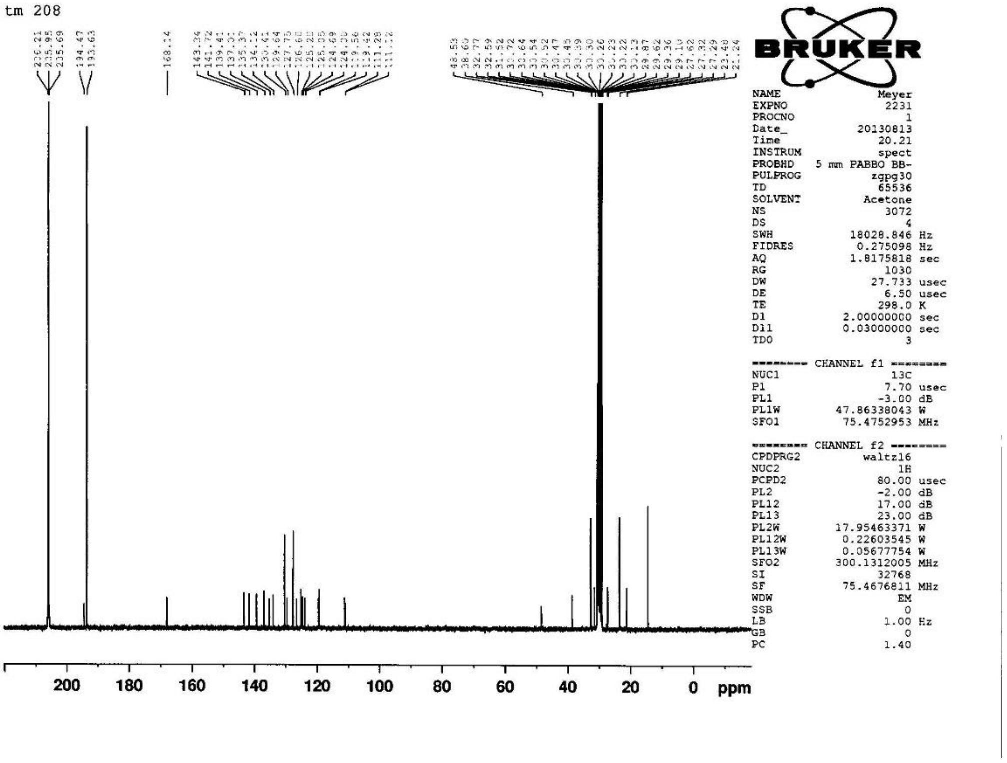

^{13}C NMR (75 MHz, acetone-d_6/CS_2 4:1) of compound **11a**.

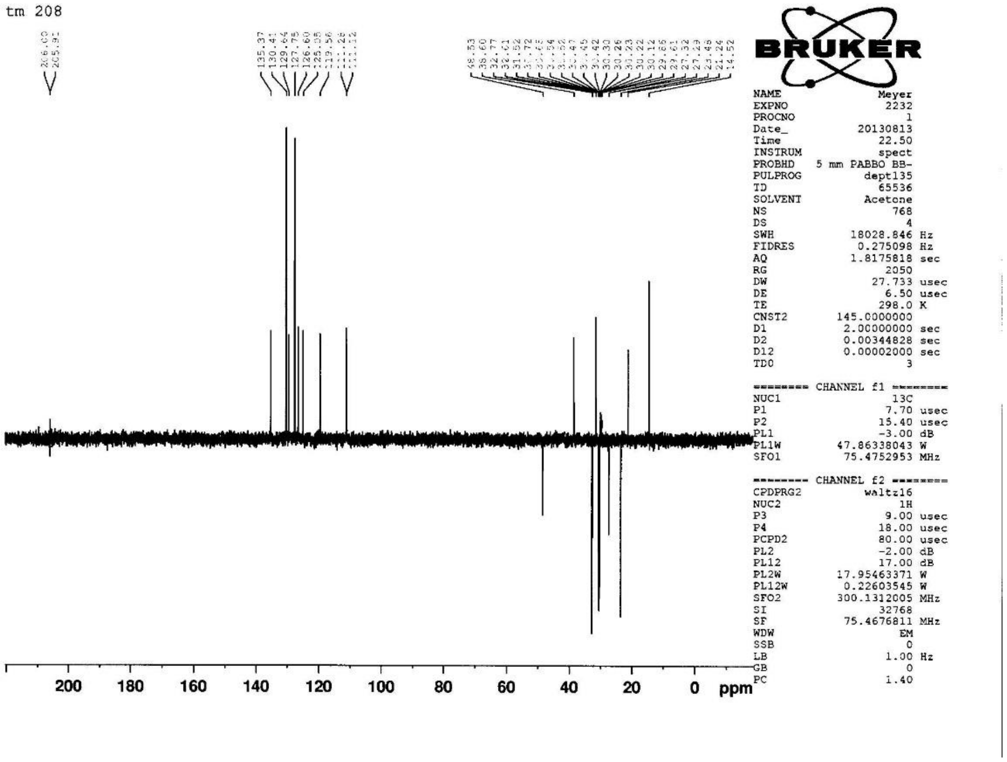

DEPT ^{13}C NMR (75 MHz, acetone-d_6/CS_2 4:1) of compound **11a**.

3.26. 5-{[9-(2-Decyltetradecyl)-6-(*p*-tolyl)-9H-carbazol-3-yl]methylene}-2-thioxothiazolidin-4-one (11b)

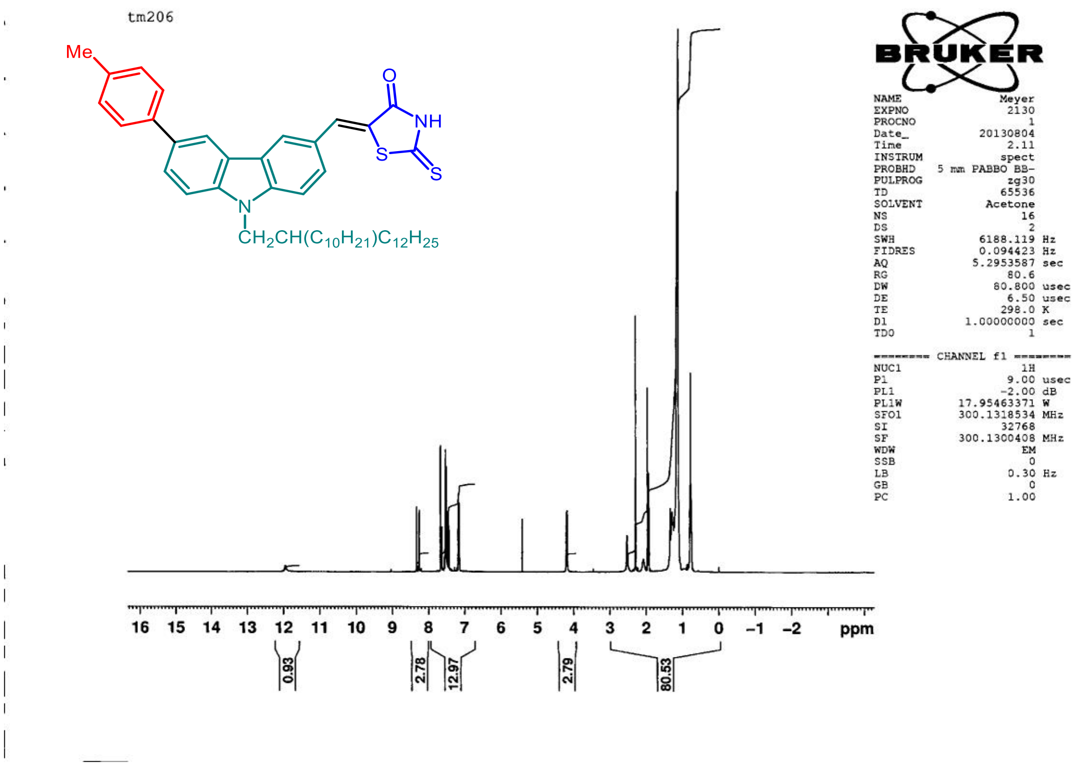

^{1}H NMR (300 MHz, acetone-d$_6$/CS$_2$ 4:1) of compound **11b**.

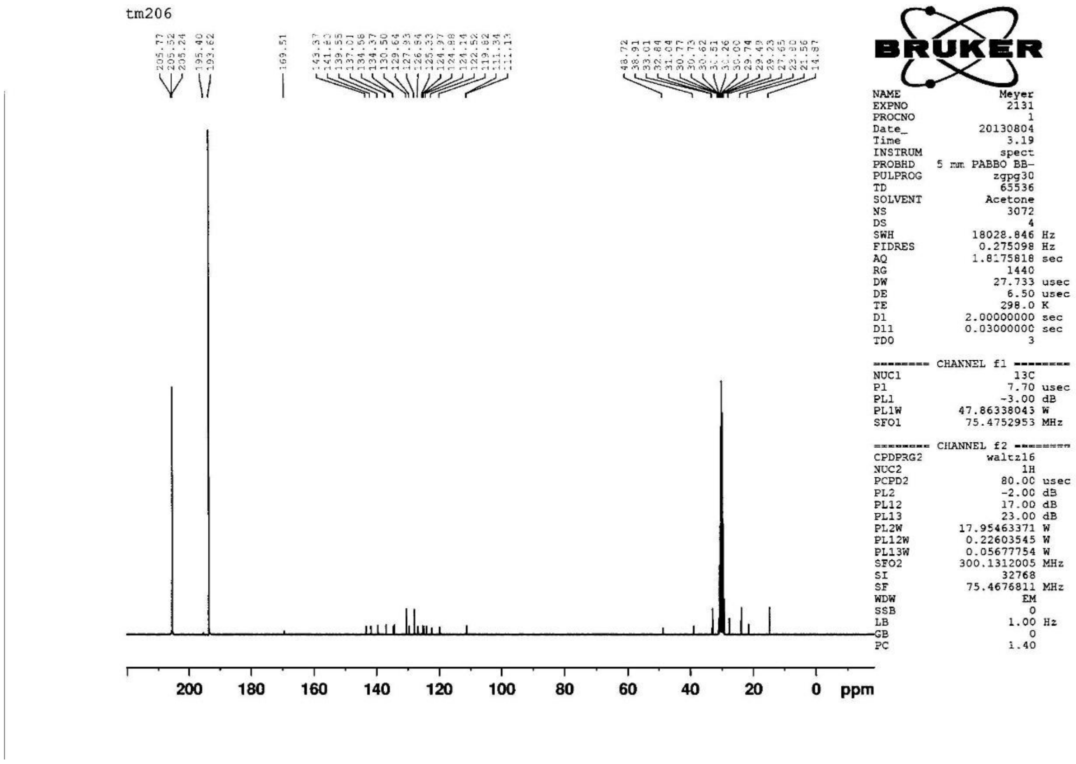

^{13}C NMR (75 MHz, acetone-d$_6$/CS$_2$ 4:1) of compound **11b**.

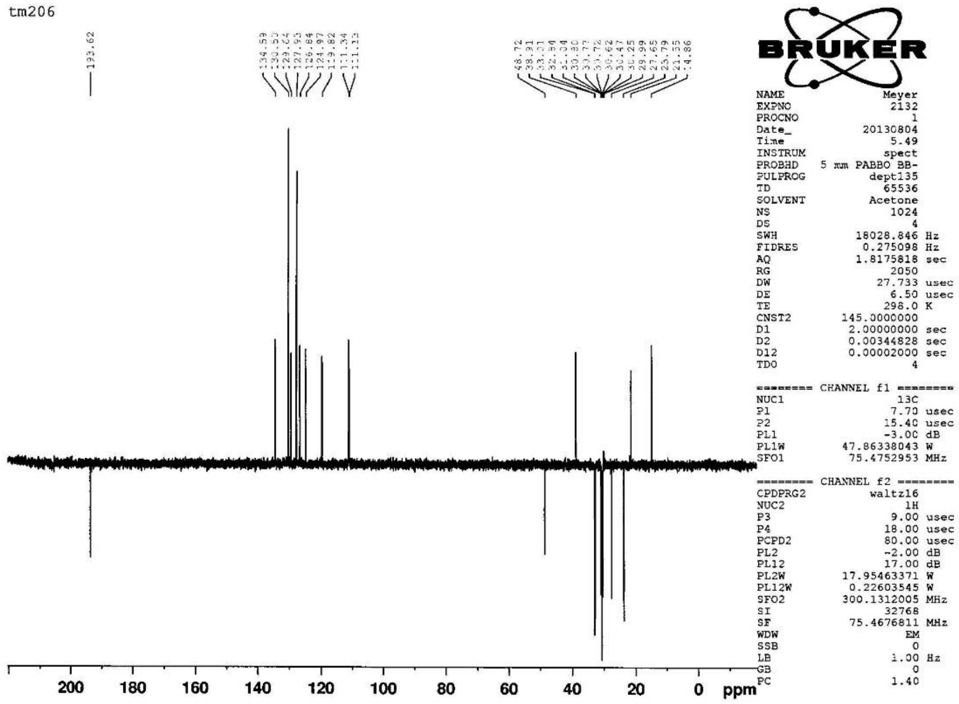

DEPT ^{13}C NMR (75 MHz, acetone-d_6/CS_2 4:1) of compound **11b**.

3.27. 2-{[9-(2-Decyltetradecyl)-6-(*p*-tolyl)-9*H*-carbazol-3-yl]methylene}-1*H*-inden-1,3[2*H*]-dione (11c)

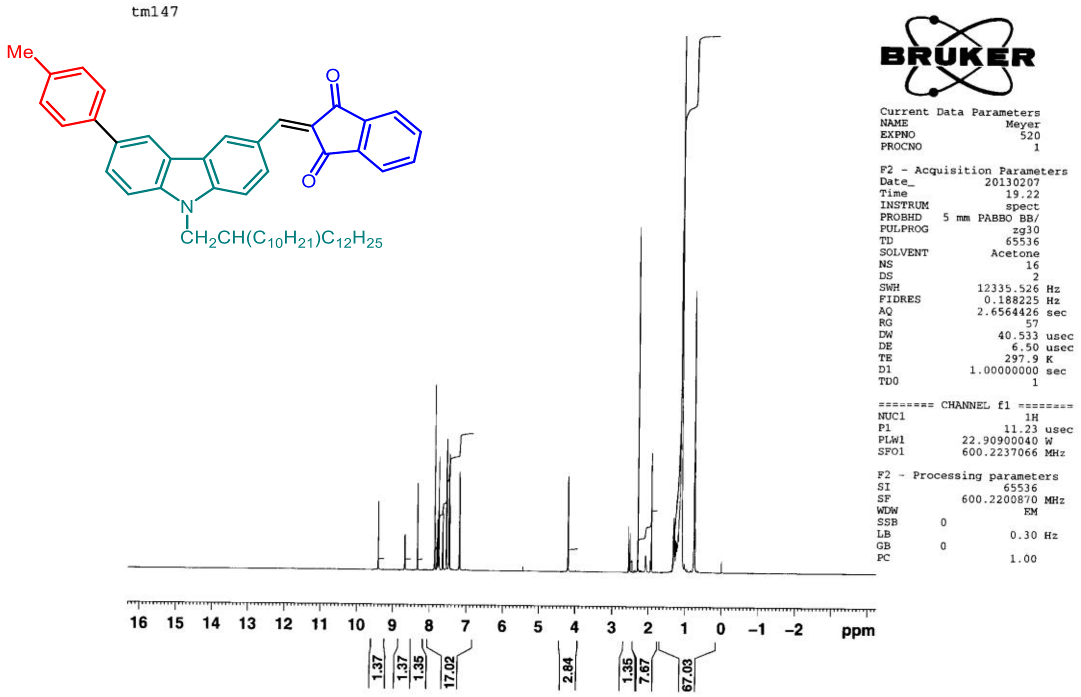

^{1}H NMR (600 MHz, acetone-d$_6$/CS$_2$ 4:1) of compound **11c**.

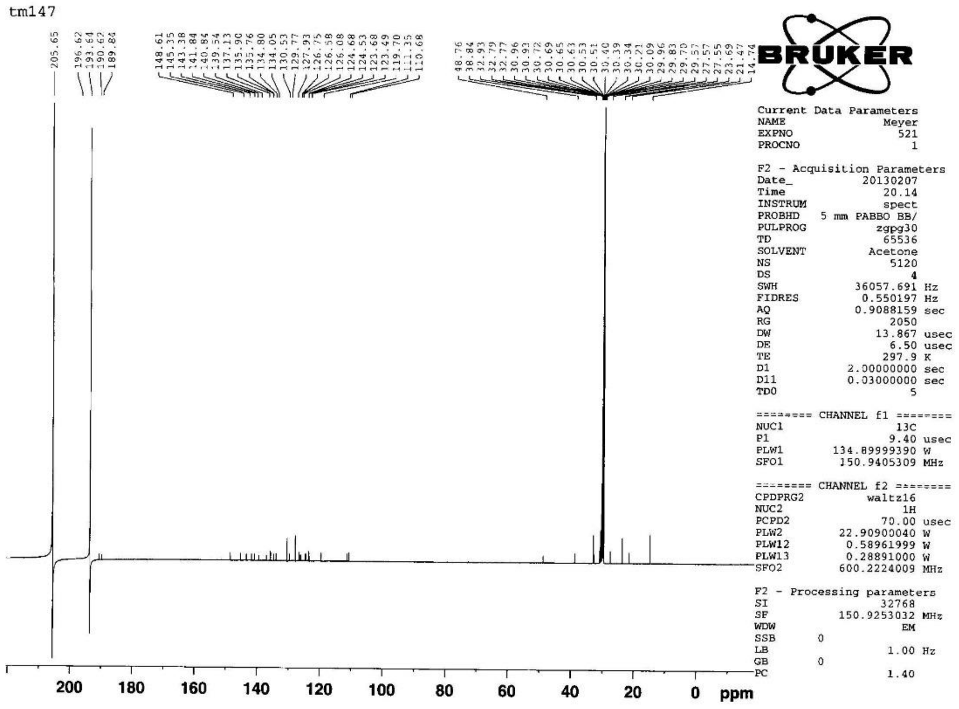

^{13}C NMR (150 MHz, acetone-d$_6$/CS$_2$ 4:1) of compound **11c**.

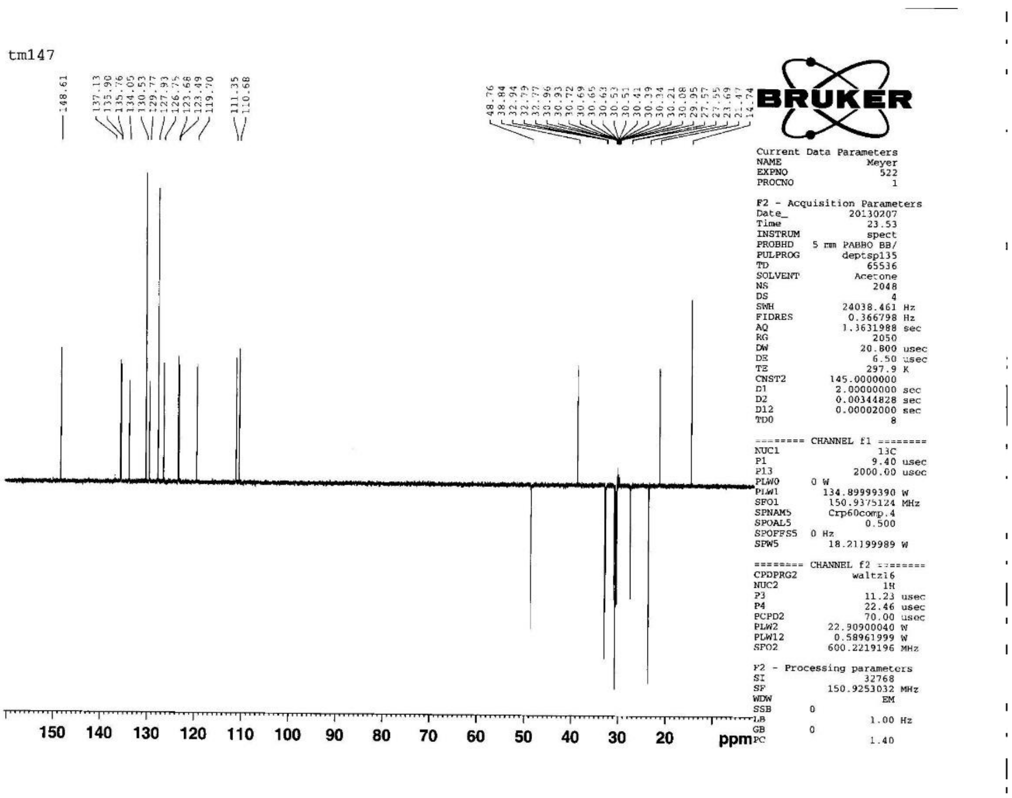

DEPT ^{13}C NMR (150 MHz, acetone-d_6/CS_2 4:1) of compound **11c**.

3.28. 4-{[9-(2-Decyltetradecyl)-6-(*p*-tolyl)-9*H*-carbazol-3-yl]methylene}-3-methyl-1-phenyl-1*H*-pyrazol-5[4*H*]-one (11d)

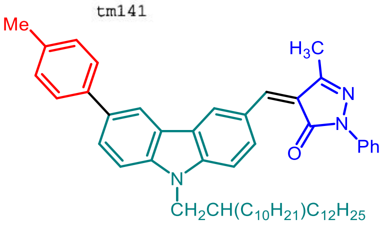

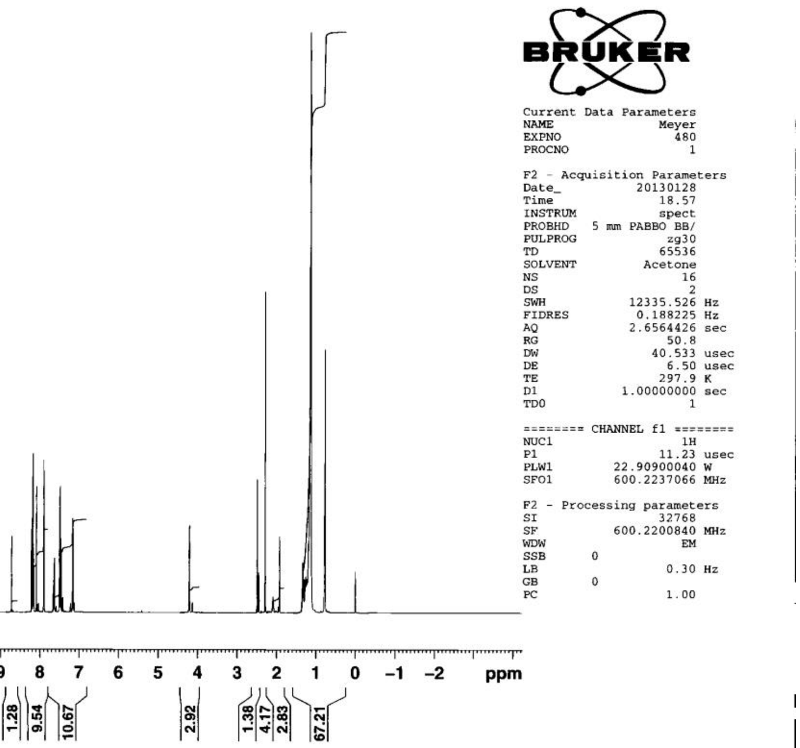

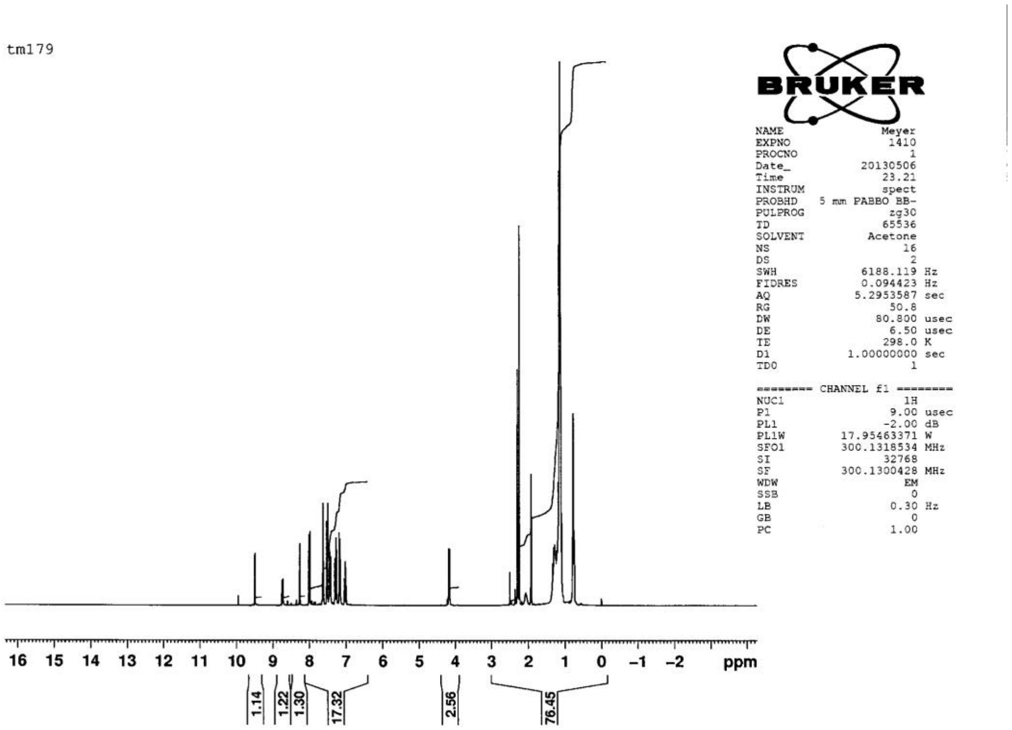

^1H NMR (300 MHz, acetone-d_6/CS_2 4:1) of compound **11d**.

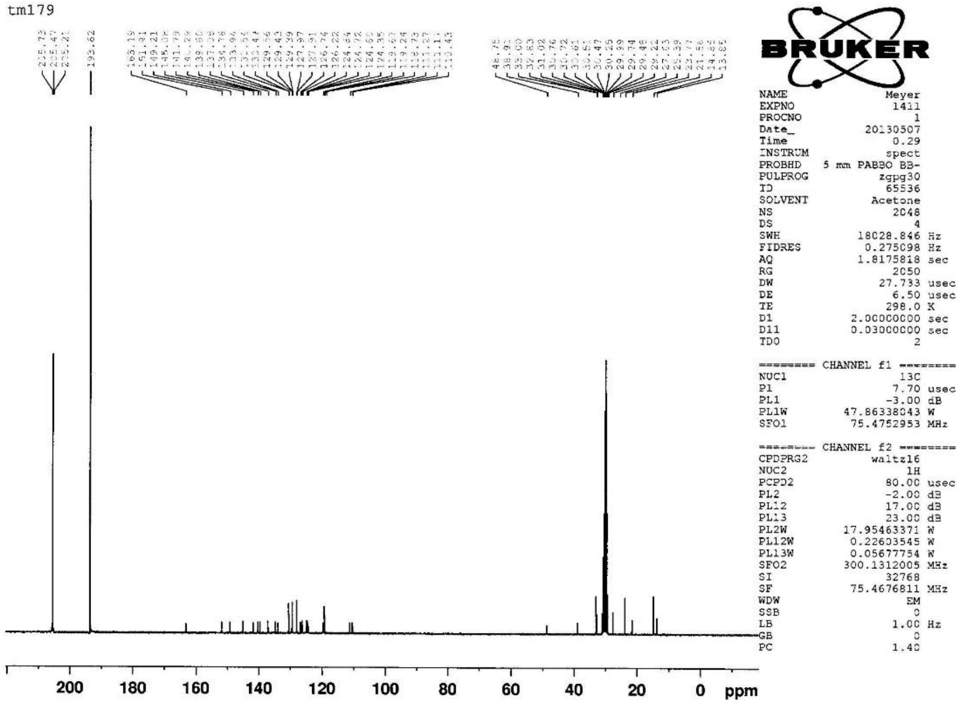

^{13}C NMR (75 MHz, acetone-d_6/CS_2 4:1) of compound **11d**.

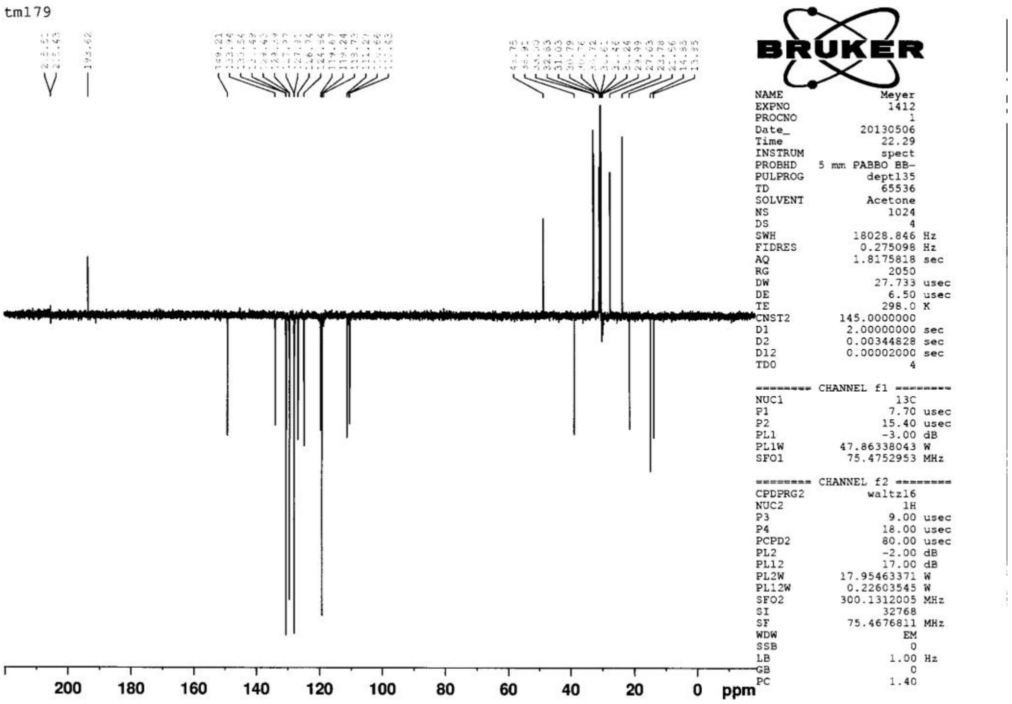

DEPT ^{13}C NMR (75 MHz, acetone-d_6/CS_2 4:1) of compound **11d**.

3.29. 3-[9-(2-Decyltetradecyl)-6-(*p*-tolyl)-9*H*-carbazol-3-yl]-2-(4-nitrophenyl)acrylonitrile (11e)

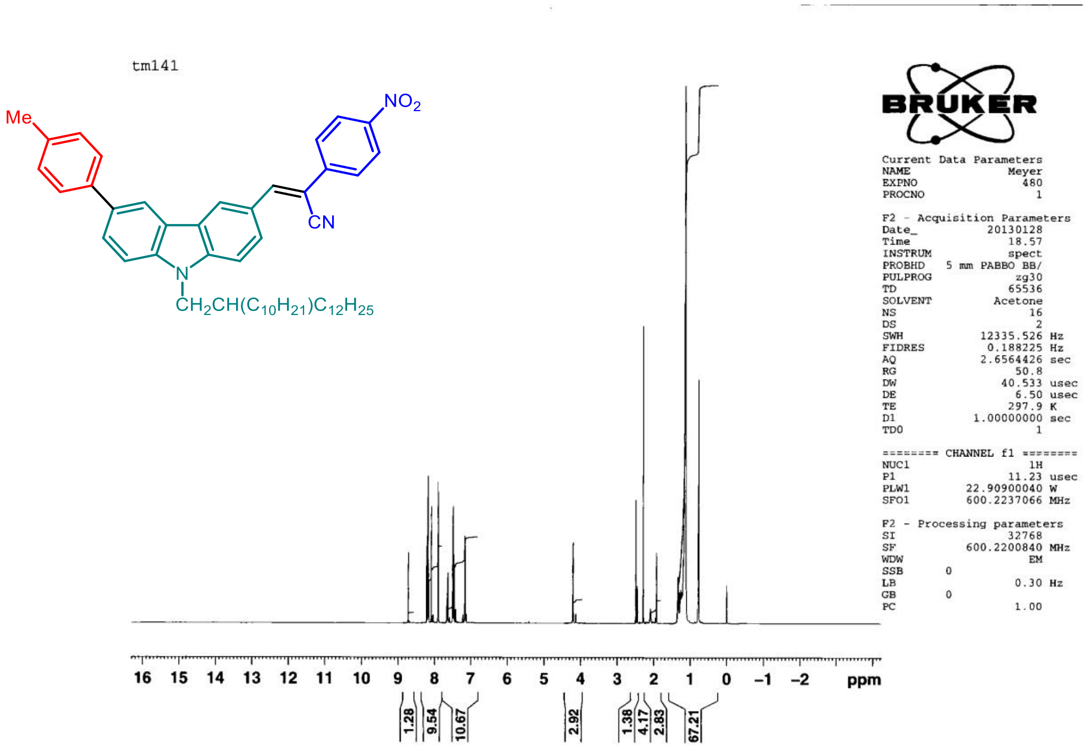

^{1}H NMR (600 MHz, acetone-d_6/CS_2 4:1) of compound **11e**.

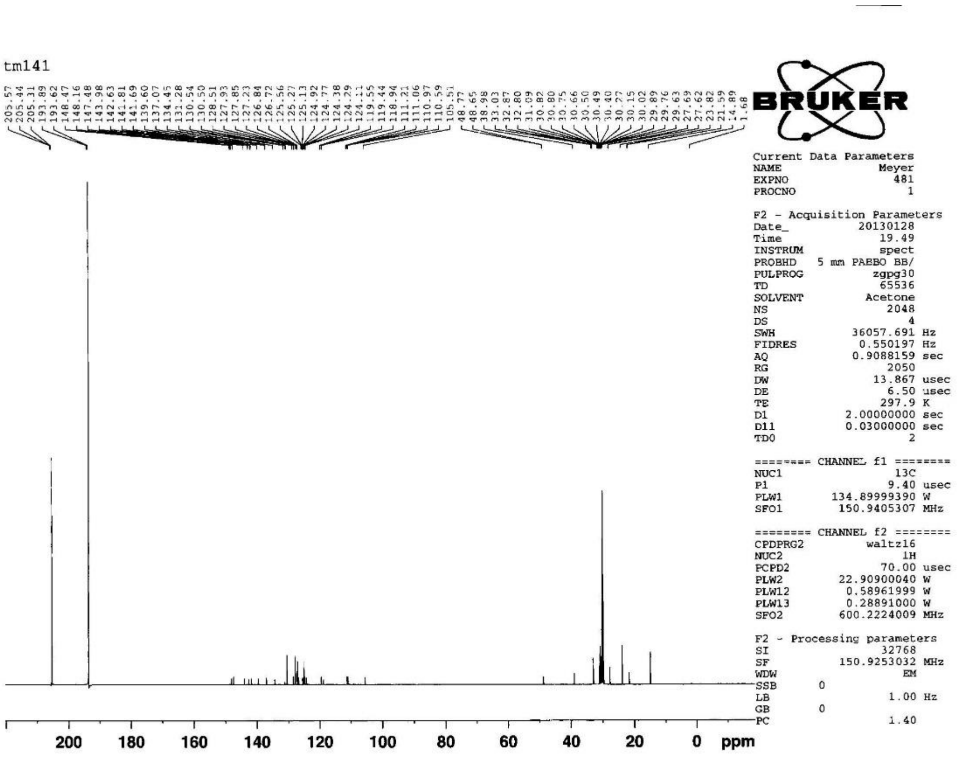

^{13}C NMR (150 MHz, acetone-d_6/CS_2 4:1) of compound **11e**.

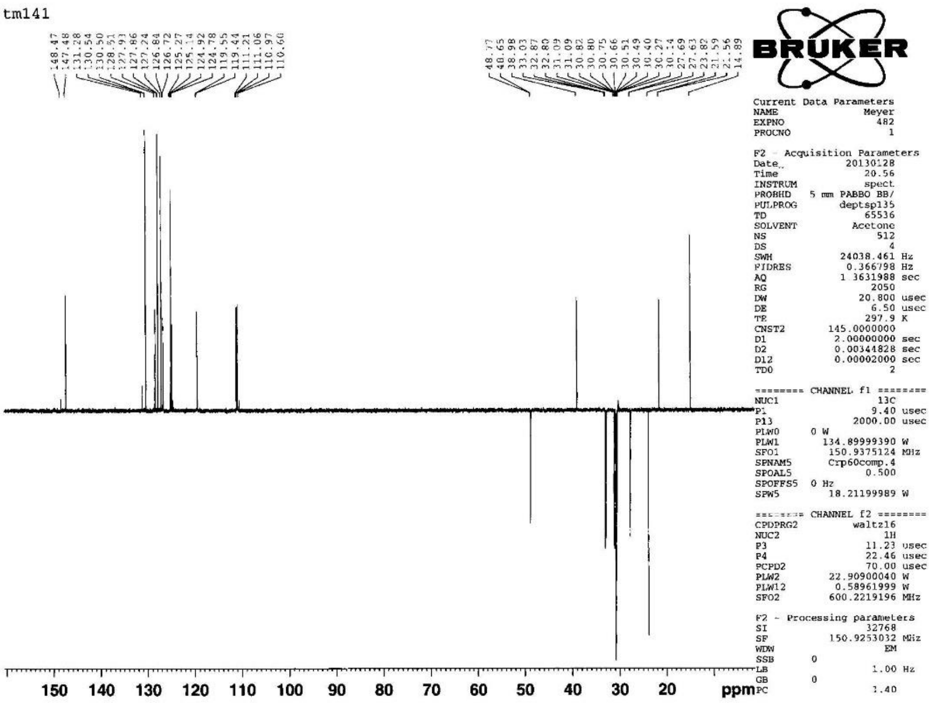

DEPT ^{13}C NMR (150 MHz, acetone-d_6/CS_2 4:1) of compound **11e**.

3.30. (*E*)-2-{3-Cyano-4-[2-(9-{2-decyltetradecyl}-6-{*p*-tolyl}-9*H*-carbazol-3-yl)vinyl]-5,5-dimethylfuran-2[5*H*]-yliden}malonitrile (11f)

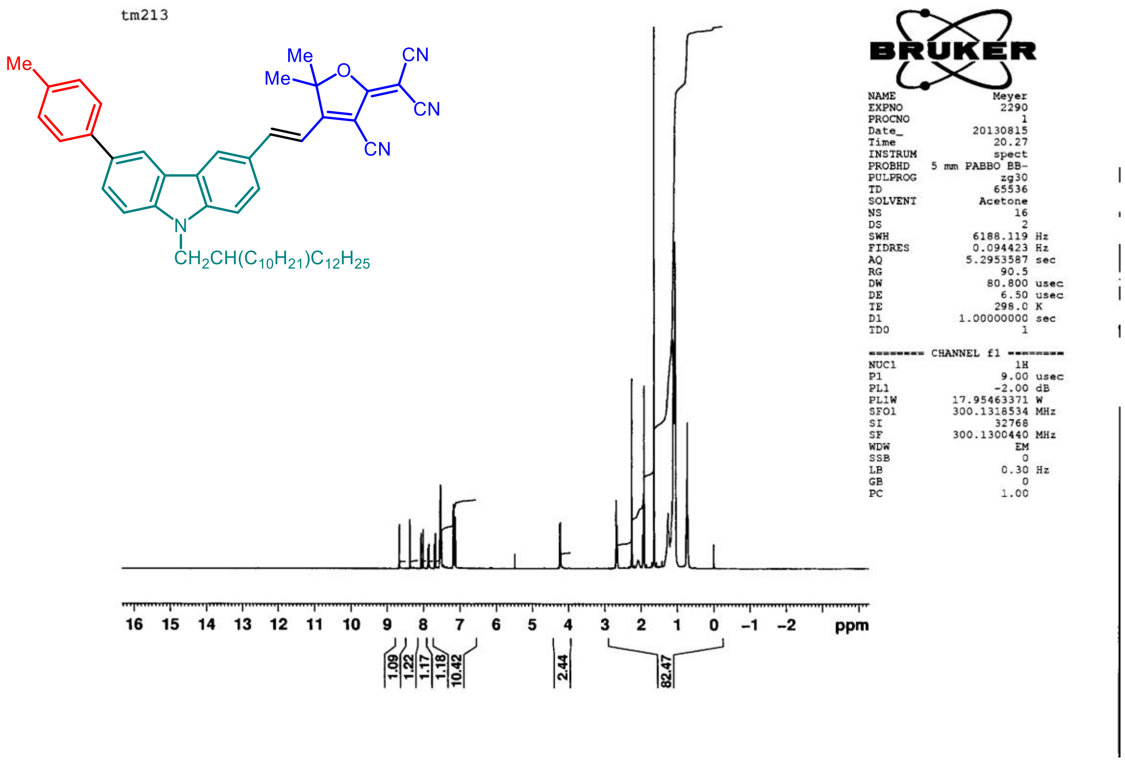

^{1}H NMR (300 MHz, acetone-d$_6$/CS$_2$ 4:1) of compound **11f**.

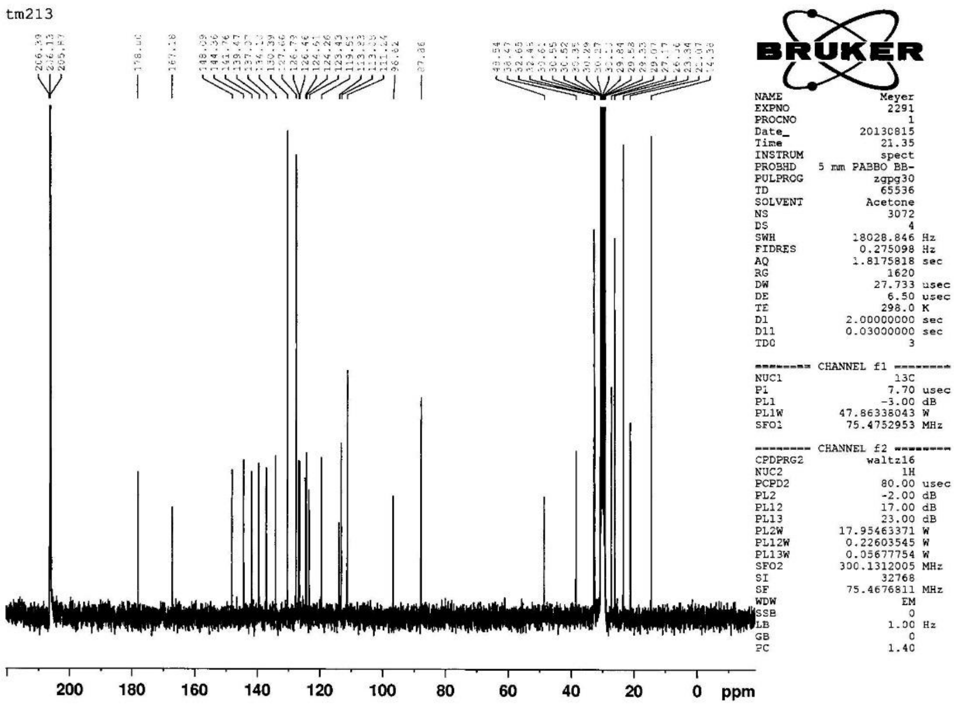

^{13}C NMR (75 MHz, acetone-d$_6$/CS$_2$ 4:1) of compound **11f**.

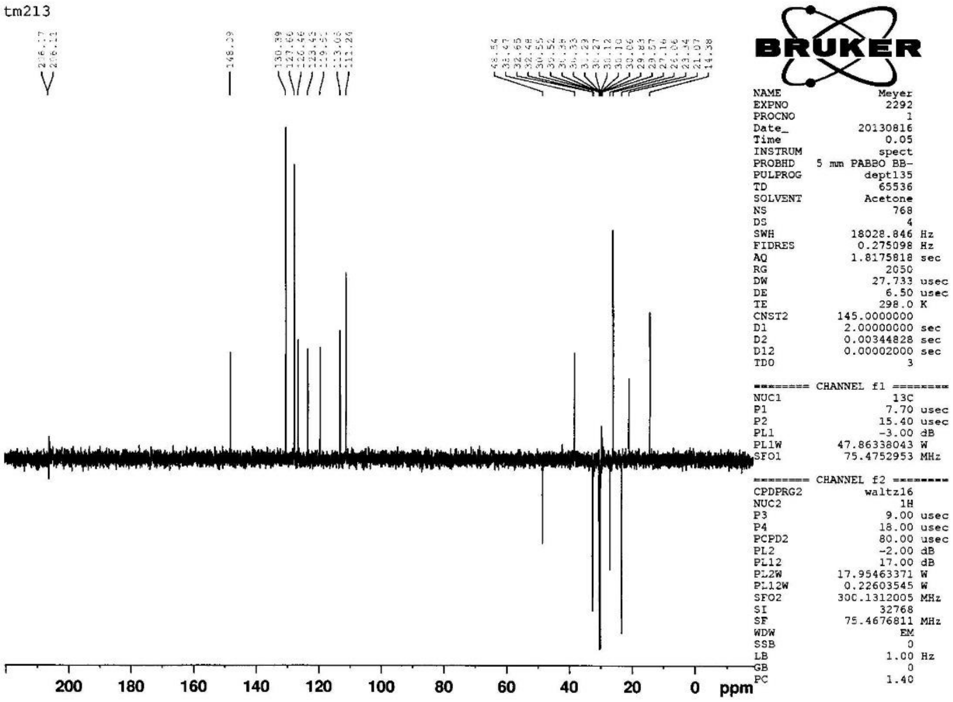

DEPT ^{13}C NMR (75 MHz, acetone-d_6/CS_2 4:1) of compound **11f**.

3.31. 2-{[9-(2-Decyltetradecyl)-6-(1-methyl-1H-pyrazol-4-yl)-9*H*-carbazol-3-yl]methylene}-1*H*-inden-1,3[2*H*]-dione (11g)

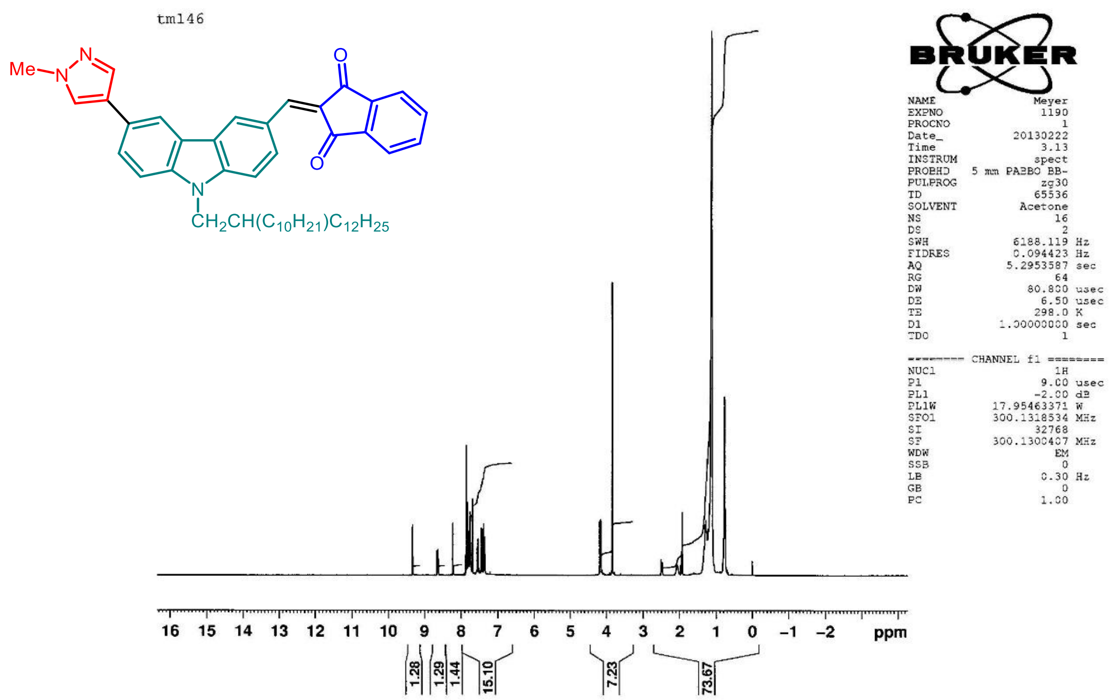

^{1}H NMR (600 MHz, acetone-d$_6$/CS$_2$ 4:1) of compound **11g**.

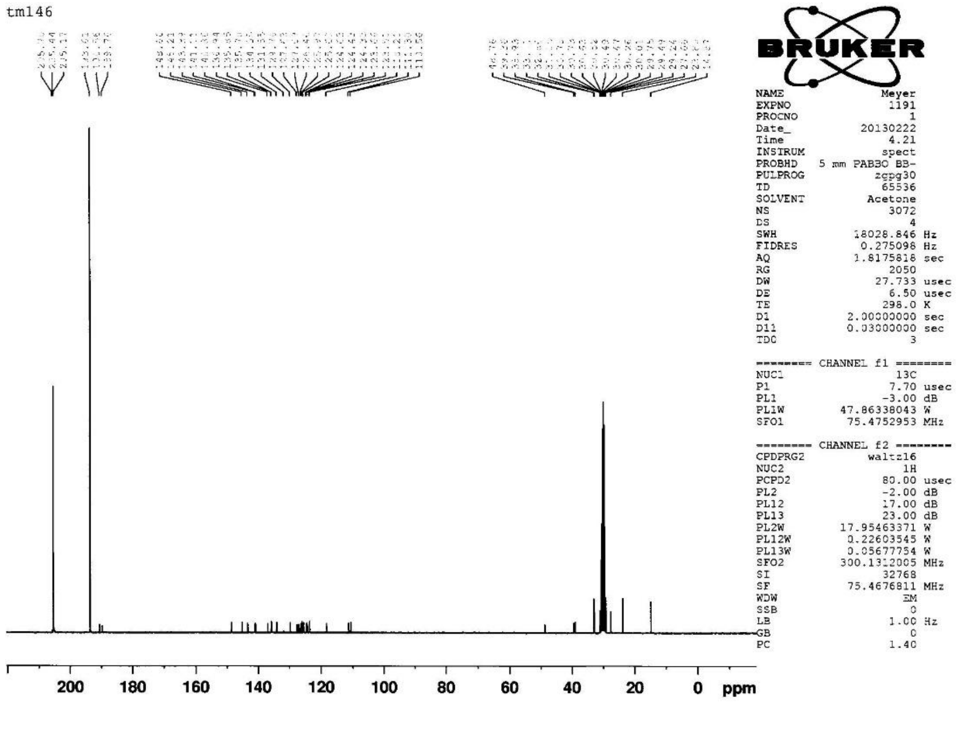

^{13}C NMR (150 MHz, acetone-d$_6$/CS$_2$ 4:1) of compound **11g**.

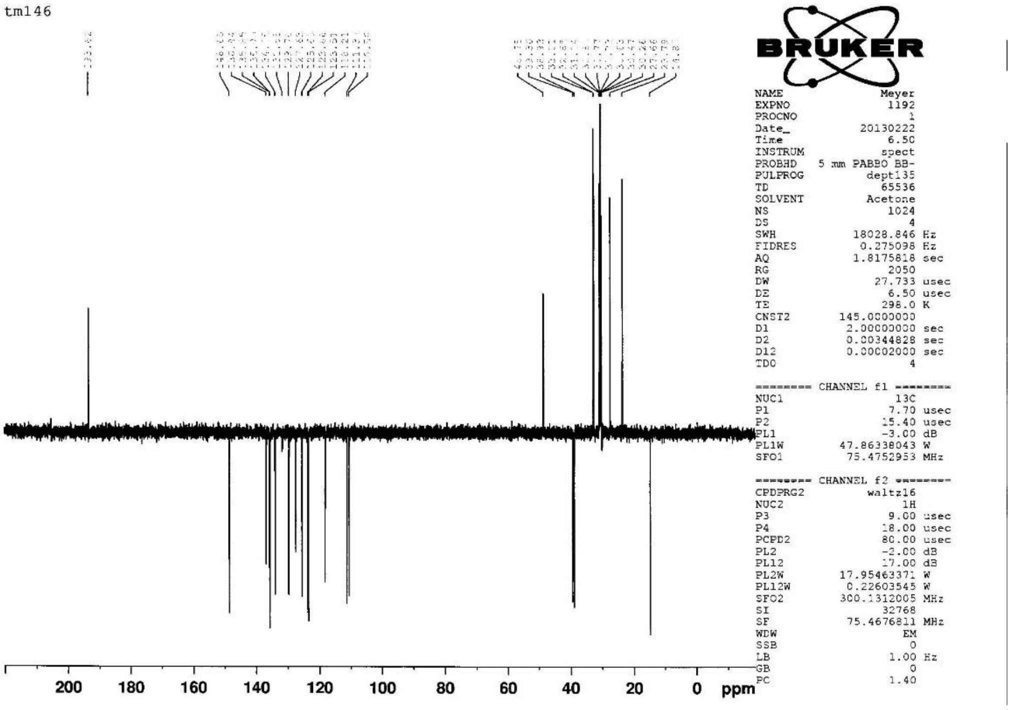

DEPT ^{13}C NMR (150 MHz, acetone-d_6/CS_2 4:1) of compound **11g**.

3.32. 4-{[6-(4-{Bis[4-methoxyphenyl]amino}phenyl)-9-(2-decyltetradecyl)-9*H*-carbazol-3-yl]methylene}-3-methyl-1-phenyl-1*H*-pyrazol-5[4*H*]-one (11h)

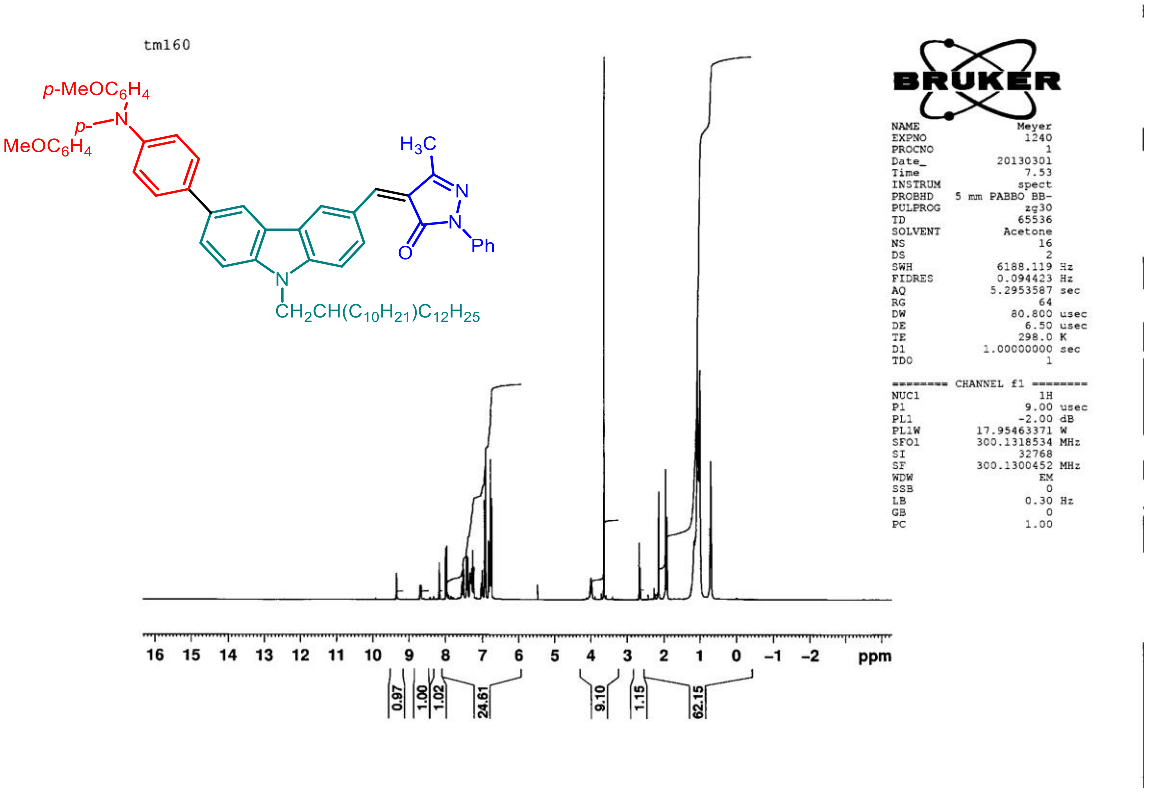

^{1}H NMR (300 MHz, acetone-d$_6$/CS$_2$ 4:1) of compound **11h**.

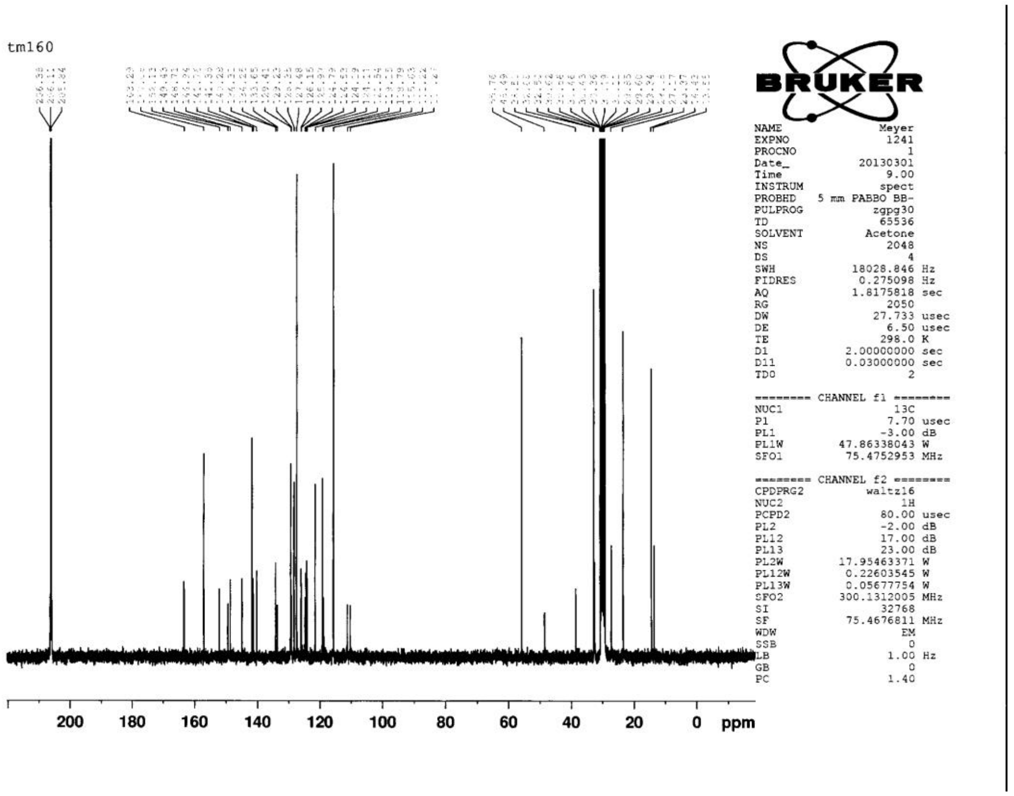

^{13}C NMR (75 MHz, acetone-d$_6$/CS$_2$ 4:1) of compound **11h**.

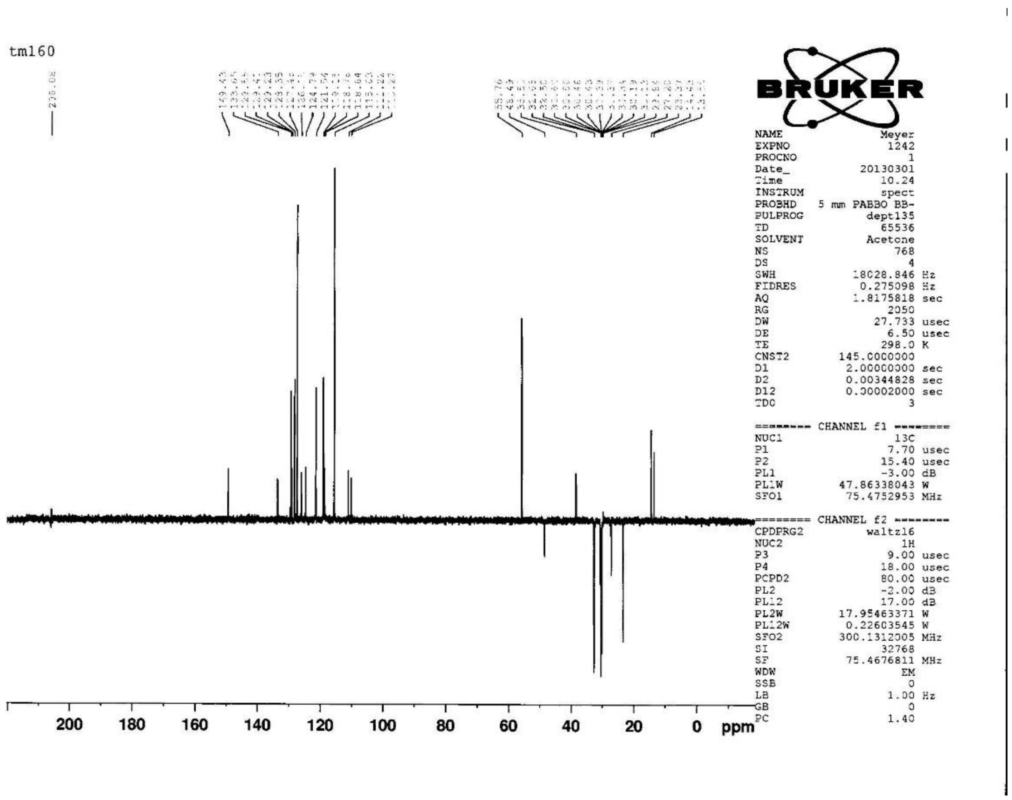

DEPT ^{13}C NMR (75 MHz, acetone-d_6/CS_2 4:1) of compound **11h**.

3.33. 4-{[9-(2-Decyltetradecyl)-6-(10-hexyl-7-{*p*-tolyl}-10*H*-phenothiazin-3-yl)-9*H*-carbazol-3-yl]methylene}-3-methyl-1-phenyl-1H-pyrazol-5[4*H*]-one (11i)

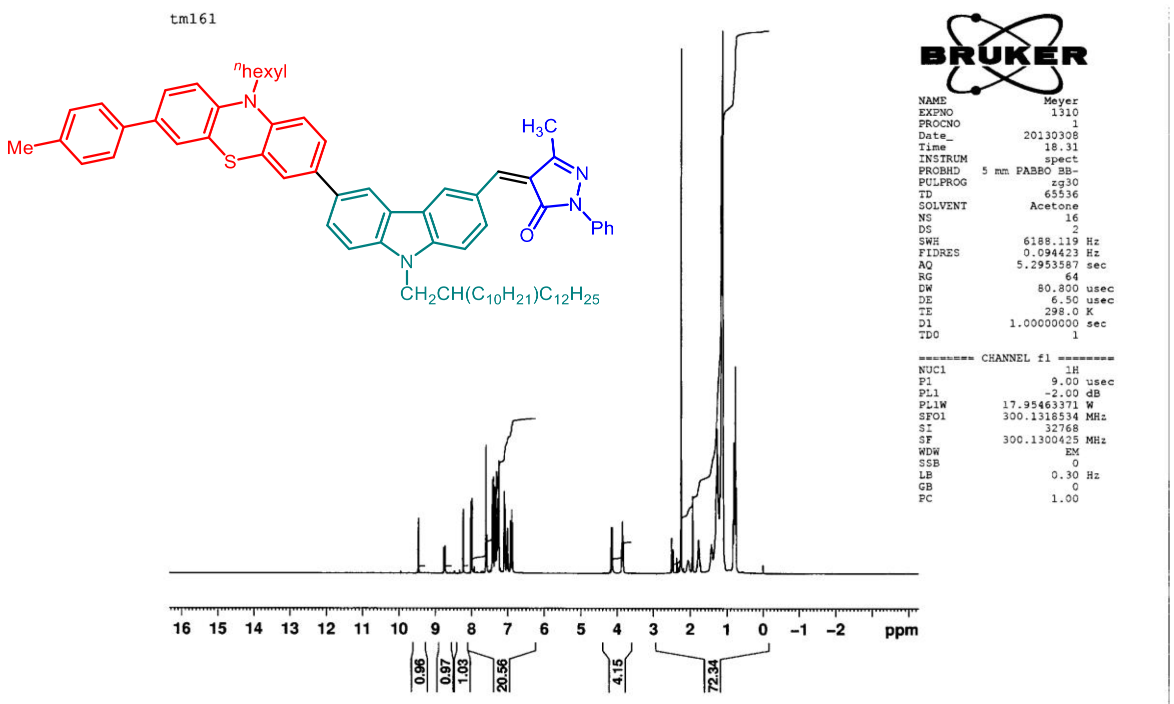

^1H NMR (300 MHz, acetone-d$_6$/CS_2 4:1) of compound **11i**.

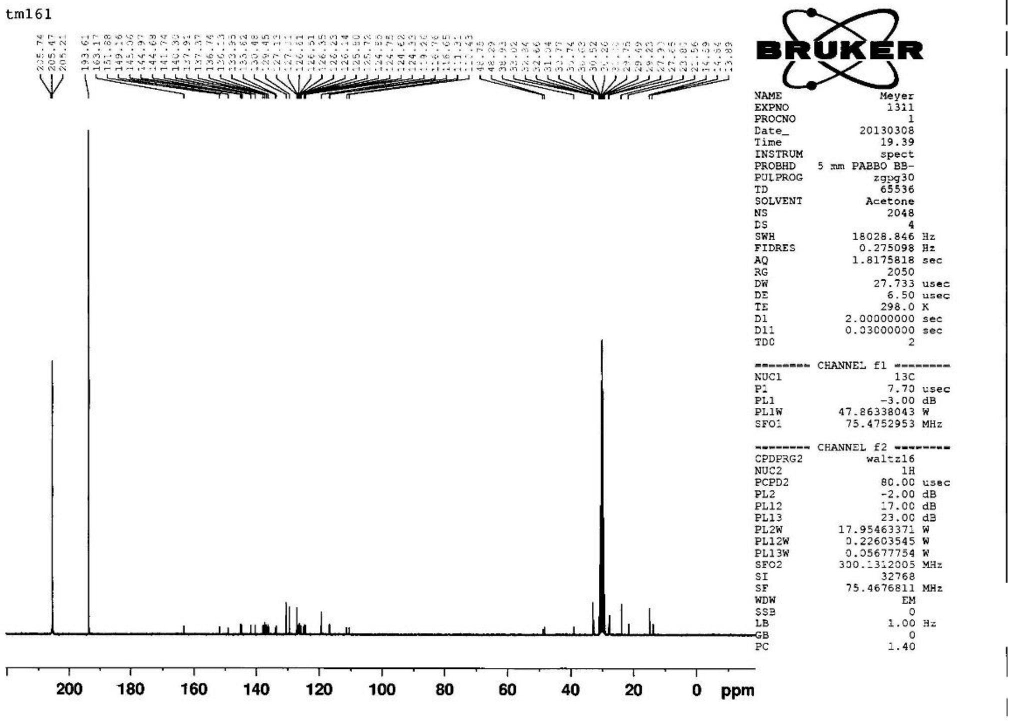

^{13}C NMR (75 MHz, acetone-d$_6$/CS_2 4:1) of compound **11i**.

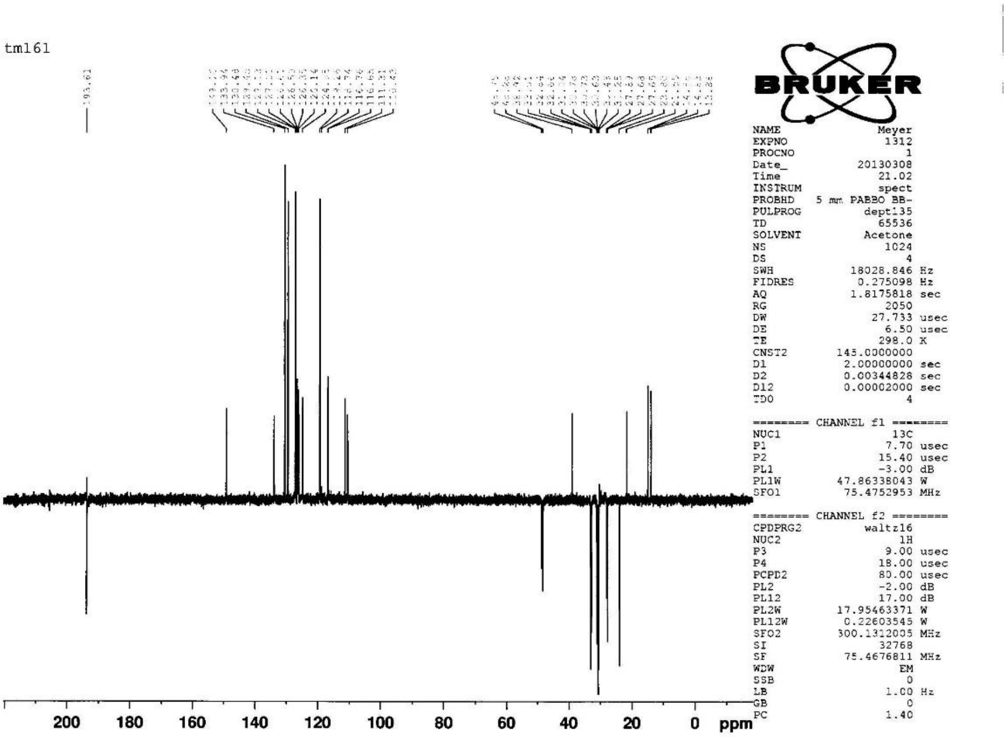

DEPT ^{13}C NMR (75 MHz, acetone-d_6/CS_2 4:1) of compound **11i**.

3.34. (*Z*)-5-{[10-(2-Decyltetradecyl)-7-(*p*-tolyl)-10*H*-phenothiazin-3-yl]methylene}-3-methyl-2-thioxothiazolidin-4-one (12a)

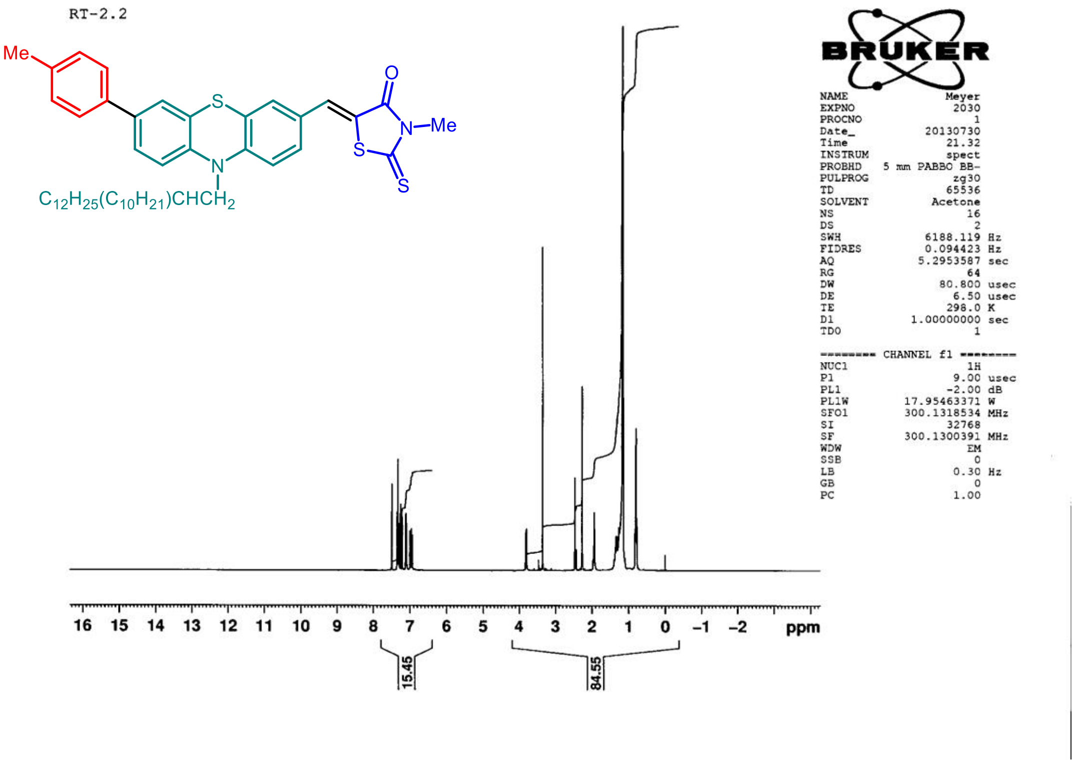

^{1}H NMR (300 MHz, acetone-d$_6$/CS$_2$ 4:1) of compound **12a**.

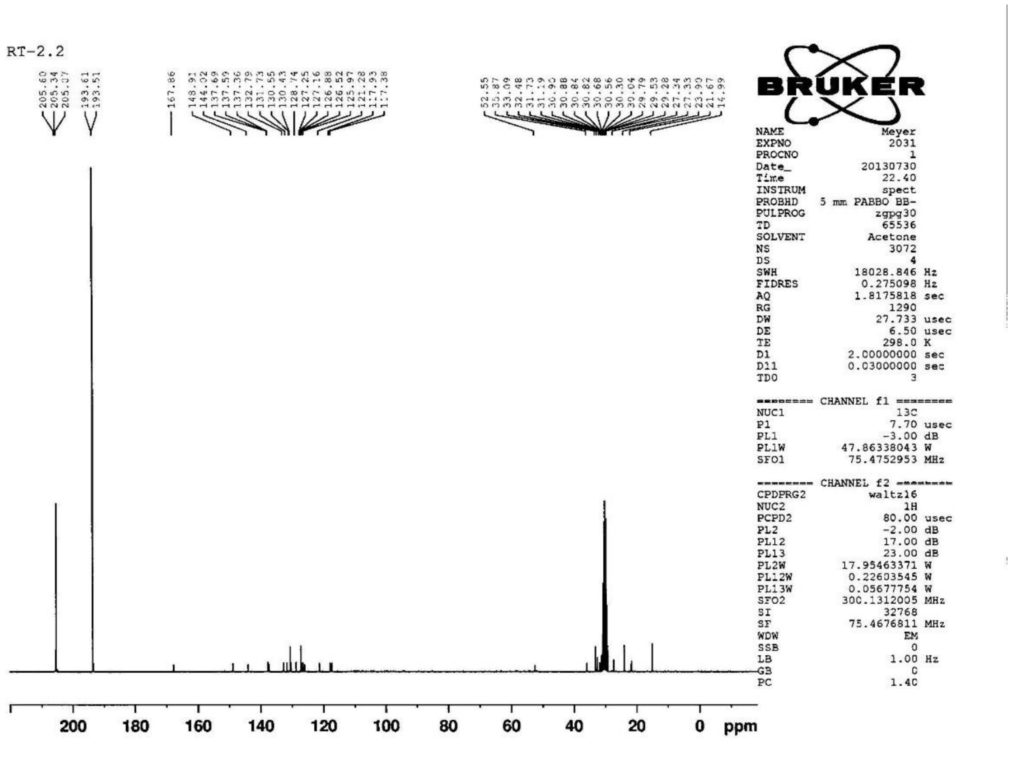

^{13}C NMR (75 MHz, acetone-d$_6$/CS$_2$ 4:1) of compound **12a**.

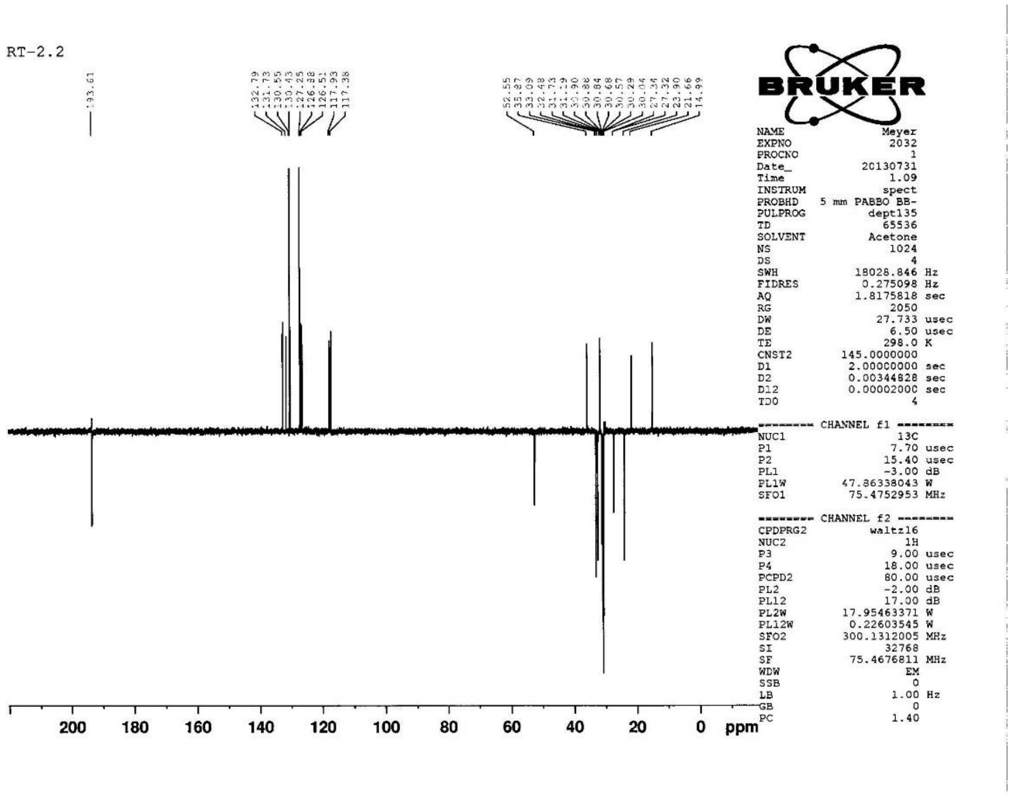

DEPT ^{13}C NMR (75 MHz, acetone-d_6/CS_2 4:1) of compound **12a**.

3.35. (*Z*)-5-{[10-(2-Decyltetradecyl)-7-(*p*-tolyl)-10*H*-phenothiazin-3-yl]methylene}-2-thioxothiazolidin-4-one (12b)

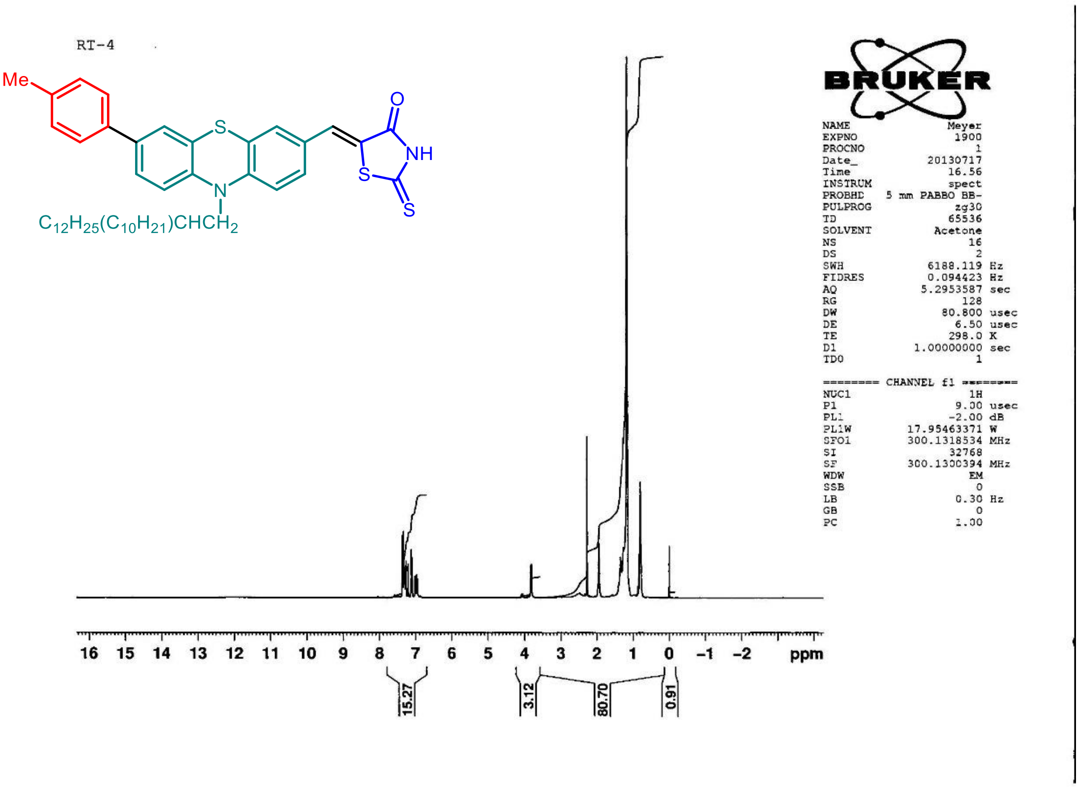

^{1}H NMR (300 MHz, acetone-d_6/CS_2 4:1) of compound **12b**.

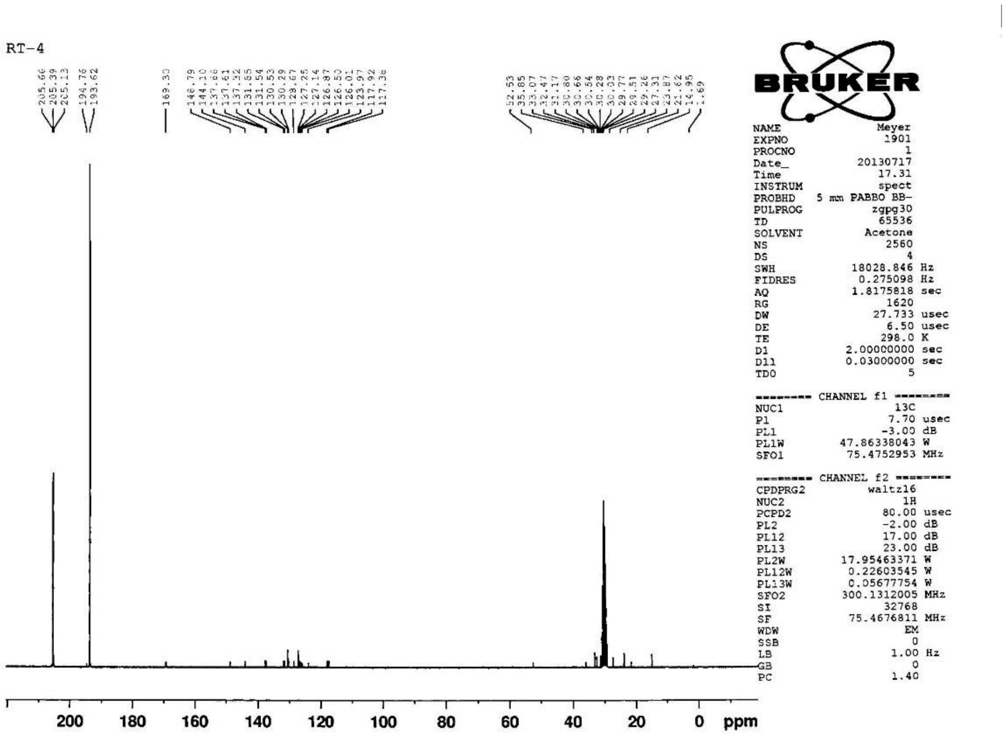

^{13}C NMR (75 MHz, acetone-d_6/CS_2 4:1) of compound **12b**.

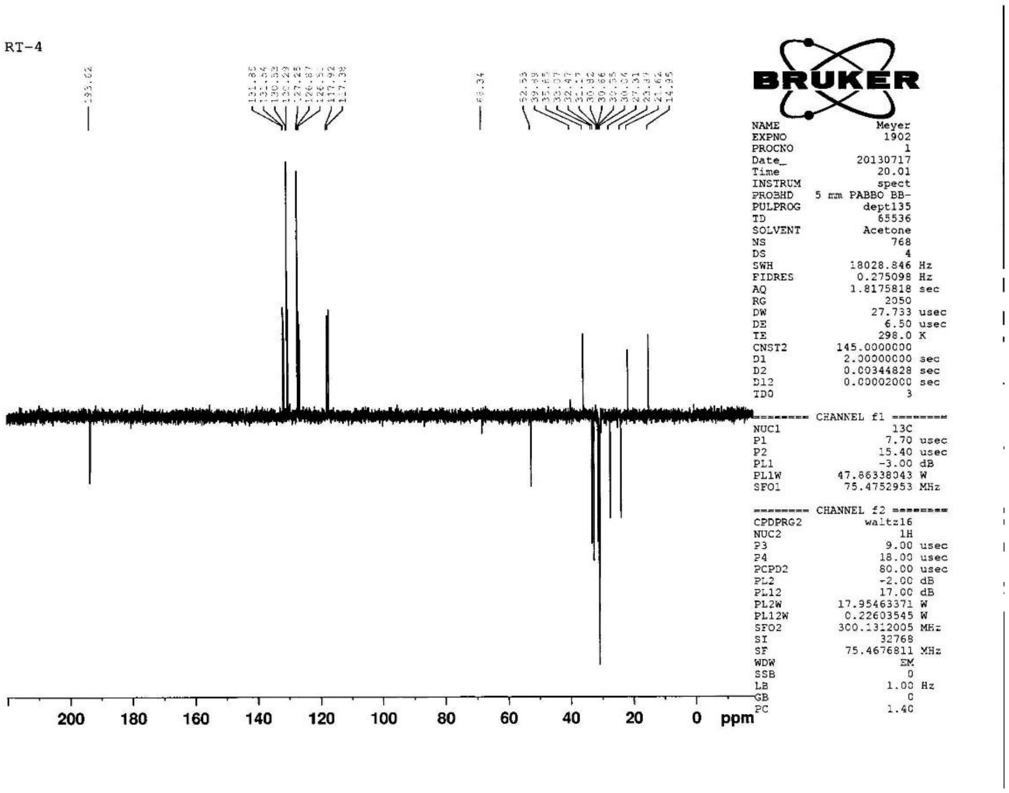

DEPT ^{13}C NMR (75 MHz, acetone-d_6/CS_2 4:1) of compound **12b**.

3.36. 2-{[10-(2-Decyltetradecyl)-7-(*p*-tolyl)-10*H*-phenothiazin-3-yl]methylene}-1*H*-inden-1,3[2*H*]-dione (12c)

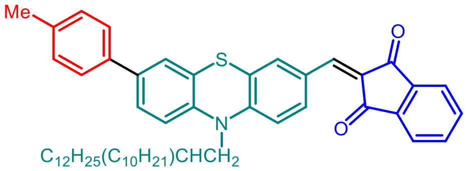

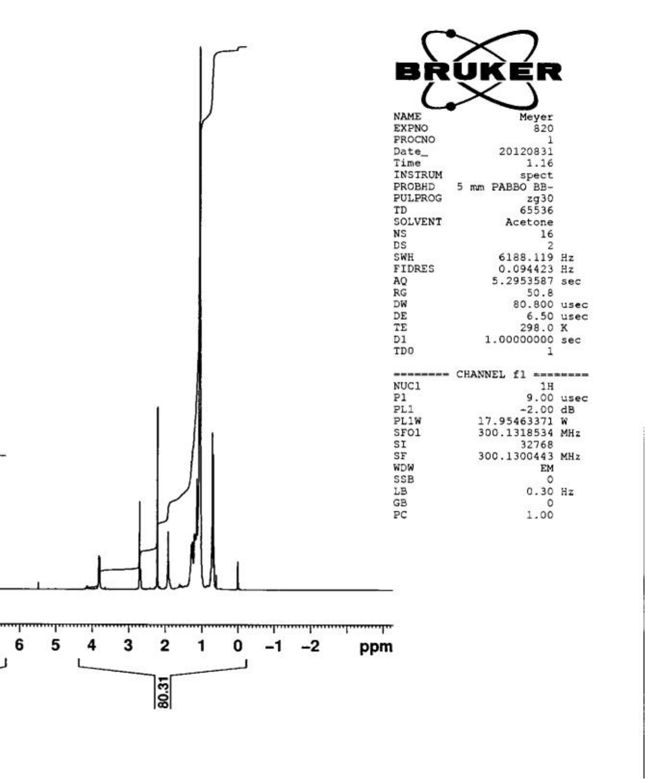

^{1}H NMR (300 MHz, acetone-d$_6$/CS$_2$ 4:1) of compound **12c**.

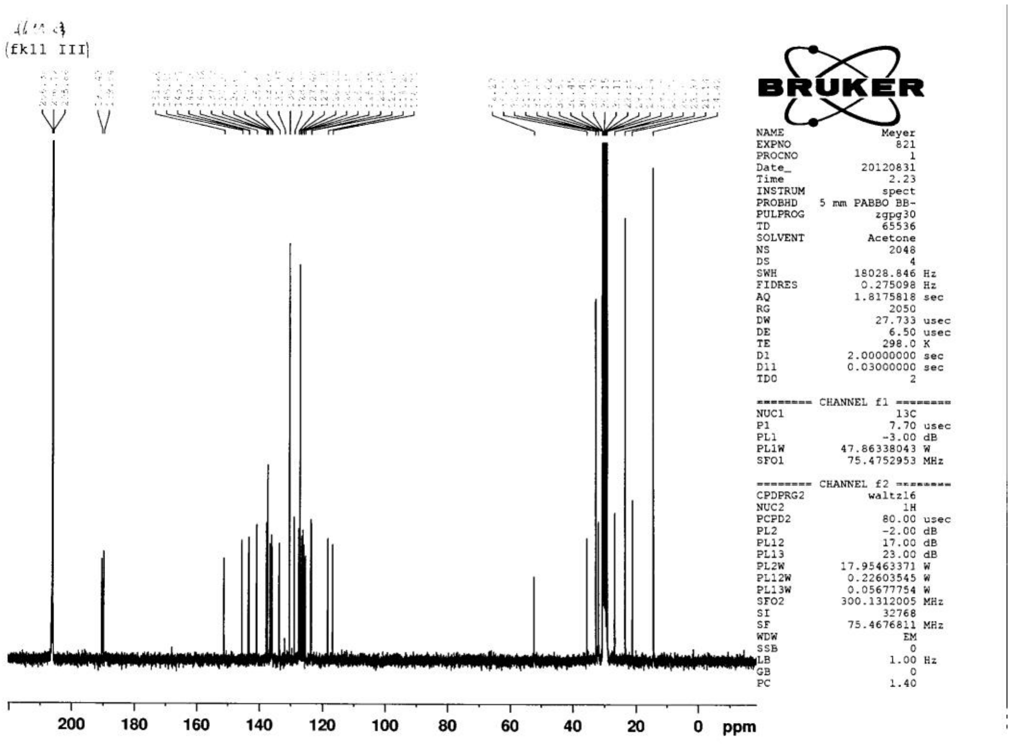

^{13}C NMR (75 MHz, acetone-d$_6$/CS$_2$ 4:1) of compound **12c**.

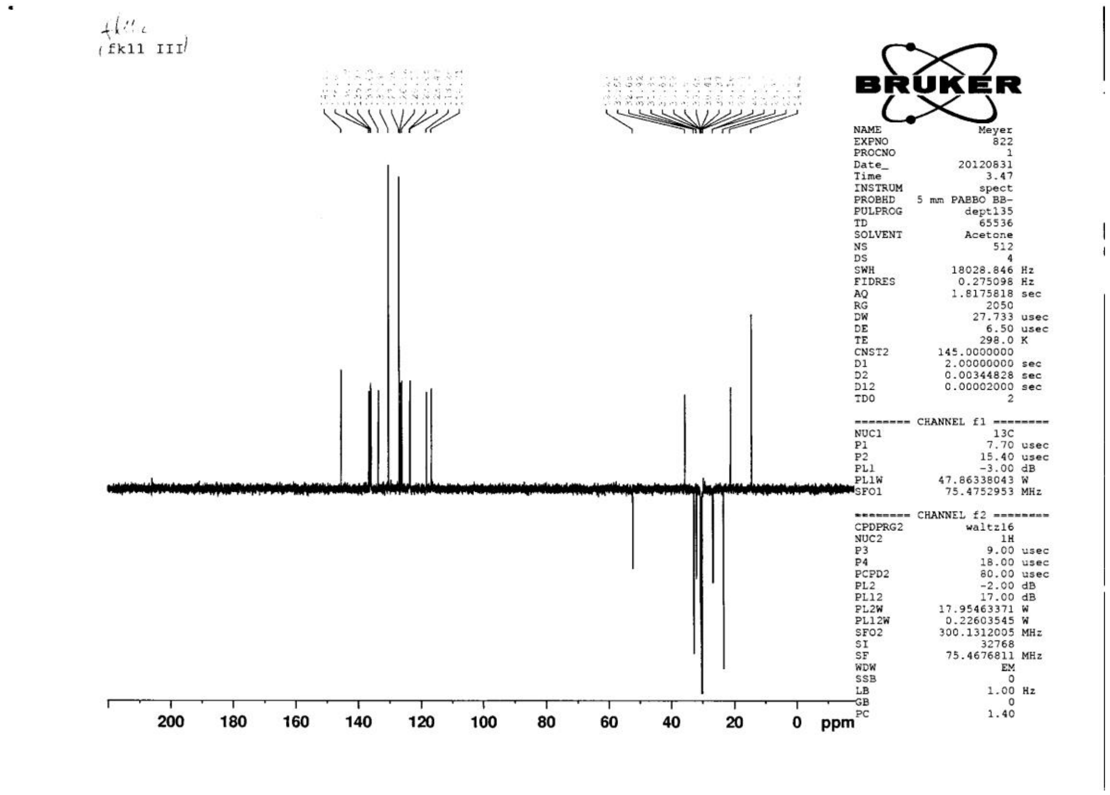

DEPT ^{13}C NMR (75 MHz, acetone-d_6/CS_2 4:1) of compound **12c**.

3.37. 11-[10-(2-Decyltetradecyl)-7-(*p*-tolyl)-10*H*-phenothiazin-3-yl]diindeno[1,2-*b*:2',1'-*e*]pyridin-10,12-dione (12d)

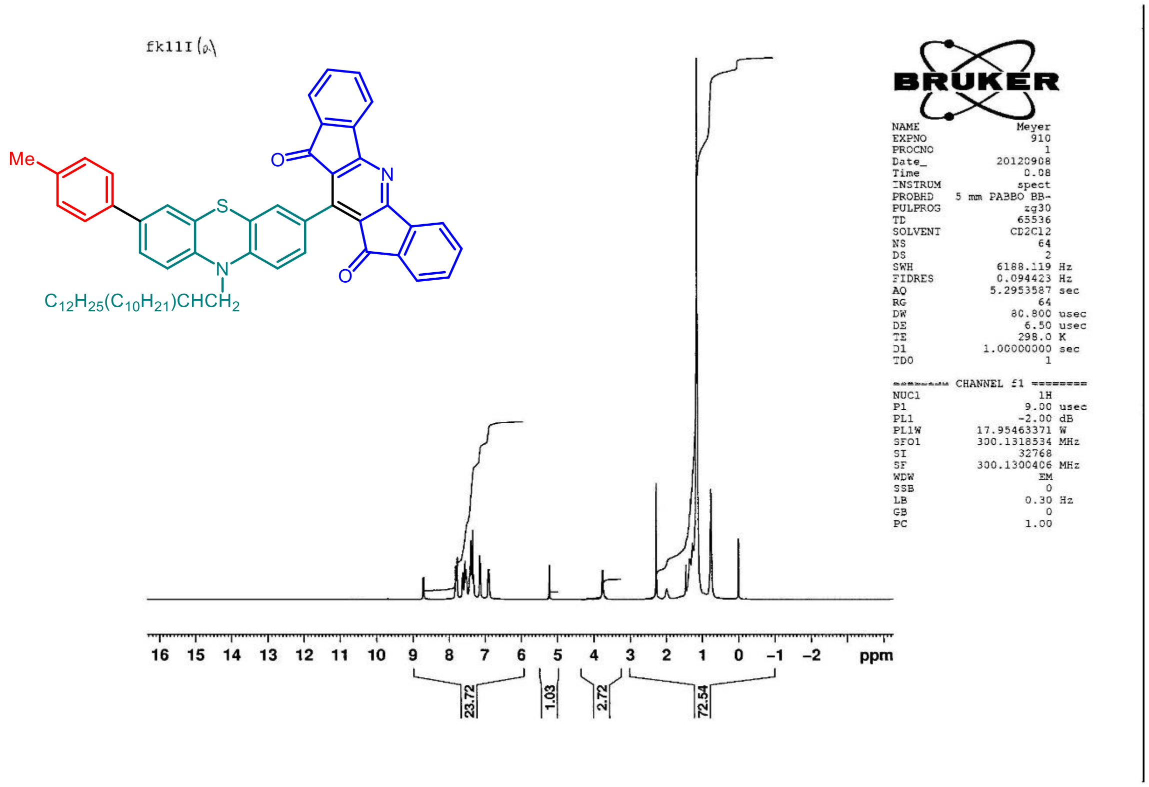

^{1}H NMR (300 MHz, CD_2Cl_2) of compound **12d**.

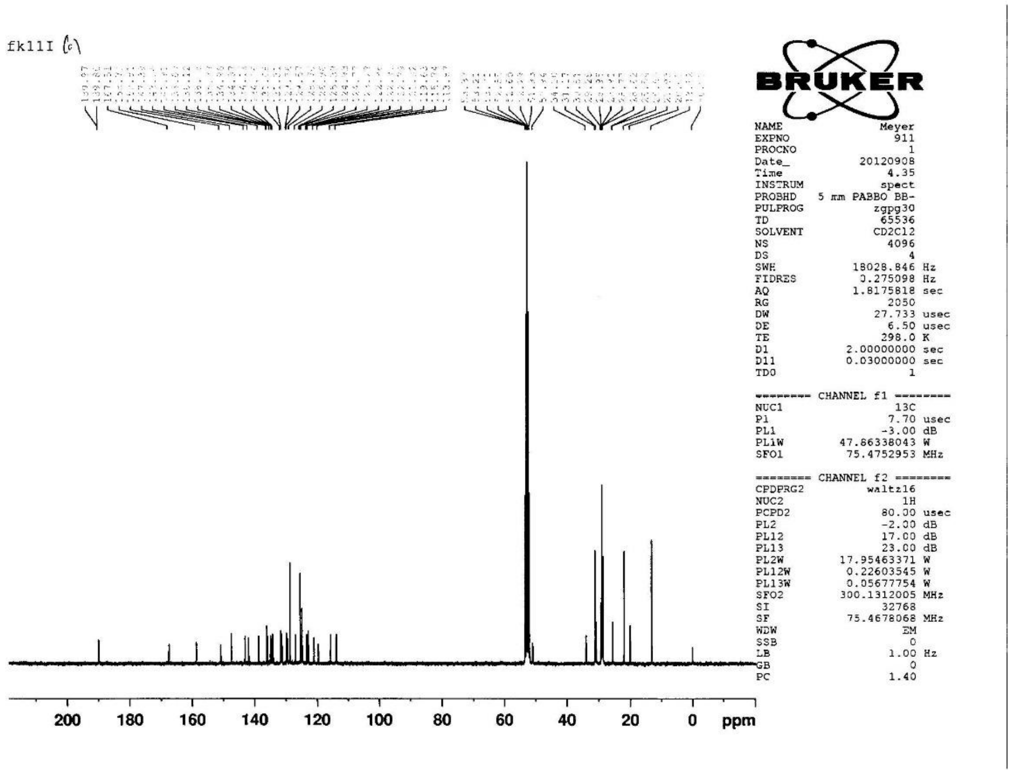

^{13}C NMR (75 MHz, CD_2Cl_2) of compound **12d**.

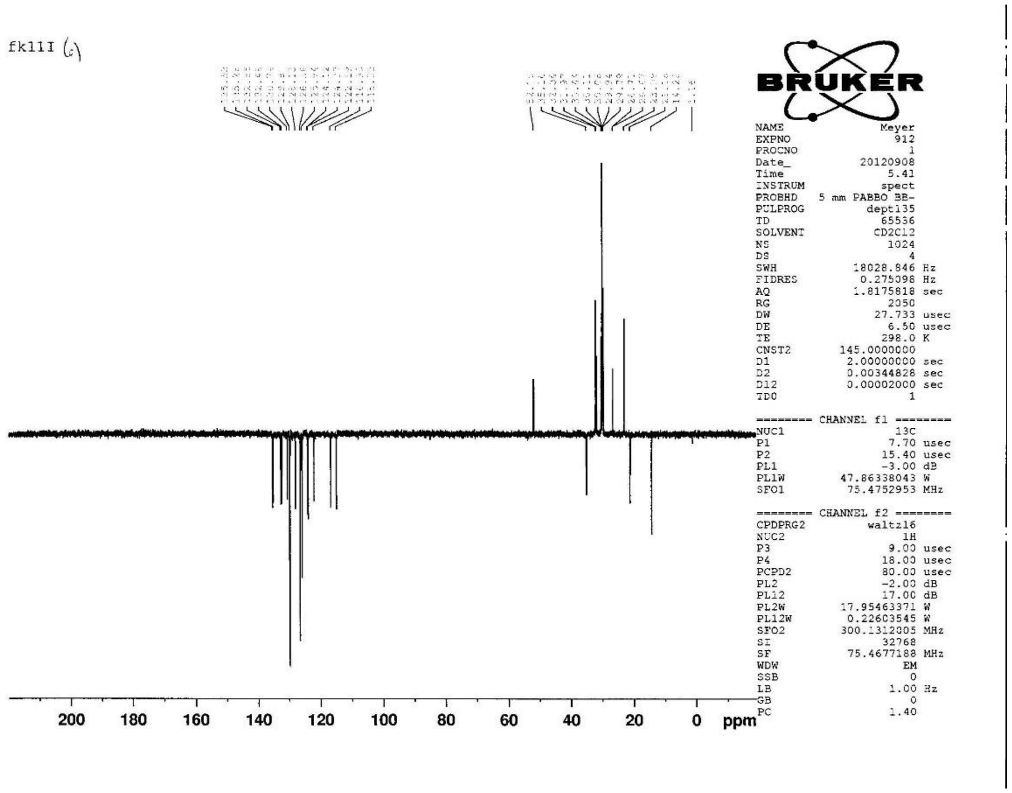

DEPT ^{13}C NMR (75 MHz, CD_2Cl_2) of compound **12d**.

3.38. (*Z*)-4-[(10-(2-Decyltetradecyl)-7-(*p*-tolyl)-10*H*-phenothiazin-3-yl)methylene]-3-methyl-1-phenyl-1*H*-pyrazol-5[4*H*]-one (12e)

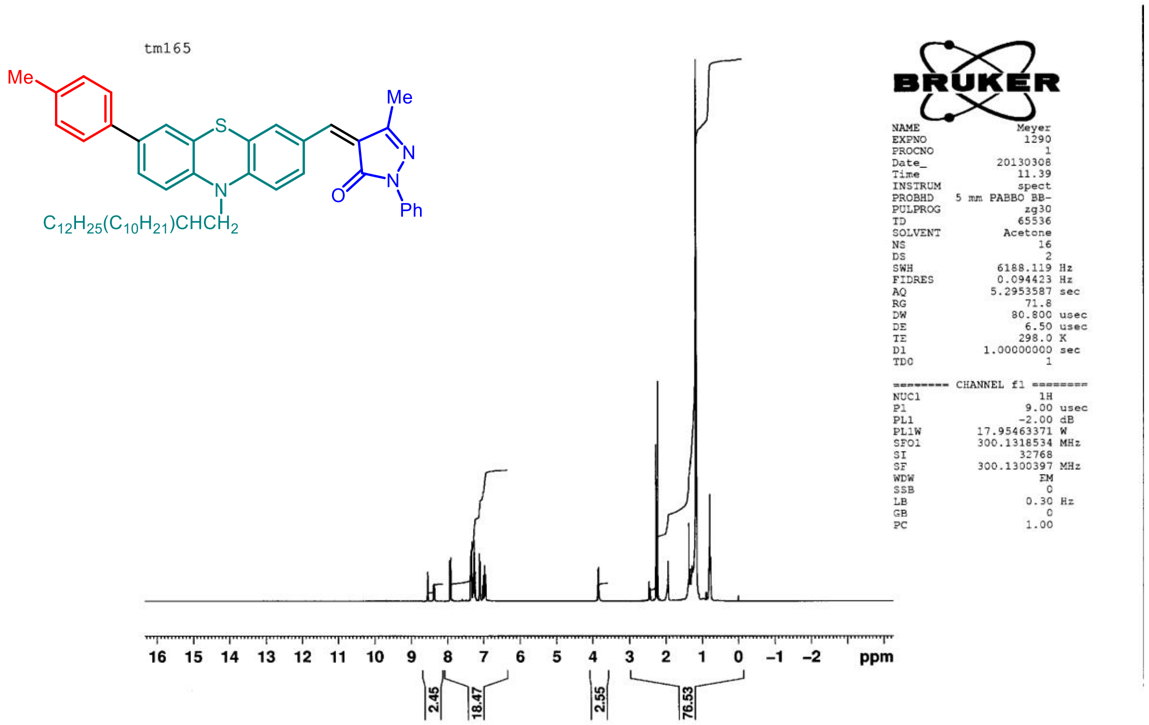

^{1}H NMR (300 MHz, acetone-d_6/CS_2 4:1) of compound **12e**.

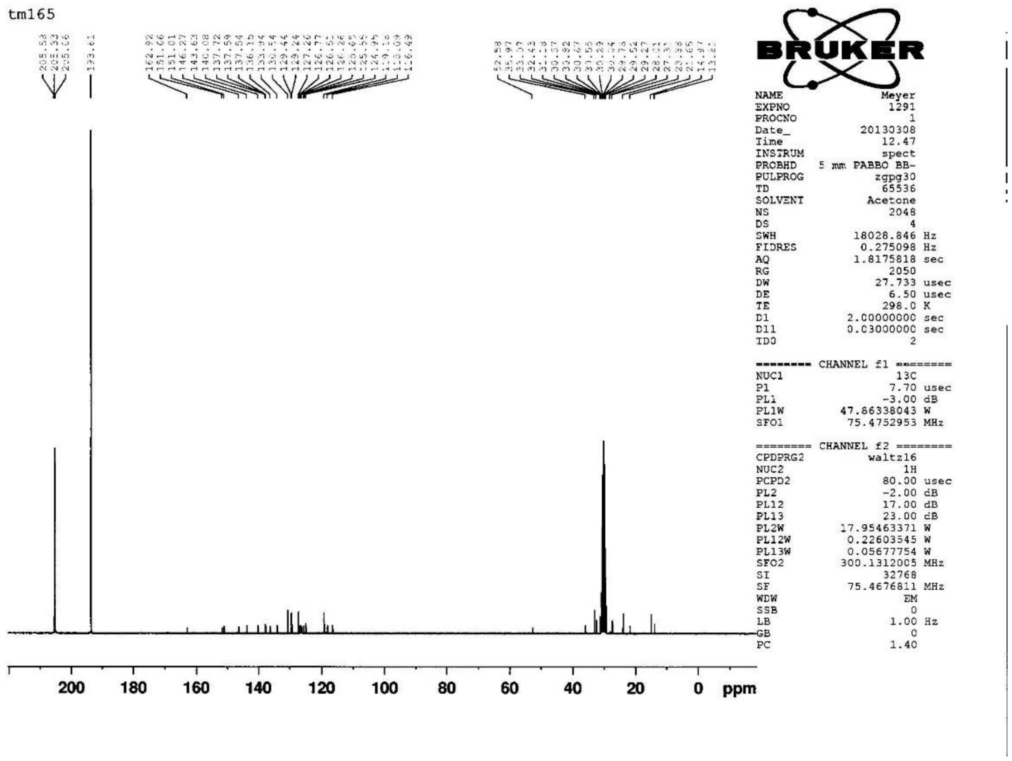

^{13}C NMR (75 MHz, acetone-d_6/CS_2 4:1) of compound **12e**.

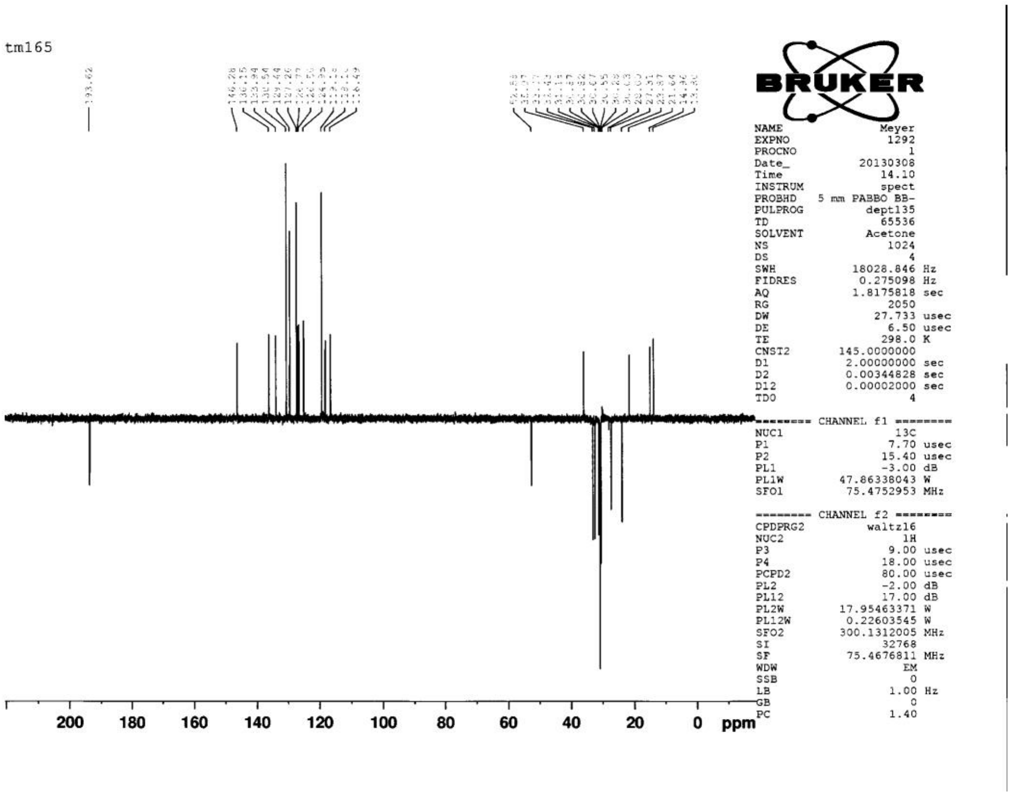

DEPT ^{13}C NMR (75 MHz, acetone-d_6/CS_2 4:1) of compound **12e**.

3.39. (*Z*)-3-[10-(2-Decyltetradecyl)-7-(*p*-tolyl)-10H-phenothiazin-3-yl]-2-(4-nitrophenyl)acrylonitrile (12f)

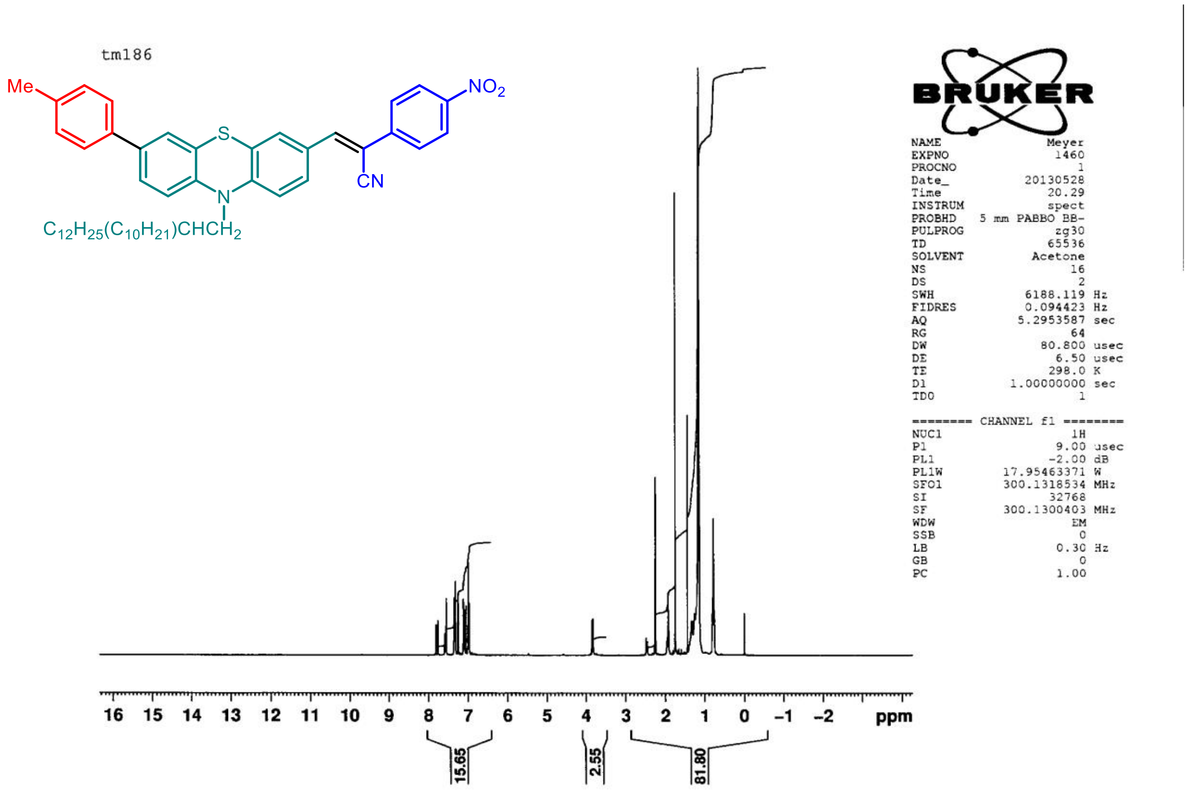

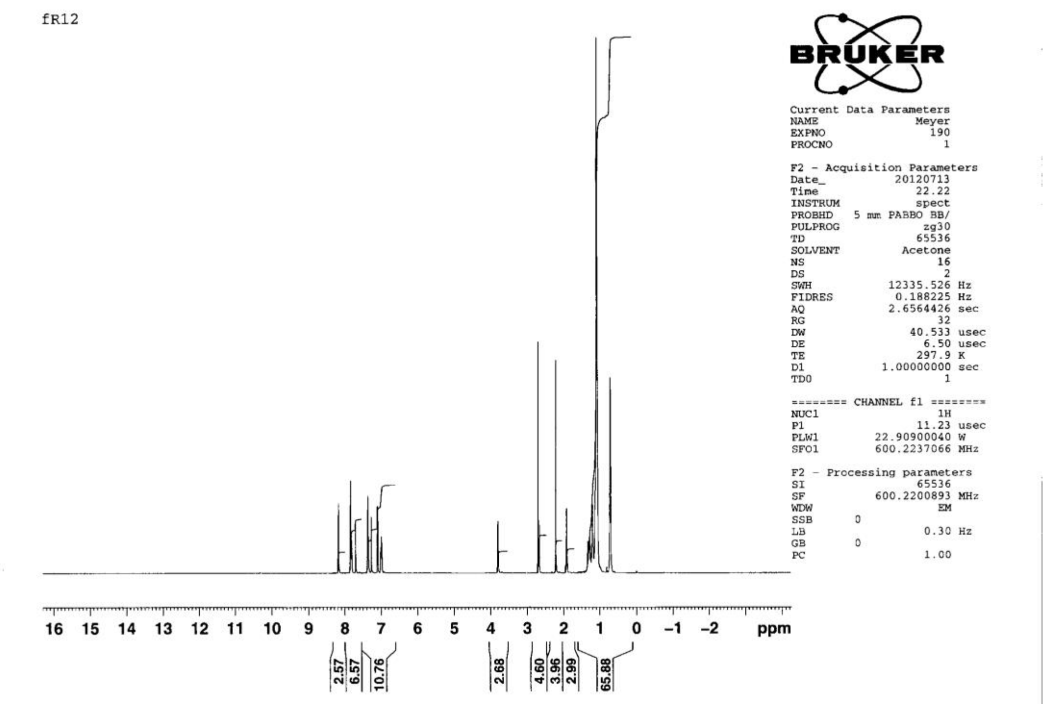

^{1}H NMR (600 MHz, acetone-d$_6$) of compound **12f**.

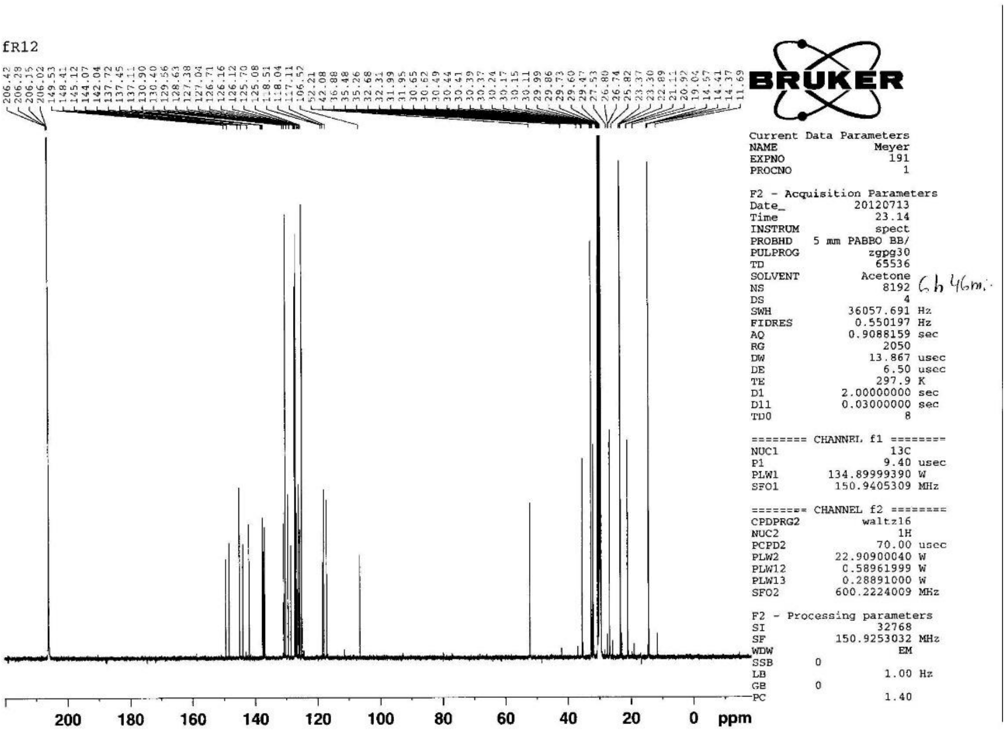

^{13}C NMR (150 MHz, acetone-d_6) of compound **12f**.

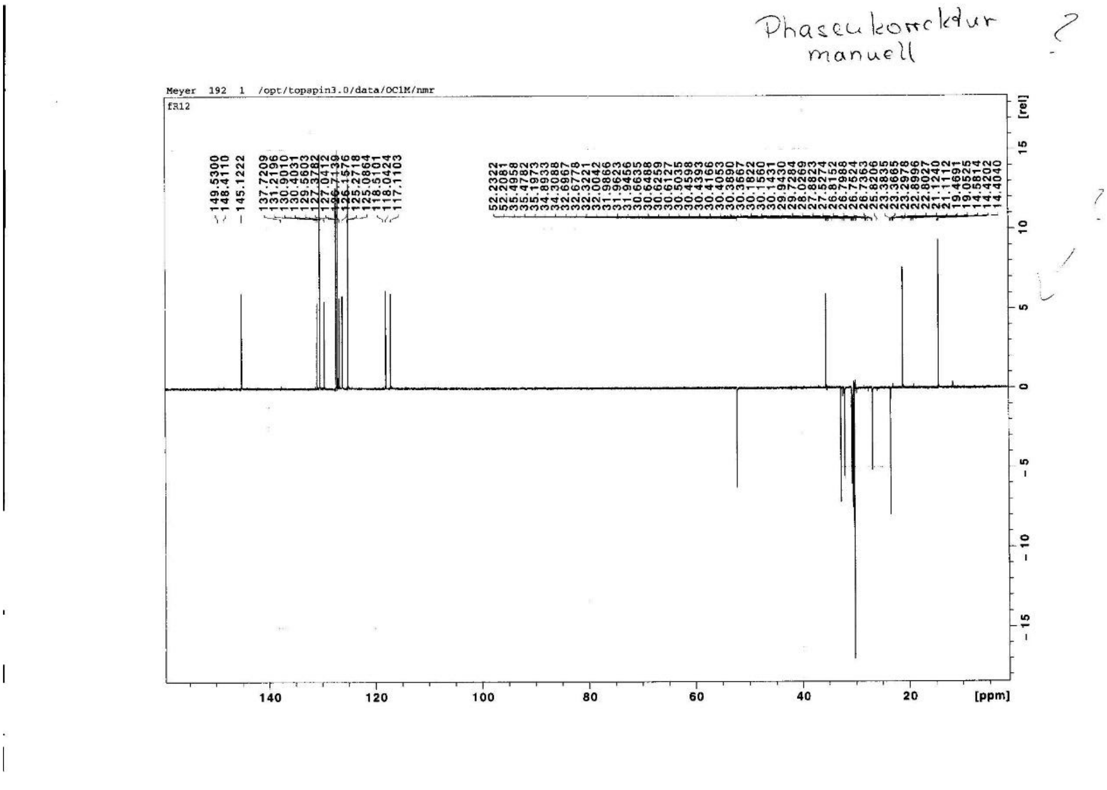

DEPT ^{13}C NMR (150 MHz, acetone-d_6) of compound **12f**.

3.40. (*E*)-2-{3-Cyano-4-[2-(10-{2-decyltetradecyl}-7-{*p*-tolyl}-10*H*-phenothiazin-3-yl)vinyl]-5,5-dimethylfuran-2[5*H*]-yliden}malonitrile (12g)

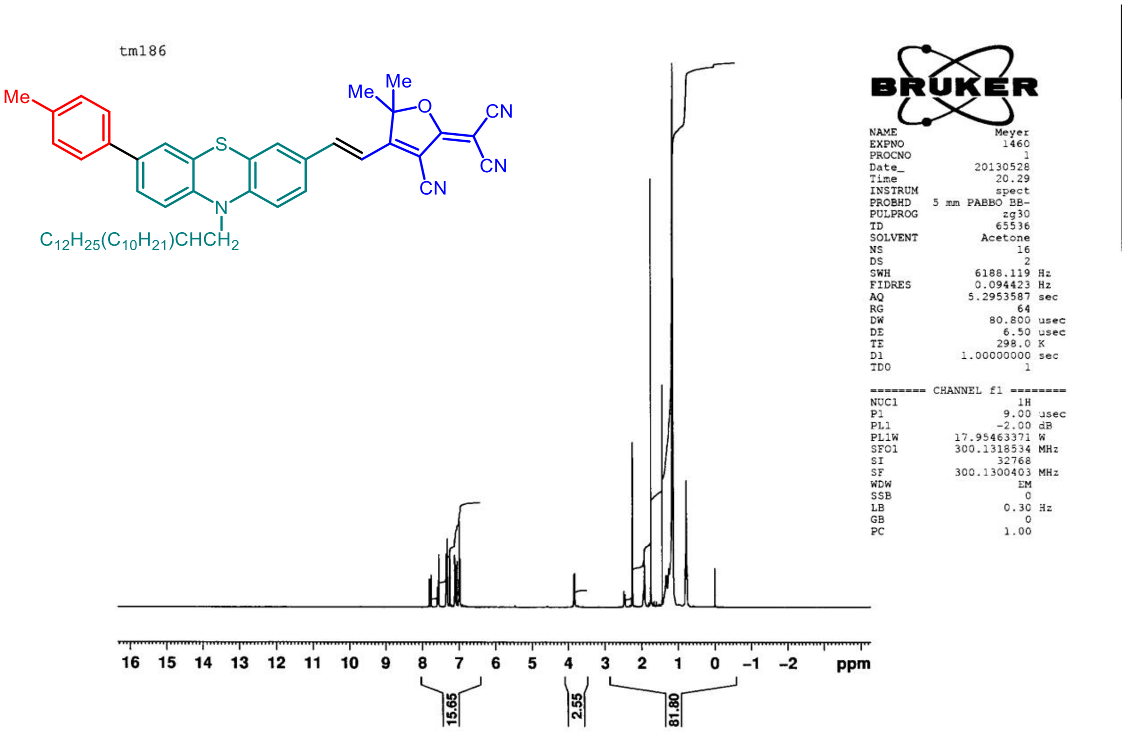

^{1}H NMR (300 MHz, acetone-d$_6$/CS$_2$ 4:1) of compound **12g**.

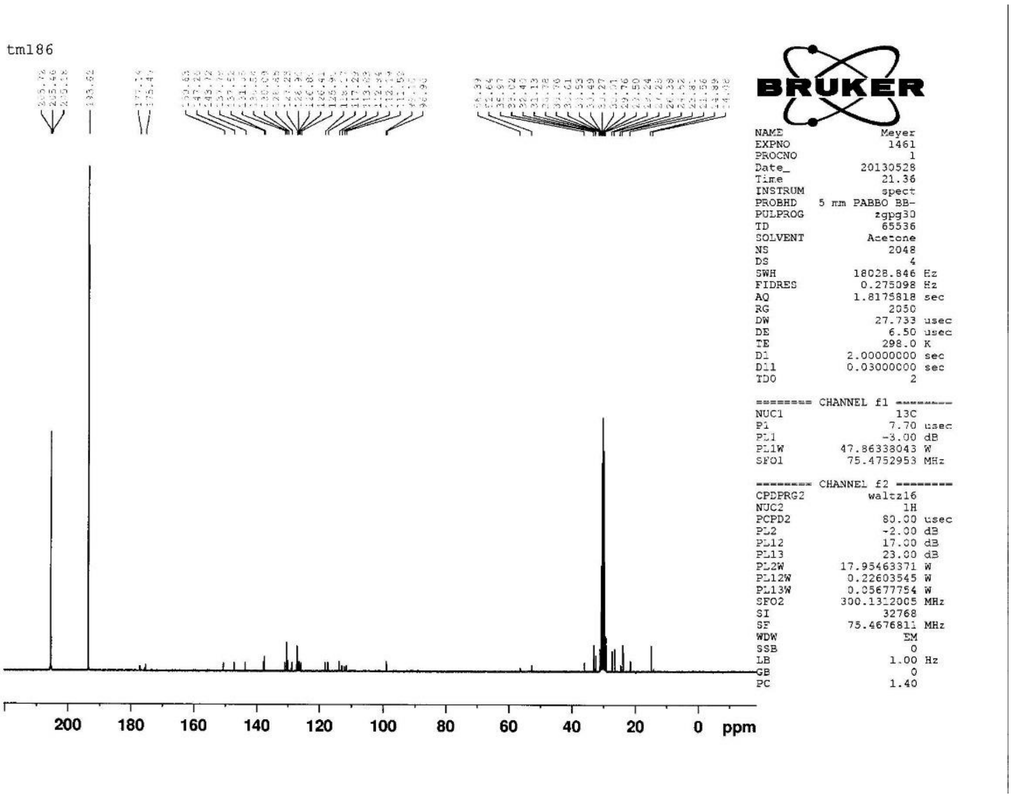

^{13}C NMR (75 MHz, acetone-d$_6$/CS$_2$ 4:1) of compound **12g**.

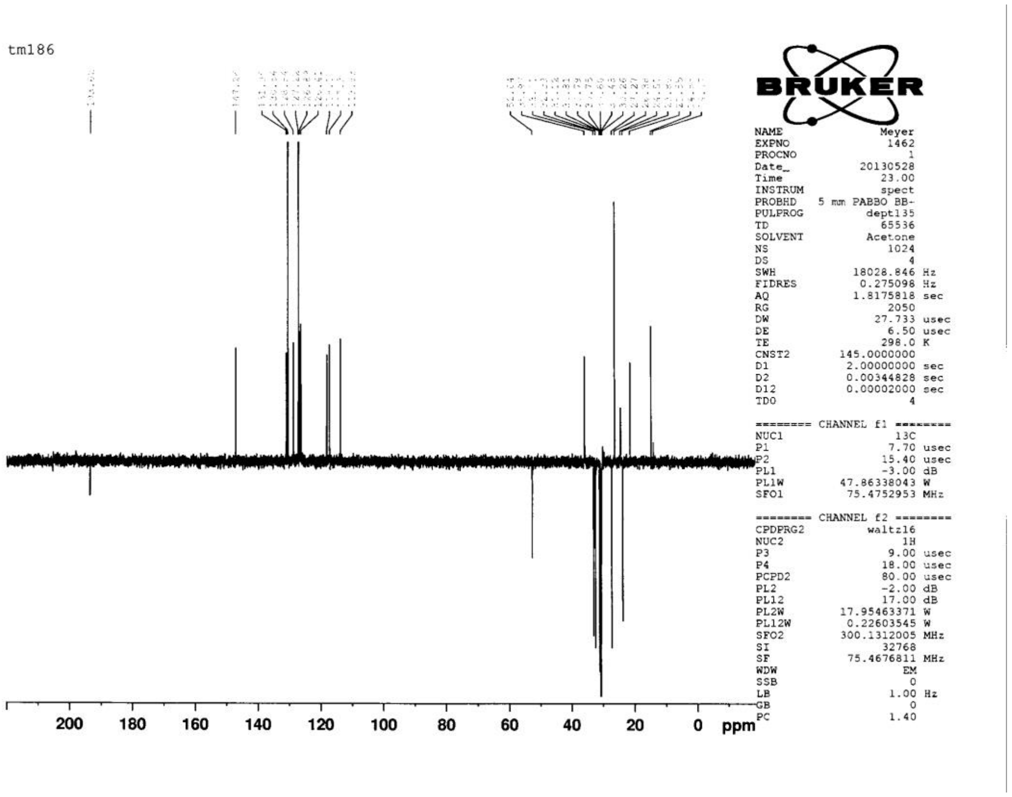

DEPT ^{13}C NMR (75 MHz, acetone-d_6/CS_2 4:1) of compound **12g**.

3.41. (*Z*)-5-{[10-(2-Decyltetradecyl)-7-(*p*-tolyl)-10*H*-phenothiazin-3-yl]methylene}-3-methyl-2-thioxothiazolidin-4-one (12h)

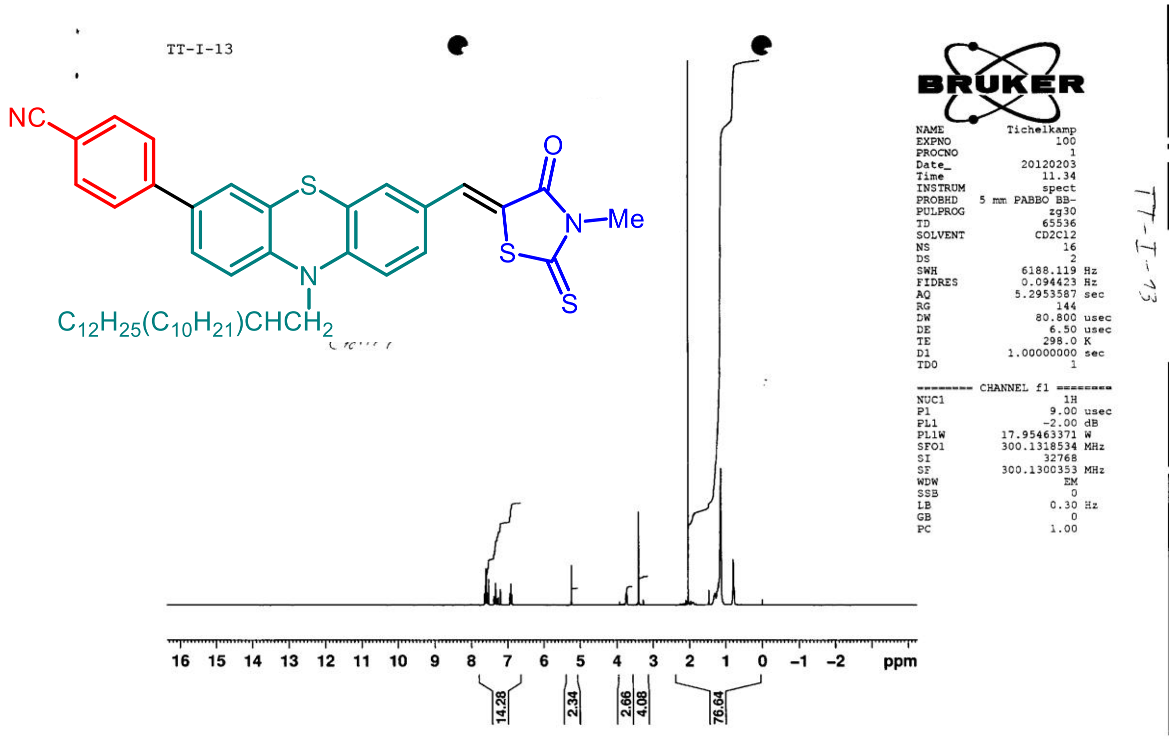

^{1}H NMR (300 MHz, CD_2Cl_2) of compound **12h**.

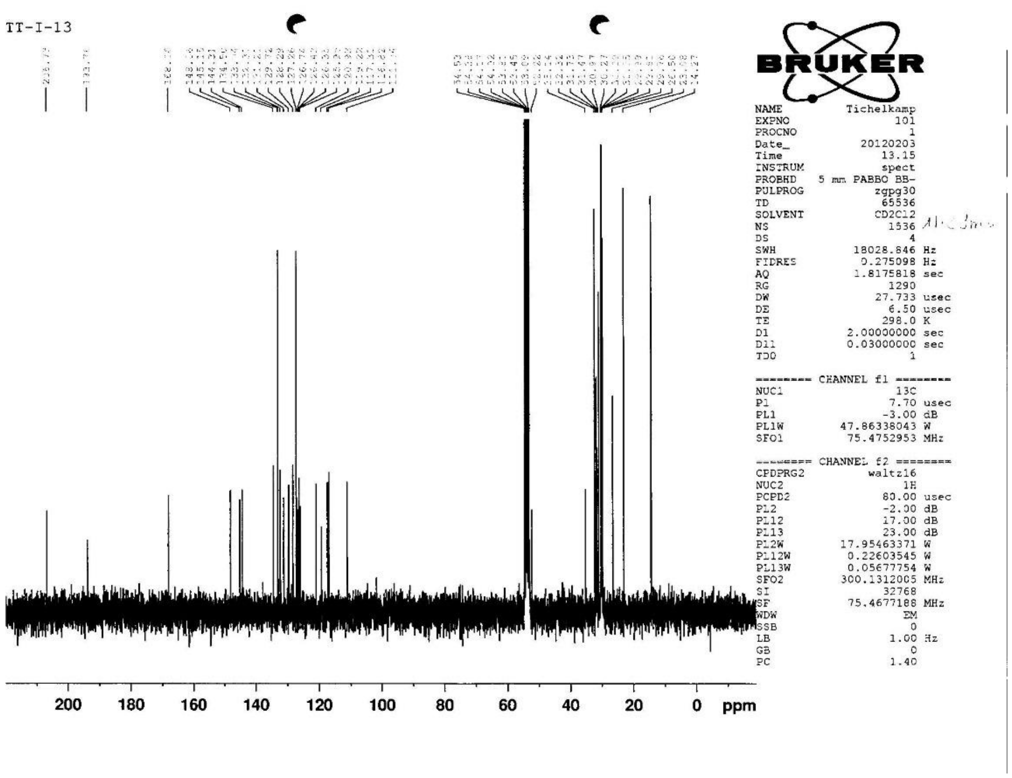

^{13}C NMR (75 MHz, CD_2Cl_2) of compound **12h**.

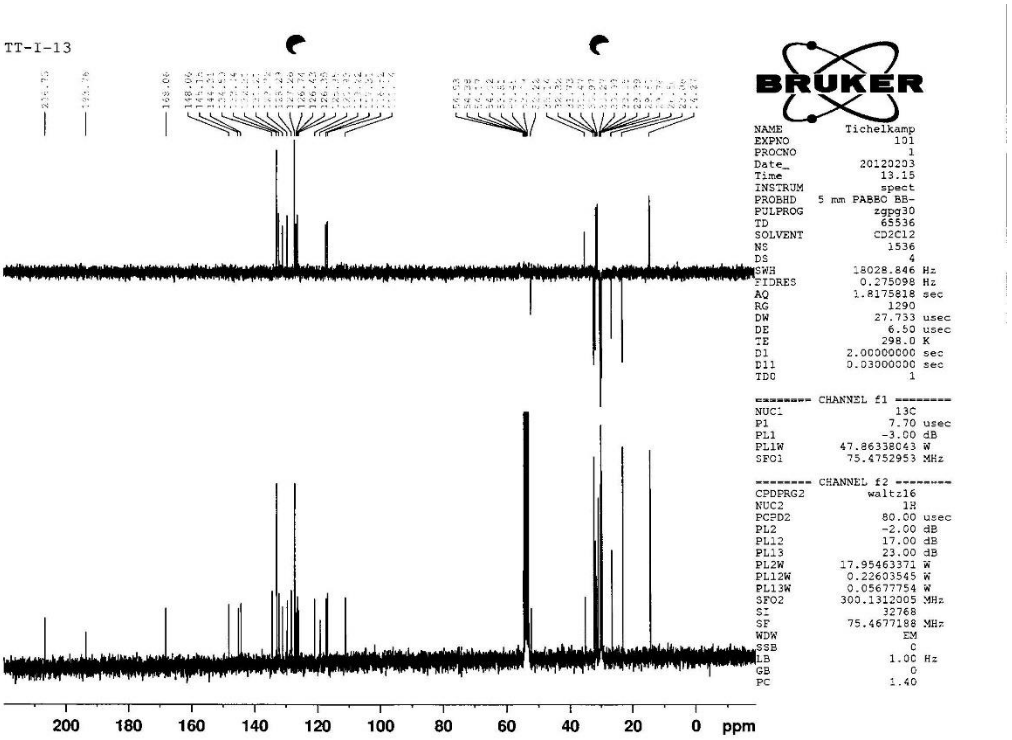

DEPT ^{13}C NMR (75 MHz, CD_2Cl_2) of compound **12h**.

3.42. 4-{10-(2-Decyltetradecyl)-7-[(1,3-dioxo-1H-inden-2[3*H*]-yliden)methyl]-10*H*-phenothiazin-3-yl}benzonitrile (12i)

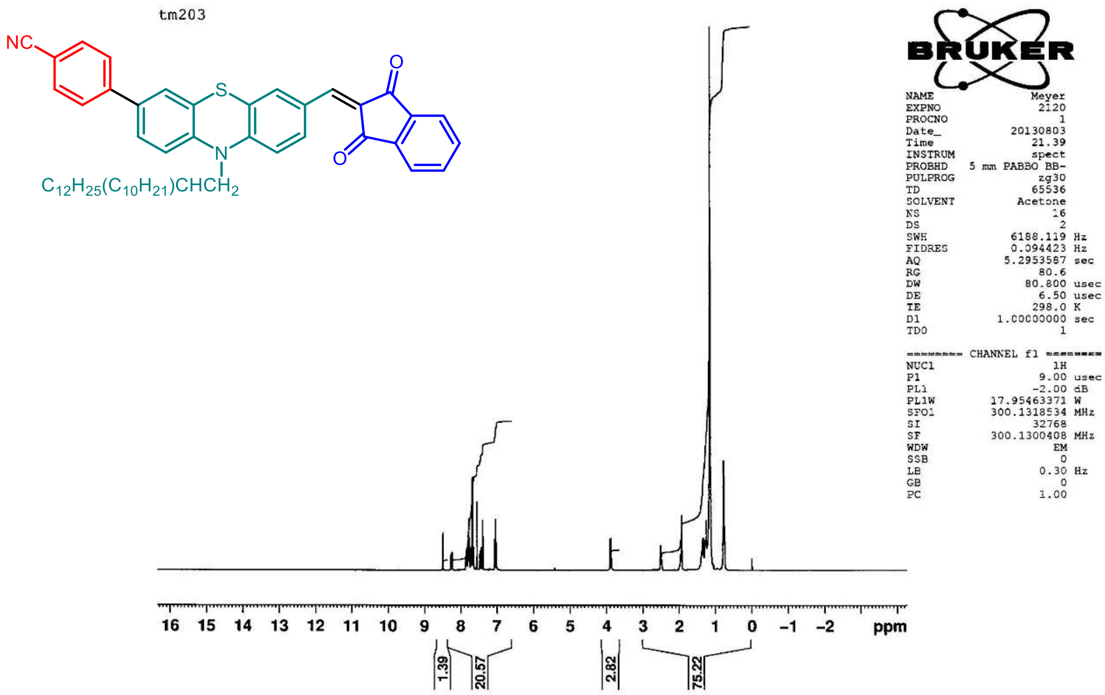

^{1}H NMR (300 MHz, acetone-d_6/CS_2 4:1) of compound **12i**.

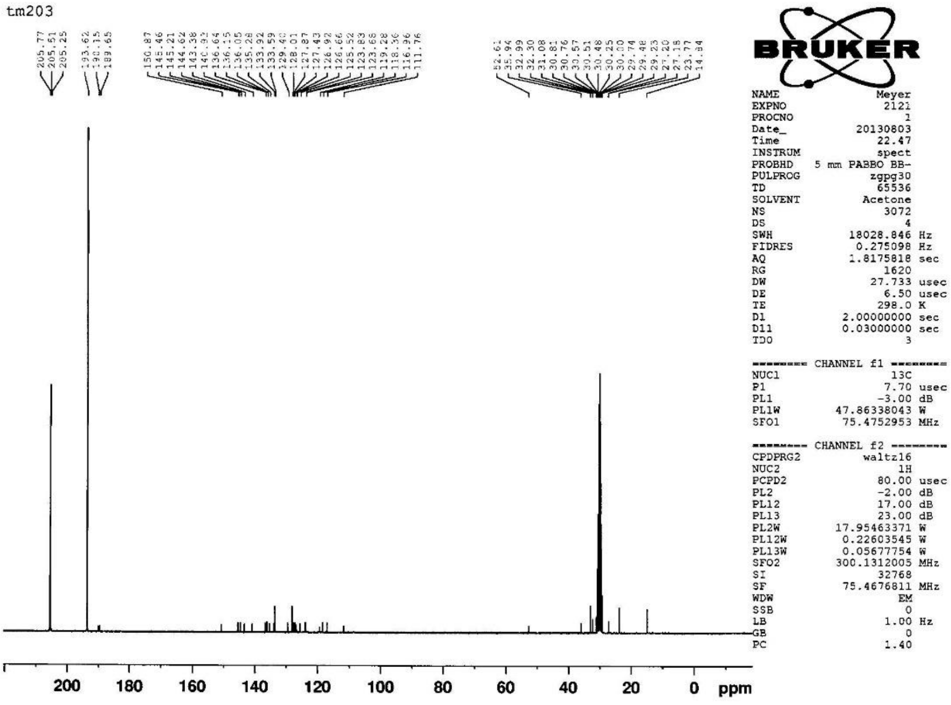

^{13}C NMR (75 MHz, acetone-d_6/CS_2 4:1) of compound **12i**.

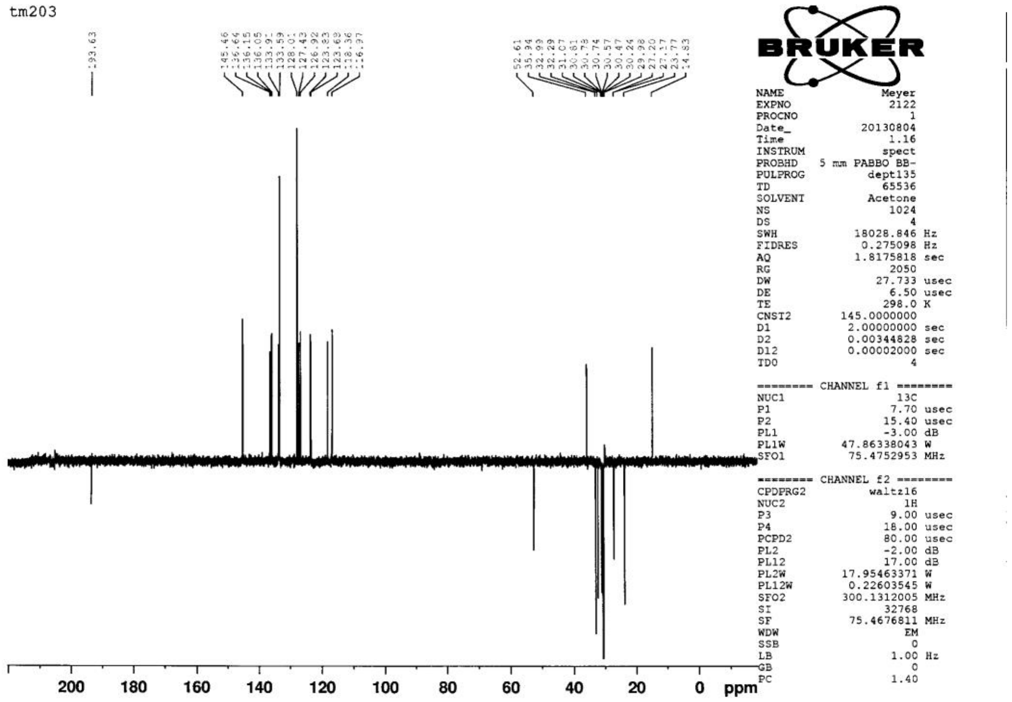

DEPT ^{13}C NMR (75 MHz, acetone-d_6/CS_2 4:1) of compound **12i**.

3.43. (Z)-5-{[10-(2-Decyltetradecyl)-7-(pyridin-4-yl)-10*H*-phenothiazin-3-yl]methylene}-3-methyl-2-thioxothiazolidin-4-one (12j)

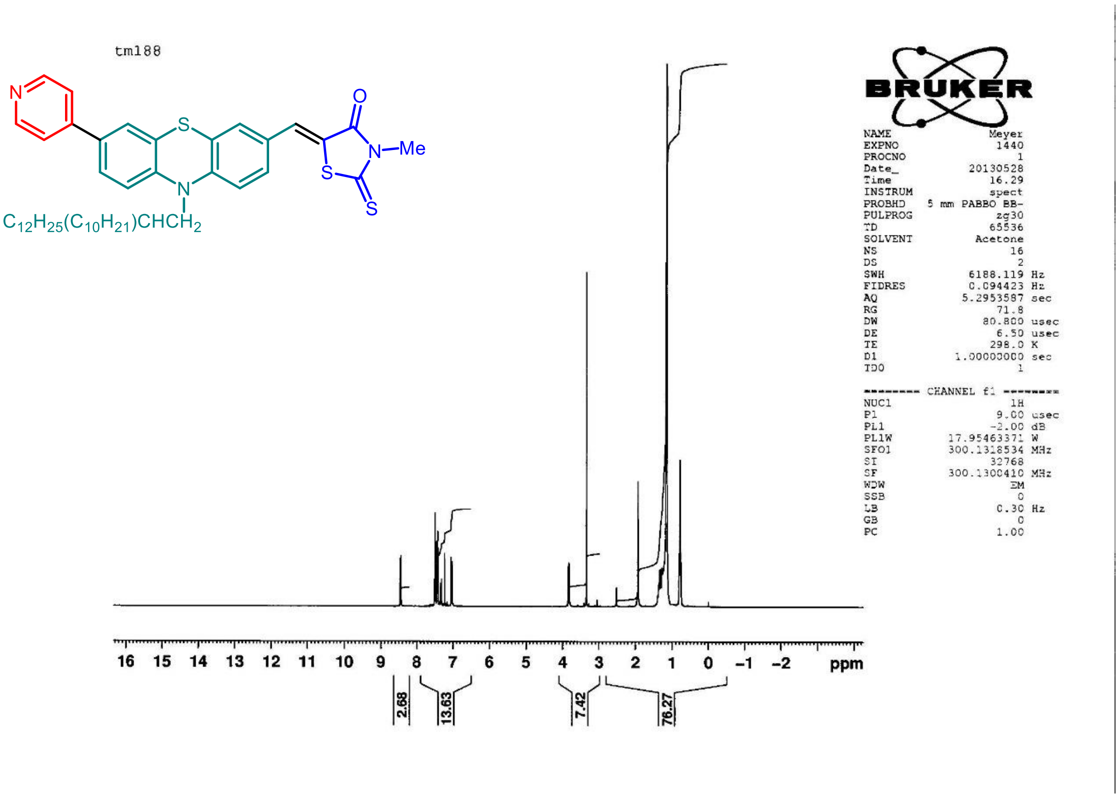

^{1}H NMR (300 MHz, acetone-d$_6$/CS$_2$ 4:1) of compound **12j**.

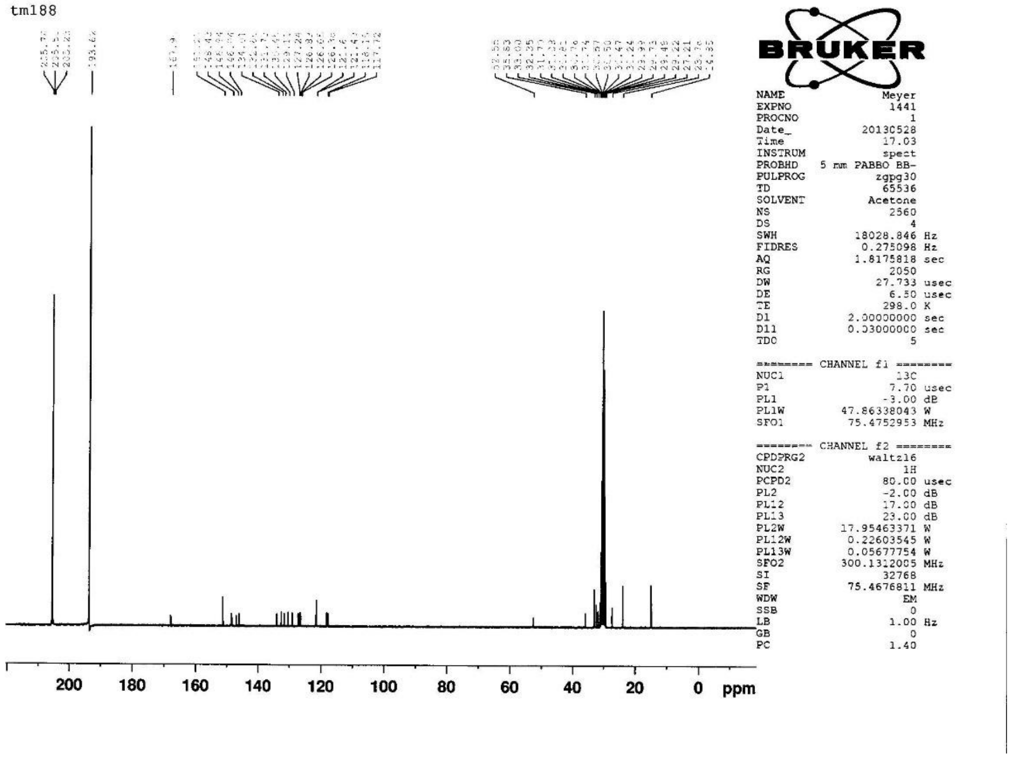

^{13}C NMR (75 MHz, acetone-d$_6$/CS$_2$ 4:1) of compound **12j**.

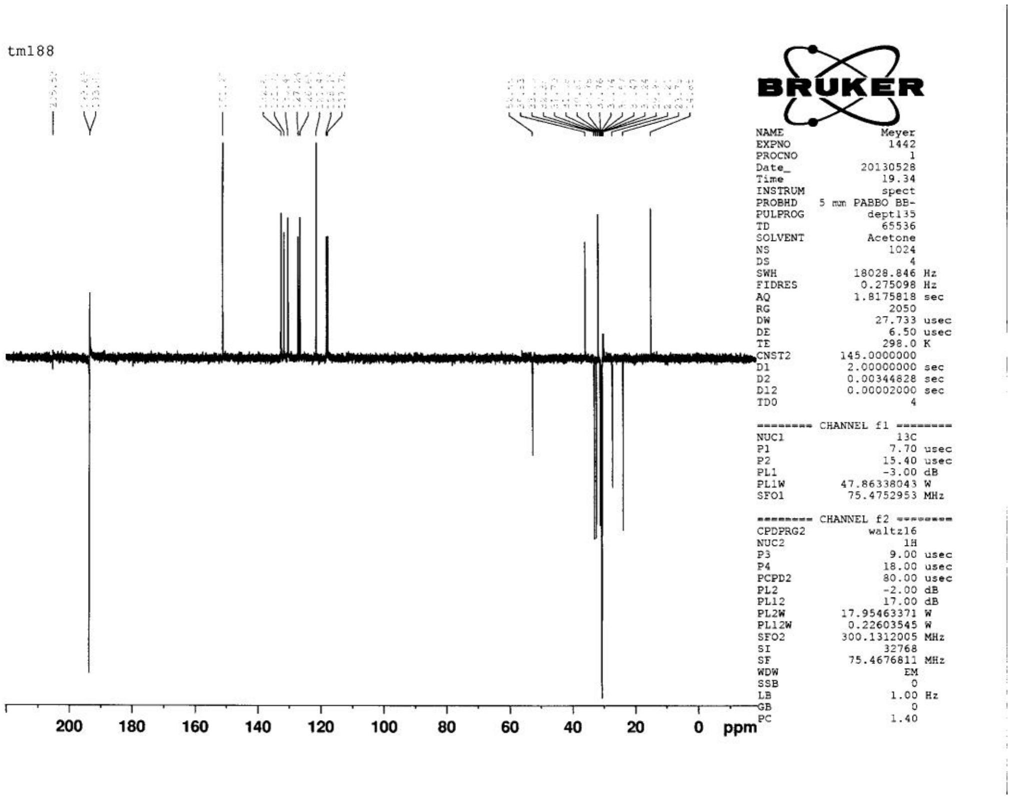

DEPT ^{13}C NMR (75 MHz, acetone-d_6/CS_2 4:1) of compound **12j**.

3.44. (*Z*)-5-{[10-(2-Decyltetradecyl)-7-(1-methyl-1*H*-pyrazol-4-yl)-10*H*-phenothiazin-3-yl]methylene}-3-methyl-2-thioxothiazolidin-4-one (12k)

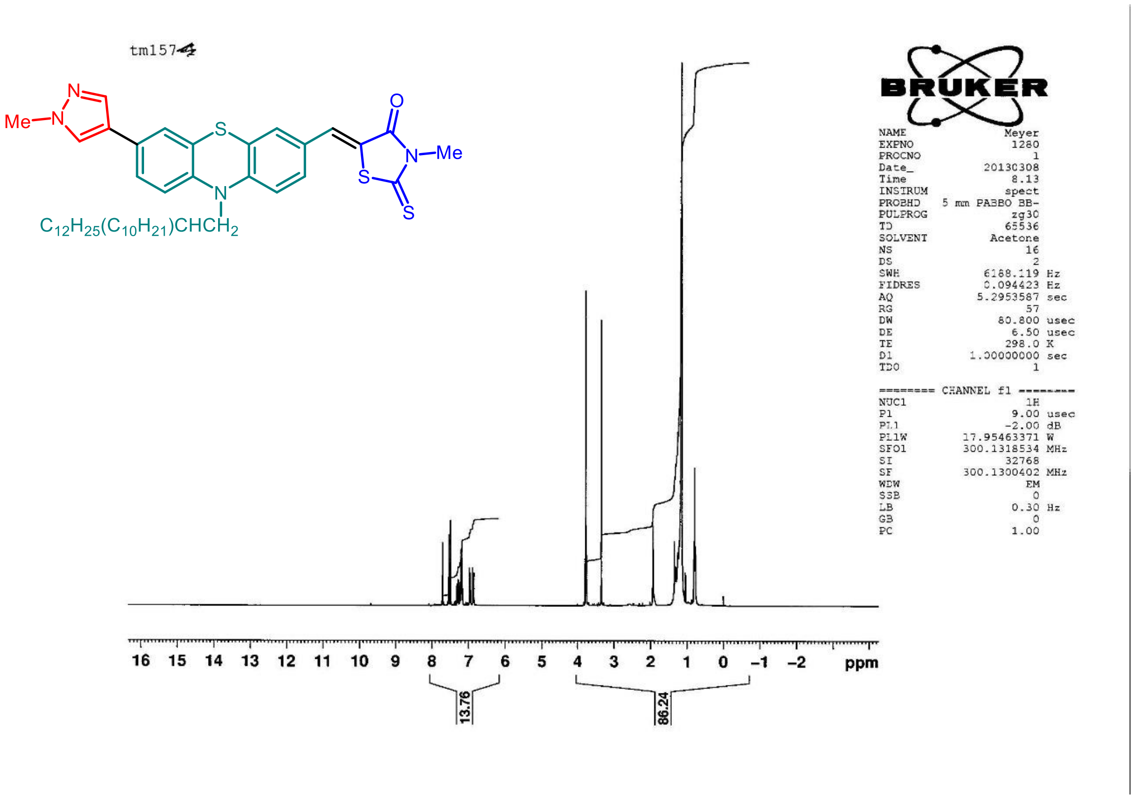

^{1}H NMR (300 MHz, acetone-d$_6$/CS$_2$ 4:1) of compound **12k**.

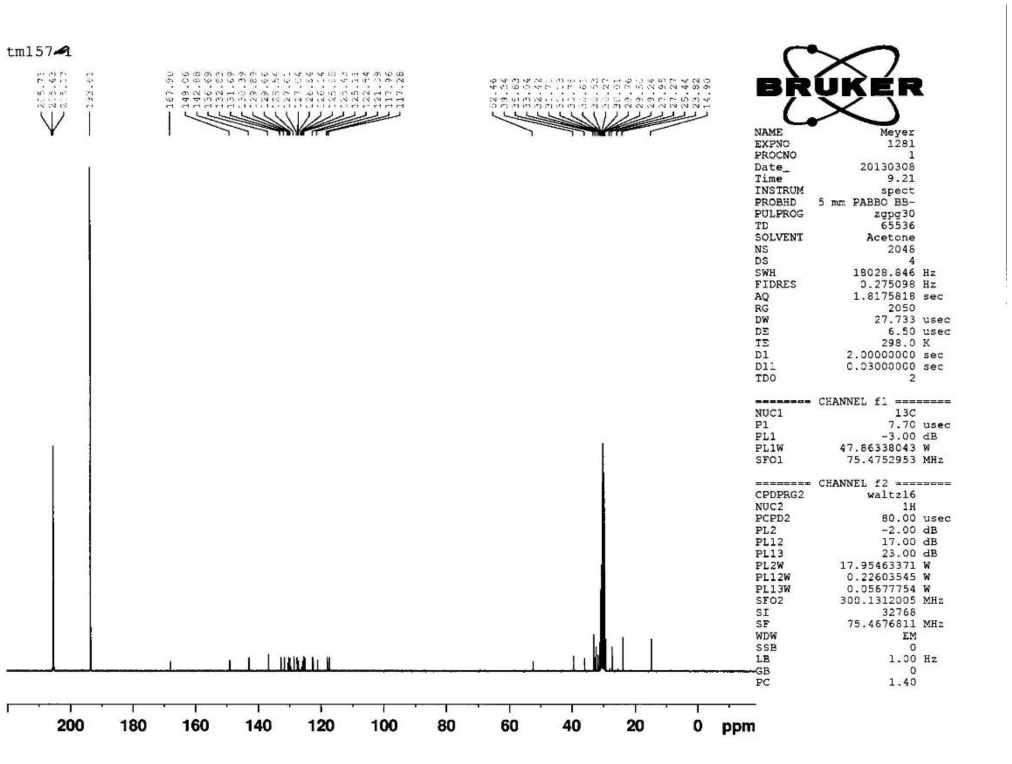

^{13}C NMR (75 MHz, acetone-d$_6$/CS$_2$ 4:1) of compound **12k**.

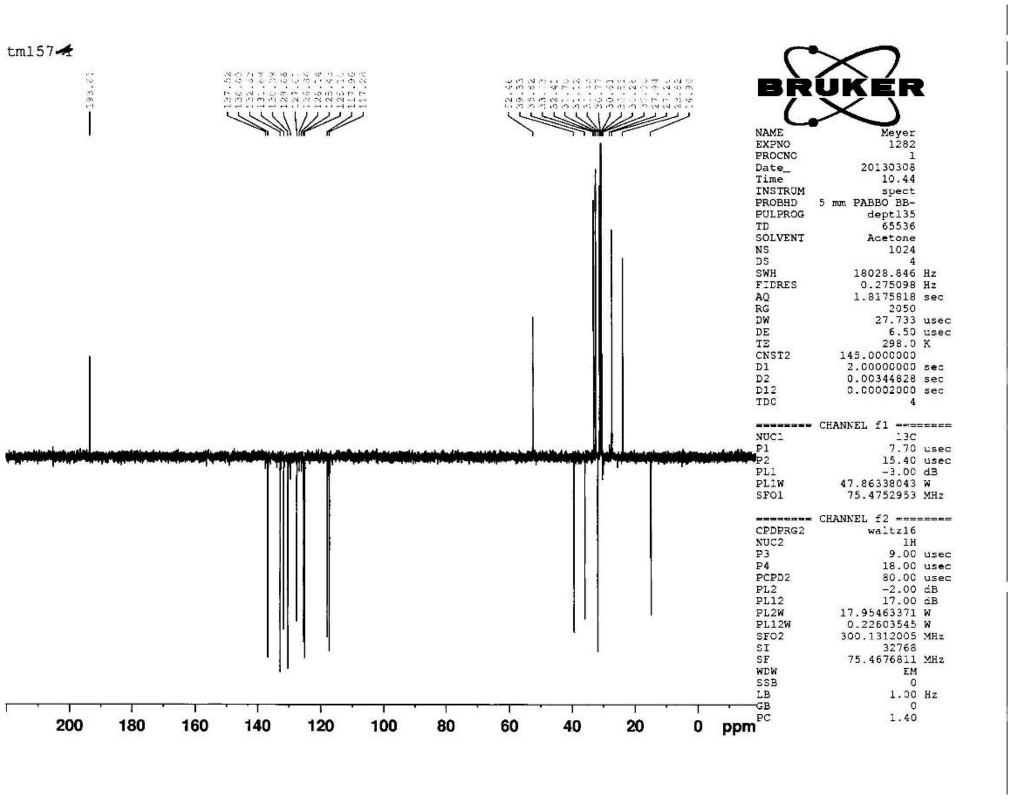

DEPT ^{13}C NMR (75 MHz, acetone-d_6/CS_2 4:1) of compound **12k**.

3.45. (*Z*)-3-[10-(2-Decyltetradecyl)-7-(1-methyl-1*H*-pyrazol-4-yl)-10*H*-phenothiazin-3-yl]-2-(4-nitrophenyl)acrylonitrile (12l)

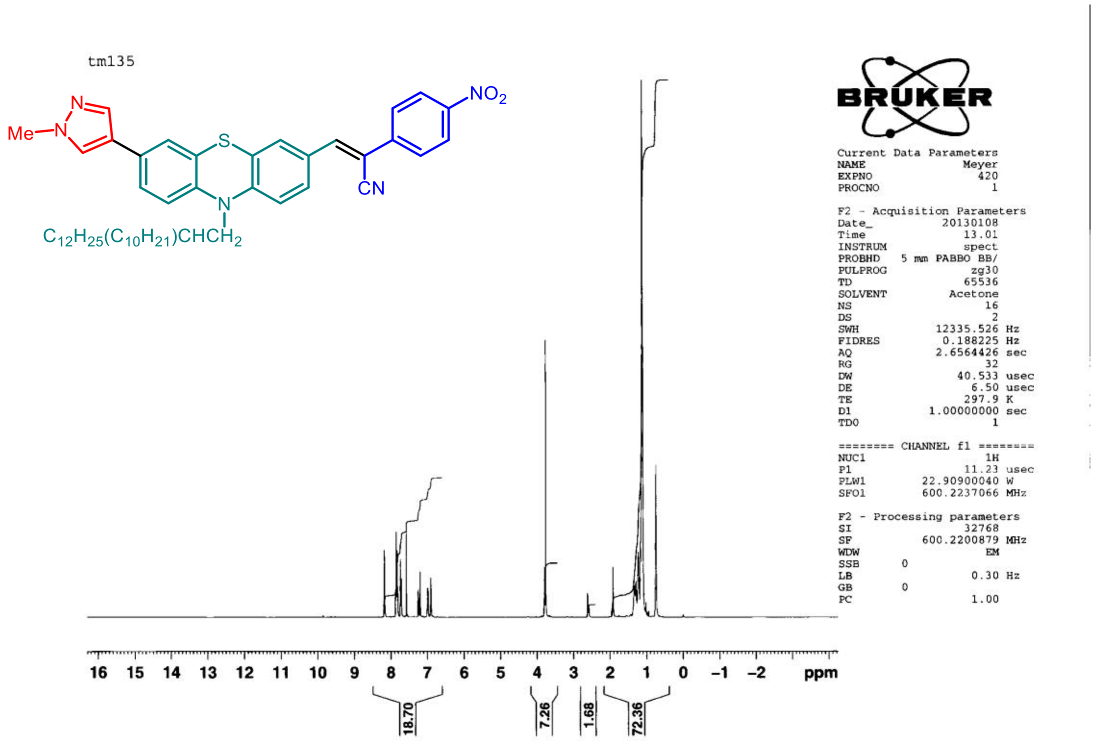

^{1}H NMR (300 MHz, acetone-d_6) of compound **12l**.

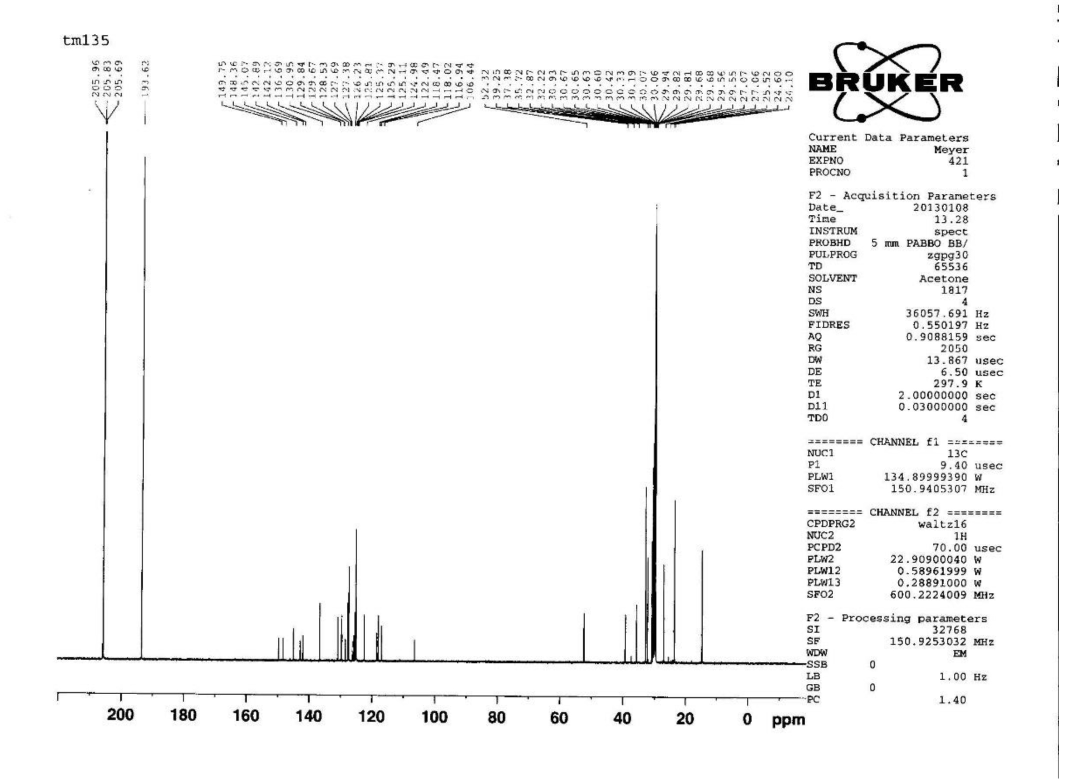

^{13}C NMR (150 MHz, acetone-d_6) of compound **12l**.

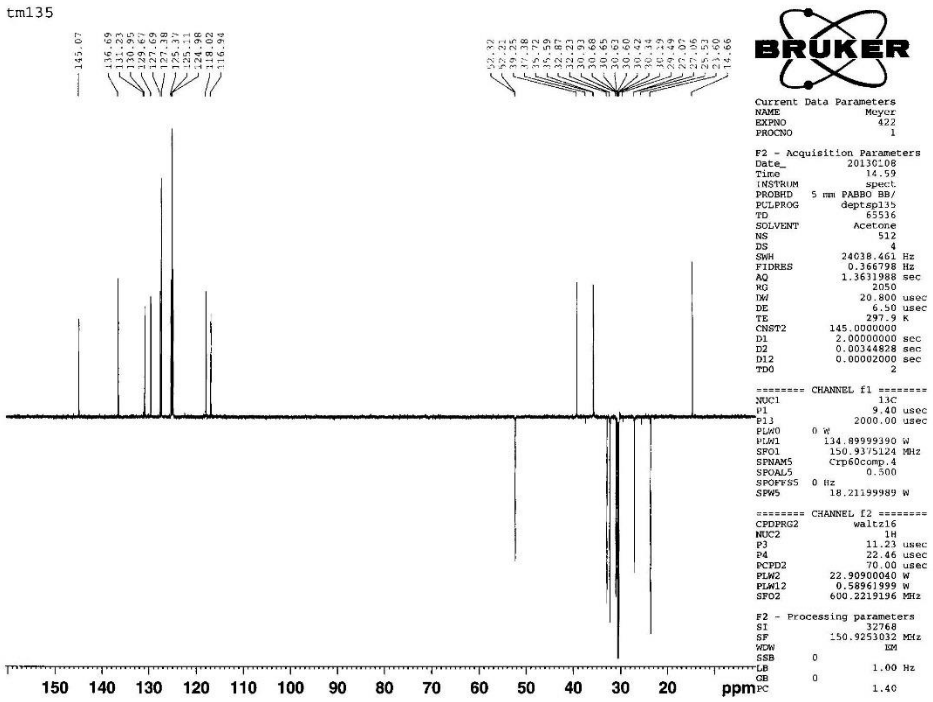

DEPT ^{13}C NMR (150 MHz, acetone-d_6) of compound **12l**.

3.46. (*Z*)-4-{[10-(2-Decyltetradecyl)-7-(1-methyl-1H-pyrazol-4-yl)-10*H*-phenothiazin-3-yl]methylene}-3-methyl-1-phenyl-1H-pyrazol-5[4*H*]-one (12m)

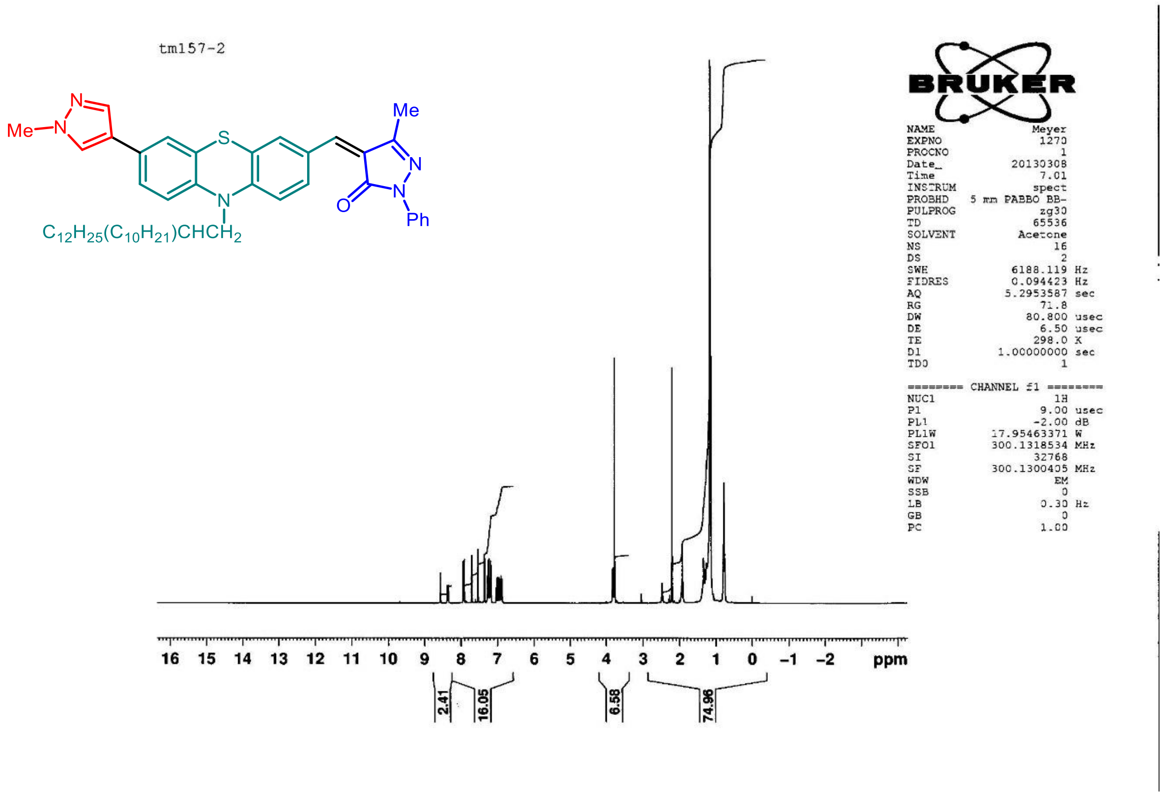

^{1}H NMR (300 MHz, acetone-d_6/CS_2 4:1) of compound **12m**.

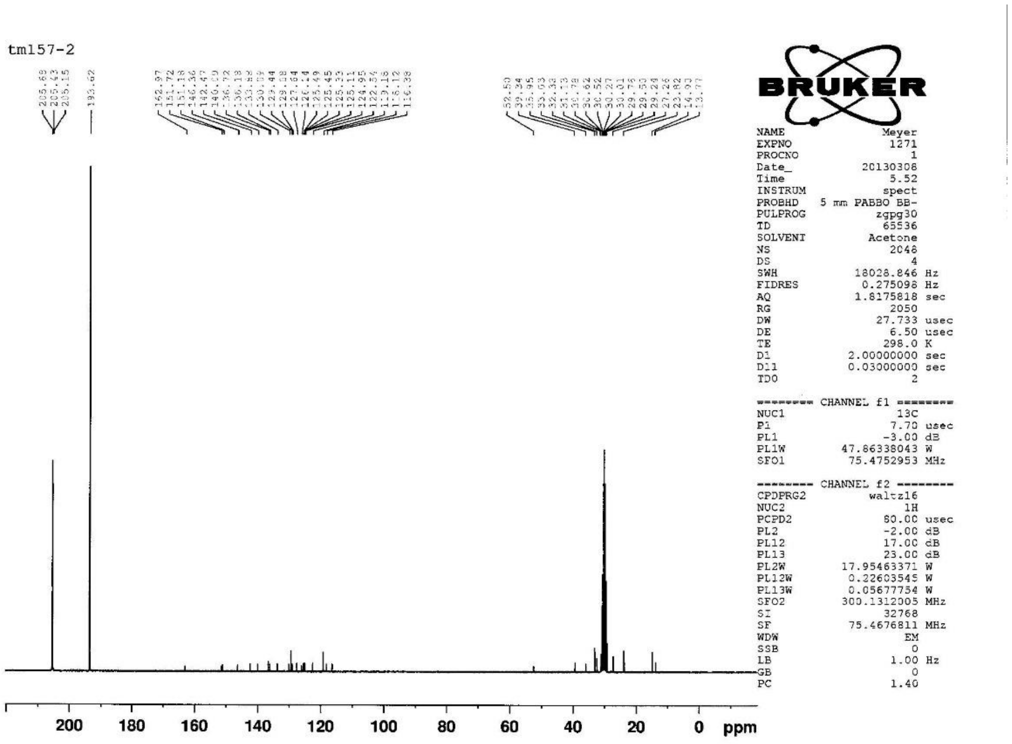

^{13}C NMR (75 MHz, acetone-d_6/CS_2 4:1) of compound **12m**.

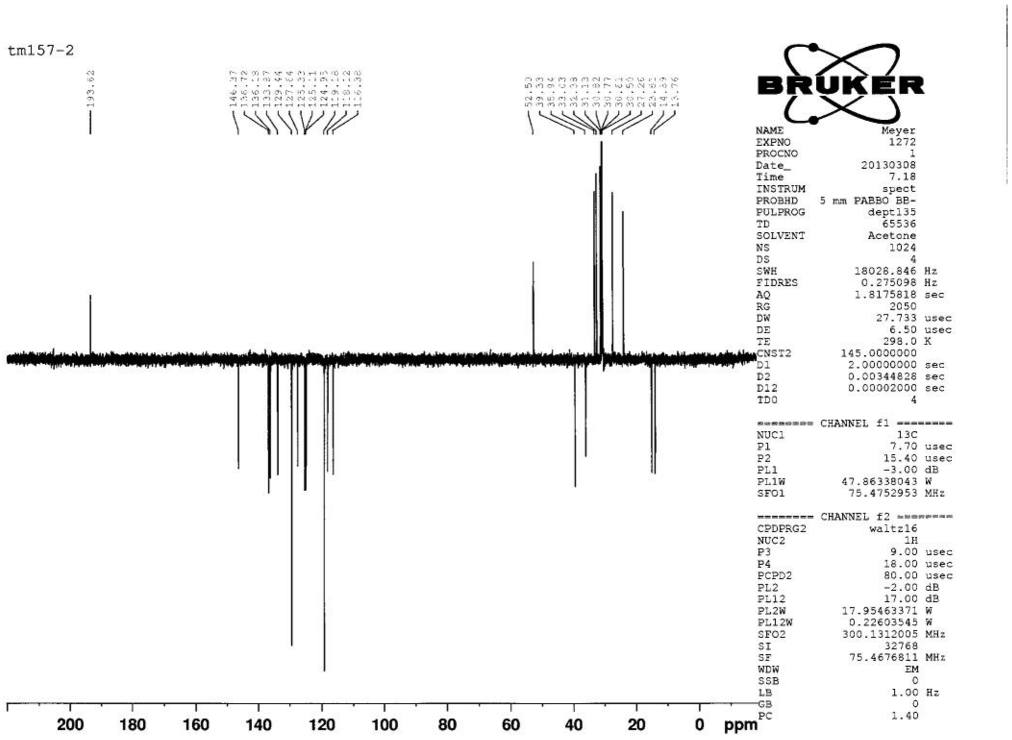

DEPT ^{13}C NMR (75 MHz, acetone-d_6/CS_2 4:1) of compound **12m**.

3.47. (*Z*)-5-{[10-(2-Decyltetradecyl)-7-(5-methylthiophen-2-yl)-10*H*-phenothiazin-3-yl]methylene}-3-methyl-2-thioxothiazolidin-4-one (12n)

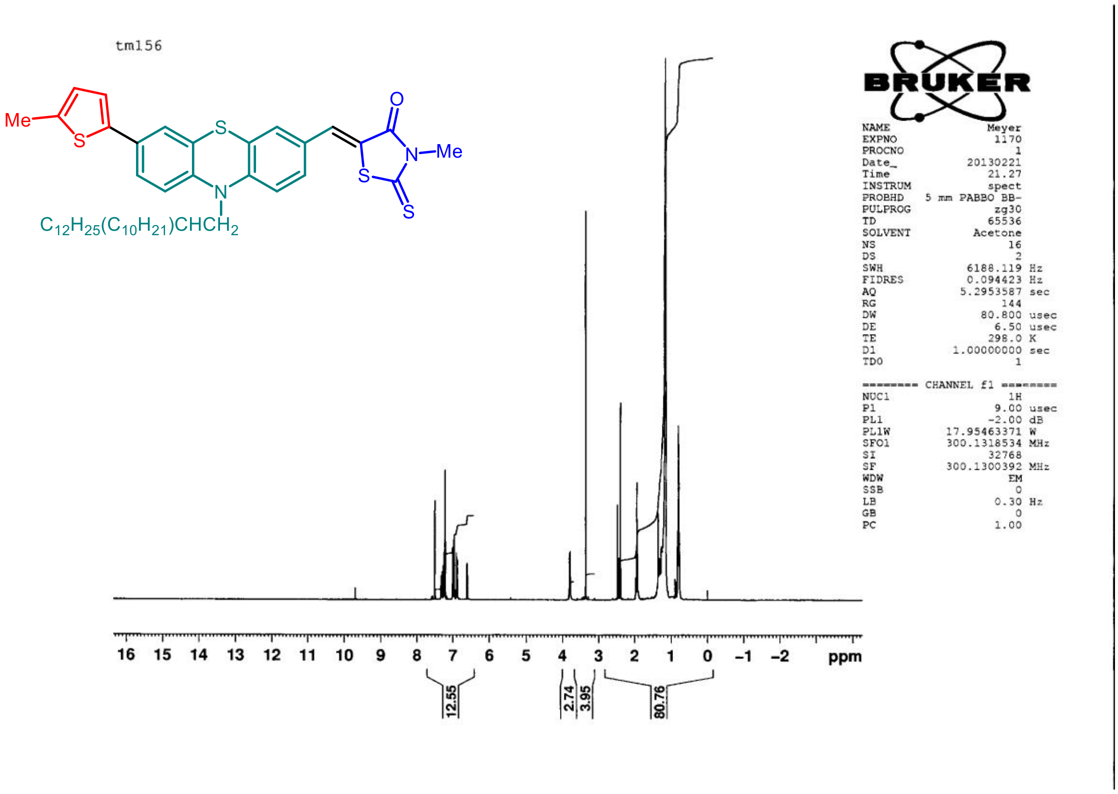

^1H NMR (300 MHz, acetone-d_6/CS_2 4:1) of compound **12n**.

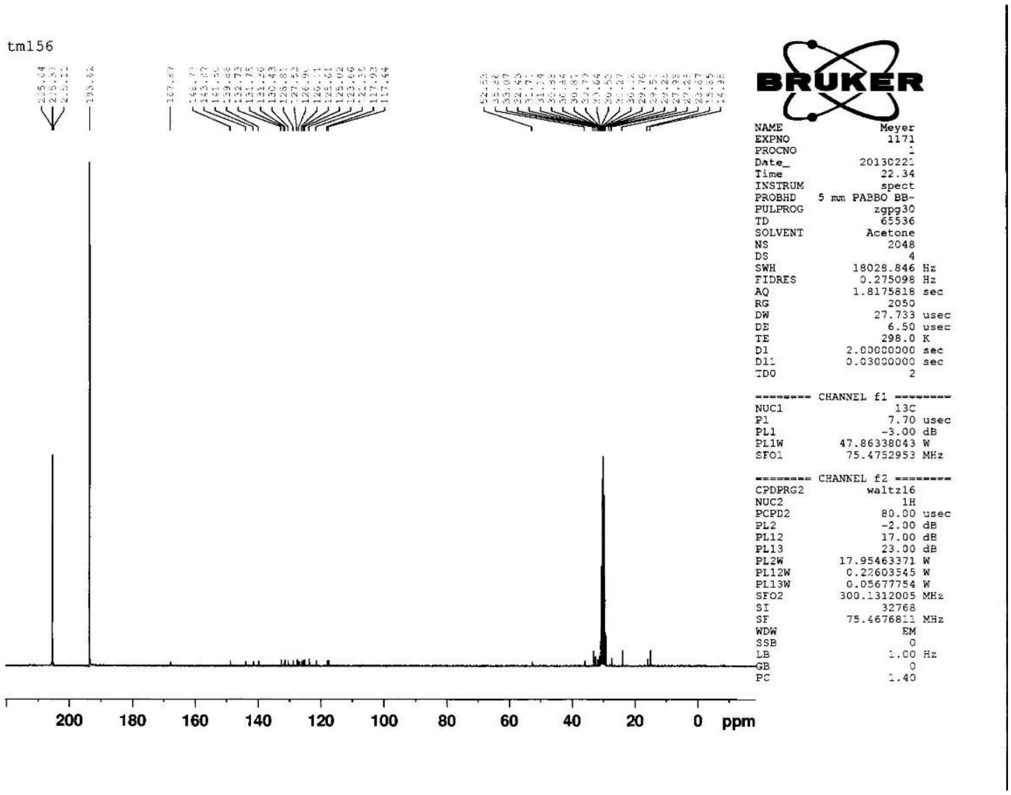

^{13}C NMR (75 MHz, acetone-d_6/CS_2 4:1) of compound **12n**.

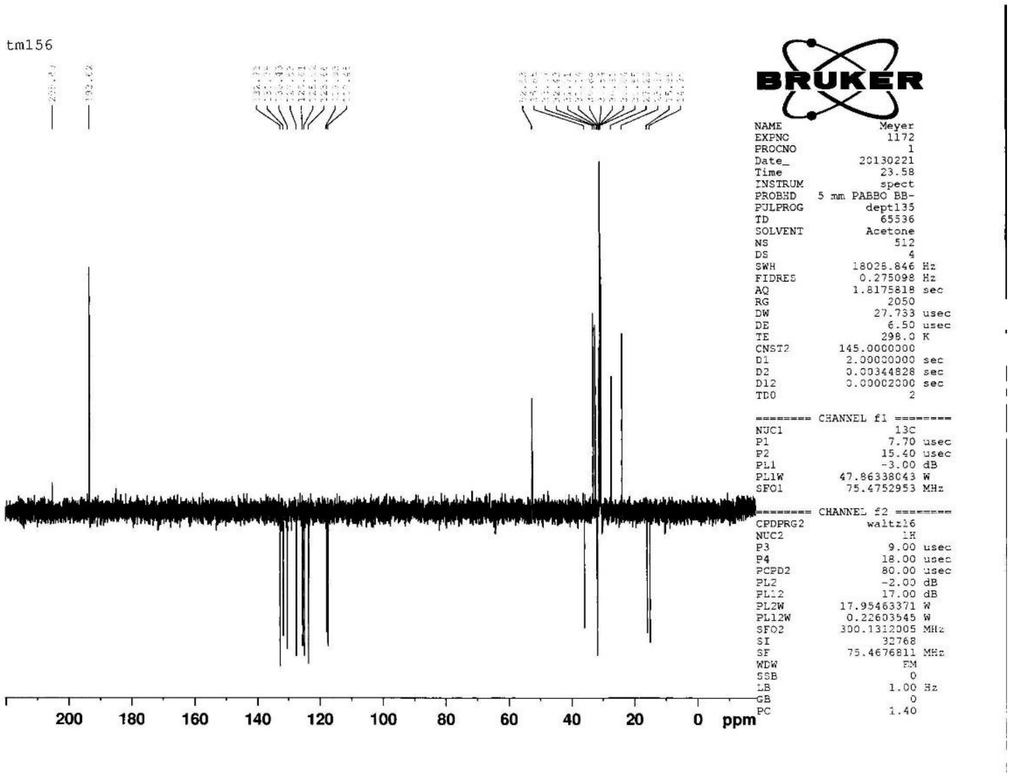

DEPT ^{13}C NMR (75 MHz, acetone-d_6/CS_2 4:1) of compound **12n**.

3.48. (*Z*)-5-{[10-(2-Decyltetradecyl)-7-(5-{4-[diethylamino]phenyl}thiophen-2-yl)-10*H*-phenothiazin-3-yl]methylene}-3-methyl-2-thioxothiazolidin-4-one (12o)

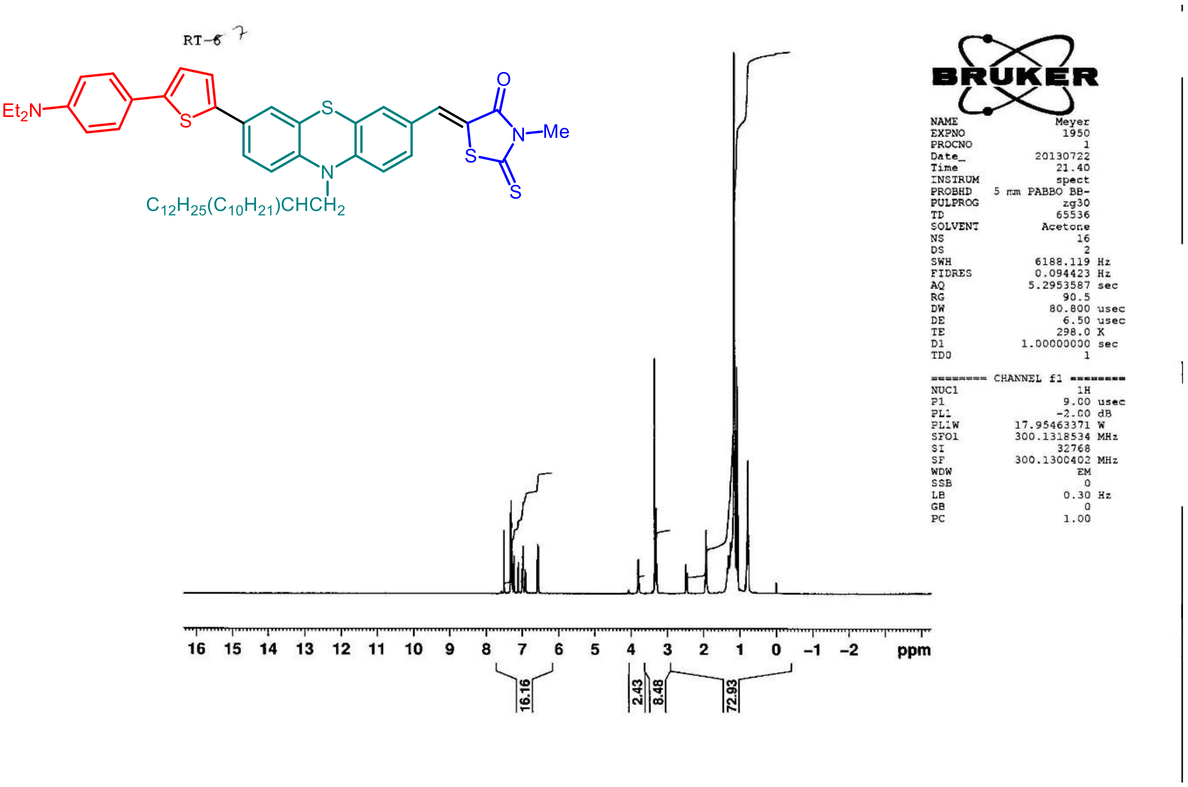

^{1}H NMR (300 MHz, acetone-d$_6$/CS_2 4:1) of compound **12o**.

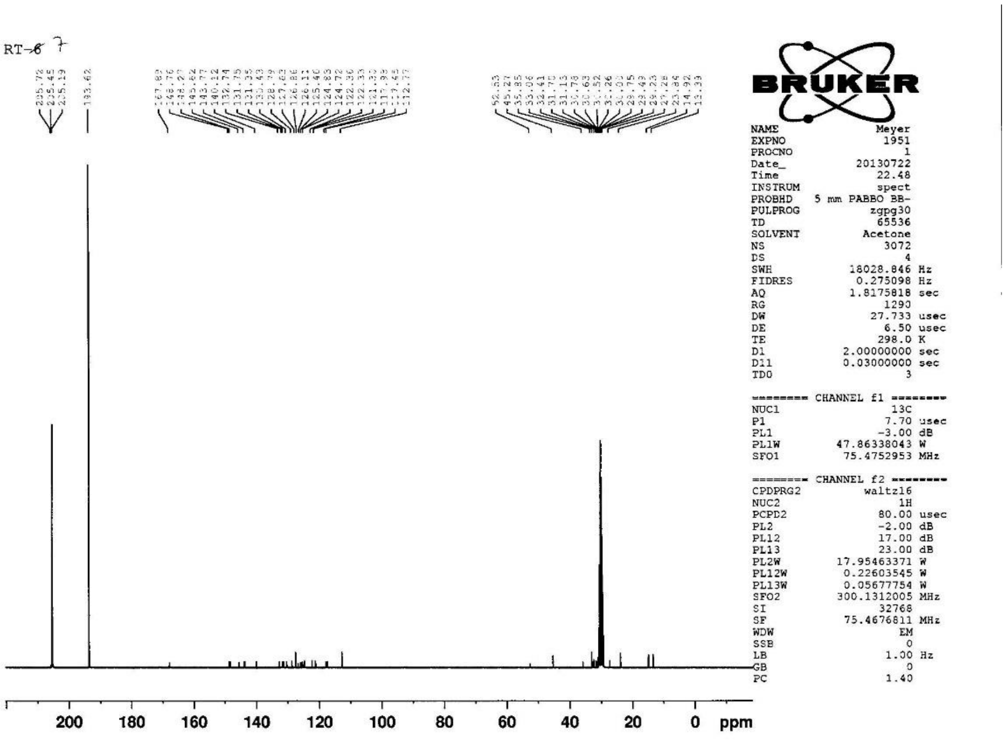

^{13}C NMR (75 MHz, acetone-d$_6$/CS_2 4:1) of compound **12o**.

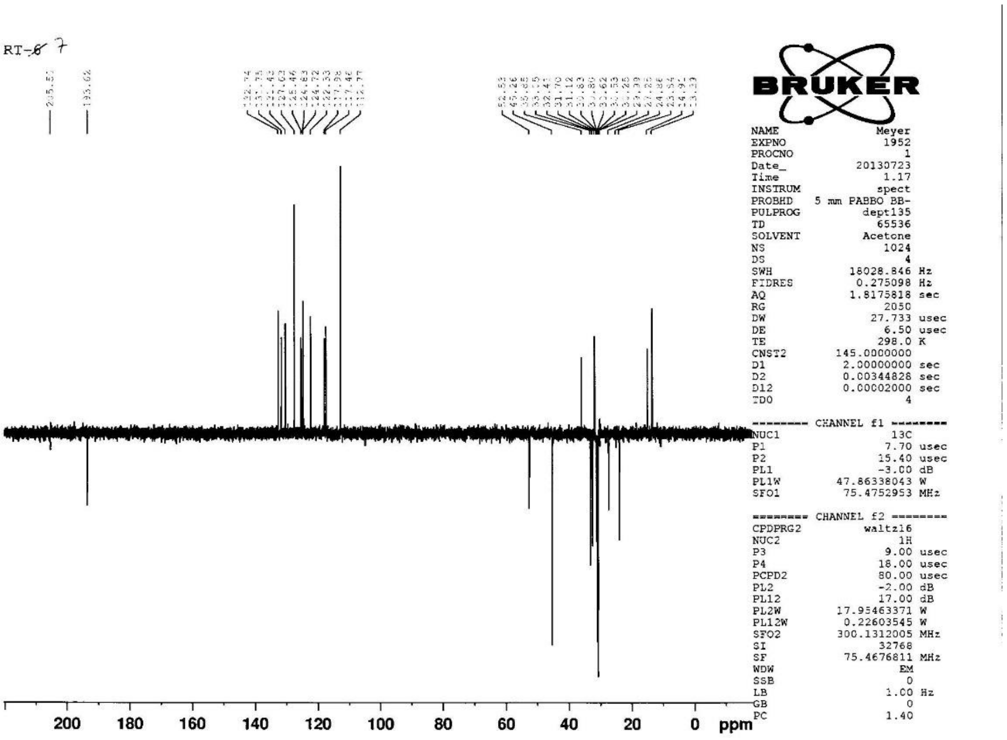

DEPT ^{13}C NMR (75 MHz, acetone-d_6/CS_2 4:1) of compound **12o**.

3.49. (*Z*)-5-{[7-(5-{9*H*-Carbazol-9-yl}thiophen-2-yl)-10-(2-decyltetradecyl)-10*H*-phenothiazin-3-yl]methylene}-3-methyl-2-thioxothiazolidin-4-one (12p)

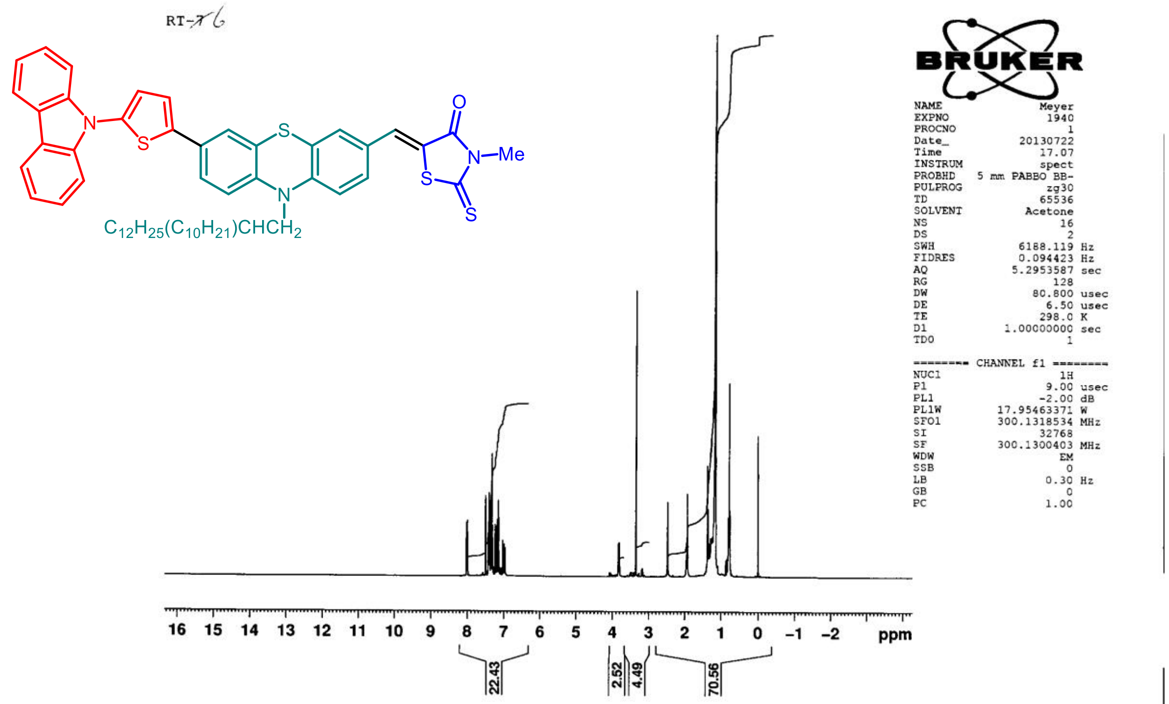

^{1}H NMR (300 MHz, acetone-d_6/CS_2 4:1) of compound **12p**.

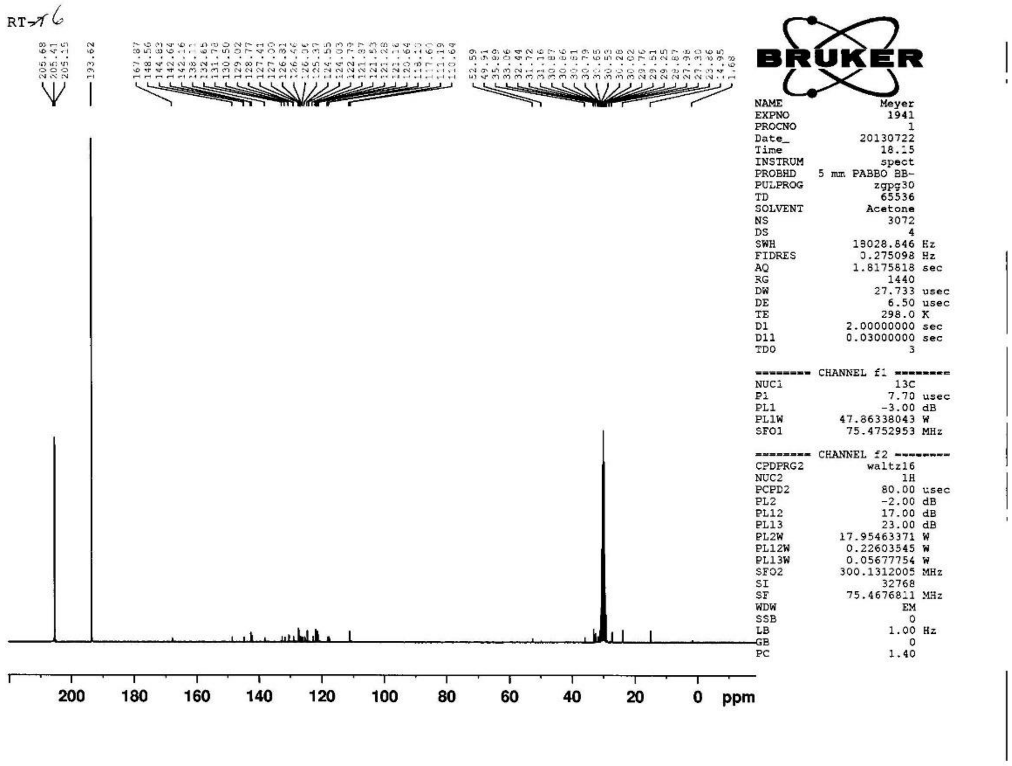

^{13}C NMR (75 MHz, acetone-d_6/CS_2 4:1) of compound **12p**.

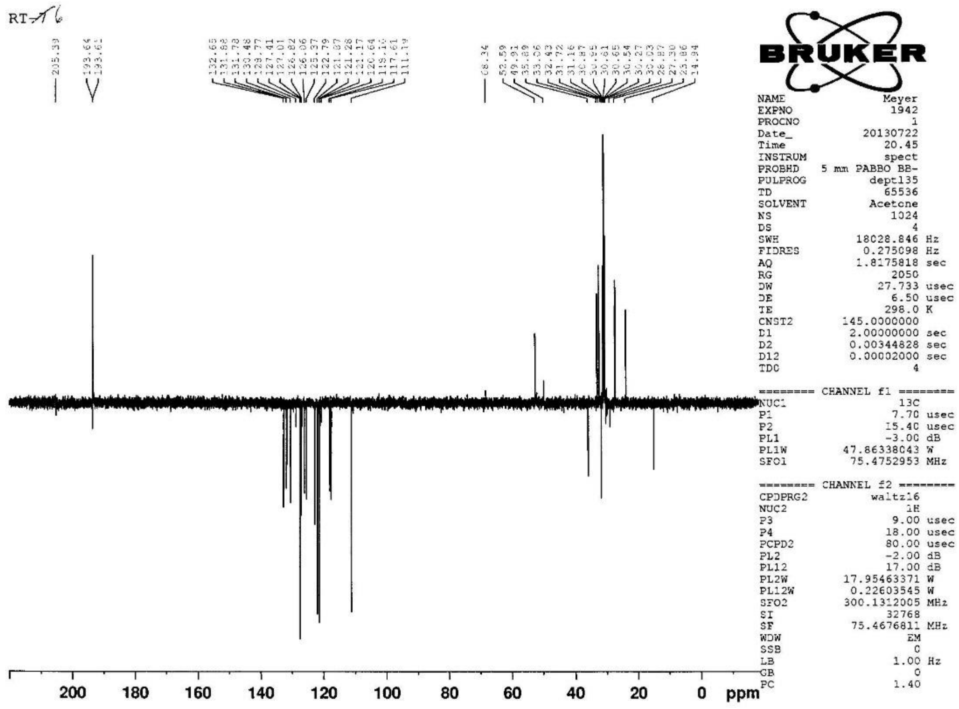

DEPT ^{13}C NMR (75 MHz, acetone-d_6/CS_2 4:1) of compound **12p**.

3.50. (*Z*)-5-{[10-(2-Decyltetradecyl)-7-(4-{diphenylamino}phenyl)-10*H*-phenothiazin-3-yl]methylene}-3-methyl-2-thioxothiazolidin-4-one (12q)

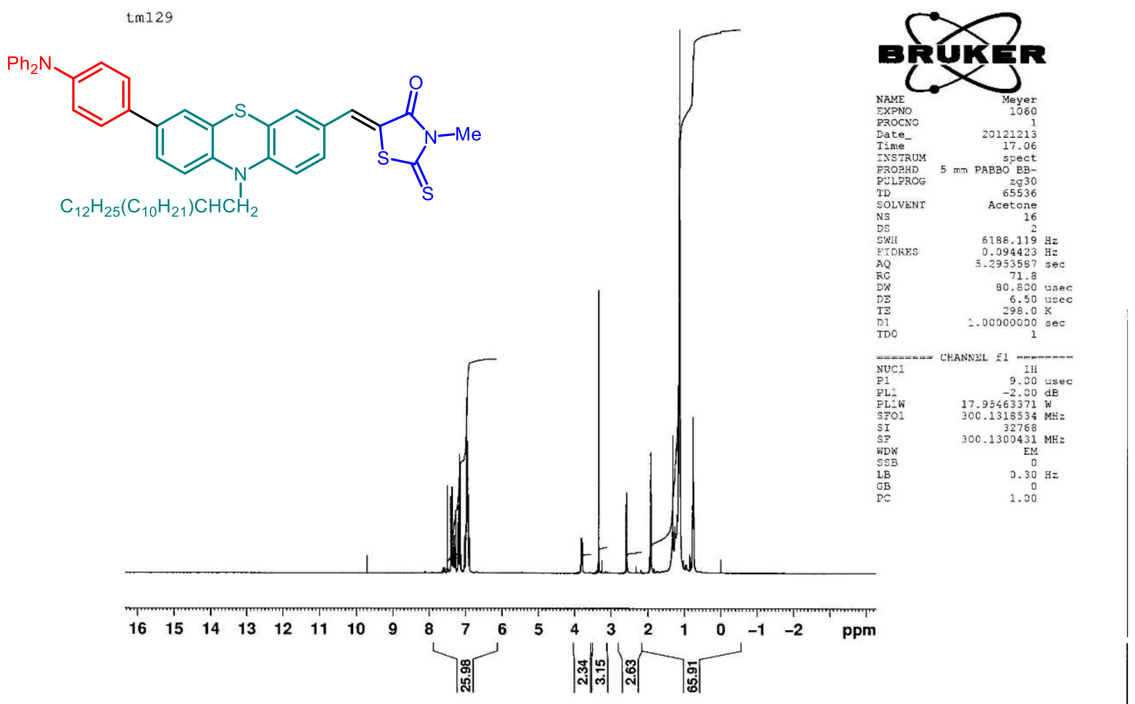

^{1}H NMR (300 MHz, acetone-d$_6$/CS_2 4:1) of compound **12q**.

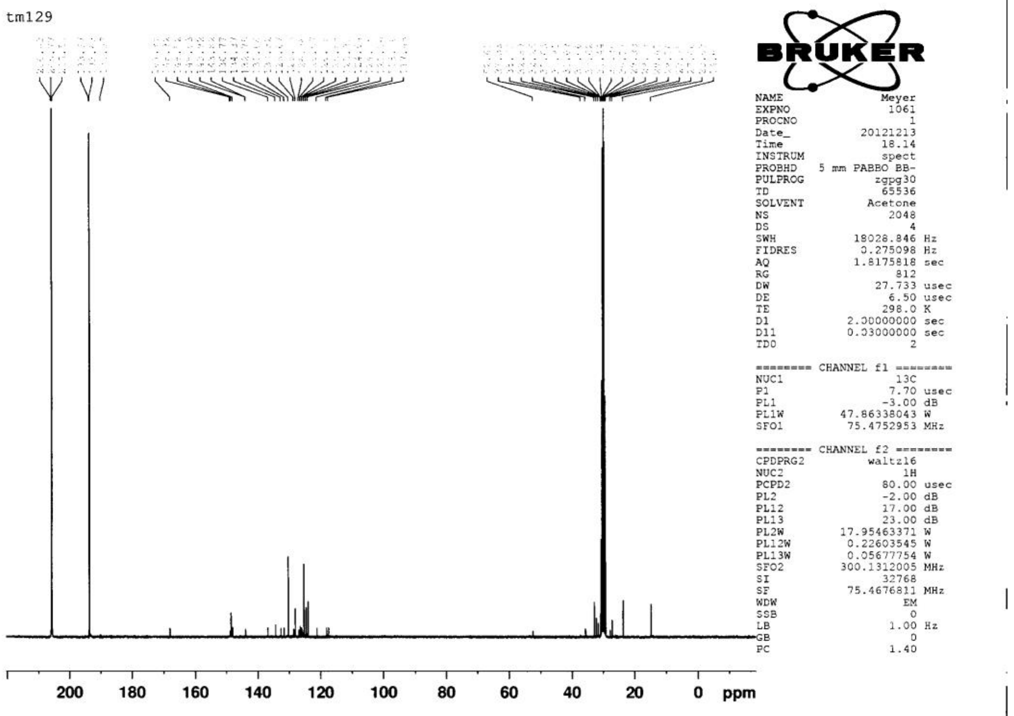

^{13}C NMR (75 MHz, acetone-d$_6$/CS_2 4:1) of compound **12q**.

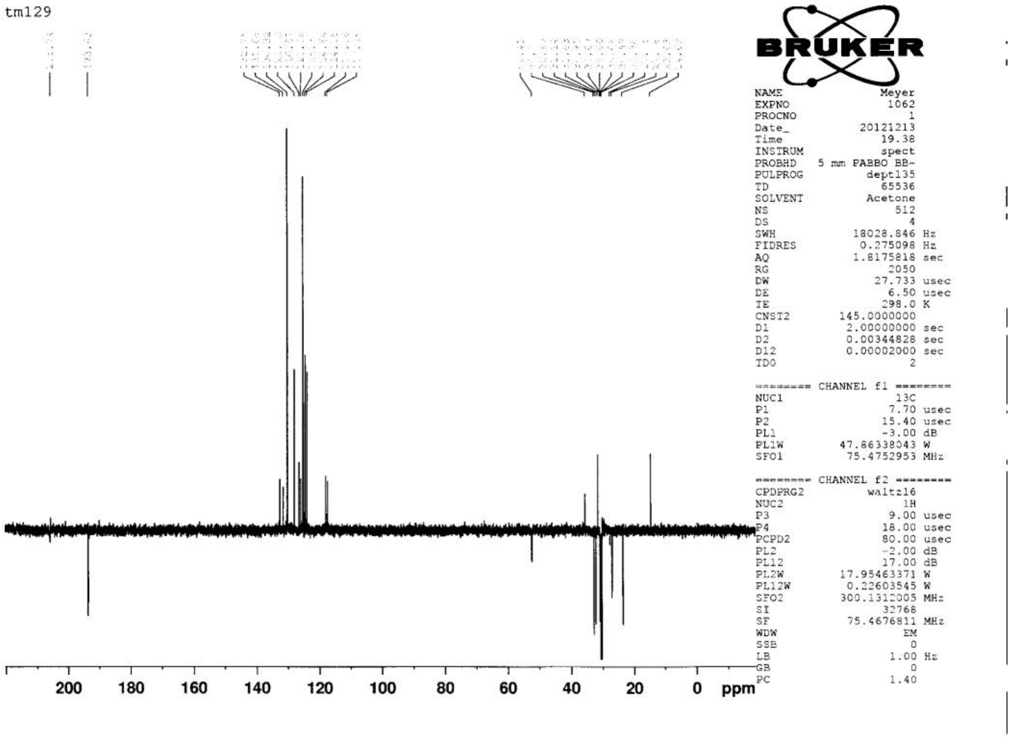

DEPT ^{13}C NMR (75 MHz, acetone-d_6/CS_2 4:1) of compound **12q**.

3.51. (*Z*)-3-{10-(2-Decyltetradecyl)-7-[4-(diphenylamino)phenyl]-10*H*-phenothiazin-3-yl}-2-(4-nitrophenyl)acrylonitrile (12r)

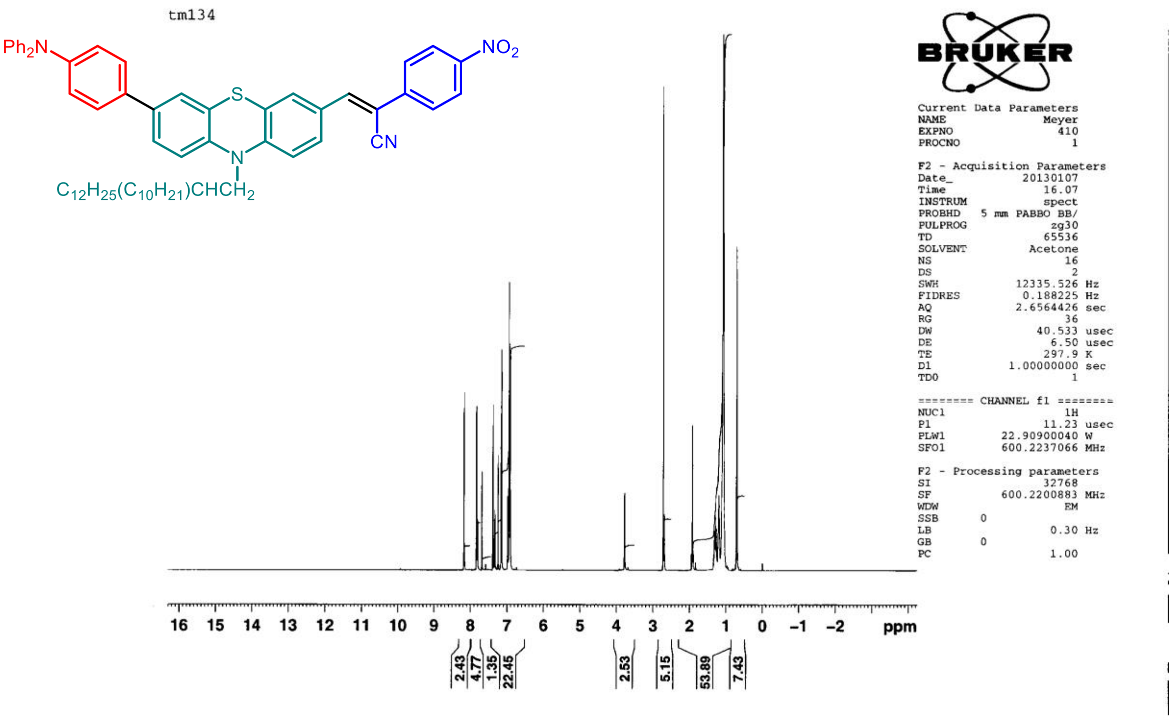

^{1}H NMR (600 MHz, acetone-d$_6$) of compound **12r**.

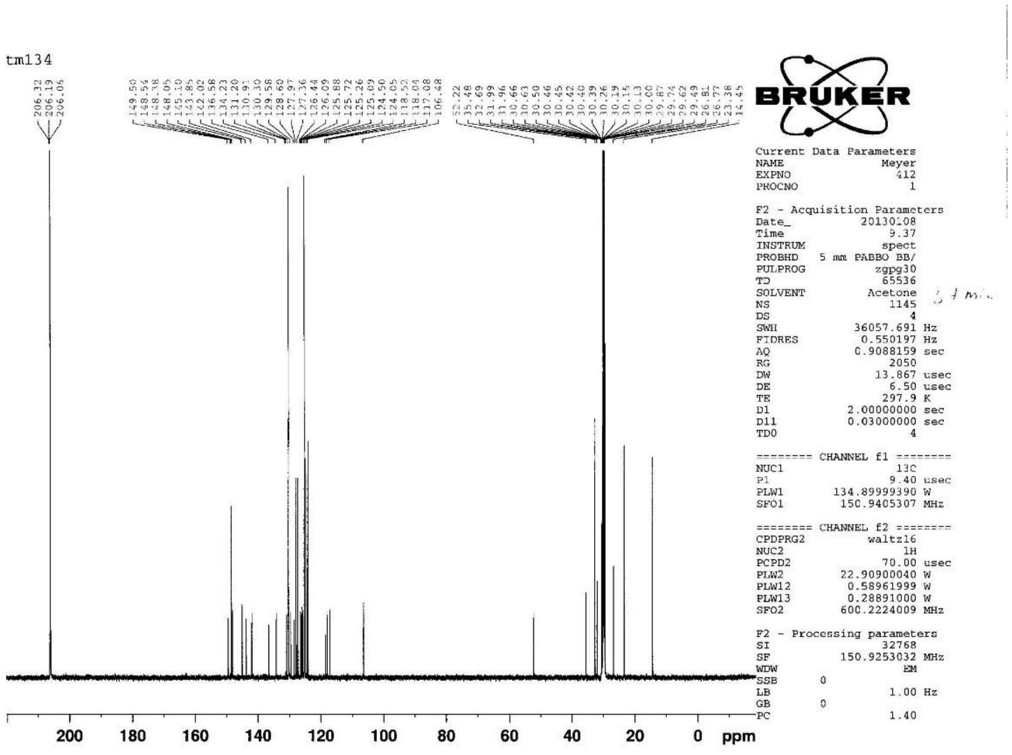

^{13}C NMR (150 MHz, acetone-d$_6$) of compound **12r**.

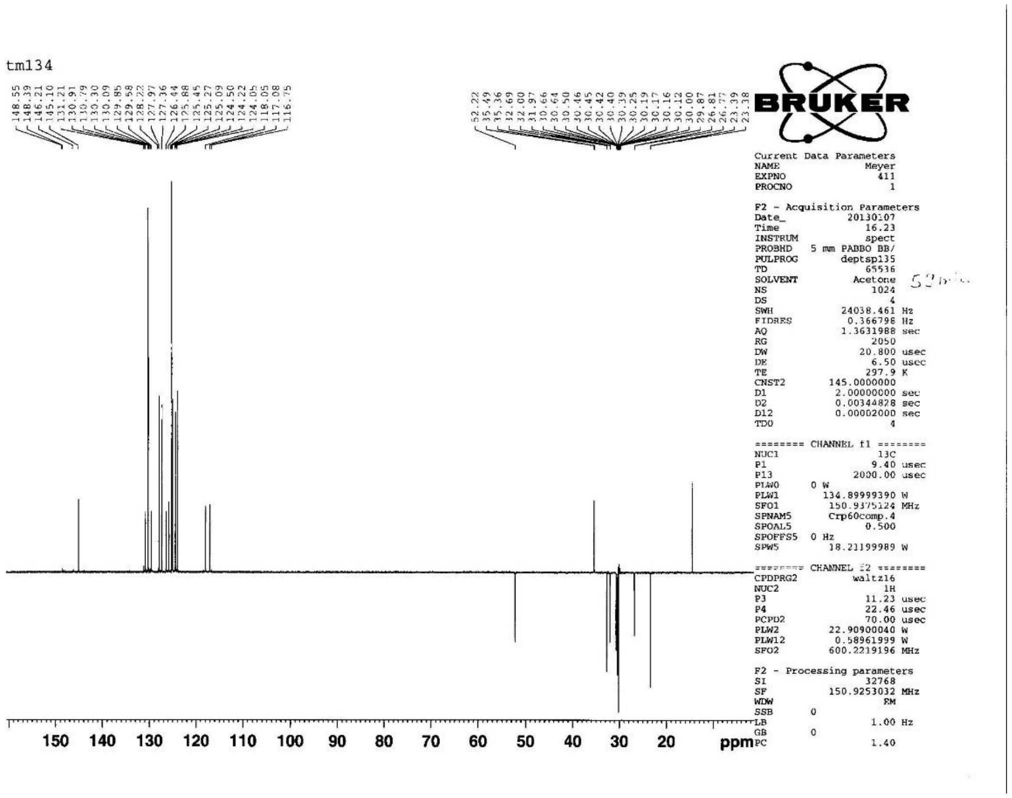

DEPT ^{13}C NMR (150 MHz, acetone-d_6) of compound **12r**.

3.52. (*Z*)-5-{[7-(4-{Bis[4-methoxyphenyl]amino}phenyl)-10-(2-decyltetradecyl)-10*H*-phenothiazin-3-yl]methylene}-3-methyl-2-thioxothiazolidin-4-one (12s)

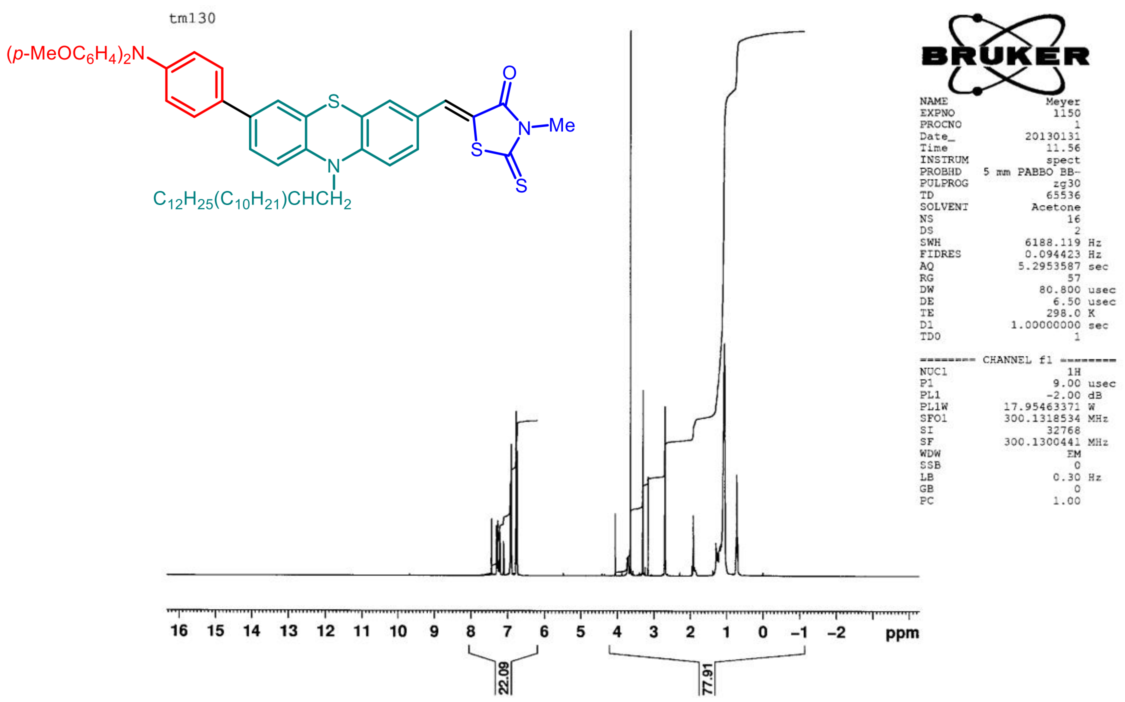

^{1}H NMR (300 MHz, acetone-d_6) of compound **12s**.

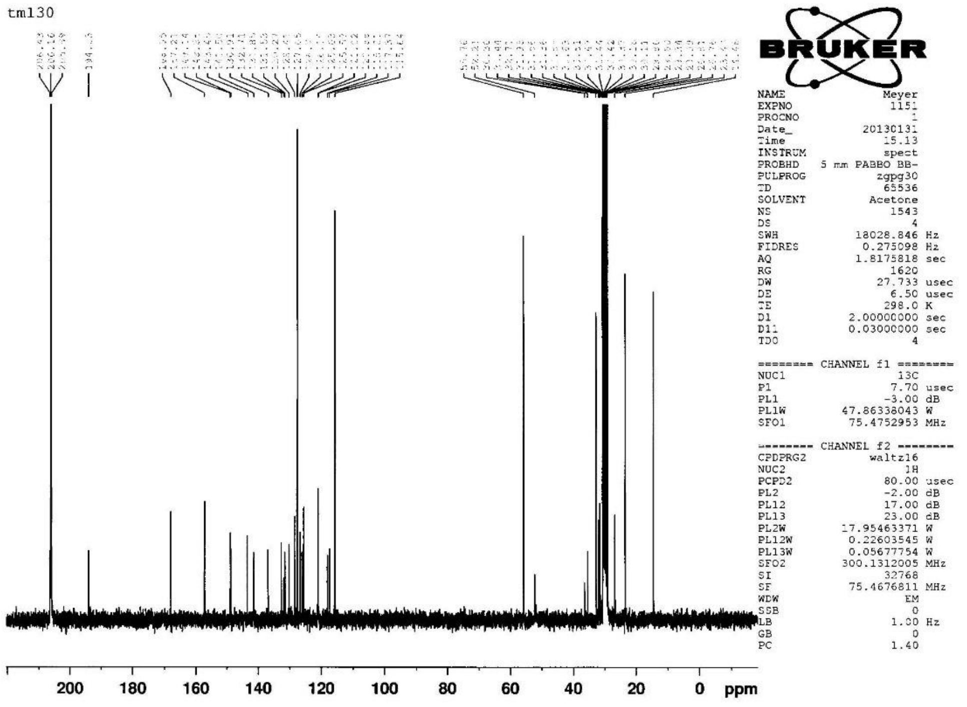

^{13}C NMR (75 MHz, acetone-d_6) of compound **12s**.

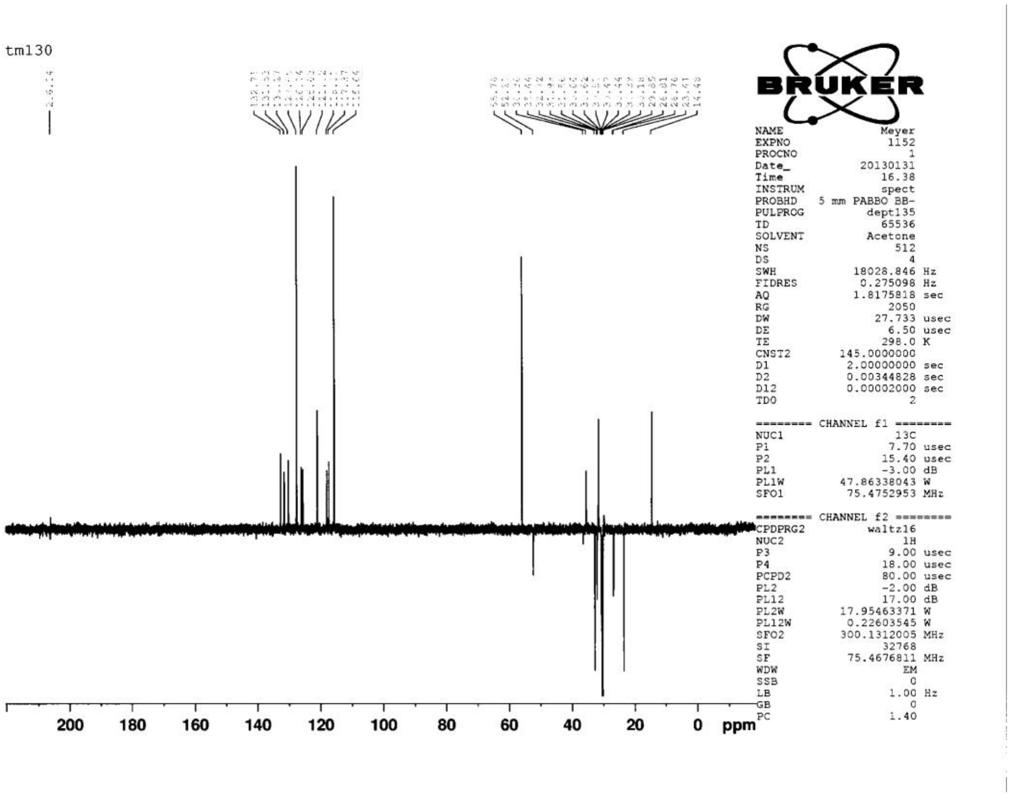

DEPT ^{13}C NMR (75 MHz, acetone-d_6) of compound **12s**.

3.53. (*Z*)-5-{[7-(4-{Bis[4-methoxyphenyl]amino}phenyl)-10-(2-decyltetradecyl)-10*H*-phenothiazin-3-yl]methylene}-2-thioxothiazolidin-4-one (12t)

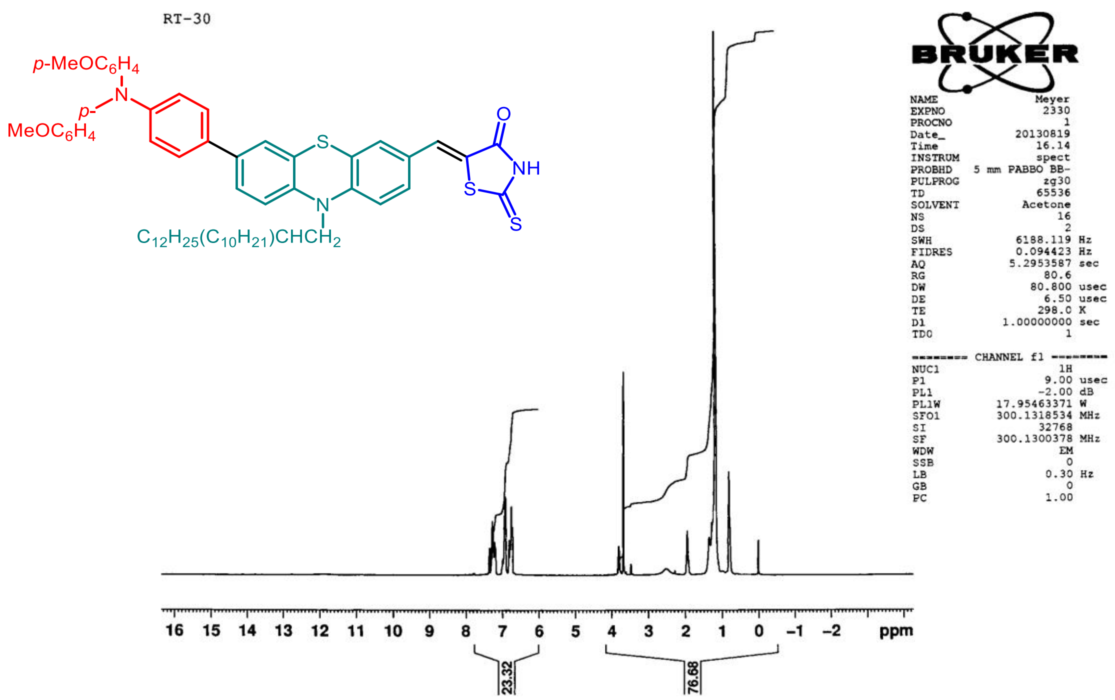

^{1}H NMR (300 MHz, acetone-d_6/CS_2 4:1) of compound **12t**.

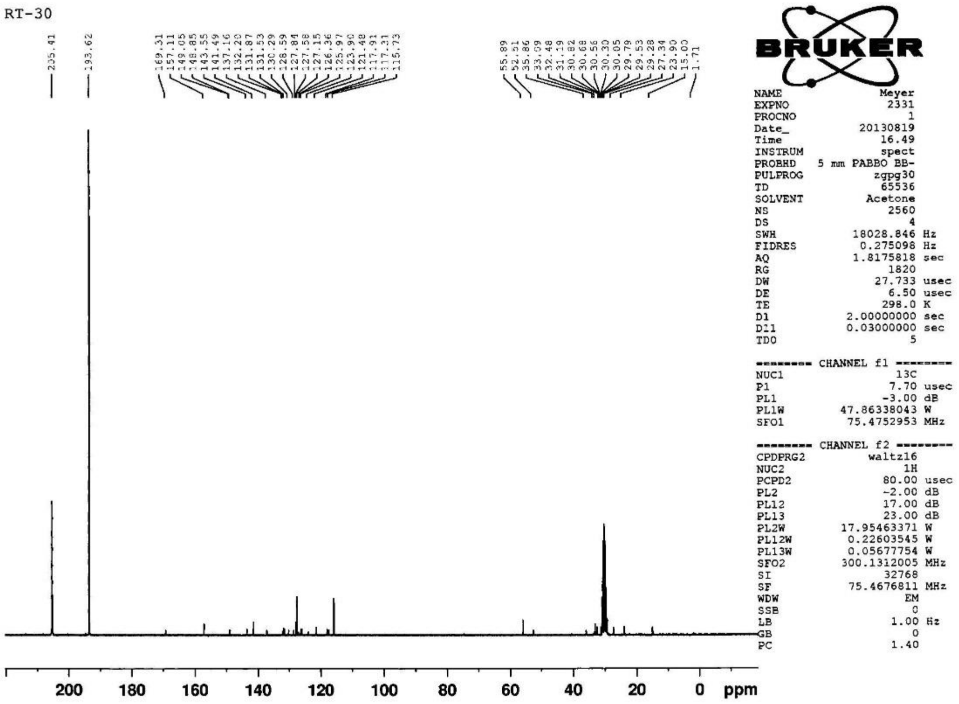

^{13}C NMR (75 MHz, acetone-d_6/CS_2 4:1) of compound **12t**.

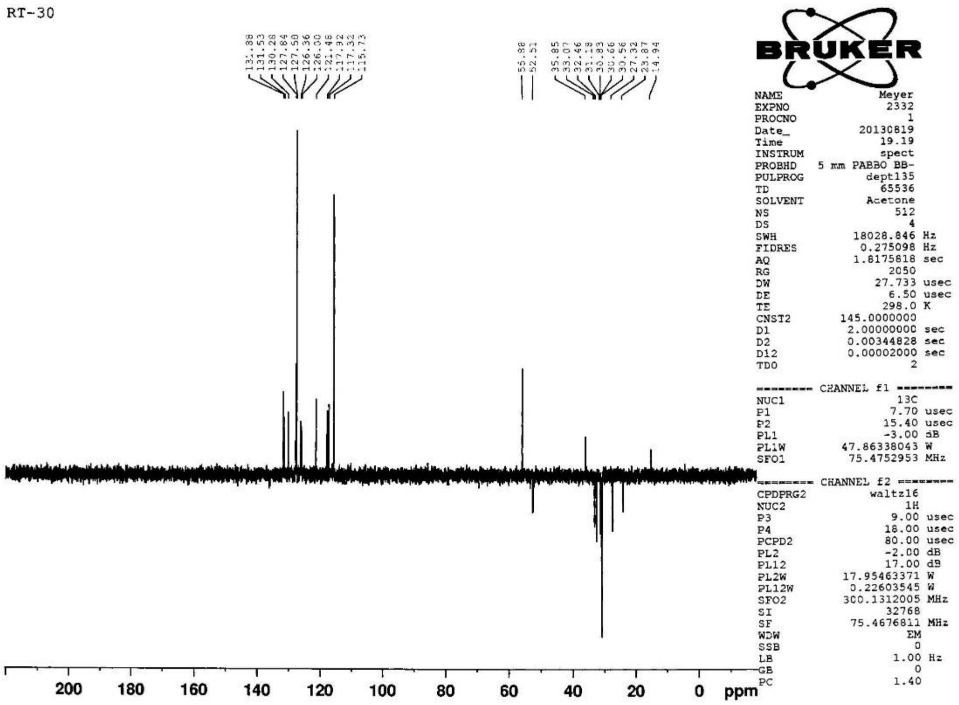

DEPT ^{13}C NMR (75 MHz, acetone-d_6/CS_2 4:1) of compound **12t**.

3.54. (*Z*)-3-{7-[4-(Bis{4-methoxyphenyl}amino)phenyl]-10-(2-decyltetradecyl)-10*H*-phenothiazin-3-yl}-2-(4-nitrophenyl)acrylonitrile (12u)

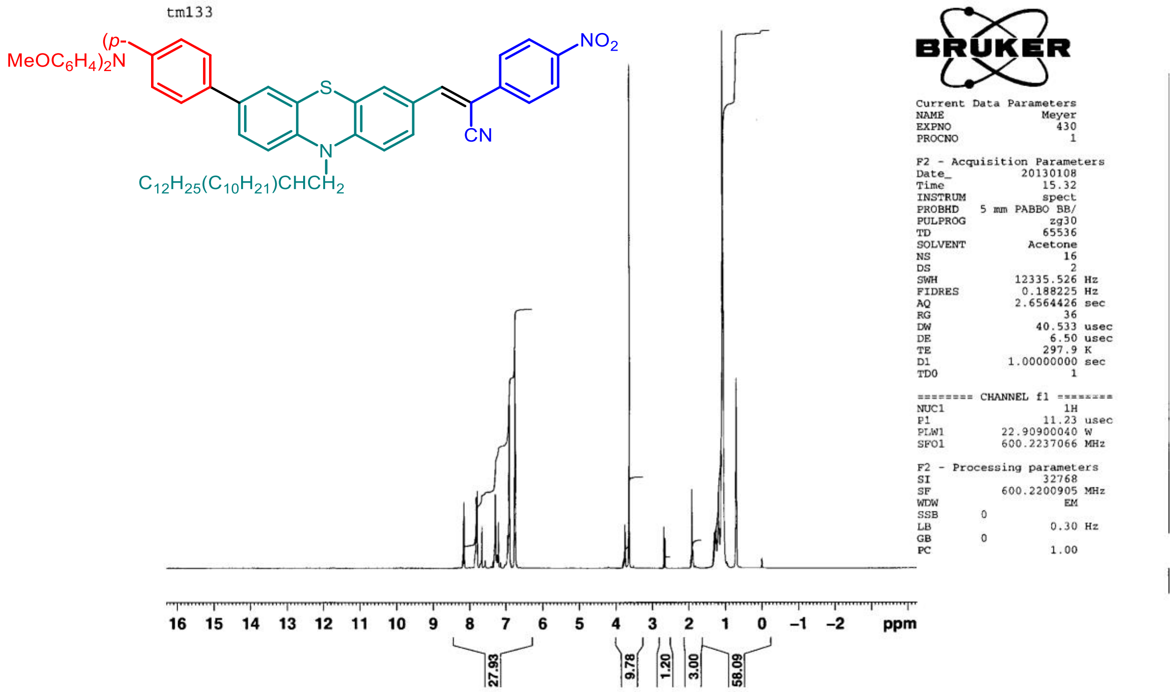

^{1}H NMR (600 MHz, acetone-d6) of compound **12u**.

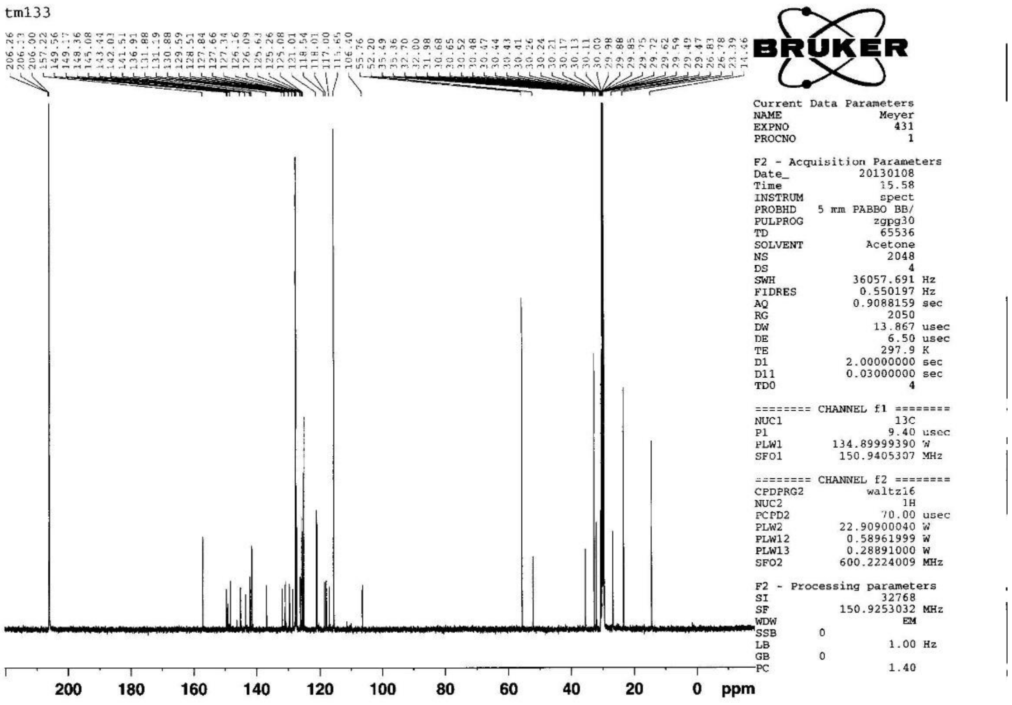

^{13}C NMR (150 MHz, acetone-d6) of compound **12u**.

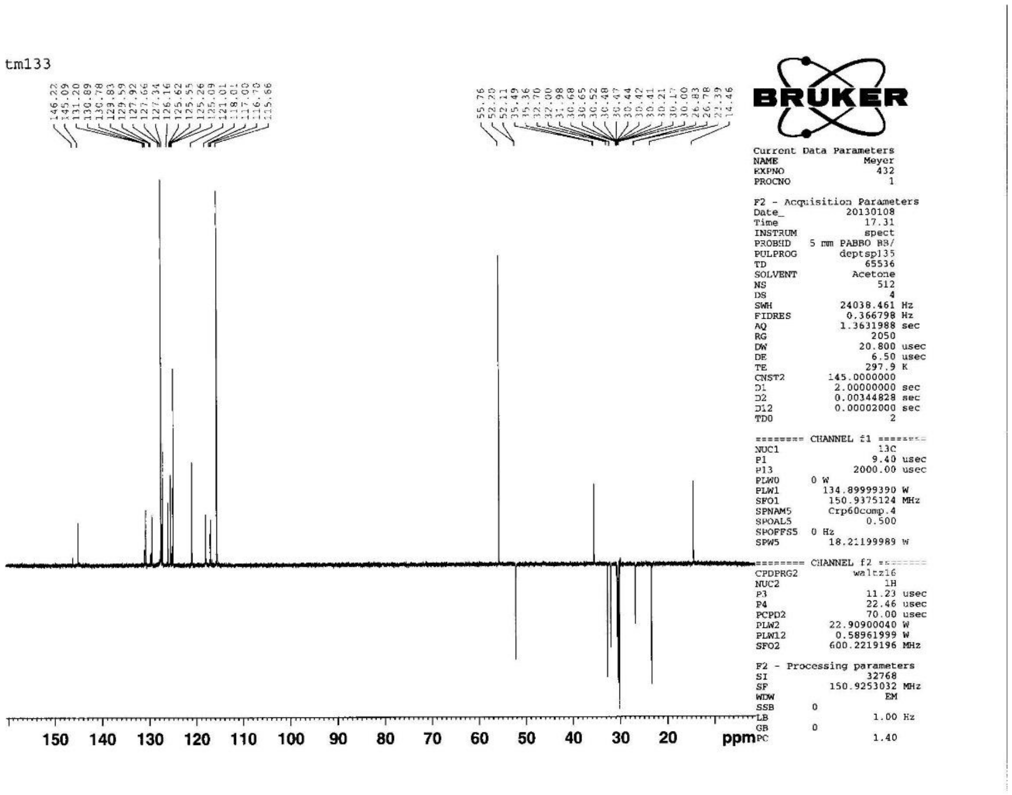

DEPT ^{13}C NMR (150 MHz, acetone-d_6) of compound **12u**.

3.55. (*Z*)-4-{[7-(4-(Bis{4-methoxyphenyl}amino)phenyl]-10-(2-decyltetradecyl)-10*H*-phenothiazin-3-yl]methylene}-3-methyl-1-phenyl-1H-pyrazol-5[4*H*]-one (12v)

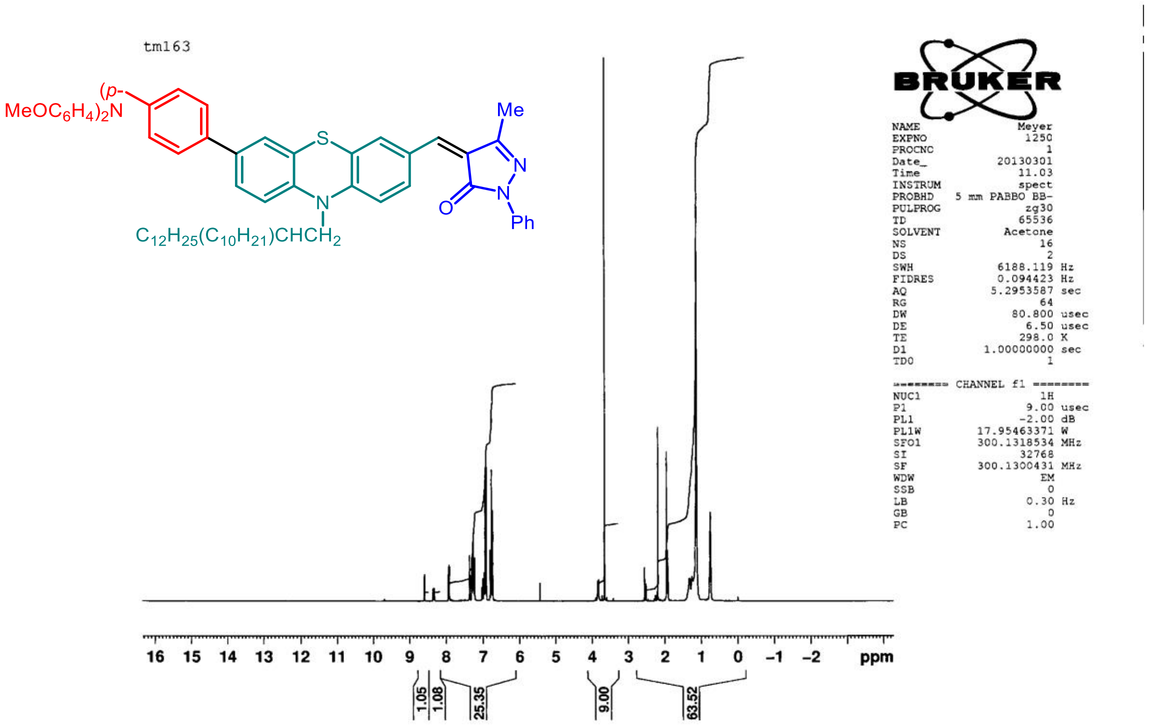

^{1}H NMR (300 MHz, acetone-d_6/CS_2 4:1) of compound **12v**.

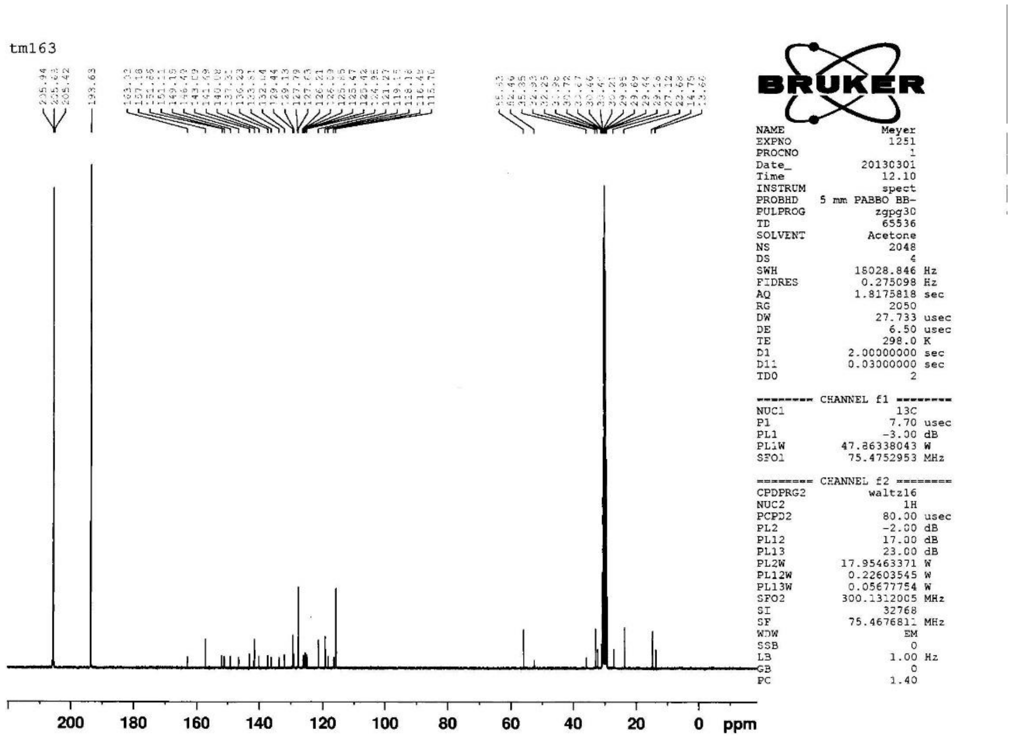

^{13}C NMR (75 MHz, acetone-d_6/CS_2 4:1) of compound **12v**.

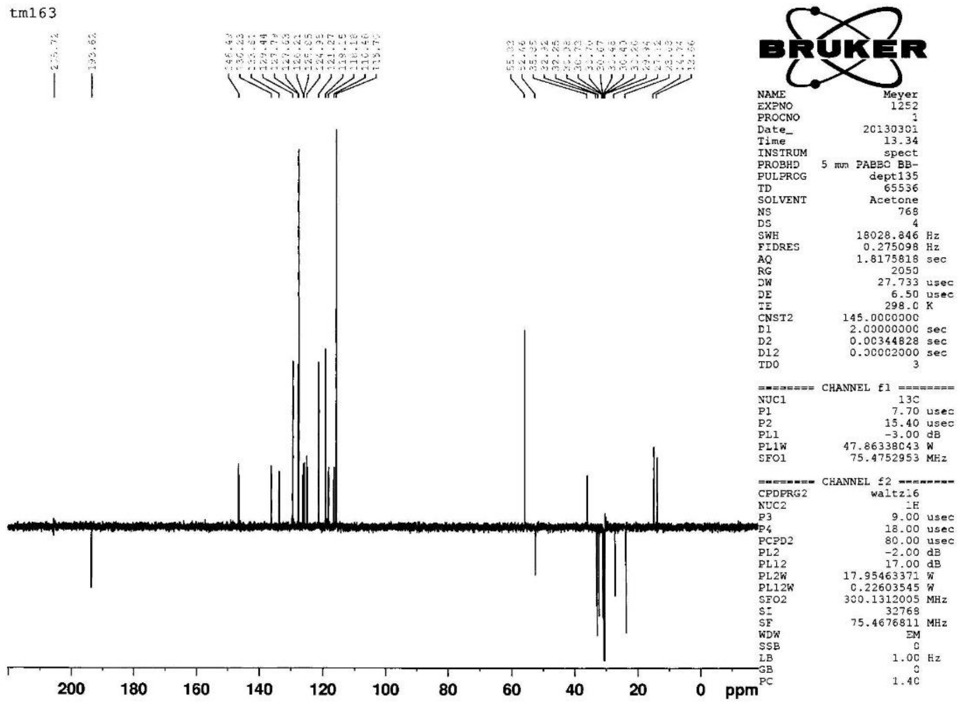

DEPT ^{13}C NMR (75 MHz, acetone-d_6/CS_2 4:1) of compound **12v**.

3.56. (*Z*)-5-{[10-(2-Decyltetradecyl)-10'-hexyl-7'-(*p*-tolyl)-10*H*,10'*H*-(3,3'-biphenothiazin)-7-yl]methylene}-3-methyl-2-thioxothiazolidin-4-one (12w)

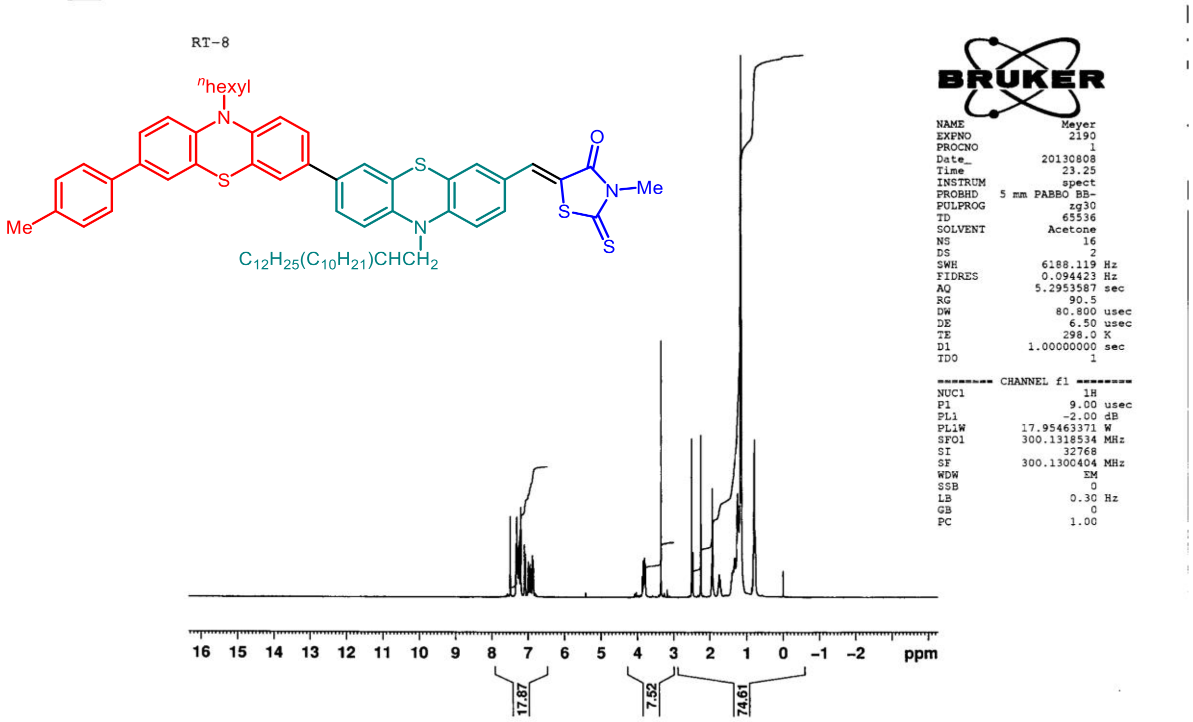

^{1}H NMR (300 MHz, acetone-d$_6$/CS$_2$ 4:1) of compound **12w**.

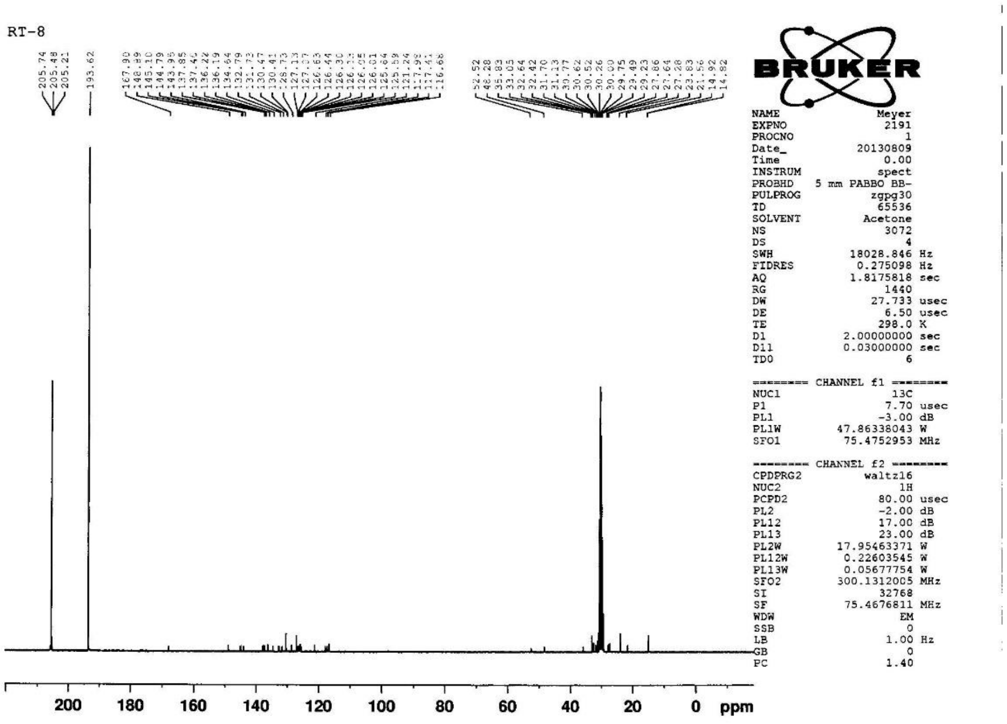

^{13}C NMR (75 MHz, acetone-d$_6$/CS$_2$ 4:1) of compound **12w**.

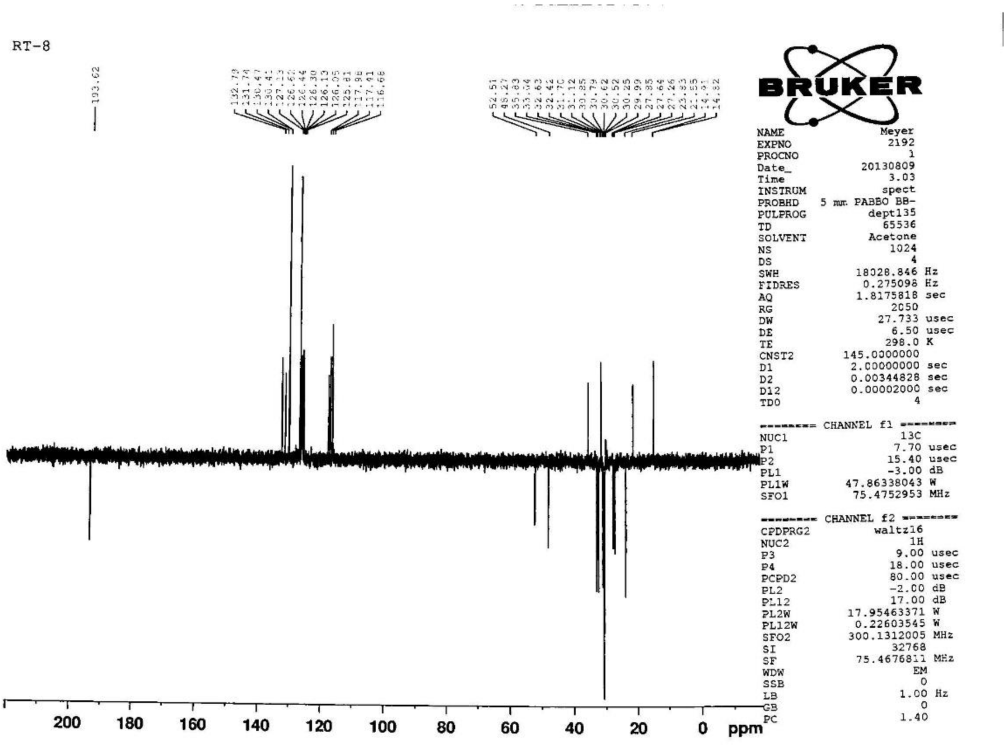

DEPT ^{13}C NMR (75 MHz, acetone-d_6/CS_2 4:1) of compound **12w**.

3.57. (*Z*)-5-{[10-(2-Decyltetradecyl)-10'-hexyl-7'-(*p*-tolyl)-10*H*,10'*H*-(3,3'-biphenothiazin)-7-yl]methylene}-2-thioxothiazolidin-4-one (12x)

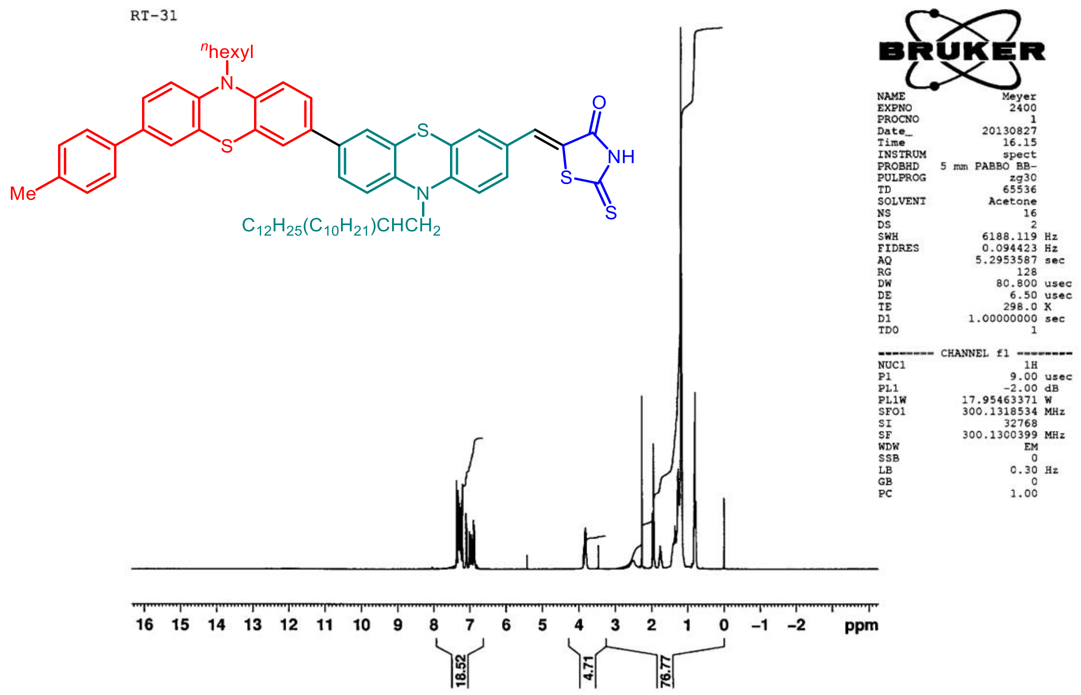

^{1}H NMR (300 MHz, acetone-d$_6$/CS$_2$ 4:1) of compound **12x**.

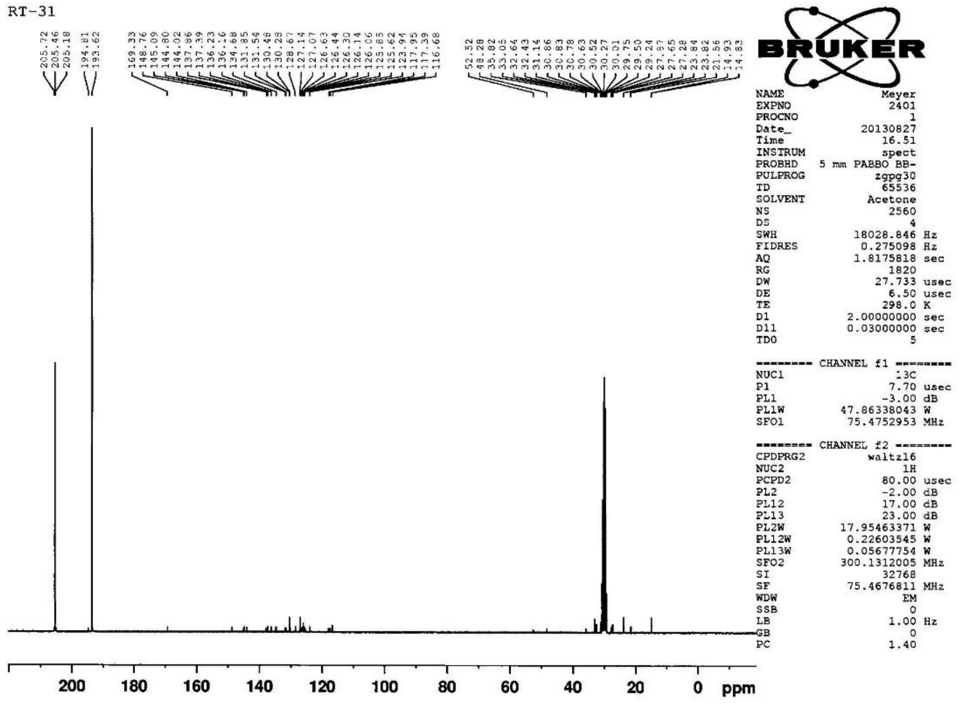

^{13}C NMR (75 MHz, acetone-d$_6$/CS$_2$ 4:1) of compound **12x**.

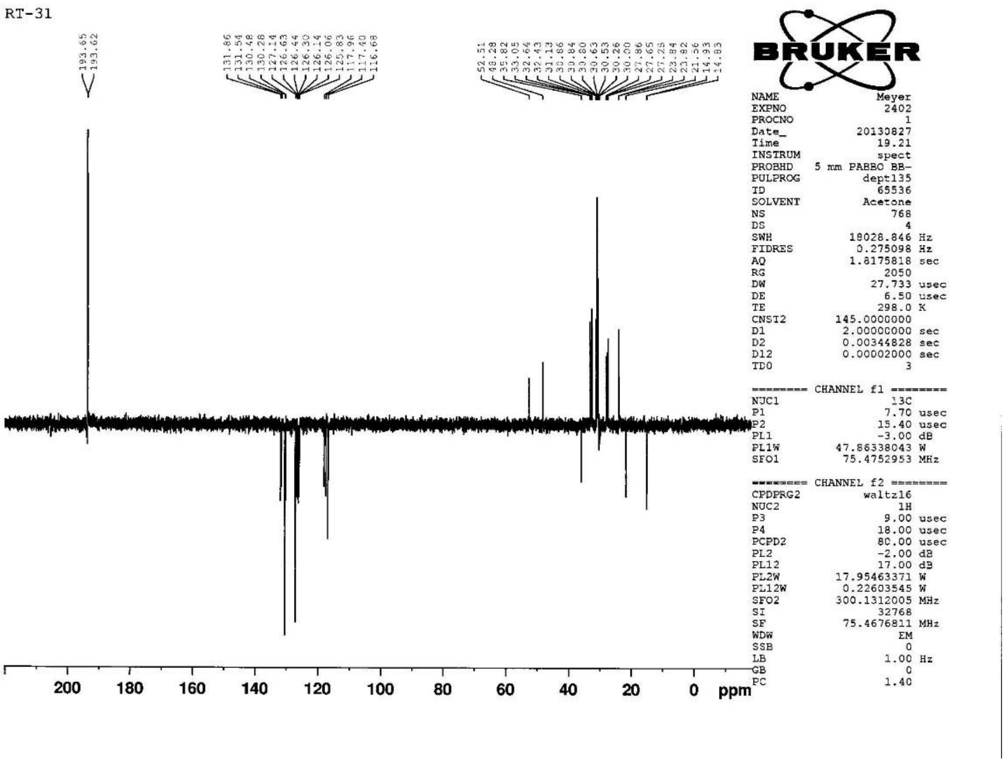

DEPT ^{13}C NMR (75 MHz, acetone-d_6/CS_2 4:1) of compound **12x**.

3.58. (*Z*)-3-[10-(2-Decyltetradecyl)-10'-hexyl-7'-(*p*-tolyl)-10*H*,10'*H*-(3,3'-biphenothiazin)-7-yl]-2-(4-nitrophenyl)acrylonitrile (12y)

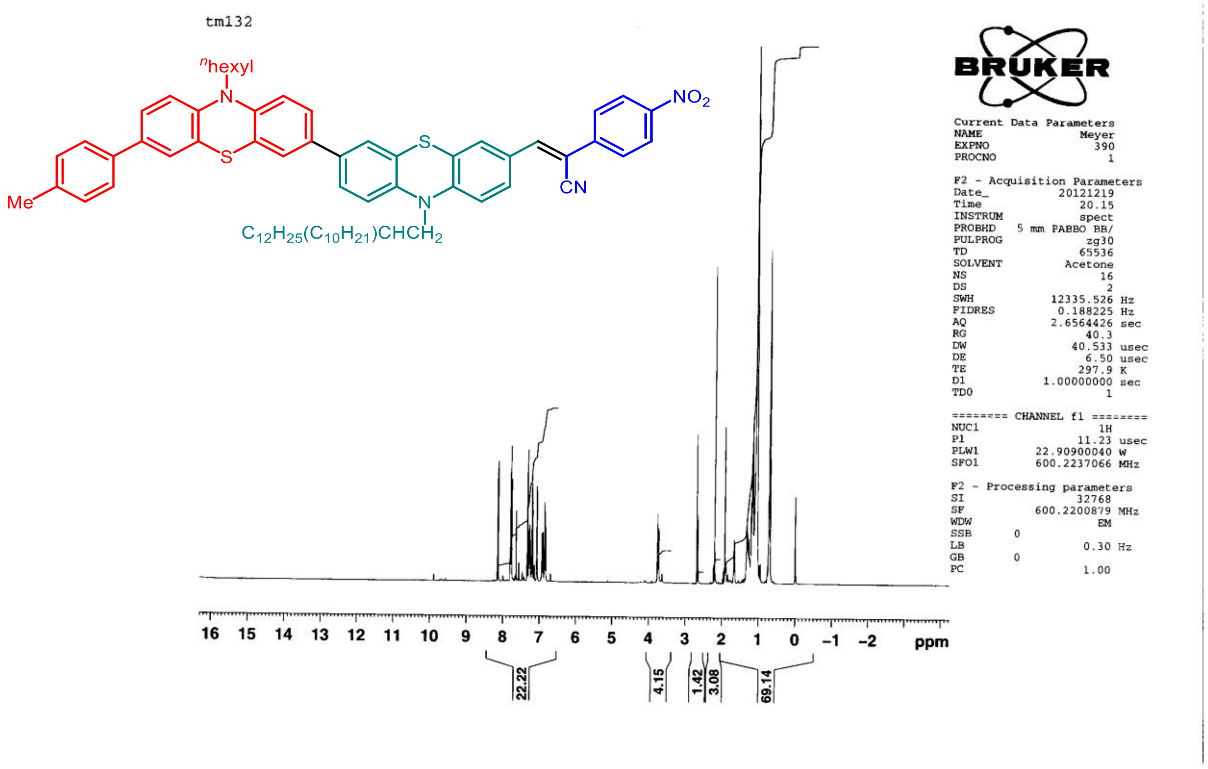

^{1}H NMR (600 MHz, acetone-d$_6$) of compound **12y**.

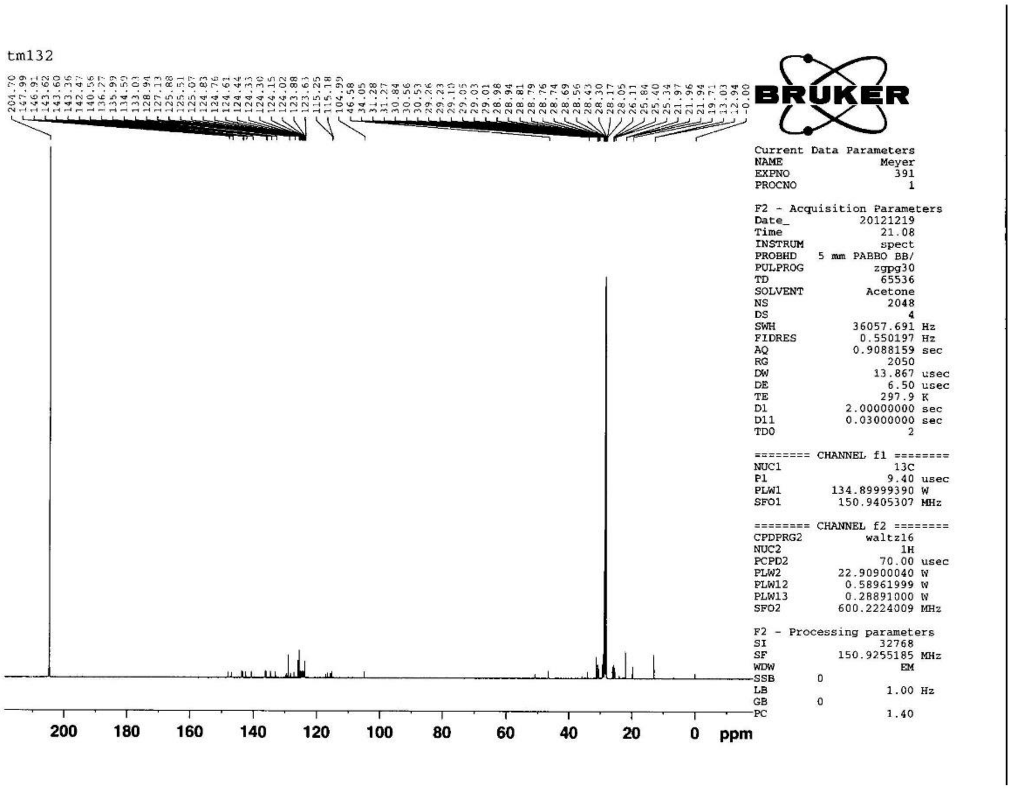

^{13}C NMR (150 MHz, acetone-d$_6$) of compound **12y**.

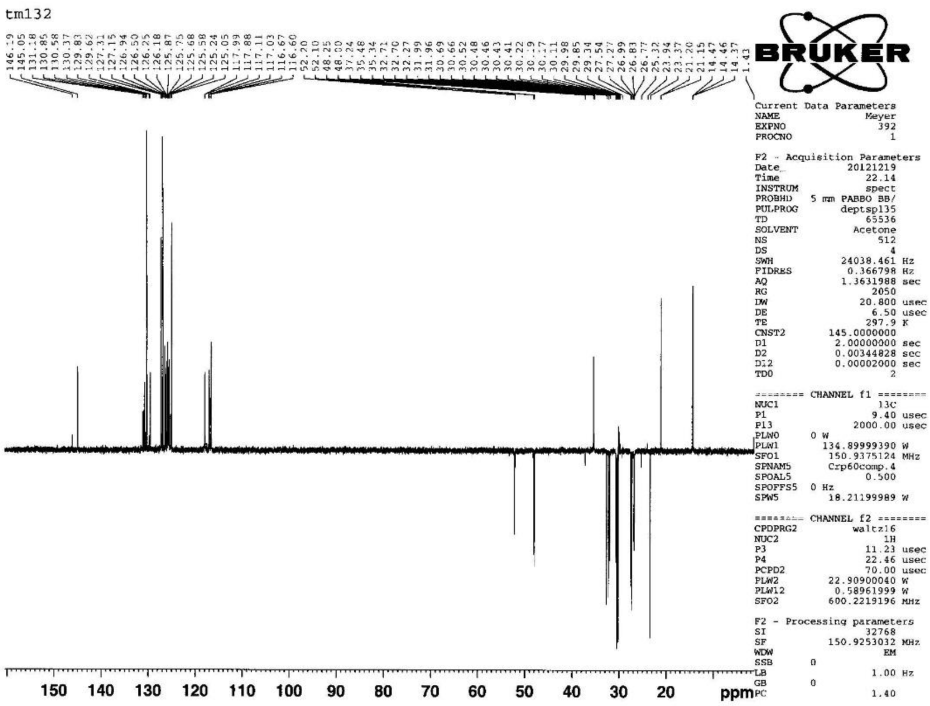

DEPT ^{13}C NMR (150 MHz, acetone-d_6) of compound **12y**.

3.59. (*Z*)-4-{[10-(2-Decyltetradecyl)-10'-hexyl-7'-(*p*-tolyl)-10H,10'H-(3,3'-biphenothiazin)-7-yl]methylene}-3-methyl-1-phenyl-1H-pyrazol-5[4H]-one (12z)

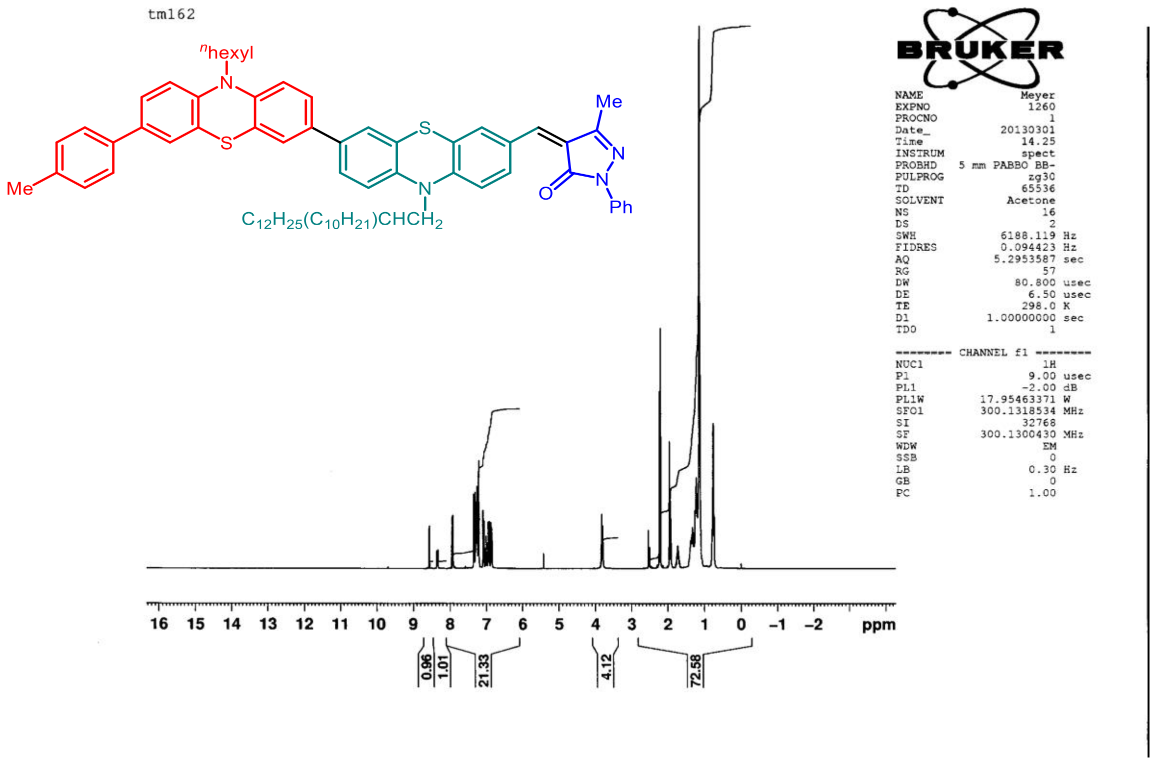

^{1}H NMR (300 MHz, acetone-d_6/CS_2 4:1) of compound **12z**.

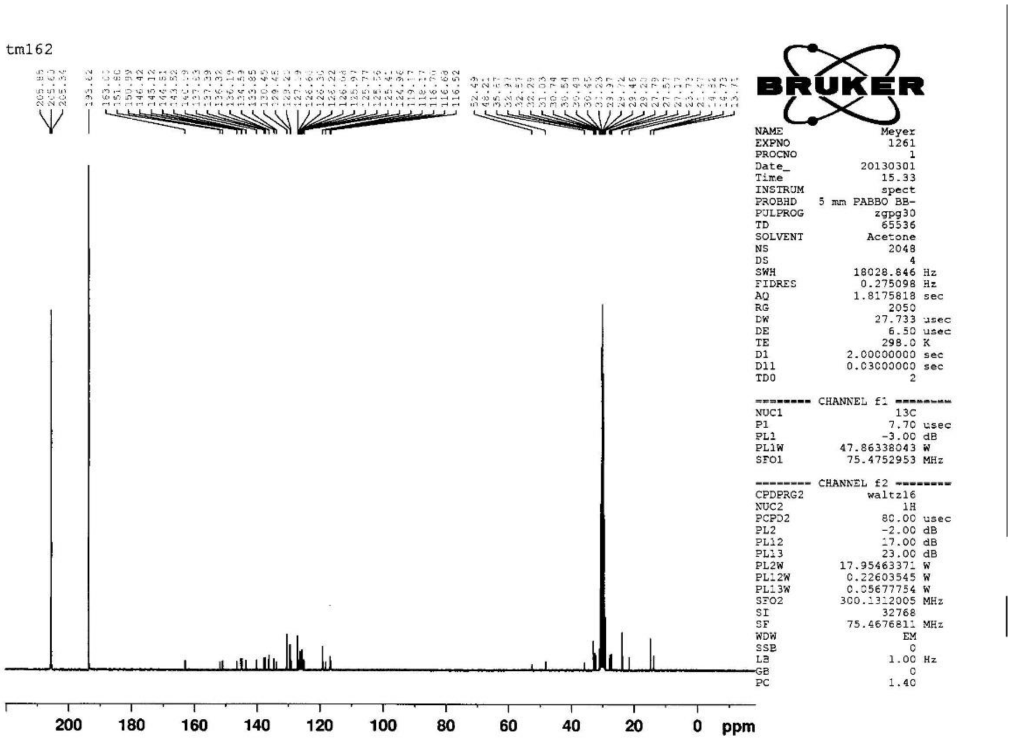

^{13}C NMR (75 MHz, acetone-d_6/CS_2 4:1) of compound **12z**.

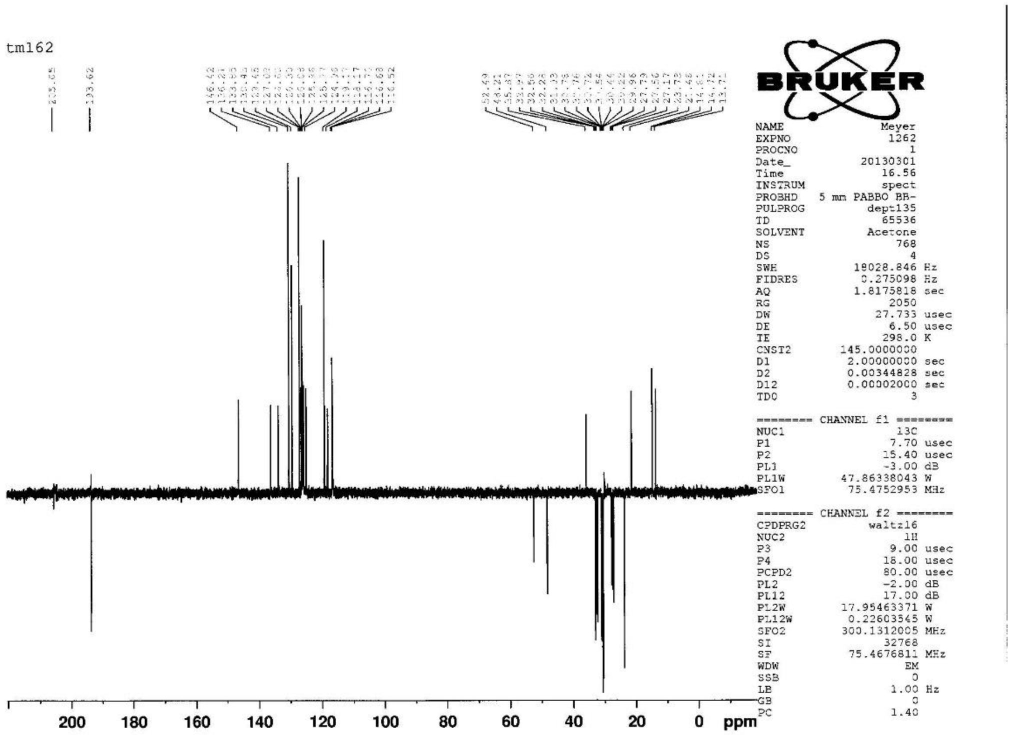

DEPT ^{13}C NMR (75 MHz, acetone-d_6/CS_2 4:1) of compound **12z**.

3.60. 2-{[10-(2-Decyltetradecyl)-10*H*-phenothiazin-3-yl]methylene}-1*H*-inden-1,3[2*H*]-dione (14)

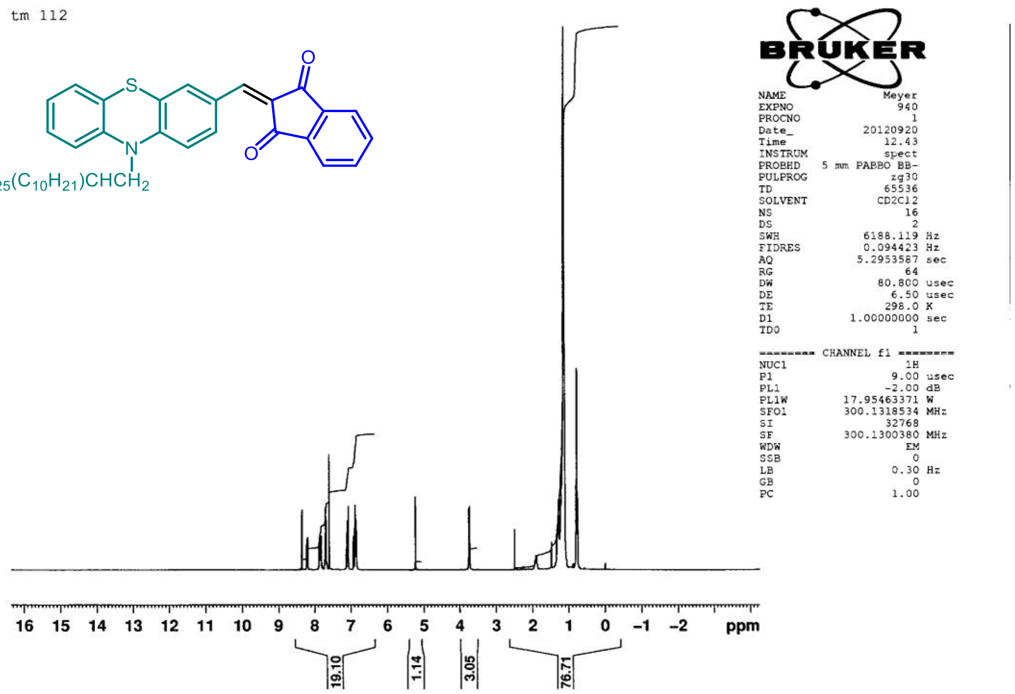

^{1}H NMR (300 MHz, CD_2Cl_2) of compound **14**.

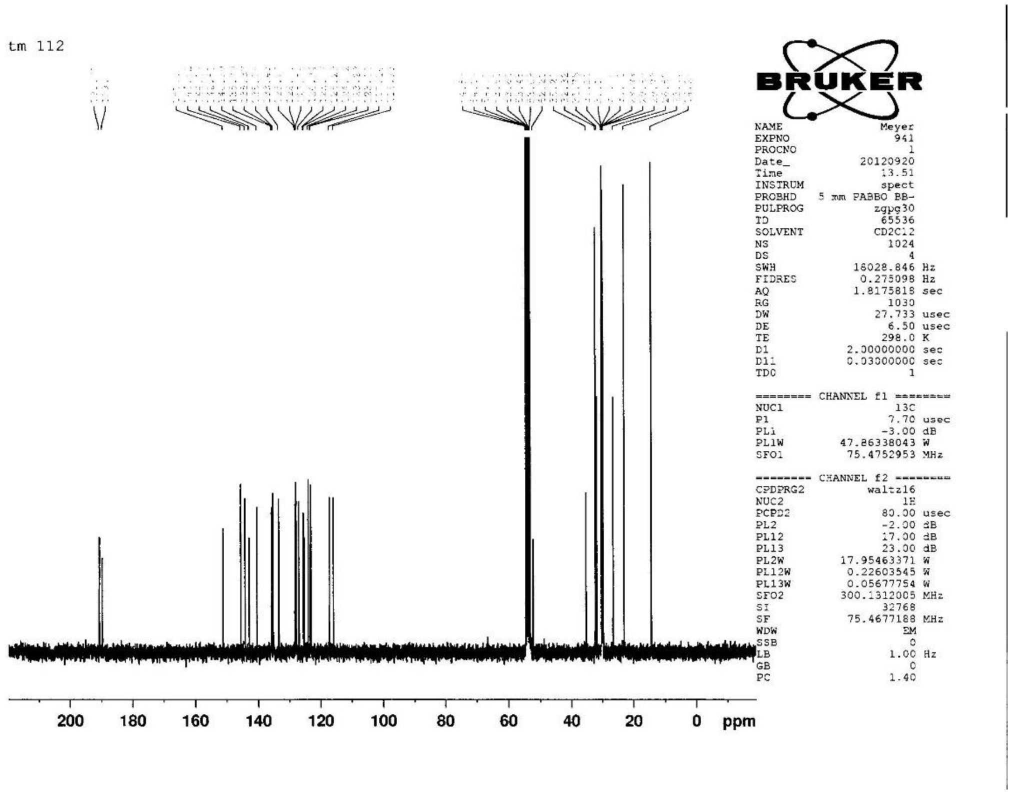

^{13}C NMR (75 MHz, CD_2Cl_2) of compound **14**.

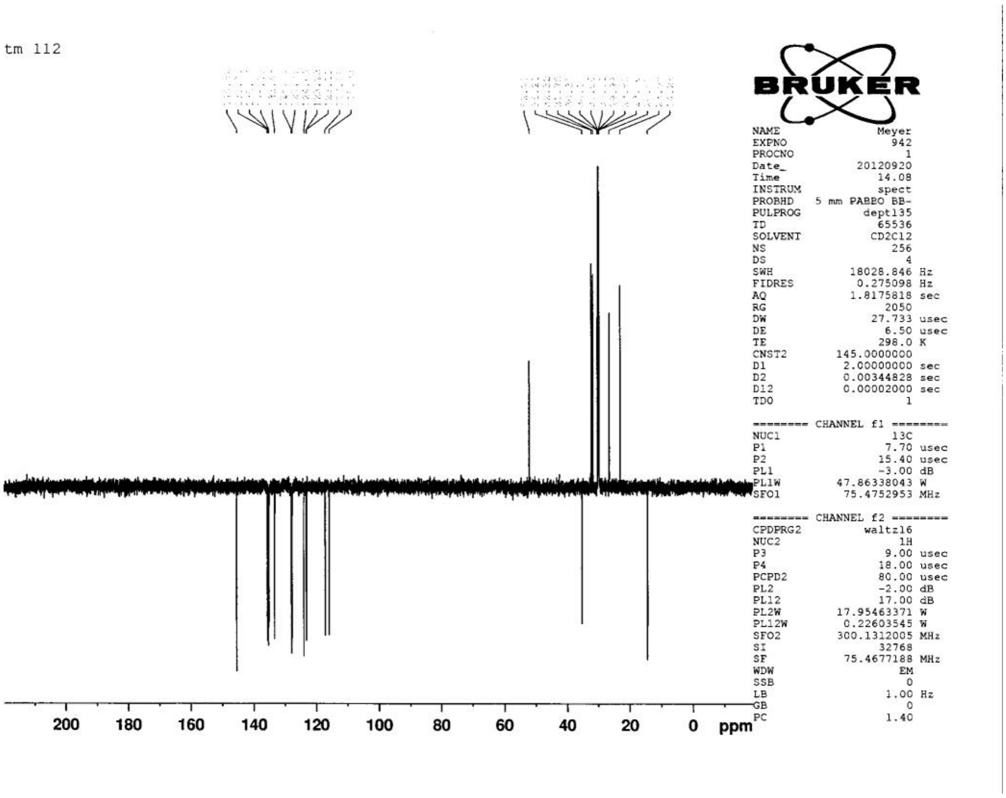

DEPT ^{13}C NMR (75 MHz, CD_2Cl_2) of compound **14**.

3.61. (*Z*)-5-{[10-(2-Decyltetradecyl)-10H-phenothiazin-3-yl]methylene}-3-methyl-2-thioxothiazolidin-4-one (15)

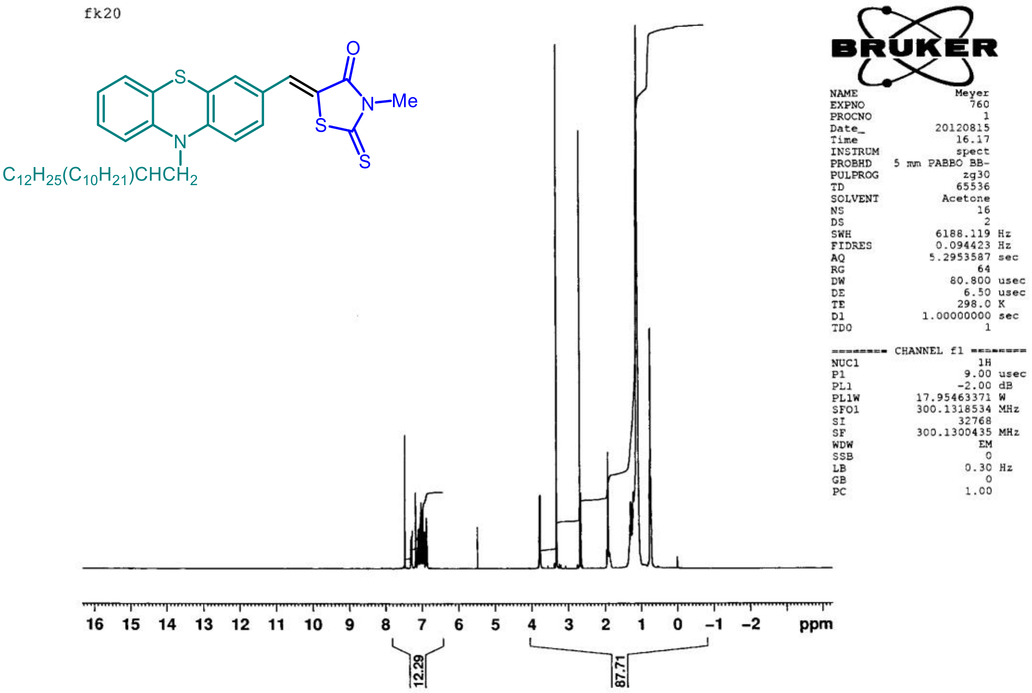

^{1}H NMR (300 MHz, acetone-d_6) of compound **15**.

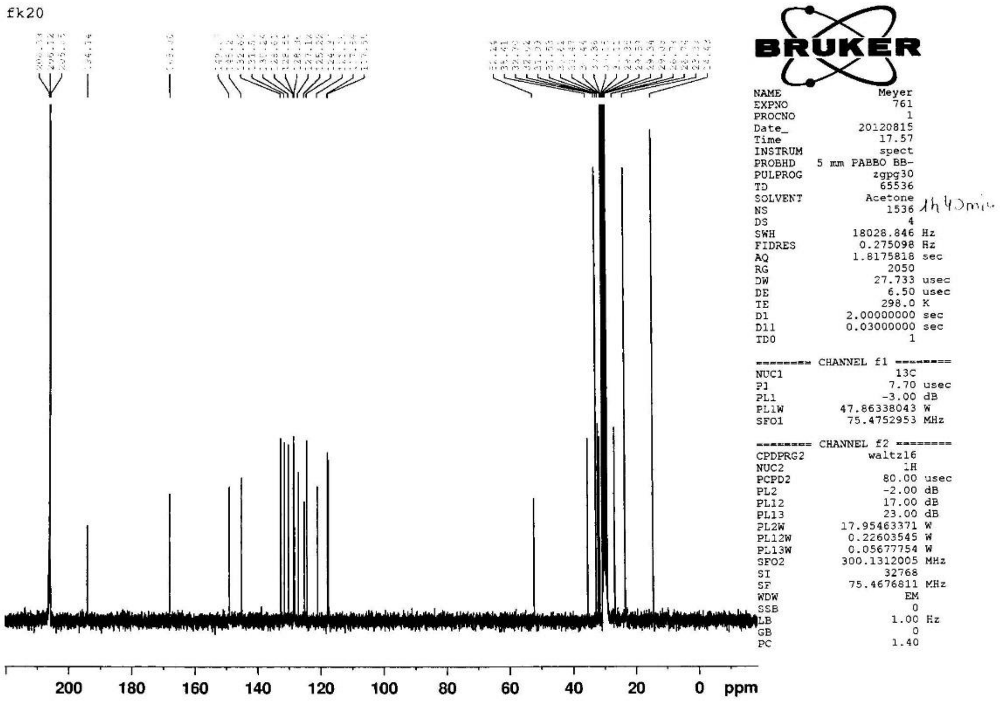

^{13}C NMR (75 MHz, acetone-d_6) of compound **15**.

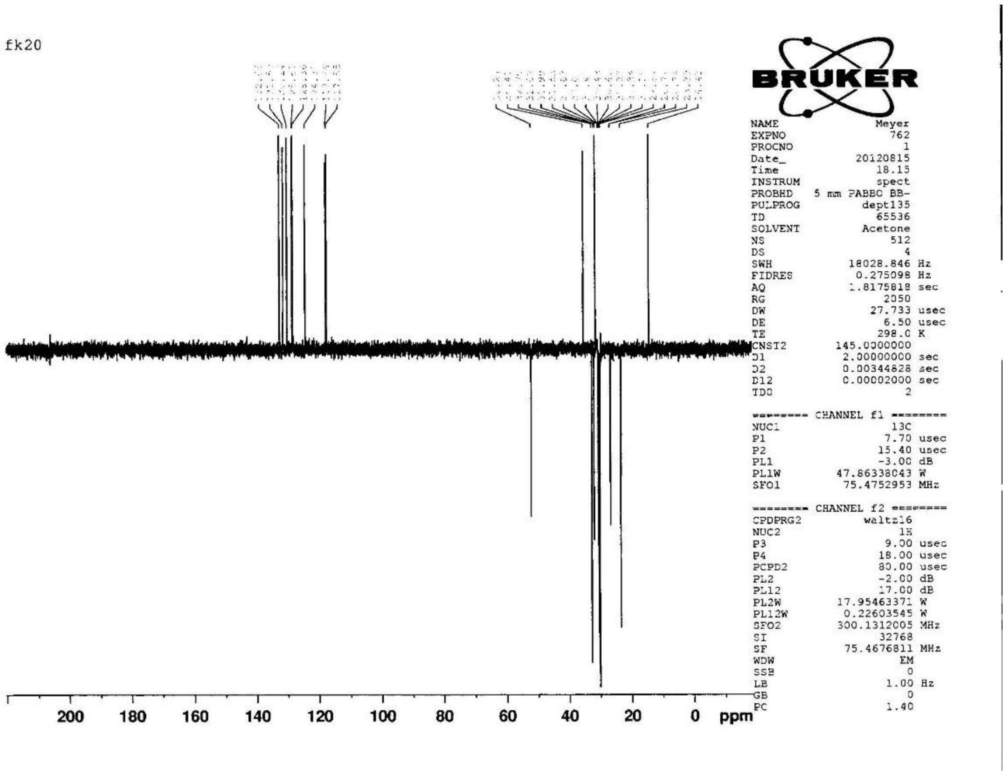

DEPT ^{13}C NMR (75 MHz, acetone-d_6) of compound **15**.

3.62. (*Z*)-3-(10-(2-Decyltetradecyl)-10*H*-phenothiazin-3-yl)-2-(4-nitrophenyl)acrylonitrile (16)

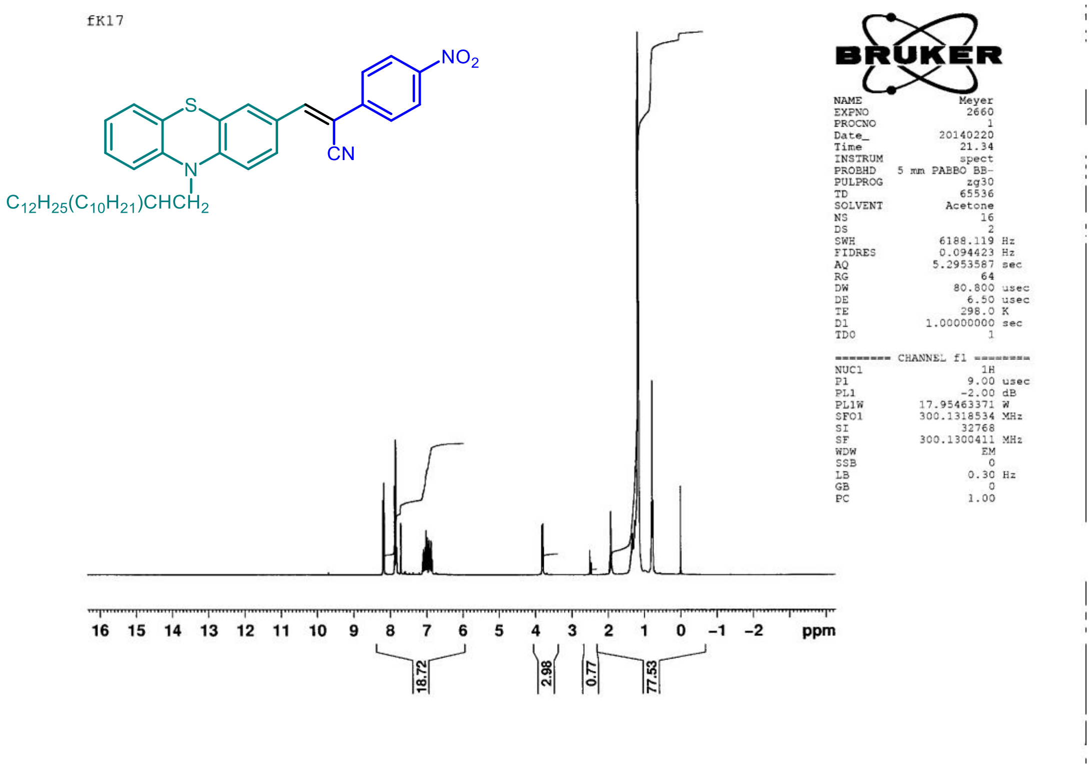

^{1}H NMR (300 MHz, acetone-d$_6$) of compound **16**.

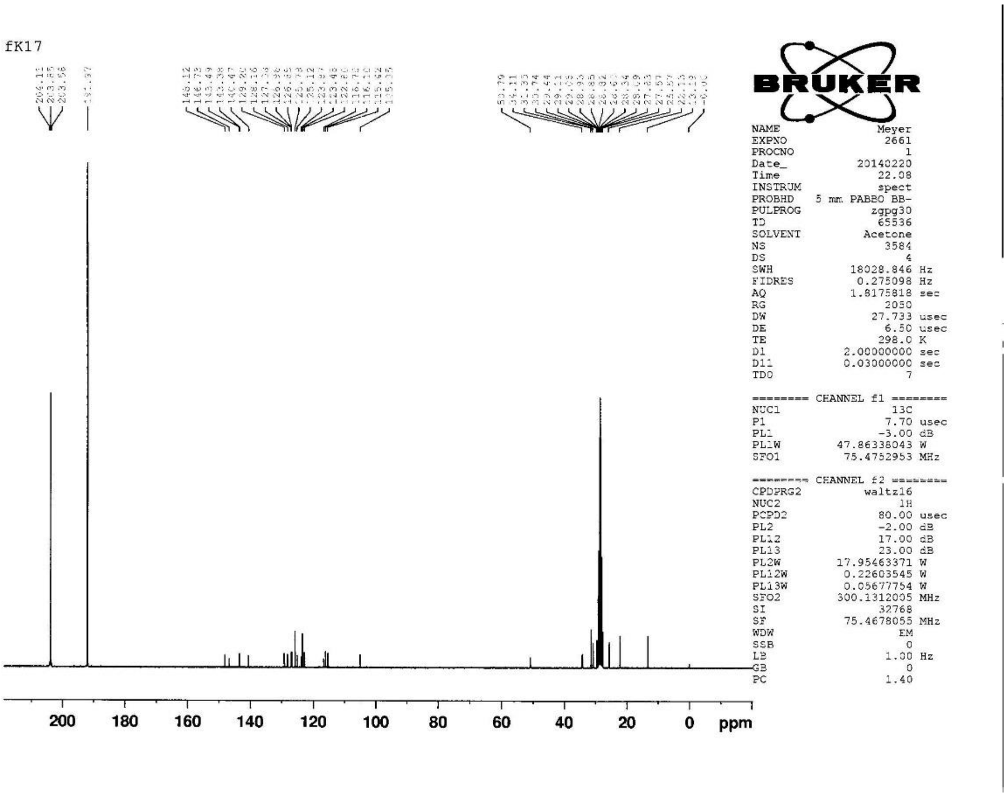

^{13}C NMR (75 MHz, acetone-d$_6$) of compound **16**.

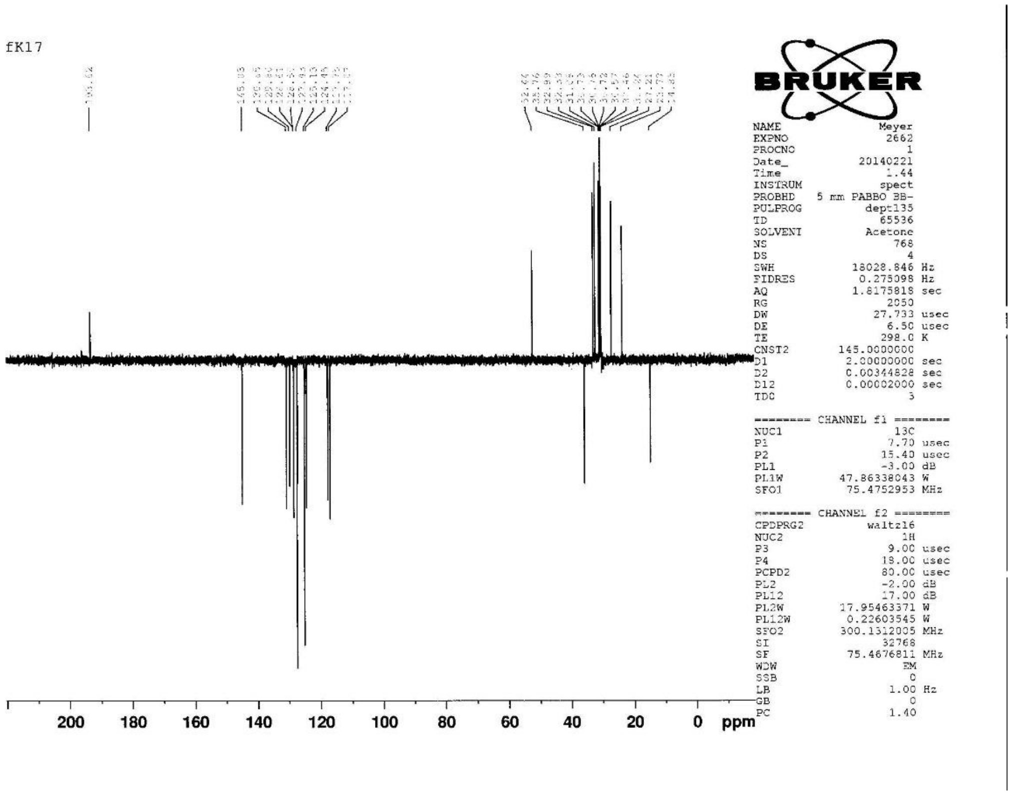

DEPT ^{13}C NMR (75 MHz, acetone-d_6) of compound **16**.

3.63. (*Z*)-4-{[10-(2-Decyltetradecyl)-10*H*-phenothiazin-3-yl]methylene}-5-methyl-2-phenyl-2,4-dihydro-3*H*-pyrazol-3-one (17)

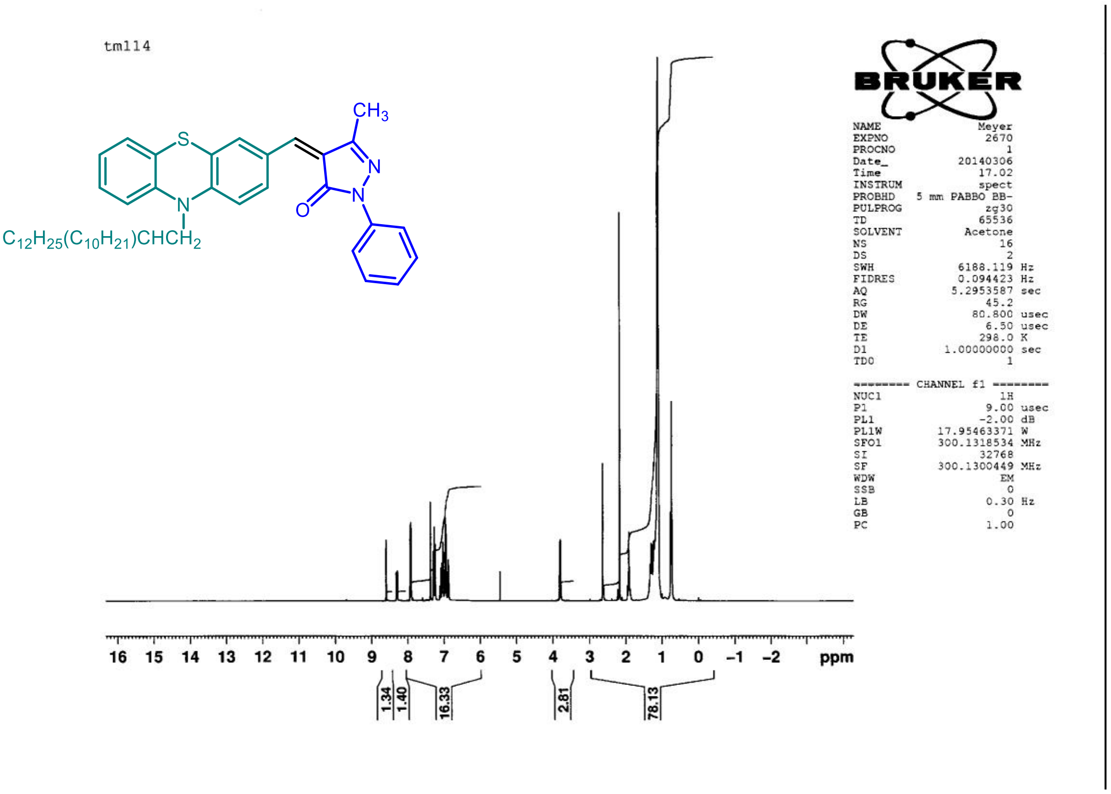

^{1}H NMR (300 MHz, CD_2Cl_2) of compound **17**.

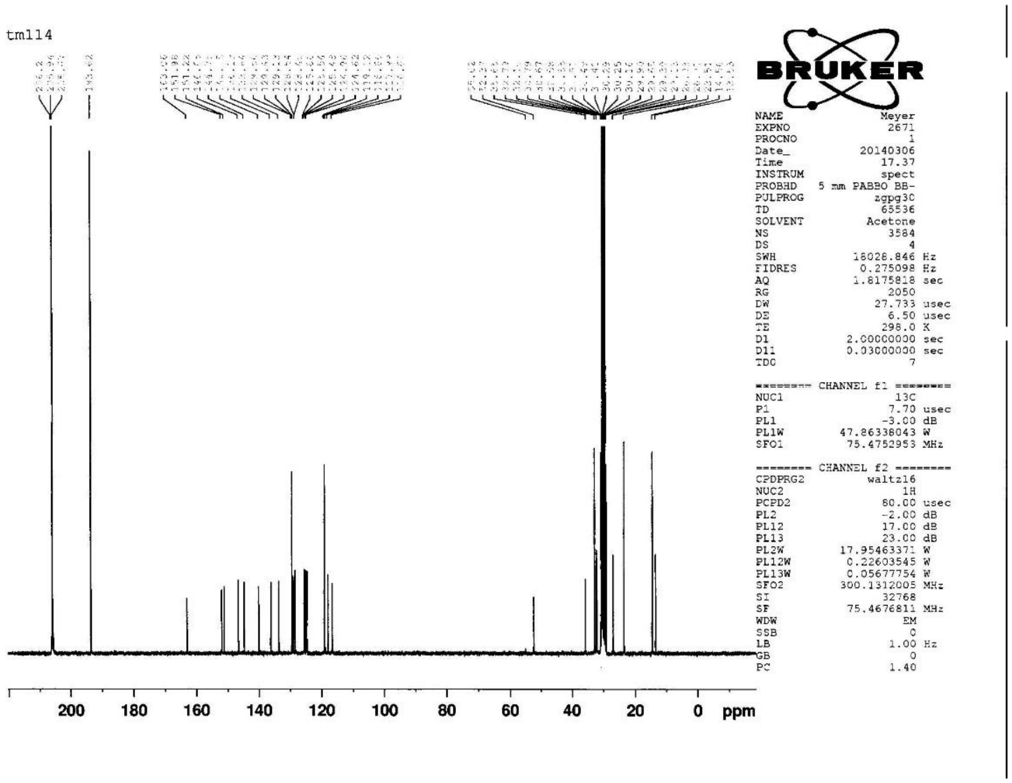

^{13}C NMR (75 MHz, CD_2Cl_2) of compound **17**.

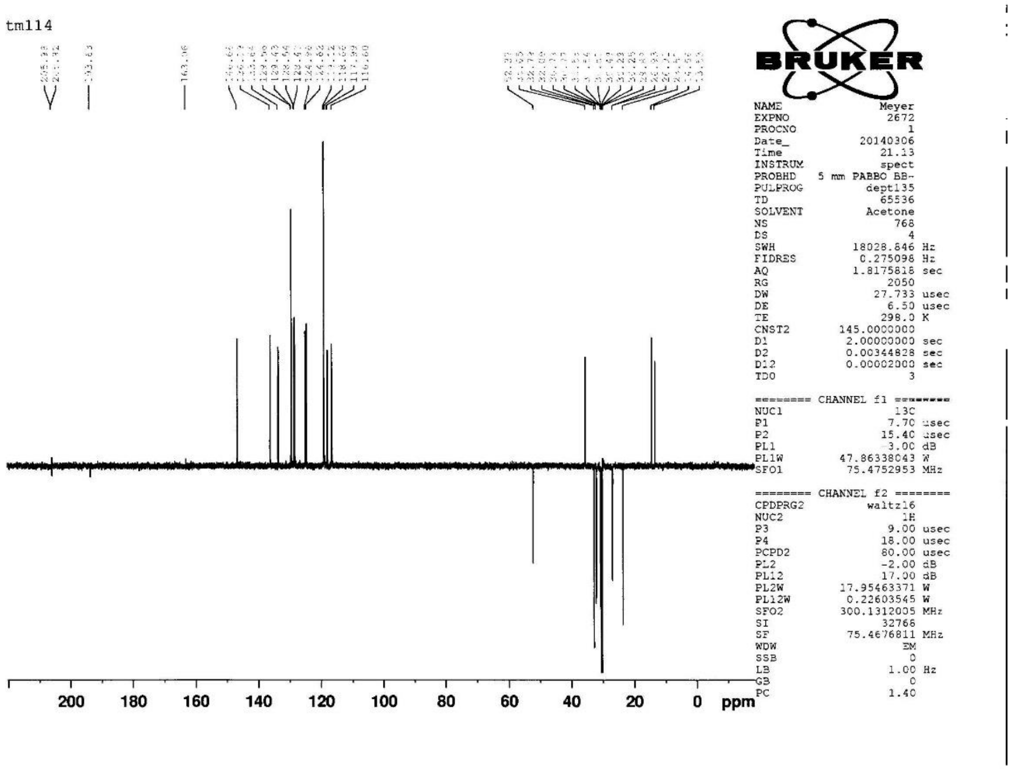

DEPT ^{13}C NMR (75 MHz, CD_2Cl_2) of compound **17**.

4. Correlation Analyses

4.1. Correlations of the Model Chromophores 14-17

Table S6. Selected electronic data of chromophores **14-17**

Compound	$E_{1/2}$ [V]	$\lambda_{max,abs}$ [eV]	$\lambda_{max,em}$ [eV]	$\Delta\tilde{\nu}$ [eV]	E_{0-0} [eV]
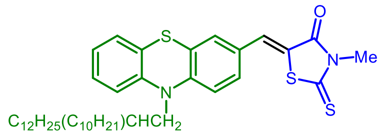 **15**	1.09	2.629	1.933	0.696	2.281
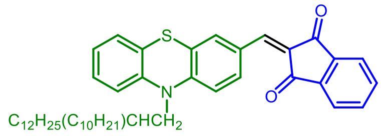 **14**	1.10	2.467	1.841	0.626	2.154
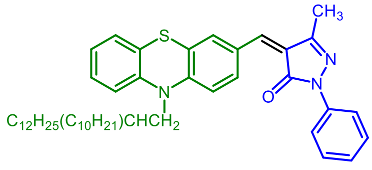 **17**	1.09	2.607	1.939	0.668	2.273
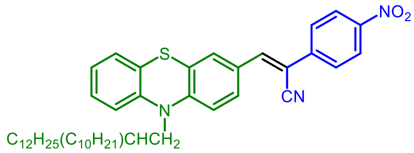 **16**	1.08	2.686	2.196	0.489	2.441

For linear correlation analyses the corresponding optical parameters (in eV) were plotted against the first oxidation potential $E_{1/2}$ (in V). The goodness of fit is expressed by the quadratic linear correlation coefficient r^2 (see Figures S1-S4).

$\lambda_{max,abs}$ vs $E_{1/2}$: $\lambda_{max,abs} = 14.53275 – 10.95 \cdot E_{1/2}$ [eV]; $r^2 = 0.92429$.

$\lambda_{max,em}$ vs $E_{1/2}$: $\lambda_{max,em} = 21.32475 – 17.75 \cdot E_{1/2}$ [eV]; $r^2 = 0.90228$.

$\Delta\tilde{\nu}$ vs $E_{1/2}$: $\Delta\tilde{\nu} = –6.84675 – 6.85 \cdot E_{1/2}$ [eV]; $r^2 = 0.37127$.

E_{00} vs $E_{1/2}$: $E_{00} = 17.92875 – 14.35 \cdot E_{1/2}$ [eV]; $r^2 = 0.98914$.

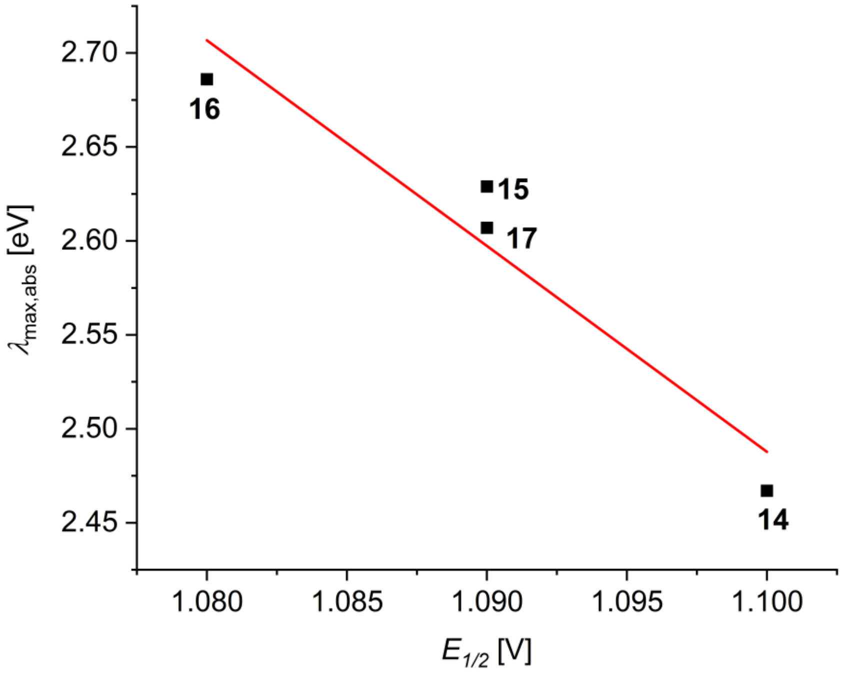

Figure S1. Correlation $\lambda_{max,abs}$ vs $E_{1/2}$: $\lambda_{max,abs} = 14.53275 - 10.95 \cdot E_{1/2}$ [eV]; $r^2 = 0.92429$.

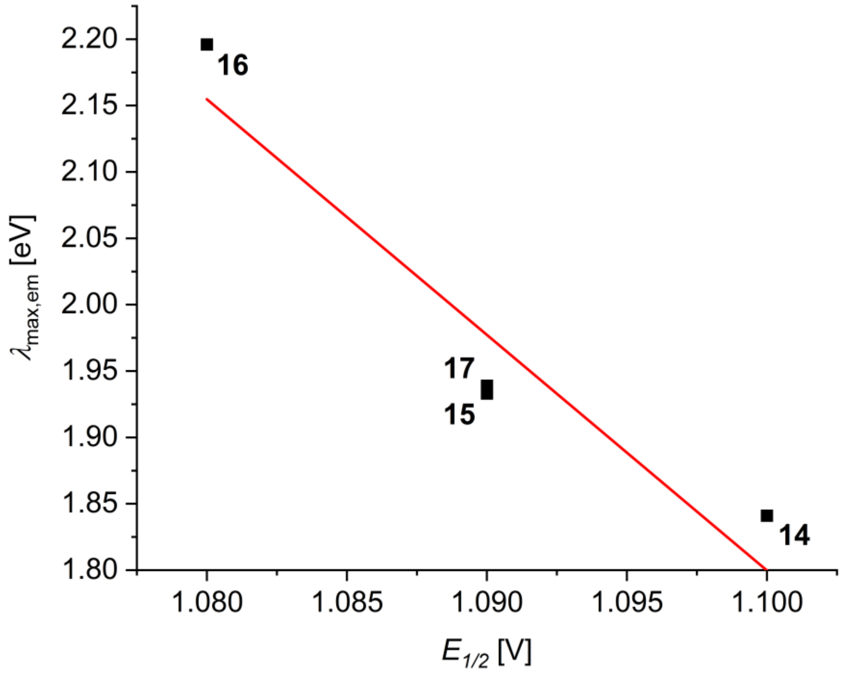

Figure S2. Correlation $\lambda_{max,em}$ vs $E_{1/2}$: $\lambda_{max,em} = 21.32475 - 17.75 \cdot E_{1/2}$ [eV]; $r^2 = 0.90228$.

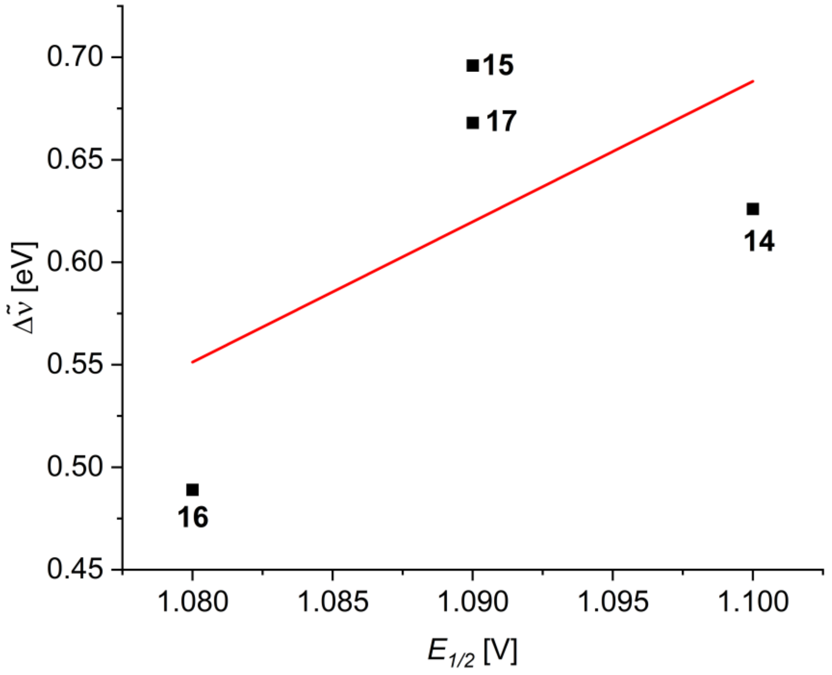

Figure S3. Correlation $\Delta\tilde{\nu}$ vs $E_{1/2}$: $\Delta\tilde{\nu} = -6.84675 - 6.85 \cdot E_{1/2}$ [eV]; $r^2 = 0.37127$.

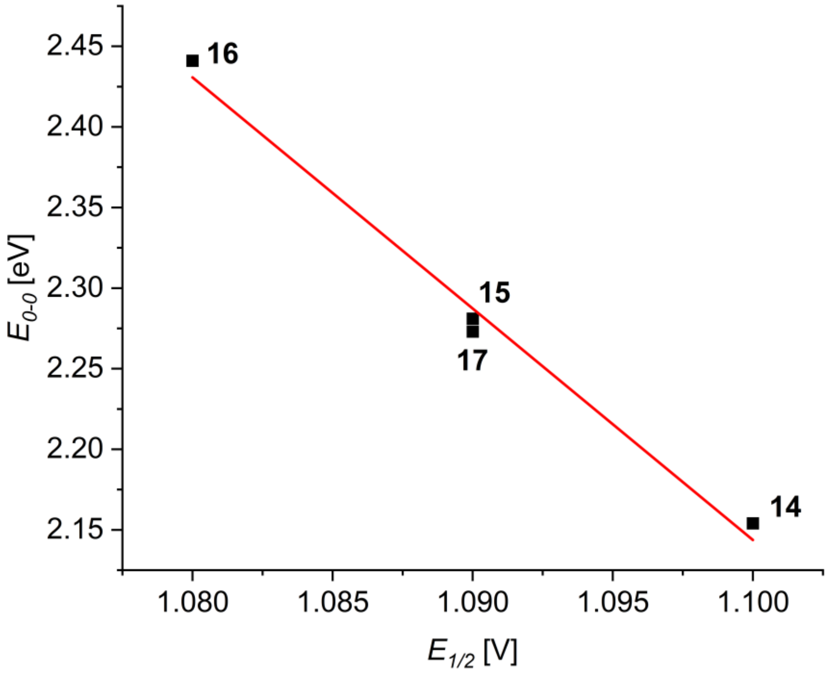

Figure S4. Correlation E_{00} vs $E_{1/2}$: $E_{00} = 17.92875 - 14.35 \cdot E_{1/2}$ [eV]; $r^2 = 0.98914$.

4.2. Correlations of Consanguineous Acceptor Series

Table S7. Selected electronic properties of merocyanine series **9**, **11**, **12**, and **14-17** employed in the correlation analyses.

Donor / Acceptor	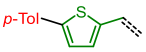 **9a, 9b, 9d, 9e**		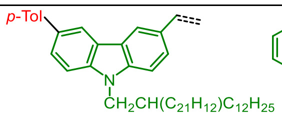 **11a, 11c, 11d, 11e**	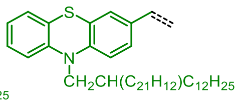 **14-17**	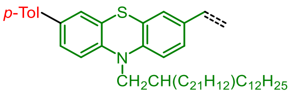 **12a, 12c, 12e, 12f**	
		$E_{1/2}$ [V]	$\lambda_{max,abs}$ [eV]	$\lambda_{max,em}$ [eV]	$\Delta\tilde{\nu}$ [eV]	E_{0-0} [eV]
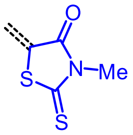	**9a**	1.66	2.752	2.414	0.337	2.583
	**11a**	1.44	2.740	2.359	0.380	2.549
	**12a**	1.05	2.575	1.895	0.680	2.235
	**15**	1.09	2.629	1.933	0.696	2.281
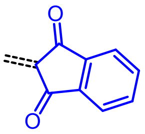	**9b**	1.76	2.758	2.419	0.339	2.588
	**11c**	1.46	2.675	2.212	0.462	2.443
	**12c**	1.05	2.424	1.812	0.612	2.118
	**14**	1.10	2.467	1.841	0.626	2.154
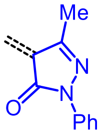	**9d**	1.61	2.948	2.580	0.368	2.764
	**11d**	1.45	2.899	2.359	0.540	2.629
	**12e**	1.05	2.548	1.945	0.603	2.246
	**17**	1.09	2.607	1.939	0.668	2.273
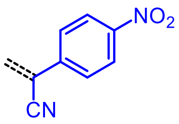	**9e**	1.71	3.207	2.364	0.843	2.785
	**11e**	1.44	2.879	2.224	0.655	2.552
	**12f**	1.03	2.629	2.140	0.490	2.384
	**16**	1.08	2.686	2.196	0.489	2.441

For linear correlation analyses of consanguineous acceptor series the corresponding optical parameters (in eV) were plotted against the first oxidation potential $E_{1/2}$ (in V). The goodness of fit is expressed by the quadratic linear correlation coefficient r^2 (see Figures S5-S20).

4.2.1. Correlations of 3-Methyl-4-oxo-2-thioxothiazolidin-5-ylidene Merocyanines

$\lambda_{max,abs}$ vs $E_{1/2}$: $\lambda_{max,abs} = 2.30772 + 0.27943 \cdot E_{1/2}$ [eV]; $r^2 = 0.89872$.

$\lambda_{max,em}$ vs $E_{1/2}$: $\lambda_{max,em} = 0.95076 + 0.91572 \cdot E_{1/2}$ [eV]; $r^2 = 0.95763$.

$\Delta\tilde{\nu}$ vs $E_{1/2}$: $\Delta\tilde{\nu} = 1.35696 - 0.63628 \cdot E_{1/2}$ [eV]; $r^2 = 0.94385$.

E_{00} vs $E_{1/2}$: $E_{00} = 1.62924 + 0.59758 \cdot E_{1/2}$ [eV]; $r^2 = 0.94309$.

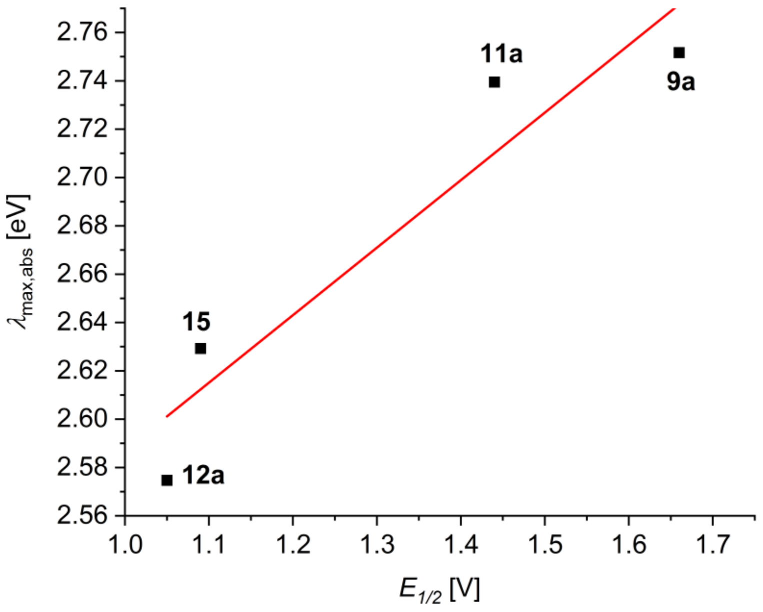

Figure S5. Correlation $\lambda_{max,abs}$ vs $E_{1/2}$: $\lambda_{max,abs} = 2.30772 + 0.27943 \cdot E_{1/2}$ [eV]; $r^2 = 0.89872$.

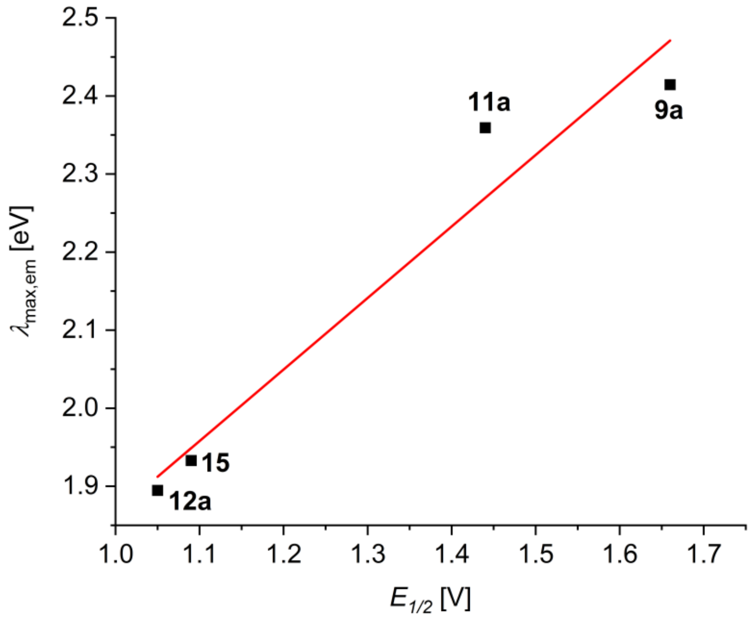

Figure S6. Correlation $\lambda_{max,em}$ vs $E_{1/2}$: $\lambda_{max,em} = 0.95076 + 0.91572 \cdot E_{1/2}$ [eV]; $r^2 = 0.95763$.

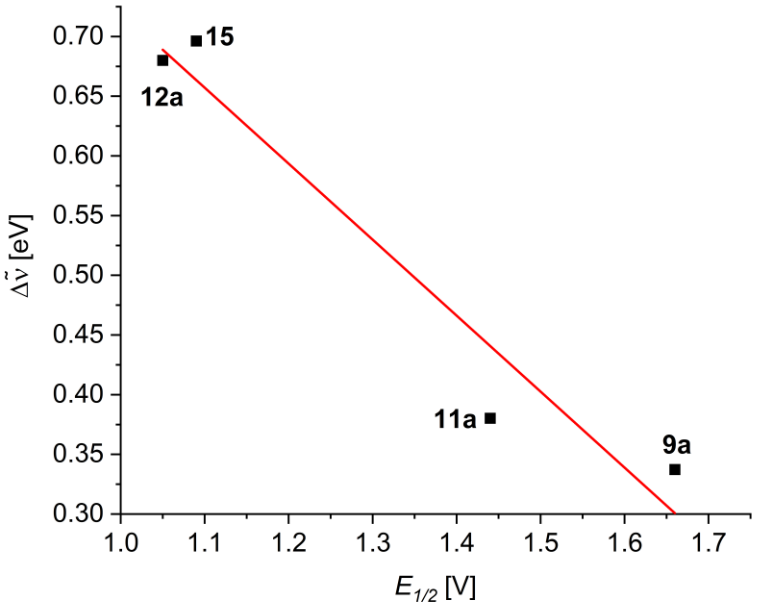

Figure S7. Correlation $\Delta\tilde{\nu}$ vs $E_{1/2}$: $\Delta\tilde{\nu} = 1.35696 - 0.63628 \cdot E_{1/2}$ [eV]; $r^2 = 0.94385$.

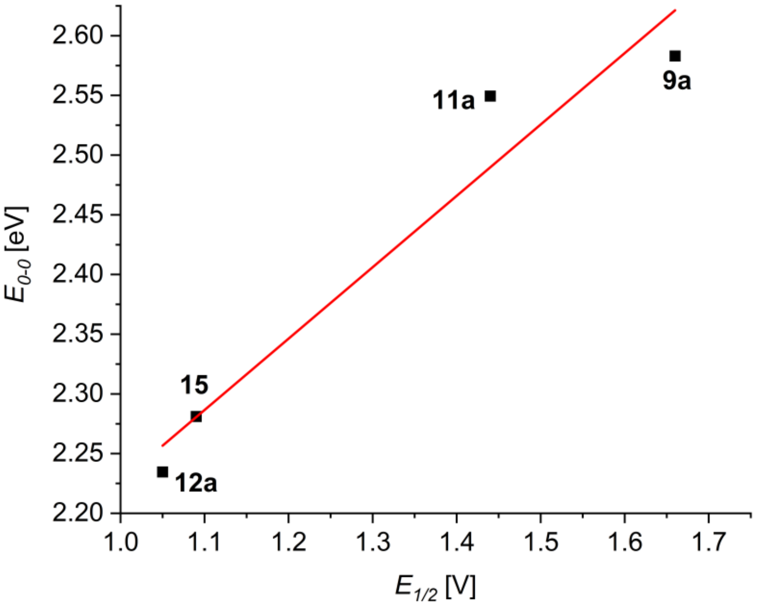

Figure S8. Correlation E_{00} vs $E_{1/2}$: $E_{00} = 1.62924 + 0.59758 \cdot E_{1/2}$ [eV]; $r^2 = 0.94309$.

4.2.2. Correlations of 1,3-Dioxo-1,3-dihydro-2*H*-inden-2-ylidene Merocyanines

$\lambda_{max,abs}$ vs $E_{1/2}$: $\lambda_{max,abs} = 1.94136 + 0.47633 \cdot E_{1/2}$ [eV]; $r^2 = 0.97137$.

$\lambda_{max,em}$ vs $E_{1/2}$: $\lambda_{max,em} = 0.88599 + 0.88272 \cdot E_{1/2}$ [eV]; $r^2 = 0.9921$.

$\Delta\tilde{\nu}$ vs $E_{1/2}$: $\Delta\tilde{\nu} = 1.05538 - 0.40638 \cdot E_{1/2}$ [eV]; $r^2 = 0.98946$.

E_{00} vs $E_{1/2}$: $E_{00} = 1.41368 + 0.67953 \cdot E_{1/2}$ [eV]; $r^2 = 0.98716$.

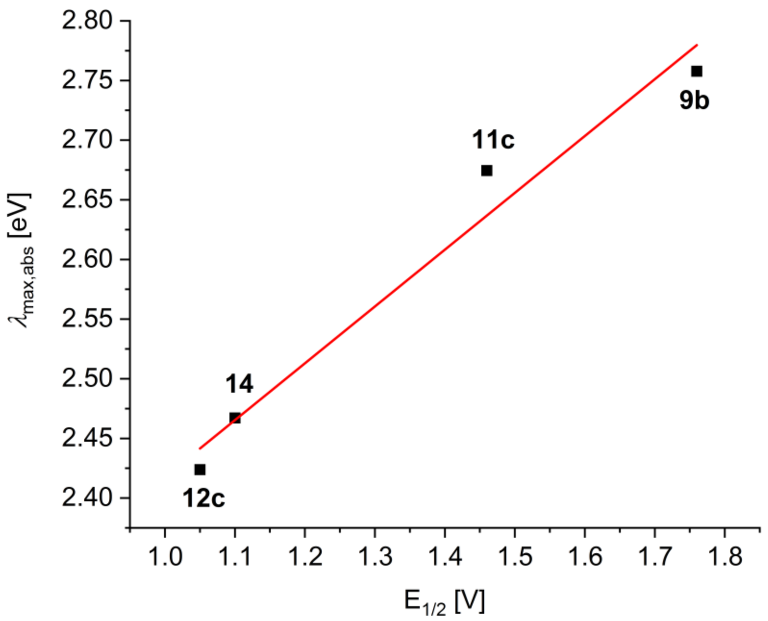

Figure S9. Correlation $\lambda_{max,abs}$ vs $E_{1/2}$: $\lambda_{max,abs} = 1.94136 + 0.47633 \cdot E_{1/2}$ [eV]; $r^2 = 0.97137$.

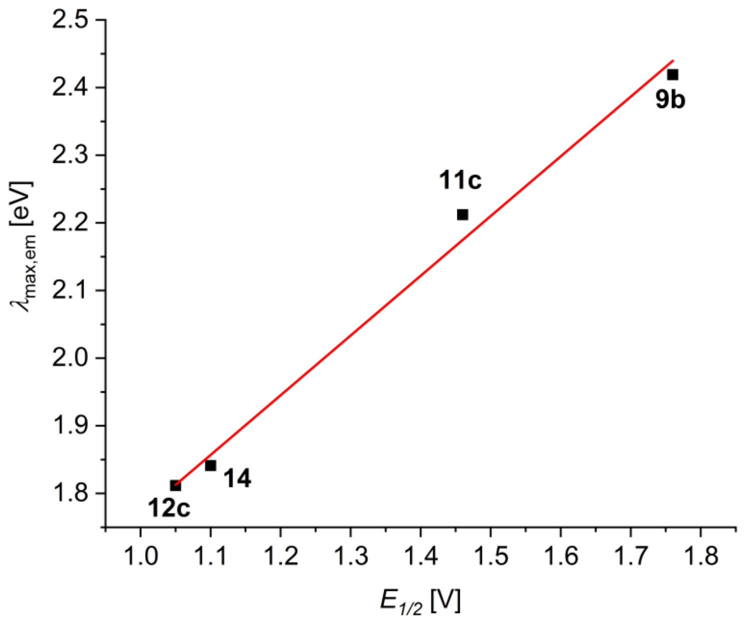

Figure S10. Correlation $\lambda_{max,em}$ vs $E_{1/2}$: $\lambda_{max,em} = 0.88599 + 0.88272 \cdot E_{1/2}$ [eV]; $r^2 = 0.9921$.

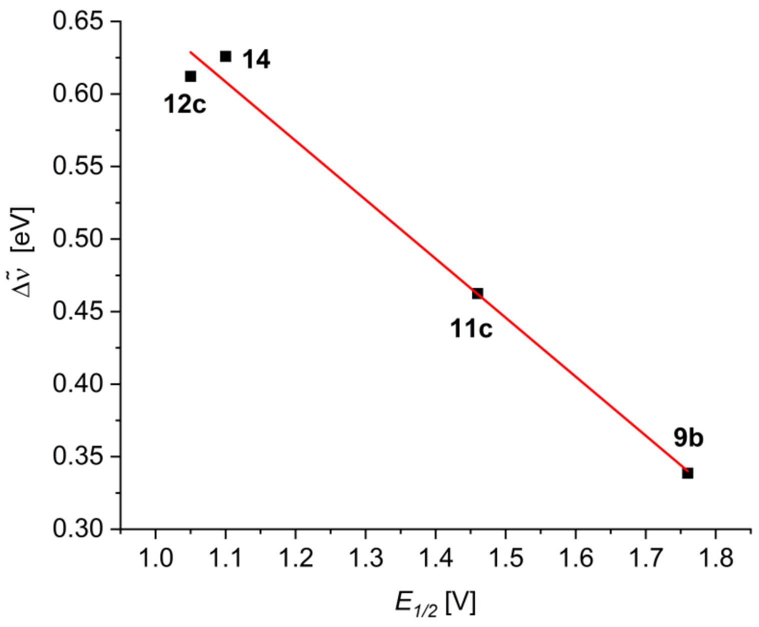

Figure S11. Correlation $\Delta\tilde{\nu}$ vs $E_{1/2}$: $\Delta\tilde{\nu} = 1.05538 - 0.40638 \cdot E_{1/2}$ [eV]; $r^2 = 0.98946$.

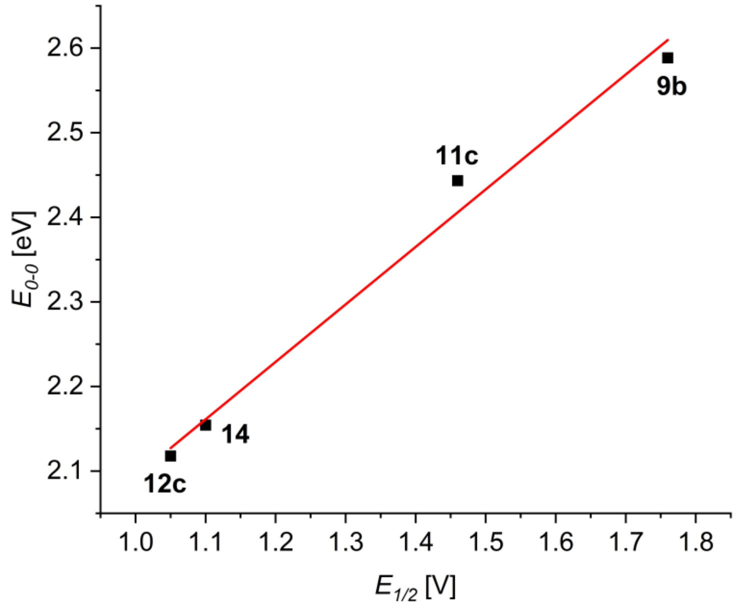

Figure S12. Correlation E_{00} vs $E_{1/2}$: $E_{00} = 1.41368 + 0.67953 \cdot E_{1/2}$ [eV]; $r^2 = 0.98716$.

4.2.3. Correlations of 3-Methyl-5-oxo-1-phenyl-1,5-dihydro-4*H*-pyrazol-4-ylidene Merocyanines

$\lambda_{max,abs}$ vs $E_{1/2}$: $\lambda_{max,abs} = 1.80299 + 0.72898 \cdot E_{1/2}$ [eV]; $r^2 = 0.97638$.

$\lambda_{max,em}$ vs $E_{1/2}$: $\lambda_{max,em} = 0.70372 + 1.15551 \cdot E_{1/2}$ [eV]; $r^2 = 0.99331$.

$\Delta\tilde{\nu}$ vs $E_{1/2}$: $\Delta\tilde{\nu} = 1.09927 - 0.42653 \cdot E_{1/2}$ [eV]; $r^2 = 0.81969$.

E_{00} vs $E_{1/2}$: $E_{00} = 1.25336 + 0.94225 \cdot E_{1/2}$ [eV]; $r^2 = 0.99896$.

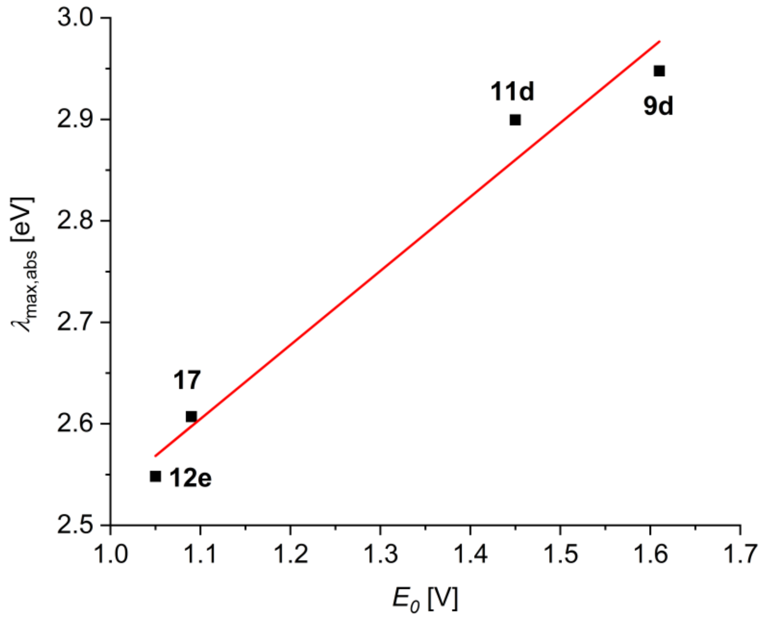

Figure S13. Correlation $\lambda_{max,abs}$ vs $E_{1/2}$: $\lambda_{max,abs} = 1.80299 + 0.72898 \cdot E_{1/2}$ [eV]; $r^2 = 0.97638$.

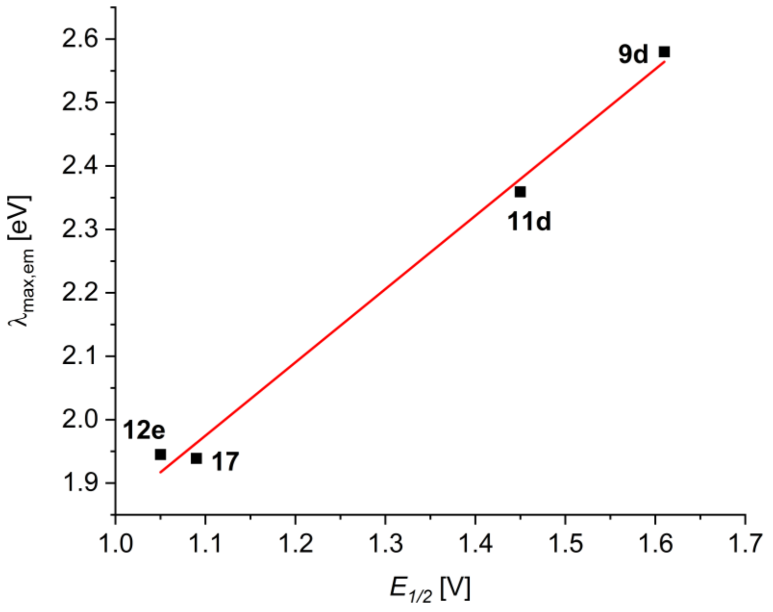

Figure S14. Correlation $\lambda_{max,em}$ vs $E_{1/2}$: $\lambda_{max,em} = 0.70372 + 1.15551 \cdot E_{1/2}$ [eV]; $r^2 = 0.99331$.

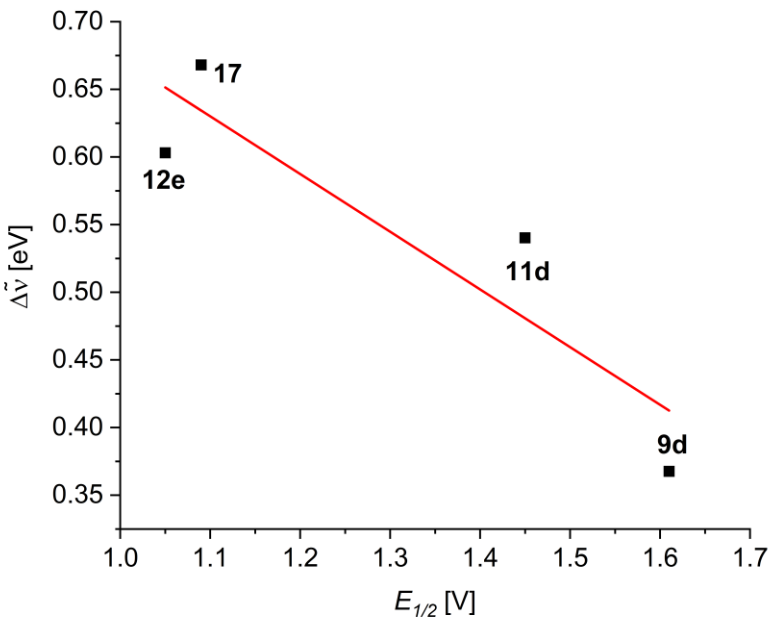

Figure S15. Correlation $\Delta\tilde{\nu}$ vs $E_{1/2}$: $\Delta\tilde{\nu} = 1.09927 - 0.42653 \cdot E_{1/2}$ [eV]; $r^2 = 0.81969$.

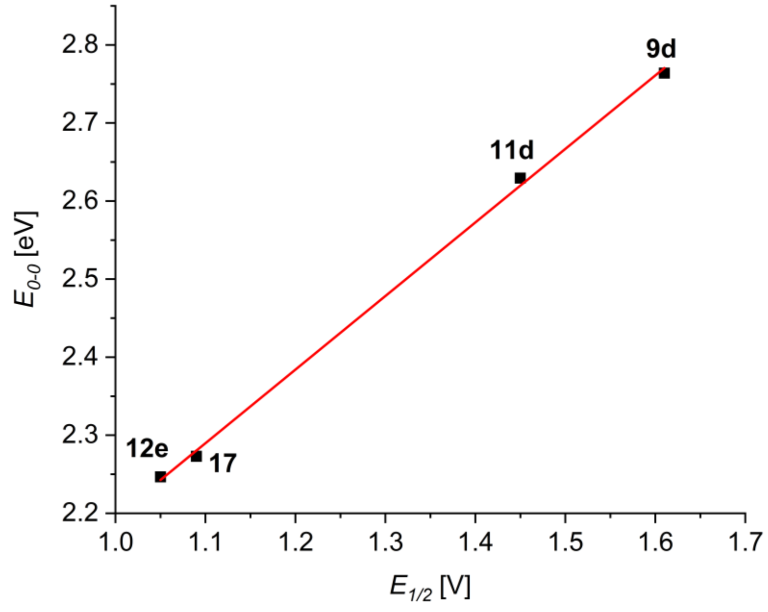

Figure S16. Correlation E_{00} vs $E_{1/2}$: $E_{00} = 1.25336 + 0.94225 \cdot E_{1/2}$ [eV]; $r^2 = 0.99896$.

4.2.4. Correlations of Cyano(4-nitrophenyl)-methylene Merocyanines

$\lambda_{max,abs}$ vs $E_{1/2}$: $\lambda_{max,abs} = 1.80299 + 0.79843 \cdot E_{1/2}$ [eV]; $r^2 = 0.96424$.

$\lambda_{max,em}$ vs $E_{1/2}$: $\lambda_{max,em} = 1.86505 + 0.27828 \cdot E_{1/2}$ [eV]; $r^2 = 0.87637$.

$\Delta\tilde{\nu}$ vs $E_{1/2}$: $\Delta\tilde{\nu} = -0.06462 + 0.52015 \cdot E_{1/2}$ [eV]; $r^2 = 0.98155$.

E_{00} vs $E_{1/2}$: $E_{00} = 1.83274 + 0.53835 \cdot E_{1/2}$ [eV]; $r^2 = 0.94765$.

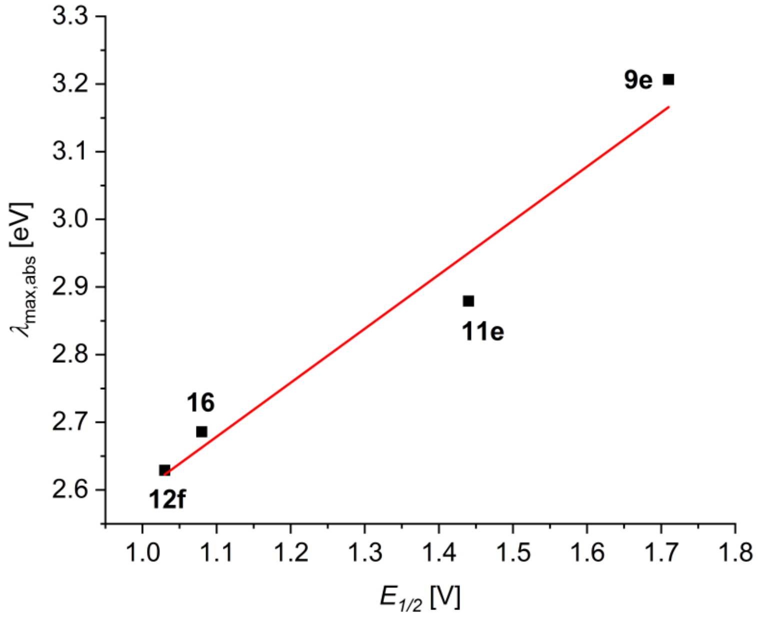

Figure S17. Correlation $\lambda_{max,abs}$ vs $E_{1/2}$: $\lambda_{max,abs} = 1.80299 + 0.79843 \cdot E_{1/2}$ [eV]; $r^2 = 0.96424$.

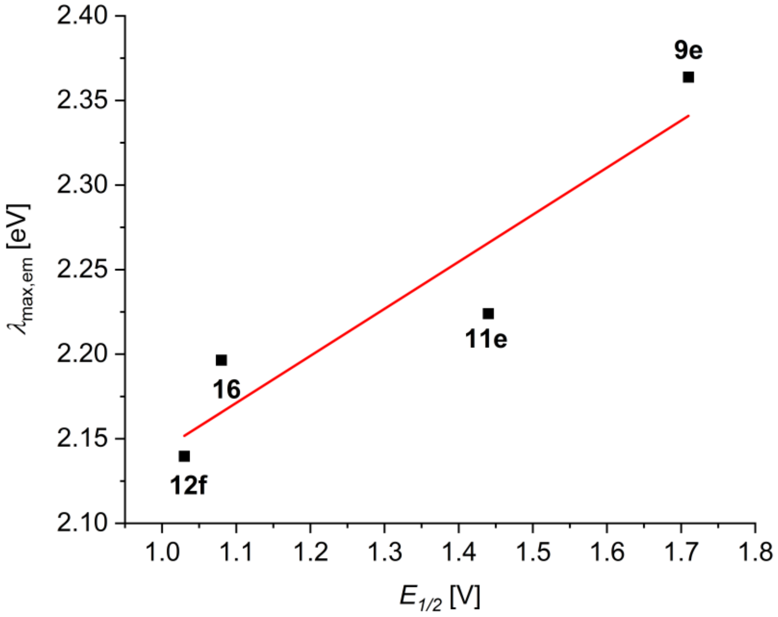

Figure S18. Correlation $\lambda_{max,em}$ vs $E_{1/2}$: $\lambda_{max,em} = 1.86505 + 0.27828 \cdot E_{1/2}$ [eV]; $r^2 = 0.87637$.

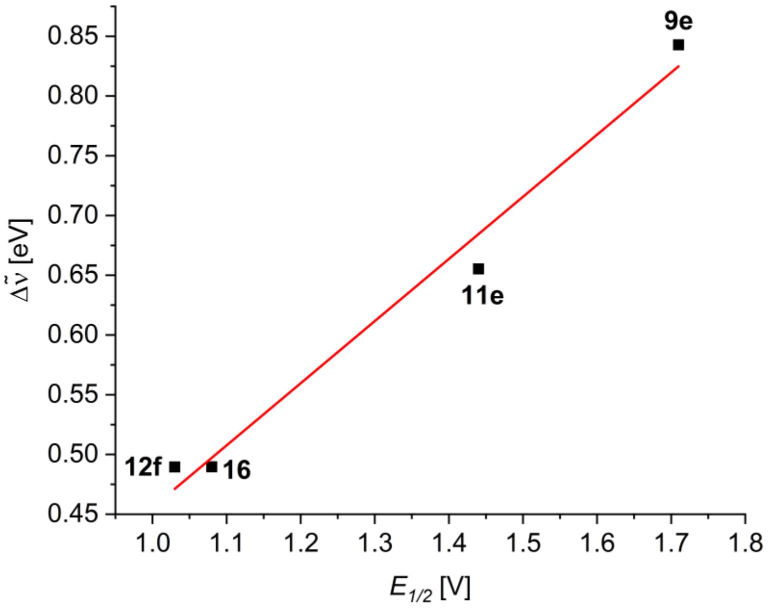

Figure S19. Correlation $\Delta\tilde{\nu}$ vs $E_{1/2}$: $\Delta\tilde{\nu} = –0.06462 + 0.52015 \cdot E_{1/2}$ [eV]; $r^2 = 0.98155$.

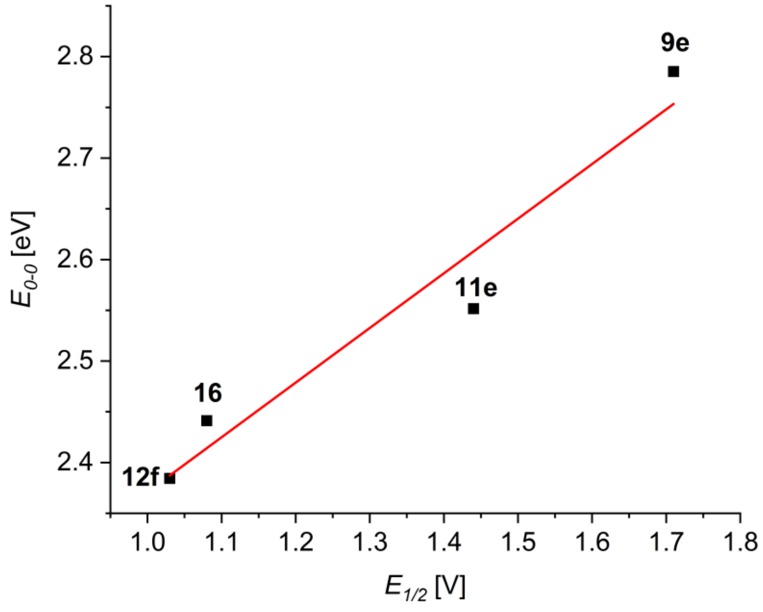

Figure S20. Correlation E_{00} vs $E_{1/2}$: $E_{00} = 1.83274 + 0.53835 \cdot E_{1/2}$ [eV]; $r^2 = 0.94765$.

4.3. Linear and Planar Correlations with 24 Compounds

For linear correlation analysis the optical band gaps E_{0-0} (in eV) were plotted against the first oxidation potential $E_{1/2}$ (in V). The goodness of fit is expressed by the quadratic linear correlation coefficient r^2 (see Figure S21). For a two parameter planar correlation analysis the optical band gaps E_{0-0} (in eV) were plotted against the first oxidation potential $E_{1/2}$ (in V) and the emission maxima $\lambda_{max,em}$ [in eV]. The goodness of fit is expressed by the quadratic linear correlation coefficient r^2 (see Figure S22).

Table S8. Selected electronic properties of 24 merocyanines the series **9**, **11**, **12**, and **14-17** employed in the correlation analysis.

Compound	$E_{1/2}$ [V]	$\lambda_{max,em}$ [eV]	E_{0-0} [eV]
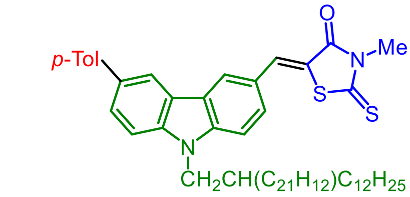 **11a**	1.44	2.419	2.549
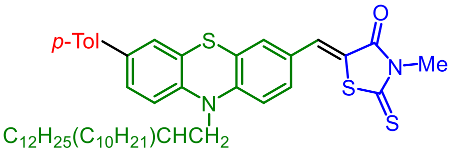 **12a**	1.05	2.212	2.235
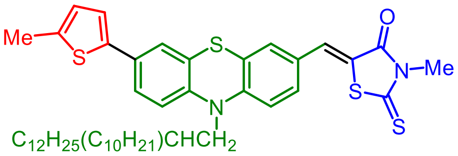 **12n**	1.03	2.129	2.254
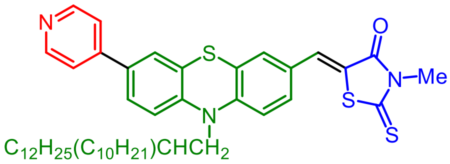 **12j**	1.17	1.812	2.290
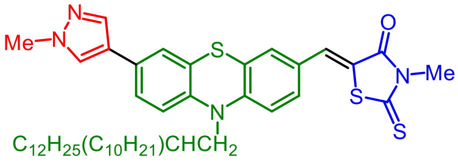 **12k**	1.01	1.858	2.222
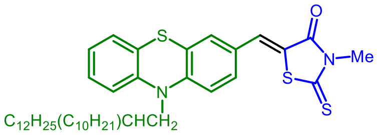 **15**	1.09	1.841	2.281
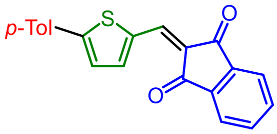 **9b**	1.76	2.359	2.588

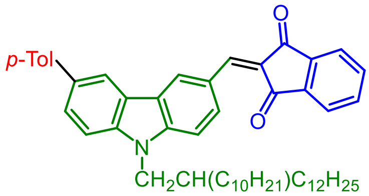 **11c**	1.46	1.895	2.443
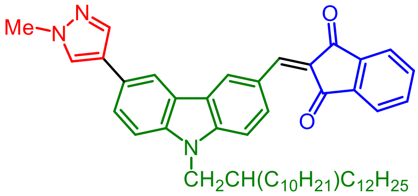 **11g**	1.34	1.939	2.393
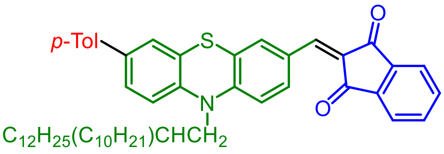 **12c**	1.05	1.951	2.118
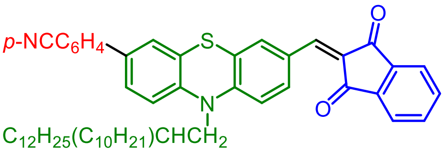 **12i**	1.13	1.880	2.160
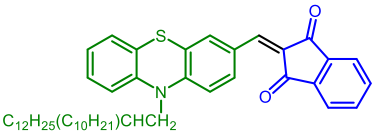 **14**	1.10	1.933	2.154
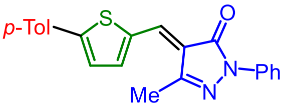 **9d**	1.61	2.364	2.764
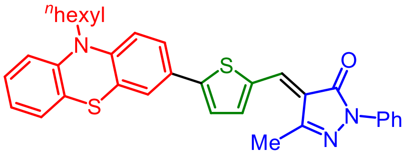 **9g**	0.93	2.224	2.294
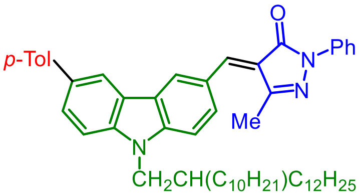 **11d**	1.45	2.140	2.629
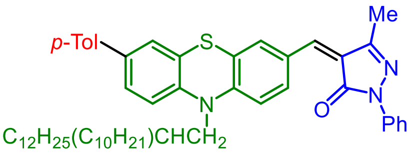 **12e**	1.05	2.140	2.246
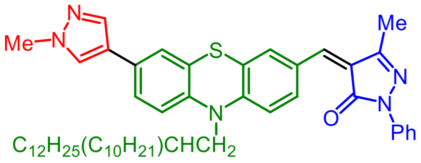 **12m**	1.00	1.889	2.279

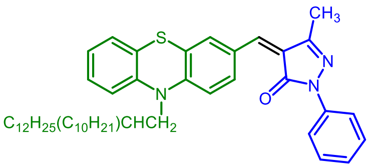 **17**	1.09	2.196	2.273
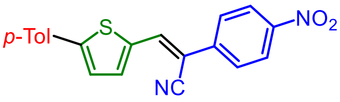 **9e**	1.71	2.580	2.785
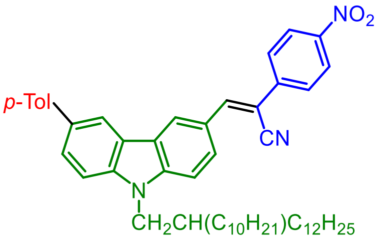 **11e**	1.44	2.055	2.552
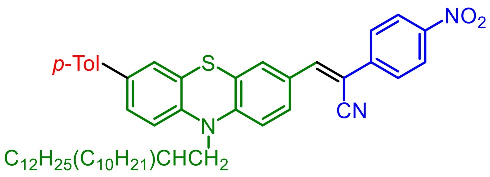 **12f**	1.03	2.359	2.384
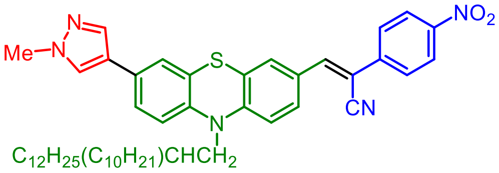 **12l**	1.03	1.945	2.379
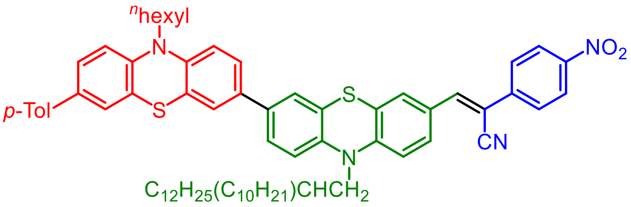 **12y**	0.85	2.021	2.195
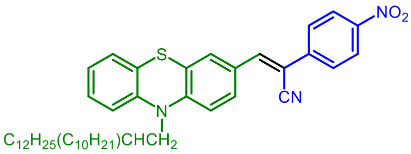 **16**	1.08	1.939	2.441

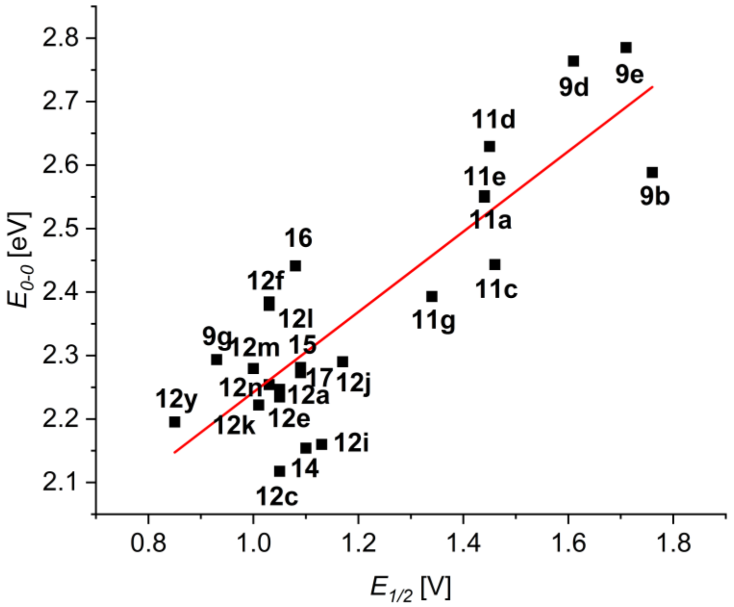

Figure S21. Correlation $E_{0\text{-}0}$ vs $E_{1/2}$: $E_{0\text{-}0}$ = 1.60975 + 0.63246 · $E_{1/2}$ [eV]; r^2 = 0.73381.

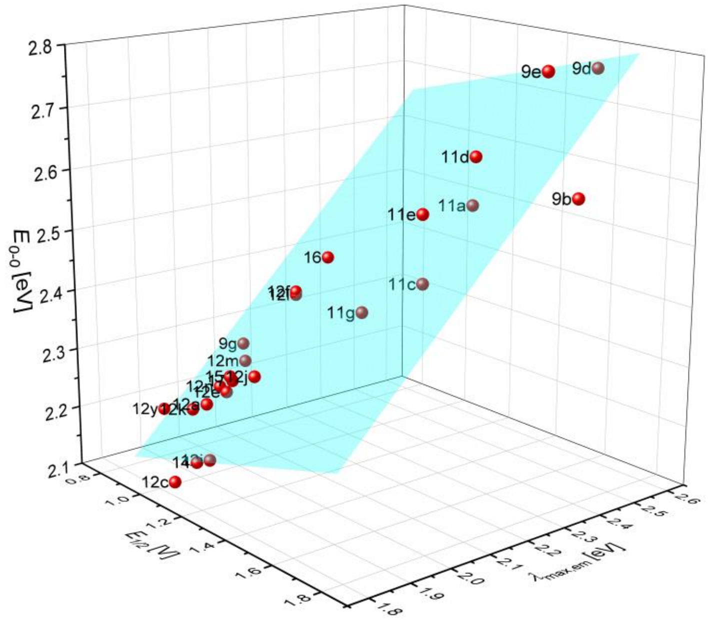

Figure S22. Correlation $E_{0\text{-}0}$ vs $E_{1/2}$ and $\lambda_{\text{max,em}}$: $E_{0\text{-}0}$ = 0.147 · $E_{1/2}$ + 0.7019 · $\lambda_{\text{max,em}}$ + 0.7296; r^2 = 0.93504.

5. References

[1] a) Krämer, C. S.; Zimmermann, T. J.; Sailer, M.; Müller, T. J. J. Syntheses of Phenothiazinyl Boronic Acid Derivatives - Suitable Starting Points for the Construction of Redox Active Materials. *Synthesis* **2002**, 1163-1170. DOI: 10.1055/s-2002-32527 b) Franz, A. W.; Müller,

T. J. J. Facile Synthesis of Functionalized (Oligo)Phenothiazines via One-Pot Bromine-Lithium Exchange-Borylation-Suzuki-Coupling (BLEBS). *Synthesis* **2008**, 1121-1125. DOI: 10.1055/s-2008-1032118

[2] a) Muschelknautz, C.; Sailer, M.; Müller, T. J. J. Sequential Electrophilic Trapping Reactions for the Desymmetrization of Dilithio(hetero)arenes. *Synlett* **2008**, 845-848. DOI: 10.1055/s-2008-1042911 b) Dostert, C.; Czajkowski, D.; Müller, T. J. J. 2,6-Difunctionalization of N-Substituted Dithienothiazines via Dilithiation. *Synlett* **2014**, *25*, 371-374. DOI: 10.1055/s-0033-1340307 c) Dostert, C.; Müller, T. J. J. A one-pot dilithiation–lithium–zinc exchange–Negishi coupling approach to 2,6-di(hetero)aryl substituted dithienothiazines – a novel class of electronically fine-tunable redox systems. *Org. Chem. Front.* **2015**, *2,* 481-491. DOI: 10.1039/C5QO00046G

[3] Meyer, T.; Ogermann, D.; Pankrath, A.; Kleinermanns, K.; Müller, T. J. J. Phenothiazinyl Rhodanylidene Merocyanines for Dye-Sensitized Solar Cells. *J. Org. Chem.* **2012**, *77*, 3704-3715. DOI: 10.1021/jo202608w

[4] Kofler, L. Identifizierung organischer Substanzen. *Sci. Pharm*. **1966**, *2*, 147-166.
